# Nuclear hormone receptors control fundamental processes of human fetal neurodevelopment: Basis for endocrine disruption assessment

**DOI:** 10.1016/j.envint.2025.109400

**Published:** 2025-03-20

**Authors:** Katharina Koch, Kevin Schlüppmann, Saskia Hüsken, Louisa Merit Stark, Nils Förster, Stefan Masjosthusmann, Jördis Klose, Arif Dönmez, Ellen Fritsche

**Affiliations:** a IUF – Leibniz Research Institute for Environmental Medicine, Duesseldorf, Germany; b DNTOX GmbH, Duesseldorf, Germany; c Bioinformatics Group, Faculty for Biology and Biotechnology, Ruhr-University Bochum, Germany; d Center for Protein Diagnostics (ProDi), Ruhr-University Bochum, Bochum, Germany; e Medical Faculty, Heinrich-Heine-University, Duesseldorf, Germany; f SCAHT – Swiss Centre for Applied Human Toxicology, Basel, Switzerland; g Department of Pharmaceutical Sciences, University of Basel, Basel, Switzerland

**Keywords:** Developmental neurotoxicity, Endocrine disruption, EDC, Neural progenitor cells, Neurons, Oligodendrocytes, New approach methodologies

## Abstract

Despite growing awareness of endocrine disrupting chemicals (EDCs), knowledge gaps remain regarding their effects on human brain development. EDC risk assessment focuses primarily on EATS modalities (estrogens, androgens, thyroid hormones, and steroidogenesis), overlooking the broader range of hormone receptors expressed in the developing brain. This limits the evaluation of chemicals for their potential to cause endocrine disruption-mediated developmental neurotoxicity (ED-DNT). The Neurosphere Assay, an in vitro test method for developmental neurotoxicity (DNT) evaluation, is an integral component of the DNT in vitro testing battery, which has been used to screen a broad domain of environmental chemicals. Here, we define the endocrine-related applicability domain of the Neurosphere Assay by assessing the impact and specificity of 14 hormone receptors on seven key neurodevelopmental processes (KNDPs), neural progenitor cell (NPC) proliferation, migration of radial glia, neurons, and oligodendrocytes, neurite outgrowth, and differentiation of neurons and oligodendrocytes. Comparative analyses in human and rat NPCs of both sexes revealed species- and sex-specific responses. Mechanistic insights were obtained through RNA sequencing and agonist/antagonist co-exposures. Most receptor agonists modulated KNDPs at concentrations in the range of physiologically relevant hormone concentrations. Phenotypic effects induced by glucocorticoid receptor (GR), liver X receptor (LXR), peroxisome proliferator-activated receptor beta/delta (PPARβδ), retinoic acid receptor (RAR) and retinoid X receptor (RXR) activation were counteracted by receptor antagonists, confirming specificity. Transcriptomics highlighted receptor crosstalk and the involvement of conserved developmental pathways (e.g. Notch and Wnt). Species comparisons identified limited concordance in hormone receptor-regulated KNDPs between human and rat NPCs. This study presents novel findings on cellular and molecular hormone actions in human fetal NPCs, highlights major species differences, and illustrates the Neurosphere Assay’s relevance for detecting endocrine MoAs, supporting its application in human-based ED-DNT risk assessment.

## 1. Introduction

Human brain development is orchestrated by complex interactions of growth factors and developmental pathways that balance proliferative and differentiation processes. This unique state of high plasticity, which is crucial for key neurodevelopmental processes (KNDPs) such as neural progenitor cell (NPC) proliferation, migration, neuro- and gliogenesis, and circuit formation (Silbereis et al., 2016), renders the developing brain highly sensitive to exogenous noxae causing developmental neurotoxicity (DNT) (Rice and Barone, 2000). There is increasing evidence that hormones such as thyroid hormones, sex steroids, and retinoids regulate KNDPs, with perturbations in hormone levels and hormone receptor signaling causing endocrine disruption (ED)-mediated DNT (Cediel-Ulloa et al., 2022; Gore et al., 2014; Jansen et al., 2019; McCaffery et al., 2003; Sauer et al., 2020). ED-mediated disruption of neurodevelopmental patterns might contribute to neurodevelopmental disorders such as attention deficit hyperactivity disorder, autism spectrum disorder, and intellectual disability (Caporale et al., 2022; Gore et al., 2014; Lupu et al., 2020; Rivollier et al., 2019; Vorhees et al., 2018).

Endocrine disrupting chemicals (EDCs) are exogenous substances that interfere with the synthesis, secretion, transport, binding, action, or elimination of physiological hormones. They disrupt growth and development, posing a major health concern by exerting adverse effects on various target tissues, including the brain (Tanner et al., 2020; Waring and Harris, 2005). EDCs include natural compounds such as flavonoids and phytoestrogens, and synthetic chemicals such as polycyclic aromatic hydrocarbons, polychlorinated biphenyls (PCBs), plasticizers (bisphenol A, BPA), phthalates, or brominated flame retardants. Due to their lipophilic nature, many EDCs can cross the placental and blood–brain barriers, accumulate in breast milk, and expose the developing child during critical stages of development. Developmental exposure to EDCs, such as PCBs or phthalates, has been associated with neurodevelopmental disorders including ADHD, learning and memory deficits, and autistic-like behavior (Engel et al., 2009; Guo et al., 2004; Schantz et al., 2003; Schug et al., 2015). Mechanistically, PCBs and phthalates have been linked to thyroid hormone receptor (THR) disruption and alterations in androgen and glucocorticoid levels during pregnancy. However, the precise pathways through which EDCs impact neurodevelopment remain elusive (Araki et al., 2017; Derakhshan et al., 2021; Dickerson et al., 2011; Giera et al., 2011; Zoeller et al., 2000). Moreover, most research on EDC effects has focused on EATS modalities (estrogens, androgens, thyroid hormones, and steroidogenesis), relies heavily on animal models, and provides insufficient mechanistic information about the interplay between endocrine signaling and neurodevelopment (Lupu et al., 2020; Schug et al., 2015). These gaps result in significant uncertainties regarding the consequences of perinatal EDC exposure on human brain development. This discrepancy is also reflected in the EDC risk assessment process: Regulatory testing frameworks, such as the OECD Conceptual Framework for Testing and Assessment of EDCs, include mechanistic in vitro assays and in vivo studies, but are limited to EATS modalities, neglecting the complexity of the endocrine system and its broader range of hormone receptors (Grignard et al., 2020; LeBaron et al., 2014; OECD, 2018a). The Developmental Neurotoxicity Study (OECD TG 426) and the DNT cohort in the Extended One-Generation Reproductive Toxicity Study (EOGRTS, OECD TG 443), are not designed to detect endocrine modes of action (MoAs), fail to account for species differences in brain development and EDC metabolism, and lack mechanistic insights (OECD, 2018b, 2007). Recognizing the socio-economic implications of ED-DNT, the Horizon 2020 research and innovation action ENDpoiNTs addressed these knowledge gaps by generating knowledge about the causal links between hormone receptor signaling and KNDPs to develop innovative ED-DNT testing strategies (Bellinger, 2012; Cediel-Ulloa et al., 2022; Lupu et al., 2020). Due to species-specific differences in brain development (Borrell, 2019; Borrell and Götz, 2014; Florio and Huttner, 2014; Rice and Barone, 2000) and endocrine function (Cooke et al., 1998; Medvedev et al., 2020; Walter et al., 2019; Whitten and Patisaul, 2001), human-based New Approach Methodologies (NAMs) are crucial for improving ED-DNT assessment (Bal-Price et al., 2018b; Fritsche et al., 2018; NRC, 2007; Ramhøj et al., 2023; Sachana et al., 2021, 2019).

In this study, we explored the causal links between hormone receptor signaling and human fetal brain development and characterized the ED-related applicability domain of the Neurosphere Assay, a mechanistically validated DNT assay (Baumann et al., 2016, 2014; Koch et al., 2022; Masjosthusmann et al., 2019, 2018) based on primary fetal human NPCs. The assay models seven KNDPs in vitro: NPC proliferation (NPC1), migration of radial glia, neurons, and oligodendrocytes (NPC2a−c), neuronal differentiation (NPC3), neurite outgrowth (NPC4a+b), and oligodendrocyte differentiation (NPC5). As an integral part of the DNT in vitro battery (DNT IVB) (Koch et al., 2022; OECD, 2023), the Neurosphere assay has been used to evaluate the DNT hazard potential of flame retardants (Klose et al., 2022b, 2021; Schreiber et al., 2010), Chinese herbal medicines (Klose et al., 2022a), and more than 300 compounds, the majority of which have environmental relevance, including various EDCs from chemical sets compiled by the US EPA, the EFSA, OECD working groups, and the NIEHS (Blum et al., 2023; Masjosthusmann et al., 2020). However, while 32 EDCs have been evaluated (Sup. Data 5), including bisphenols, PFAS, and phthalates, putative molecular mechanisms underlying observed phenotypic effects remain enigmatic. To address this, and investigate EDC MoAs within the applicability domain of the Neurosphere Assay, we analyzed the effects of specific agonists and antagonists of 14 hormone receptors, representing major receptors of the human endocrine system, namely the androgen receptor (AR), aryl hydrocarbon receptor (AhR), estrogen receptor (ER), glucocorticoid receptor (GR), liver X receptor (LXR), peroxisome proliferator-activated receptors (PPARs), progesterone receptor (PR), prostaglandin E2 receptor (PGE2R), retinoic acid receptor (RAR), retinoid X receptor (RXR), THR, and vitamin D receptor (VDR). Incorporating human NPCs from male and female individuals allowed us to investigate sex-specific hormone receptor signaling and its impact on neurodevelopment. To ensure physiological relevance of the in vitro model, we compared hormone receptor expression patterns of human NPCs with fetal human cortical tissue, the origin of our in vitro culture, and linked benchmark concentrations (BMCs) from concentration-response curves to hormone levels in human cord blood. Transcriptome analyses indicated that hormone receptor activation influences key developmental pathways, such as epidermal growth factor receptor (EGFR), Notch, Wnt, and MAPK/ERK signaling. Finally, we compared hormone receptor-regulated KNDPs in human and time-matched rat NPCs, revealing that most fetal KNDPs are regulated by hormone receptors in a species-specific manner.

## 2. Material and methods

### 2.1. Study design

**Fig. 1.**
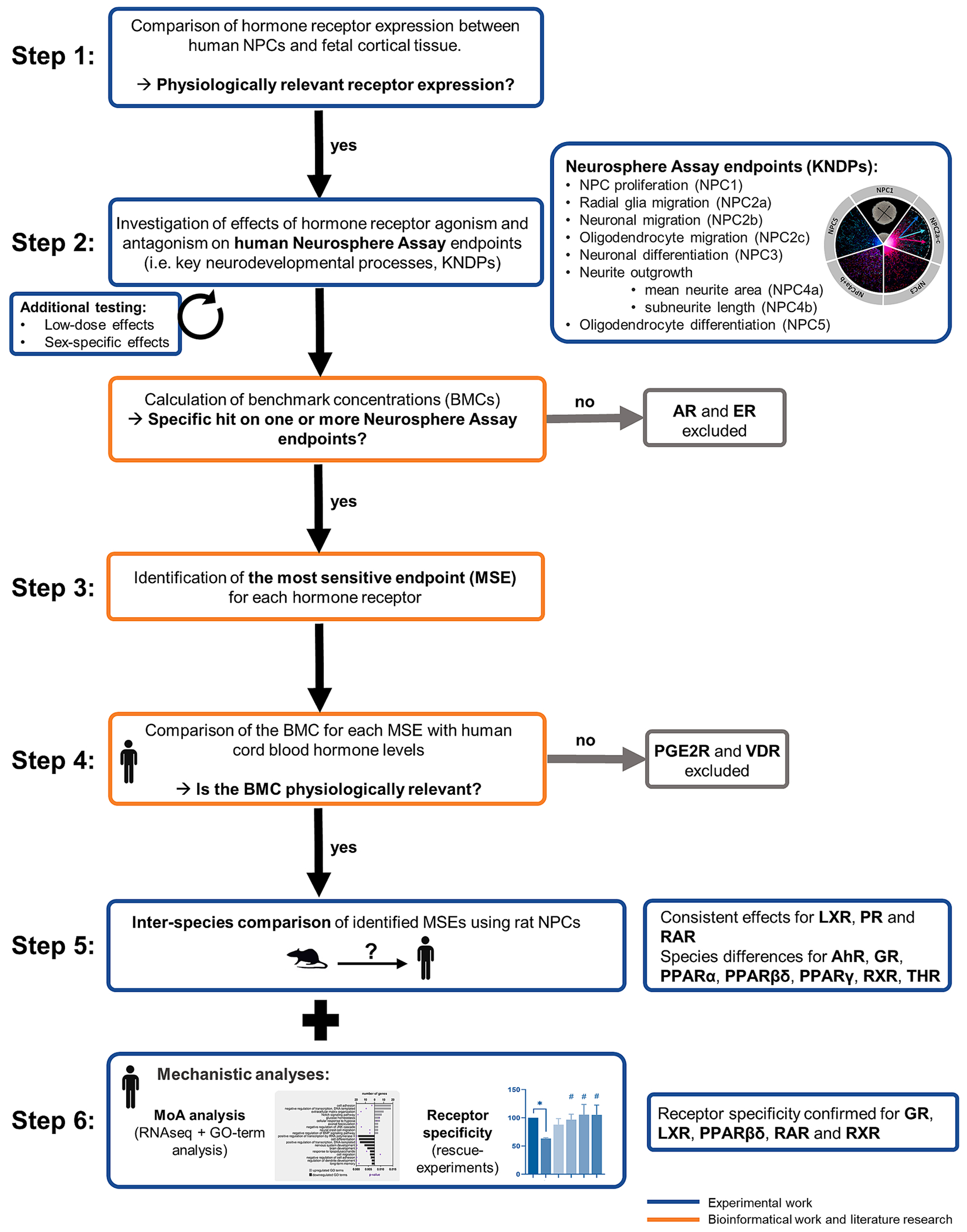
Overview of the study design. Schematic representation of the six-phase study design. (1) Hormone receptor expression in human NPCs was compared to fetal cortical tissue to confirm the model’s physiological relevance. (2) Receptor agonist and antagonist (except PGE2R and VDR) effects on seven KNDPs were assessed in the Neurosphere Assay using male and female NPCs. AR/ER agonism showed no effect. (3) The most sensitive endpoint (MSE) was identified for receptor agonism (except AR/ER). (4) MSE physiological relevance was assessed by comparing benchmark concentrations (BMCs) with fetal cord blood hormone levels, leading to the exclusion of PGE2R and VDR. (5) Species-specific differences were investigated by comparing receptor expression and exposing rat NPCs to receptor agonists. Proliferating rat NPCs were exposed to GR and RAR agonists, while differentiating rat NPCs were exposed to AhR, LXR, PPARs, PR, RXR, and THR agonists. (6) For hormone receptor agonists affecting KNDPs at physiologically relevant concentrations, mechanistic studies were conducted using RNA sequencing and coexposure experiments with the respective receptor antagonists.

This study was conducted in six consecutive phases outlined in Fig. 1. First, hormone receptor expression was compared between human NPCs and fetal human cortical tissue to confirm the physiological relevance of the in vitro model (see 2.3). Second, the effects of hormone receptor agonists and antagonists (Table 1) on seven KNDPs were assessed in the Neurosphere Assay using female and male human NPCs (see 2.4 and 2.5). Cytotoxicity and mitochondrial activity were assessed in parallel (Sup. Figs. 1 and 2), and non-cytotoxic (<10 % cytotoxicity) concentrations were used for concentration–response modeling of KNDP endpoints (see 2.6). All curves and BMCs are provided in Sup. Data 6–8. Antagonism of PGE2R and VDR was not tested since the chemicals were not available. AR and ER agonism showed no effect. Third, the most sensitive endpoint (MSE) was determined for each receptor activation. Fourth, the physiological relevance of the MSEs in human NPCs was assessed by comparing BMCs with cord blood concentrations of natural hormones. PGE2R and VDR were excluded from further analysis due to lack of relevance. Fifth, for the remaining 10 receptors, species-specificities were investigated by comparing receptor expression (see 2.3) and exposing rat NPCs to the same receptor agonists (see 2.4 and 2.5). Since human-relevant MSEs were the focus, proliferating rat NPCs were exposed to GR and RAR agonists, while differentiating rat NPCs were exposed to agonists of AhR, LXR, PPARs, PR, RXR, and THR. Rat NPCs from female and male preparations were investigated separately; no differences were observed. Since neuronal and oligodendrocyte differentiation are multiplexed, both endpoints were evaluated in rat NPCs. All curves and BMCs are provided in Sup. Data 8–10. Sixth, for receptors whose agonists elicited effects on KNDPs at physiologically relevant concentrations (all but AR, ER, PGE2R and VDR), mechanistic studies were performed. RNA sequencing of agonist-exposed (BMC_30_ MSE) male human NPCs was performed to identify genetic signatures underlying the phenotypic observation. Based on the MSE, either proliferating (RAR and GR) or differentiating NPCs (AhR, LXR, PPARs, PR, RXR, THR) were exposed. Additionally, receptor specificity (MSE) was analyzed through co-exposure experiments with agonists and antagonists.

### 2.2. Reagents

(see Table 1).

### 2.3. Comparative hormone receptor expression analysis

Human in vivo – in vitro comparison: Receptor expression in fetal cortex tissue was derived from the BrainSpan atlas (https://www.brainspan.org/), a foundational resource for studying transcriptional mechanisms involved in human brain development (Miller et al., 2014). Expression in different donors was averaged per gestational week (GW8 – 1 donor; GW12 – three donors; GW16 – three donors), if applicable, and for different cortical regions (see Sup. Data 11 for raw RPKM values, donor information, and stratification by brain region). Data were compared to FPKM values from unexposed proliferating and differentiating human NPCs generated within this study (see 2.7). Human – Rat comparison: Microarray datasets for proliferating and differentiating human NPCs (60 h) and rat NPCs (72 h) were previously published and described in detail in (Klose et al., 2021; Masjosthusmann et al., 2018). Data generation and analysis was performed as described in the aforementioned publications.

### 2.4. Basic human and rat neurosphere culture

Primary human NPCs derived from cortical homogenates of male and female fetuses (GW 16–19) were purchased from Lonza Verviers SPRL, Belgium (#PT-2599). Time-matched rat NPCs were prepared as described previously (Baumann et al., 2014; Klose et al., 2021; Masjosthusmann et al., 2019). Briefly, whole brains of post-natal day one (PND1) Wistar rat pups (Charles River) were dissected, enzymatically digested, and homogenized to obtain rat NPC suspensions, with male and female homogenates pooled separately before culture. Rat NPC isolation was approved by the “Landesamt für Natur, Umwelt und Verbraucherschutz” (81–02.05.50.18.001) in accordance with §4 Abs. 3 TierSchG. Human and rat NPCs were cultured as free-floating neurospheres in proliferation medium containing DMEM (#31966–021, Life Technologies) and Ham’s F12 (#31765–027, Life Technologies) in a 2:1 ratio (v:v) supplemented with 2 % B27 (#17504044, Life Technologies), 20 ng/ml EGF (#PHG0313, Thermo Fisher), 100 U/mL penicillin, 100 μg/ml streptomycin (#P06-07100, Pan-Biotech), and either 20 ng/ml human FGF2 for human NPCs (#233-FB, R&D Systems) or 10 ng/ml rat FGF2 for rat NPCs (#3339-FB-025, R&D Systems). Neurospheres were maintained at 37 °C and 5 % CO_2_ in 10 cm diameter cell culture dishes coated with poly-2-hydroxyethyl methacrylate (poly–HEMA; #P3932, Sigma-Aldrich). Weekly, neurospheres were mechanically passaged into 200 μm cubicles using a McIlwain tissue chopper (#TC752, Campden Instruments), and half of the medium was refreshed three times per week.

### 2.5. The Neurosphere Assay (NPC1-5)

#### 2.5.1. Exposure conditions in the Neurosphere Assay

Human NPCs were exposed to seven serial concentrations (serial dilution factor of three, 1:999 for the highest concentration) or solvent (0.1 %) in 96-well plates (five technical replicates per condition) for the whole assay duration. At least three biological replicates were performed, including human NPC donors from both genetic sexes. If sex differences emerged, additional experiments were conducted to generate data from three male and three female donors. If low-dose effects emerged, additional experiments adjusted the concentration range. Rat NPCs were exposed at three concentrations covering the full range of effects observed in human NPCs, using a higher dilution factor (five technical and at least three biological replicates). For co-exposure experiments, four serial dilutions of the receptor antagonist (dilution factor of ten, 1:999 dilution) were prepared in cell culture medium containing the receptor agonist (BMC_30_, 1:999 dilution). Controls included solvent alone and agonist alone (BMC_30_) exposures, with adjustments for agonists and antagonists solvents. Exposures were performed in 96-well plates (five technical replicates per condition).

#### 2.5.2. Endpoint measurement: NPC proliferation (NPC1)

The proliferative capacity of human NPCs and rat NPCs was assessed via bromodeoxyuridine (BrdU) incorporation, as described previously (Koch et al., 2022). Briefly, 300 μm diameter neurospheres were cultured in 100 μl proliferation medium in poly–HEMA-coated 96-well plates for 3 days (five technical replicates with one sphere per condition). Growth factor-free medium served as a positive control for proliferation arrest. BrdU incorporation was measured using the Cell Proliferation ELISA (#11669915001, Roche), according to the manufacturer’s protocol. A detailed NPC1 assay description is available in (Masjosthusmann et al., 2020).

#### 2.5.3. Endpoint measurement: NPC differentiation (NPC2-5)

Spontaneous differentiation of human and rat NPCs was achieved by transferring 300 μm neurospheres into 96-well plates coated with 0.1 mg/mL poly-D-lysine (PDL, #P0899-50MG, Sigma-Aldrich) and 12.5 μg/ml laminin (#L2020-1MG, Sigma-Aldrich). Cells were cultured for five days in differentiation medium consisting of DMEM (#31966–021, Life Technologies) and Ham’s F12 (#31765–027, Life Technologies) (2:1, v:v) supplemented with 1 % N2 (Thermo Fisher, #17502–048) and 100 U/mL penicillin, and 100 μg/ml streptomycin (#P06-07100, Pan-Biotech). During this period, progenitors migrate radially out of the sphere core, form a circular migration area, and differentiate into neurons, astrocytes, and oligodendrocytes. After five days, cells were fixed with 4 % paraformaldehyde for 30 min at 37 °C and immunostained for β(III)tubulin (neurons) and O4 (oligodendrocytes) as described previously (Klose et al., 2022b; Koch et al., 2022). Briefly, nonspecific binding sides were blocked with 10 % goat serum (GS, #G9023-10 mL, Sigma Aldrich) in PBS for 30 min at 37 °C. Cells were incubated overnight at 4 °C with primary antibodies: rabbit anti-β(III)tubulin (1:400, Alexa Fluor^®^ 647-conjugated, #ab190575, Abcam) and mouse anti-O4, (1:400, #MAB1326, R&D Systems) in PBS containing 0.01 % Triton-X and 2 % GS. After three PBS washes, the secondary antibody goat anti-mouse IgM-Alexa Fluor^®^ 488 (1:400, #A-21042, Thermo Fisher) and Hoechst 33258 (1:100, #94403-1ML, Merck) were incubated at 37 ° C for 60 min in PBS containing 2 % GS. After three PBS washes, immunocytochemical images were acquired using the Cellomics ArrayScan HCA platform at 200 × magnification, 552 × 552 pixel resolution, and analyzed with the HCS Studio Cellomics software (version 6.6.3; Thermo Fisher Scientific).

##### 2.5.3.1. Assessment of radial glia (NPC2a), neuronal (NPC2b) and oligodendrocyte migration (NPC2c).

Radial glia migration (NPC2a) was manually assessed at 72 h using brightfield microscopy and automatically at 120 h by analyzing immunocytochemical stainings with the Omnisphero software, as described previously (Schmuck et al., 2017). After 72 h, brightfield images of the entire well were taken with the Cellomics ArrayScan at 50x magnification. The radial distance of the furthest migrated cells to the sphere core was measured in four directions in pixels and converted to μm using the Fiji Image J software (Schneider et al., 2012). After 120 h, radial glia migration was automatically determined by measuring the area of Hoechst 33258-stained nuclei using Omnisphero software. Neuronal (NPC2b) and oligodendrocyte migration (NPC2c) at 120 h were calculated as the mean distance of neurons (β(III)tubulin-positive cells) or oligodendrocytes (O4-positive cells) relative to the radial glia migration distance using Omnisphero software.

##### 2.5.3.2. Assessment of neuronal differentiation (NPC3), neurite outgrowth (NPC4a+b), and oligodendrocyte differentiation (NPC5).

Multiplexed with NPC2a–c, neuronal differentiation (NPC3), neurite outgrowth (NPC4a+b), and oligodendrocyte differentiation (NPC5) were assessed using two convolutional neural networks (CNNs) and the Omnisphero software, as described previously (Förster et al., 2022; Schmuck et al., 2017). Briefly, after immunochemical staining, images were acquired with the Cellomics ArrayScan and merged into a single well image including all three channels: nuclei (Hoechst 33258), neurons (Alexa647^®^), and oligodendrocytes (Alexa488^®^). Nuclei counts were determined using the Spot Detector (V4.1) bio-application in HCS Studio (version 6.6.0, Thermo Fisher Scientific). Neurons and oligodendrocytes were identified using two CNNs in Python 3 (Keras-based) (Chollet, 2017), trained on historical expert-annotated data (Förster et al., 2022). Differentiation was quantified as the percentage of β(III)tubulin-positive (neurons, NPC3) and O4-positive (oligodendrocytes, NPC5) cells relative to the total nuclei count within the migration area. Neurite morphology of all annotated neurons was analyzed by determining the mean neurite area (NPC4a) and total subneurite length (NPC4b), as described in (Schmuck et al., 2017). For rat NPCs, neuronal numbers were quantified using the co-localization tool of the HCS Studio Cellomics software (version 6.6.0, Thermo Fisher Scientific). Oligodendrocyte differentiation in rat NPCs was assessed manually in Omnisphero, normalizing O4-positive cells to the total nuclei count in two defined areas (1098 μm × 823 μm) placed once above and below the sphere core. Percentages from both areas were averaged per sphere, and medians were calculated for five neurospheres per treatment condition.

#### 2.5.4. Endpoint measurement: Mitochondrial activity and cytotoxicity assays

To discriminate specific effects on KNDPs from nonspecific cytotoxicity or reduced viability, cell membrane integrity (CytoTox-ONE Homogeneous Membrane Integrity Assay; #G7891, Promega) and mitochondrial activity (CellTiter-Blue Assay, #G8081, Promega) were assessed in the NPC1 and NPC2–5 assays following chemical exposure. As a positive control, neurospheres were exposed to 0.2 % Triton X-100 for 45 min. Fluorescence was measured using a Tecan Infinite M200 Pro reader (ex: 540 nm; em: 590 nm). Relative fluorescence unit values of replicates were averaged (median) and medium without cells was used to correct for background fluorescence. Notably, reduced NPC proliferation and radial glia migration lower CellTiter-Blue signals due to fewer cells within the neurospheres and the migration area. Thus, the CellTiter-Blue Assay is unsuitable for assessing viability for compounds that inhibit these processes (Klose et al., 2022b).

### 2.6. Statistics and prediction model

Statistical analyses were conducted using the R package CRStats (https://crstats.github.io/; github.com/iuf-duesseldorf/koch-lab-CRStats), which integrates drc (Ritz et al., 2015) and mvtnorm (Genz et al., 2020) with Dunnett and Tamhane’s step-down multiple test procedure (Dunnett and Tamhane, 1991). Data are presented as the mean ± SEM, with significance set at p ≤ 0.05 unless stated otherwise. For concentration–response analysis, customized R packages were used to calculate the benchmark response (BMR) and estimate the BMC, with its confidence interval (BMCL, BMCU). The BMR for all seven Neurosphere Assay endpoints (NPC1–5) was based on the coefficient of variation (CV) of the lowest compound concentration across N = 244 experiments: Endpoint responses of the lowest concentrations were normalized to the respective solvent control, and the BMR was set as CV x 1.5 (rounded to the nearest multiple of 5). BMC_10_ was calculated for cytotoxicity, and all exposure concentrations exceeding this threshold were excluded from further NPC1–5 endpoint analysis. The best curve-fitting model was selected from 13 mathematical concentration–response functions (linear, sigmoidal, monotonic, and non-monotonic) according to robust statistical criteria, assuming continuous response data. Main figures include BMC calculations only for receptor modulator concentrations causing < 10 % cytotoxicity (for all curves see Sup. Figs. 1 and 2, Sup. Data 6, 7, 9 and 10). Statistical significance was assessed using the stepdown multiple test procedure of Dunnett and Tamhane and all data are expressed as mean ± SEM, unless indicated otherwise. A hormone receptor modulator was classified as a “KNDP hit” if at least the highest non-cytotoxic concentration was significantly different from the lowest concentration (assessed via Dunnett and Tamhane step-down multiple test) and/or if the complete 95 % confidence interval of the concentration–response curve intersected the BMR line.

### 2.7. RNA sequencing of human NPCs exposed to hormone receptor agonists

A total of 850 proliferating (GR and RAR) or differentiating (AhR, LXR, PPARα, PPARβδ, PPARγ, PR, RXR, THR) human NPC spheres (100 μm diameter) were exposed to receptor agonists (BMC_30_ for the MSE) or solvent (0.1 % DMSO) for 60 h in poly–HEMA-coated or PDL–laminin-coated 6-well plates. Total RNA was isolated using the RNeasy Mini Kit (#74104, Qiagen) with on-column DNase digests (#79254, Qiagen). Four biological replicates were performed per exposure condition. RNA sequencing was performed by BGI Genomics Co., Ltd. (Hong Kong) and reads were mapped to the human reference genome hg38. Library Preparation: RNA quality was confirmed (RINs ≥ 7) using the Agilent 2100 Bio Analyzer (Agilent RNA 6000 Nano Kit), mRNA was purified, and fragmented using oligo(dT)-labeled magnetic beads. After synthesis of the first and second cDNA strands, end repair and “A” base was added to the 3′-end. Adaptor ligands were added and PCR was performed. PCR products were purified using XP beads, quality-checked, denatured, and circularized by splint oligo-sequencing. The resulting single-stranded circular DNA was formatted as a final library. The library was amplified with phi29 to produce DNA nanoballs (DNB) having more than 300 copies of a molecule. The DNBs were loaded into the patterned nanoarray for sequencing via combinatorial probe anchor synthesis (cPAS), generating 50 bp single-end or 100/150 bp paired-end reads. Data processing involved removing rRNA-mapped reads and filtering low-quality (>40 % of base qualities <20), adaptor-contaminated, or unknown base (N bases > 0.1 %) reads using SOAPnuke software v1.5.2. Clean reads (~20 M clean reads per sample) were saved as FASTQ files and mapped to the human reference genome (GCF_000001405.39_GRCh38.p13) using HISAT2 software v2.0.4. Further analysis included novel transcript prediction (StringTie v1.0.4; Cuffcompare v2.2.1; CPC v0.9-r2), SNP & INDEL calling (GATK), and gene-splicing detection (rMATS v4.0.2). Gene expression analysis was performed by mapping clean reads to the reference genome (Bowtie2 v2.2.5) and calculating expression levels using RSEM (v1.2.12). Differentially expressed genes (DEGs) were identified using DEseq2 (Kong et al., 2007). DEG criteria of |log2(FC)| > 0.486, FPKM ≥ 1 and q-value < 0.05 were used. For target gene analysis, DEGs were screened for target genes that have been described in the literature either for the brain or other organs using a non-systematic literature search. For an unbiased approach, all DEGs were analyzed and significantly enriched GO-terms (p-value < 0.01) were sorted by DEG count. Analyses and visualization were performed using Dr. Tom software (BGI Genomics Co., Ltd.). All DEGs can be found in Sup. Data 13.

## 3. Results

### 3.1. Expression patterns and functional relevance of hormone receptors in proliferating and differentiating human NPCs

**Fig. 2.**
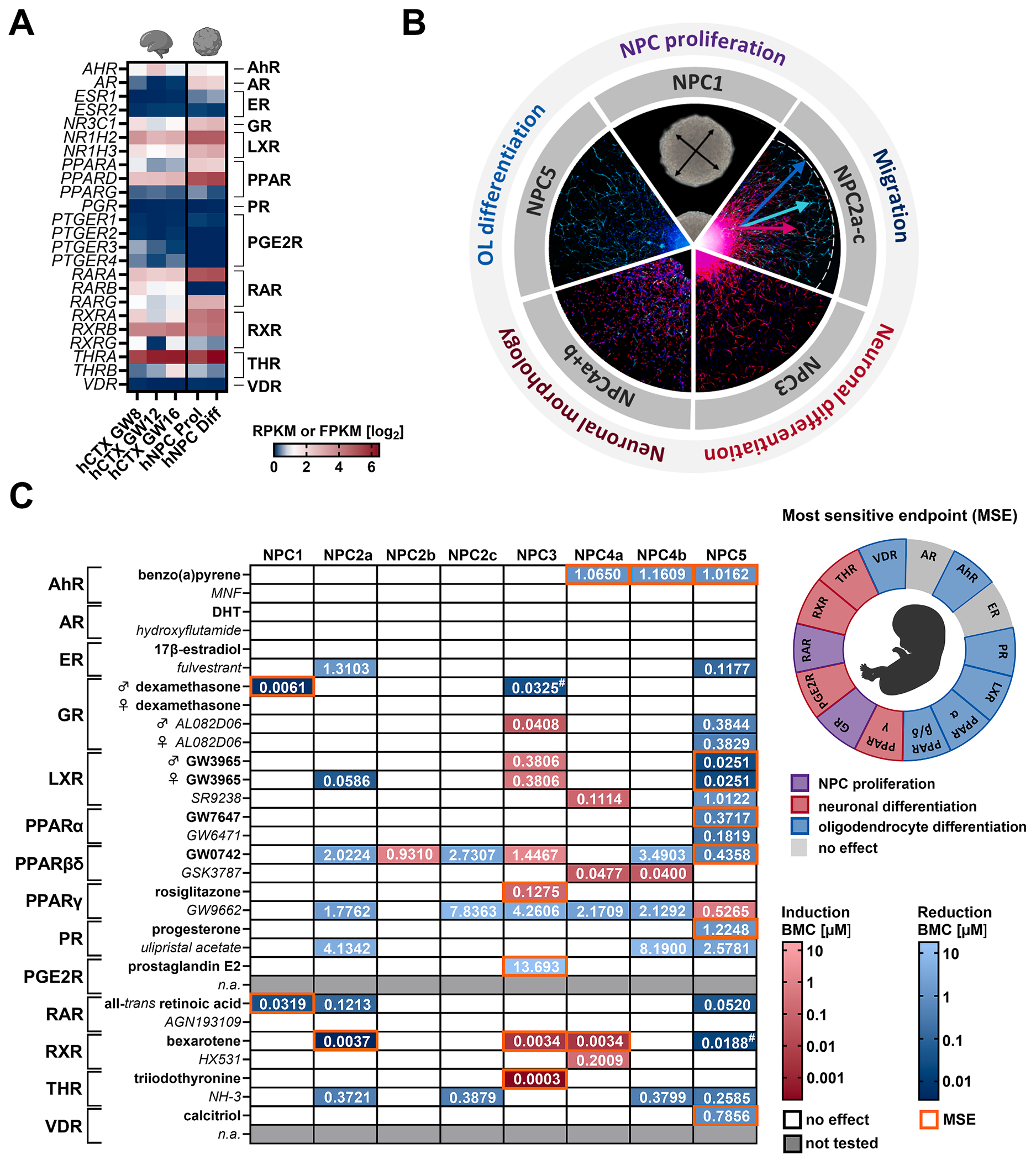
Hormone receptor expression and phenotypic effects of receptor modulation in human NPCs. **A**. Hormone receptor expression in human fetal cortical tissue (hCTX, GW8, 12, and 16) and proliferating (hNPC Prol) and 60 h-differentiated (hNPC Diff) human fetal NPCs (GW16), presented as log2 RPKM (hCTX) and log2 FPKM (hNPC) values. **B**. Overview of Neurosphere Assay endpoints. **C**. Summary of receptor agonist (bold) and antagonist (*italics*) effects on KNDPs modelled in the Neurosphere Assay (NPC1–5), expressed as BMCs (μM). Only effects at non-cytotoxic concentrations are shown. The most sensitive endpoint (MSE) for receptor activation is highlighted in orange; for AhR and RXR, no clear MSE could be defined. ^#^ effects reaching the BMR limit but not significant according to the prediction model. Created with biorender.com. (For interpretation of the references to color in this figure legend, the reader is referred to the web version of this article.)

RNA sequencing revealed highest expression of *THRA, RARA, PPARD, LXRB, RXRA, RXRB, LXRA, RARG*, and *GR* in proliferating (Prol) human NPCs, with *THRA* expression increasing significantly (2.03-fold, p < 0.0001) during differentiation (Diff) (Fig. 2A). In contrast, *ER*, *PR*, *PPARG*, *PGE2R1-4*, *RXRG, THRB* and *VDR* exhibited negligible expression, regardless of the differentiation state. These findings were consistent when comparing the human NPC in vitro model (gestational week 16) to primary fetal cortical tissues at gestational weeks 8, 12, and 16 (Fig. 2A), confirming that the in vitro human NPC model preserves the receptor expression profile of human fetal cortex tissue (Fig. 2A and Sup. Data 11). To evaluate the regulatory role of hormone receptors on fetal KNDPs, proliferating and differentiating human NPCs were exposed to agonists and antagonists of 14 hormone receptors in the human Neurosphere Assay (Fig. 2B and C). The derived KNDP-specific assay endpoints were NPC proliferation (NPC1), radial glia (NPC2a), neuronal (NPC2b) and oligodendrocyte (NPC2c) migration, neuronal differentiation (NPC3), neurite outgrowth (NPC4), and oligodendrocyte differentiation (NPC5). For each receptor, the KNDP with the highest sensitivity to receptor activation (lowest BMC), defined as the most sensitive endpoint (MSE), was identified. Highly expressed receptors, such as *GR*, *LXR*, *PPARD*, *RAR*, *RXR*, and *THR* regulated MSEs at nanomolar or picomolar agonist concentrations, with GR (BMC_20_ = 6.1 nM, male NPCs) and RAR (BMC_20_ = 31.9 nM) primarily affecting NPC proliferation (NPC1), RXR (BMC_25_ = 3.4 nM) and THR (BMC_25_ = 0.26 nM) especially influencing neuron-related KNDPs (NPC3 and 4), and LXR (BMC_25_ = 25.1 nM) and PPARβ/δ (BMC_25_ = 435.8 nM) especially regulating oligodendrocyte differentiation (NPC5). In contrast, receptors with low expression levels, including *PGR* and *PGE2R*, regulated KNDPs only at micromolar concentrations or had no significant impact (AR and ER). Of note, for AhR and RXR, no clear MSE could be defined since several endpoints were affected at similar concentrations, thus all were tested for receptor specificity. To determine the biological relevance of the observed effects, BMCs for the MSE of each receptor were compared to fetal cord blood concentrations of their natural ligands (Table 2). For all hormone receptors except PGE2R and VDR, BMCs were within or below physiological ligand concentrations, indicating the biological relevance of the observed effects on KNDPs. The negligible expression of *PGE2R* and *VDR* and their effects only at non-physiological concentrations suggest receptor-independent effects for PGE2 and calcitriol. Additionally, as AR and ER activation did not affect any KNDP, no further analyses were performed for PGE2R, VDR, AR, and ER.

### 3.2. Species-specific regulation of KNDPs by hormone receptors in human and rat NPCs in vitro

**Fig. 3.**
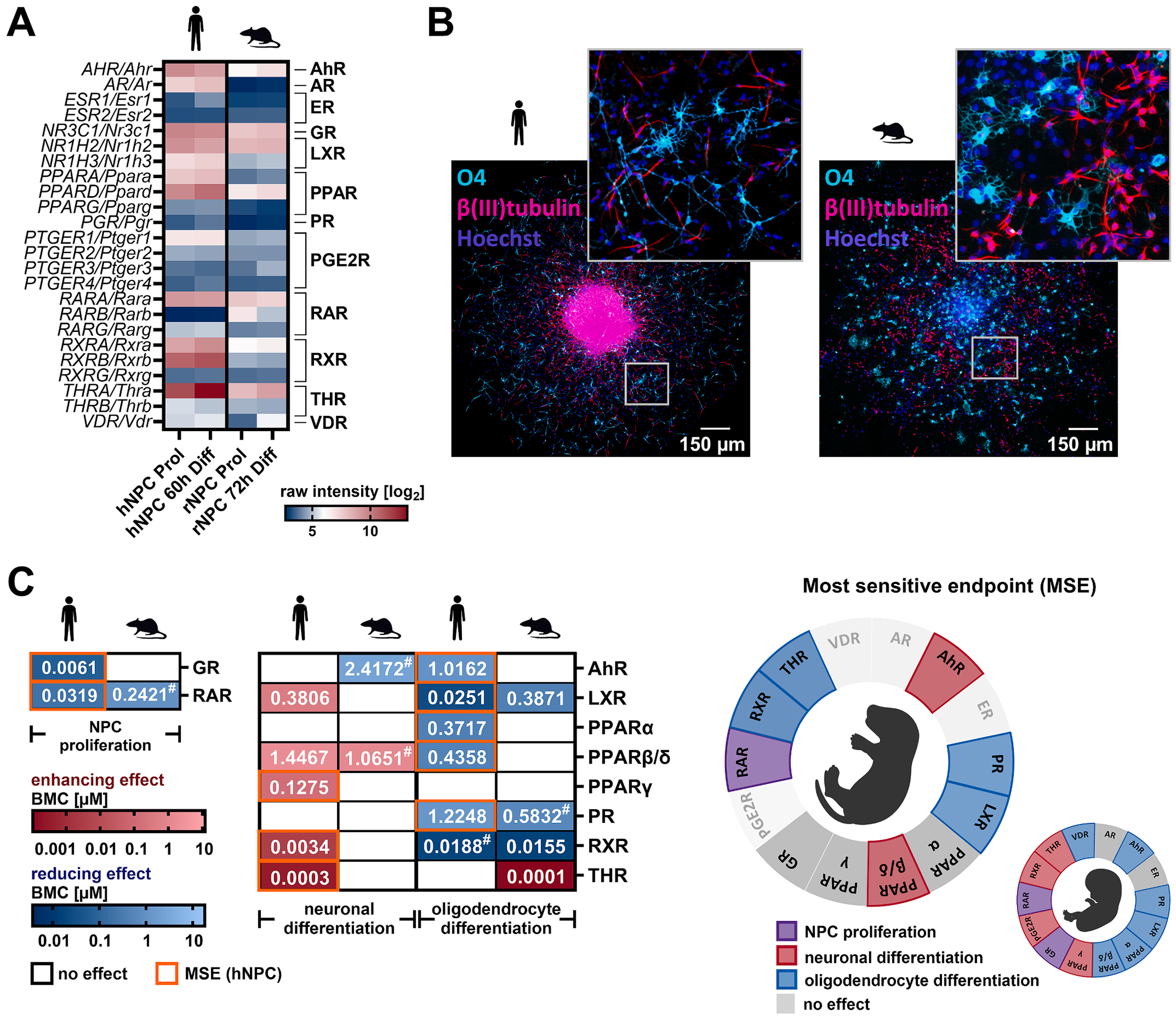
Species-specific effects of neurodevelopmental hormone receptor regulation in human and rat NPCs. **A**. Hormone receptor expression in proliferating (rNPC Prol) and 72 h-differentiated rat NPCs (rNPC 72 h Diff) compared with proliferating (hNPC Prol) and 60 h-differentiated (hNPC 60 h Diff) human NPCs. **B**. Representative immunocytochemical stainings of 5 day-differentiated human and rat NPCs. Neurons were stained with β(III)tubulin, oligodendrocytes with O4 and nuclei with Hoechst 33258. **C**. Proliferating rat NPCs were exposed to GR and RAR agonists or solvent (0.1 % DMSO) for 3 days before BrdU incorporation was measured. Differentiating rat NPCs were exposed to AhR, LXR, PPAR, PR, RXR, and THR agonists or solvent (0.1 % DMSO) for 5 days before neuronal (NPC3) and oligodendrocyte (NPC5) differentiation was measured. Receptor agonist effects in rat NPCs were compared to MSEs derived for human NPCs, expressed as BMCs (μM). Only effects at non-cytotoxic concentrations are shown. AR, ER, PGE2R and VDR activation was not assessed in rat NPCs. Created with biorender.com.

EDCs are primarily evaluated for neurodevelopmental effects using rodent in vivo studies, yet species-specific differences in hormonal regulation raise concerns about their relevance for human health. To address this, we used our previously published primary PND1 rat NPC neurosphere model, which has been extensively characterized in phenomic and transcriptomic studies (Dach et al., 2017; Klose et al., 2021, 2022a; Masjosthusmann et al., 2019, 2018; Walter et al., 2019). To investigate whether hormone receptors regulate the same KNDPs across species, we compared baseline hormone receptor expression in human and rat NPCs and attempted to phenocopy the MSE observed in human NPCs using rat NPCs. Rat NPCs largely recapitulate the receptor expression pattern of human proliferating (Prol) and 3-day differentiated (3d Diff) NPCs, showing high expression of *GR*, *LXRB*, *PPARD*, *RARA*, and *THRA* and low expression of *ER, PPARG, PGR, PTGER1-4, RXRG, THRB*, and *VDR* (Fig. 3A). Morphologically, proliferating human and rat NPC neurospheres appear indistinguishable (Walter et al., 2019), whereas differentiating rat NPCs generate clustered neurons with shorter neurites and oligodendrocytes with shorter processes (Fig. 3B). Functionally, we tested whether MSEs identified in human NPCs (Fig. 2C) were conserved in rat NPCs following hormone receptor agonist exposure (Fig. 3C and Sup. Data 9 and 10). Three receptors (LXR, PR, and RAR) showed conserved neurodevelopmental involvement between species, however, species differences in sensitivity were observed: human NPCs were more sensitive to LXR (BMC_25_: 25.1 nM (h) vs. 387.1 nM (r)) and RAR (BMC_20_: 31.9 nM (h) vs. 242 nM (r, not statistically significant) activation. Both species were equally sensitive to PR activation (BMC_25_: 1.22 μM (h) vs. 0.58 μM (r, not statistically significant)). For PPARβ/δ and RXR activation, a less sensitive endpoint than the MSE was equally affected in both species. PPARβ/δ activation influenced neuronal differentiation, while RXR activation affected oligodendrocyte differentiation in both species. Activation of THR, the highest expressed receptor in both species, revealed species-specific effects at picomolar triiodothyronine (T3) concentrations. While neuronal differentiation (BMC_25_ = 262 pM T3) was promoted in human NPCs, oligodendrocyte differentiation (BMC_25_ = 106 pM T3) was promoted in rat NPCs exposed to T3. For the GR, PPARα, and PPARγ, we observed no effects on the KNDPs assessed in agonist-exposed rat NPCs. In both species, oligodendrocytes were the most sensitive cell type to hormone receptor activation. These findings highlight both conserved and species-specific hormone receptor-dependent mechanisms, emphasizing the importance of human-relevant models for assessing EDC effects on neurodevelopment.

### 3.3. Investigating physiological relevance, receptor specificity, and molecular mechanisms of hormonal receptor activation in human NPCs

To uncover molecular mechanisms underlying the phenotypic effects of hormone receptor activation in human NPCs, and assess receptor specificity, we performed transcriptomic analyses and rescue experiments using receptor antagonists. Based on the identified MSE (Fig. 2C), either proliferating or differentiating human NPCs were exposed to receptor agonists (BMC_30_) for 60 h, followed by RNA sequencing. Gene Ontology (GO) enrichment analyses were performed to identify functional pathways associated with the observed phenotypic effects, further elucidating the molecular mechanisms of hormone receptor activation. Given the sex-specific anti-proliferative effect of GR activation, male human NPCs were used for RNA sequencing analyses. Receptor specificity was assessed by co-exposing hormone receptor agonists (BMC_30_) with their respective antagonists to determine whether the observed phenotypic effects could be reversed. Successful antagonization confirmed receptor-specific effects for GR, RAR, LXR, PPARβδ, and RXR (only NPC3) which are discussed alongside the GO-term analysis in the following sections. In contrast, hormone receptor modulations that did not produce receptor-specific, antagonizable effects, specifically AhR, PPARα, PPARγ, PR, and THR, are presented in the supplementary material (Sup. Figs. 3 and 4), with additional discussion provided in Sup. Data 12.

**Fig. 4.**
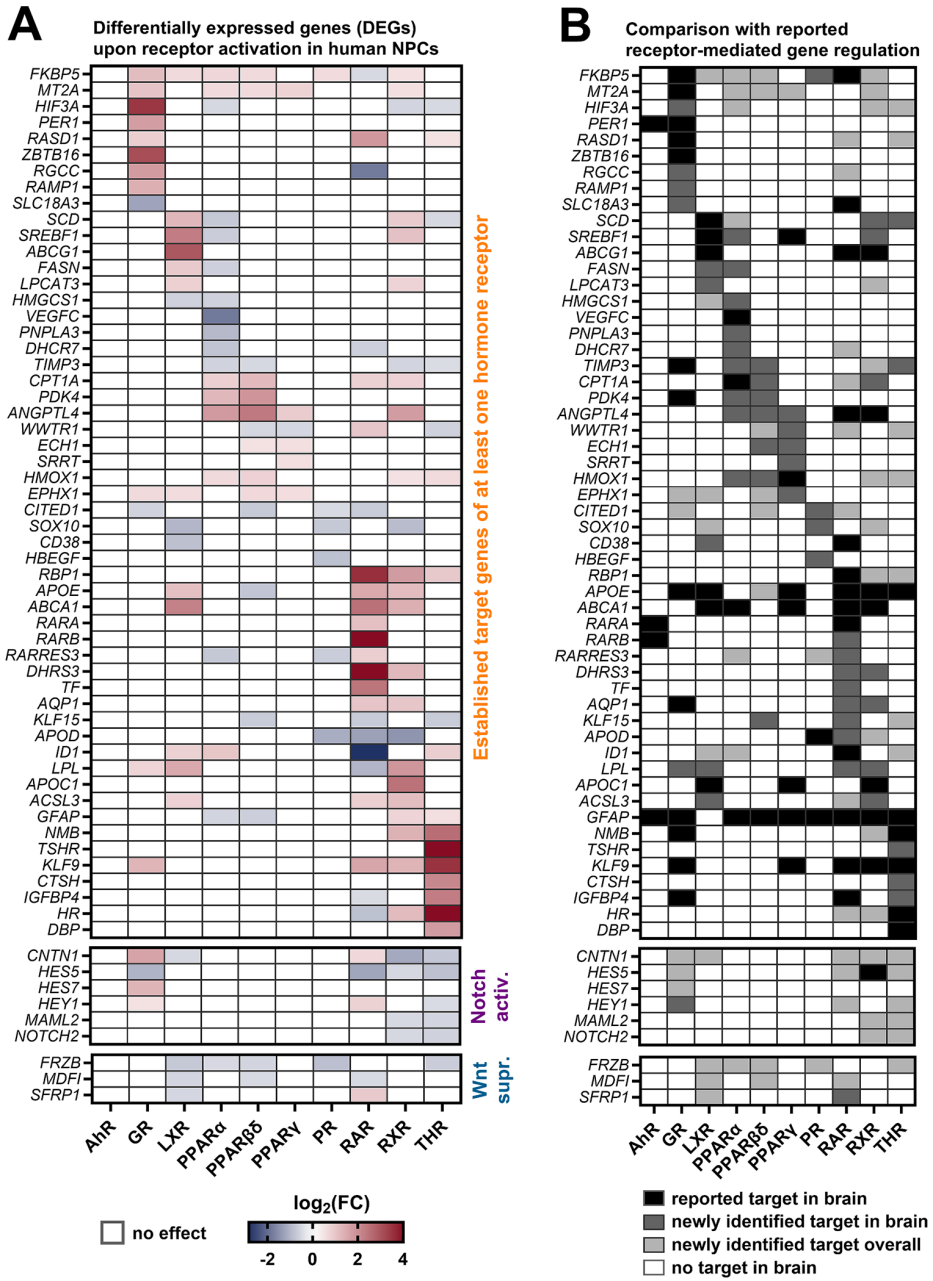
Transcriptomic analysis of hormone receptor target genes regulation in human NPCs. **A**. Proliferating (GR and RAR) and differentiating (AhR, LXR, PPAR, PR, RXR, and THR) human NPCs were exposed to receptor agonists for 60 h (BMC_30_ of MSE) before total RNA was extracted and subjected to RNA sequencing. Genes with a |log2(FC)| > 0.486 and q < 0.05 were defined as DEGs. Presented are genes within all DEGs identified as receptor targets based on a non-systematic literature search. **B**. Literature evidence for hormone receptor-dependent gene regulation (PubMed) distinguishing receptor targets (i) in brain tissue or neuronal cultures (black), (ii) in non-brain tissue or non-neuronal cultures (dark gray), and (iii) genes without evidence of hormone receptor dependency in the literature but in the present study (light gray). Genes with no evidence of hormone receptor-mediated regulation in the brain in the literature or in the present study are highlighted in white. Lists of all DEGs and full reference list are provided in Sup. Data 13 and 14. Created with biorender.com.

To further explore the molecular mechanisms of receptor activation, DEGs in receptor agonist-exposed human NPCs were contextualized within existing literature on hormone receptor-dependent gene regulation. DEGs (Fig. 4A and Sup. Data 13) were compared to previously reported receptor targets (Fig. 4B) using a non-systematic PubMed literature search (Sup. Data 14), incorporating both human and non-human in vivo and in vitro studies. Genes previously identified as receptor targets in brain tissue or neural cultures were labeled ’*reported target in brain*’, while those previously identified as receptor targets in non-brain tissues or non-neural cultures, and newly confirmed as neural targets in this study, were labeled ’*newly identified target in brain*’. Additionally, several genes were identified as receptor targets in human NPCs for the first time (’*newly identified target overall*’). While human NPCs largely recapitulate the published brain-specific hormone receptor target gene pattern, we identified many receptor targets in human NPCs that were previously reported to be under hormone receptor control only in organs other than the brain (e.g., liver, kidney, or adipose tissue). Specific genes will be discussed in the following sections alongside the rescue experiments and GO-term analyses.

#### 3.3.1. Glucocorticoids regulate human NPC proliferation and terminal differentiation in a sex-specific manner

**Fig. 5.**
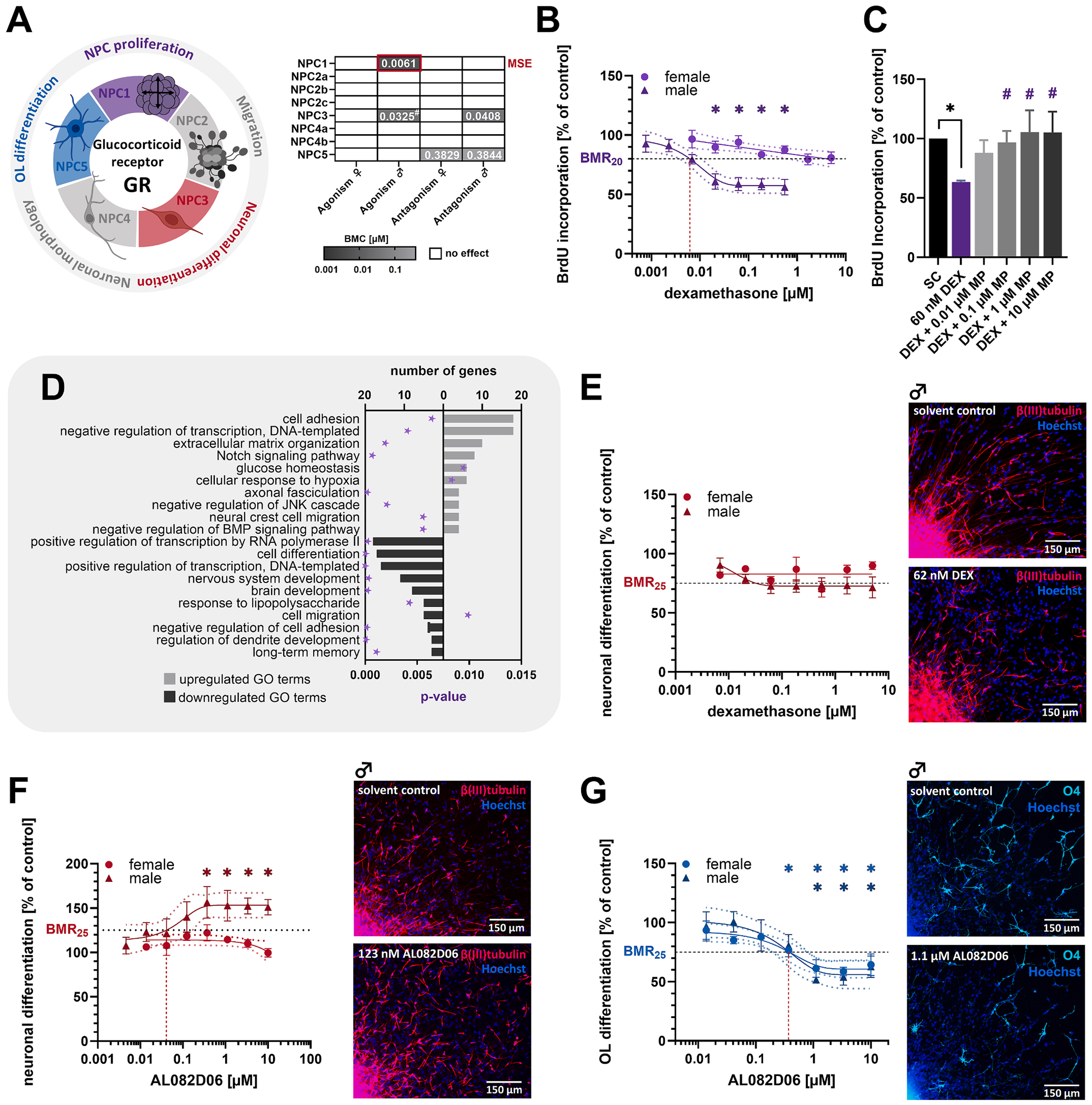
The glucocorticoid receptor regulates human NPC proliferation and differentiation in a sex-specific manner. **A**. GR-sensitive KNDPs (color) and respective BMCs (μM) derived from Fig. 2C. **B**. Proliferating human NPCs were exposed to the GR agonist dexamethasone (DEX) or solvent (0.1 % DMSO) for 3 days before the proliferative capacity was assessed. Data were stratified by genetic sex. **C**. Male proliferating human NPCs were exposed to solvent (0.2 % DMSO), 60 nM DEX (BMC_30_), or DEX in combination with the GR antagonist mifepristone (MP, 0.01 – 10 μM) for 3 days before the proliferative capacity was assessed. *Significant (p < 0.05, two-tailed Student *t*-test) compared to the solvent control. #Significant (p < 0.05, Dunnett and Tamhane) compared to 60 nM DEX. **D**. Top ten GO terms with the most upregulated (light gray) and downregulated (dark gray) DEGs in proliferating human NPCs exposed to 60 nM DEX (BMC_30_) for 60 h. Significant enrichment of DEGs in GO terms was defined by p < 0.01 (purple star). DEGs in GO terms are provided in Sup. Data 15. **E-G**. Differentiating male and female human NPCs were exposed to solvent (0.1 % DMSO) and either DEX (E) or the GR-specific antagonist AL082D06 (F + G) for 5 days before neuronal differentiation (E + F) or oligodendrocyte differentiation (G) was assessed. For B, E, F and G: *significant (p < 0.05, Dunnett and Tamhane) compared to the lowest concentration. Data (B, C, E-G) are expressed as mean ± SEM. Created with biorender.com. (For interpretation of the references to color in this figure legend, the reader is referred to the web version of this article.)

Glucocorticoids (GCs) and their receptor are indispensable for brain development, with over-stimulation linked to neurodevelopmental disorders (Buss et al., 2012; Miranda and Sousa, 2018). While cortisol is the major GR ligand in humans, we used the more stable synthetic GC dexamethasone (DEX). GR activation significantly reduced human NPC proliferation in male (56 % of controls), but not female, NPCs (MSE, BMC_20_ = 6.1 nM DEX, Fig. 5A and B). Given human cord blood cortisol levels (179–254 nM, Table 2) and the higher potency of DEX to activate the GR (He et al., 2014), the observed effects are physiologically relevant. Proliferation of DEX-treated male human NPCs was concentration-dependently restored by co-exposure to the GR antagonist mifepristone (0.1 μM MP), underscoring receptor specificity (Fig. 5C). Co-exposure to the GR-specific, but weaker, antagonist AL082D06 (10 μM AL08) was less effective (data not shown). Transcriptomics of proliferating male human NPCs exposed to 60 nM DEX (BMC_30_) revealed 438 DEGs, including upregulation of proliferation suppressors *FKBP5*, *MT2A*, *PER1*, *RASD1*, and *ZBTB16*, reported GR targets (Fig. 4). GO-term analysis revealed suppression of DNA and RNA transcription and activation of the Notch pathway, a negative regulator of neuronal differentiation (Fig. 5D, Sup. Data 15). Consistent with the transcriptomics, GR activation impaired neuronal differentiation in male but not female human NPCs to 72 % of controls (BMC_25_ = 32.5 nM DEX, Fig. 5E), while GR inhibition enhanced neuronal differentiation to 152 % of controls upon GR inhibition (BMC_25_ = 40.8 nM AL082D06, Fig. 5F). Due to low-dose effects (90 % of control at 6.8 nM DEX), the results of DEX exposure on neuronal differentiation were not statistically significant in this study, but were in a previous study on human NPCs (Moors et al., 2012). Additionally, GR inhibition reduced oligodendrocyte differentiation to 50 % of controls (BMC_25_ = 38.3 nM AL082D06) in both sexes (Fig. 5G).

#### 3.3.2. Liver X receptor activation impairs oligodendrocyte differentiation, alters lipid metabolism, and affects neuronal development in human NPCs

**Fig. 6.**
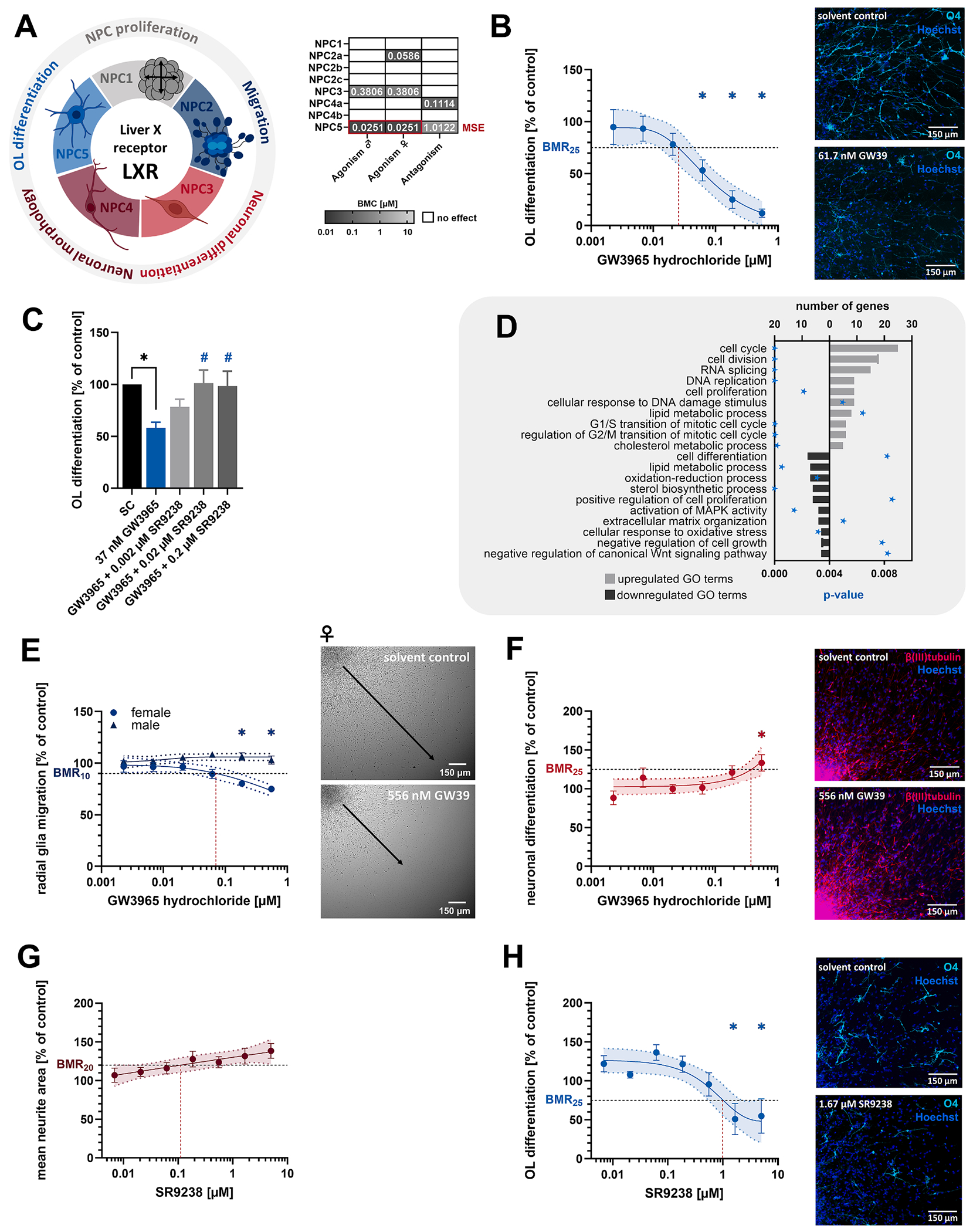
The liver X receptor controls multiple KNDPs, including differentiation of neurons and oligodendrocytes and radial glia migration. **A**. LXR-sensitive KNDPs (color) and respective BMCs (μM) derived from Fig. 2C. **B**. Differentiating human NPCs were exposed to the LXR agonist GW3965 (GW39) or solvent (0.1 % DMSO) for 5 days before oligodendrocyte differentiation was assessed. **C**. Differentiating human NPCs were exposed to solvent (0.2 % DMSO), 37 nM GW39 (BMC_30_), or GW39 in combination with the LXR antagonist SR9238 (SR92, 0.002 – 0.2 μM) for 5 days before oligodendrocyte differentiation was assessed. *Significant (p < 0.05, two-tailed Student *t*-test) compared to solvent control (SC). #Significant (p < 0.05, Dunnett and Tamhane) compared to 37 nM GW39. **D**. Top ten GO terms with the most upregulated (light gray) and downregulated (dark gray) DEGs in differentiating human NPCs exposed to 37 nM GW39 (BMC_30_) for 60 h. Significant enrichment of DEGs in GO terms was defined by p < 0.01 (blue star). DEGs in GO terms are provided in Sup. Data 15. **E**. Differentiating human NPCs were exposed to GW39 or solvent (0.1 % DMSO) for 5 days before assessing radial glia migration. Data were stratified by genetic sex. **F-H**. Differentiating human NPCs were exposed to solvent (0.1 % DMSO) and either GW39 (F) or SR92 (G + H) for 5 days before neuronal differentiation (F), the mean neurite area (G), or oligodendrocyte differentiation (H) was assessed. For B, E-H: *significant (p < 0.05, Dunnett and Tamhane) compared to the lowest concentration. Data (B, C, E-H) are expressed as mean ± SEM. Created with biorender.com. (For interpretation of the references to color in this figure legend, the reader is referred to the web version of this article.)

The LXR is the master regulator of cholesterol homeostasis in the brain, with oxysterols and cholestenoic acid being its major endogenous ligands (Courtney and Landreth, 2016). LXR activation with the synthetic agonist GW3965 concentration-dependently impaired oligodendrocyte differentiation to 12 % of controls (MSE, BMC_25_ = 25.1 nM, Fig. 6A and B). Given that human cord blood levels of natural LXR ligands 24S-hydroxycholesterol (86.9–153.9 nM) and 27-hydroxycholesterol (52.2–96.9 nM) fall within the range of the derived BMC (Table 2), the effects are of physiological relevance. Receptor specificity was confirmed by successfully antagonizing GW3965-induced effects with the specific LXR antagonist SR9238 (20 nM SR9238, Fig. 6C). Transcriptomics of differentiating human NPCs treated with 37 nM GW3965 (BMC_30_) revealed 188 DEGs, including reported LXR targets involved in cholesterol and lipid metabolism (Fig. 4). Among these, we observed induction of lipid biosynthesis suppressors and cholesterol efflux promoters (e.g. *ABCA1, ABCG1, APOE*, and *LPL*). GO-term analysis revealed downregulation of lipid metabolism and sterol biosynthesis (including *FAXDC2, FDFT1, HMGCS1, MSMO1, NSDHL, PTGDS, SC5D*, and *SERAC1*), Wnt pathway activation, and MAPK suppression (Fig. 6D, Sup. Data 15). LXR activation further repressed transcription of oligodendrocyte lineage determinants *SOX10* and *CD38*. Functionally, LXR activation impaired radial glia migration of female, but not male, human NPCs to 75 % of controls (BMC_10_ = 58.9 nM, Fig. 6E) and enhanced neuronal differentiation in both sexes to 133 % of controls (BMC_25_ = 380.8 nM, Fig. 6F). Furthermore, blocking basal LXR activity by SR9238 in the absence of GW3965 increased neurite outgrowth to 138 % of controls (BMC_20_ = 111.4 nM, Fig. 6G) and attenuated oligodendrocyte differentiation of human NPCs to 51 % of controls (BMC_25_ = 1.02 μM; Fig. 6H), though at higher concentrations than required to antagonize GW3965.

#### 3.3.3. PPARβδ is the most abundant PPAR subtype in human NPCs, regulating multiple KNDPs similar to LXR and RXR

**Fig. 7.**
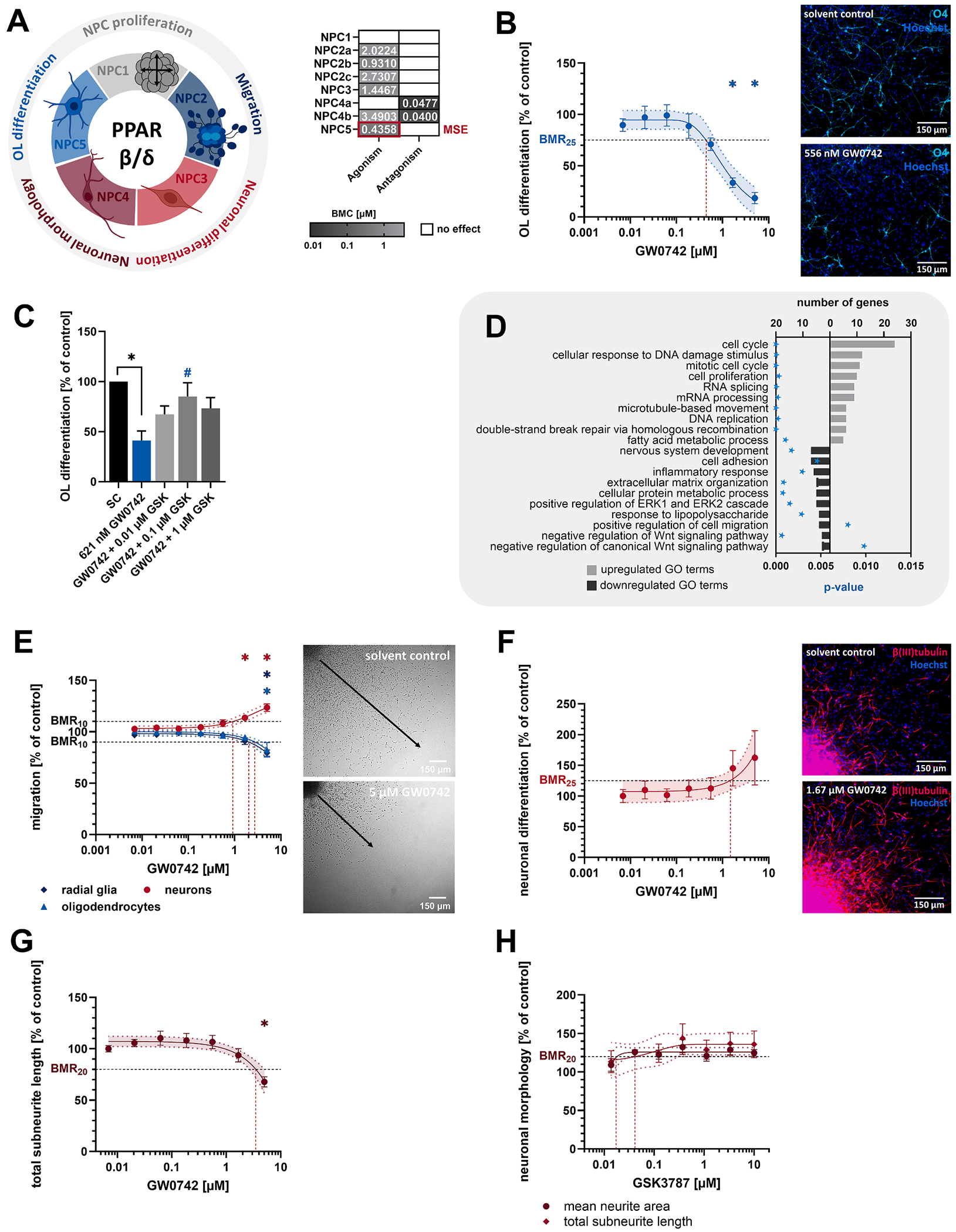
PPARβδ activity affects human NPC lineage specification, resembling LXR and RXR effects. **A**. PPARβδ-sensitive KNDPs (color) and respective BMCs (μM) derived from Fig. 2C. **B**. Differentiating human NPCs were exposed to the PPARβδ agonist GW0742 or solvent (0.1 % DMSO) for 5 days before oligodendrocyte differentiation was assessed. **C**. Differentiating human NPCs were exposed to solvent (0.2 % DMSO), 621 nM GW0742 (BMC_30_), or GW0742 in combination with the PPARβδ antagonist GSK3787 (GSK, 0.01 – 1 μM) for 5 days before oligodendrocyte differentiation was assessed. *Significant (p < 0.05, two-tailed Student *t*-test) compared to the solvent control. #Significant (p < 0.05, Dunnett and Tamhane) compared to 621 nM GW0742. **D**. Top ten GO terms with the most upregulated (light gray) and downregulated (dark gray) DEGs in differentiating human NPCs exposed to 621 nM GW0742 (BMC_30_) for 60 h. Significant enrichment of DEGs in GO terms was defined by p < 0.01 (blue star). DEGs in GO terms are provided in Sup. Data 15. **E**. Differentiating human NPCs were exposed to GW0742 or solvent (0.1 % DMSO) for 5 days before the migration of radial glia, neurons and oligodendrocytes was assessed. **F-H**. Differentiating human NPCs were exposed to solvent (0.1 % DMSO) and either GW0742 (F + G) or GSK (H) for 5 days before neuronal differentiation (F), the mean neurite area (G + H), or total subneurite length (H) were assessed. For B, E-H: *significant (p < 0.05, Dunnett and Tamhane) compared to the lowest concentration. Data (B, C, E-H) are expressed as mean ± SEM. Created with biorender.com. (For interpretation of the references to color in this figure legend, the reader is referred to the web version of this article.)

PPARs are hormone and lipid-activated nuclear receptors that regulate fatty acid, cholesterol, and sphingolipid metabolism, and maintain carbohydrate and glucose homeostasis (Strosznajder et al., 2021). Upon heterodimerization with RXR, they bind a wide range of lipophilic substances including fatty acids and their derivatives, such as prostaglandins (Aleshin and Reiser, 2013). Activation of PPARβδ with the specific agonist GW0742 concentration-dependently reduced oligodendrocyte differentiation to 18 % of controls (MSE, BMC_25_ = 436 nM, Fig. 7A and B). Notably, this effect occurred at agonist concentrations below natural PPARβδ ligand levels in fetal cord blood (> 100 μM total n-6 polyunsaturated fatty acids (PUFAs), Table 2). Receptor specificity was confirmed by antagonizing GW0742-induced effects with the PPARβδ antagonist GSK3787 (100 nM GSK3787, Fig. 7C). Transcriptomics of differentiating human NPCs exposed to 621 nM GW0742 (BMC_30_) revealed 182 DEGs, including several reported PPARβδ target genes (Fig. 4). PPARβδ activation suppressed several genes involved in reactive oxygen species (ROS) scavenging (*TIMP3, WWTR1, APOE*, and *KLF15*, Fig. 4). GO-term analysis revealed upregulation of fatty acid metabolism (*ACACB, ACSBG1, BRCA1, CPT1A*, and *EM1*), Wnt pathway activation, inflammatory response suppression (*CD44, CSF1, FOS, JMJD7-PLA2G4B, NAIP*, and *SELENOP*), MAPK inhibition, and reduced cell adhesion and migration (Fig. 7D and Sup. Data 15). At concentrations higher than those affecting the MSE, PPARβδ activation impaired radial glia migration to 79 % of controls (BMC_10_ = 2.02 μM) and oligodendrocyte migration to 82 % of controls (BMC_10_ = 2.73 μM) In contrast, it enhanced neuronal migration to 123 % of controls (BMC_10_ = 930.7 nM) and neuronal differentiation to 162 % of controls (BMC_25_ = 1.45 μM) (Fig. 7E and F). Further supporting the involvement of PPARβδ signaling in neurogenesis and neuronal maturation, activation reduced the subneurite length to 60 % of controls (BMC_20_ = 3.49 μM), whereas receptor antagonism increased the subneurite length to 136 % of controls (BMC_20_ = 40 nM) and the neurite area to 125 % of controls (BMC_20_ = 47.7 nM) (Fig. 7G and H).

#### 3.3.4. Retinoic acid signaling impairs human NPC proliferation and migration by disrupting transcriptional regulation of the cell cycle and adhesion pathways

**Fig. 8.**
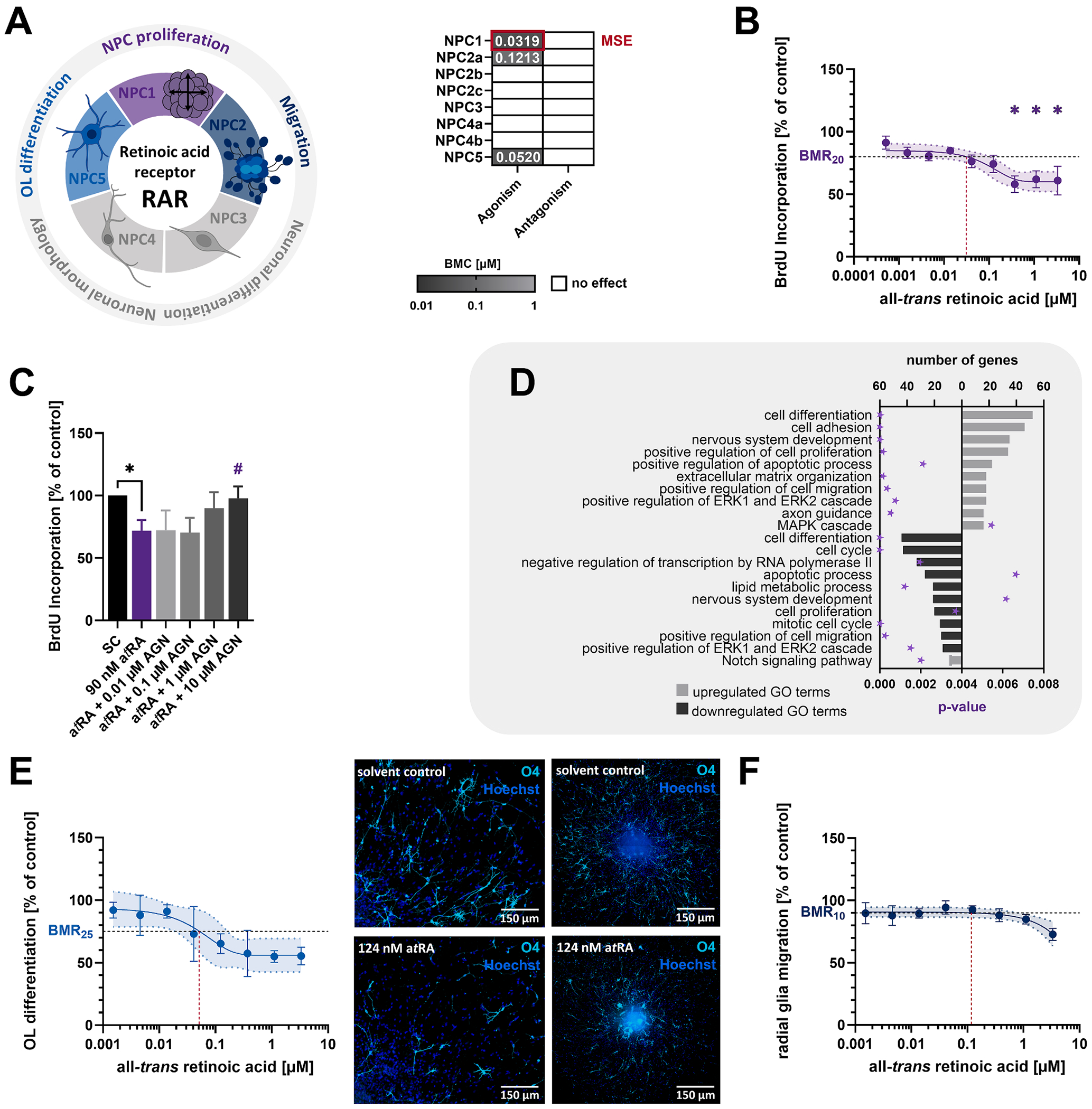
The retinoic acid receptor regulates human NPC proliferation and oligodendrocyte differentiation. **A**. RAR-sensitive KNDPs (color) and respective BMCs (μM) derived from Fig. 2C. **B**. Proliferating human NPCs were exposed to the RAR agonist all-*trans* retinoic acid (a*t*RA) or solvent (0.1 % DMSO) for 3 days before the proliferative capacity was assessed. **C**. Proliferating human NPCs were exposed to solvent (0.2 % DMSO), 90 nM a*t*RA (BMC_30_), or a*t*RA in combination with the RAR antagonist AGN193109 (AGN, 0.01–10 μM) for 3 days before the proliferative capacity was assessed. *Significant (p < 0.05, two-tailed Student *t*-test) compared to the solvent control. #Significant (p < 0.05, Dunnett and Tamhane) compared to 90 nM a*t*RA. **D**. Top ten GO terms with the most upregulated (light gray) and downregulated (dark gray) DEGs in proliferating human NPCs exposed to 90 nM a*t*RA (BMC_30_) for 60 h. Significant enrichment of DEGs in GO terms was defined by p < 0.01 (purple star). DEGs in GO terms are provided in Sup. Data 15. **E + F**. Differentiating human NPCs were exposed to a*t*RA for five days before oligodendrocyte differentiation (E) and radial glia migration (F) were assessed. Representative pictures show the differentiated sphere including a closeup of the migration area for (E). For B, E and F: *significant (p < 0.05, Dunnett and Tamhane) compared to the lowest concentration. Data (B, C, E and F) are expressed as mean ± SEM. Created with biorender.com. (For interpretation of the references to color in this figure legend, the reader is referred to the web version of this article.)

All-*trans* retinoic acid (a*t*RA) and associated RAR signaling are critical for cell differentiation, prefrontal cortex patterning, and neural tube closure (Cunningham and Duester, 2015; Janesick et al., 2015; Li et al., 2018; Shibata et al., 2021). RAR activation impaired NPC proliferation to 58 % of controls (MSE, BMC_20_ = 31.9 nM) at physiologically relevant a*t*RA concentrations (fetal cord blood total RA concentration: 100–550 nM, Table 2) (Fig. 8A and B). Receptor specificity was confirmed by antagonizing a*t*RA-induced anti-proliferative effects with the RAR antagonist AGN193109 (10 μM AGN, Fig. 8C). Transcriptomics of proliferating human NPCs exposed to 90 nM a*t*RA (BMC_30_) revealed 1,083 DEGs, including reported RAR targets (Fig. 4). GO-term analysis revealed MAPK signaling activation, upregulation of genes involved in cell adhesion (46 genes, including cadherins, contactins, collagens, laminins, and integrins), and impairment of cell cycle progression (43 genes) and cell proliferation (20 genes) (Fig. 8D and Sup. Data 15). Functionally, a*t*RA exposure reduced the percentage of oligodendrocytes within the migration area to 55 % of controls (BMC_25_ = 52 nM), but this was due to premature differentiation and immobilization of oligodendrocytes within the sphere core, rather than impaired oligodendrogenesis (Fig. 8E). Moreover, a*t*RA exposure impaired radial glia migration to 73 % of controls (BMC_10_ = 121.3 nM, Fig. 8F).

#### 3.3.5. Retinoid X receptor activity regulates multiple KNDPs, mirroring other nuclear receptors and indicating heterodimer involvement

**Fig. 9.**
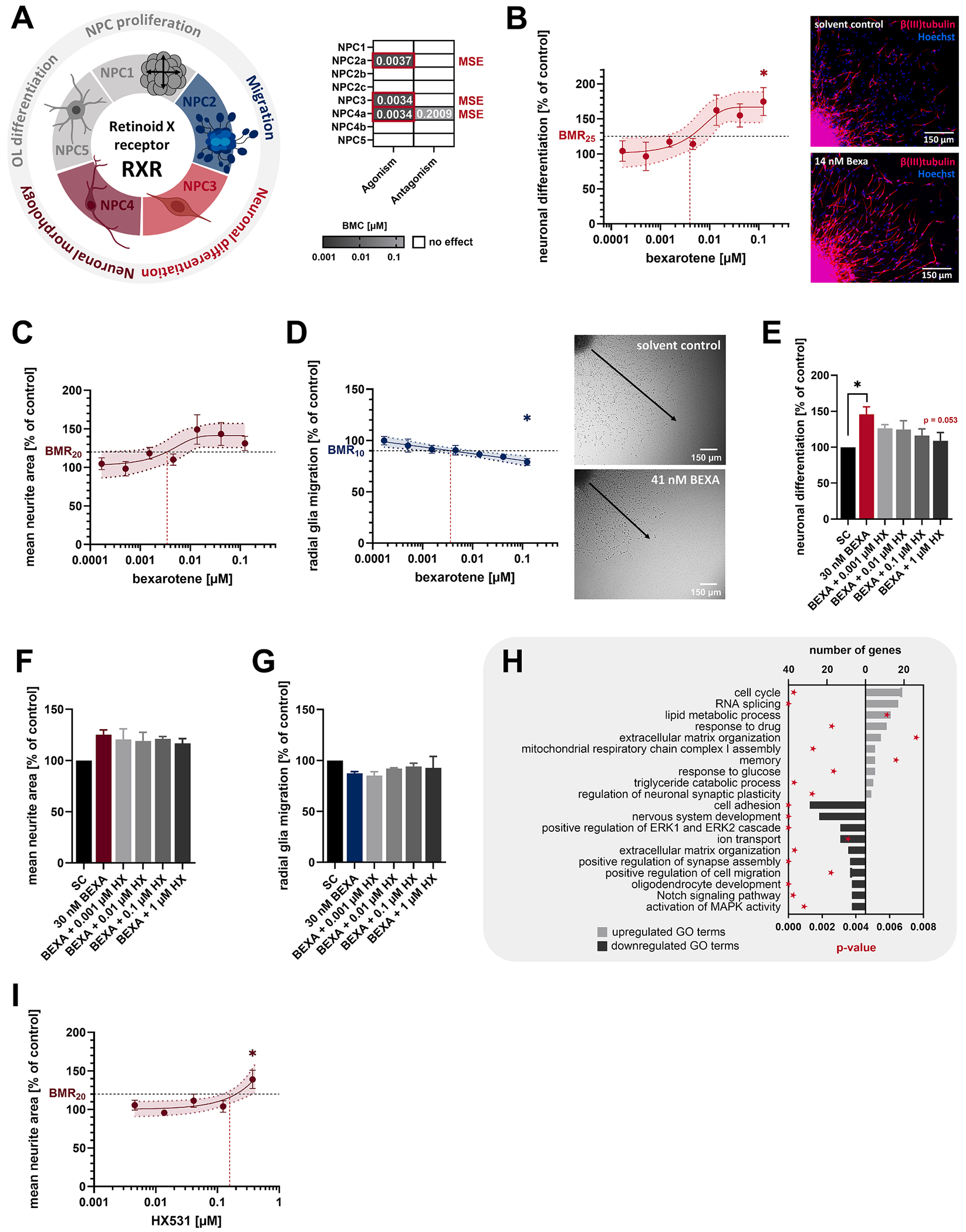
Retinoid X receptor signaling promotes neurogenesis and exhibits transcriptomic similarities to LXR activation. **A**. RXR-sensitive KNDPs (color) and respective BMCs (μM) derived from Fig. 2C. **B-D**. Differentiating human NPCs were exposed to the RXR agonist bexarotene (BEXA) or solvent (0.1 % DMSO) for 5 days before neuronal differentiation (B), the mean neurite area (C), and radial glia migration (D) was assessed. **E-G**. Differentiating human NPCs were exposed to solvent (0.2 % DMSO), 30 nM BEXA (BMC_30_), or BEXA in combination with the RXR antagonist HX531 (HX, 0.001 – 1 μM) for 5 days before neuronal differentiation (E), the mean neurite area (F) and radial glia migration (G) were assessed. *Significant (p < 0.05, two-tailed Student *t*-test) compared to the solvent control (SC). #Significant (p < 0.05, Dunnett and Tamhane) compared to 30 nM BEXA. **H**. Top ten GO terms with the most upregulated (light gray) and downregulated (dark gray) DEGs in differentiating human NPCs exposed to 30 nM BEXA (BMC_30_) for 60 h. Significant enrichment of DEGs in GO terms was defined by p < 0.01 (red star). DEGs in GO terms are provided in Sup. Data 15. **I**. Differentiating human NPCs were exposed to HX531 or solvent (0.1 % DMSO) for 5 days before the mean neurite area was assessed. For B, C, D, and I: *Significant (p < 0.05, Dunnett and Tamhane) compared to the lowest concentration. Data (B-G, I) are expressed as mean ± SEM. Created with biorender.com. (For interpretation of the references to color in this figure legend, the reader is referred to the web version of this article.)

The RXR forms both homo- and heterodimers and binds multiple physiological ligands, including 9-*cis*-retinoic acid (9*c*RA) and dietary docosahexaenoic acid (DHA), a major structural component of the brain (De Urquiza et al., 2000; Gamoh et al., 1999). RXR activation with the synthetic RXR agonist bexarotene (BEXA) affected several KNDPs in differentiating human NPCs at low nanomolar concentrations (Fig. 9A) far below fetal cord blood DHA levels (127–231 μM, Table 2). BEXA exposure induced neuronal differentiation to 176 % of controls (MSE, BMC_25_ = 3.4 nM, Fig. 9B), increased the neurite area to 143 % of controls (MSE, BMC_20_ = 3.4 nM, Fig. 9C) and impaired radial glia migration to 79 % of controls (MSE, BMC_10_ = 3.7 nM, Fig. 9D). While the proneurogenic effect of BEXA was antagonized by the RXR antagonist HX531 (1 μM HX531, Fig. 9E), the effects on neurite outgrowth (Fig. 9F) and radial glia migration (Fig. 9G) were only partially reversed, since HX531 itself impaired radial glia migration (not statistically significant) and increased the neurite area (Fig. 9I) at higher concentrations than BEXA. Transcriptomics of differentiating human NPCs exposed to 30 nM BEXA (BMC_30_) identified 475 DEGs, including known RXR targets involved in lipid metabolism that are also LXR targets (*ABCA1, ACSL3, APOE, LPCAT3, LPL, SCD*, and *SREBF1*, Fig. 4), likely mediated by LXR–RXR heterodimers. Consistent with the phenotypic effects on neurogenesis, GO-term analysis linked RXR activation to Notch pathway suppression and activation of genes involved in memory and neuronal synaptic plasticity (Fig. 9H, Sup. Data 15). Indicative of LXR–RXR heterodimers, RXR activation further induced genes involved in lipid metabolism and downregulated genes involved in oligodendrocyte development (e.g. *SOX10*) and MAPK signaling. Similar to LXR activation, RXR activation reduced oligodendrocyte differentiation to 65 % of controls (BMC_25_ = 19 nM, Sup. Data 7), but the effect was not statistically significant.

#### 3.3.6. Thyroid hormones promote neurogenesis, potentially through RXR–THR heterodimers and Notch pathway suppression

Thyroid hormones are critical for white matter development in humans, primarily through their role in oligodendrocyte maturation (Annunziata et al., 1983; Baas et al., 1997). While we previously established a NAM for detecting EDCs that disrupt thyroid hormone-dependent oligodendrocyte maturation (Dach et al., 2017; Klose et al., 2021), we further investigated if thyroid hormones affect other processes of fetal neurodevelopment. T3 exposure increased neuronal differentiation to 160 % of controls at picomolar concentrations (MSE, BMC_25_ = 262 pM, Sup. Fig. 3I), a physiologically relevant range given fetal cord blood T3 concentrations (0.85–0.92 nM, Table 2). However, the effect could not be antagonized by the THR antagonist NH-3 (Sup. Fig. 3J and K). Transcriptomics of differentiating human NPCs exposed to 3 nM T3 (BMC_30_) revealed 590 DEGs, including reported THR targets (e.g. *DBP*, *HR, KLF9*, and *NMB*, Fig. 4) that we and others have previously associated with oligodendrocyte maturation (Dugas et al., 2012; Klose et al., 2021). Since RXR activation elicits similar effects on developing neurons (Fig. 9B) and THR is a non-permissive partner of RXR, RXR–THR heterodimers may coordinate the observed effects. This is supported by similar repressive effects of THR and RXR activation on the Notch pathway (11 genes) and broad similarities within GO term enrichment analyses (Fig. 9H, Sup. Fig. 3L and Sup. Data 15). THR inhibition with NH-3 impaired several KNDPs in differentiating human NPCs in the absence of exogenous thyroid hormones (Fig. 2C and Sup. Data 7). Since 3 nM T3 is present in the proliferation medium, residual THR activity in differentiating NPCs is conceivable.

## 4. Discussion

A complete picture of how hormones regulate neurodevelopment is essential to evaluate the hazard potential of EDCs. Hormone receptors play crucial roles in neurodevelopmental processes, including sexual differentiation (i.e. AR and ER), neural tube closure (RAR), and white matter development (THR). Perturbations in hormone receptor activities are associated with neurodevelopmental disorders such as cognitive deficits, intellectual disability, or behavioral impairments (Gika et al., 2010; Gupta et al., 1995; López-Espíndola et al., 2014). These consequences illustrate the danger posed by EDC exposure during development, and highlight the need to fill the data gaps on the neurodevelopmental effects of the wide range of hormone receptors expressed in the brain.

Fetal human NPCs recapitulate the hormone receptor expression pattern of human corticogenesis (GW8, 12, and 16) and provide a relevant model to study hormonal dependencies in fetal brain development and neurodevelopmental effects of EDC exposure. Our findings demonstrate that several key EDC MoAs fall within the applicability domain of the Neurosphere Assay, including activation of GR, LXR, PPARβ∂, RAR, RXR and THR. These highest expressed receptors control fetal KNDPs at low nanomolar agonist concentrations, within the range of reported cord blood concentrations of natural receptor ligands. Of note, biomonitoring data on fetal brain hormone levels are scarce, necessitating the use of cord blood concentrations as reference. The value and applicability of the Neurosphere Assay is further supported by the diverse effects observed in previous studies when exposing human NPCs to EDCs (Blum et al., 2023; Klose et al., 2022b). This study mechanistically links putative EDC MoA to those phenotypic observations, reinforcing the Neurosphere Assay’s value for EDC hazard identification without the need for ED-DNT-specific NAMs. AR and ER activation fall outside the assay’s applicability domain. While AR modulation had no effect on any KNDP, only ER inhibition by Fulvestrant influenced KNDPs. Nonetheless, both AR and ER activation are already covered within the existing EDC risk assessment framework (OECD, 2018a). Moreover, transcriptomics identified several novel hormone receptor targets, supporting the idea that hormone receptor-mediated transcription varies by species and developmental stage (Hunter, 1996; Liu and Brent, 2010), highlighting the need for human-based models for studying ED-DNT.

Fetal human NPCs do not synthesize GR, RAR, or THR ligands, since they do not express key steroidogenesis (e.g. *CYP11B1*, *CYP11A1*, *CYP17A1*, *HSD3B*) and retinoid synthesis (e.g. *ALDH1A1–3*) enzymes. However, due to the presence of 58 nM corticosterone and 3 nM T3 in the proliferation medium (Brewer et al., 1993), residual receptor activity could explain the phenotypic effects of GR and THR antagonists on differentiating human NPCs. Moreover, NH-3 has been described as a mixed THR agonist/antagonist, both repressing co-activator binding and promoting co-repressor release (Shah et al., 2008; Webb et al., 2002), possibly modifying THR transcription in the absence of T3. For LXR, PPARs, and RXR, we hypothesize intrinsic activity in human NPCs, as supported by various receptor antagonist effects on KNDPs (Fig. 2C). While the fetal brain is efficient in cholesterol and fatty acid *de novo* synthesis, it relies on placental supply for RXR ligands (e.g., DHA, phytanic acid, and 9*c*RA). Notably, RXR is a universal heterodimerization partner, interacting with non-permissive partner receptors (RAR, THR, and VDR) and permissive partners (LXR and PPARs) (Evans and Mangelsdorf, 2014; Ribeiro et al., 1995). Heterodimers with permissive partners that exhibit intrinsic activity in human NPCs (LXR and PPARs) would have synergistic effects, indicating that the RXR also exhibits basal activity.

Species-specific effects of endocrine signaling during development question the predictability of rodent-based models for human health (Medvedev et al., 2020; Walter et al., 2019; Whitten and Patisaul, 2001). Despite the similarities in hormone receptor expression between human fetal cortex tissue, human and rat NPCs, and adult rat cortex tissue (highest expression of GR, LXRβ, PPARβδ, RARα, and THRα) (Gofflot et al., 2007), the most sensitive effects of receptor activation in human NPCs were not reproducible in rat NPCs for AhR, GR, PPARα, PPARβ/δ, PPARγ, RXR, and THR. Furthermore, human NPCs were generally more sensitive to receptor activation than rat NPCs. One explanation for this difference is that PND1 rat NPCs may represent a more advanced developmental stage than human NPCs (GW16–18). However, an empirical data-based matrix of 95 neurodevelopmental events in nine species, generated to facilitate literature comparison (Clancy et al., 2001), and a corresponding online tool (https://www.translatingtime.org/translate/) suggest that GW16–18 in humans corresponds to gestational day 21–24 in rats, the perinatal stage (Workman et al., 2013).

Since hormones have sex-specific effects on development, we analyzed effects of receptor modulators in female and male human NPCs. GR activation revealed sex-specific effects, with DEX exposure disrupting NPC proliferation and neuronal differentiation more strongly in male NPCs. Our findings align with higher fetal cortisol and corticosterone levels in female infants, potentially providing higher tolerance to GR activation (Giesbrecht et al., 2016). Rodent studies further linked perinatal BPA exposure to sex-specific alterations in GC levels, GR expression and offspring behavior, with females exhibiting anti-anxiety-like behavior and males showing depression-like behavior (Chen et al., 2014). Mood and anxiety disorders are caused by altered neurotransmission and circuit formation in the limbic, brain stem, and higher cortical brain areas (Martin et al., 2009), which the Neurosphere Assay cannot assess. This limitation is covered by neural network formation assays in the DNT IVB (Bartmann et al., 2023; Brown et al., 2016; OECD, 2023). Additionally, LXR activation impaired radial glia migration exclusively in female human NPCs, being the first mention of sexspecific LXR effects during neurodevelopment. Given that the LXR regulates radial glia migration and oligodendrogenesis during cortical layer formation in mice (Fan et al., 2008; Xu et al., 2014), excessive LXR activation may disrupt radial glia migration in a sex-specific manner. The underlying mechanisms remain to be investigated.

Dysregulated progenitor cell proliferation can cause microcephaly or megalencephaly, which are pathognomonic for various neurological disorders (Guerrini and Dobyns, 2014). We showed previously that human NPCs proliferation is EGFR-dependent and disrupted by DNT chemicals including cadmium, arsenic, and methyl mercury (Baumann et al., 2016; Koch et al., 2022; Masjosthusmann et al., 2020). Now, we correlate GR activation with impaired NPC proliferation and repression of RNA- and DNA-based transcriptional promoters, consistent with studies in human immortalized hippocampal progenitors, rodent neural stem cells, and human NPCs (Alnoud et al., 2021; Anacker et al., 2013; Moors et al., 2012; Sundberg et al., 2006). Elevated fetal GC levels due to prenatal GC treatment, placental dysfunction, or maternal stress correlate with learning and memory deficits, ADHD, lower IQ and anxiety disorders in children (LeWinn et al., 2009; Moisiadis and Matthews, 2014). The RAR activation-dependent NPC proliferation arrest was accompanied by transcriptional suppression of cell division cycle proteins (*CDC20, CDC25B, CDC25C, CDCA3, CDCA8*), regulator of cell cycle (*RGCC*), tubulins (*TUBA1B, TUBA1C, TUBA4A, TUBB, TUBB4B, TUBG1*), and the EGFR. Our results are consistent with previous studies linking EGFR suppression to impaired NPC proliferation and microcephaly (Carpentieri et al., 2022), and a*t*RA exposure to proliferation arrest and prolonged cell cycle G1 phase (Lin et al., 2017).

White matter injury due to impaired oligodendrogenesis can result in severe motor deficits and intellectual disability (Back, 2017). Oligodendrocytes were the most sensitive cell type to hormone receptor interference (GR, LXR, PPARα and βδ, PR, RAR, RXR, THR and VDR), suggesting that hormone receptor dysregulation is a major driver of oligodendrocyte dysfunction. LXR, PR and RXR activation further repressed expression of *SOX10* (all) and *CD38* (LXR), two key oligodendrocyte lineage determinants (Hattori et al., 2017; Stolt et al., 2002). Oligodendrogenesis is regulated by multiple signaling pathways, including MAPK/ERK and Wnt signaling (Gaesser and Fyffe-Maricich, 2016; van Tilborg et al., 2016). While previous rodent in vitro studies reported that MAPK/ERK signaling promotes oligodendrogenesis (Dai et al., 2014; Fyffe-Maricich et al., 2011; Guardiola-Diaz et al., 2012), we provide first evidence that this process is regulated by LXR, PPARβδ, RAR and RXR and thus susceptible to EDC exposure. In contrast, prenatal Wnt pathway overactivation impairs oligodendrocyte precursor cell (OPC) differentiation, while Wnt inhibition increase OPC numbers (Fancy et al., 2009; Guo et al., 2015; Langseth et al., 2010). We observed that, LXR (0.595-fold), PPARα (0.690-fold), PPARβδ (0.668-fold), and PG (0.541-fold) suppress *FRZB* expression, a key Wnt pathway inhibitor, illustrating extensive involvement of hormone receptors in fetal Wnt pathway regulation. Additionally, we provide evidence that hormone receptors control oligodendrogenesis by regulating fatty acid and cholesterol homeostasis. Impaired oligodendrocyte differentiation upon LXR, RXR, and PPARα activation correlated with dysregulation of cholesterol biosynthesis, efflux, and lipid metabolism genes, including *ABCA1, ABCD1, ABCG1, ACSL3, APOE, CPT1A, DHCR24, DHCR7, ECH1, FASN, HMGCS1, LPCAT3, LPL, PNPLA3, SCD*, and *SREBF1*. Given that oligodendrocytes synthesize more than 3-fold their weight in myelin per day (73 % lipids including cholesterol), maintaining lipid homeostasis is crucial for their development and function (Mathews and Appel, 2016; Norton and Poduslo, 1973). While excessive cholesterol causes oligodendrocyte lipotoxicity, cholesterol shortage causes hypomyelination and reduced oligodendrocyte numbers (Berghoff et al., 2017; Saher et al., 2012). Our observations align with studies linking PPARs to fatty acid oxidation and lipid metabolism (Grygiel-Górniak, 2014). We also previously reported that disturbed cholesterol metabolism impairs oligodendrocyte differentiation in human NPCs exposed to the flame retardant tetrabromobisphenol A (TBBPA), a known EDC (Klose et al., 2021). In our previous study, we identified THR disruption-dependent impaired oligodendrocyte maturation as a second TBBPA MoA, demonstrating the threat of EDCs disrupting multiple hormonal pathways that converge on the same KNDP, thus fortifying the hazard. Although astrocytes are present in the human NPC culture, they are not directly assessed in the Neurosphere Assay. This represents a limitation of the study, however, we hypothesize that astrocyte-related effects might indirectly influence the co-cultured neurons and oligodendrocytes, which are directly evaluated. To address this gap, we are currently developing a NAM designed to detect chemical effects on astrocyte differentiation and maturation, which will be evaluated for its added value in ED-DNT assessment in the future.

**Fig. 10.**
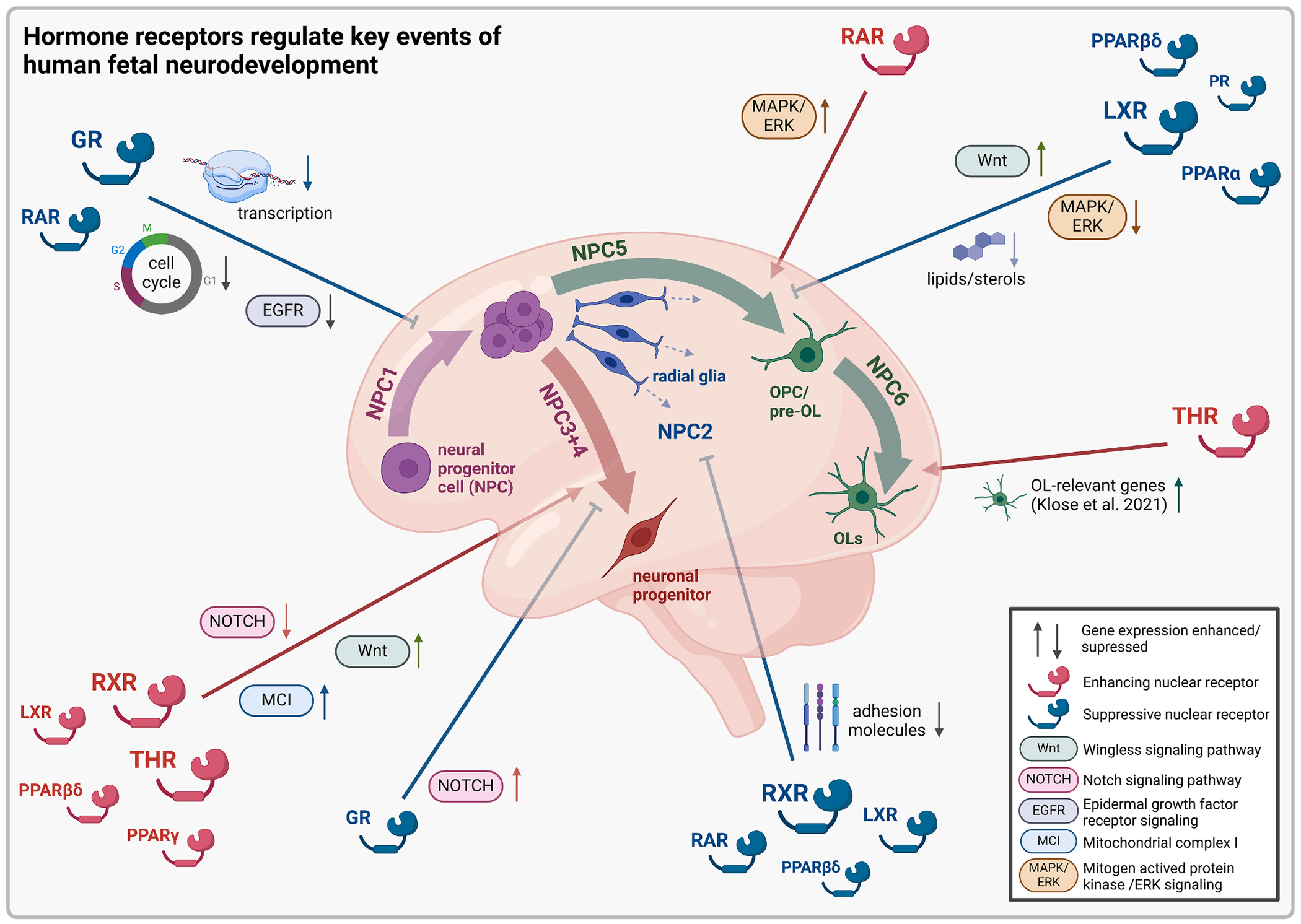
Overview of putative mechanisms underlying hormone receptor regulation of KNDPs. Receptors promoting specific KNDPs are highlighted in red, whereas those suppressing KNDPs are shown in blue. The size of the receptor icon represents sensitivity (lowest BMC) of the KNDP to activation of the respective receptor. Created with biorender.com. (For interpretation of the references to color in this figure legend, the reader is referred to the web version of this article.)

A balanced neurogenesis is a prerequisite for neural network formation and learning and memory functions (Berdugo-Vega et al., 2020), with deficits linked to intellectual disability and epileptogenesis (Guarnieri et al., 2018; Jessberger and Parent, 2015; Stagni et al., 2018). We provide evidence that THR and RXR, two reported partner receptors forming RXR–THR heterodimers, enhance neurogenesis via Notch pathway suppression, a key regulatory pathway of neurogenesis at multiple developmental stages (Pierfelice et al., 2011). The THR target and reported Notch suppressor *KLF9* (Ying et al., 2011) was strongly upregulated in T3- (11-fold) and BEXA-exposed (3-fold) human NPCs. Our new data align with our previous study linking accelerated neuronal differentiation to Notch pathway inhibition in human NPCs, studies correlating Notch with long-term memory formation (Borghese et al., 2010; Costa et al., 2005; Koch et al., 2022), studies reporting enhanced neuronal differentiation upon THR and RXR activation, and human data associating thyroid hormone deficiency with impaired neuronal differentiation and intellectual disability (Chen et al., 2012; Liu et al., 2008; López-Espíndola et al., 2014; Mounier et al., 2015; Namba et al., 2008). However, we are the first to connect RXR- and THR-dependent neurogenesis to Notch pathway suppression. Conversely, GR activation reduced neurogenesis and increased Notch pathway gene expression in human NPCs, in line with studies linking cortisol and DEX exposure to enhanced Notch signaling and reduced neurogenesis in human hippocampal progenitors and human NPCs, respectively (Anacker et al., 2013; Moors et al., 2012). THR and RXR activation further upregulated mitochondrial complex I (MCI)-related genes (*NDUFA1, NDUFAF2, NDUFAF5, NDUFB1, NDUFB2, NDUFS5*), crucial for neurogenesis and synaptogenesis (Cabello-Rivera et al., 2019), and higher cognitive function (Wirtz and Schuelke, 2011). The significant overlap between THR- and RXR-dependent DEGs, suggests the involvement of THR–RXR heterodimers. Mitochondrial dysfunction during development manifests in severe pathologies such as Leigh syndrome, which is associated with defective metabolic reprogramming and impaired neurogenesis (Inak et al., 2021). Additionally, LXR and PPARβδ increased neurogenesis likely though Wnt signaling, a pathway crucial for neurogenesis with disruptions causing intellectual disability (Da Silva et al., 2021; Zhuang et al., 2023). Their receptor ligands DHA and other PUFAs are essential for synaptic function, neuronal differentiation, and neurite outgrowth, acting as protective antioxidants in the brain (Basak and Duttaroy, 2023; Cao et al., 2009; Drolet et al., 2021; Katakura et al., 2009). Our results align with rodent studies reporting elevated neuronal numbers upon LXR activation, whereas LXR deficiency resulted in reduced neurogenesis and autistic-like behavior (Cai et al., 2018; Theofilopoulos et al., 2013). Similarly, PPARβδ activation was associated with the restoration of Wnt pathway activity in a rat model of liver regeneration after ethanol exposure (Xu et al., 2015). Fig. 10 summarizes the proposed signaling pathways and cellular processes by which hormone receptors regulate human KNDPs in vitro.

Possible species differences in hormone receptor-regulated KNDPs are just one limitation of in vivo (ED)-DNT studies, reinforcing the need for human-based NAMs in risk assessment (Paparella et al., 2020). Concerns over insufficient testing throughout, high animal use, and lack of mechanistic information led to scientific consensus by different stakeholders supporting the integration of NAMs in DNT testing (Fritsche et al., 2018). In 2018, Bal Price et al. introduced a scoring system to assess NAM readiness for application in screening and prioritization or risk assessment and evaluate their use in fit-for-purpose integrated approach to testing and assessment (IATA) (Bal-Price et al., 2018a; Crouzet et al., 2023). Since in vitro NAMs model KNDPs or molecular initiating events (MIEs) but lack information on an organism level, their development is supported by the adverse outcome pathway (AOP) concept (Klose et al., 2022a) and the integration into testing batteries covering all relevant models and endpoints (Bal-Price et al., 2010). For (ED)-DNT, we support a tiered approach, in which NAMs with broad applicability domains, like the Neurosphere Assay, serve as tier 1 screening tools, while higher-tier NAMs focus on more mechanistic ED-DNT-specific assessment. Additionally, Physiologically Based Pharmacokinetic (PBPK) modeling enables the prediction of organ-specific concentrations, pharmacokinetics, species translation, and quantitative AOP development, facilitating the integration of human-based NAMs into EDC risk assessment (Deepika and Kumar, 2023; Maass et al., 2023).

## 5. Conclusion

Although the exact mechanisms of endocrine disruption during brain development are not entirely delineated, the consequences of EDC exposure can be severe (Caporale et al., 2022; Özel and Rüegg, 2023; Tanner et al., 2020). This study addresses key data gaps by identifying hormonal dependencies and EDC sensitivities of neurodevelopmental processes, marking an important step toward developing human-relevant ED-DNT test methods to eventually improve EDC hazard assessment (Cediel-Ulloa et al., 2024, 2022; Lupu et al., 2020). We show that hormones and their receptors exhibit KNDP-specific patterns, indicating that EDCs will disturb brain development in KNDP-specific ways, depending on their molecular MoAs. This implies the need for mechanistic, KNDP-based ED-DNT test methods like the Neurosphere Assay that assess endophenotypes of toxicity rather than apical endpoints. We further question the human relevance of the rodent-based EDC risk assessment, since rat neural in vitro models fail to recapitulate the neurodevelopmental consequences of hormone receptor disruption observed in human NPCs. Our results expand the applicability domain of the DNT IVB (OECD, 2023) towards ED-DNT, showing that KNDPs modelled in the Neurosphere Assay like NPC proliferation and differentiation are highly sensitive to GR, LXR, PPAR, PR, RAR, RXR, and THR modulation. Future research will elucidate receptor crosstalk underlying our phenotypic observations and study the chemical applicability domain of the assay.

## Supplementary Material

1

2

3

4

5

6

7

8

9

10

11

## Figures and Tables

**Table 1:** List of hormone receptor agonists and antagonist used for modulation of receptor activity, including their manufacturers, catalog numbers, solvents, and stock concentrations.

Receptor	Modulation	Chemical	Abbrev.	CAS-Nr.	Manufacturer	Catalog-Nr.	Solvent	Stock [mM]
AR	Agonist	Dihydrotestosterone	DHT	521–18-6	Selleckchem	S4757	DMSO	5
	Antagonist	Hydroxyflutamide	HFU	52806–53-8	Merck (Sigma)	H4166-5MG	DMSO	10
AHR	Agonist	Benzo[*a*]pyrene	BaP	50–32-8	Merck (Sigma)	B1760-100MG	DMSO	5
	Antagonist	3′-Methoxy-4′-nitroflavone	MNF	145370–39-4	Symrise AG	customized order	DMSO	10
ER	Agonist	17β-Estradiol	E2	50–28-2	Merck (Sigma)	E2758-250MG	2 % EtOH in DMEM	5
	Antagonist	Fulvestrant	FU	129453–61-8	Biomol (Cayman)	Cay10011269-1	DMSO	5
GR	Agonist	Dexamethasone	DEX	50–02-2	Merck (Sigma)	D4902-25MG	DMSO	5
	Antagonist	AL 082D06	AL08	256925–03-8	Biomol (Cayman)	Cay23455-1	DMSO	10
	Antagonist	Mifepristone	MP	84371–65-3	Merck (Sigma)	M8046-100MG	DMSO	10
LXR	Agonist	GW3965 hydrochloride	GW39	405911–17-3	Biomol (Cayman)	Cay10054-1	DMSO	5
	Antagonist	SR9238	SR92	1416153–62-2	Biomol (Cayman)	Cay18771-1	DMSO	5
PPARα	Agonist	GW7647	GW7647	265129–71-3	Biomol (Cayman)	Cay10008613-1	DMSO	5
	Antagonist	GW6471	GW6471	880635–03-0	Biomol (Cayman)	Cay11697-1	DMSO	10
PPARβ	Agonist	GW0742	GW0742	317318–84-6	Biomol (Cayman)	Cay10006798-5	DMSO	5
	Antagonist	GSK3787	GSK	188591–46-0	Biomol (Cayman)	Cay15219-5	DMSO	10
PPARγ	Agonist	Rosiglitazone	Rosi	122320–73-4	Biomol (Cayman)	Cay71740-10	DMSO	5
	Antagonist	GW9662	GW9662	22978–25-2	Biomol (Cayman)	Cay70785-1	DMSO	10
PR	Agonist	Progesterone	PG	57–83-0	Merck (Sigma)	P6149-1MG	DMSO	5
	Antagonist	Ulipristal Acetate	UA	126784–99-4	Biomol (Cayman)	Cay23657-5	DMSO	10
PGE2R	Agonist	Prostaglandin E2	PGE2	363–24-6	Merck (Sigma)	P5640-1MG	H_2_O	20
RAR	Agonist	all-*trans* retinoic acid	a*t*RA	302–79-4	Merck (Sigma)	R2625-50MG	DMSO	5
	Antagonist	AGN193109	AGN	171746–21-7	Merck (Sigma)	SML2034-5MG	DMSO	10
RXR	Agonist	Bexarotene	BEXA	153559–49-0	Merck (Sigma)	200499-50MG	DMSO	10
	Antagonist	HX531	HX	188844–34-0	Biomol (Cayman)	Cay20762-1	DMSO	10
THR	Agonist	Triiodothyronine	T3	6893–02-3	Merck (Sigma)	T2877	96 %EtOH/1M HCl (1:1)	0.081
	Antagonist	NH-375	NH-3	Nguyen 2002; Singh 2016 (provided by UC Davis)			DMSO	0.729
VDR	Agonist	Calcitriol	Calci	32222–06-3	Biomol (Cayman)	Cay71820-5	DMSO	5

**Table 2:** Comparison of fetal cord blood concentrations of physiological hormone receptor ligands (current literature) and BMCs of KNDP-related hormone receptor agonist effects (present study).

Receptor	natural receptor ligand	fetal cord blood concentration [ng/ml or μg/ml]	fetal cord blood concentration [nM]	agonist(present study)	BMC [nM] (present study)	BMC biologically relevant?	Reference
**Aryl hydrocyrbon receptor (AhR)**	benzo(a)pyrenechlorodibenzo-p-dioxin (TCDD)			benzo(a)pyrene	*1020.0*	*questionable*	
**Glucocorticoid receptor (GR)**	cortisol	65–92 ng/ml	179.0–254.0	dexamethasone	**6.1**	**yes**	Pearson Murphy (1983)
**Liver X receptor (LXR)**	oxysterols and cholestenoic acid	24S-hydroxycholesterol: 35–62 ng/ml; 27-hydroxycholesterol: 21–39 ng/ml	86.9–153.9; 52.2–96.9	GW3965	**25.1**	**yes**	Lütjohann et al. (2001)
**Peroxisome proliferator-activated receptors (PPARs)**	poly unsaturated fatty acids (PUFAs) – eicosatetraenoic acids (i.e. arachidonic acid and metabolites) – octadecadienoic acids (i.e. linoleic acid and metabolites) docosahexaenoic acid (DHA)	Total n-6 PUFAs: 199.9–536.1 μg/ml; arachidonic acid: 86.6–145.4 μg/ml; linoleic acid: 84–154 μg/ml; DHA: 41.7–75.9 μg/ml	n.a.; 284,400.0–477,551.2; 299,521.6–549,123.0; 126,944.5–231,057.3	GW7647 (PPARα) GW0742 (PPARβδ) rosiglitazone (PPARγ)	**371.7** **435.8** **127.5**	**yes** **yes** **yes**	Grygiel-Górniak (2014); Sakamoto et al.(2018)
**Progesterone receptor (PR)**	progesterone	176.4–2818.0 ng/ml	560.9–8961.1	progesterone	**1224.8**	**yes**	Lagiou et al. (2011)
**Prostaglandin E2 receptor (PGE2R)**	prostaglandin E2	9.5 ng/ml	27	prostaglandin E2	*13693.0*	*no*	Schlagenhauf et al. (2015)
**Retinoic acid receptor (RAR)**	all-*trans* retinoic acid	30–150.2 ng/ml	100–550	all-*trans* retinoic acid	**31.9**	**yes**	Manolescu et al. (2010)
**Retinoid X receptor (RXR)**	docosahexaenoic acid (DHA); 9-*cis* retinoic acid (9cRA)	DHA: 41.7–75.9 μg/ml; n.a.	126,944.5–231,057.3; n.a.	bexarotene	**3.4**	**yes**	Sakamoto et al. (2018)
**Thyroid hormone receptor (THR)**	triiodothyronine (T3)	0.543–0.606 ng/ml	0.77 – 0.92	triiodothyronine (T3)	**0.3**	**yes**	Wang et al. (2005)
**Vitamin D receptor (VDR)**	calcitriol (1,25-dihydroxyvitamin D, active form); calcidiol (25-hydroxyvitamin D, 25 [OH]D, stable metabolite)	25[OH]D: 16.6 ng/ml (mean over all studies included in meta-analysis)	41.55	calcitriol	*785.6*	*no*	Wong et al. (2022)

## Data Availability

Data will be made available on request.

## Abbreviations:

AhR: aryl hydrocarbon receptor
AI: artificial intelligence
AOP: adverse outcome pathway
AR: androgen receptor
BEXA: bexarotene
BMC: benchmark concentration
BMCL: benchmark concentration lower confidence limit
BMCU: benchmark concentration upper confidence limit
BMR: benchmark response
BrdU: bromodeoxyuridine
CNN: convolutional neural network
CV: coefficient of variation
DEG: differentially expressed gene
DEX: dexamethasone
DHT: dihydrotestosterone
DMSO: dimethyl sulfoxide
DNT: developmental neurotoxicity
DHA: docosahexaenoic acid
EATS: estrogens, androgens, thyroid hormones, and steroidogenesis
ED: endocrine disruption
EDC: endocrine disrupting chemical
ED-DNT: endocrine disruption-mediated developmental neurotoxicity
EFSA: European Food Safety Authority
EGF: epidermal growth factor
EGFR: epidermal growth factor receptor
EM: emission
EOGRTS: Extended One-Generation Reproductive Toxicity Study
ER: estrogen receptor
EtOH: ethanol
FGF2: fibroblast growth factor 2
FPKM: fragments per kilobase of transcript per million mapped reads
FRZB: frizzled-related protein B
GC: glucocorticoid
GO: gene ontology
GR: glucocorticoid receptor
GW: gestational week
HCA: high-content analysis
hormone receptor: hormone receptor
IATA: integrated approach to testing and assessment
IVB: in vitro battery
KLF9: Krüppel-like factor 9
KNDP: key neurodevelopmental process
LXR: liver X receptor
MAPK/ERK: mitogen-activated protein kinase/extracellular signal-regulated kinase
MCI: mitochondrial complex I
MNF: 3′-methoxy-4′-nitroflavone
MoA: mode of action
MSE: most sensitive endpoint
NAM: New Approach Methodology
NIEHS: National Institute of Environmental Health Sciences
NPC: neural progenitor cell
NR1H2: nuclear receptor subfamily 1, group H, member 2 (LXRβ)
NR3C1: nuclear receptor subfamily 3, group C, member 1 (GR)
OECD: Organisation for Economic Co-operation and Development
OL: oligodendrocytes
OPC: oligodendrocyte precursor cell
PBPK: physiologically based pharmacokinetic modeling
PCB: polychlorinated biphenyl
PDL: poly-D-lysine
PFAS: per- and polyfluoroalkyl substances
PGE2R: prostaglandin E2 receptor
PND: postnatal day
PPAR: peroxisome proliferator-activated receptor
PR: progesterone receptor
PUFA: polyunsaturated fatty acid
RAR: retinoic acid receptor
RIN: RNA integrity number
RXR: retinoid X receptor
SOX10: SRY-box transcription factor 10
Sup. Fig.: supplementary figure
T3: triiodothyronine
TG: test guideline
THR: thyroid hormone receptor
US EPA: United States Environmental Protection Agency
VDR: vitamin D receptor
Wnt: wingless-related integration site
w/o: without

## References

[R1] Aleshin S, Reiser G, 2013. Role of the peroxisome proliferator-activated receptors (PPAR)-α, β/δ and γ triad in regulation of reactive oxygen species signaling in brain. Biol. Chem 394, 1553–1570. 10.1515/hsz-2013-0215. 24021597

[R2] Alnoud MAH, Chen W, Liu N, Zhu W, Qiao J, Chang S, Wu Y, Wang S, Yang Y, Sun Q, Kang J, 2021. Sirt7-p21 Signaling Pathway Mediates Glucocorticoid-Induced Inhibition of Mouse Neural Stem Cell Proliferation. Neurotox. Res 39, 444–455. 10.1007/s12640-020-00294-x. 33025360

[R3] Anacker C, Cattaneo A, Luoni A, Musaelyan K, Zunszain PA, Milanesi E, Rybka J, Berry A, Cirulli F, Thuret S, Price J, Riva MA, Gennarelli M, Pariante CM, 2013. Glucocorticoid-Related Molecular Signaling Pathways Regulating Hippocampal Neurogenesis. Neuropsychopharmacology 38, 872–883. 10.1038/npp.2012.253. 23303060 PMC3672002

[R4] Annunziata P, Federico A, D’Amore I, Corona RM, Guazzi GC, 1983. Impairment of human brain development: glycoconjugate and lipid changes in congenital athyroidism. Early Hum. Dev 8, 269–278. 10.1016/0378-3782(83)90009-9. 6227470

[R5] Araki A, Mitsui T, Goudarzi H, Nakajima T, Miyashita C, Itoh S, Sasaki S, Cho K, Moriya K, Shinohara N, Nonomura K, Kishi R, 2017. Prenatal di(2-ethylhexyl) phthalate exposure and disruption of adrenal androgens and glucocorticoids levels in cord blood: The Hokkaido Study. Sci. Total Environ 581–582, 297–304. 10.1016/J.SCITOTENV.2016.12.124. 28043700

[R6] Baas D, Bourbeau D, Sarlieve LL, Ittel M-E, Dussault JH, Puymirat J, 1997. Oligodendrocyte maturation and progenitor cell proliferation are independently regulated by thyroid hormone. Glia 19, 324–332. 10.1002/(SICI)1098-1136(199704)19:4<324::AID-GLIA5>3.0.CO;2-X. 9097076

[R7] Back SA, 2017. White matter injury in the preterm infant: pathology and mechanisms. Acta Neuropathol. 134, 331–349. 10.1007/s00401-017-1718-6. 28534077 PMC5973818

[R8] Bal-Price A, Hogberg HT, Crofton KM, Daneshian M, FitzGerald RE, Fritsche E, Heinonen T, Hougaard Bennekou S, Klima S, Piersma AH, Sachana M, Shafer TJ, Terron A, Monnet-Tschudi F, Viviani B, Waldmann T, Westerink RHS, Wilks MF, Witters H, Zurich MG, Leist M, 2018a. Recommendation on test readiness criteria for new approach methods in toxicology: Exemplified for developmental neurotoxicity. ALTEX 35, 306–352. 10.14573/altex.1712081. 29485663 PMC6545888

[R9] Bal-Price A, Pistollato F, Sachana M, Bopp SK, Munn S, Worth A, 2018b. Strategies to improve the regulatory assessment of developmental neurotoxicity (DNT) using in vitro methods. Toxicol. Appl. Pharmacol 354, 7–18. 10.1016/j.taap.2018.02.008. 29476865 PMC6095942

[R10] Bal-Price AK, Hogberg HT, Buzanska L, Lenas P, van Vliet E, Hartung T, 2010. In vitro developmental neurotoxicity (DNT) testing: relevant models and endpoints. Neurotoxicology 31, 545–554. 10.1016/j.neuro.2009.11.006. 19969020

[R11] Bartmann K, Bendt F, Dönmez A, Haag D, Keßel HE, Masjosthusmann S, Noel C, Wu J, Zhou P, Fritsche E, 2023. A human iPSC-based in vitro neural network formation assay to investigate neurodevelopmental toxicity of pesticides. ALTEX 40, 452–470. 10.14573/altex.2206031. 37158368

[R12] Basak S, Duttaroy AK, 2023. Maternal PUFAs, Placental Epigenetics, and Their Relevance to Fetal Growth and Brain Development. Reprod. Sci 30, 408–427. 10.1007/s43032-022-00989-w. 35676498

[R13] Baumann J, Barenys M, Gassmann K, Fritsche E, 2014. Comparative human and rat “neurosphere assay” for developmental neurotoxicity testing. Curr. Protoc. Toxicol 59, 12.21.1–24. 10.1002/0471140856.tx1221s59. 24898107

[R14] Baumann J, Gassmann K, Masjosthusmann S, DeBoer D, Bendt F, Giersiefer S, Fritsche E, 2016. Comparative human and rat neurospheres reveal species differences in chemical effects on neurodevelopmental key events. Arch. Toxicol 90, 1415–1427. 10.1007/s00204-015-1568-8. 26216354

[R15] Bellinger DC, 2012. Comparing the population neurodevelopmental burdens associated with children’s exposures to environmental chemicals and other risk factors. Neurotoxicology 33, 641–643. 10.1016/J.NEURO.2012.04.003. 22525934

[R16] Berdugo-Vega G, Arias-Gil G, López-Fernández A, Artegiani B, Wasielewska JM, Lee C-C, Lippert MT, Kempermann G, Takagaki K, Calegari F, 2020. Increasing neurogenesis refines hippocampal activity rejuvenating navigational learning strategies and contextual memory throughout life. Nat. Commun 11, 135. 10.1038/s41467-019-14026-z. 31919362 PMC6952376

[R17] Berghoff SA, Gerndt N, Winchenbach J, Stumpf SK, Hosang L, Odoardi F, Ruhwedel T, Böhler C, Barrette B, Stassart R, Liebetanz D, Dibaj P, Möbius W, Edgar JM, Saher G, 2017. Dietary cholesterol promotes repair of demyelinated lesions in the adult brain. Nat. Commun 8, 14241. 10.1038/ncomms14241. 28117328 PMC5286209

[R18] Blum J, Masjosthusmann S, Bartmann K, Bendt F, Dolde X, Dönmez A, Förster N, Holzer A-K, Hübenthal U, Keßel HE, Kilic S, Klose J, Pahl M, Stürzl L-C, Mangas I, Terron A, Crofton KM, Scholze M, Mosig A, Leist M, Fritsche E, 2023. Establishment of a human cell-based in vitro battery to assess developmental neurotoxicity hazard of chemicals. Chemosphere 311, 137035. 10.1016/j.chemosphere.2022.137035. 36328314

[R19] Borghese L, Dolezalova D, Opitz T, Haupt S, Leinhaas A, Steinfarz B, Koch P, Edenhofer F, Hampl A, Brüstle O, 2010. Inhibition of notch signaling in human embryonic stem cell-derived neural stem cells delays G1/S phase transition and accelerates neuronal differentiation in vitro and in vivo. Stem Cells 28, 955–964. 10.1002/stem.408. 20235098

[R20] Borrell V, 2019. Recent advances in understanding neocortical development. F1000 Res. 8, 1791. 10.12688/f1000research.20332.1. PMC6816450 31681469

[R21] Borrell V, Götz M, 2014. Role of radial glial cells in cerebral cortex folding. Curr. Opin. Neurobiol 27, 39–46. 10.1016/j.conb.2014.02.007. 24632307

[R22] Brewer GJ, Torricelli JR, Evege EK, Price PJ, 1993. Optimized survival of hippocampal neurons in B27-supplemented Neurobasal, a new serum-free medium combination. J. Neurosci. Res 35, 567–576. 10.1002/JNR.490350513. 8377226

[R23] Brown JP, Hall D, Frank CL, Wallace K, Mundy WR, Shafer TJ, 2016. Editor’s Highlight: Evaluation of a Microelectrode Array-Based Assay for Neural Network Ontogeny Using Training Set Chemicals. Toxicol. Sci 154, 126–139. 10.1093/toxsci/kfw147. 27492221

[R24] Buss C, Davis EP, Shahbaba B, Pruessner JC, Head K, Sandman CA, 2012. Maternal cortisol over the course of pregnancy and subsequent child amygdala and hippocampus volumes and affective problems. Proc. Natl. Acad. Sci. u. s. a 109. 10.1073/PNAS.1201295109. PMC3356611 22529357

[R25] Cabello-Rivera D, Sarmiento-Soto H, López-Barneo J, Muñoz-Cabello AM, 2019. Mitochondrial Complex I Function Is Essential for Neural Stem/Progenitor Cells Proliferation and Differentiation. Front. Neurosci 13, 664. 10.3389/fnins.2019.00664. 31297047 PMC6607990

[R26] Cai Y, Tang X, Chen X, Li X, Wang Y, Bao X, Wang L, Sun D, Zhao J, Xing Y, Warner M, Xu H, Gustafsson J-Å, Fan X, 2018. Liver X receptor β regulates the development of the dentate gyrus and autistic-like behavior in the mouse. Proc. Natl. Acad. Sci 115, E2725–E2733. 10.1073/pnas.1800184115. 29507213 PMC5866608

[R27] Cao D, Kevala K, Kim J, Moon H, Jun SB, Lovinger D, Kim H, 2009. Docosahexaenoic acid promotes hippocampal neuronal development and synaptic function. J. Neurochem 111, 510–521. 10.1111/j.1471-4159.2009.06335.x. 19682204 PMC2773444

[R28] Caporale N, Leemans M, Birgersson L, Germain PL, Cheroni C, Borbély G, Engdahl E, Lindh C, Bressan RB, Cavallo F, Chorev NE, D’Agostino GA, Pollard SM, Rigoli MT, Tenderini E, Tobon AL, Trattaro S, Troglio F, Zanella M, Bergman Å, Damdimopoulou P, Jönsson M, Kiess W, Kitraki E, Kiviranta H, Nånberg E, Öberg M, Rantakokko P, Rudén C, Söder O, Bornehag CG, Demeneix B, Fini JB, Gennings C, Rüegg J, Sturve J, Testa G, 2022. From cohorts to molecules: Adverse impacts of endocrine disrupting mixtures. Science 375. 10.1126/SCIENCE.ABE8244. 35175820

[R29] Carpentieri JA, Di Cicco A, Lampic M, Andreau D, Del Maestro L, El Marjou F, Coquand L, Bahi-Buisson N, Brault J-B, Baffet AD, 2022. Endosomal trafficking defects alter neural progenitor proliferation and cause microcephaly. Nat. Commun 13, 16. 10.1038/s41467-021-27705-7. 35013230 PMC8748540

[R30] Cediel-Ulloa A, Awoga R, Dönmez A, Yu X, Gliga A, Attoff K, Forsby A, Rüegg J, 2024. Characterization of the C17.2 cell line as testing system for endocrine disruption-induced developmental neurotoxicity. ALTEX 10.14573/altex.2404131. 39246236

[R31] Cediel-Ulloa A, Lupu DL, Johansson Y, Hinojosa M, Özel F, Rüegg J, 2022. Impact of endocrine disrupting chemicals on neurodevelopment: the need for better testing strategies for endocrine disruption-induced developmental neurotoxicity. Expert Rev. Endocrinol. Metab 17, 131–141. 10.1080/17446651.2022.2044788. 35255767

[R32] Chen C, Zhou Z, Zhong M, Zhang Y, Li M, Zhang L, Qu M, Yang J, Wang Y, Yu Z, 2012. Thyroid Hormone Promotes Neuronal Differentiation of Embryonic Neural Stem Cells by Inhibiting STAT3 Signaling Through TRα1. Stem Cells Dev. 21, 2667–2681. 10.1089/scd.2012.0023. 22468949 PMC3438880

[R33] Chen F, Zhou L, Bai Y, Zhou R, Chen L, 2014. Sex differences in the adult HPA axis and affective behaviors are altered by perinatal exposure to a low dose of bisphenol A. Brain Res. 1571, 12–24. 10.1016/j.brainres.2014.05.010. 24857958

[R34] Chollet F, 2017. Keras (2015).

[R35] Clancy B, Darlington RB, Finlay BL, 2001. Translating developmental time across mammalian species. Neuroscience 105, 7–17. 10.1016/S0306-4522(01)00171-3. 11483296

[R36] Cooke B, Hegstrom CD, Villeneuve LS, Breedlove SM, 1998. Sexual Differentiation of the Vertebrate Brain: Principles and Mechanisms. Front. Neuroendocrinol 19, 323–362. 10.1006/frne.1998.0171. 9799588

[R37] Costa RM, Drew C, Silva AJ, 2005. Notch to remember. Trends Neurosci. 28, 429–435. 10.1016/j.tins.2005.05.003. 15922461

[R38] Courtney R, Landreth GE, 2016. LXR Regulation of Brain Cholesterol: From Development to Disease. Trends Endocrinol. Metab 27, 404–414. 10.1016/j.tem.2016.03.018. 27113081 PMC4986614

[R39] Crouzet T, Grignard E, Brion F, Blanc EB, Podechard N, Langouet S, Alonso-Magdalena P, Hubert P, Kim MJ, Audouze K, 2023. ReadEDTest: A tool to assess the readiness of in vitro test methods under development for identifying endocrine disruptors. Environ. Int 174. 10.1016/J.ENVINT.2023.107910. 37028267

[R40] Cunningham TJ, Duester G, 2015. Mechanisms of retinoic acid signalling and its roles in organ and limb development. Nat. Rev. Mol. Cell Biol 16, 110–123. 10.1038/NRM3932. 25560970 PMC4636111

[R41] Da Silva F, Zhang K, Pinson A, Fatti E, Wilsch-Bräuninger M, Herbst J, Vidal V, Schedl A, Huttner WB, Niehrs C, 2021. Mitotic WNT signalling orchestrates neurogenesis in the developing neocortex. EMBO J. 40, e108041. 10.15252/embj.2021108041. 34431536 PMC8488556

[R42] Dach K, Bendt F, Huebenthal U, Giersiefer S, Lein PJ, Heuer H, Fritsche E, 2017. BDE-99 impairs differentiation of human and mouse NPCs into the oligodendroglial lineage by species-specific modes of action. Sci. Rep 7, 44861. 10.1038/srep44861. 28317842 PMC5357893

[R43] Dai J, Bercury KK, Macklin WB, 2014. Interaction of mTOR and Erk1/2 signaling to regulate oligodendrocyte differentiation. Glia 62, 2096–2109. 10.1002/glia.22729. 25060812 PMC4406223

[R44] De Urquiza AM, Liu S, Sjoberg M, Zetterstrom RH, Griffiths W, Sjovall J, Perlmann T, 2000. Docosahexaenoic acid, a ligand for the retinoid X receptor in mouse brain. Science 290, 2140–2144. 10.1126/SCIENCE.290.5499.2140. 11118147

[R45] Deepika D, Kumar V, 2023. The Role of “Physiologically Based Pharmacokinetic Model (PBPK)” New Approach Methodology (NAM) in Pharmaceuticals and Environmental Chemical Risk Assessment. Int. J. Environ. Res. Public Health 20, 3473. 10.3390/ijerph20043473. 36834167 PMC9966583

[R46] Derakhshan A, Shu H, Broeren MAC, Lindh CH, Peeters RP, Kortenkamp A, Demeneix B, Bornehag CG, Korevaar TIM, 2021. Association of phthalate exposure with thyroid function during pregnancy. Environ. Int 157, 106795. 10.1016/J.ENVINT.2021.106795. 34358912

[R47] Dickerson SM, Cunningham SL, Gore AC, 2011. Prenatal PCBs disrupt early neuroendocrine development of the rat hypothalamus. Toxicol. Appl. Pharmacol 252, 36–46. 10.1016/j.taap.2011.01.012. 21277884 PMC3060304

[R48] Drolet J, Buchner-Duby B, Stykel MG, Coackley C, Kang JX, Ma DWL, Ryan SD, 2021. Docosahexanoic acid signals through the Nrf2–Nqo1 pathway to maintain redox balance and promote neurite outgrowth. Mol. Biol. Cell 32, 511–520. 10.1091/mbc.E20-09-0599. 33502893 PMC8101469

[R49] Dugas JC, Ibrahim A, Barres BA, 2012. The T3-induced gene KLF9 regulates oligodendrocyte differentiation and myelin regeneration. Mol. Cell. Neurosci 50, 45–57. 10.1016/j.mcn.2012.03.007. 22472204 PMC4441621

[R50] Dunnett CW, Tamhane AC, 1991. Step-down multiple tests for comparing treatments with a control in unbalanced one-way layouts. Stat. Med 10, 939–947. 10.1002/SIM.4780100614. 1876783

[R51] Engel SM, Zhu C, Berkowitz GS, Calafat AM, Silva MJ, Miodovnik A, Wolff MS, 2009. Prenatal phthalate exposure and performance on the Neonatal Behavioral Assessment Scale in a multiethnic birth cohort. Neurotoxicology 30, 522–528. 10.1016/J.NEURO.2009.04.001. 19375452 PMC4026936

[R52] Evans RM, Mangelsdorf DJ, 2014. Nuclear Receptors, RXR, and the Big Bang. Cell 157, 255–266. 10.1016/j.cell.2014.03.012. 24679540 PMC4029515

[R53] Fan X, Kim H-J, Bouton D, Warner M, Gustafsson J-Å, 2008. Expression of liver X receptor β is essential for formation of superficial cortical layers and migration of later-born neurons. Proc. Natl. Acad. Sci 105, 13445–13450. 10.1073/pnas.0806974105. 18768805 PMC2533209

[R54] Fancy SPJ, Baranzini SE, Zhao C, Yuk D-I, Irvine K-A, Kaing S, Sanai N, Franklin RJM, Rowitch DH, 2009. Dysregulation of the Wnt pathway inhibits timely myelination and remyelination in the mammalian CNS. Genes Dev. 23, 1571–1585. 10.1101/gad.1806309. 19515974 PMC2704469

[R55] Florio M, Huttner WB, 2014. Neural progenitors, neurogenesis and the evolution of the neocortex. Development 141, 2182–2194. 10.1242/dev.090571. 24866113

[R56] Förster N, Butke J, Keßel HE, Bendt F, Pahl M, Li L, Fan X, Leung P, Klose J, Masjosthusmann S, Fritsche E, Mosig A, 2022. Reliable identification and quantification of neural cells in microscopic images of neurospheres. Cytom. Part A 101, 411–422. 10.1002/cyto.a.24514. 34747115

[R57] Fritsche E, Grandjean P, Crofton KM, Aschner M, Goldberg A, Heinonen T, Hessel EVS, Hogberg HT, Bennekou SH, Lein PJ, Leist M, Mundy WR, Paparella M, Piersma AH, Sachana M, Schmuck G, Solecki R, Terron A, Monnet-Tschudi F, Wilks MF, Witters H, Zurich M-G, Bal-Price A, 2018. Consensus statement on the need for innovation, transition and implementation of developmental neurotoxicity (DNT) testing for regulatory purposes. Toxicol. Appl. Pharmacol 354, 3–6. 10.1016/j.taap.2018.02.004. 29447839 PMC6097873

[R58] Fyffe-Maricich SL, Karlo JC, Landreth GE, Miller RH, 2011. The ERK2 Mitogen-Activated Protein Kinase Regulates the Timing of Oligodendrocyte Differentiation. J. Neurosci 31, 843–850. 10.1523/JNEUROSCI.3239-10.2011. 21248107 PMC3568938

[R59] Gaesser JM, Fyffe-Maricich SL, 2016. Intracellular signaling pathway regulation of myelination and remyelination in the CNS. Exp. Neurol 283, 501–511. 10.1016/j.expneurol.2016.03.008. 26957369 PMC5010983

[R60] Gamoh S, Hashimoto M, Sugioka K, Shahdat Hossain M, Hata N, Misawa Y, Masumura S, 1999. Chronic administration of docosahexaenoic acid improves reference memory-related learning ability in young rats. Neuroscience 93, 237–241. 10.1016/S0306-4522(99)00107-4. 10430487

[R61] Genz A, Bretz F, Miwa T, Mi X, Leisch F, Scheipl F, Hothorn T, 2020. mvtnorm: Multivariate Normal and t Distributions.

[R62] Giera S, Bansal R, Ortiz-Toro TM, Taub DG, Zoeller RT, 2011. Individual polychlorinated biphenyl (PCB) congeners produce tissue- and gene-specific effects on thyroid hormone signaling during development. Endocrinology 152, 2909–2919. 10.1210/EN.2010-1490. 21540284 PMC3115602

[R63] Giesbrecht GF, Rash JA, Edwards HE, Wynne-Edwards KE, 2016. Full-term deliveries without antecedent labor reveal sex differences in umbilical cord glucocorticoid concentrations. Psychoneuroendocrinology 74, 121–125. 10.1016/j.psyneuen.2016.08.030. 27608361

[R64] Gika AD, Siddiqui A, Hulse AJ, Edward S, Fallon P, McEntagart ME, Jan W, Josifova D, Lerman-Sagie T, Drummond J, Thompson E, Refetoff S, Bönnemann CG, Jungbluth H, 2010. White matter abnormalities and dystonic motor disorder associated with mutations in the SLC16A2 gene. Dev. Med. Child Neurol 52, 475–482. 10.1111/j.1469-8749.2009.03471.x. 19811520 PMC5800746

[R65] Gofflot F, Chartoire N, Vasseur L, Heikkinen S, Dembele D, Le Merrer J, Auwerx J, 2007. Systematic Gene Expression Mapping Clusters Nuclear Receptors According to Their Function in the Brain. Cell 131, 405–418. 10.1016/J.CELL.2007.09.012. 17956739

[R66] Gore AC, Martien KM, Gagnidze K, Pfaff D, 2014. Implications of Prenatal Steroid Perturbations for Neurodevelopment, Behavior, and Autism. Endocr. Rev 35, 961–991. 10.1210/er.2013-1122. 25211453 PMC4234775

[R67] Grignard E, Håkansson H, Munn S, 2020. Regulatory needs and activities to address the retinoid system in the context of endocrine disruption: The European viewpoint. Reprod. Toxicol 93, 250–258. 10.1016/J.REPROTOX.2020.03.002. 32171711 PMC7322530

[R68] Grygiel-Górniak B, 2014. Peroxisome proliferator-activated receptors and their ligands: nutritional and clinical implications - a review. Nutr. J 13, 17. 10.1186/1475-2891-13-17. 24524207 PMC3943808

[R69] Guardiola-Diaz HM, Ishii A, Bansal R, 2012. Erk1/2 MAPK and mTOR signaling sequentially regulates progression through distinct stages of oligodendrocyte differentiation. Glia 60, 476–486. 10.1002/glia.22281. 22144101 PMC3265651

[R70] Guarnieri FC, de Chevigny A, Falace A, Cardoso C, 2018. Disorders of neurogenesis and cortical development. Dialogues Clin. Neurosci 20, 255–266. 10.31887/DCNS.2018.20.4/ccardoso. 30936766 PMC6436956

[R71] Guerrini R, Dobyns WB, 2014. Malformations of cortical development: clinical features and genetic causes. Lancet Neurol. 13, 710–726. 10.1016/S1474-4422(14)70040-7. 24932993 PMC5548104

[R72] Guo F, Lang J, Sohn J, Hammond E, Chang M, Pleasure D, 2015. Canonical Wnt signaling in the oligodendroglial lineage-puzzles remain. Glia 63, 1671–1693. 10.1002/glia.22813. 25782433

[R73] Guo YL, Lambert GH, Hsu CC, Hsu MML, 2004. Yucheng: health effects of prenatal exposure to polychlorinated biphenyls and dibenzofurans. Int. Arch. Occup. Environ. Health 77, 153–158. 10.1007/S00420-003-0487-9. 14963712

[R74] Gupta RK, Bhatia V, Poptani H, Gujral RB, 1995. Brain metabolite changes on in vivo proton magnetic resonance spectroscopy in children with congenital hypothyroidism. J. Pediatr 126, 389–392. 10.1016/S0022-3476(95)70454-X. 7869198

[R75] Hattori T, Kaji M, Ishii H, Jureepon R, Takarada-Iemata M, Minh Ta H, Manh Le T, Konno A, Hirai H, Shiraishi Y, Ozaki N, Yamamoto Y, Okamoto H, Yokoyama S, Higashida H, Kitao Y, Hori O, 2017. CD38 positively regulates postnatal development of astrocytes cell-autonomously and oligodendrocytes non-cell-autonomously. Glia 65, 974–989. 10.1002/glia.23139. 28295574

[R76] He Y, Yi W, Suino-Powell K, Zhou XE, Tolbert WD, Tang X, Yang J, Yang H, Shi J, Hou L, Jiang H, Melcher K, Xu HE, 2014. Structures and mechanism for the design of highly potent glucocorticoids. Cell Res. 24, 713–726. 10.1038/CR.2014.52. 24763108 PMC4042175

[R77] Hunter J, 1996. Crosstalk between the thyroid hormone and peroxisome proliferator-activated receptors in regulating peroxisome proliferator-responsive genes. Mol. Cell. Endocrinol 116, 213–221. 10.1016/0303-7207(95)03717-9. 8647322

[R78] Inak G, Rybak-Wolf A, Lisowski P, Pentimalli TM, Jüttner R, Glažar P, Uppal K, Bottani E, Brunetti D, Secker C, Zink A, Meierhofer D, Henke M-T, Dey M, Ciptasari U, Mlody B, Hahn T, Berruezo-Llacuna M, Karaiskos N, Di Virgilio M, Mayr JA, Wortmann SB, Priller J, Gotthardt M, Jones DP, Mayatepek E, Stenzel W, Diecke S, Kühn R, Wanker EE, Rajewsky N, Schuelke M, Prigione A, 2021. Defective metabolic programming impairs early neuronal morphogenesis in neural cultures and an organoid model of Leigh syndrome. Nat. Commun 12, 1929. 10.1038/s41467-021-22117-z. 33771987 PMC7997884

[R79] Janesick A, Wu SC, Blumberg B, 2015. Retinoic acid signaling and neuronal differentiation. Cell. Mol. Life Sci 72, 1559–1576. 10.1007/S00018-014-1815-9. 25558812 PMC11113123

[R80] Jansen TA, Korevaar TIM, Mulder TA, White T, Muetzel RL, Peeters RP, Tiemeier H, 2019. Maternal thyroid function during pregnancy and child brain morphology: a time window-specific analysis of a prospective cohort. lancet. Diabetes Endocrinol. 7, 629–637. 10.1016/S2213-8587(19)30153-6 31262704

[R81] Jessberger S, Parent JM, 2015. Epilepsy and Adult Neurogenesis. Cold Spring Harb. Perspect. Biol 7, a020677. 10.1101/cshperspect.a020677. 26552418 PMC4665072

[R82] Katakura M, Hashimoto M, Shahdat HM, Gamoh S, Okui T, Matsuzaki K, Shido O, 2009. Docosahexaenoic acid promotes neuronal differentiation by regulating basic helix–loop–helix transcription factors and cell cycle in neural stem cells. Neuroscience 160, 651–660. 10.1016/j.neuroscience:2009.02.057. 19272428

[R83] Klose J, Li L, Pahl M, Bendt F, Hübenthal U, Jüngst C, Petzsch P, Schauss A, Köhrer K, Leung PC, Wang CC, Koch K, Tigges J, Fan X, Fritsche E, 2022a. Application of the adverse outcome pathway concept for investigating developmental neurotoxicity potential of Chinese herbal medicines by using human neural progenitor cells in vitro. Cell Biol. Toxicol 39, 319–343. 10.1007/s10565-022-09730-4. 35701726 PMC10042984

[R84] Klose J, Pahl M, Bartmann K, Bendt F, Blum J, Dolde X, Förster N, Holzer A-K, Hübenthal U, Keßel HE, Koch K, Masjosthusmann S, Schneider S, Stürzl L-C, Woeste S, Rossi A, Covaci A, Behl M, Leist M, Tigges J, Fritsche E, 2022b. Neurodevelopmental toxicity assessment of flame retardants using a human DNT in vitro testing battery. Cell Biol. Toxicol 38, 781–807. 10.1007/s10565-021-09603-2. 33969458 PMC9525352

[R85] Klose J, Tigges J, Masjosthusmann S, Schmuck K, Bendt F, Hübenthal U, Petzsch P, Köhrer K, Koch K, Fritsche E, 2021. TBBPA targets converging key events of human oligodendrocyte development resulting in two novel AOPs. ALTEX 38, 215–234. 10.14573/altex.2007201. 33099281

[R86] Koch K, Bartmann K, Hartmann J, Kapr J, Klose J, Kuchovská E, Pahl M, Schlüppmann K, Zühr E, Fritsche E, 2022. Scientific Validation of Human Neurosphere Assays for Developmental Neurotoxicity Evaluation. Front. Toxicol 4, 816370. 10.3389/ftox.2022.816370. 35295221 PMC8915868

[R87] Kong L, Zhang Y, Ye Z-Q, Liu X-Q, Zhao S-Q, Wei L, Gao G, 2007. CPC: assess the protein-coding potential of transcripts using sequence features and support vector machine. Nucleic Acids Res. 35, W345–W349. 10.1093/nar/gkm391. 17631615 PMC1933232

[R88] Lagiou P, Samoli E, Okulicz W, Xu B, Lagiou A, Lipworth L, Georgila C, Vatten L, Adami HO, Trichopoulos D, Hsieh CC, 2011. Maternal and cord blood hormone levels in the United States and China and the intrauterine origin of breast cancer. Ann. Oncol. off. J. Eur. Soc. Med. Oncol 22, 1102–1108. 10.1093/ANNONC/MDQ565. 20943596

[R89] Langseth AJ, Munji RN, Choe Y, Huynh T, Pozniak CD, Pleasure SJ, 2010. Wnts Influence the Timing and Efficiency of Oligodendrocyte Precursor Cell Generation in the Telencephalon. J. Neurosci 30, 13367–13372. 10.1523/JNEUROSCI.1934-10.2010. 20926663 PMC2954511

[R90] LeBaron MJ, Coady KK, O’Connor JC, Nabb DL, Markell LK, Snajdr S, Sue Marty M, 2014. Key Learnings from Performance of the U.S. EPA Endocrine Disruptor Screening Program (EDSP) Tier 1 In Vitro Assays. Birth Defects Res. Part B Dev. Reprod. Toxicol 101, 23–42. 10.1002/bdrb.21094. 24515815

[R91] LeWinn KZ, Stroud LR, Molnar BE, Ware JH, Koenen KC, Buka SL, 2009. Elevated maternal cortisol levels during pregnancy are associated with reduced childhood IQ. Int. J. Epidemiol 38, 1700–1710. 10.1093/ije/dyp200. 19423658 PMC2786250

[R92] Li H, Zhang J, Chen S, Wang F, Zhang T, Niswander L, 2018. Genetic contribution of retinoid-related genes to neural tube defects. Hum. Mutat 39, 550–562. 10.1002/HUMU.23397. 29297599 PMC5839987

[R93] Lin Y-L, Persaud SD, Nhieu J, Wei L-N, 2017. Cellular Retinoic Acid–Binding Protein 1 Modulates Stem Cell Proliferation to Affect Learning and Memory in Male Mice. Endocrinology 158, 3004–3014. 10.1210/en.2017-00353. 28911165 PMC5659671

[R94] Liu Y-Y, Brent GA, 2010. Thyroid hormone crosstalk with nuclear receptor signaling in metabolic regulation. Trends Endocrinol. Metab 21, 166–173. 10.1016/j.tem.2009.11.004. 20015660 PMC2831161

[R95] Liu Y, Kagechika H, Ishikawa J, Hirano H, Matsukuma S, Tanaka K, Nakamura S, 2008. Effects of retinoic acids on the dendritic morphology of cultured hippocampal neurons. J. Neurochem 106, 1104–1116. 10.1111/j.1471-4159.2008.05445.x. 18466335

[R96] López-Espíndola D, Morales-Bastos C, Grijota-Martínez C, Liao X-H, Lev D, Sugo E, Verge CF, Refetoff S, Bernal J, Guadaño-Ferraz A, 2014. Mutations of the Thyroid Hormone Transporter MCT8 Cause Prenatal Brain Damage and Persistent Hypomyelination. J. Clin. Endocrinol. Metab 99, E2799–E2804. 10.1210/jc.2014-2162. 25222753 PMC4255116

[R97] Lupu D, Andersson P, Bornehag C-G, Demeneix B, Fritsche E, Gennings C, Lichtensteiger W, Leist M, Leonards PEG, Ponsonby A-L, Scholze M, Testa G, Tresguerres JAF, Westerink RHS, Zalc B, Rüegg J, 2020. The ENDpoiNTs Project: Novel Testing Strategies for Endocrine Disruptors Linked to Developmental Neurotoxicity. Int. J. Mol. Sci 21, 3978. 10.3390/ijms21113978. 32492937 PMC7312023

[R98] Lütjohann D, Björkhem I, Locatelli S, Dame C, Schmolling J, Von Bergmann K, Fahnenstich H, 2001. Cholesterol dynamics in the foetal and neonatal brain as reflected by circulatory levels of 24S-hydroxycholesterol. Acta Paediatr. 90, 652–657. 10.1080/080352501750258720. 11440099

[R99] Maass C, Schaller S, Dallmann A, Bothe K, Müller D, 2023. Considering developmental neurotoxicity in vitro data for human health risk assessment using physiologically-based kinetic modeling: deltamethrin case study. Toxicol. Sci 192, 59–70. 10.1093/toxsci/kfad007. 36637193 PMC10025876

[R100] Manolescu DC, El-Kares R, Lakhal-Chaieb L, Montpetit A, Bhat PV, Goodyer P, 2010. Newborn Serum Retinoic Acid Level Is Associated With Variants of Genes in the Retinol Metabolism Pathway. Pediatr. Res 67, 598–602. 10.1203/PDR.0b013e3181dcf18a. 20308937

[R101] Martin EI, Ressler KJ, Binder E, Nemeroff CB, 2009. The Neurobiology of Anxiety Disorders: Brain Imaging, Genetics, and Psychoneuroendocrinology. Psychiatr. Clin. North Am 32, 549–575. 10.1016/j.psc.2009.05.004. 19716990 PMC3684250

[R102] Masjosthusmann S, Becker D, Petzuch B, Klose J, Siebert C, Deenen R, Barenys M, Baumann J, Dach K, Tigges J, Hübenthal U, Köhrer K, Fritsche E, 2018. A transcriptome comparison of time-matched developing human, mouse and rat neural progenitor cells reveals human uniqueness. Toxicol. Appl. Pharmacol 354, 40–55. 10.1016/j.taap.2018.05.009. 29753005

[R103] Masjosthusmann S, Blum J, Bartmann K, Dolde X, Holzer A-K, Stürzl L-C, Keßel EH, Förster N, Dönmez A, Klose J, Pahl M, Waldmann T, Bendt F, Kisitu J, Suciu I, Hübenthal U, Mosig A, Leist M, Fritsche E, 2020. Establishment of an a priori protocol for the implementation and interpretation of an in-vitro testing battery for the assessment of developmental neurotoxicity. EFSA Support. Publ 17, 1938E. 10.2903/SP.EFSA.2020.EN-1938.

[R104] Masjosthusmann S, Siebert C, Hübenthal U, Bendt F, Baumann J, Fritsche E, 2019. Arsenite interrupts neurodevelopmental processes of human and rat neural progenitor cells: The role of reactive oxygen species and species-specific antioxidative defense. Chemosphere 235, 447–456. 10.1016/j.chemosphere.2019.06.123. 31272005

[R105] Mathews ES, Appel B, 2016. Cholesterol Biosynthesis Supports Myelin Gene Expression and Axon Ensheathment through Modulation of P13K/Akt/mTor Signaling. J. Neurosci 36, 7628–7639. 10.1523/JNEUROSCI.0726-16.2016. 27445141 PMC4951573

[R106] McCaffery PJ, Adams J, Maden M, Rosa-Molinar E, 2003. Too much of a good thing: retinoic acid as an endogenous regulator of neural differentiation and exogenous teratogen. Eur. J. Neurosci 18, 457–472. 10.1046/J.1460-9568.2003.02765.X. 12911743

[R107] Medvedev AV, Medvedeva LA, Martsen E, Moeser M, Gorman KL, Lin B, Blackwell B, Villeneuve DL, Houck KA, Crofton KM, Makarov SS, 2020. Harmonized Cross-Species Assessment of Endocrine and Metabolic Disruptors by Ecotox FACTORIAL Assay. Environ. Sci. Technol 54, 12142–12153. 10.1021/ACS.EST.0C03375. 32901485 PMC11285471

[R108] Miller JA, Ding SL, Sunkin SM, Smith KA, Ng L, Szafer A, Ebbert A, Riley ZL, Royall JJ, Aiona K, Arnold JM, Bennet C, Bertagnolli D, Brouner K, Butler S, Caldejon S, Carey A, Cuhaciyan C, Dalley RA, Dee N, Dolbeare TA, Facer BAC, Feng D, Fliss TP, Gee G, Goldy J, Gourley L, Gregor BW, Gu G, Howard RE, Jochim JM, Kuan CL, Lau C, Lee CK, Lee F, Lemon TA, Lesnar P, McMurray B, Mastan N, Mosqueda N, Naluai-Cecchini T, Ngo NK, Nyhus J, Oldre A, Olson E, Parente J, Parker PD, Parry SE, Stevens A, Pletikos M, Reding M, Roll K, Sandman D, Sarreal M, Shapouri S, Shapovalova NV, Shen EH, Sjoquist N, Slaughterbeck CR, Smith M, Sodt AJ, Williams D, Zöllei L, Fischl B, Gerstein MB, Geschwind DH, Glass IA, Hawrylycz MJ, Hevner RF, Huang H, Jones AR, Knowles JA, Levitt P, Phillips JW, Šestan N, Wohnoutka P, Dang C, Bernard A, Hohmann JG, Lein ES, 2014. Transcriptional landscape of the prenatal human brain. Nature 508, 199–206. 10.1038/NATURE13185. 24695229 PMC4105188

[R109] Miranda A, Sousa N, 2018. Maternal hormonal milieu influence on fetal brain development. Brain Behav. 8, e00920. 10.1002/BRB3.920. 29484271 PMC5822586

[R110] Moisiadis VG, Matthews SG, 2014. Glucocorticoids and fetal programming part 1: outcomes. Nat. Rev. Endocrinol 10, 391–402. 10.1038/nrendo.2014.73. 24863382

[R111] Moors M, Bose R, Johansson-Haque K, Edoff K, Okret S, Ceccatelli S, 2012. Dickkopf 1 Mediates Glucocorticoid-Induced Changes in Human Neural Progenitor Cell Proliferation and Differentiation. Toxicol. Sci 125, 488–495. 10.1093/toxsci/kfr304. 22048647

[R112] Mounier A, Georgiev D, Nam KN, Fitz NF, Castranio EL, Wolfe CM, Cronican AA, Schug J, Lefterov I, Koldamova R, 2015. Bexarotene-Activated Retinoid X Receptors Regulate Neuronal Differentiation and Dendritic Complexity. J. Neurosci 35, 11862–11876. 10.1523/JNEUROSCI.1001-15.2015. 26311769 PMC4549399

[R113] Namba N, Etani Y, Kitaoka T, Nakamoto Y, Nakacho M, Bessho K, Miyoshi Y, Mushiake S, Mohri I, Arai H, Taniike M, Ozono K, 2008. Clinical phenotype and endocrinological investigations in a patient with a mutation in the MCT8 thyroid hormone transporter. Eur. J. Pediatr 167, 785–791. 10.1007/s00431-007-0589-6. 17899191

[R114] Norton WT, Poduslo SE, 1973. Myelination in rat brain: changes in myelin composition during brain maturation. J. Neurochem 21, 759–773. 10.1111/j.1471-4159.1973.tb07520.x. 4754856

[R115] NRC, 2007. Toxicity Testing in the 21st Century, Toxicity Testing in the 21st Century: A Vision and a Strategy. National Academies Press, Washington, D.C. doi: 10.17226/11970.

[R116] OECD, 2023. Initial Recommendations on Evaluation of Data from the Developmental Neurotoxicity (DNT) In-Vitro Testing Battery.

[R117] OECD, 2018a. Revised Guidance Document 150 on Standardised Test Guidelines for Evaluating Chemicals for Endocrine Disruption, OECD Series on Testing and Assessment, OECD Series on Testing and Assessment. OECD, Paris. doi: 10.1787/9789264304741-en.

[R118] OECD, 2018b. Test No. 443: Extended One-Generation Reproductive Toxicity Study. doi: doi: 10.1787/20745788.

[R119] OECD, 2007. Test No. 426: Developmental Neurotoxicity Study, in: OECD Guidelines for the Testing of Chemicals, Section 4. OECD. doi: 10.1787/9789264067394-en.

[R120] Özel F, Rüegg J, 2023. Exposure to endocrine-disrupting chemicals and implications for neurodevelopment. Dev. Med. Child Neurol 65, 1005–1011. 10.1111/dmcn.15551. 36808586

[R121] Paparella M, Bennekou SH, Bal-Price A, 2020. An analysis of the limitations and uncertainties of in vivo developmental neurotoxicity testing and assessment to identify the potential for alternative approaches. Reprod. Toxicol 96, 327–336. 10.1016/j.reprotox.2020.08.002. 32781019

[R122] Pearson Murphy BE, 1983. Human fetal serum cortisol levels at delivery: a review. Endocr. Rev 4, 150–154. 10.1210/edrv-4-2-150. 6345146

[R123] Pierfelice T, Alberi L, Gaiano N, 2011. Notch in the Vertebrate Nervous System: An Old Dog with New Tricks. Neuron 69, 840–855. 10.1016/j.neuron.2011.02.031. 21382546

[R124] Ramhøj L, Axelstad M, Baert Y, Cañas-Portilla AI, Chalmel F, Dahmen L, De La Vieja A, Evrard B, Haigis A-C, Hamers T, Heikamp K, Holbech H, Iglesias-Hernandez P, Knapen D, Marchandise L, Morthorst JE, Nikolov NG, Nissen ACVE, Oelgeschlaeger M, Renko K, Rogiers V, Schüürmann G, Stinckens E, Stub MH, Torres-Ruiz M, Van Duursen M, Vanhaecke T, Vergauwen L, Wedebye EB, Svingen T, 2023. New approach methods to improve human health risk assessment of thyroid hormone system disruption-a PARC project. Front. Toxicol 5, 1189303. 10.3389/ftox.2023.1189303. 37265663 PMC10229837

[R125] Ribeiro RC, Kushner PJ, Baxter JD, 1995. The nuclear hormone receptor gene superfamily. Annu. Rev. Med 46, 443–453. 10.1146/annurev.med.46.1.443. 7598477

[R126] Rice D, Barone S, 2000. Critical periods of vulnerability for the developing nervous system: evidence from humans and animal models. Environ. Health Perspect. 108, 511–533. 10.1289/ehp.00108s3511. 10852851 PMC1637807

[R127] Ritz C, Baty F, Streibig JC, Gerhard D, 2015. Dose-Response Analysis Using R. PLoS One 10, e0146021. 10.1371/journal.pone.0146021. 26717316 PMC4696819

[R128] Rivollier F, Krebs M-O, Kebir O, 2019. Perinatal Exposure to Environmental Endocrine Disruptors in the Emergence of Neurodevelopmental Psychiatric Diseases: A Systematic Review. Int. J. Environ. Res. Public Health 16, 1318. 10.3390/ijerph16081318. 31013727 PMC6517937

[R129] Sachana M, Bal-Price A, Crofton KM, Bennekou SH, Shafer TJ, Behl M, Terron A, 2019. International Regulatory and Scientific Effort for Improved Developmental Neurotoxicity Testing. Toxicol. Sci 167, 45–57. 10.1093/toxsci/kfy211. 30476307

[R130] Sachana M, Shafer TJ, Terron A, 2021. Toward a Better Testing Paradigm for Developmental Neurotoxicity: OECD Efforts and Regulatory Considerations. Biology (basel). 10, 86. 10.3390/biology10020086. 33498772 PMC7912397

[R131] Saher G, Rudolphi F, Corthals K, Ruhwedel T, Schmidt K-F, Löwel S, Dibaj P, Barrette B, Möbius W, Nave K-A, 2012. Therapy of Pelizaeus-Merzbacher disease in mice by feeding a cholesterol-enriched diet. Nat. Med 18, 1130–1135. 10.1038/nm.2833. 22706386

[R132] Sakamoto M, Chan HM, Domingo JL, Koriyama C, Murata K, 2018. Placental transfer and levels of mercury, selenium, vitamin E, and docosahexaenoic acid in maternal and umbilical cord blood. Environ. Int 111, 309–315. 10.1016/j.envint.2017.11.001. 29150340

[R133] Sauer UG, Asiimwe A, Botham PA, Charlton A, Hallmark N, Jacobi S, Marty S, Melching-Kollmuss S, Palha JA, Strauss V, van Ravenzwaay B, Swaen G, 2020. Toward a science-based testing strategy to identify maternal thyroid hormone imbalance and neurodevelopmental effects in the progeny - part I: which parameters from human studies are most relevant for toxicological assessments? Crit. Rev. Toxicol 50, 740–763. 10.1080/10408444.2020.1839380. 33305658

[R134] Schantz SL, Widholm JJ, Rice DC, 2003. Effects of PCB exposure on neuropsychological function in children. Environ. Health Perspect 111, 357–376. 10.1289/EHP.5461. 12611666 PMC1241394

[R135] Schlagenhauf A, Haidl H, Leschnik B, Leis H-J, Heinemann A, Muntean W, 2015. Prostaglandin E2 levels and platelet function are different in cord blood compared to adults. Thromb. Haemost. 113, 97–106. 10.1160/TH14-03-0218. 25118631

[R136] Schmuck MR, Temme T, Dach K, de Boer D, Barenys M, Bendt F, Mosig A, Fritsche E, 2017. Omnisphero: a high-content image analysis (HCA) approach for phenotypic developmental neurotoxicity (DNT) screenings of organoid neurosphere cultures in vitro. Arch. Toxicol 91, 2017–2028. 10.1007/s00204-016-1852-2. 27722930

[R137] Schneider CA, Rasband WS, Eliceiri KW, 2012. NIH Image to ImageJ: 25 years of image analysis. Nat. Methods 9, 671–675. 10.1038/nmeth.2089. 22930834 PMC5554542

[R138] Schreiber T, Gassmann K, Götz C, Hübenthal U, Moors M, Krause G, Merk HF, Nguyen N-H, Scanlan TS, Abel J, Rose CR, Fritsche E, 2010. Polybrominated diphenyl ethers induce developmental neurotoxicity in a human in vitro model: evidence for endocrine disruption. Environ. Health Perspect 118, 572–578. 10.1289/ehp.0901435. 20368126 PMC2854737

[R139] Schug TT, Blawas AM, Gray K, Heindel JJ, Lawler CP, 2015. Elucidating the Links Between Endocrine Disruptors and Neurodevelopment. Endocrinology 156, 1941–1951. 10.1210/en.2014-1734. 25714811 PMC5393340

[R140] Shah V, Nguyen P, Nguyen N-H, Togashi M, Scanlan TS, Baxter JD, Webb P, 2008. Complex actions of thyroid hormone receptor antagonist NH-3 on gene promoters in different cell lines. Mol. Cell. Endocrinol 296, 69–77. 10.1016/j.mce:2008.09.016. 18930112 PMC4180716

[R141] Shibata M, Pattabiraman K, Lorente-Galdos B, Andrijevic D, Kim S-K, Kaur N, Muchnik SK, Xing X, Santpere G, Sousa AMM, Sestan N, 2021. Regulation of prefrontal patterning and connectivity by retinoic acid. Nature 598, 483–488. 10.1038/s41586-021-03953-x. 34599305 PMC9018119

[R142] Silbereis JC, Pochareddy S, Zhu Y, Li M, Sestan N, 2016. The Cellular and Molecular Landscapes of the Developing Human Central Nervous System. Neuron. 10.1016/j.neuron.2015.12.008. PMC4959909 26796689

[R143] Stagni F, Giacomini A, Emili M, Guidi S, Bartesaghi R, 2018. Neurogenesis impairment: An early developmental defect in Down syndrome. Free Radic. Biol. Med 114, 15–32. 10.1016/j.freeradbiomed.2017.07.026. 28756311

[R144] Stolt CC, Rehberg S, Ader M, Lommes P, Riethmacher D, Schachner M, Bartsch U, Wegner M, 2002. Terminal differentiation of myelin-forming oligodendrocytes depends on the transcription factor Sox10. Genes Dev. 16, 165–170. 10.1101/GAD.215802. 11799060 PMC155320

[R145] Strosznajder AK, Wójtowicz S, Jeżyna MJ, Sun GY, Strosznajder JB, 2021. Recent Insights on the Role of PPAR-β/δ in Neuroinflammation and Neurodegeneration, and Its Potential Target for Therapy. NeuroMolecular Med. 23, 86–98. 10.1007/s12017-020-08629-9. 33210212 PMC7929960

[R146] Sundberg M, Savola S, Hienola A, Korhonen L, Lindholm D, 2006. Glucocorticoid Hormones Decrease Proliferation of Embryonic Neural Stem Cells through Ubiquitin-Mediated Degradation of Cyclin D1. J. Neurosci 26, 5402–5410. 10.1523/JNEUROSCI.4906-05.2006. 16707792 PMC6675314

[R147] Tanner EM, Hallerbäck MU, Wikström S, Lindh C, Kiviranta H, Gennings C, Bornehag C-G, 2020. Early prenatal exposure to suspected endocrine disruptor mixtures is associated with lower IQ at age seven. Environ. Int 134, 105185. 10.1016/j.envint.2019.105185. 31668669

[R148] Theofilopoulos S, Wang Y, Kitambi SS, Sacchetti P, Sousa KM, Bodin K, Kirk J, Saltó C, Gustafsson M, Toledo EM, Karu K, Gustafsson J-Å, Steffensen KR, Ernfors P, Sjövall J, Griffiths WJ, Arenas E, 2013. Brain endogenous liver X receptor ligands selectively promote midbrain neurogenesis. Nat. Chem. Biol 9, 126–133. 10.1038/nchembio.1156. 23292650

[R149] van Tilborg E, Heijnen CJ, Benders MJ, van Bel F, Fleiss B, Gressens P, Nijboer CH, 2016. Impaired oligodendrocyte maturation in preterm infants: Potential therapeutic targets. Prog. Neurobiol 136, 28–49. 10.1016/j.pneurobio.2015.11.002. 26655283

[R150] Vorhees CV, Sprowles JN, Regan SL, Williams MT, 2018. A better approach to in vivo developmental neurotoxicity assessment: Alignment of rodent testing with effects seen in children after neurotoxic exposures. Toxicol. Appl. Pharmacol 354, 176–190. 10.1016/J.TAAP.2018.03.012. 29544898

[R151] Walter KM, Dach K, Hayakawa K, Giersiefer S, Heuer H, Lein PJ, Fritsche E, 2019. Ontogenetic expression of thyroid hormone signaling genes: An in vitro and in vivo species comparison. PLoS One 14. 10.1371/JOURNAL.PONE.0221230. PMC6742404 31513589

[R152] Wang SL, Su PH, Jong SB, Guo YL, Chou WL, Päpke O, 2005. In utero exposure to dioxins and polychlorinated biphenyls and its relations to thyroid function and growth hormone in newborns. Environ. Health Perspect 113, 1645–1650. 10.1289/EHP.7994. 16263525 PMC1310932

[R153] Waring RH, Harris RM, 2005. Endocrine disrupters: a human risk? Mol. Cell. Endocrinol 244, 2–9. 10.1016/J.MCE.2005.02.007. 16271281

[R154] Webb P, Nguyen N-H, Chiellini G, Yoshihara HAI, Cunha Lima ST, Apriletti JW, Ribeiro RCJ, Marimuthu A, West BL, Goede P, Mellstrom K, Nilsson S, Kushner PJ, Fletterick RJ, Scanlan TS, Baxter JD, 2002. Design of thyroid hormone receptor antagonists from first principles. J. Steroid Biochem. Mol. Biol 83, 59–73. 10.1016/S0960-0760(02)00270-4. 12650702

[R155] Whitten PL, Patisaul HB, 2001. Cross-species and interassay comparisons of phytoestrogen action. Environ. Health Perspect 109 (Suppl), 5–20. 10.1289/ehp.01109s15. 11250801 PMC1240538

[R156] Wirtz S, Schuelke M, 2011. Region-Specific Expression of Mitochondrial Complex I Genes during Murine Brain Development. PLoS One 6, e18897. 10.1371/journal.pone.0018897. 21556144 PMC3083399

[R157] Wong RS, Tung KTS, Mak RTW, Leung WC, Yam JC, Chua GT, Fung GPG, Ho MHK, Wong ICK, Ip P, 2022. Vitamin D concentrations during pregnancy and in cord blood: a systematic review and meta-analysis. Nutr. Rev 80, 2225–2236. 10.1093/nutrit/nuac023. 35442446

[R158] Workman AD, Charvet CJ, Clancy B, Darlington RB, Finlay BL, 2013. Modeling transformations of neurodevelopmental sequences across mammalian species. J. Neurosci 33, 7368–7383. 10.1523/JNEUROSCI.5746-12.2013. 23616543 PMC3928428

[R159] Xu CQ, de la Monte SM, Tong M, Huang CK, Kim M, 2015. Chronic Ethanol-Induced Impairment of Wnt/β-Catenin Signaling is Attenuated by PPAR-δ Agonist. Alcohol. Clin. Exp. Res 39, 969–979. 10.1111/ACER.12727. 25903395 PMC4452420

[R160] Xu P, Xu H, Tang X, Xu L, Wang Y, Guo L, Yang Z, Xing Y, Wu Y, Warner M, Gustafsson J-A, Fan X, 2014. Liver X receptor β is essential for the differentiation of radial glial cells to oligodendrocytes in the dorsal cortex. Mol. Psychiatry 19, 947–957. 10.1038/mp.2014.60. 24934178

[R161] Ying M, Sang Y, Li Y, Guerrero-Cazares H, Quinones-Hinojosa A, Vescovi AL, Eberhart CG, Xia S, Laterra J, 2011. Krüppel-Like Family of Transcription Factor 9, a Differentiation-Associated Transcription Factor, Suppresses Notch1 Signaling and Inhibits Glioblastoma-Initiating Stem Cells. Stem Cells 29, 20–31. 10.1002/stem.561. 21280156 PMC3516843

[R162] Zhuang W, Ye T, Wang W, Song W, Tan T, 2023. CTNNB1 in neurodevelopmental disorders. Front. Psychiatry 14, 1143328. 10.3389/fpsyt.2023.1143328. 37009120 PMC10061110

[R163] Zoeller RT, Dowling ALS, Vas AA, 2000. Developmental exposure to polychlorinated biphenyls exerts thyroid hormone-like effects on the expression of RC3/neurogranin and myelin basic protein messenger ribonucleic acids in the developing rat brain. Endocrinology 141, 181–189. 10.1210/ENDO.141.1.7273. 10614638

