# Supplementary material for: Nuclear hormone receptors control fundamental processes of human fetal neurodevelopment: Basis for endocrine disruption assessment
Source: Environ Int. Author manuscript; Available in PMC 2025 Jun 2. (PMC12127433; doi:10.1016/j.envint.2025.109400)
Supplement: 1 [file NIHMS2077722-supplement-1.pdf]

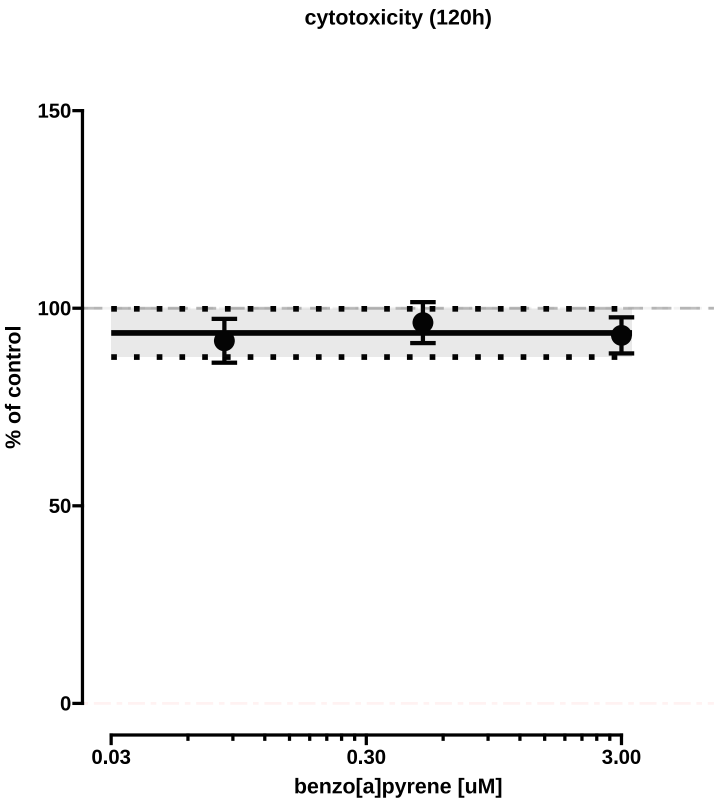

Model: 1-Parameter
Model abbr.: lm.1
Bechmark-Response (BMR): 10

BMCL: NA
BMC: NA
BMCU: NA

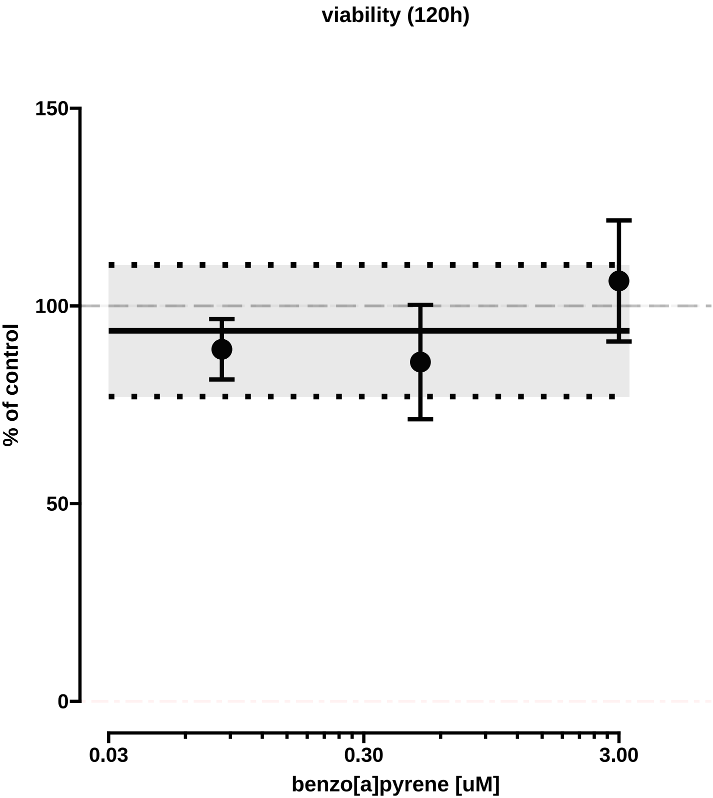

Model: 1-Parameter
Model abbr.: lm.1
Bechmark-Response (BMR): 20

BMCL: NA
BMC: NA
BMCU: NA

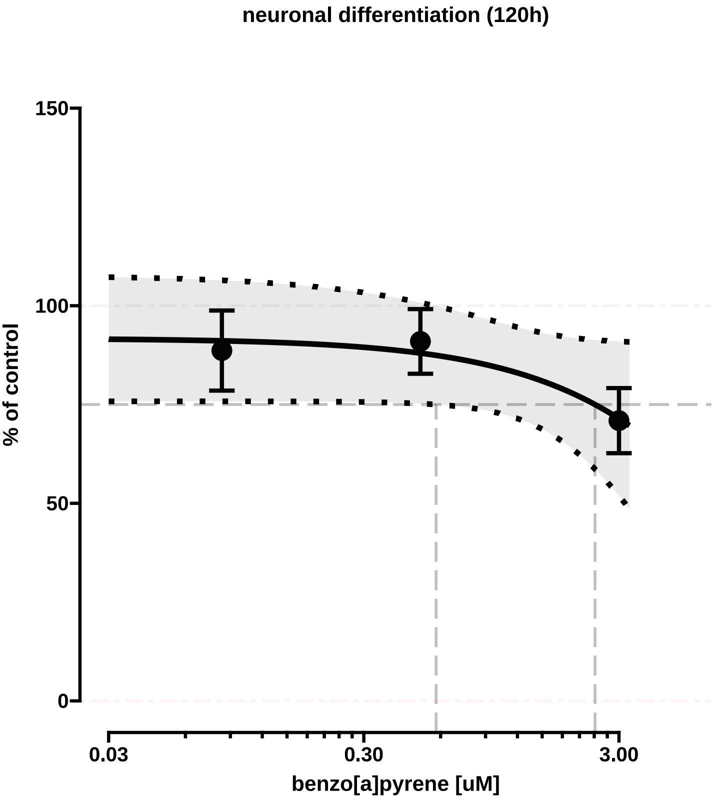

Model: Exponential decay with lower limit at 0
Model abbr.: EXD.2()
Bechmark-Response (BMR): 25

BMCL: 0.576
BMC: 2.417
BMCU: NA

oligodendrocyte differentiation (120h)

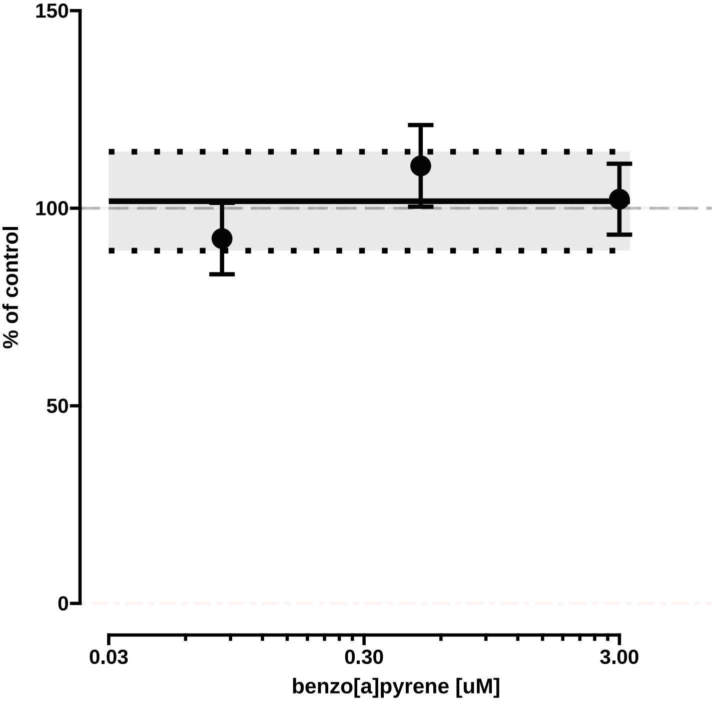

Model: 1-Parameter
Model abbr.: lm.1
Bechmark-Response (BMR): 25

BMCL: NA
BMC: NA
BMCU: NA

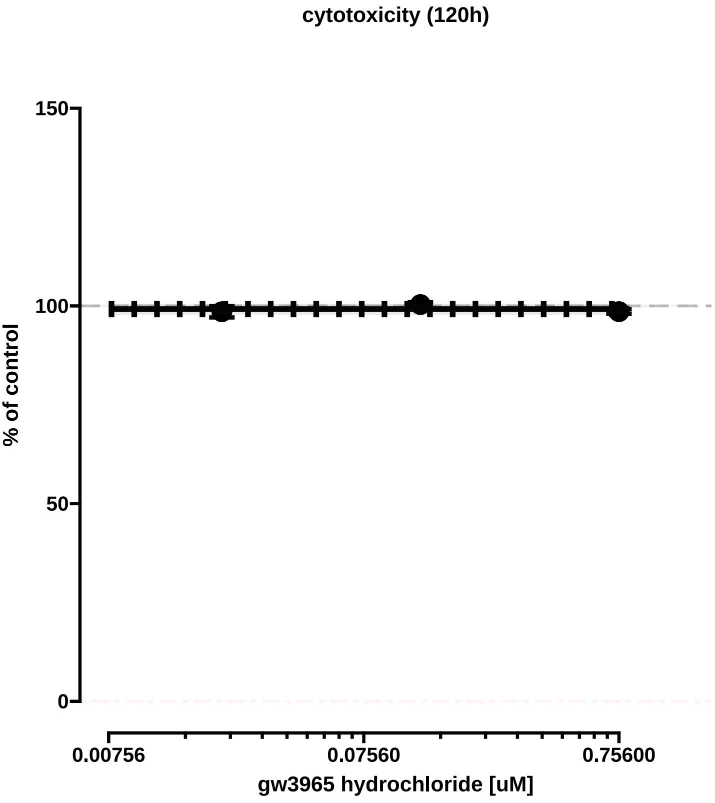

Model: 1-Parameter
Model abbr.: lm.1
Bechmark-Response (BMR): 10

BMCL: NA
BMC: NA
BMCU: NA

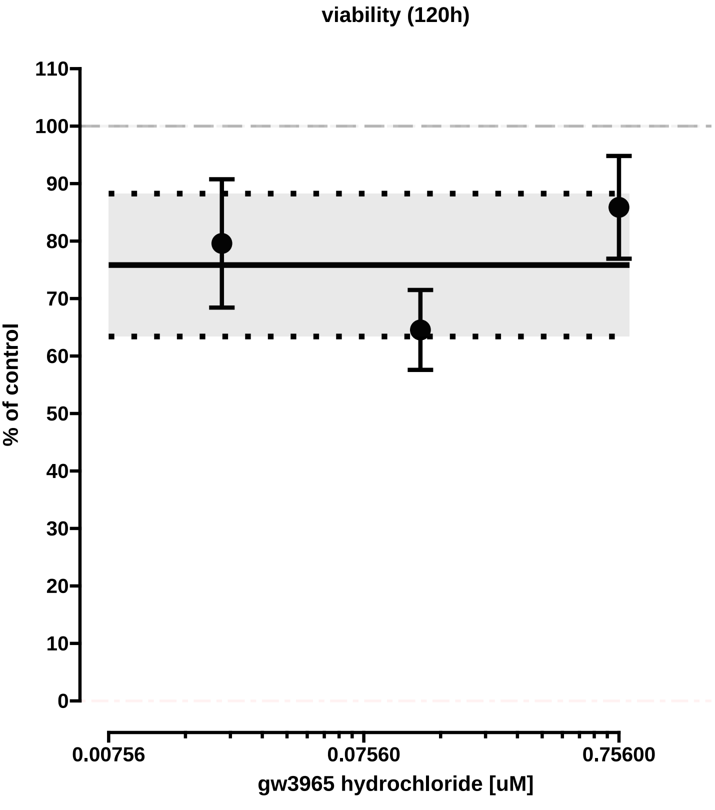

Model: 1-Parameter
Model abbr.: lm.1
Bechmark-Response (BMR): 20

BMCL: NA
BMC: NA
BMCU: NA

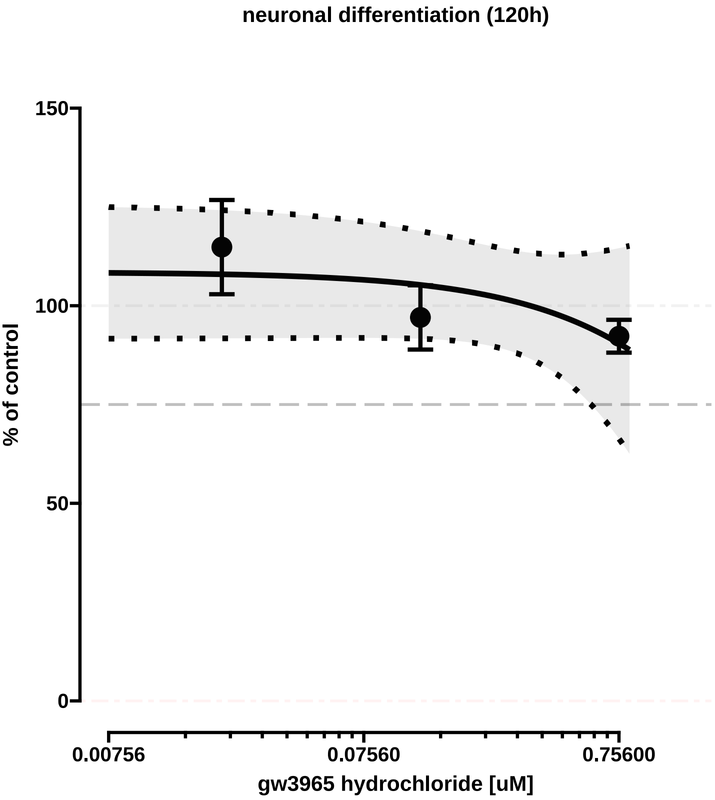

Model: Exponential decay with lower limit at 0
Model abbr.: EXD.2()
Bechmark-Response (BMR): 25

BMCL: NA
BMC: NA
BMCU: NA

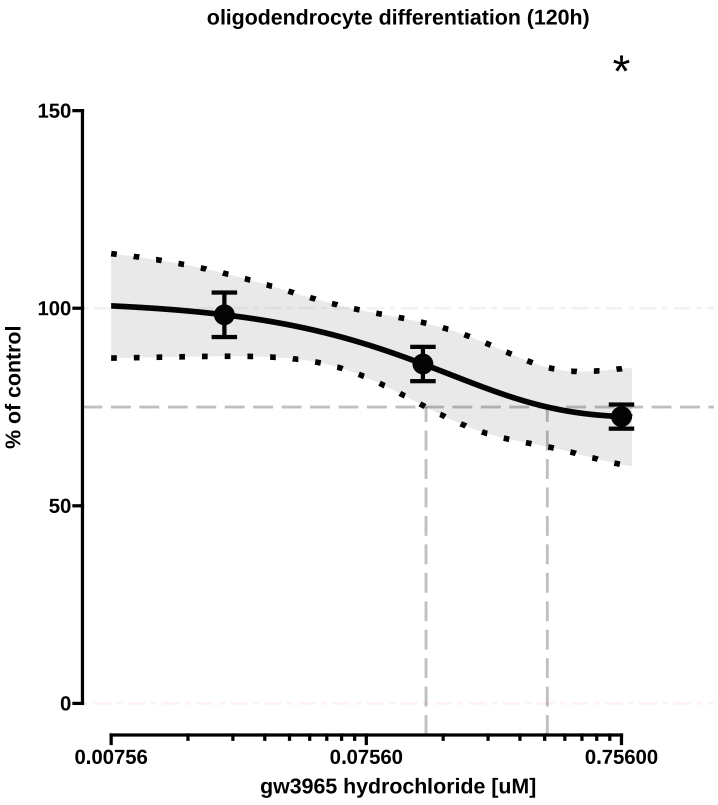

Model: Shifted exponential decay
Model abbr.: EXD.3()
Bechmark-Response (BMR): 25

BMCL: 0.13
BMC: 0.387
BMCU: NA

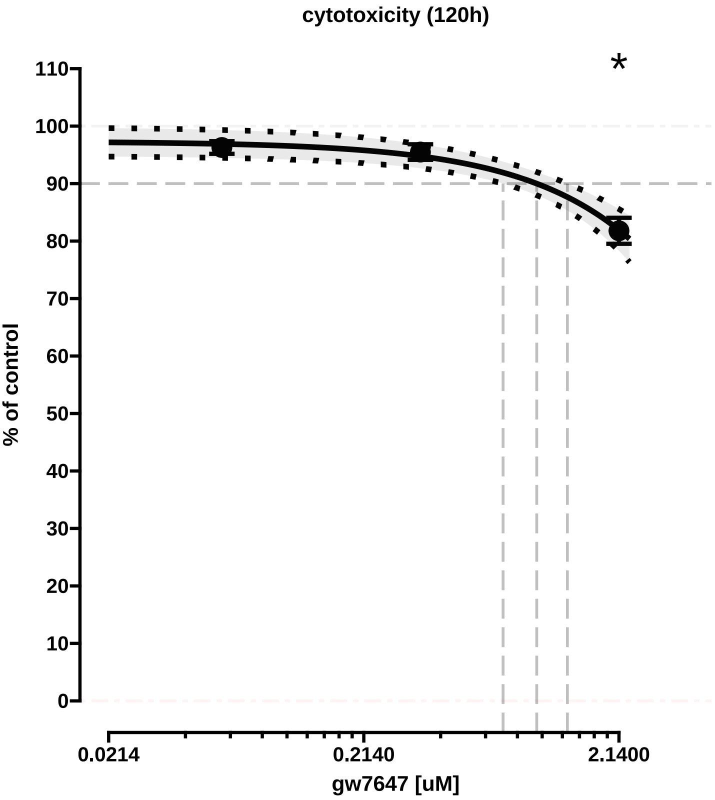

Model: Linear
Model abbr.: lm
Bechmark-Response (BMR): 10

BMCL: 0.752
BMC: 1.019
BMCU: 1.344

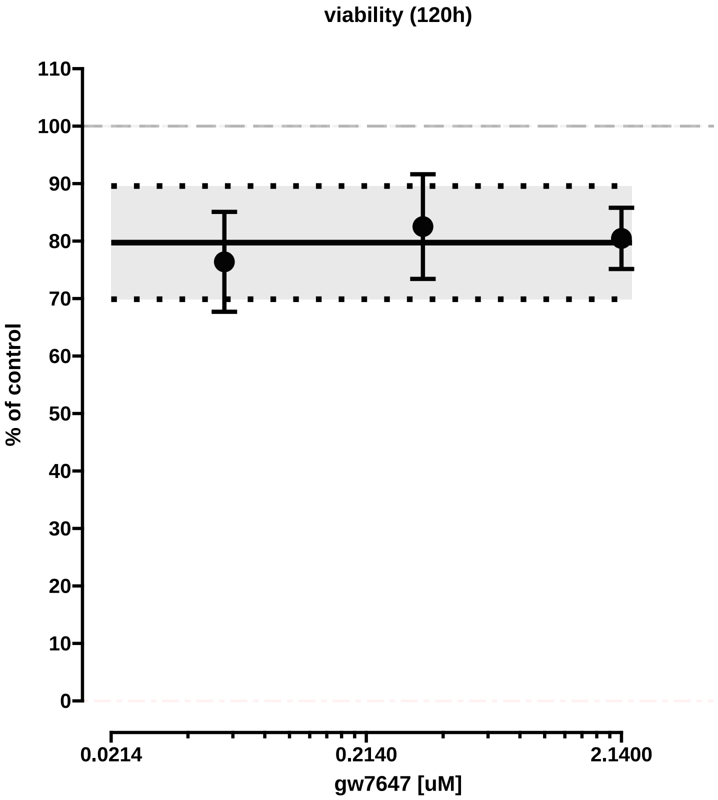

Model: 1-Parameter
Model abbr.: lm.1
Bechmark-Response (BMR): 20

BMCL: NA
BMC: NA
BMCU: NA

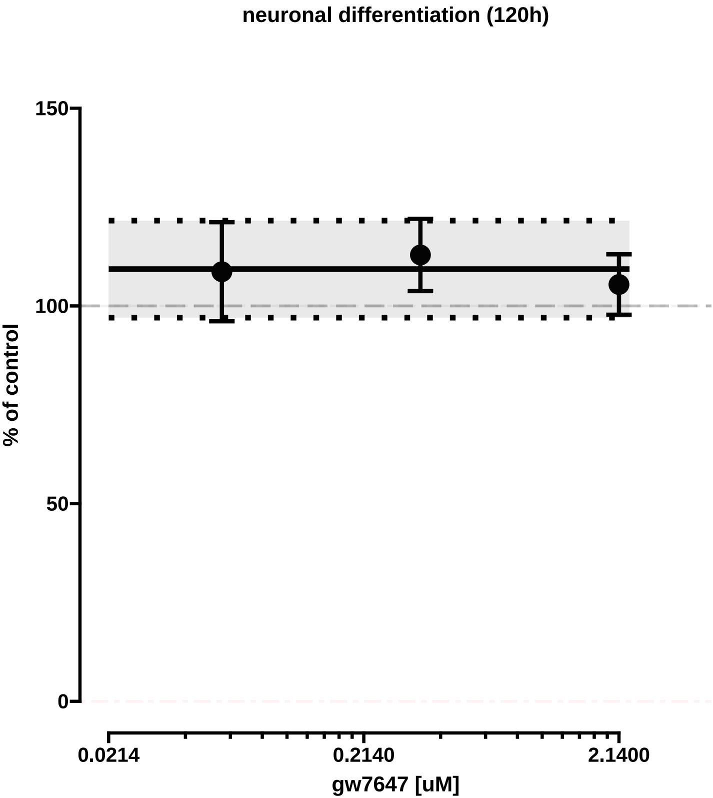

Model: 1-Parameter
Model abbr.: lm.1
Bechmark-Response (BMR): 25

BMCL: NA
BMC: NA
BMCU: NA

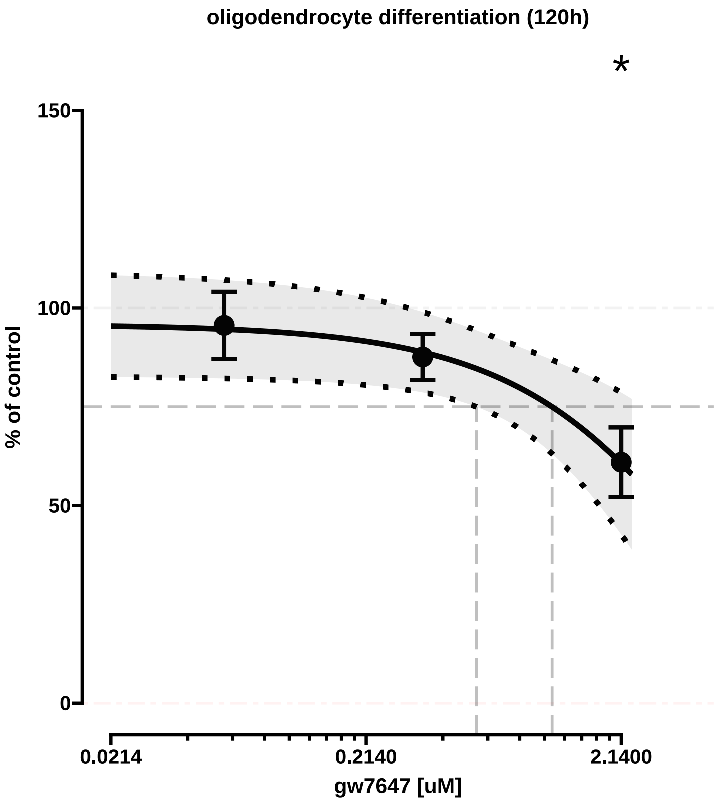

Model: Exponential decay with lower limit at 0
Model abbr.: EXD.2()
Bechmark-Response (BMR): 25

BMCL: 0.579
BMC: 1.147
BMCU: NA

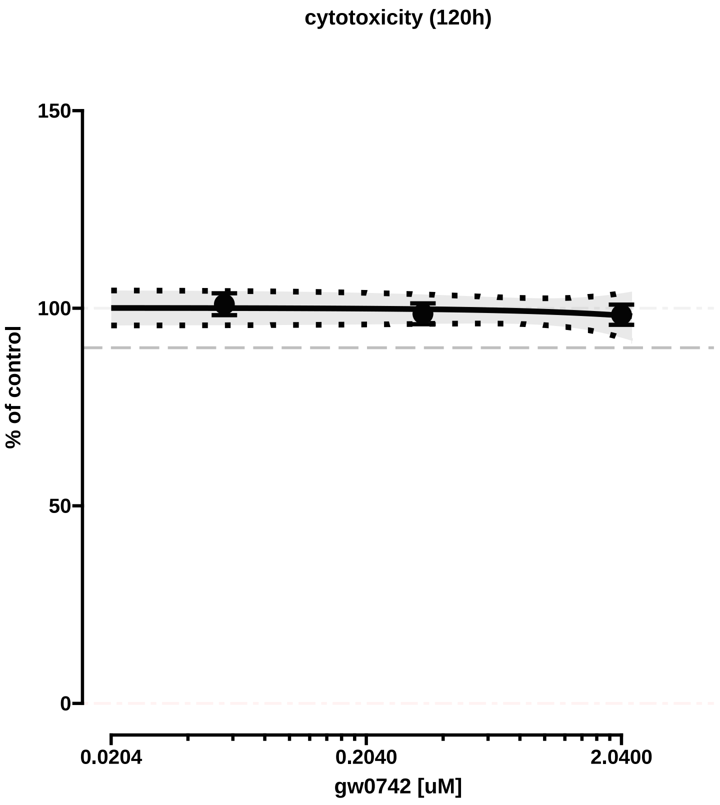

Model: Exponential decay with lower limit at 0
Model abbr.: EXD.2()
Bechmark-Response (BMR): 10

BMCL: NA
BMC: NA
BMCU: NA

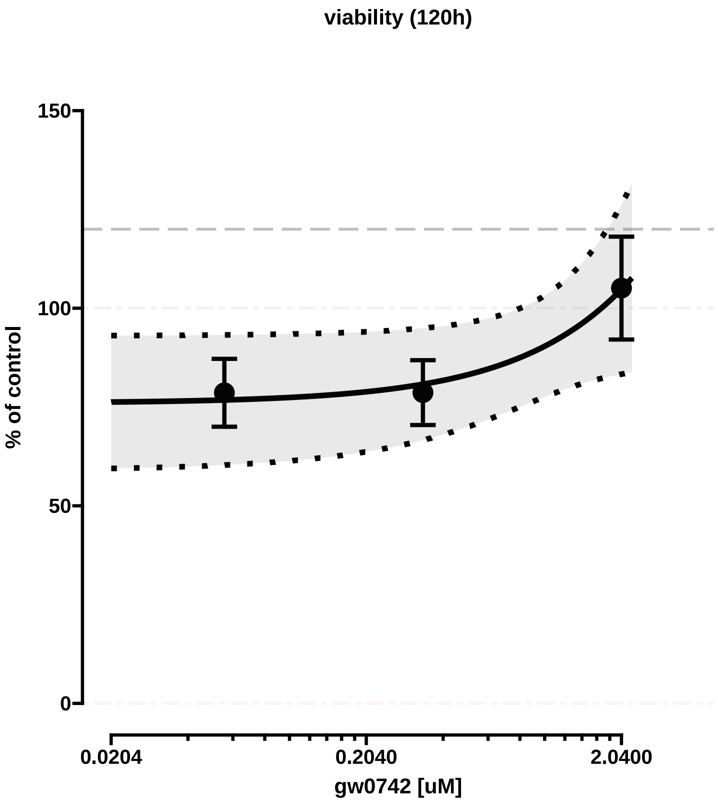

Model: Linear
Model abbr.: lm
Bechmark-Response (BMR): 20

BMCL: NA
BMC: NA
BMCU: NA

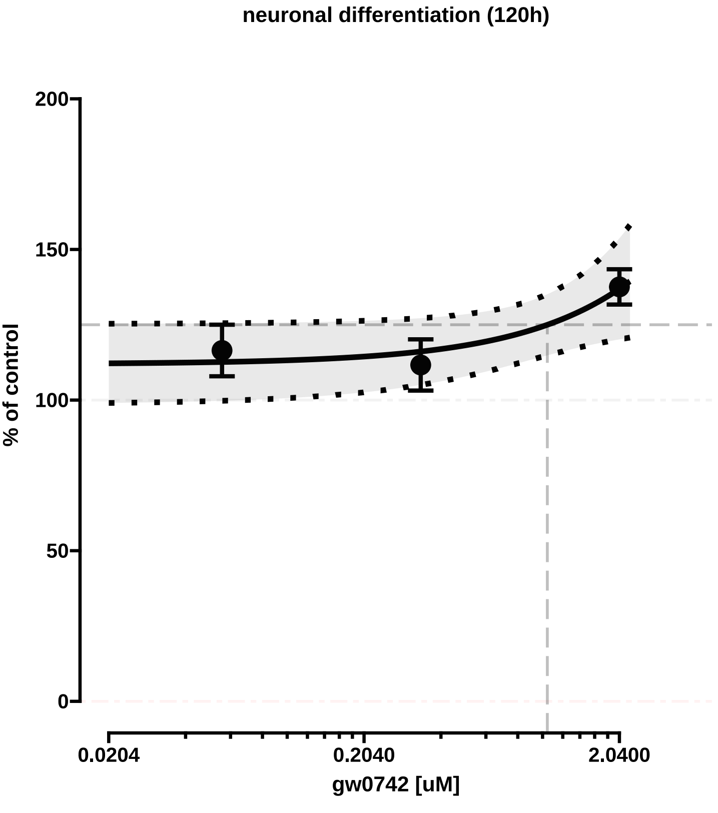

Model: Linear
Model abbr.: lm
Bechmark-Response (BMR): 25

BMCL: NA
BMC: 1.065
BMCU: NA

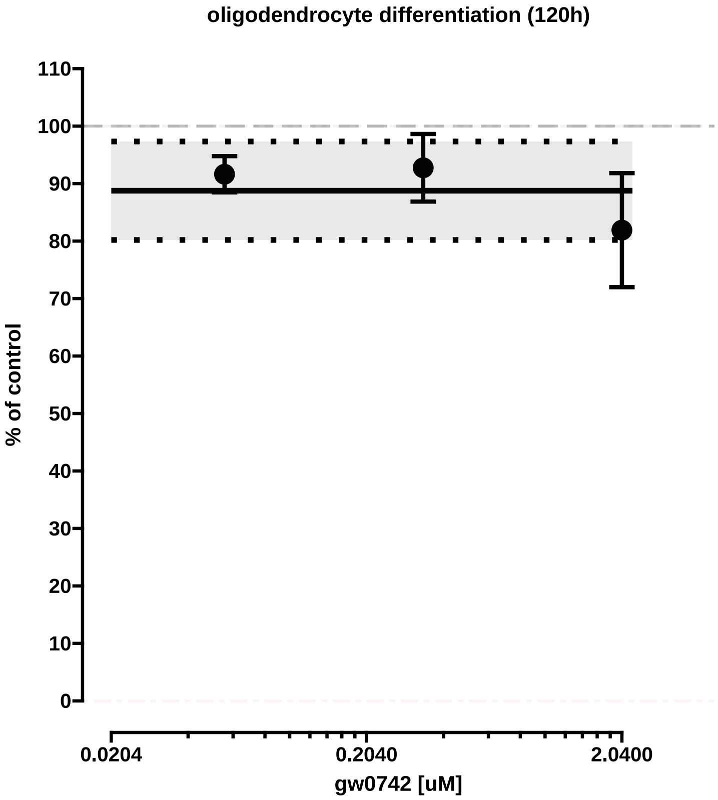

Model: 1-Parameter
Model abbr.: lm.1
Bechmark-Response (BMR): 25

BMCL: NA
BMC: NA
BMCU: NA

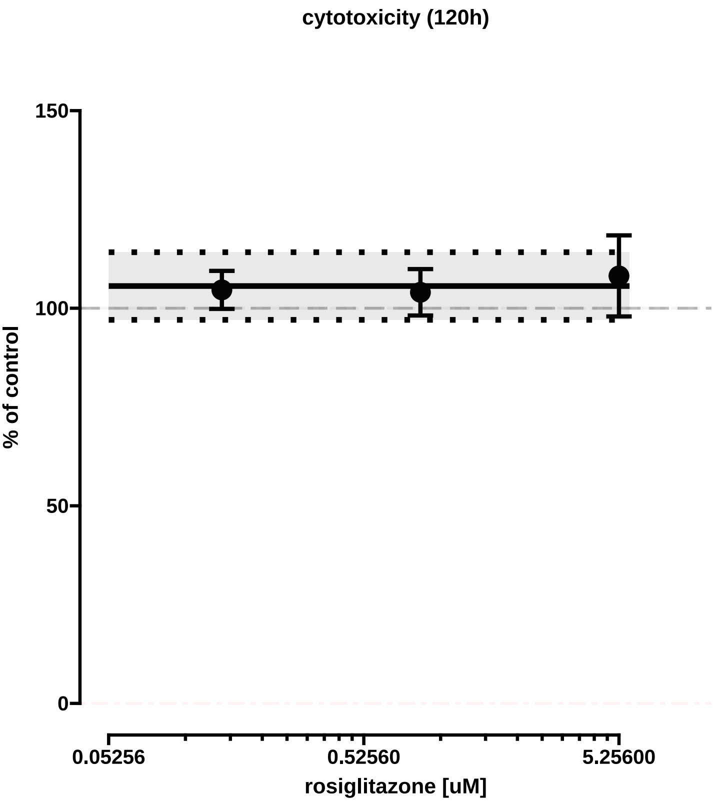

Model: 1-Parameter
Model abbr.: lm.1
Bechmark-Response (BMR): 10

BMCL: NA
BMC: NA
BMCU: NA

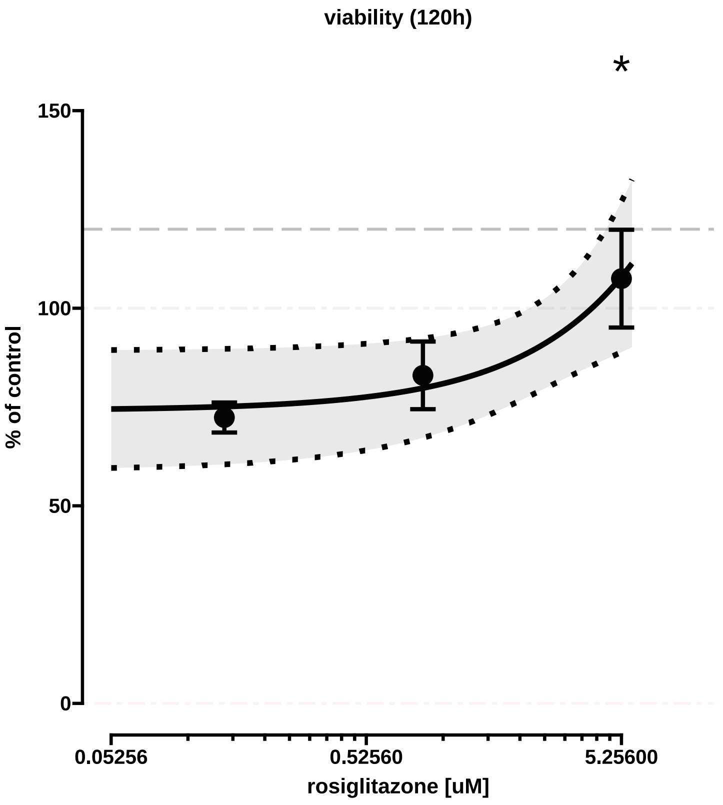

Model: Linear
Model abbr.: lm
Bechmark-Response (BMR): 20

BMCL: NA
BMC: NA
BMCU: NA

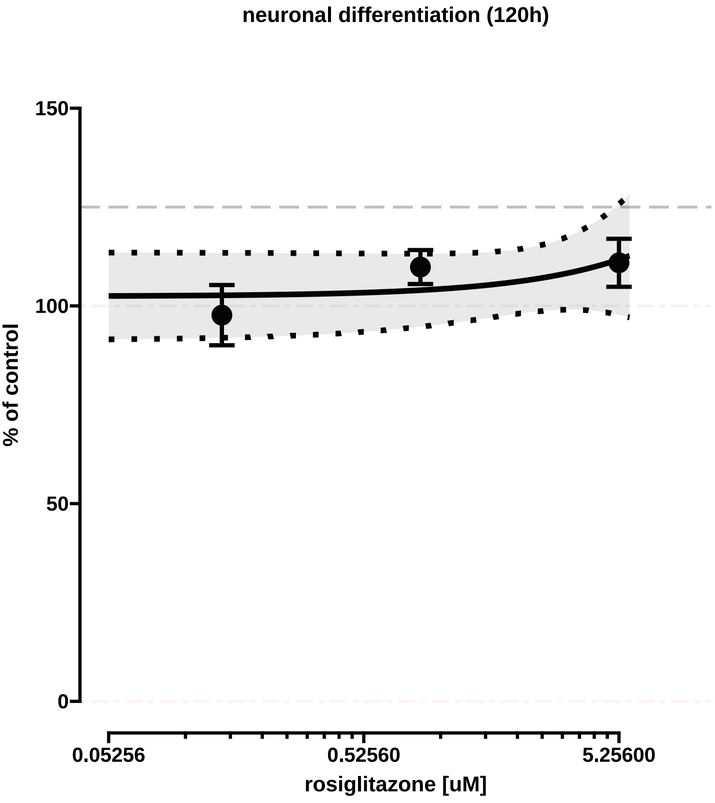

Model: Linear
Model abbr.: lm
Bechmark-Response (BMR): 25

BMCL: NA
BMC: NA
BMCU: NA

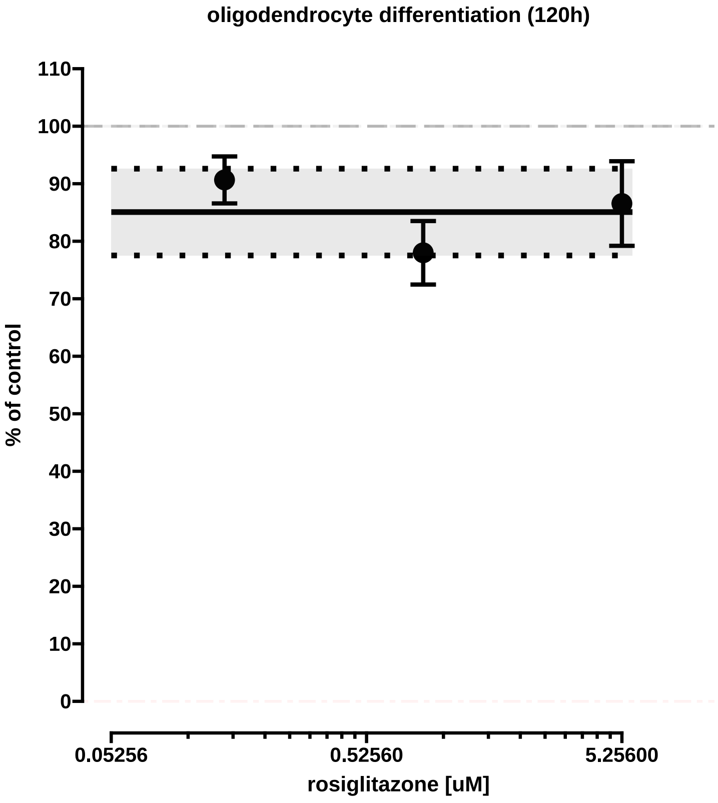

Model: 1-Parameter
Model abbr.: lm.1
Bechmark-Response (BMR): 25

BMCL: NA
BMC: NA
BMCU: NA

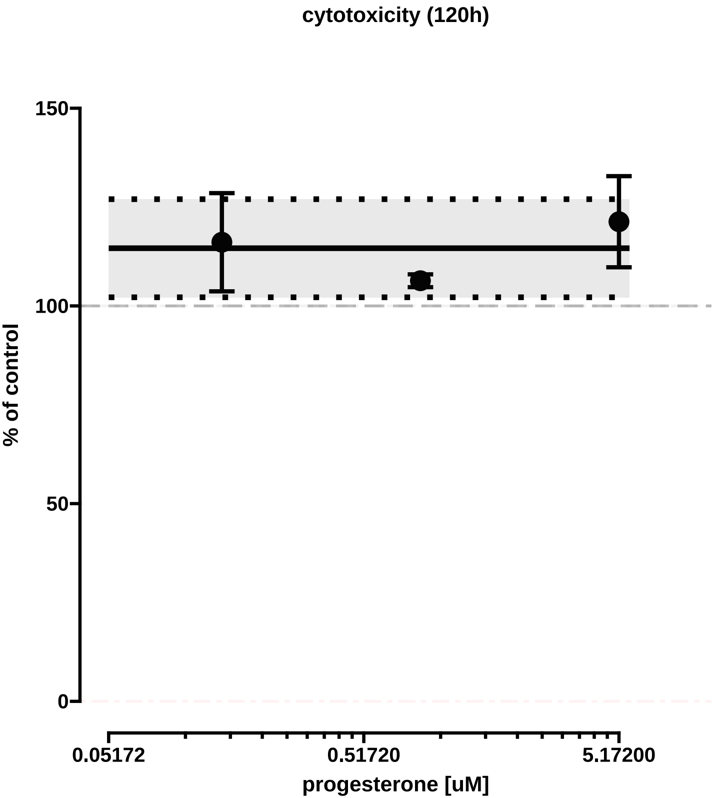

Model: 1-Parameter
Model abbr.: lm.1
Bechmark-Response (BMR): 10

BMCL: NA
BMC: NA
BMCU: NA

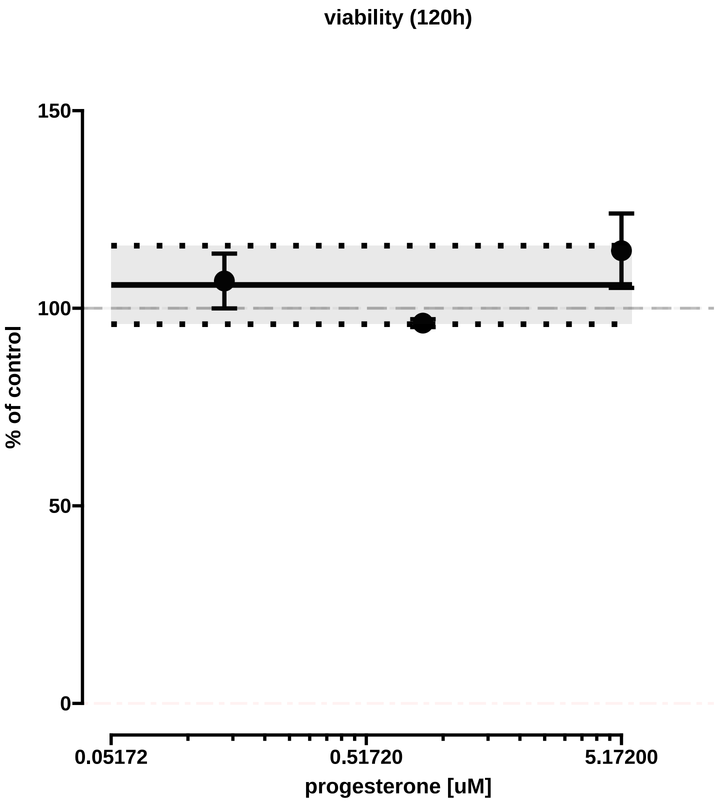

Model: 1-Parameter
Model abbr.: lm.1
Bechmark-Response (BMR): 20

BMCL: NA
BMC: NA
BMCU: NA

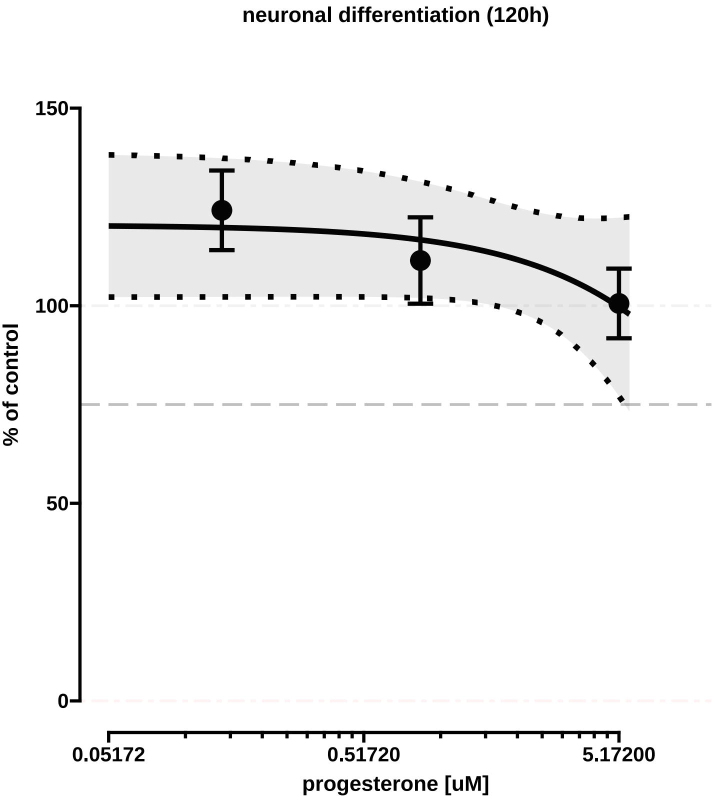

Model: Exponential decay with lower limit at 0
Model abbr.: EXD.2()
Bechmark-Response (BMR): 25

BMCL: NA
BMC: NA
BMCU: NA

oligodendrocyte differentiation (120h)

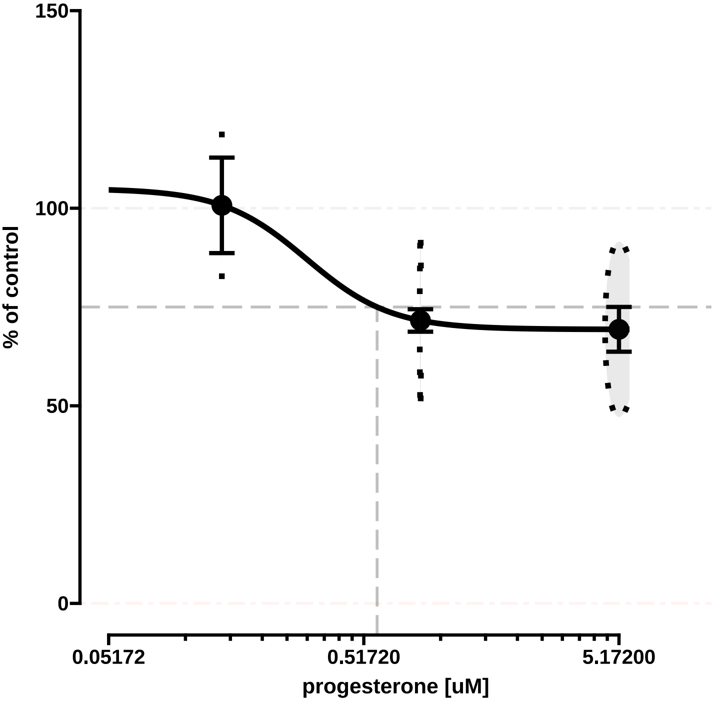

Model: Log-logistic (log(ED50) as parameter)
Model abbr.: LL2.4()
Bechmark-Response (BMR): 25

BMCL: NA
BMC: 0.583
BMCU: NA

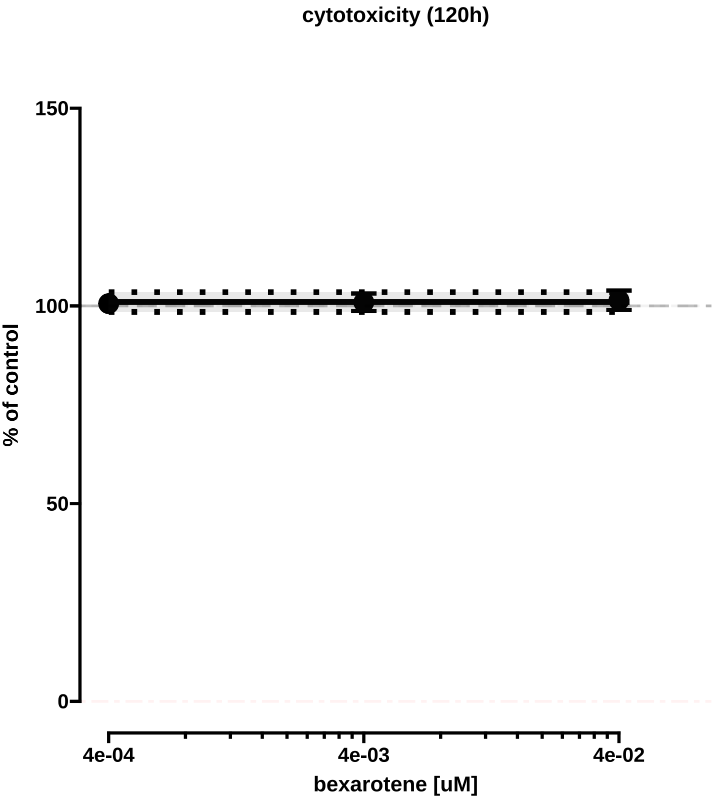

Model: 1-Parameter
Model abbr.: lm.1
Bechmark-Response (BMR): 10

BMCL: NA
BMC: NA
BMCU: NA

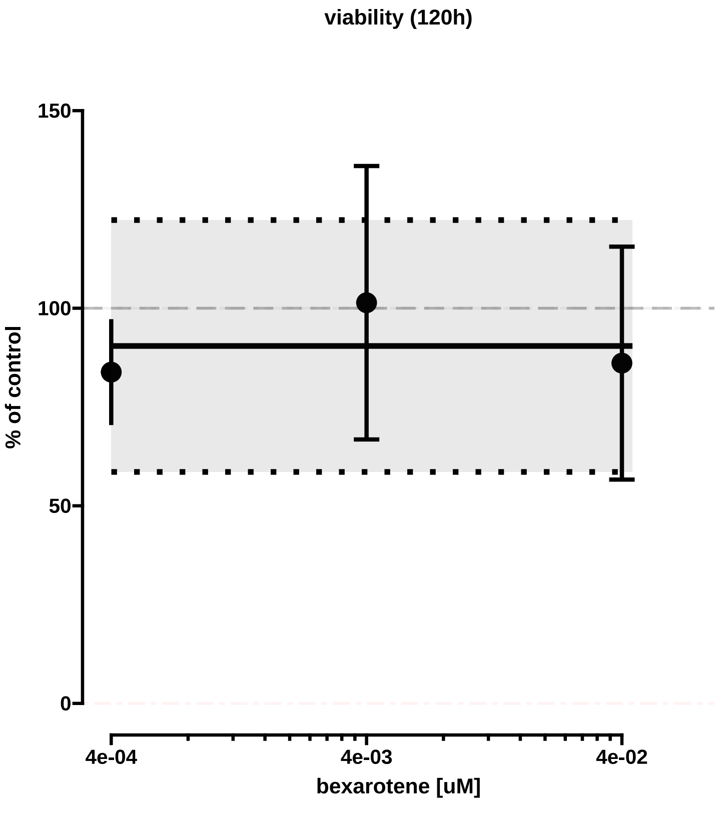

Model: 1-Parameter
Model abbr.: lm.1
Bechmark-Response (BMR): 20

BMCL: NA
BMC: NA
BMCU: NA

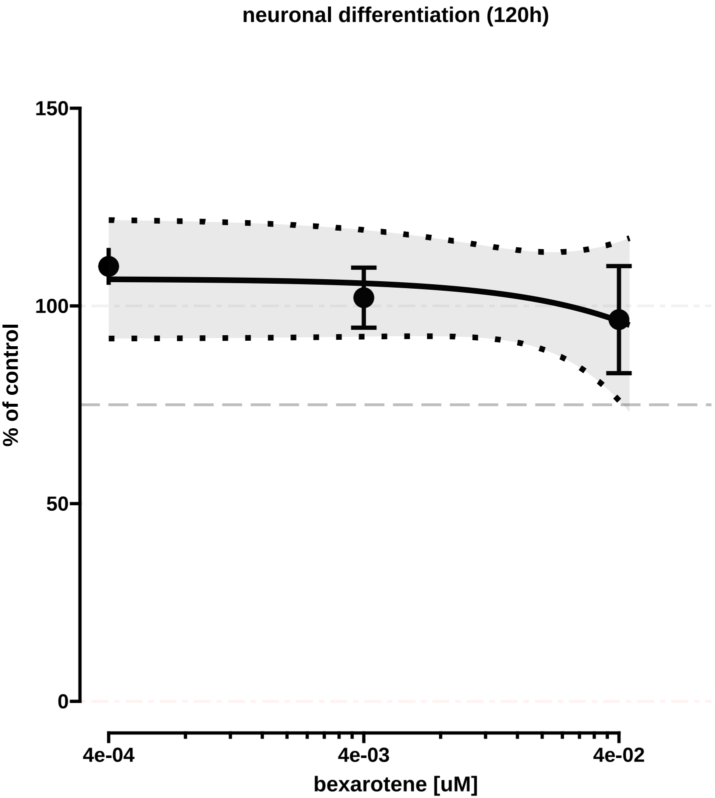

Model: Exponential decay with lower limit at 0
Model abbr.: EXD.2()
Bechmark-Response (BMR): 25

BMCL: NA
BMC: NA
BMCU: NA

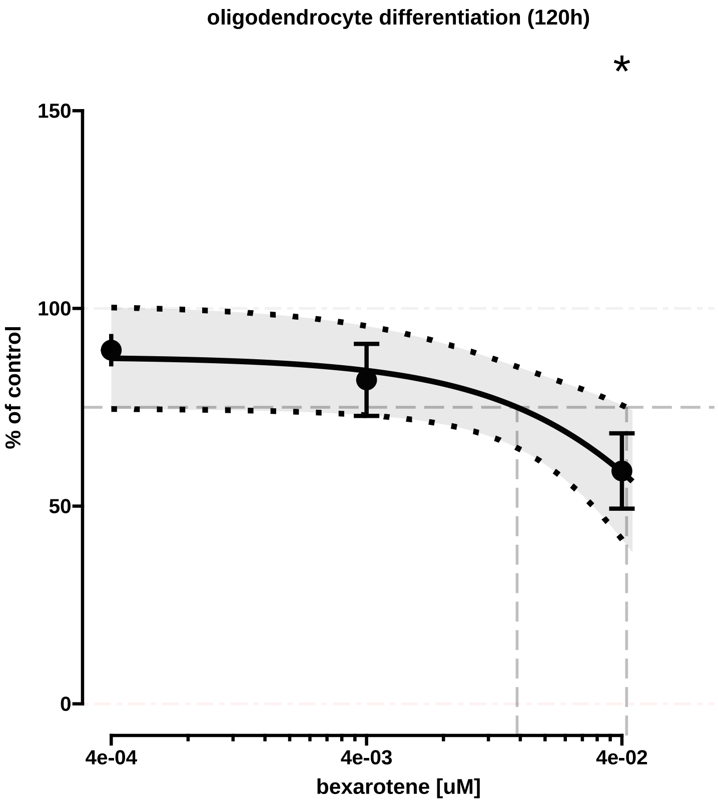

Model: Exponential decay with lower limit at 0
Model abbr.: EXD.2()
Bechmark-Response (BMR): 25

BMCL: NA
BMC: 0.016
BMCU: 0.042

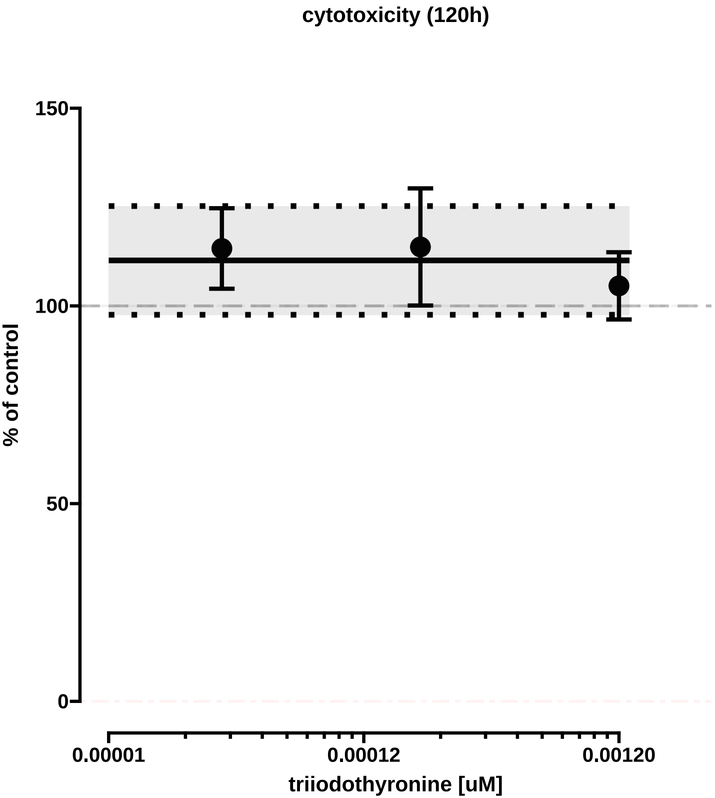

Model: 1-Parameter
Model abbr.: lm.1
Bechmark-Response (BMR): 10

BMCL: NA
BMC: NA
BMCU: NA

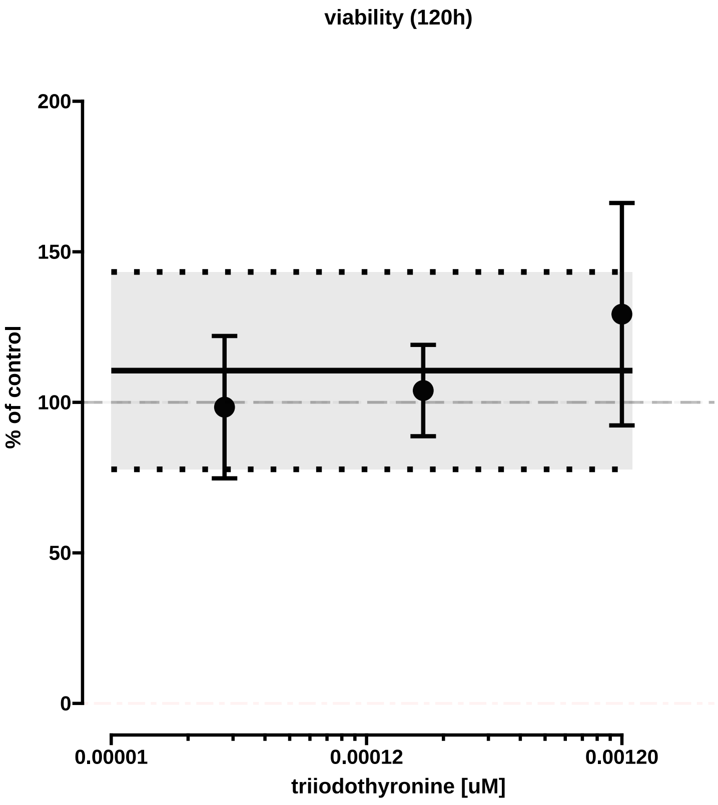

Model: 1-Parameter
Model abbr.: lm.1
Bechmark-Response (BMR): 20

BMCL: NA
BMC: NA
BMCU: NA

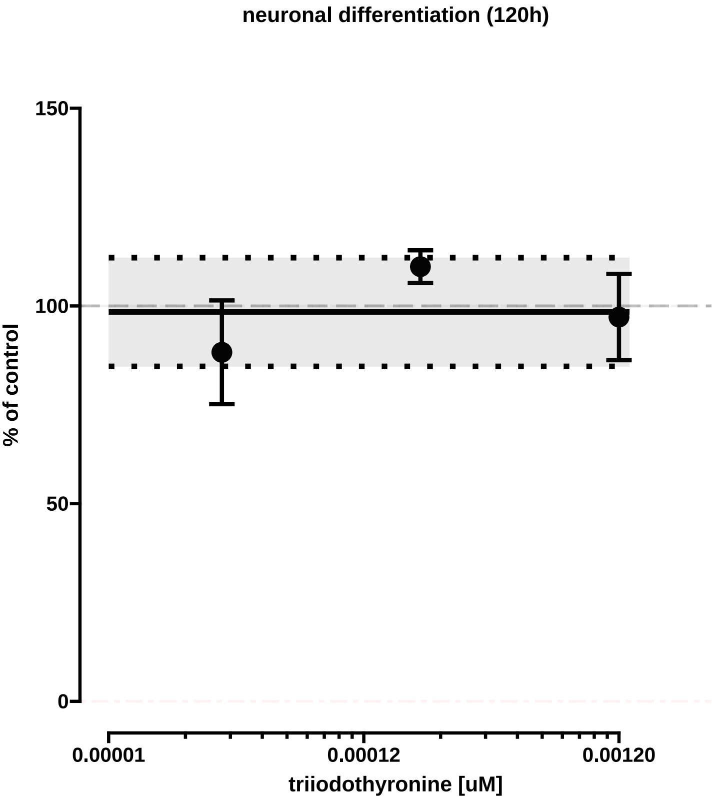

Model: 1-Parameter
Model abbr.: lm.1
Bechmark-Response (BMR): 25

BMCL: NA
BMC: NA
BMCU: NA

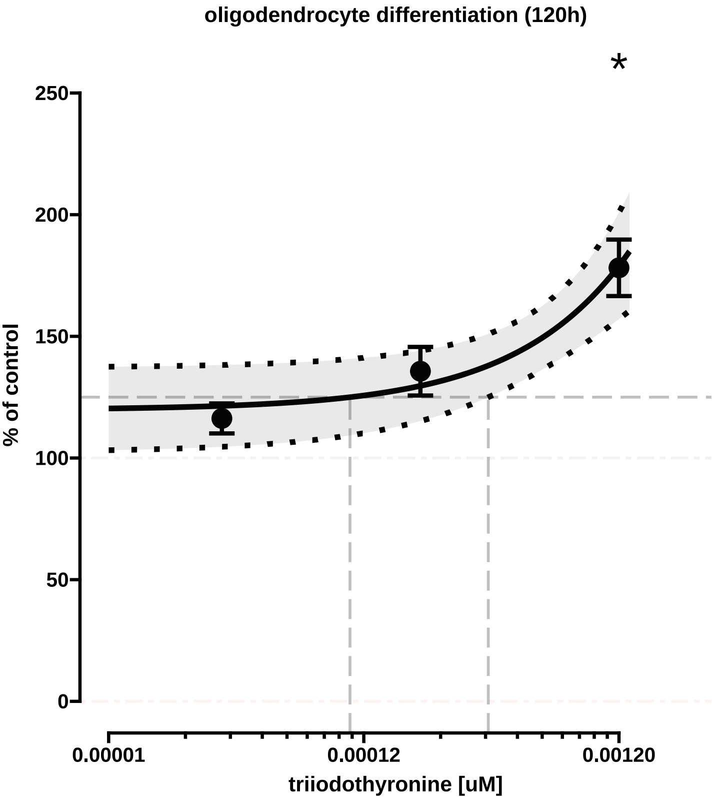

Model: Linear
Model abbr.: lm
Bechmark-Response (BMR): 25

BMCL: NA
BMC: 0
BMCU: 0
