# Supplementary material for: Nuclear hormone receptors control fundamental processes of human fetal neurodevelopment: Basis for endocrine disruption assessment
Source: Environ Int. Author manuscript; Available in PMC 2025 Jun 2. (PMC12127433; doi:10.1016/j.envint.2025.109400)
Supplement: 3 [file NIHMS2077722-supplement-3.docx]

**Supplementary Data 12 – Koch et al**

**Supplementary results + discussion**

## The AhR agonist benzo(*a*)pyrene elicits AhR-unspecific DNT effects in human NPC

The aryl hydrocarbon receptor (AhR) is a ligand-activated nuclear transcription factor that binds environmental pollutants such as chlorodibenzo-p-dioxin (TCDD) and benzo(*a*)pyrene (B(*a*)P), food contaminants, photoproducts from ultraviolet light irradiation and endogenous substances ultimately regulating the expression of several xenobiotic metabolizing enzymes including CYP1A1 (Abel and Haarmann-Stemmann, 2010; Fritsche et al., 2007) . While several in vivo rodent studies have reported neurodevelopmental AhR expression in neuronal and cortical progenitors (Collins et al., 2008; Kimura and Tohyama, 2017), we have previously observed species-specific AhR expression patterns, with fetal human NPCs being protected from AhR ligands due to the lack of AhR expression (Gassmann et al., 2010). However, the absence of AhR cannot be generalized to the developing human brain, as transcriptome data from the human NPC and primary fetal prefrontal cortex tissues analyzed in this study confirm AhR expression (Fig. 1A). However, the AhR does not appear to be functional, since exposure of differentiating human NPCs to the AhR agonist B(*a*)P differentially regulated only 3 genes (*CDKN1A*, *CPLX1* and *PDE2A*) that are not described AhR targets (Fig. 2). Phenotypically we observed altered neurite outgrowth, as measured by significantly reduced neurite area and total subneurite length (Supl. Fig 4A+C), as well as reduced percentage of oligodendrocytes within the migration area (Supl. Fig. 4E). However, none of these endophenotypes was antagonized by co-exposure to the AhR antagonist 3’-methoxy-4’-nitroflavone (MNF) (Supl. Fig. 4B, D and F). These observations and the lack of AhR-specific gene regulation suggest that B(*a*)P exerts AhR-independent effects in differentiating human NPCs.

## PPARα and γ regulate KNDPs in a non-antagonizable manner

In the adult rodent brain, PPAR alpha has been reported to be involved in synaptic plasticity as well as spatial learning and memory. However, its role in human brain development, and thus the effects of PPAR disruption by EDCs, remain poorly understood. Overactivation of PPARα in differentiating human NPCs with 576 µM GW7647 differentially regulated 325 genes, but in contrast to previous studies, PPARα overactivation suppressed the expression of several genes involved in cholesterol biosynthesis and fatty acid synthesis, suggesting depletion of cholesterol stores and disruption of lipid turnover, similar to LXR activation (Fig. 4). Consistent with this, we identified impaired oligodendrogenesis as the only significant phenotype of GW7647-exposed human NPCs (Supl. Fig. 3A). However, this effect could not be reversed by co-exposure to the PPARα antagonist GW6471, suggesting crosstalk with other hormone receptors (Sup. Fig. 3C). Similar to the LXR, inhibiting PPARα basal activity by GW6471 exposure reduced oligodendrogenesis (Supl. Fig. 3B). Our hypothesis of impaired oligodendrogenesis due to disturbed cholesterol metabolism is supported by the downregulation of genes in GO-terms involved in lipid and steroid metabolism as well as cholesterol biosynthetic processes (*DHCR24, DHCR7, FASN, HMGCS1, HSD17B14, LDLR, LSS, MSMO1, PNPLA3, PTGDS, RARRES3, SCD, SREBF1* and *TM7SF2*) upon GW7647 exposure of human NPCs (Sup. Fig. 3D and Supl. Data 15). PPARγ is poorly expressed in differentiating human NPCs and is also the least expressed subtype in the fetal prefrontal cortex (Fig. 2A). Treatment with the specific agonist 321 nM rosiglitazone differentially regulated five known PPARγ targets (*ANGPTL4*, *ECH1*, *EPHX1*, *SRRT* and *WWTR1*) (Fig. 4). Phenotypically, we observed a concentration-dependent increase in differentiating neurons, that could not be antagonized by the PPARγ antagonist GW9662 (Supl. Fig. 3E+G). However, opposite effects on neurogenesis were observed when PPARγ basal activity was inhibited (Supl. Fig. 3F), suggesting that PPARγ is involved, at least indirectly, in the regulation of neurogenesis via a receptor crosstalk with presumably the RXR. GO-term analysis only yielded few significantly regulated GO-terms (Supl. Fig. 3H and Supl. Data 15). Further phenotypic effects were observed upon PPARγ inhibition, but mainly at BMCs above 1 µM (Fig. 2C). In conclusion, PPARs α and γ have minor effects in human NPCs, presumably mediated by receptor cross-talk with other NRs. Nevertheless, our data clearly highlight the impact of EDCs that interfere with PPAR transcriptional activities.

## The progesterone receptor exerts mediocre effects on KNDPs

The *de novo* synthesis of progesterone in the brain from cholesterol is catalyzed by 3beta-hydroxysteroid dehydrogenase (3beta-HSD), which is expressed particularly neurons and glial cells (Schumacher et al., 2004). 3beta-HSD activity has previously been found in brain tissue of second trimester human fetuses, but expression was very low compared to other fetal organs and placental tissue (Milewich et al., 1991). Human NPCs do not express 3beta-HSD indicating that progesterone *de novo* synthesis can be neglected in our cell model and that the PR receptor is generally inactive in human NPCs. PR activation with 1.5 µM progesterone caused mostly gene inhibition (63%) also of reported target genes (*APOD*, *CITED1* and *SOX10*), which is in contrast to previous studies describing activation of expression (Fig. 4). As the only endpoint affected by PR activation, oligodendrogenesis was impaired (BMC_25_ = 1.22 µM, Supl. Fig. 4G) at concentrations in the range of fetal cord blood progesterone levels (176.4 - 2818.0 ng/ml, equivalent to 0.56 – 8.96 µM, table 2). However, the effect could not be antagonized by the PR antagonist ulipristal acetate (UA), suggesting PR-unspecific effects, consistent with the low PR expression in human NPCs (Supl. Fig. 4H and Fig. 2A). Although PR inhibition with UA negatively affected radial glia migration, subneurite length, oligodendrocyte differentiation and nuclei count, all effects were observed at micromolar concentrations. GO-term analysis revealed upregulation of genes involved in cell cycle and proliferation processes, while lipid/cholesterol metabolic processes and negative regulators of the Wnt pathway were suppressed (Supl. Fig. 4I and Supl. Data 15), indicating mechanistic similarities to LXR activation. As both, the NPC proliferation and differentiation media, contain 20 nM progesterone (Bottenstein, 1985; Brewer et al., 1993), the PR exhibits basal activity in human NPCs. This is supported by the multiple KNDPs affected by exposure to the PR antagonist ulipristal acetate at relatively high concentrations (2.6 - 8.2 µM, Fig. 2C). The synergistic effects of co-exposure to progesterone and ulipristal acetate on oligodendrogenesis, however, hint at agonistic effects at high (10 µM) ulipristal acetate concentrations (Supl. Fig. 4H).
