# Supplementary material for: Nuclear hormone receptors control fundamental processes of human fetal neurodevelopment: Basis for endocrine disruption assessment
Source: Environ Int. Author manuscript; Available in PMC 2025 Jun 2. (PMC12127433; doi:10.1016/j.envint.2025.109400)
Supplement: 8 [file NIHMS2077722-supplement-8.pdf]

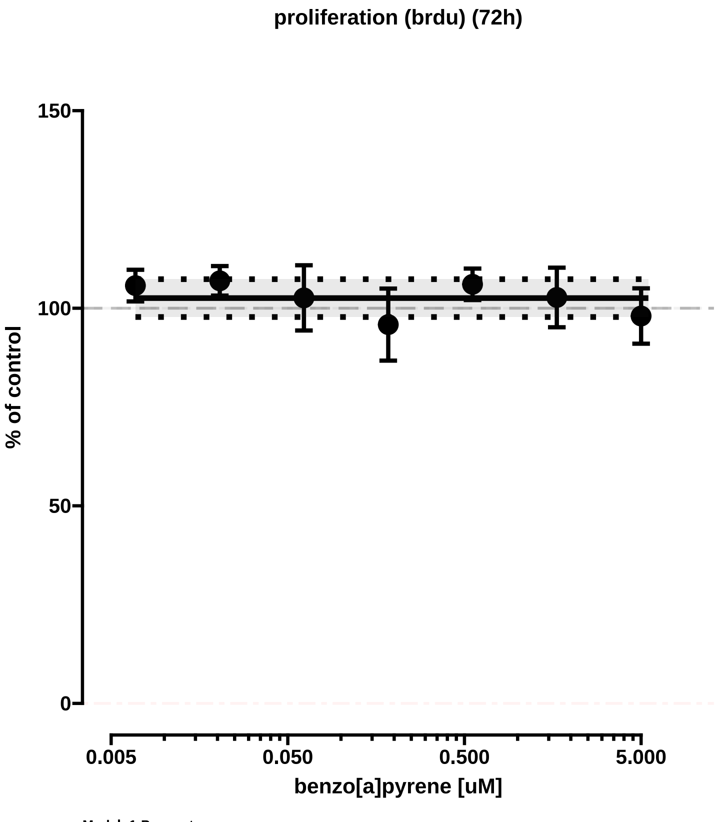

Model: 1-Parameter
Model abbr.: lm.1
Bechmark-Response (BMR): 20

BMCL: NA
BMC: NA
BMCU: NA

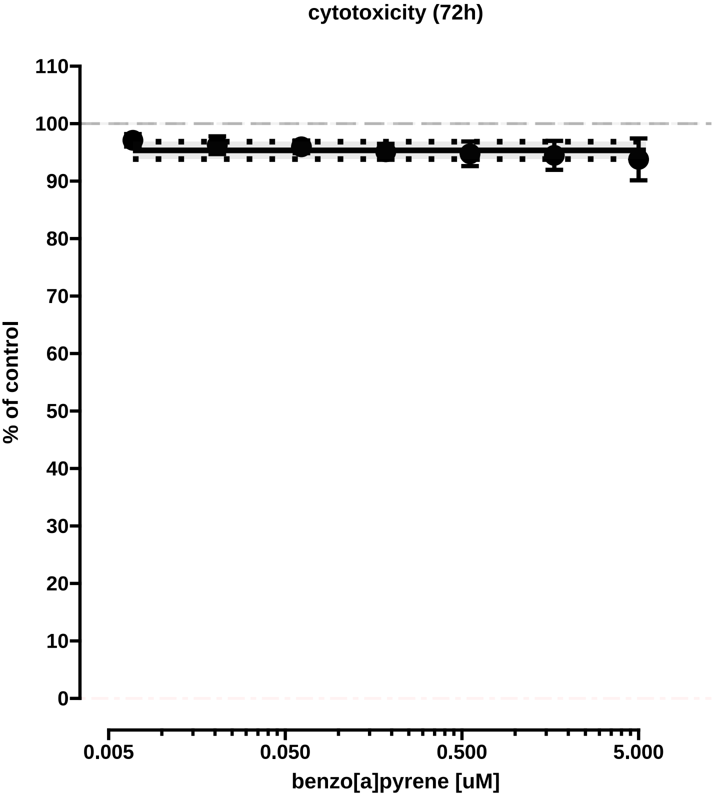

Model: 1-Parameter
Model abbr.: lm.1
Bechmark-Response (BMR): 10

BMCL: NA
BMC: NA
BMCU: NA

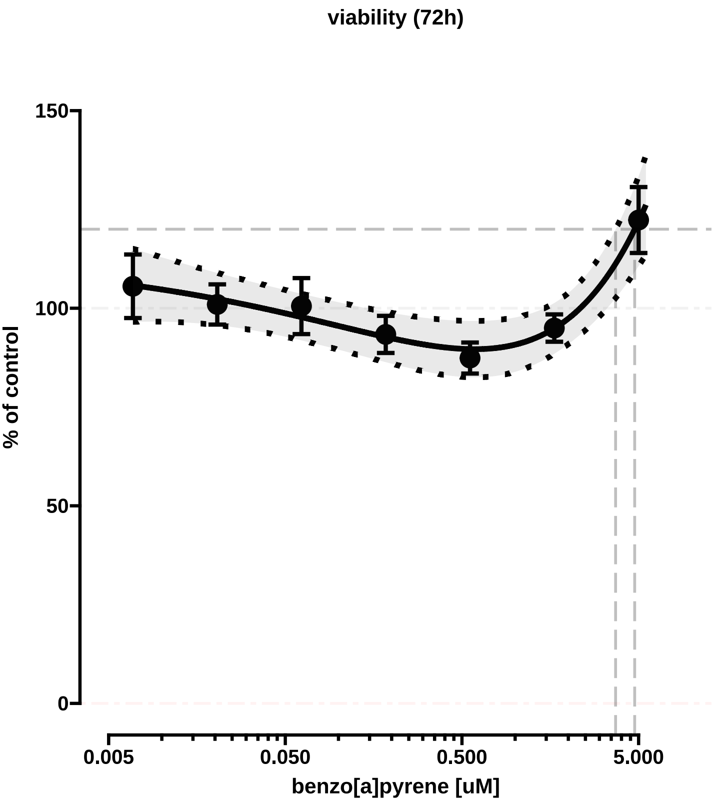

Model: Brain-Cousens (hormesis) with lower limit fixed at 0
Model abbr.: BC.4()
Bechmark-Response (BMR): 20

BMCL: 3.704
BMC: 4.751
BMCU: NA

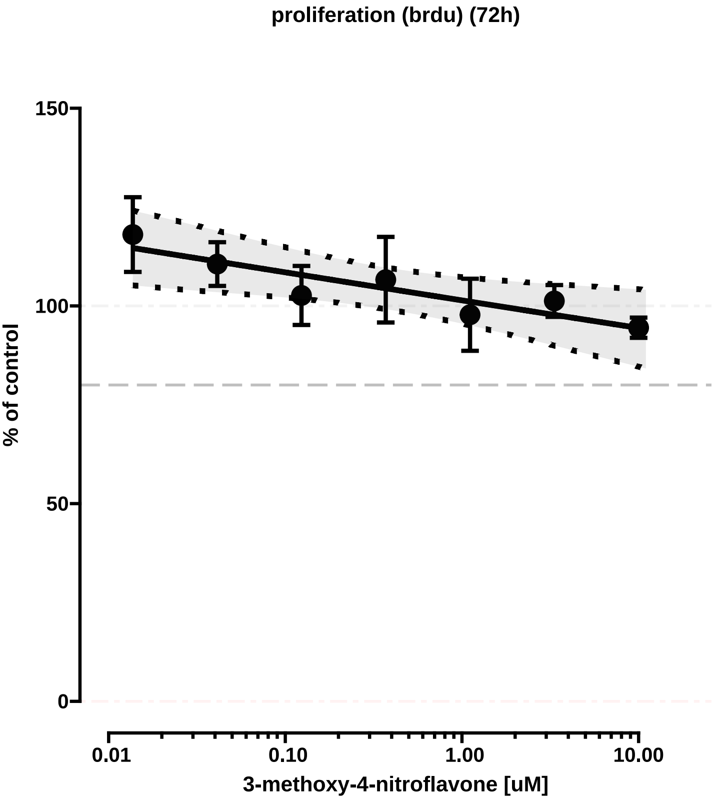

Model: Weibull (type 2) with lower limit at 0
Model abbr.: W2.3()
Bechmark-Response (BMR): 20

BMCL: NA
BMC: NA
BMCU: NA

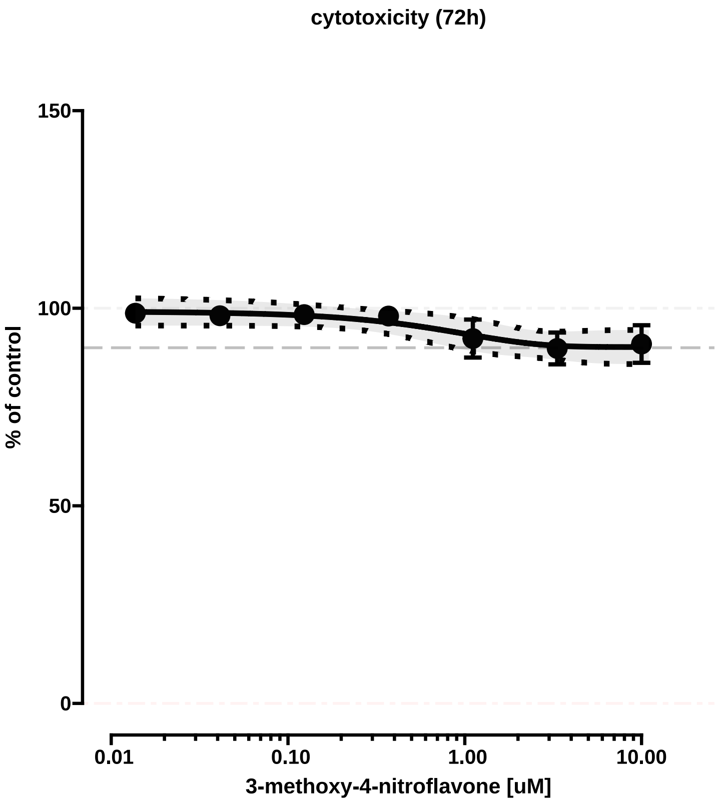

Model: Shifted exponential decay
Model abbr.: EXD.3()
Bechmark-Response (BMR): 10

BMCL: NA
BMC: NA
BMCU: NA

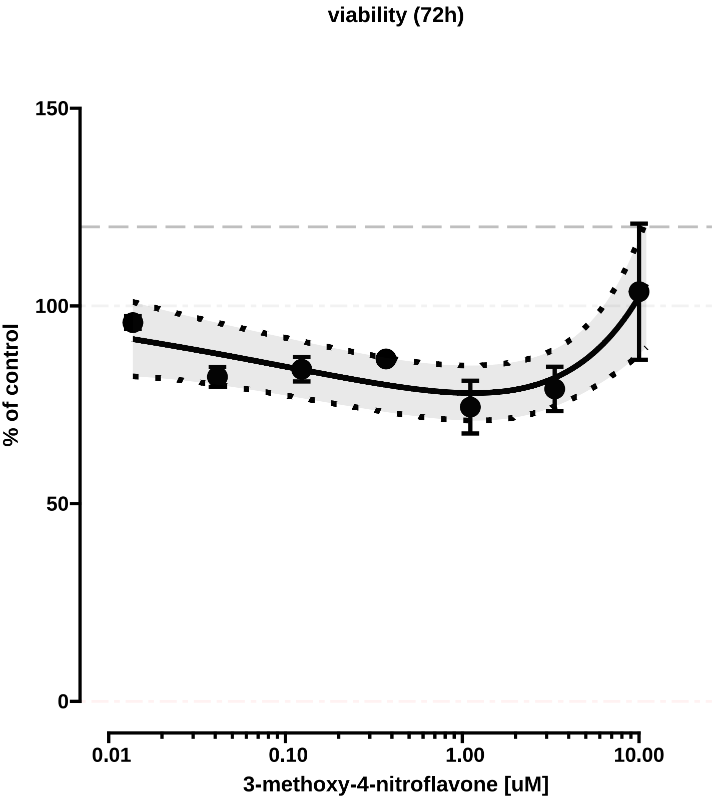

Model: Brain-Cousens (hormesis) with lower limit fixed at 0
Model abbr.: BC.4()
Bechmark-Response (BMR): 20

BMCL: NA
BMC: NA
BMCU: NA

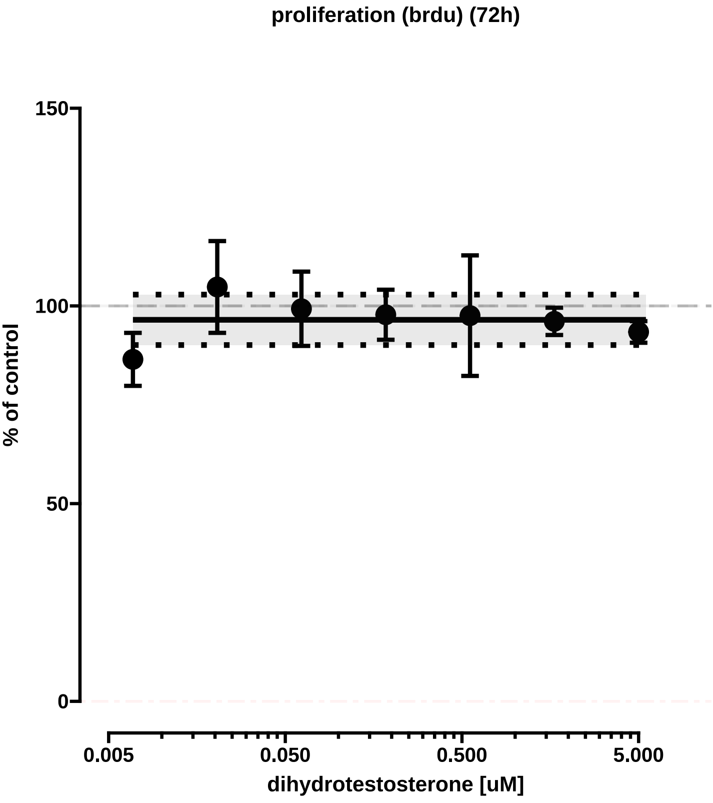

Model: 1-Parameter
Model abbr.: lm.1
Bechmark-Response (BMR): 20

BMCL: NA
BMC: NA
BMCU: NA

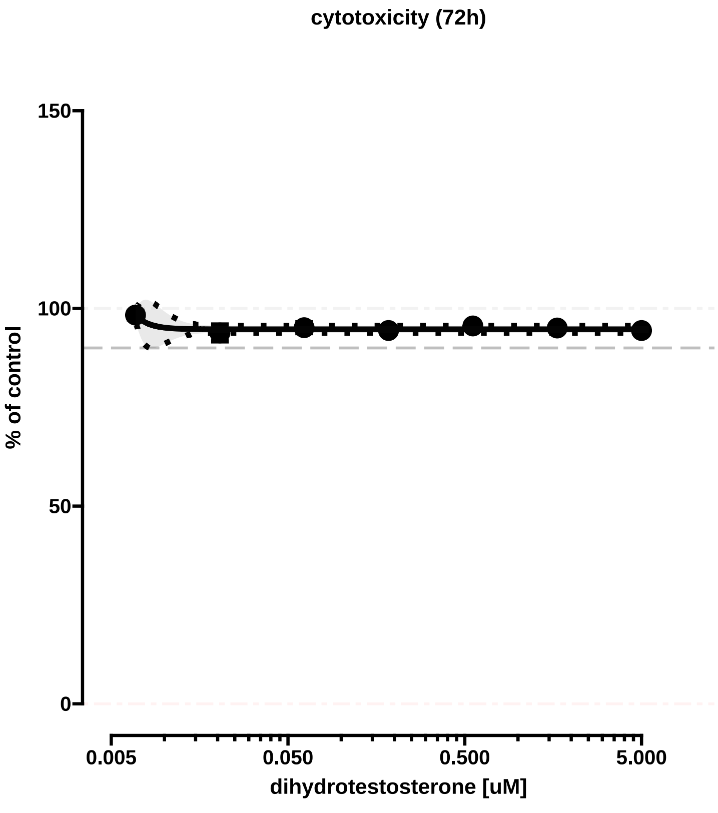

Model: Log-logistic (log(ED50) as parameter)
Model abbr.: LL2.4()
Bechmark-Response (BMR): 10

BMCL: NA
BMC: NA
BMCU: NA

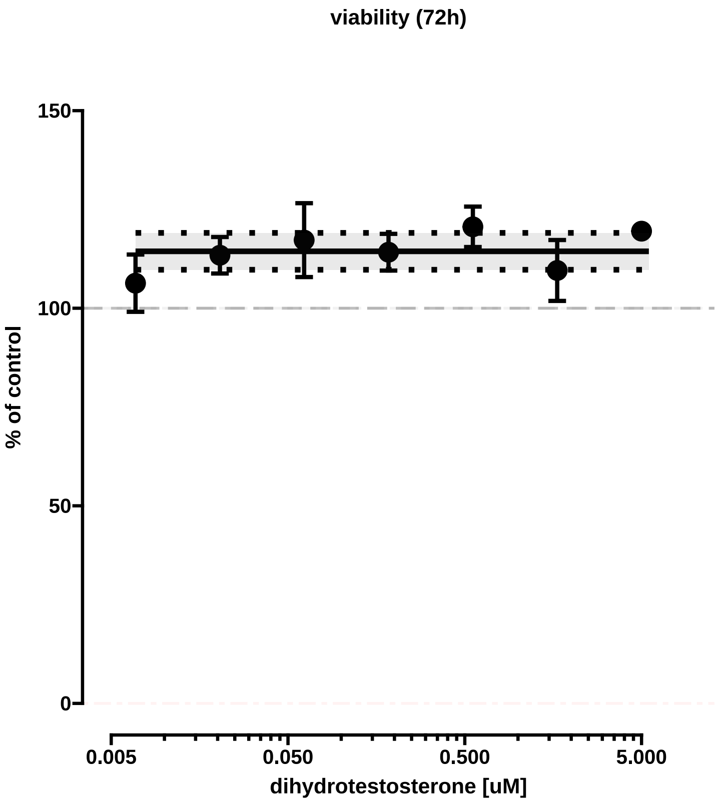

Model: 1-Parameter
Model abbr.: lm.1
Bechmark-Response (BMR): 20

BMCL: NA
BMC: NA
BMCU: NA

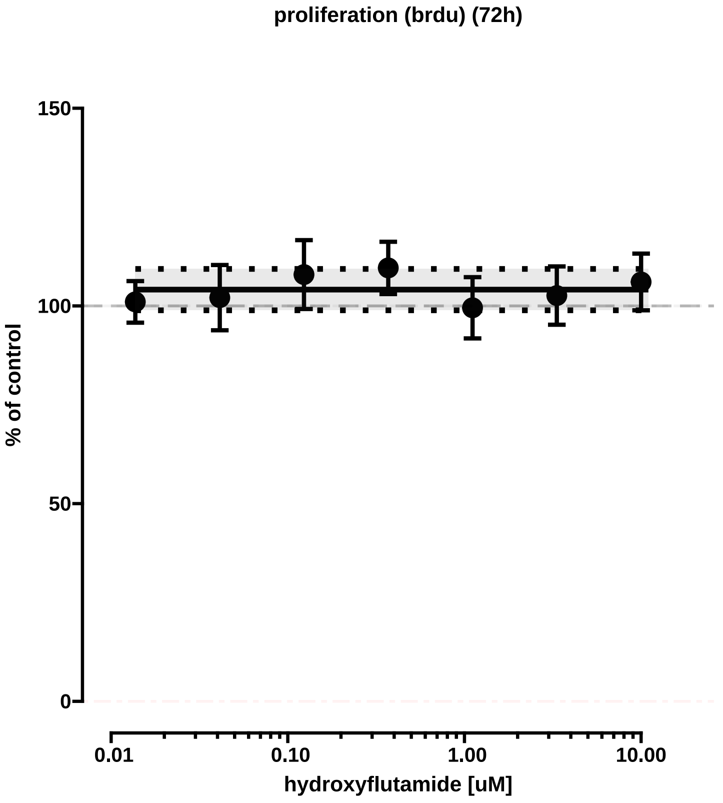

Model: 1-Parameter
Model abbr.: lm.1
Bechmark-Response (BMR): 20

BMCL: NA
BMC: NA
BMCU: NA

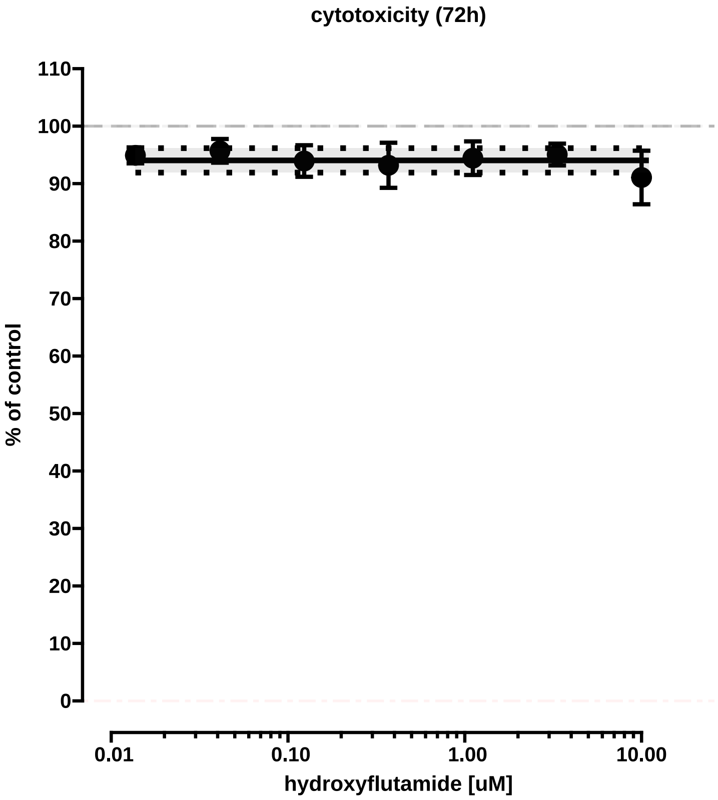

Model: 1-Parameter
Model abbr.: lm.1
Bechmark-Response (BMR): 10

BMCL: NA
BMC: NA
BMCU: NA

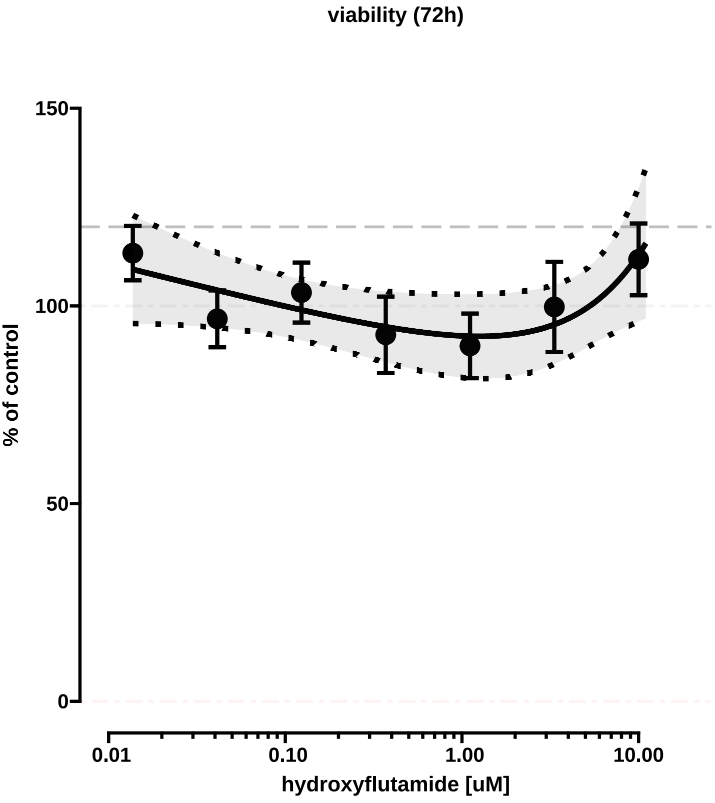

Model: Brain-Cousens (hormesis) with lower limit fixed at 0
Model abbr.: BC.4()
Bechmark-Response (BMR): 20

BMCL: NA
BMC: NA
BMCU: NA

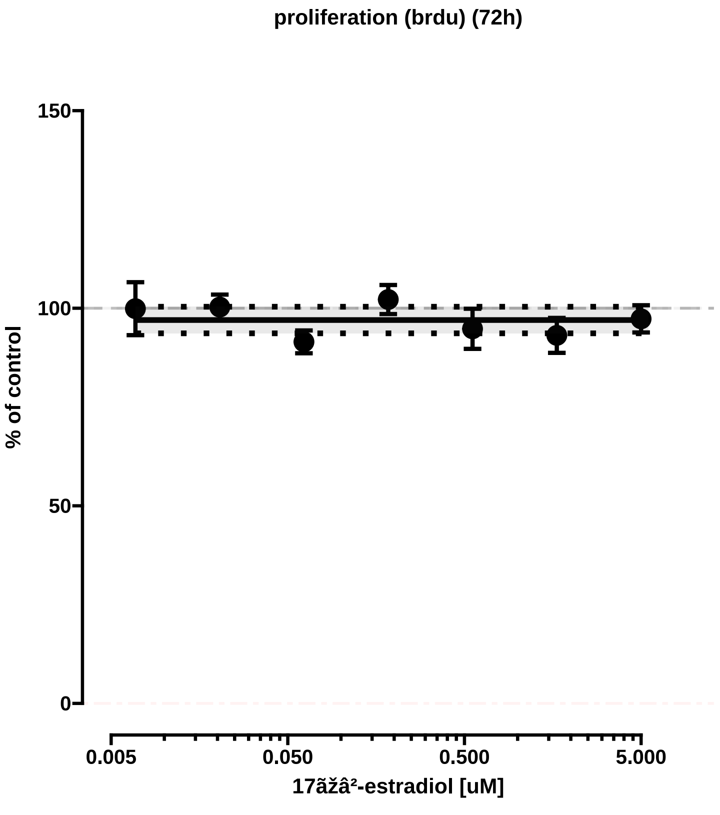

Model: 1-Parameter
Model abbr.: lm.1
Bechmark-Response (BMR): 20

BMCL: NA
BMC: NA
BMCU: NA

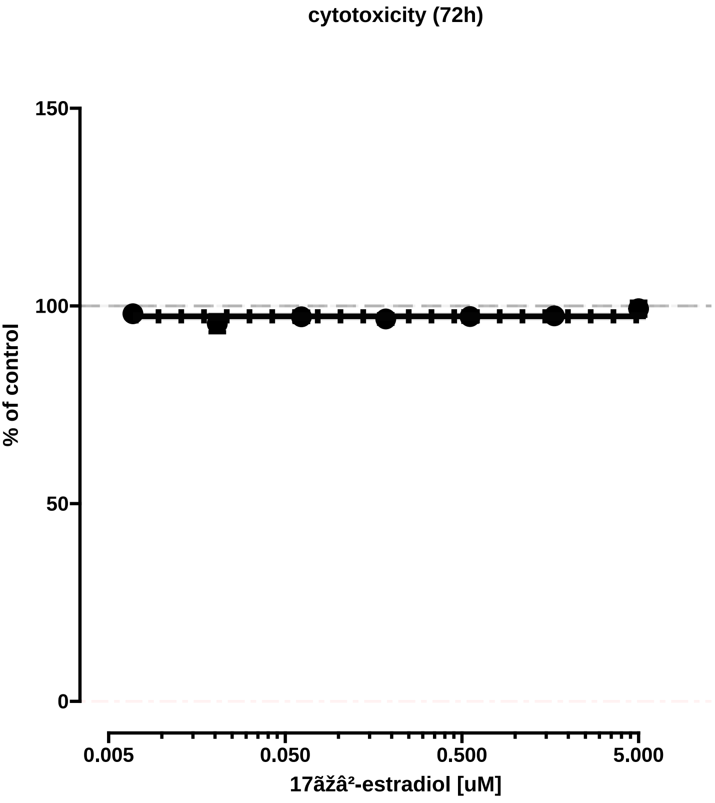

Model: 1-Parameter
Model abbr.: lm.1
Bechmark-Response (BMR): 10

BMCL: NA
BMC: NA
BMCU: NA

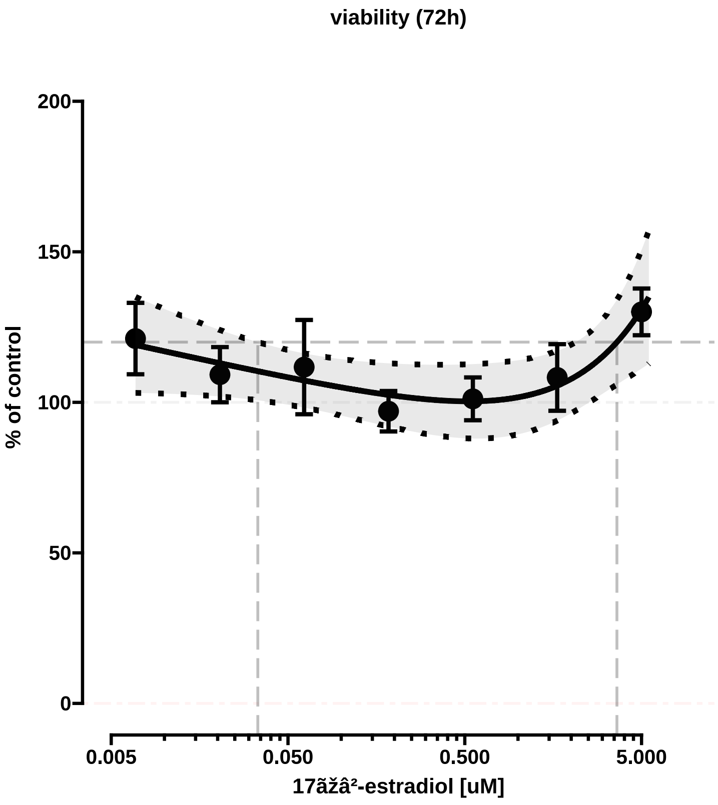

Model: Brain-Cousens (hormesis) with lower limit fixed at 0
Model abbr.: BC.4()
Bechmark-Response (BMR): 20

BMCL: 0.034
BMC: 3.628
BMCU: NA

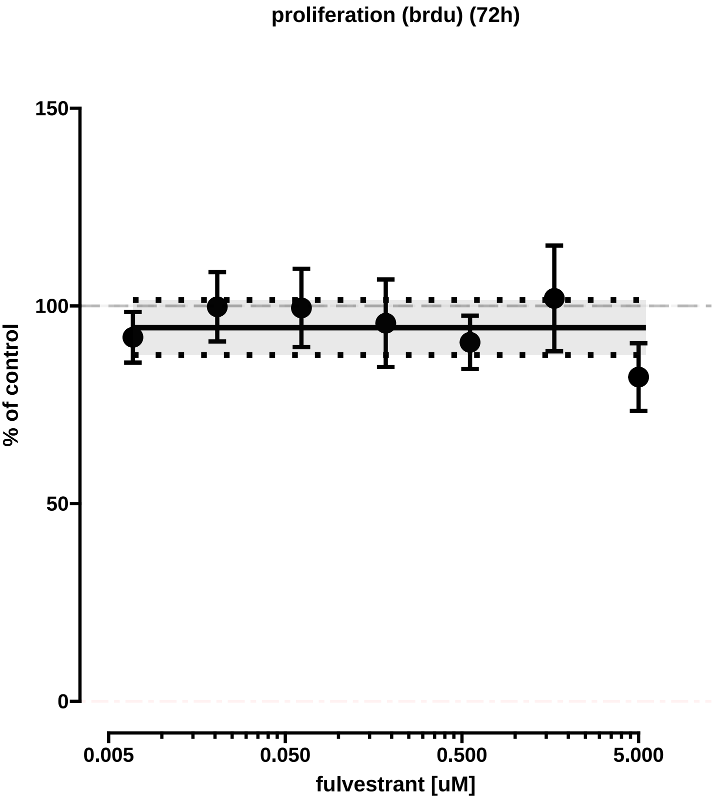

Model: 1-Parameter
Model abbr.: lm.1
Bechmark-Response (BMR): 20

BMCL: NA
BMC: NA
BMCU: NA

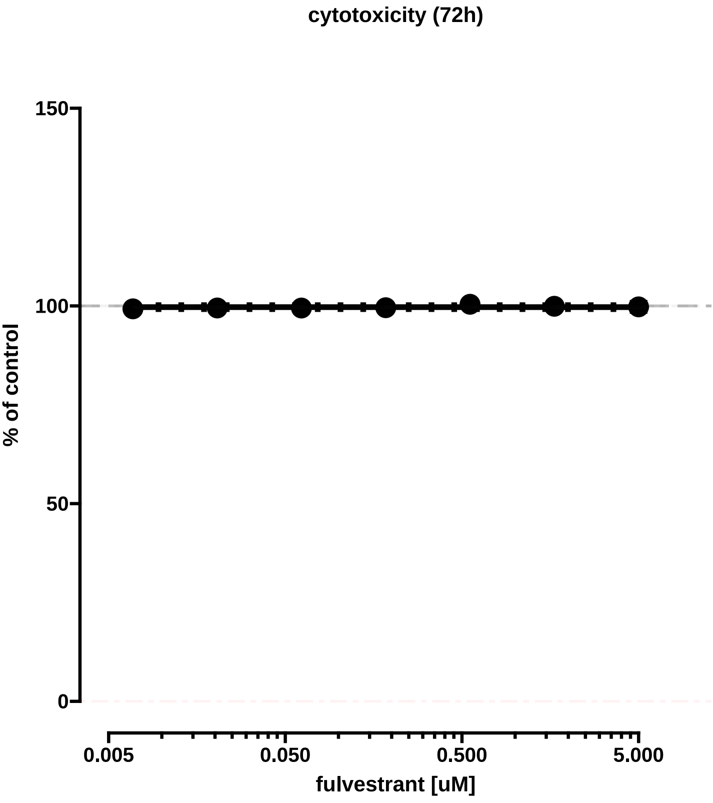

Model: 1-Parameter
Model abbr.: lm.1
Bechmark-Response (BMR): 10

BMCL: NA
BMC: NA
BMCU: NA

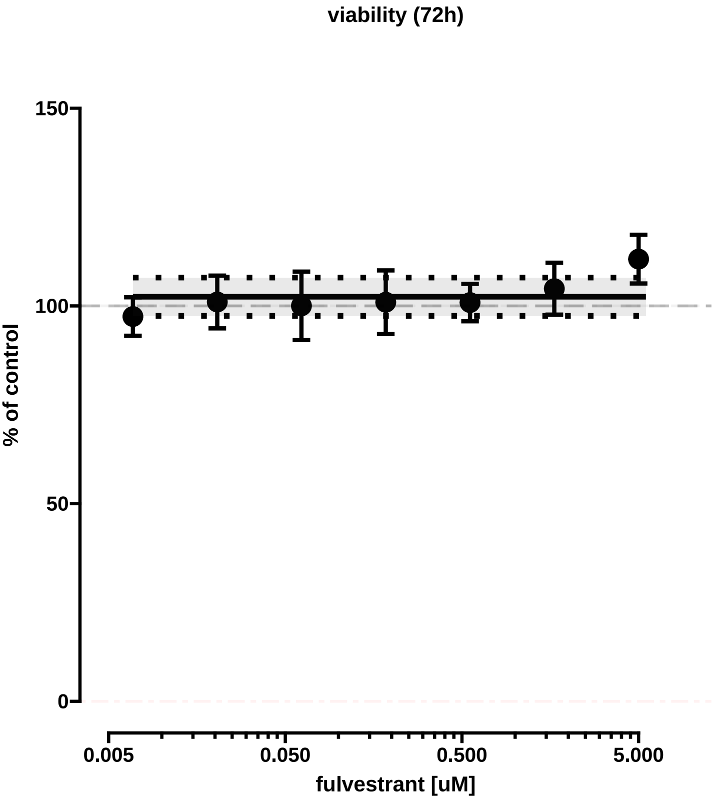

Model: 1-Parameter
Model abbr.: lm.1
Bechmark-Response (BMR): 20

BMCL: NA
BMC: NA
BMCU: NA

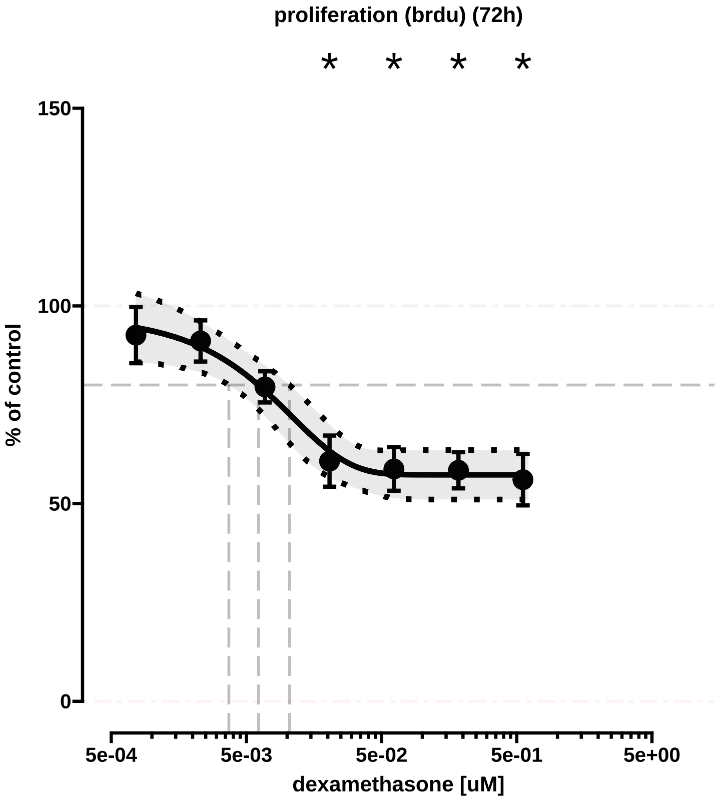

Model: Shifted exponential decay
Model abbr.: EXD.3()
Bechmark-Response (BMR): 20

BMCL: 0.004
BMC: 0.006
BMCU: 0.01

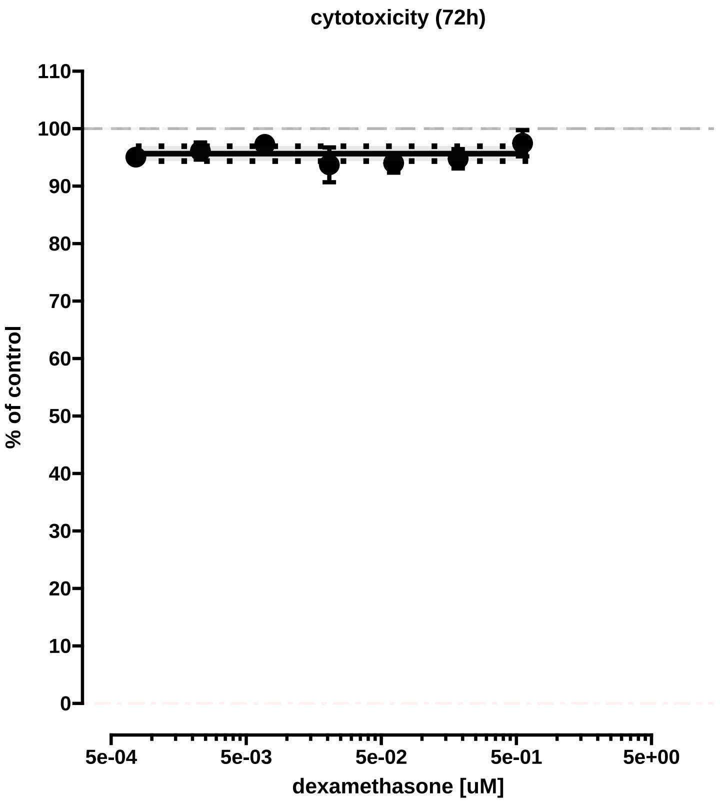

Model: 1-Parameter
Model abbr.: lm.1
Bechmark-Response (BMR): 10

BMCL: NA
BMC: NA
BMCU: NA

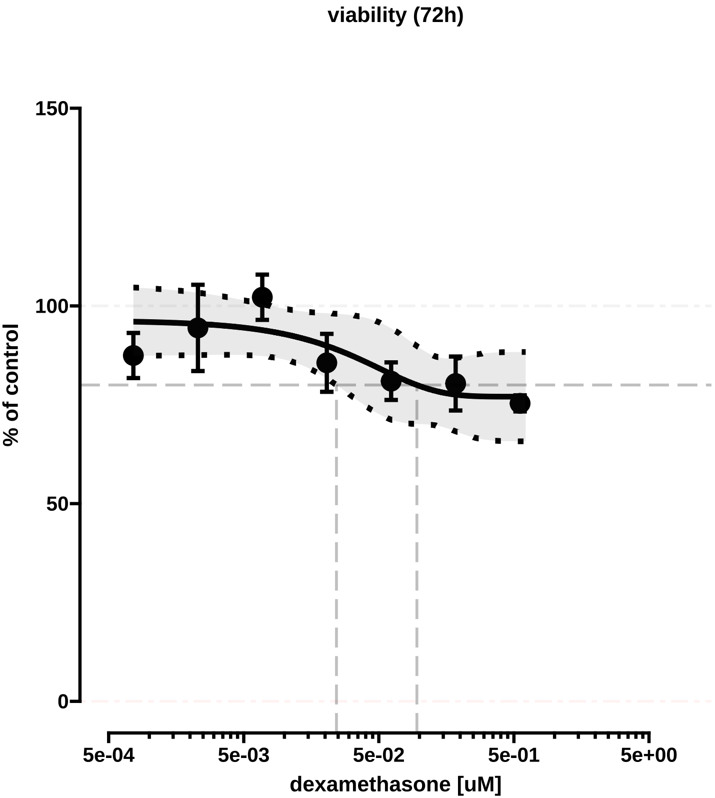

Model: Shifted exponential decay
Model abbr.: EXD.3()
Bechmark-Response (BMR): 20

BMCL: 0.024
BMC: 0.096
BMCU: NA

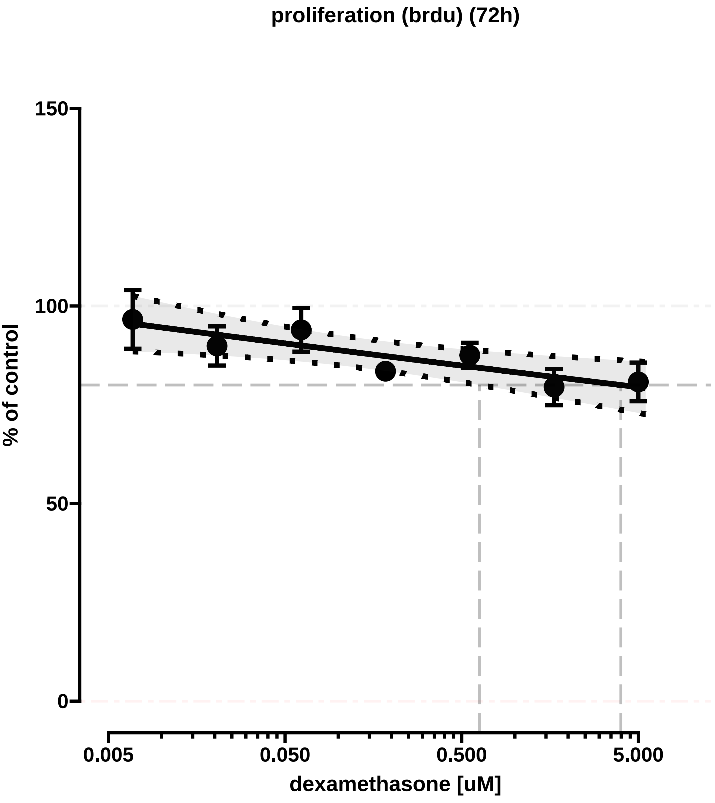

Model: Log-logistic (log(ED50) as parameter) with lower limit at 0
Model abbr.: LL2.3()
Bechmark-Response (BMR): 20

BMCL: 0.63
BMC: 3.979
BMCU: NA

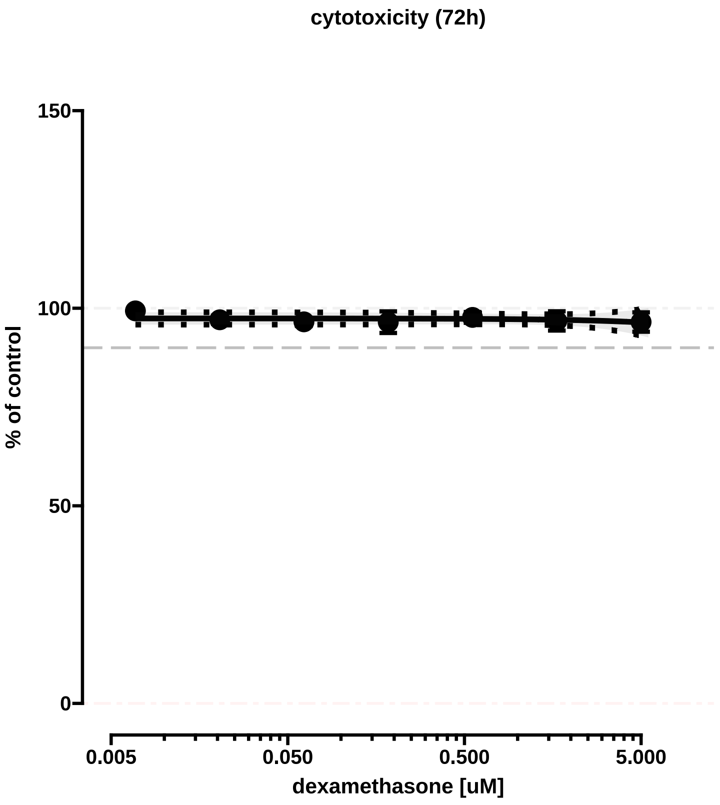

Model: Exponential decay with lower limit at 0
Model abbr.: EXD.2()
Bechmark-Response (BMR): 10

BMCL: NA
BMC: NA
BMCU: NA

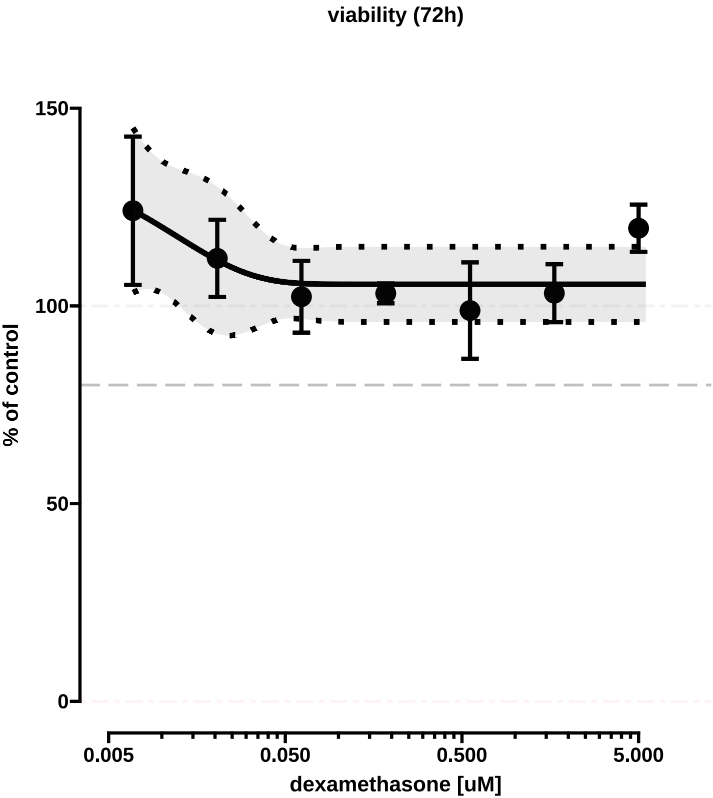

Model: Shifted exponential decay
Model abbr.: EXD.3()
Bechmark-Response (BMR): 20

BMCL: NA
BMC: NA
BMCU: NA

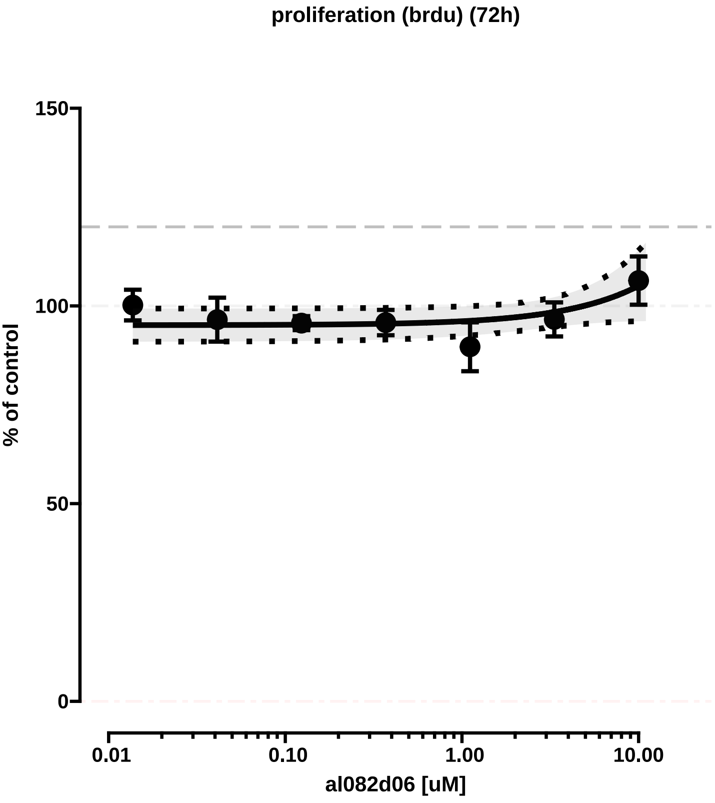

Model: Linear
Model abbr.: lm
Bechmark-Response (BMR): 20

BMCL: NA
BMC: NA
BMCU: NA

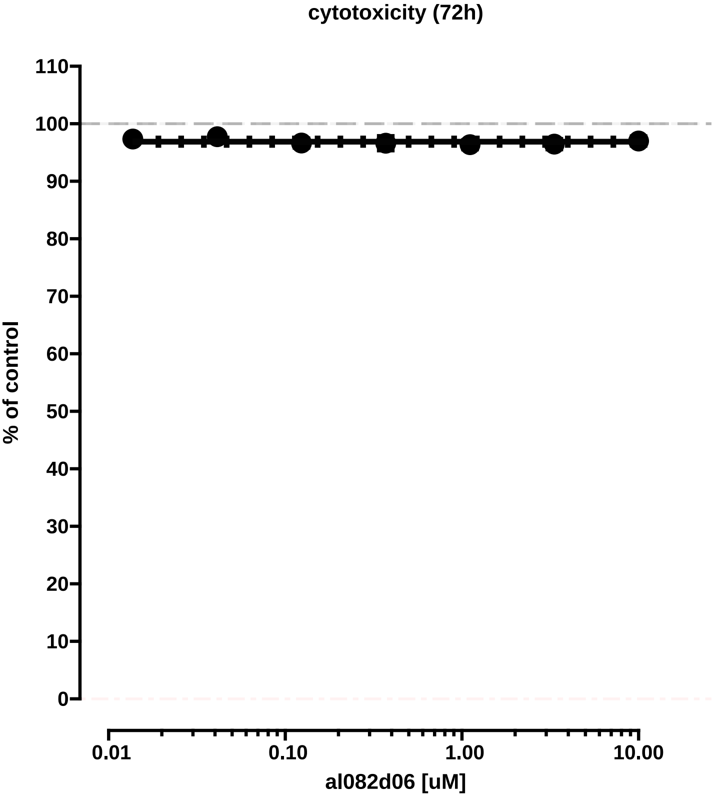

Model: 1-Parameter
Model abbr.: lm.1
Bechmark-Response (BMR): 10

BMCL: NA
BMC: NA
BMCU: NA

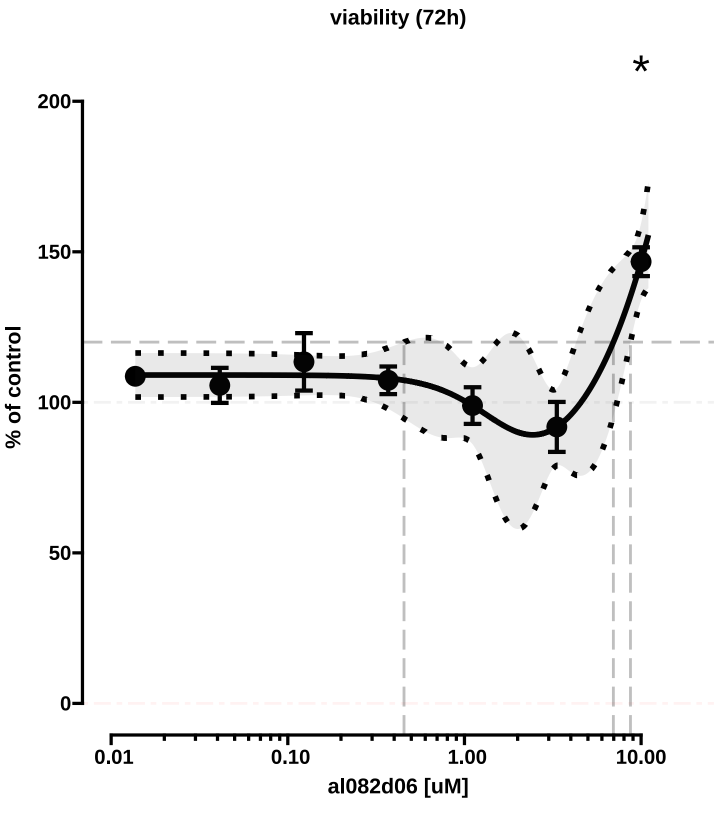

Model: Brain-Cousens (hormesis)
Model abbr.: BC.5()
Bechmark-Response (BMR): 20

BMCL: 0.455
BMC: 6.966
BMCU: 8.704

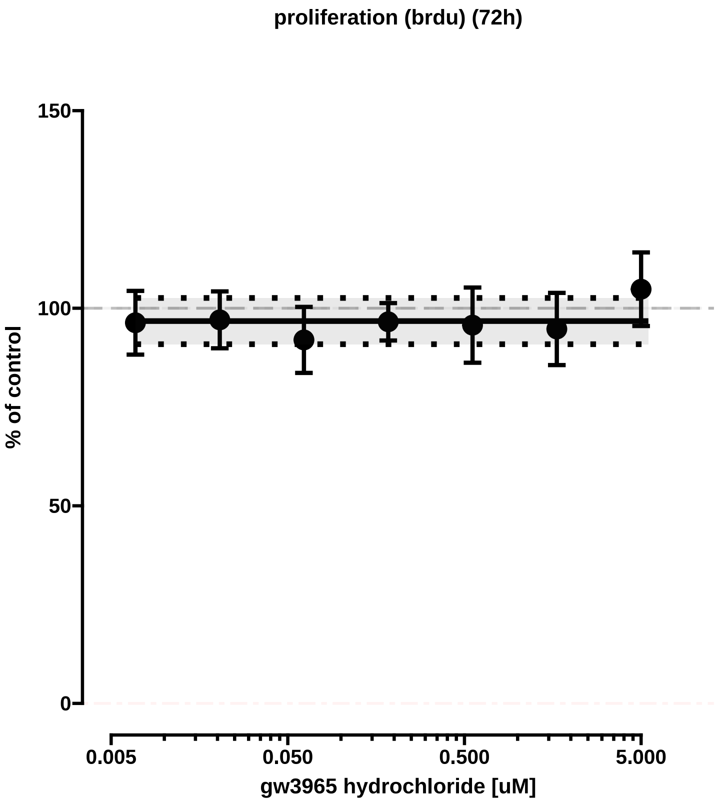

Model: 1-Parameter
Model abbr.: lm.1
Bechmark-Response (BMR): 20

BMCL: NA
BMC: NA
BMCU: NA

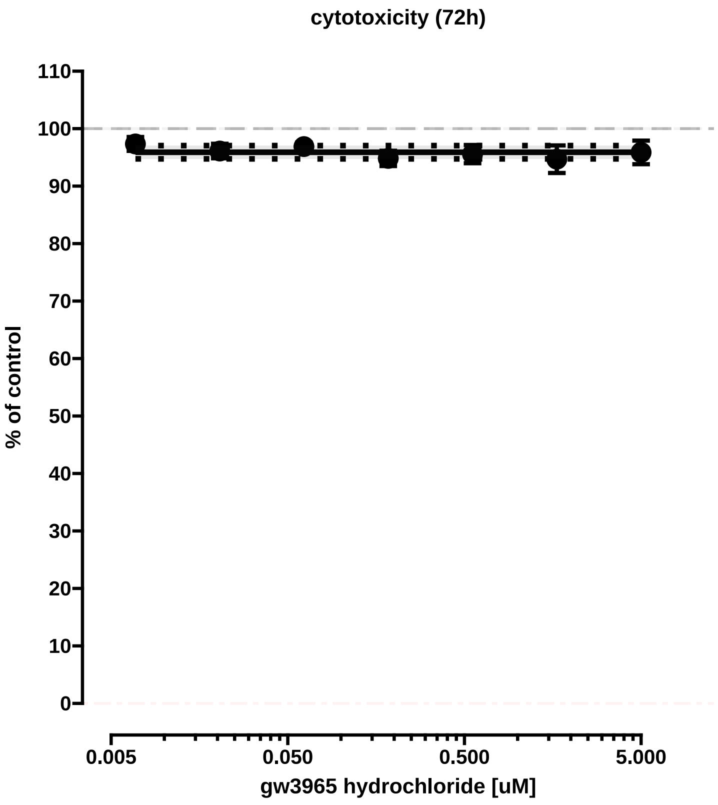

Model: 1-Parameter
Model abbr.: lm.1
Bechmark-Response (BMR): 10

BMCL: NA
BMC: NA
BMCU: NA

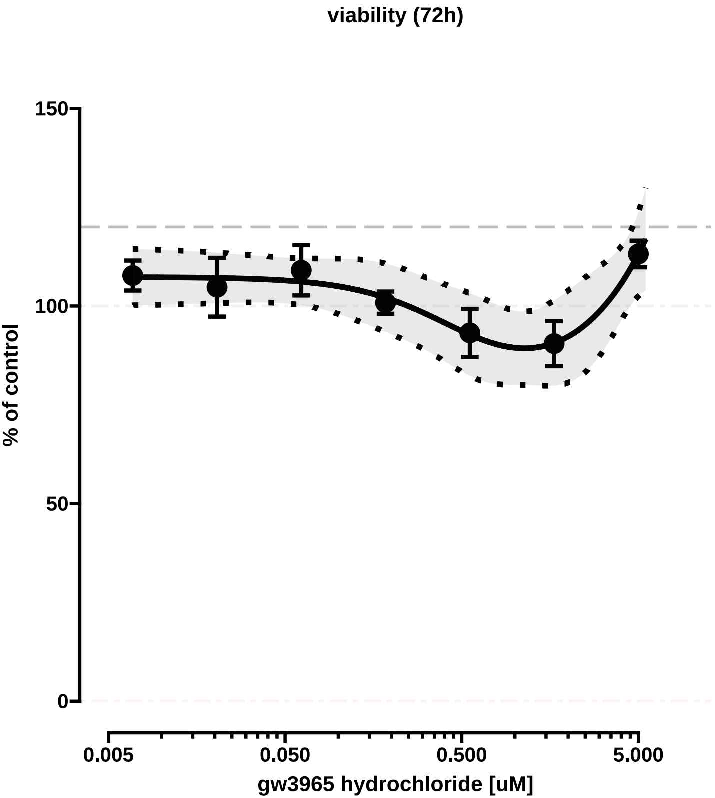

Model: Brain-Cousens (hormesis)
Model abbr.: BC.5()
Bechmark-Response (BMR): 20

BMCL: NA
BMC: NA
BMCU: NA

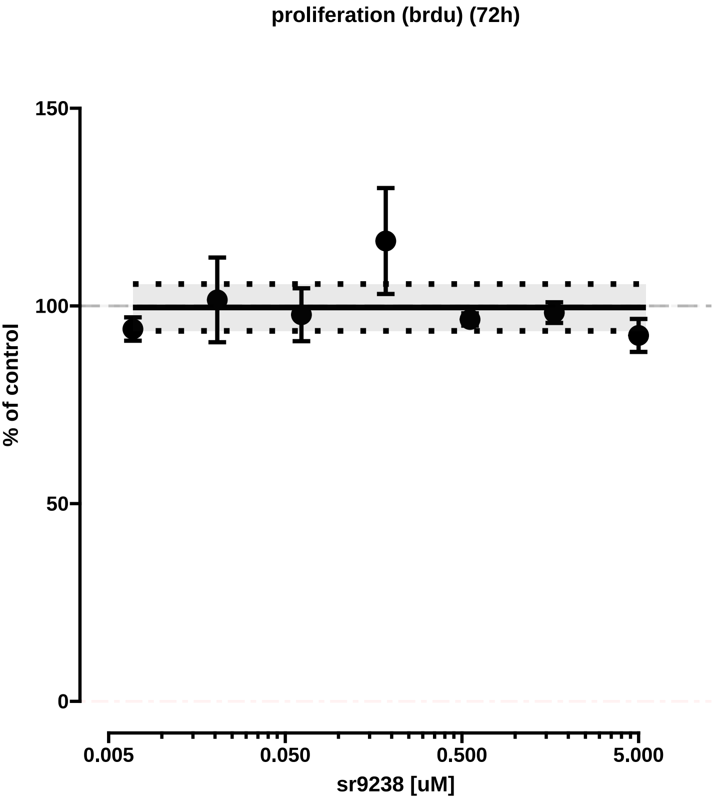

Model: 1-Parameter
Model abbr.: lm.1
Bechmark-Response (BMR): 20

BMCL: NA
BMC: NA
BMCU: NA

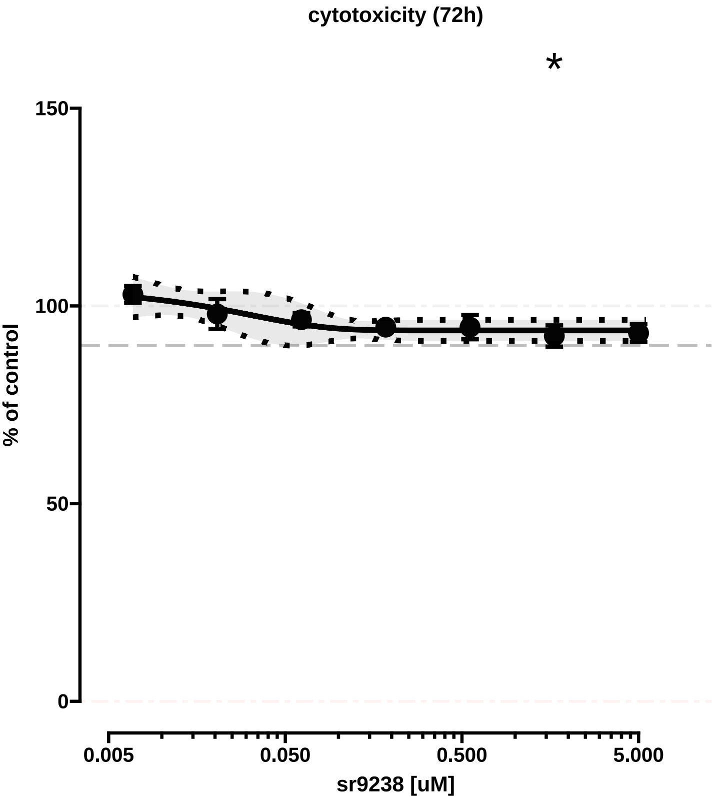

Model: Shifted exponential decay
Model abbr.: EXD.3()
Bechmark-Response (BMR): 10

BMCL: NA
BMC: NA
BMCU: NA

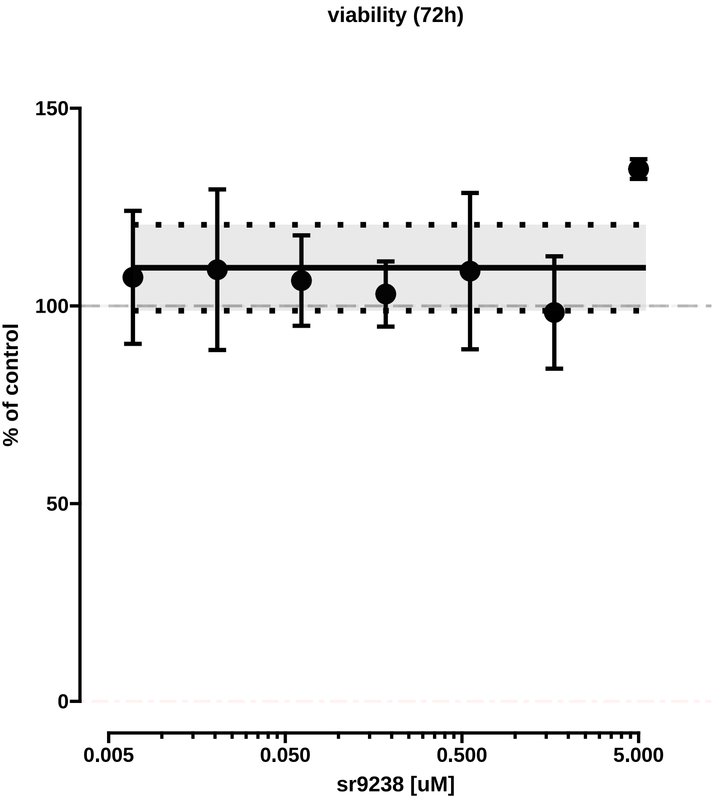

Model: 1-Parameter
Model abbr.: lm.1
Bechmark-Response (BMR): 20

BMCL: NA
BMC: NA
BMCU: NA

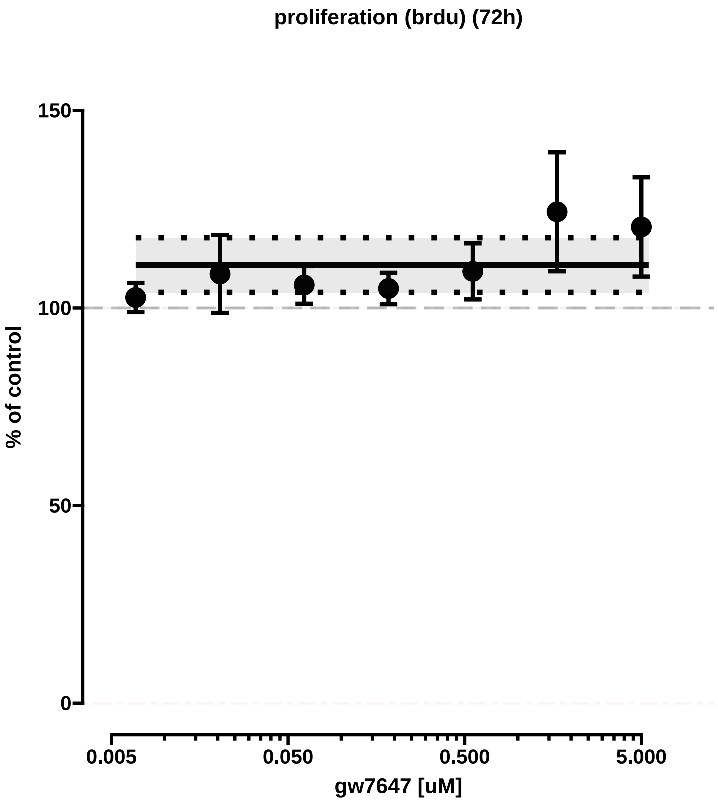

Model: 1-Parameter
Model abbr.: lm.1
Bechmark-Response (BMR): 20

BMCL: NA
BMC: NA
BMCU: NA

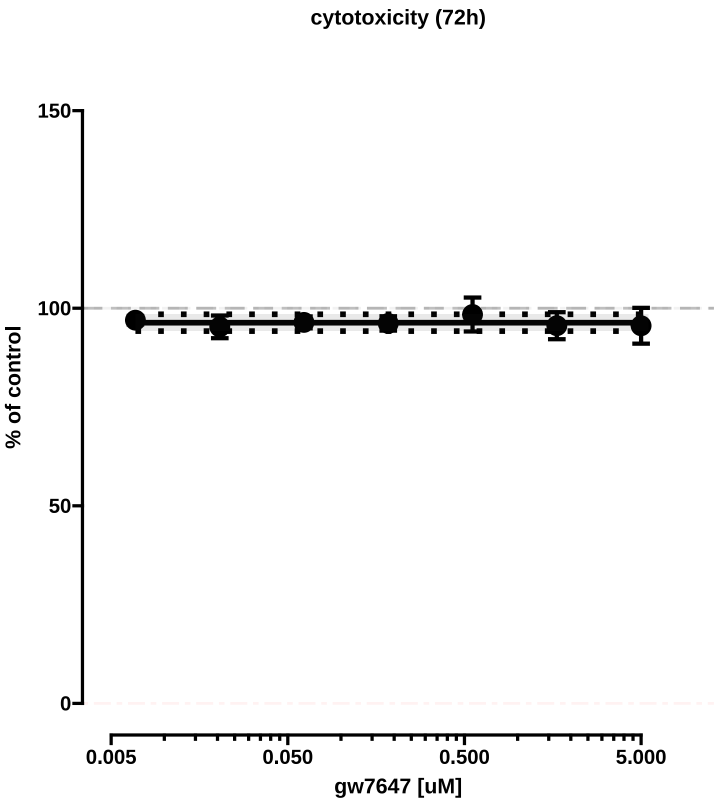

Model: 1-Parameter
Model abbr.: lm.1
Bechmark-Response (BMR): 10

BMCL: NA
BMC: NA
BMCU: NA

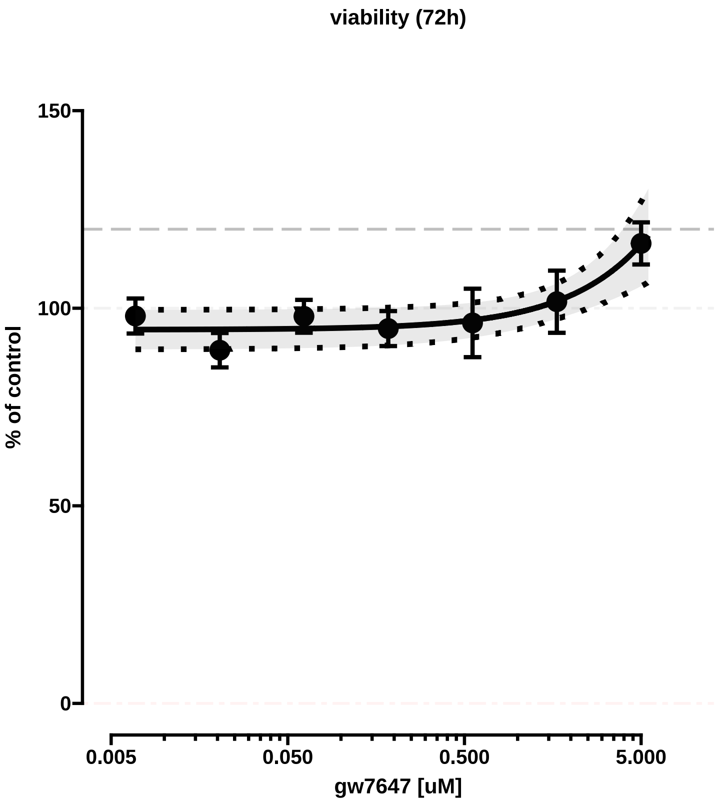

Model: Linear
Model abbr.: lm
Bechmark-Response (BMR): 20

BMCL: NA
BMC: NA
BMCU: NA

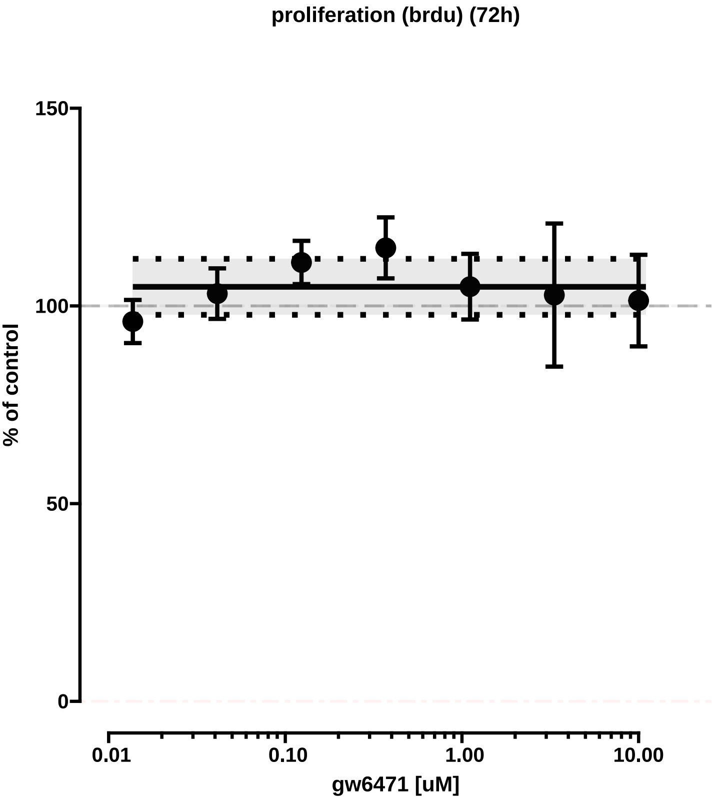

Model: 1-Parameter
Model abbr.: lm.1
Bechmark-Response (BMR): 20

BMCL: NA
BMC: NA
BMCU: NA

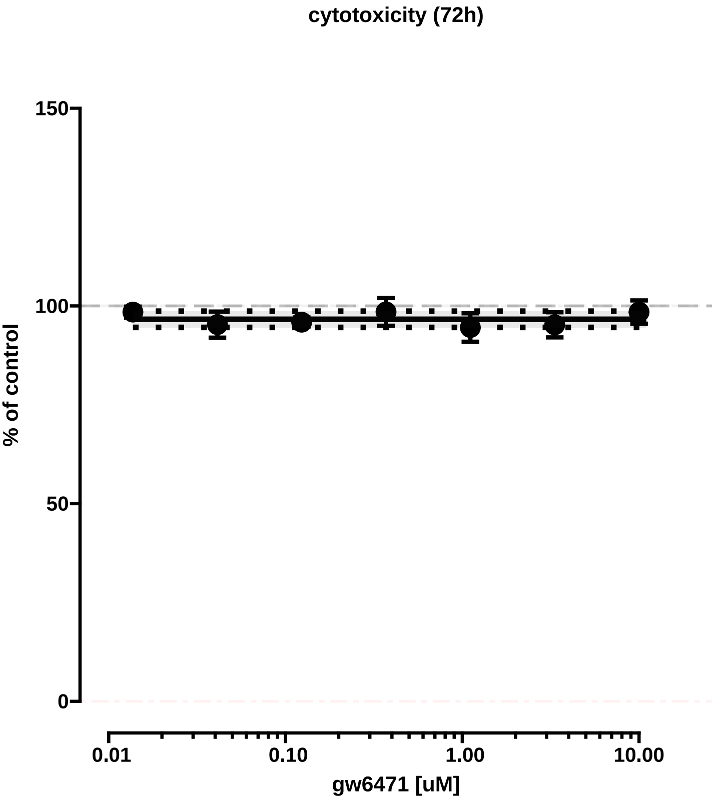

Model: 1-Parameter
Model abbr.: lm.1
Bechmark-Response (BMR): 10

BMCL: NA
BMC: NA
BMCU: NA

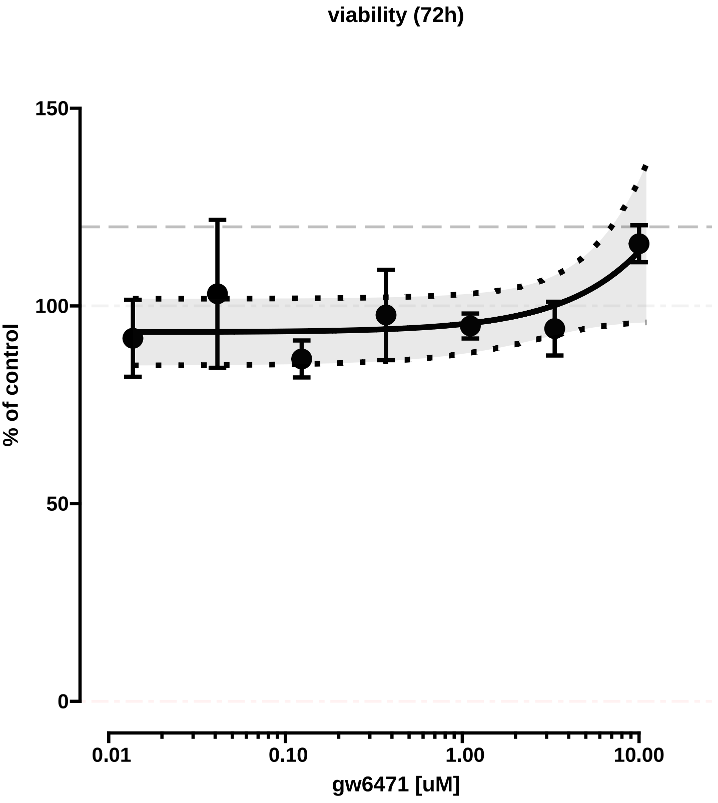

Model: Linear
Model abbr.: lm
Bechmark-Response (BMR): 20

BMCL: NA
BMC: NA
BMCU: NA

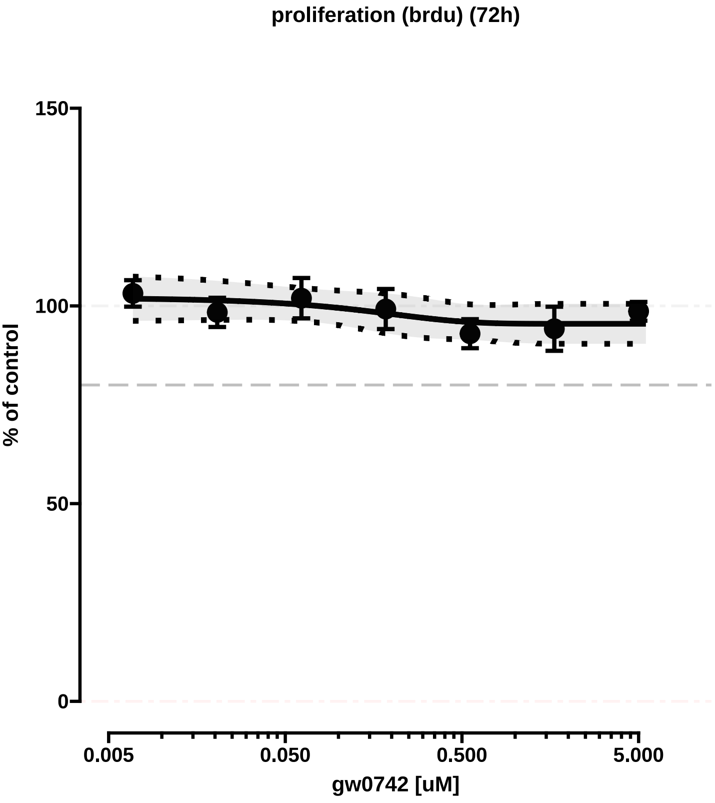

Model: Shifted exponential decay
Model abbr.: EXD.3()
Bechmark-Response (BMR): 20

BMCL: NA
BMC: NA
BMCU: NA

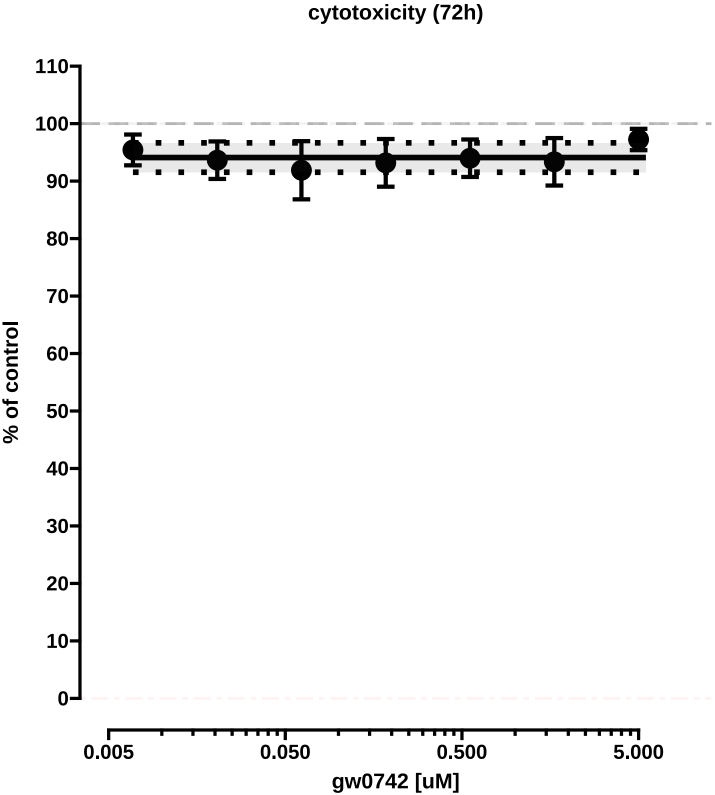

Model: 1-Parameter
Model abbr.: lm.1
Bechmark-Response (BMR): 10

BMCL: NA
BMC: NA
BMCU: NA

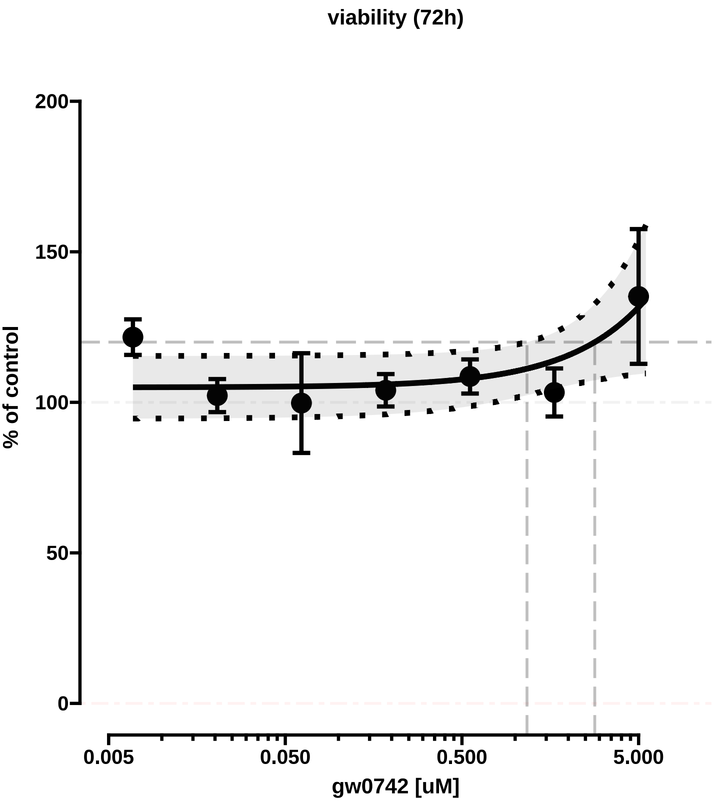

Model: Linear
Model abbr.: lm
Bechmark-Response (BMR): 20

BMCL: 1.167
BMC: 2.824
BMCU: NA

proliferation (brdu) (72h)

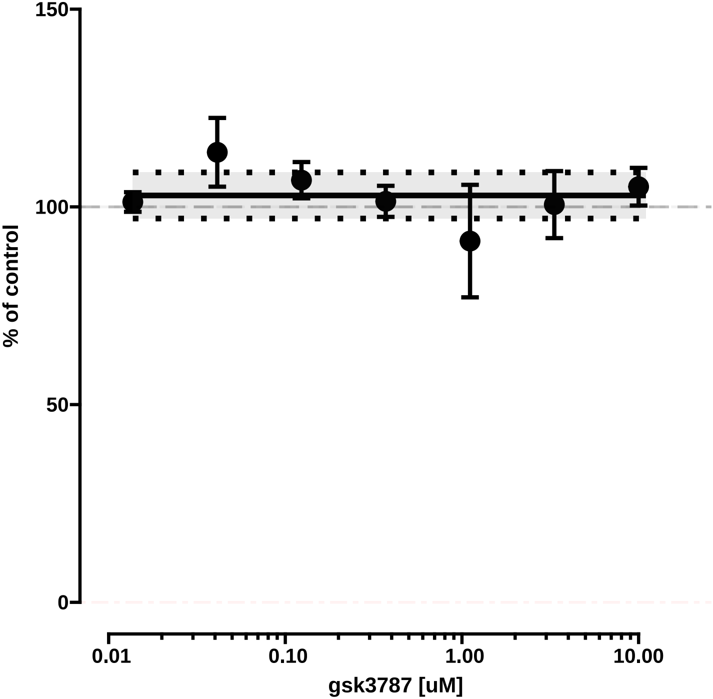

Model: 1-Parameter
Model abbr.: lm.1
Bechmark-Response (BMR): 20

BMCL: NA
BMC: NA
BMCU: NA

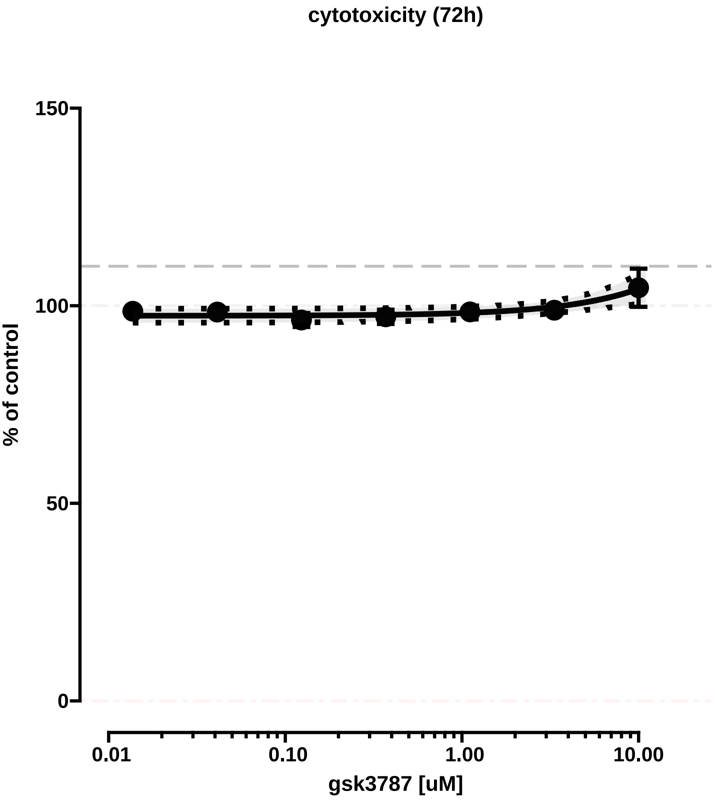

Model: Linear
Model abbr.: lm
Bechmark-Response (BMR): 10

BMCL: NA
BMC: NA
BMCU: NA

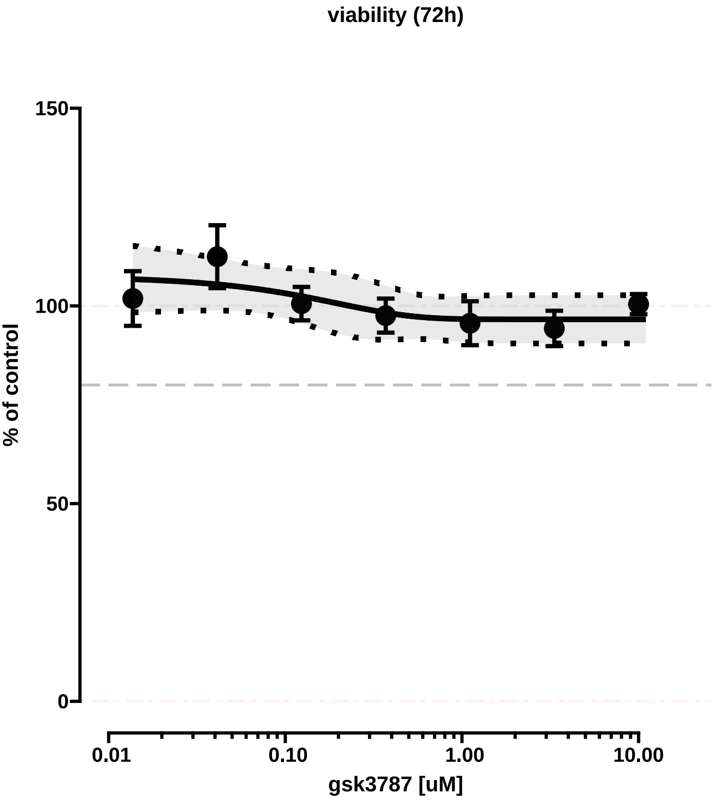

Model: Shifted exponential decay
Model abbr.: EXD.3()
Bechmark-Response (BMR): 20

BMCL: NA
BMC: NA
BMCU: NA

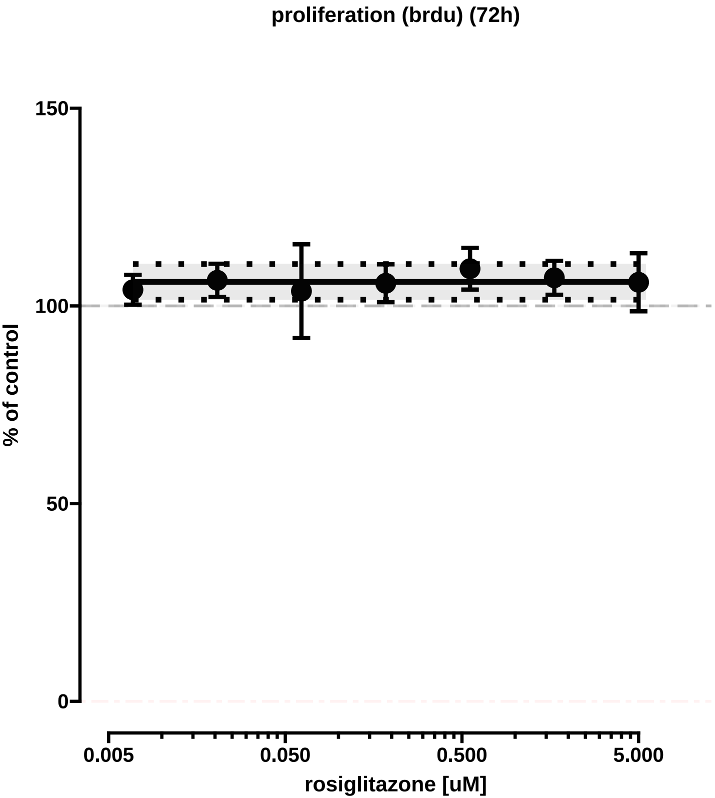

Model: 1-Parameter
Model abbr.: lm.1
Bechmark-Response (BMR): 20

BMCL: NA
BMC: NA
BMCU: NA

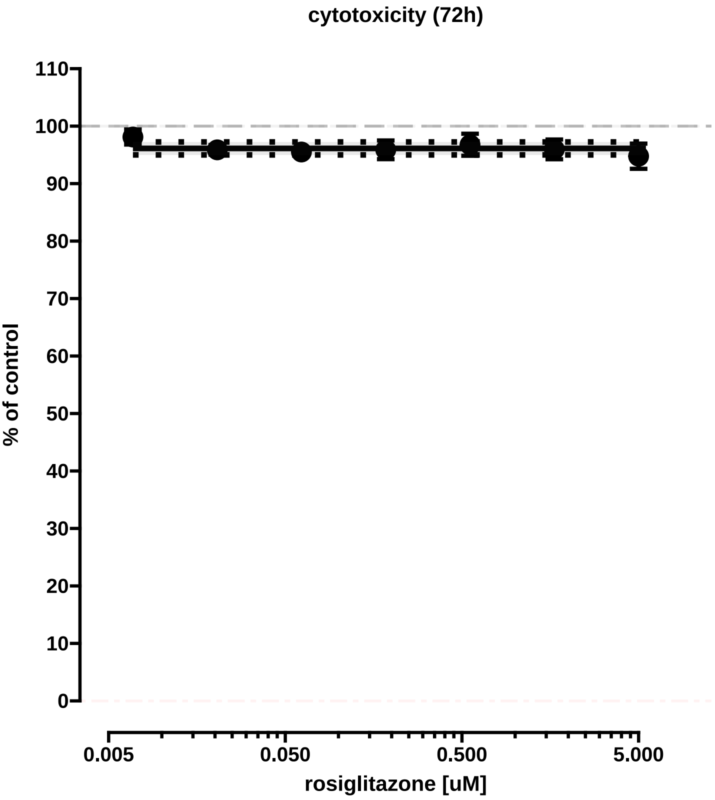

Model: 1-Parameter
Model abbr.: lm.1
Bechmark-Response (BMR): 10

BMCL: NA
BMC: NA
BMCU: NA

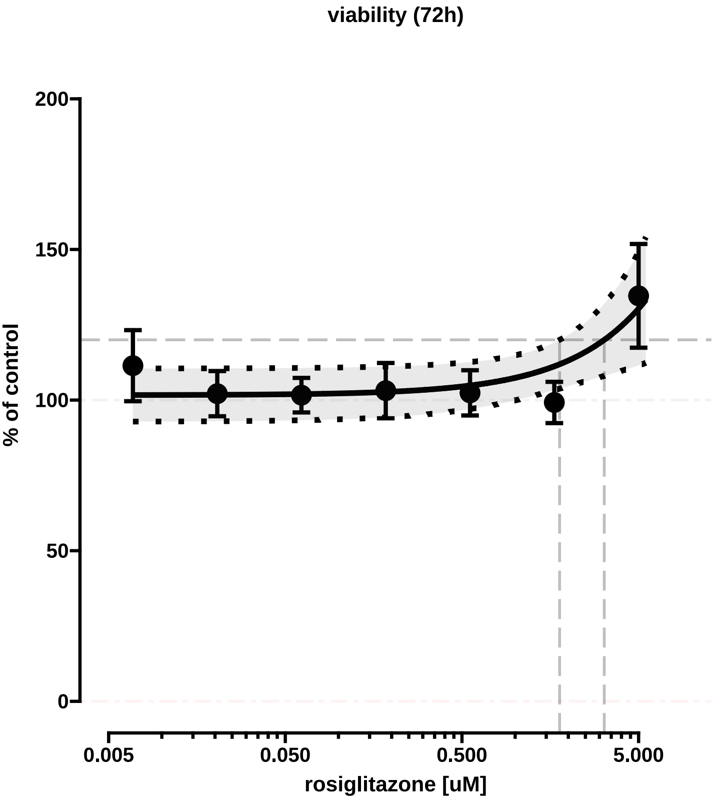

Model: Linear
Model abbr.: lm
Bechmark-Response (BMR): 20

BMCL: 1.786
BMC: 3.195
BMCU: NA

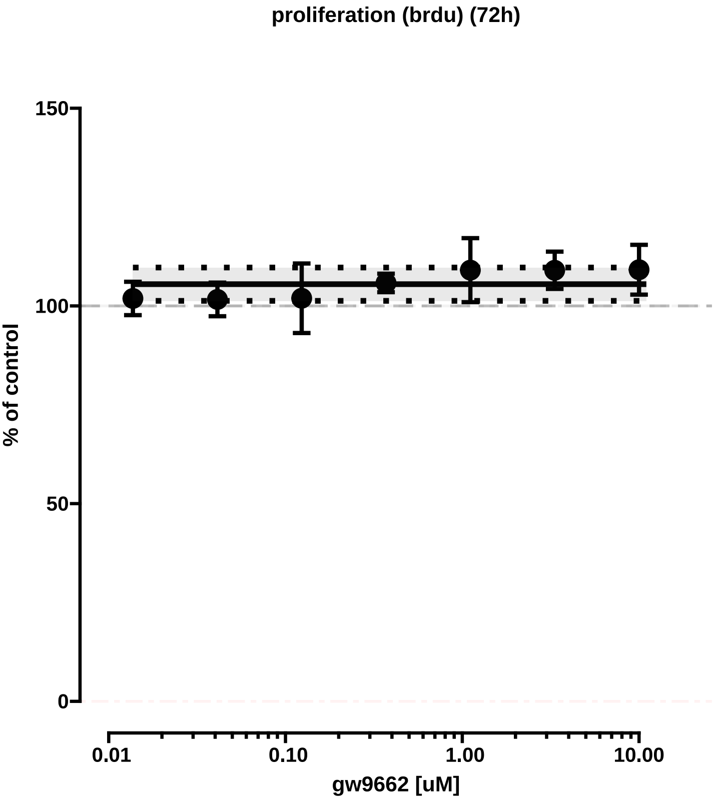

Model: 1-Parameter
Model abbr.: lm.1
Bechmark-Response (BMR): 20

BMCL: NA
BMC: NA
BMCU: NA

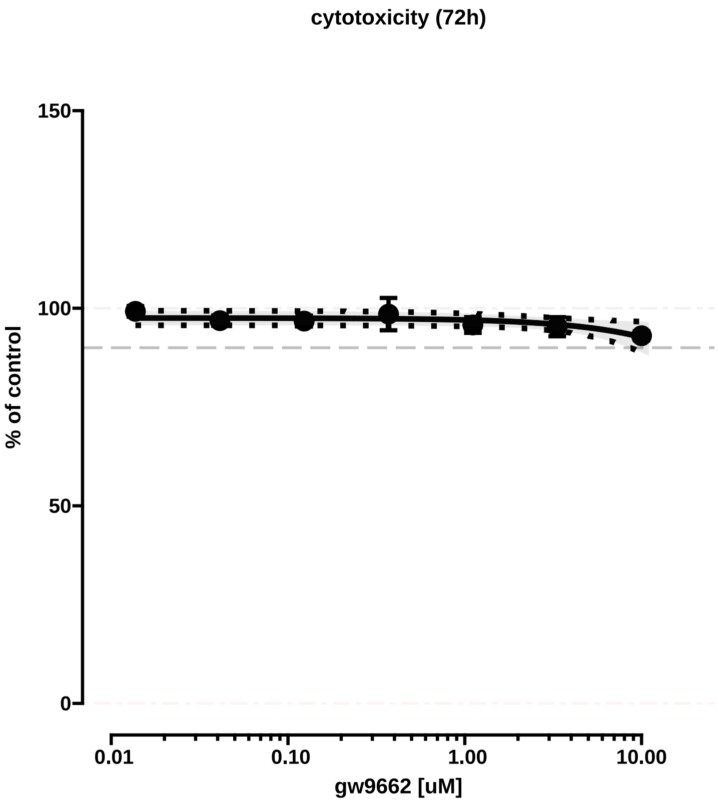

Model: Exponential decay with lower limit at 0
Model abbr.: EXD.2()
Bechmark-Response (BMR): 10

BMCL: NA
BMC: NA
BMCU: NA

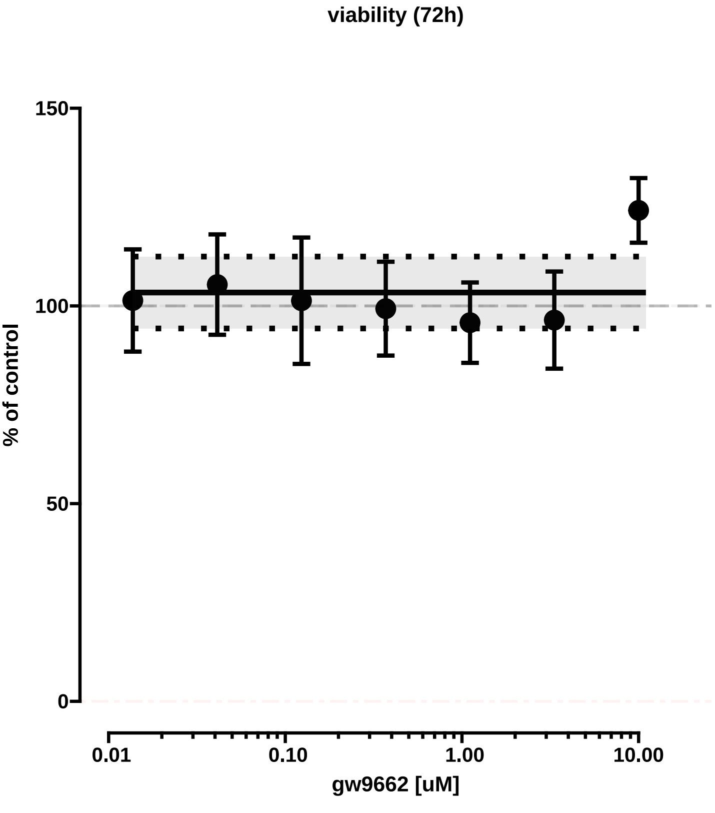

Model: 1-Parameter
Model abbr.: lm.1
Bechmark-Response (BMR): 20

BMCL: NA
BMC: NA
BMCU: NA

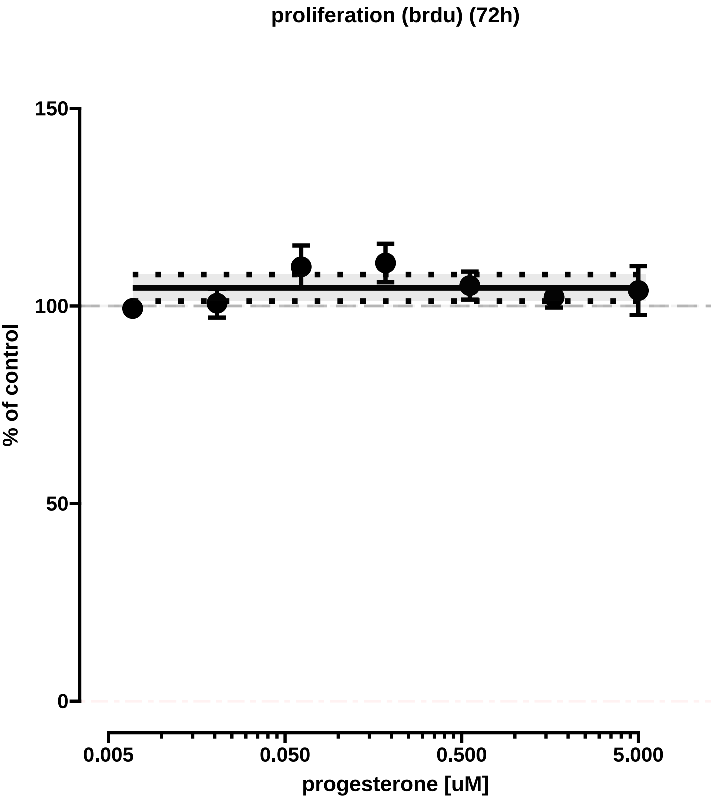

Model: 1-Parameter
Model abbr.: lm.1
Bechmark-Response (BMR): 20

BMCL: NA
BMC: NA
BMCU: NA

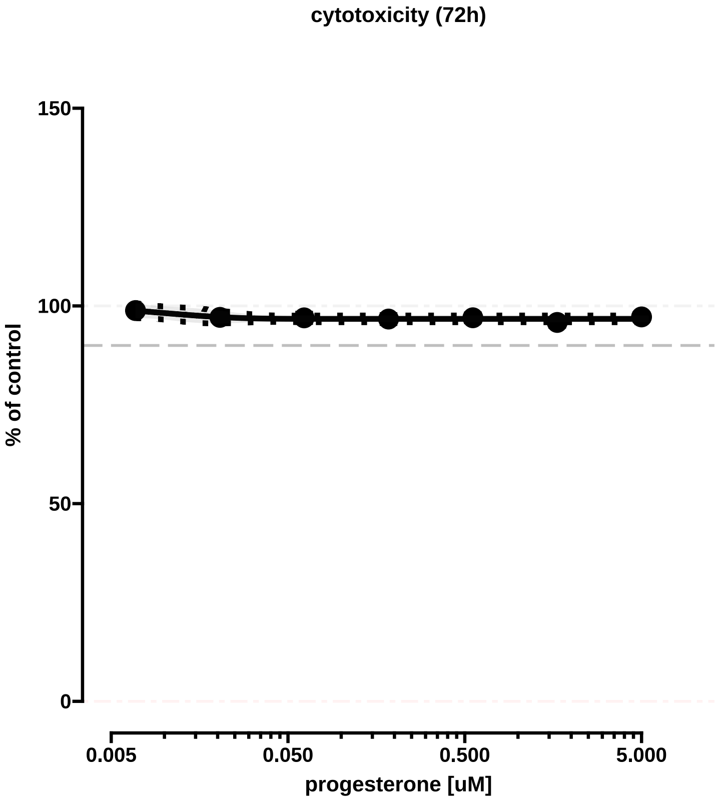

Model: Shifted exponential decay
Model abbr.: EXD.3()
Bechmark-Response (BMR): 10

BMCL: NA
BMC: NA
BMCU: NA

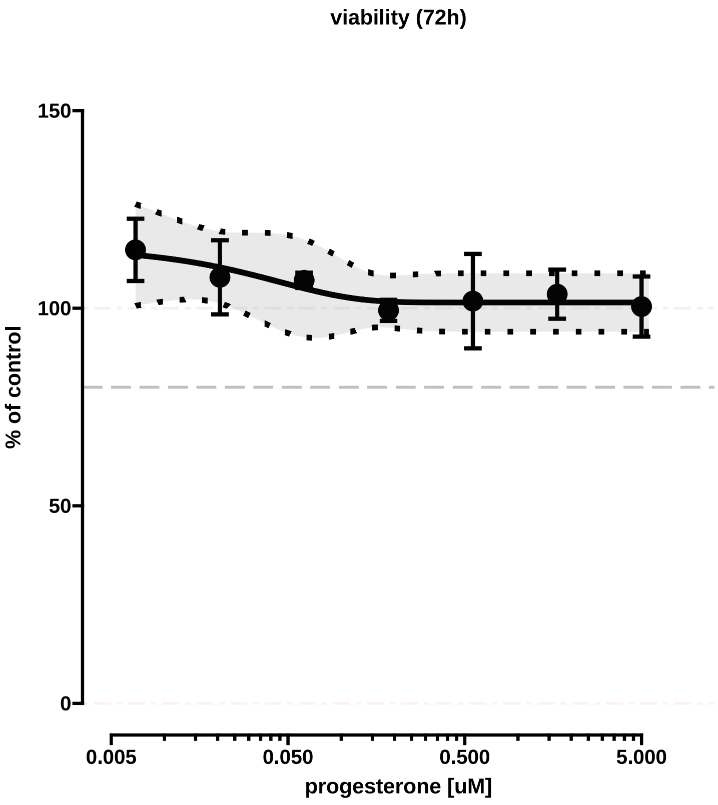

Model: Shifted exponential decay
Model abbr.: EXD.3()
Bechmark-Response (BMR): 20

BMCL: NA
BMC: NA
BMCU: NA

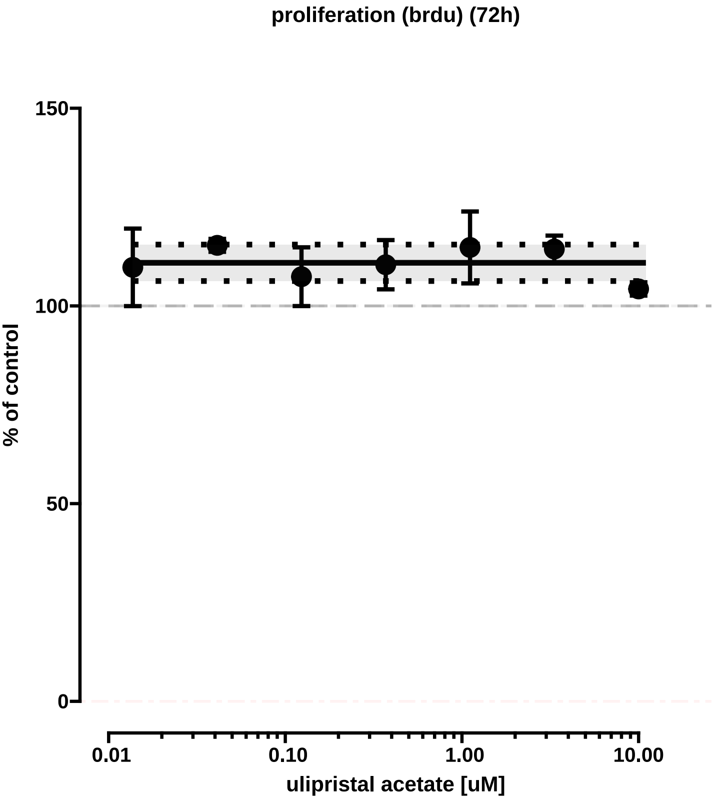

Model: 1-Parameter
Model abbr.: lm.1
Bechmark-Response (BMR): 20

BMCL: NA
BMC: NA
BMCU: NA

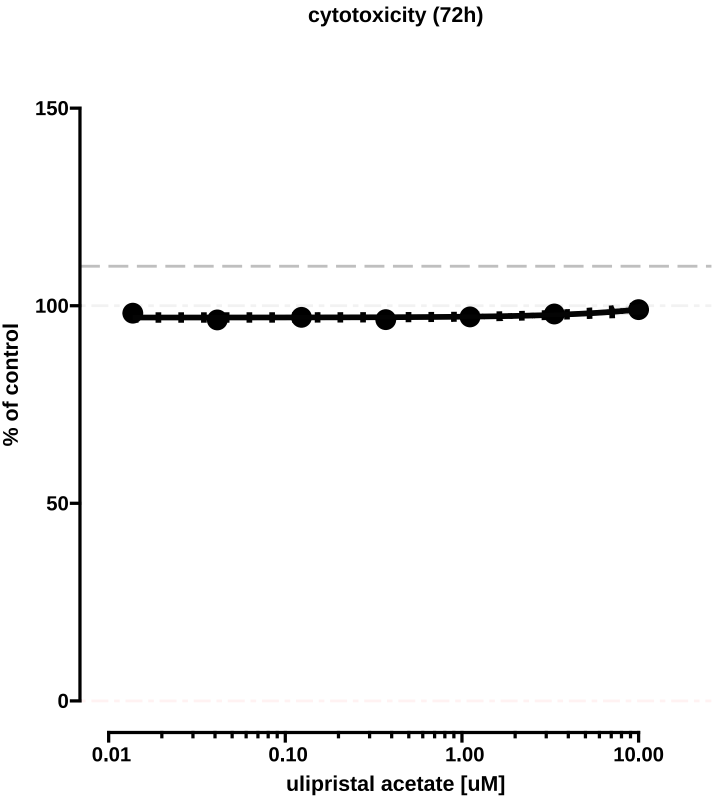

Model: Linear
Model abbr.: lm
Bechmark-Response (BMR): 10

BMCL: NA
BMC: NA
BMCU: NA

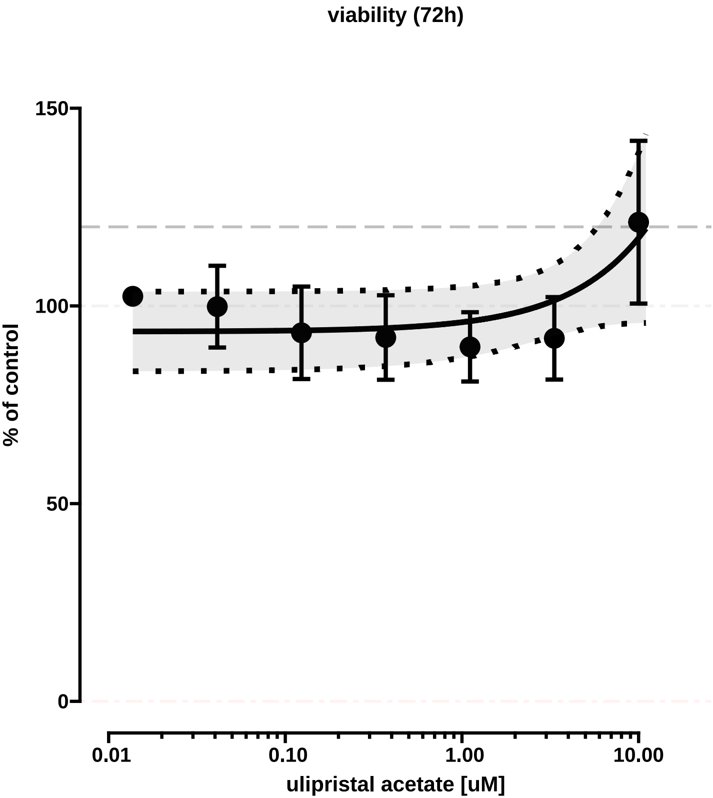

Model: Linear
Model abbr.: lm
Bechmark-Response (BMR): 20

BMCL: NA
BMC: NA
BMCU: NA

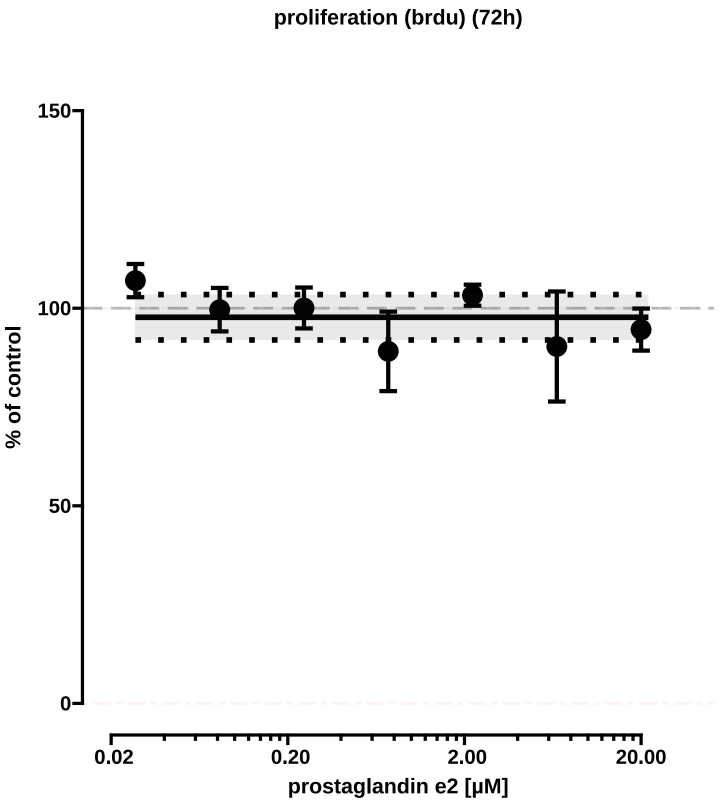

Model: 1-Parameter
Model abbr.: lm.1
Bechmark-Response (BMR): 20

BMCL: NA
BMC: NA
BMCU: NA

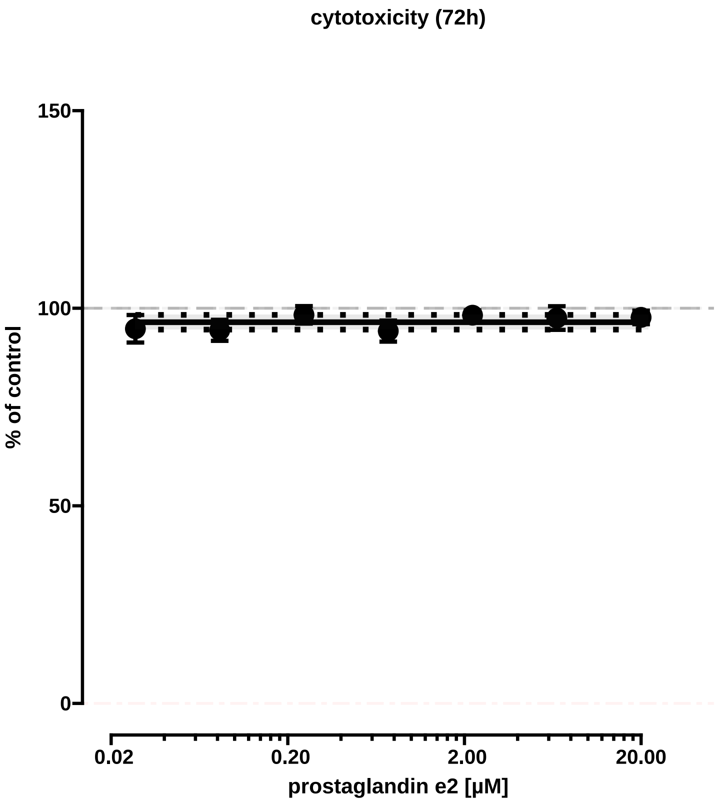

Model: 1-Parameter
Model abbr.: lm.1
Bechmark-Response (BMR): 10

BMCL: NA
BMC: NA
BMCU: NA

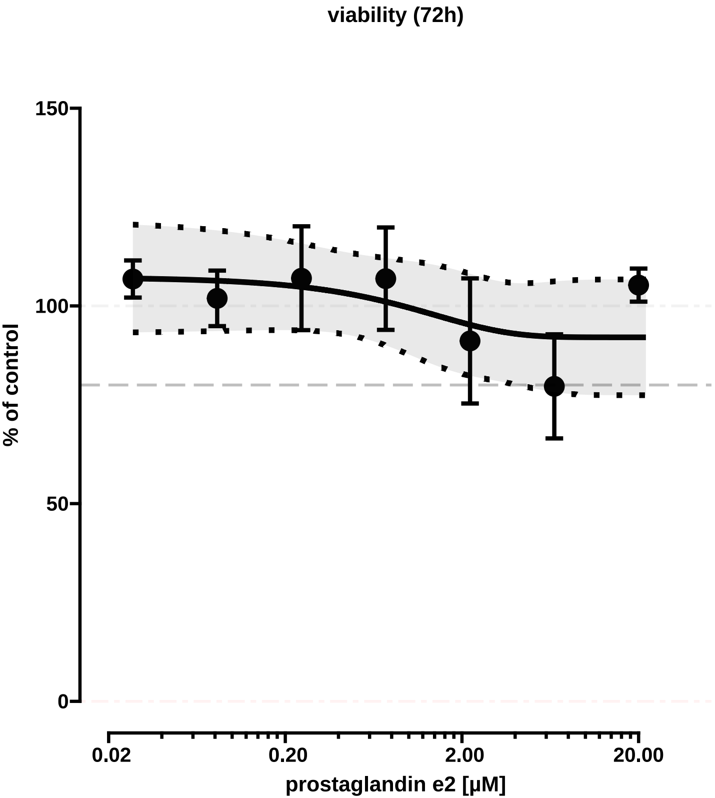

Model: Shifted exponential decay
Model abbr.: EXD.3()
Bechmark-Response (BMR): 20

BMCL: NA
BMC: NA
BMCU: NA

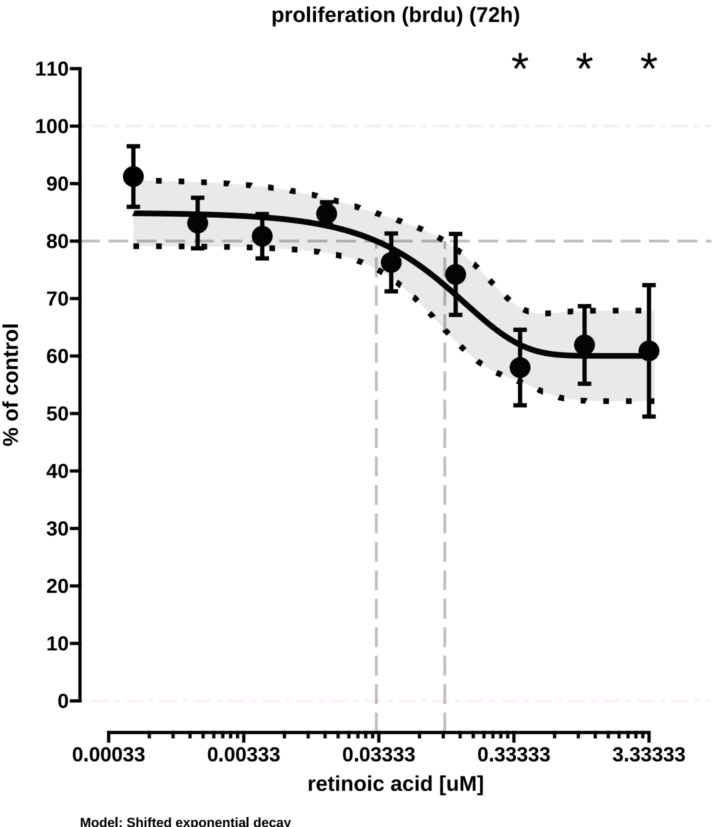

Model: Shifted exponential decay
Model abbr.: EXD.3()
Bechmark-Response (BMR): 20

BMCL: NA
BMC: 0.032
BMCU: 0.103

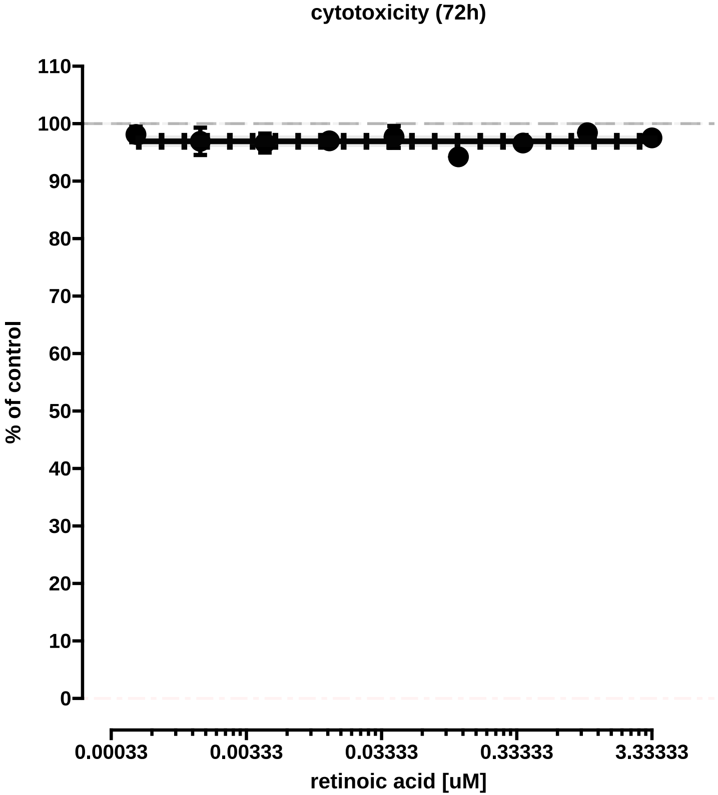

Model: 1-Parameter
Model abbr.: lm.1
Bechmark-Response (BMR): 10

BMCL: NA
BMC: NA
BMCU: NA

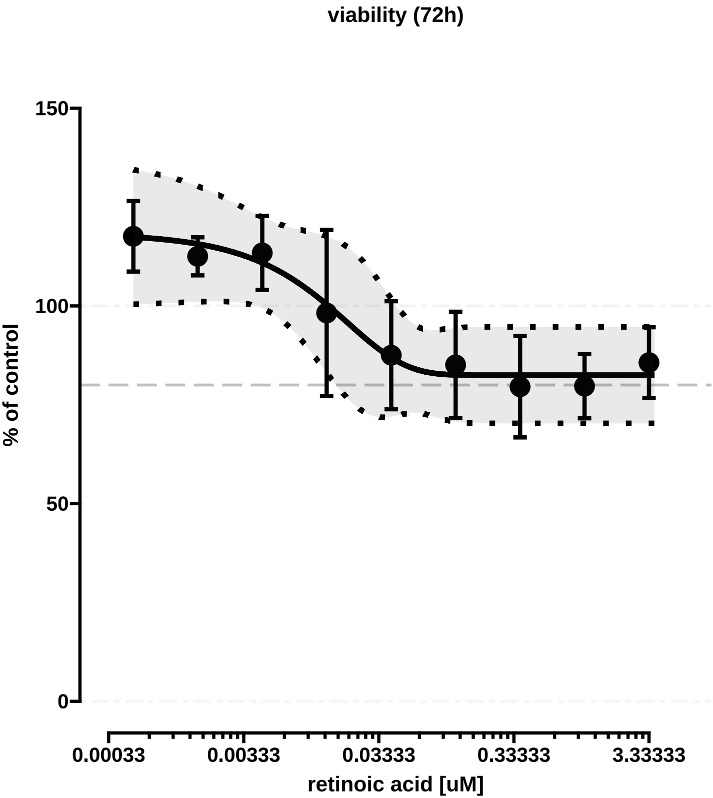

Model: Shifted exponential decay
Model abbr.: EXD.3()
Bechmark-Response (BMR): 20

BMCL: NA
BMC: NA
BMCU: NA

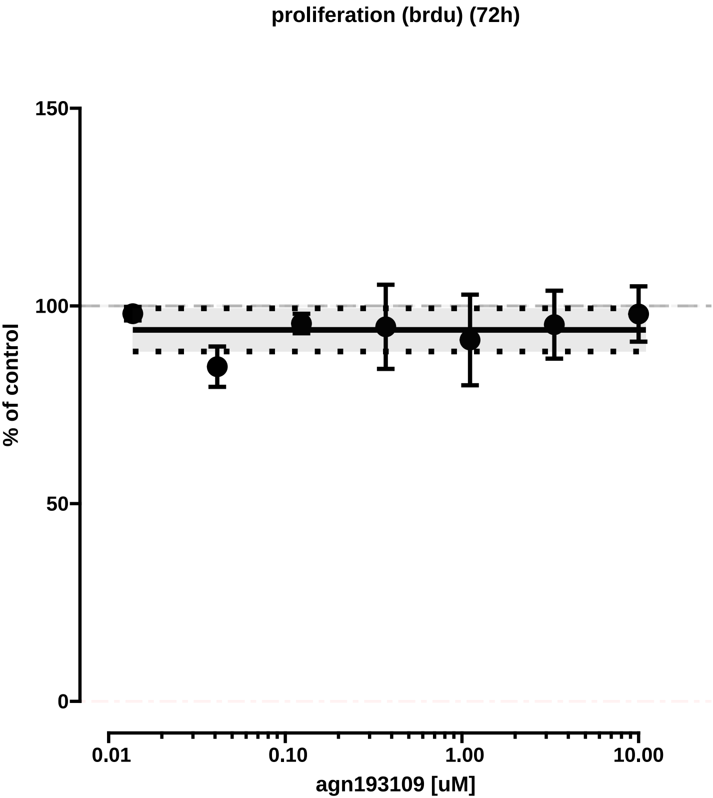

Model: 1-Parameter
Model abbr.: lm.1
Bechmark-Response (BMR): 20

BMCL: NA
BMC: NA
BMCU: NA

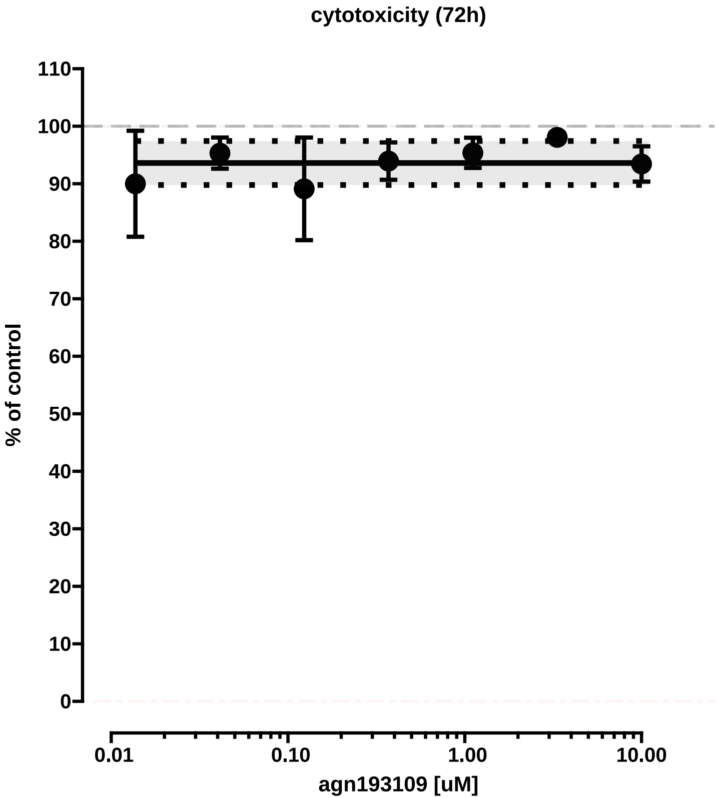

Model: 1-Parameter
Model abbr.: lm.1
Bechmark-Response (BMR): 10

BMCL: NA
BMC: NA
BMCU: NA

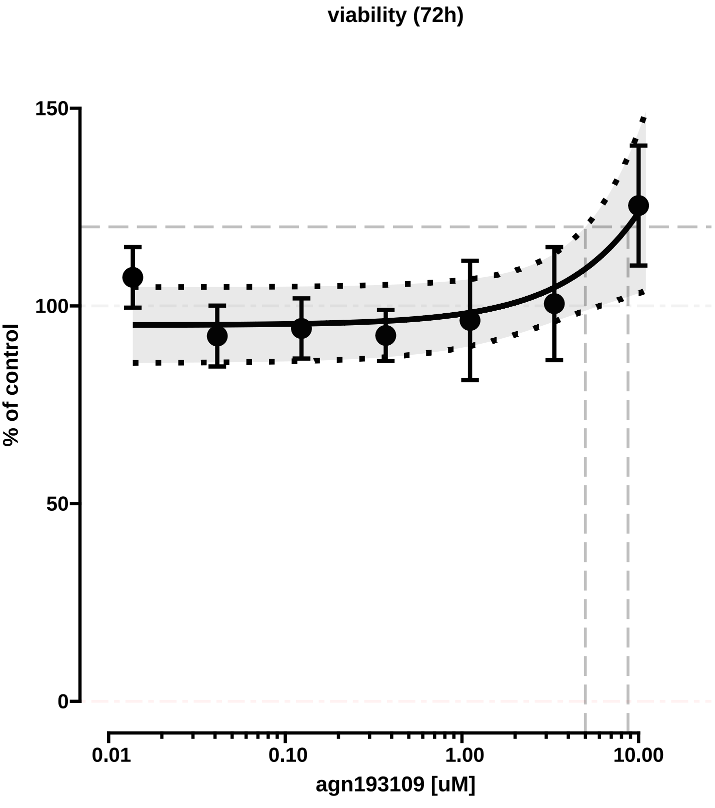

Model: Linear
Model abbr.: lm
Bechmark-Response (BMR): 20

BMCL: 4.996
BMC: 8.71
BMCU: NA

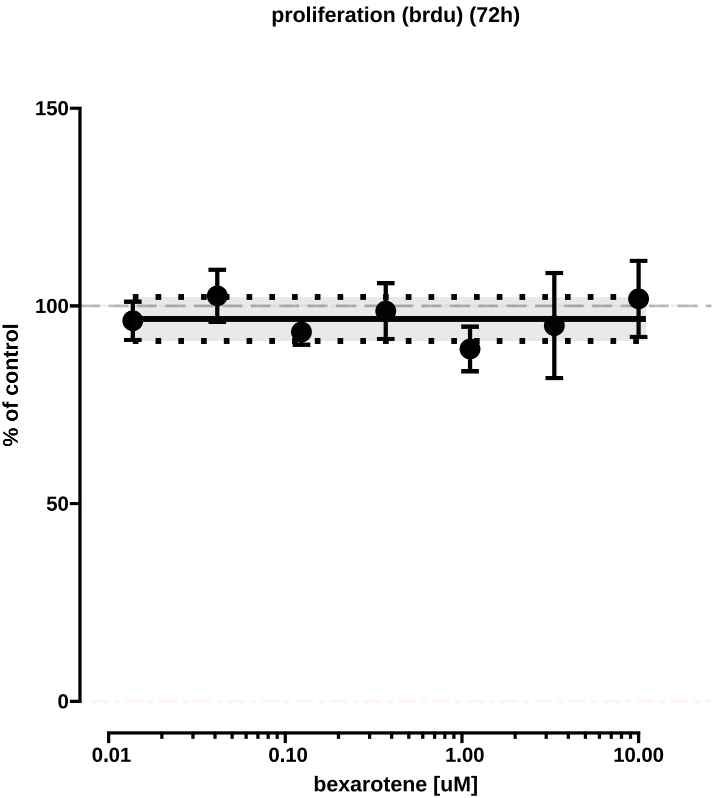

Model: 1-Parameter
Model abbr.: lm.1
Bechmark-Response (BMR): 20

BMCL: NA
BMC: NA
BMCU: NA

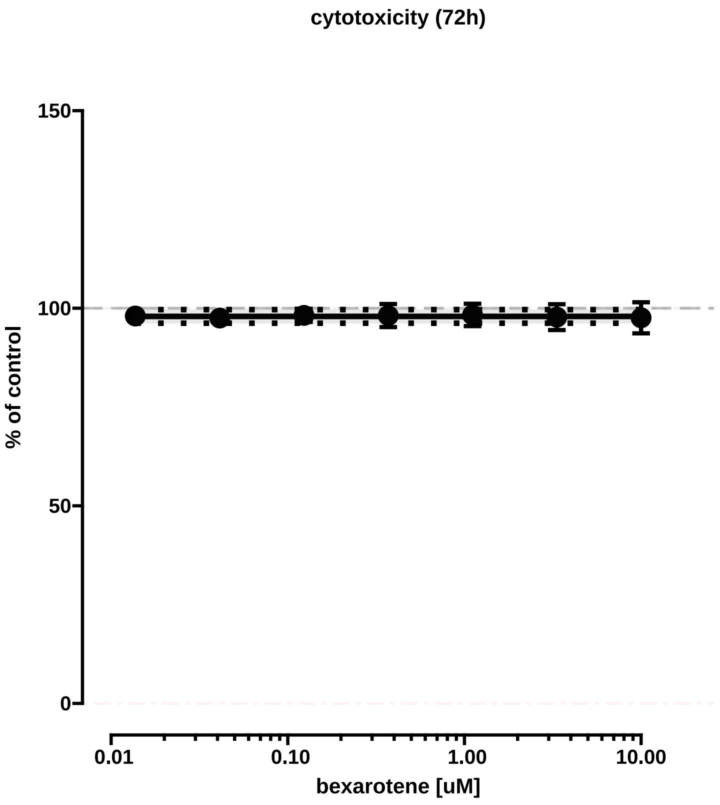

Model: 1-Parameter
Model abbr.: lm.1
Bechmark-Response (BMR): 10

BMCL: NA
BMC: NA
BMCU: NA

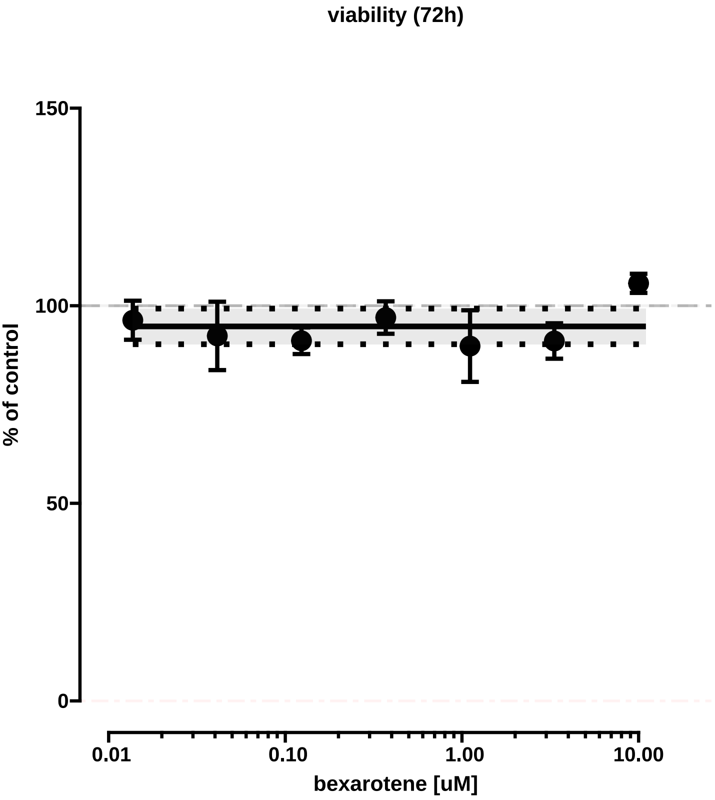

Model: 1-Parameter
Model abbr.: lm.1
Bechmark-Response (BMR): 20

BMCL: NA
BMC: NA
BMCU: NA

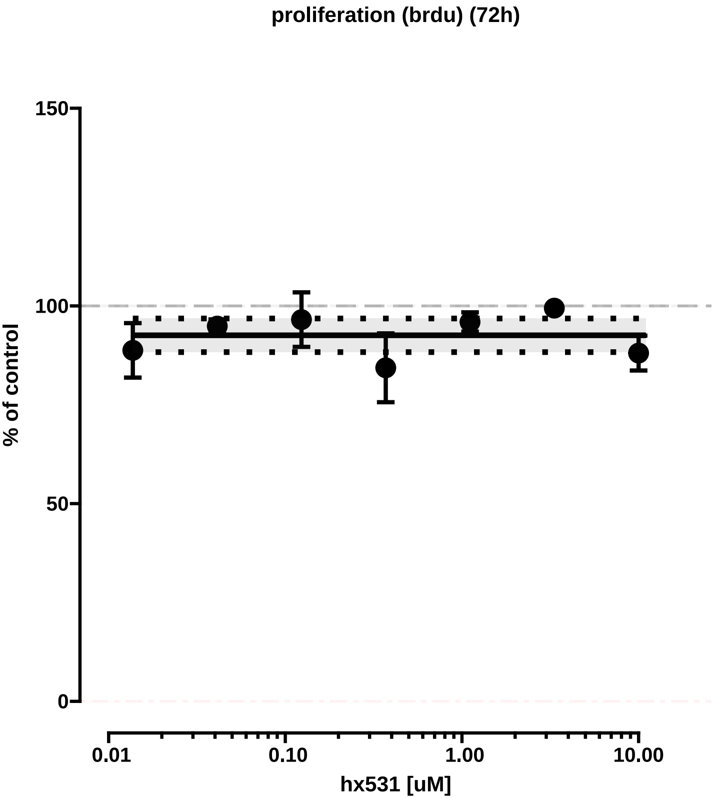

Model: 1-Parameter
Model abbr.: lm.1
Bechmark-Response (BMR): 20

BMCL: NA
BMC: NA
BMCU: NA

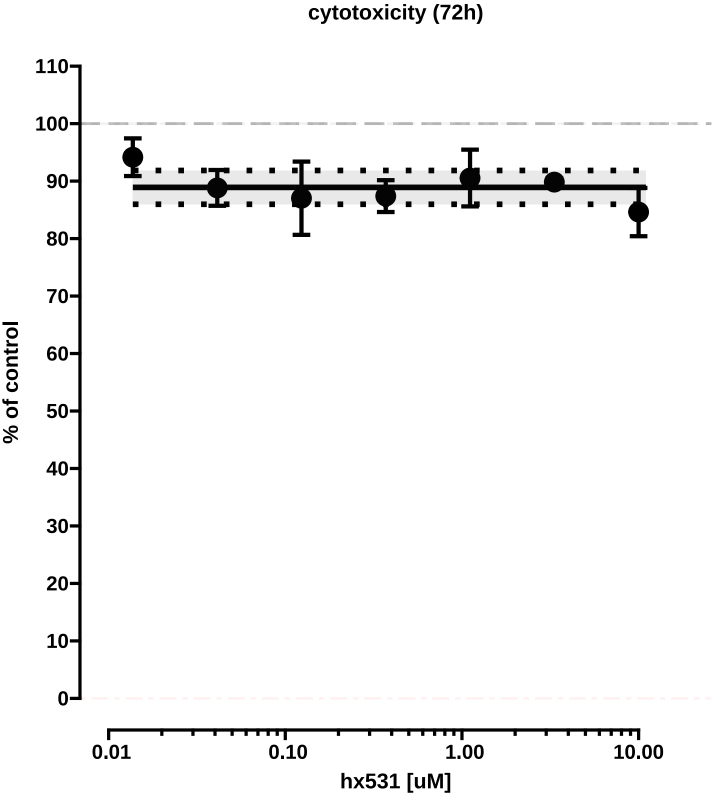

Model: 1-Parameter
Model abbr.: lm.1
Bechmark-Response (BMR): 10

BMCL: NA
BMC: NA
BMCU: NA

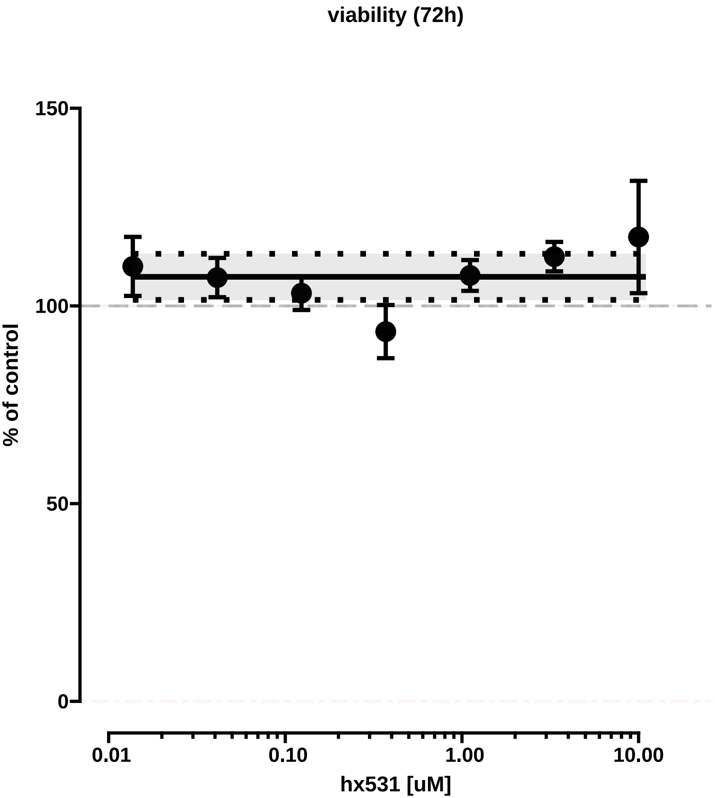

Model: 1-Parameter
Model abbr.: lm.1
Bechmark-Response (BMR): 20

BMCL: NA
BMC: NA
BMCU: NA

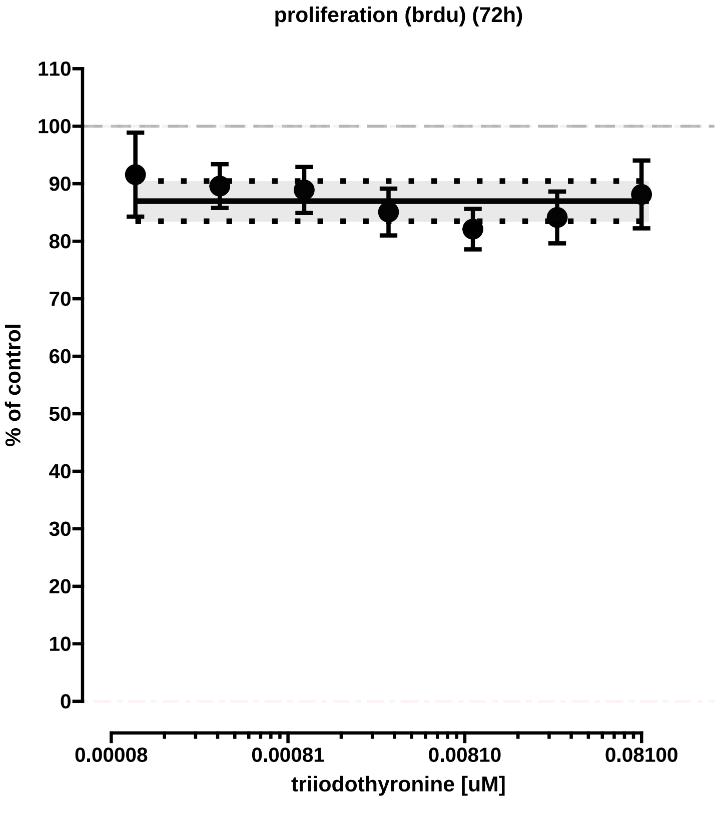

Model: 1-Parameter
Model abbr.: lm.1
Bechmark-Response (BMR): 20

BMCL: NA
BMC: NA
BMCU: NA

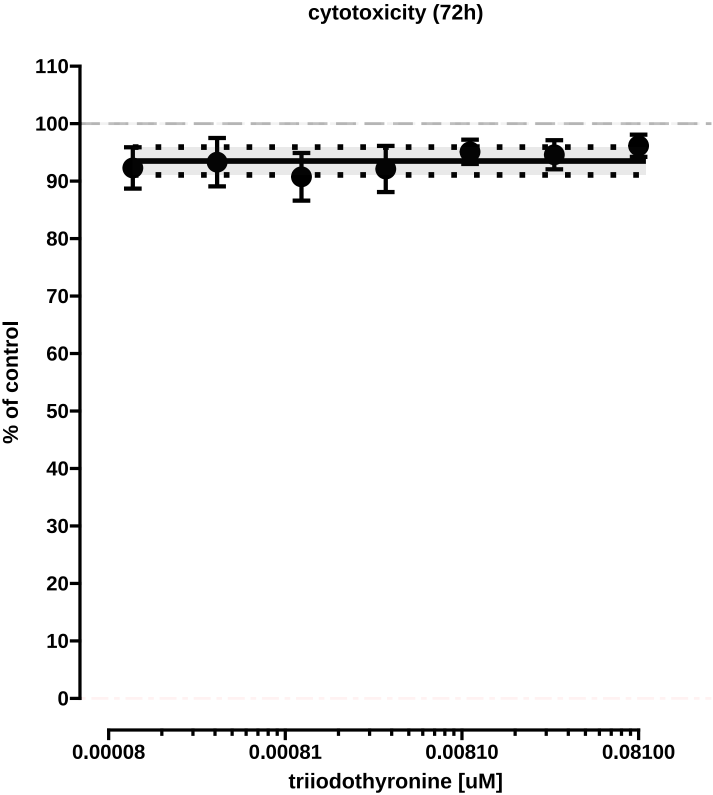

Model: 1-Parameter
Model abbr.: lm.1
Bechmark-Response (BMR): 10

BMCL: NA
BMC: NA
BMCU: NA

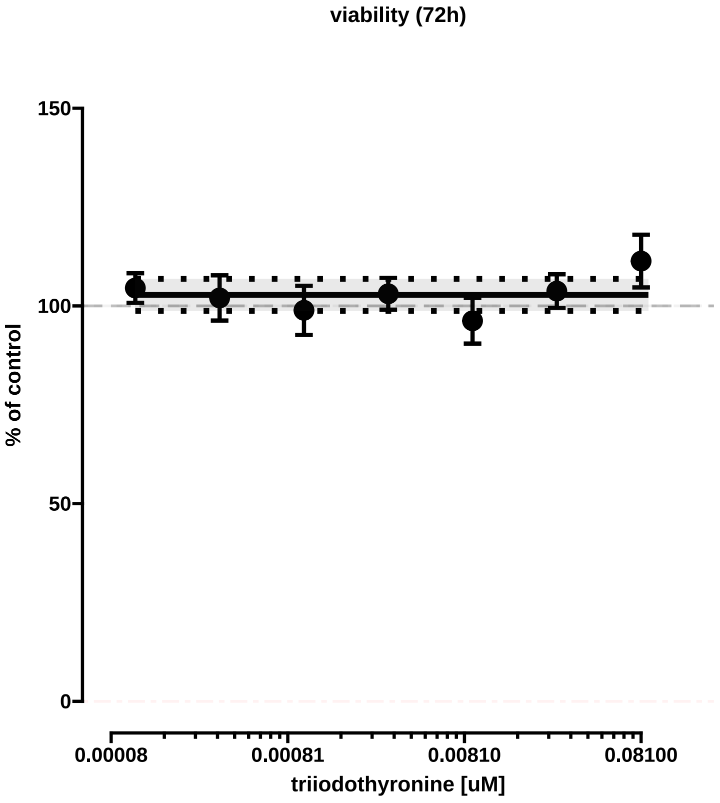

Model: 1-Parameter
Model abbr.: lm.1
Bechmark-Response (BMR): 20

BMCL: NA
BMC: NA
BMCU: NA

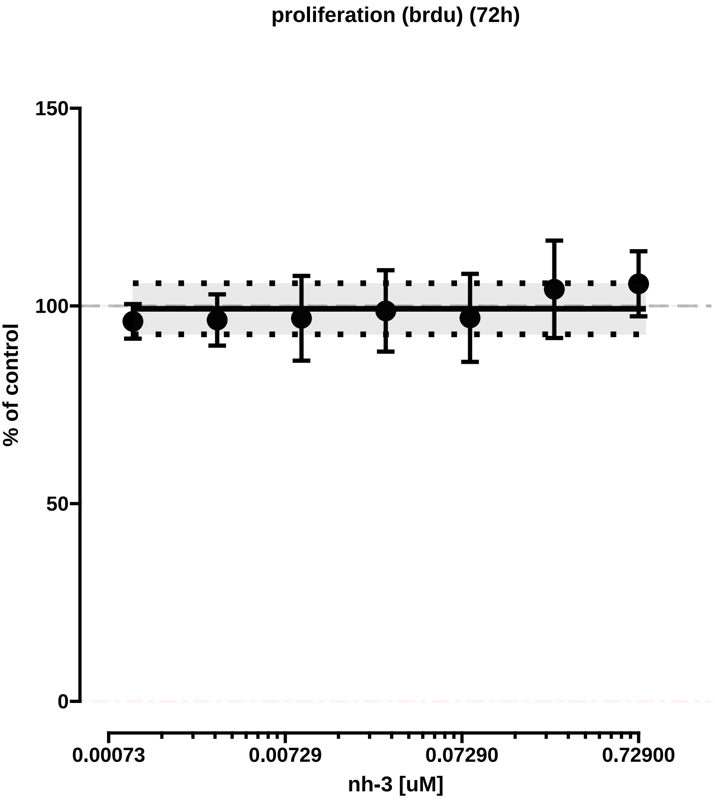

Model: 1-Parameter
Model abbr.: lm.1
Bechmark-Response (BMR): 20

BMCL: NA
BMC: NA
BMCU: NA

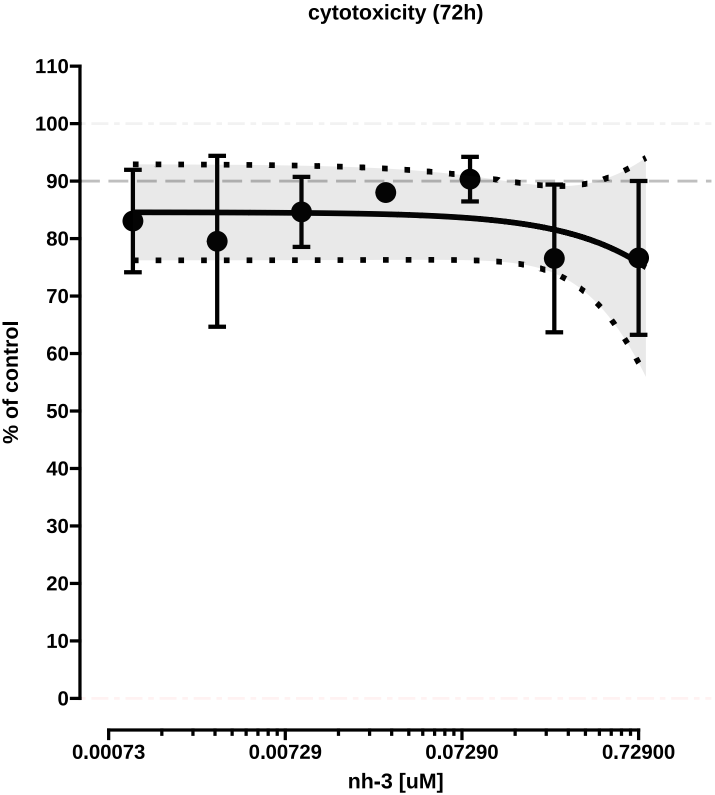

Model: Exponential decay with lower limit at 0
Model abbr.: EXD.2()
Bechmark-Response (BMR): 10

BMCL: NA
BMC: NA
BMCU: NA

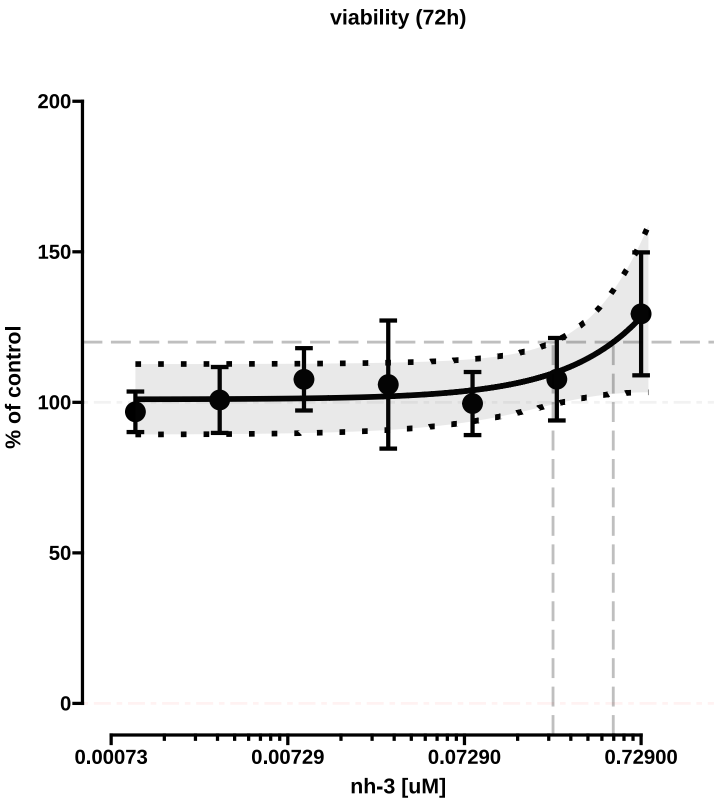

Model: Linear
Model abbr.: lm
Bechmark-Response (BMR): 20

BMCL: 0.231
BMC: 0.507
BMCU: NA

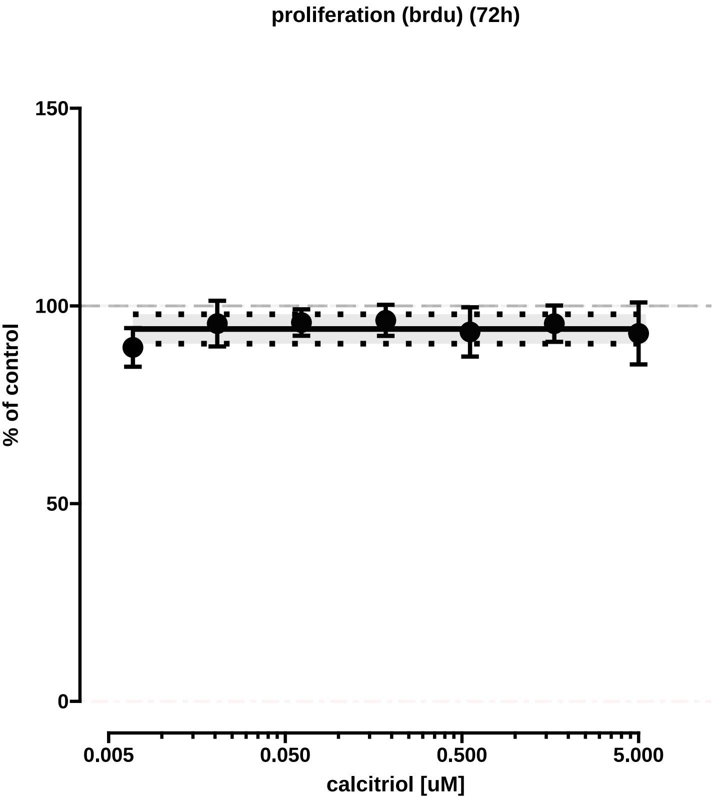

Model: 1-Parameter
Model abbr.: lm.1
Bechmark-Response (BMR): 20

BMCL: NA
BMC: NA
BMCU: NA

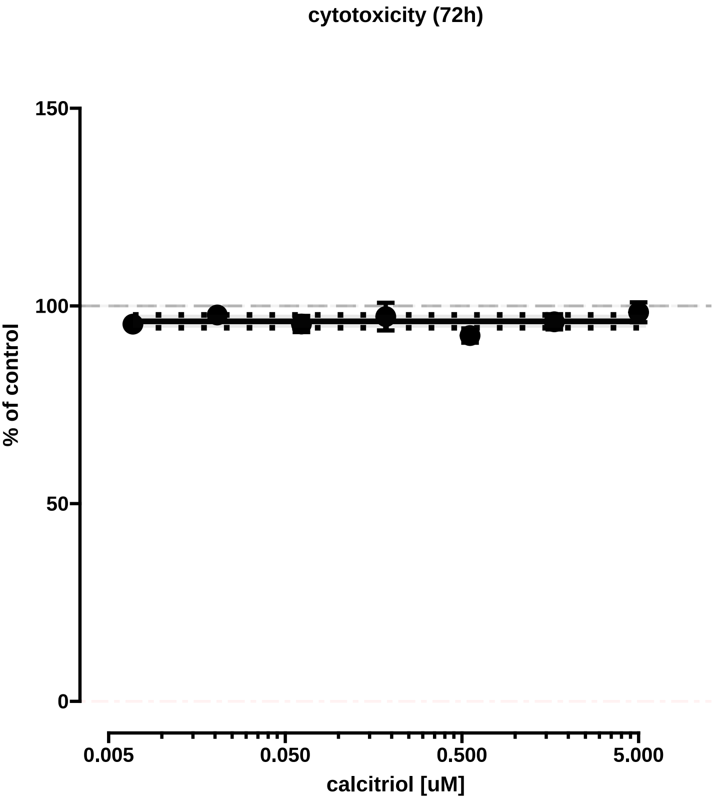

Model: 1-Parameter
Model abbr.: lm.1
Bechmark-Response (BMR): 10

BMCL: NA
BMC: NA
BMCU: NA

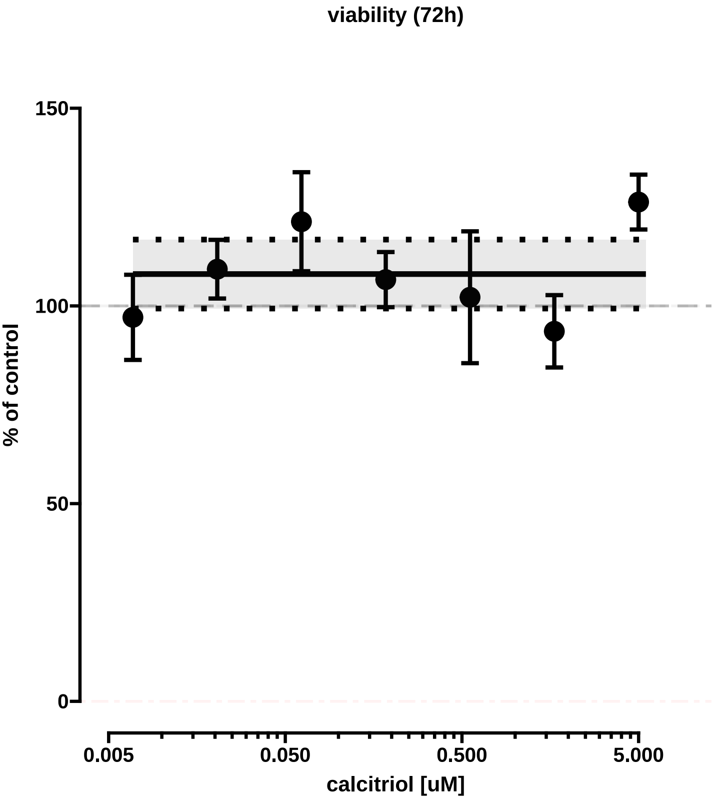

Model: 1-Parameter
Model abbr.: lm.1
Bechmark-Response (BMR): 20

BMCL: NA
BMC: NA
BMCU: NA
