# Supplementary material for: Nuclear hormone receptors control fundamental processes of human fetal neurodevelopment: Basis for endocrine disruption assessment
Source: Environ Int. Author manuscript; Available in PMC 2025 Jun 2. (PMC12127433; doi:10.1016/j.envint.2025.109400)
Supplement: 9 [file NIHMS2077722-supplement-9.pdf]

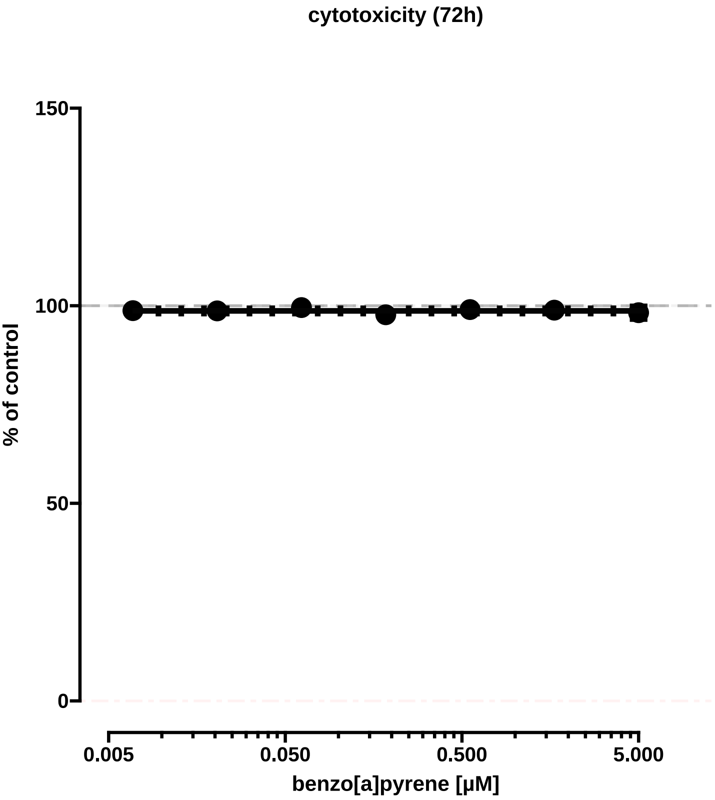

Model: 1-Parameter
Model abbr.: lm.1
Bechmark-Response (BMR): 10

BMCL: NA
BMC: NA
BMCU: NA

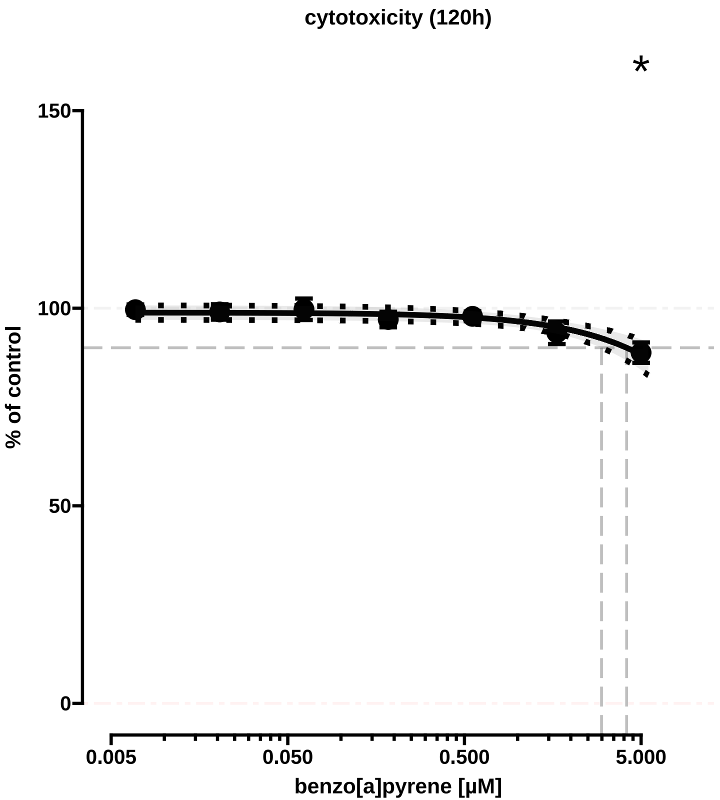

Model: Exponential decay with lower limit at 0
Model abbr.: EXD.2()
Bechmark-Response (BMR): 10

BMCL: 2.985
BMC: 4.139
BMCU: NA

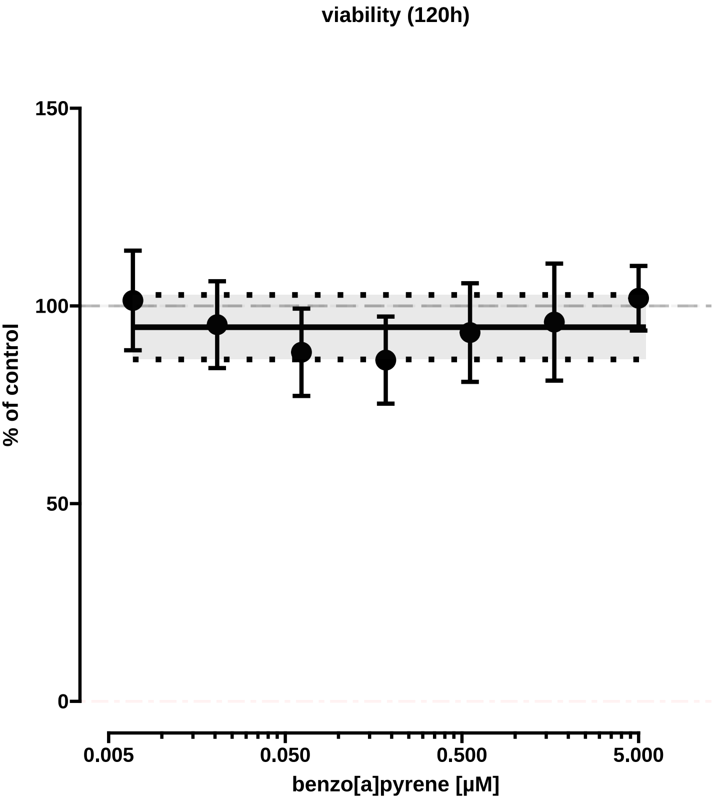

Model: 1-Parameter
Model abbr.: lm.1
Bechmark-Response (BMR): 20

BMCL: NA
BMC: NA
BMCU: NA

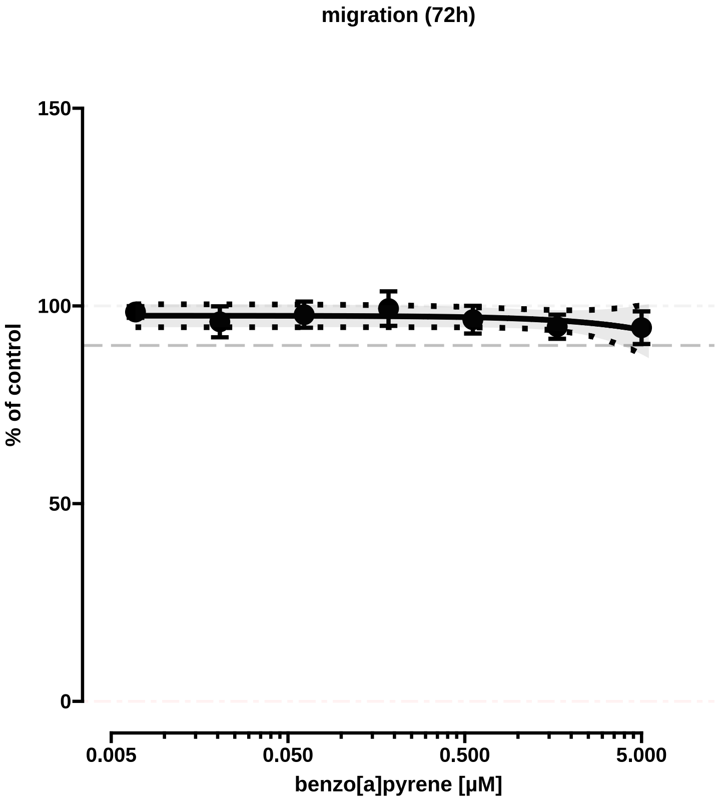

Model: Exponential decay with lower limit at 0
Model abbr.: EXD.2()
Bechmark-Response (BMR): 10

BMCL: NA
BMC: NA
BMCU: NA

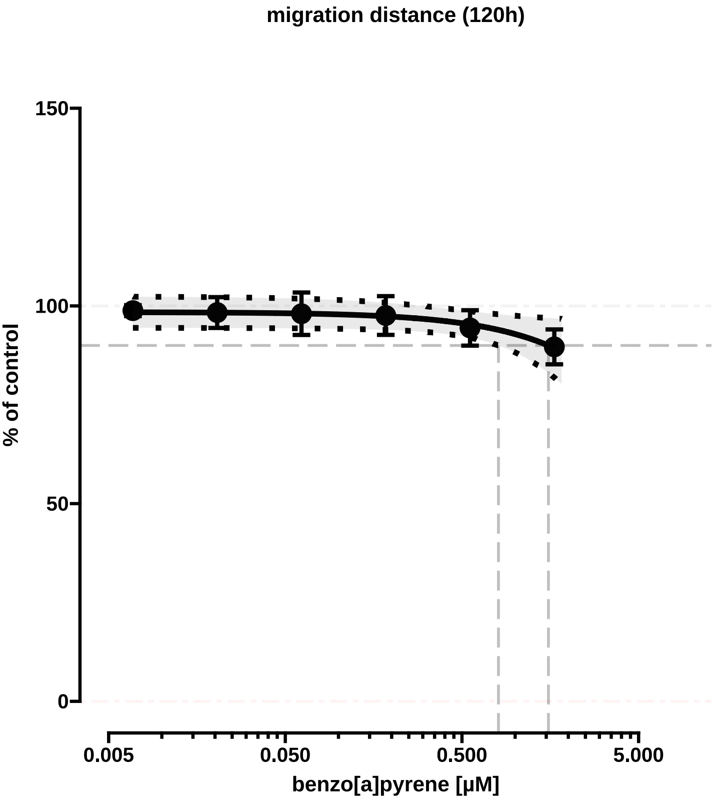

Model: Exponential decay with lower limit at 0
Model abbr.: EXD.2()
Bechmark-Response (BMR): 10

BMCL: 0.805
BMC: 1.544
BMCU: NA

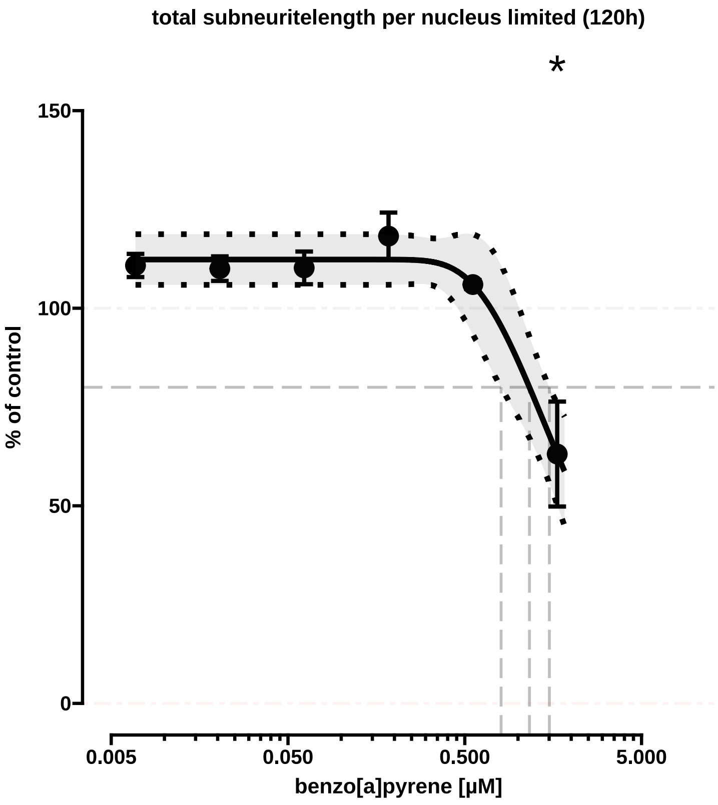

Model: Weibull (type 2) with lower limit at 0
Model abbr.: W2.3()
Bechmark-Response (BMR): 20

BMCL: 0.803
BMC: 1.161
BMCU: 1.505

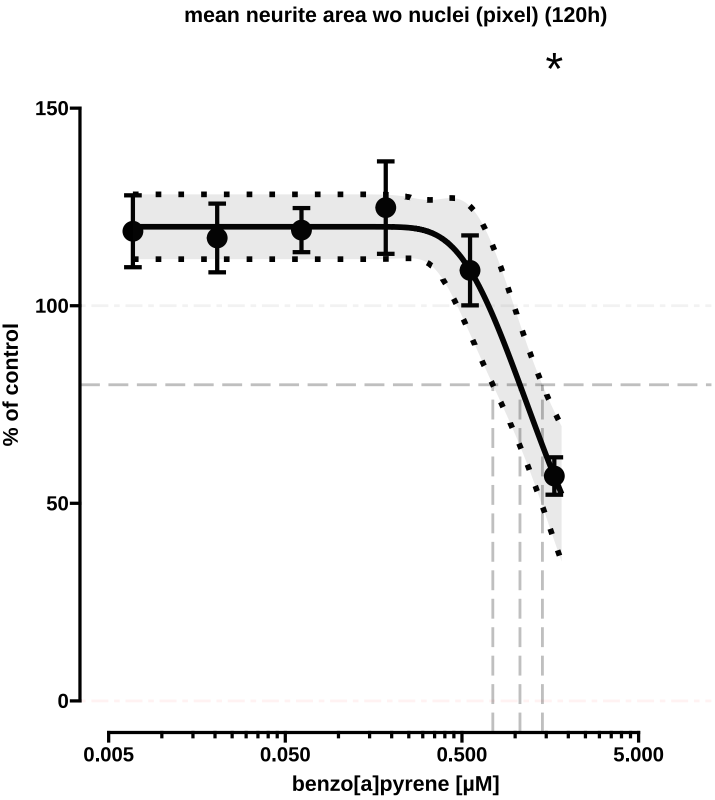

Model: Weibull (type 2) with lower limit at 0
Model abbr.: W2.3()
Bechmark-Response (BMR): 20

BMCL: 0.748
BMC: 1.065
BMCU: 1.427

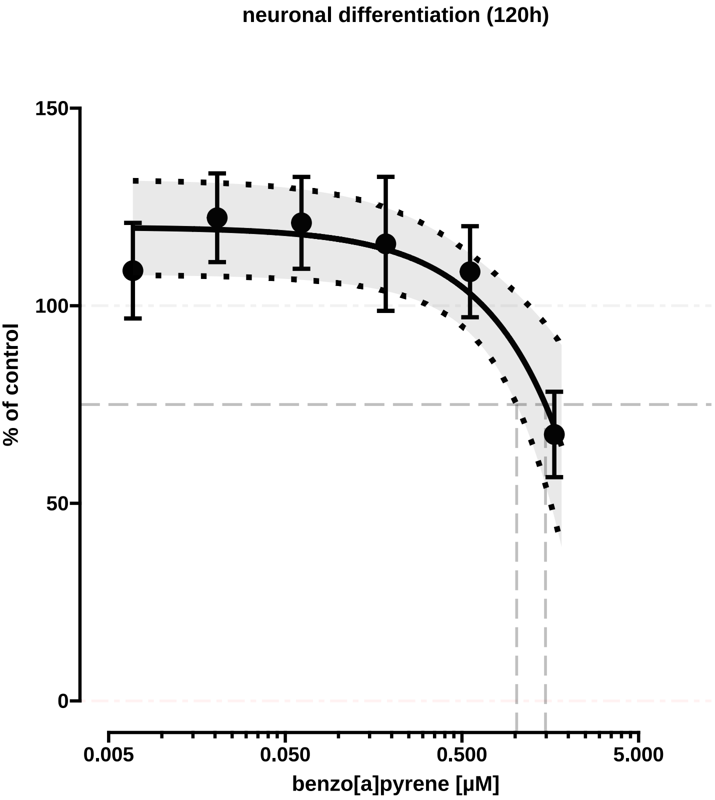

Model: Linear
Model abbr.: lm
Bechmark-Response (BMR): 25

BMCL: 1.02
BMC: 1.486
BMCU: NA

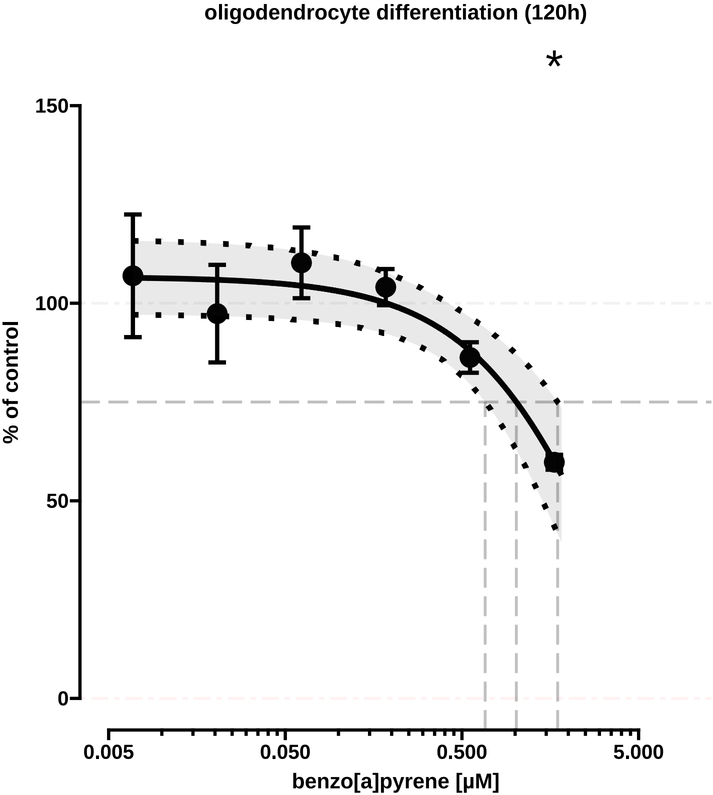

Model: Exponential decay with lower limit at 0
Model abbr.: EXD.2()
Bechmark-Response (BMR): 25

BMCL: 0.677
BMC: 1.016
BMCU: 1.741

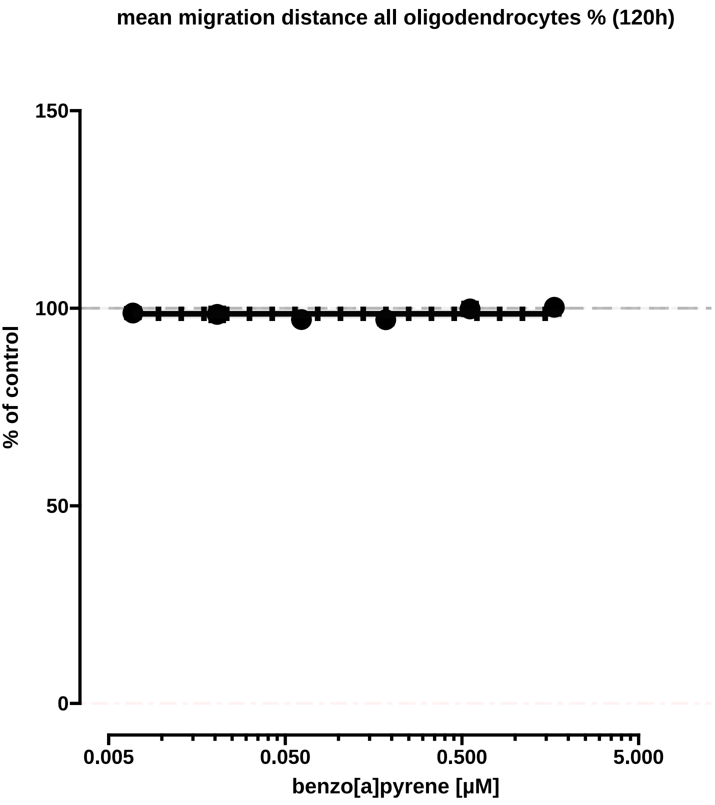

Model: 1-Parameter
Model abbr.: lm.1
Bechmark-Response (BMR): 10

BMCL: NA
BMC: NA
BMCU: NA

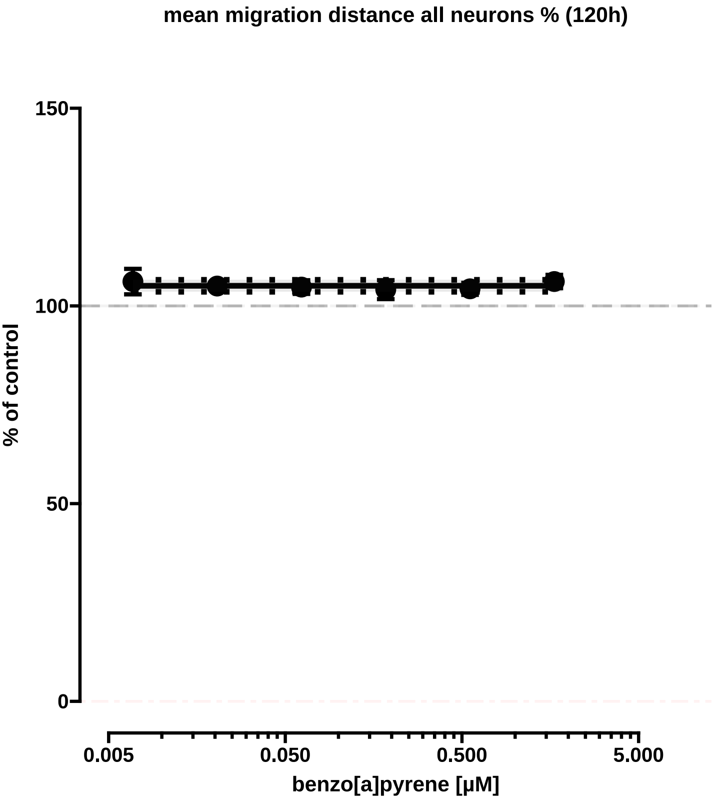

Model: 1-Parameter
Model abbr.: lm.1
Bechmark-Response (BMR): 10

BMCL: NA
BMC: NA
BMCU: NA

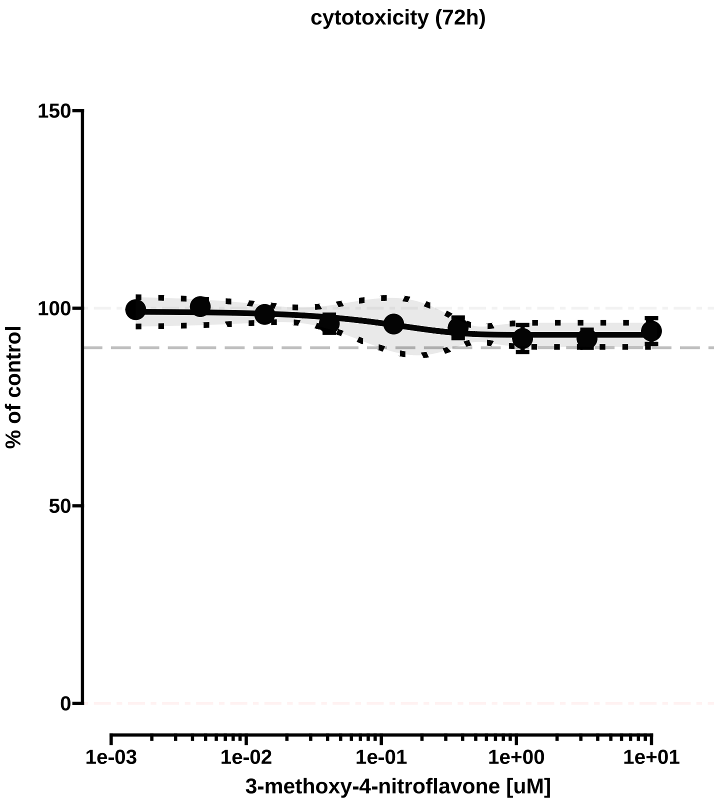

Model: Shifted exponential decay
Model abbr.: EXD.3()
Bechmark-Response (BMR): 10

BMCL: NA
BMC: NA
BMCU: NA

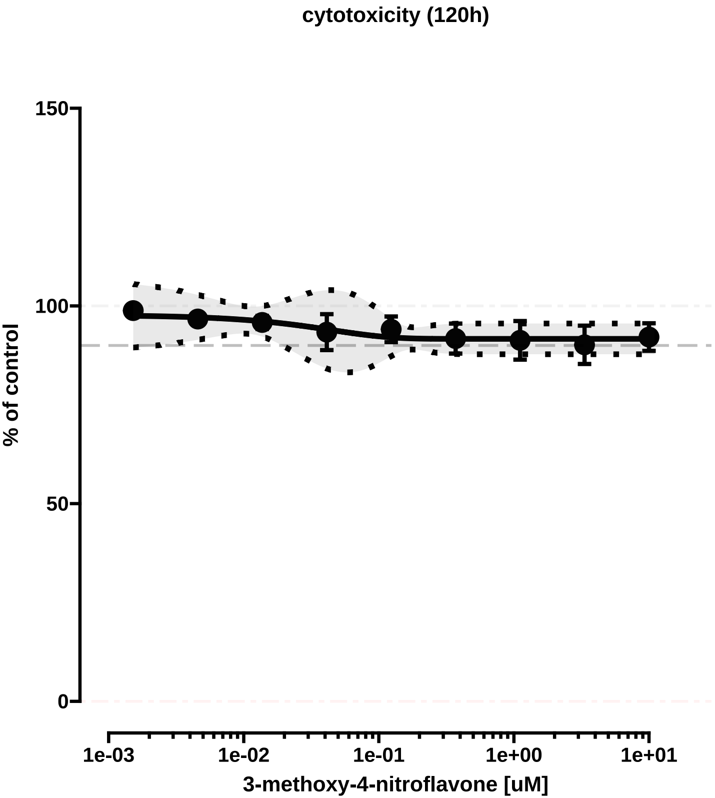

Model: Shifted exponential decay
Model abbr.: EXD.3()
Bechmark-Response (BMR): 10

BMCL: NA
BMC: NA
BMCU: NA

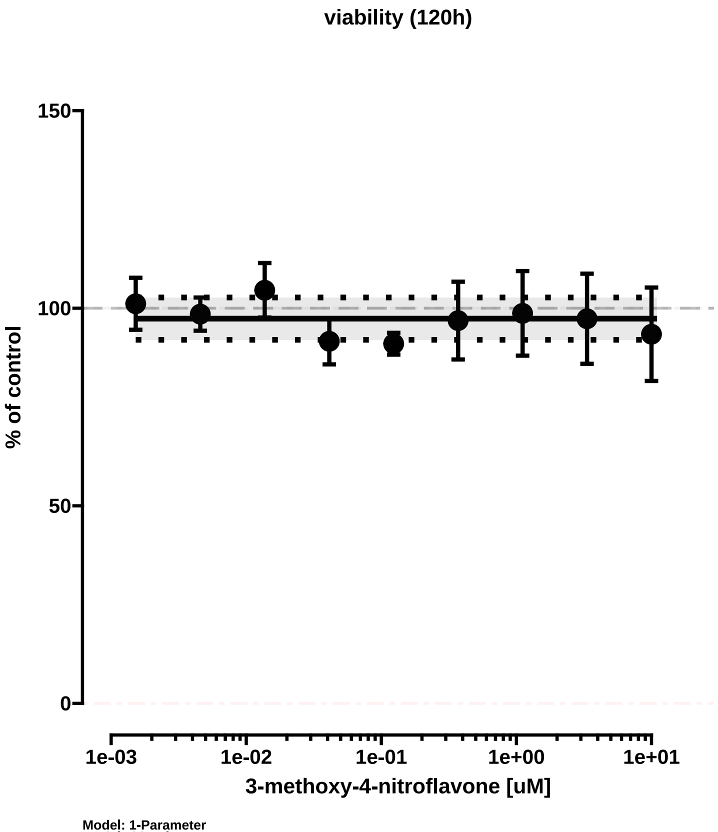

Model: 1-Parameter
Model abbr.: lm.1
Bechmark-Response (BMR): 20

BMCL: NA
BMC: NA
BMCU: NA

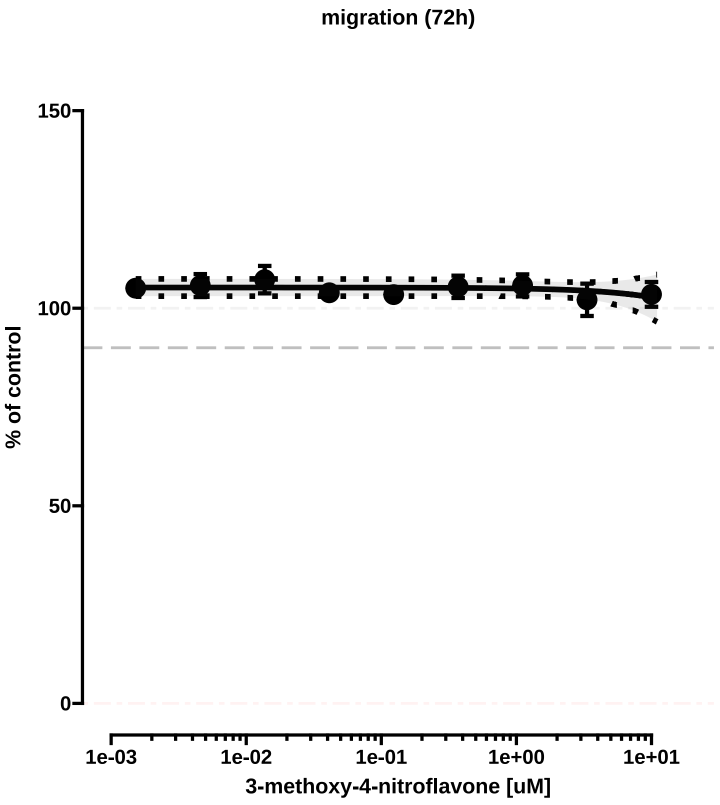

Model: Exponential decay with lower limit at 0
Model abbr.: EXD.2()
Bechmark-Response (BMR): 10

BMCL: NA
BMC: NA
BMCU: NA

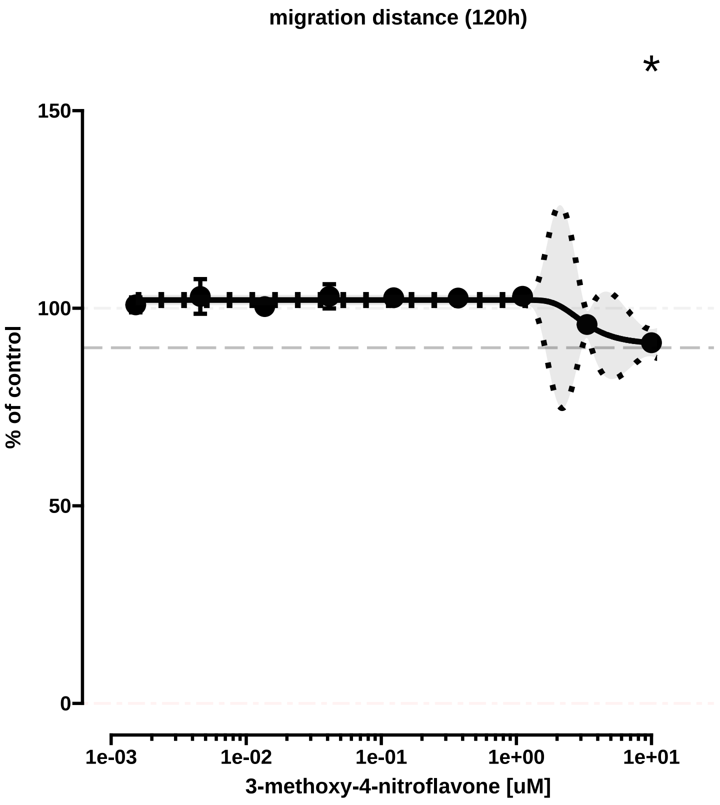

Model: Weibull (type 2)
Model abbr.: W2.4()
Bechmark-Response (BMR): 10

BMCL: NA
BMC: NA
BMCU: NA

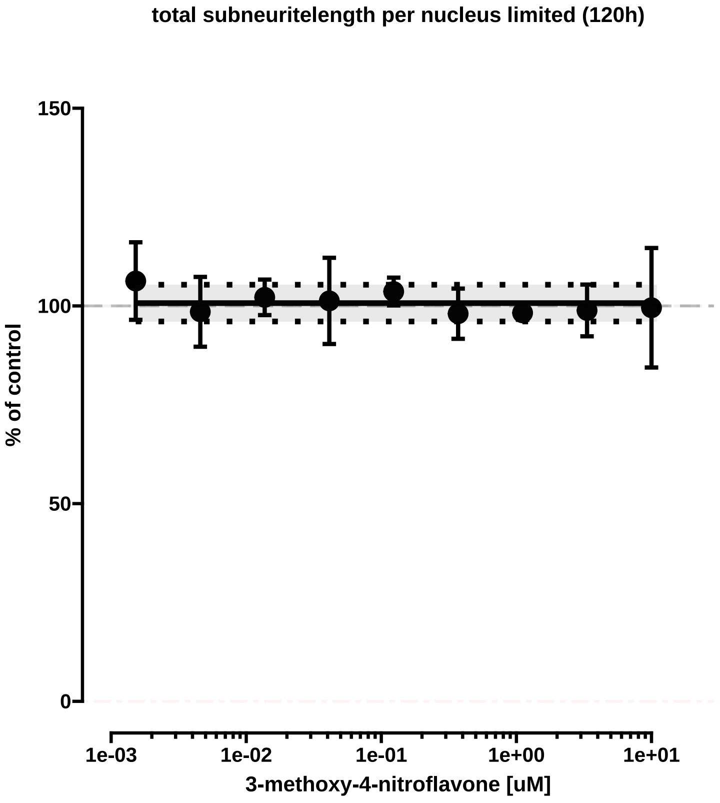

Model: 1-Parameter
Model abbr.: lm.1
Bechmark-Response (BMR): 20

BMCL: NA
BMC: NA
BMCU: NA

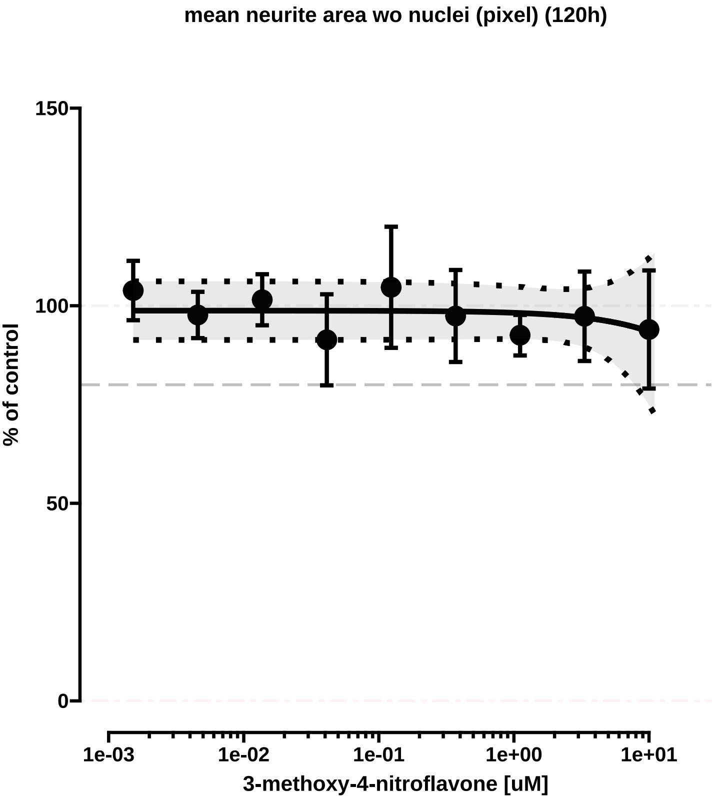

Model: Exponential decay with lower limit at 0
Model abbr.: EXD.2()
Bechmark-Response (BMR): 20

BMCL: NA
BMC: NA
BMCU: NA

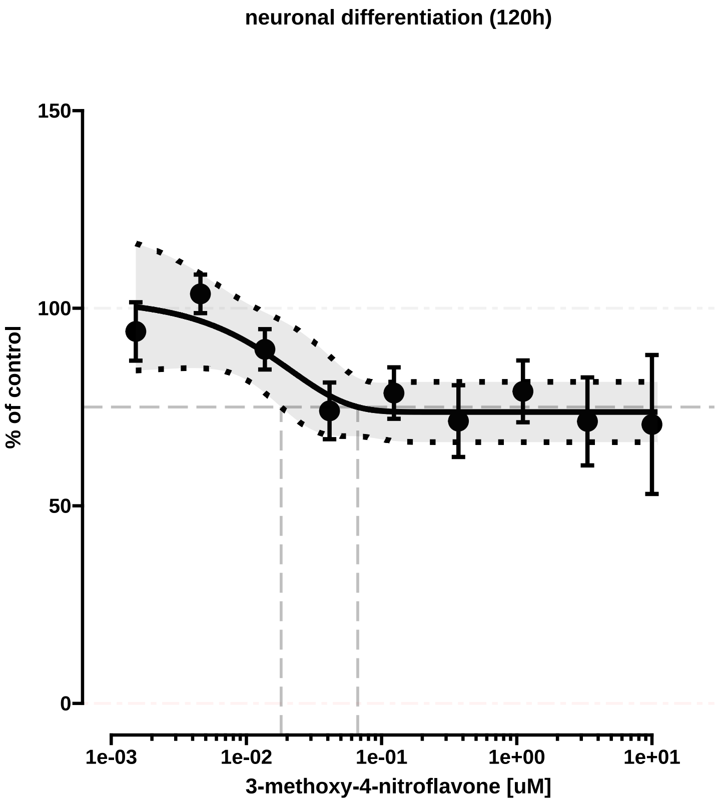

Model: Shifted exponential decay
Model abbr.: EXD.3()
Bechmark-Response (BMR): 25

BMCL: 0.018
BMC: 0.067
BMCU: NA

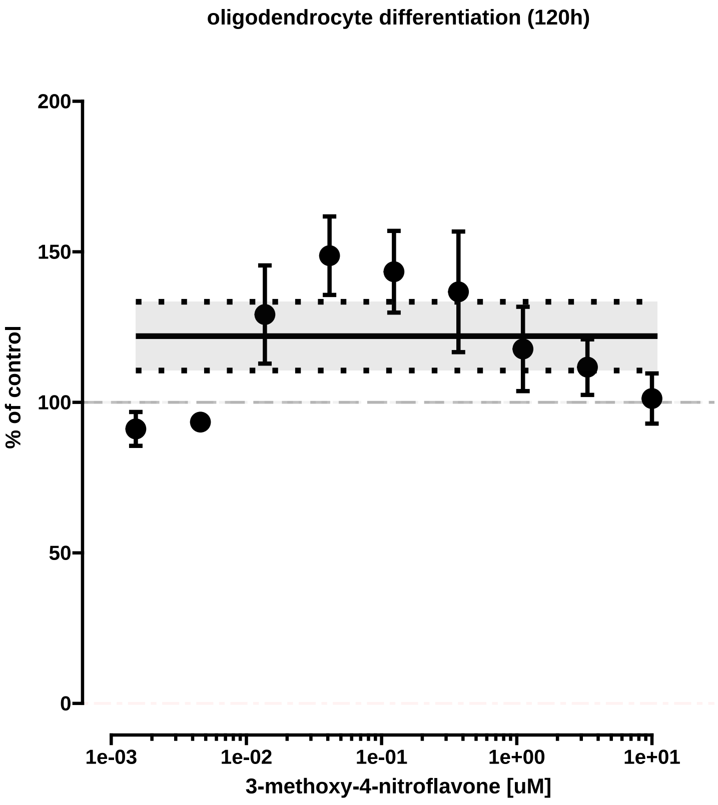

Model: 1-Parameter
Model abbr.: lm.1
Bechmark-Response (BMR): 25

BMCL: NA
BMC: NA
BMCU: NA

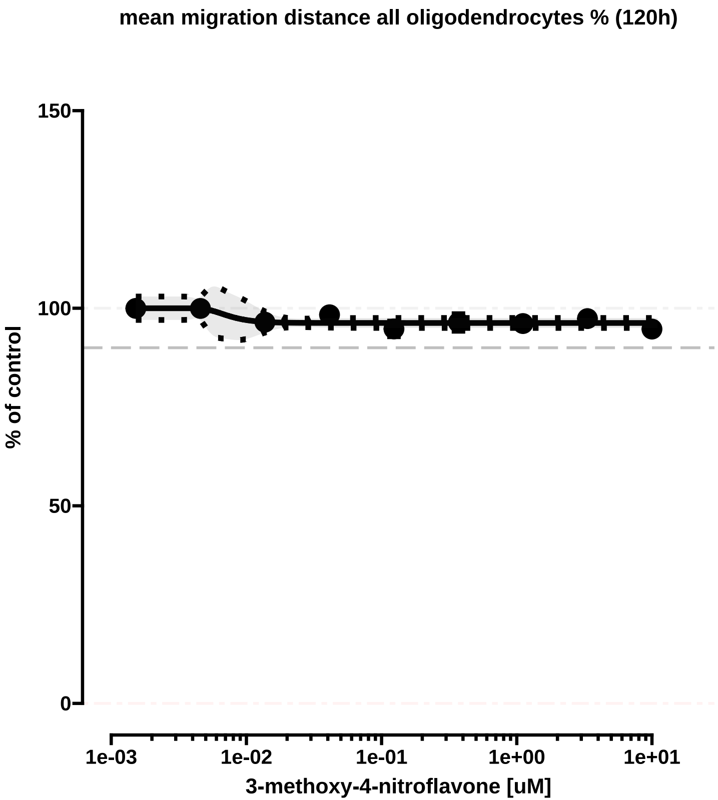

Model: Weibull (type 2)
Model abbr.: W2.4()
Bechmark-Response (BMR): 10

BMCL: NA
BMC: NA
BMCU: NA

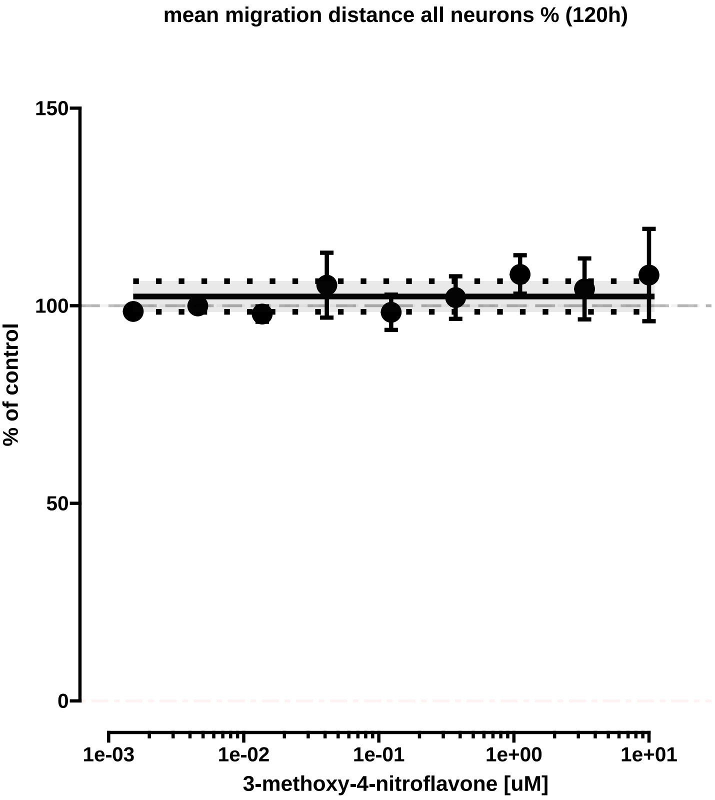

Model: 1-Parameter
Model abbr.: lm.1
Bechmark-Response (BMR): 10

BMCL: NA
BMC: NA
BMCU: NA

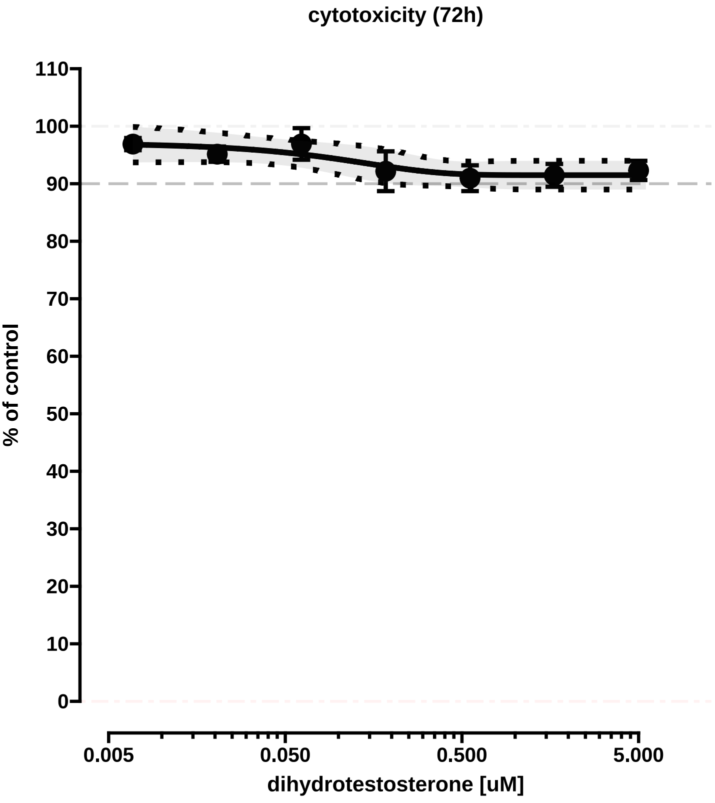

Model: Shifted exponential decay
Model abbr.: EXD.3()
Bechmark-Response (BMR): 10

BMCL: NA
BMC: NA
BMCU: NA

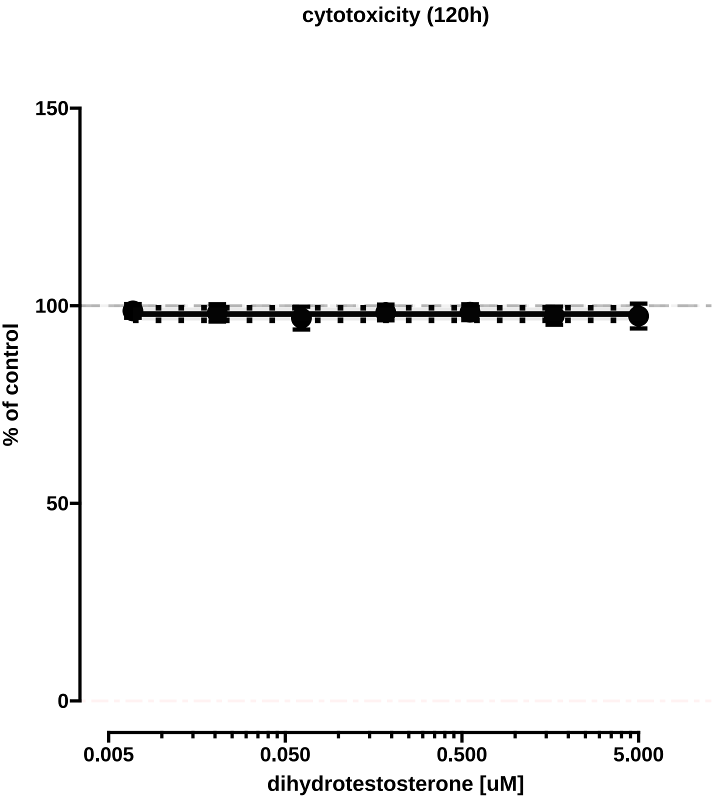

Model: 1-Parameter
Model abbr.: lm.1
Bechmark-Response (BMR): 10

BMCL: NA
BMC: NA
BMCU: NA

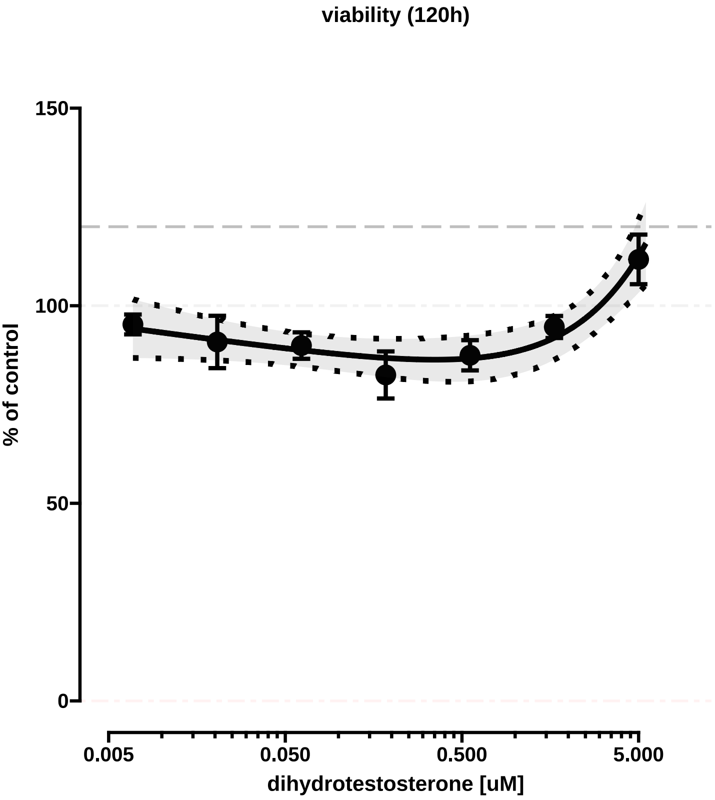

Model: Brain-Cousens (hormesis) with lower limit fixed at 0
Model abbr.: BC.4()
Bechmark-Response (BMR): 20

BMCL: NA
BMC: NA
BMCU: NA

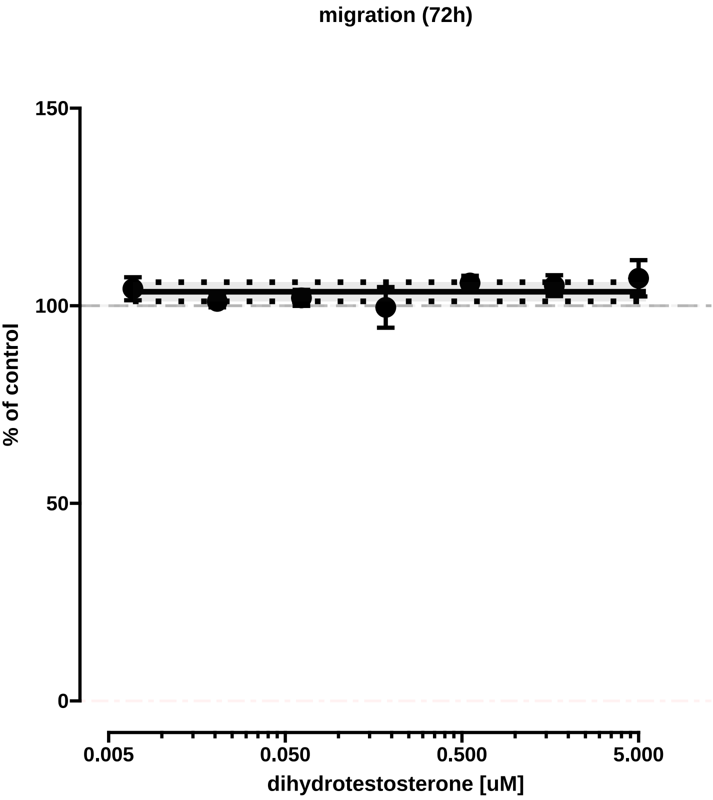

Model: 1-Parameter
Model abbr.: lm.1
Bechmark-Response (BMR): 10

BMCL: NA
BMC: NA
BMCU: NA

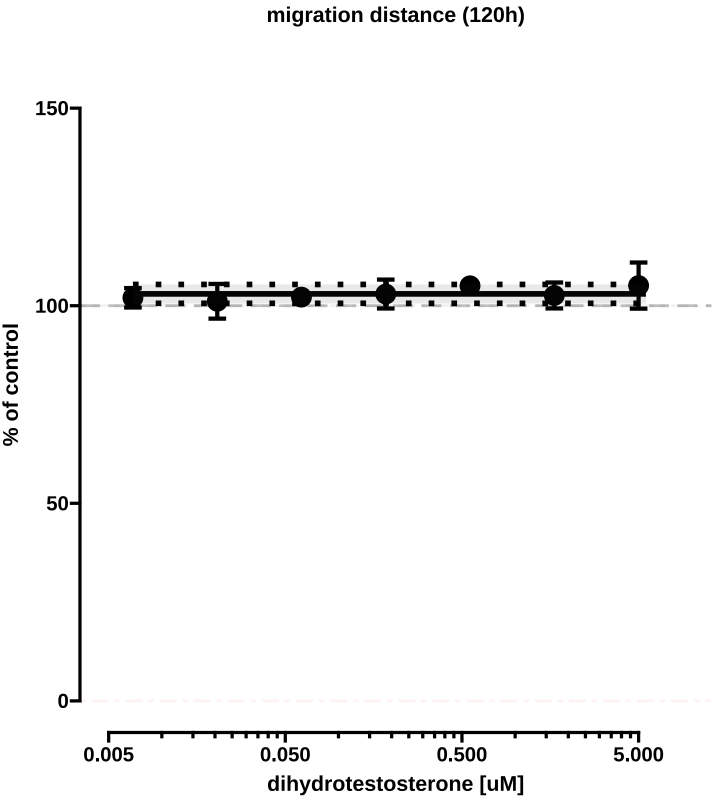

Model: 1-Parameter
Model abbr.: lm.1
Bechmark-Response (BMR): 10

BMCL: NA
BMC: NA
BMCU: NA

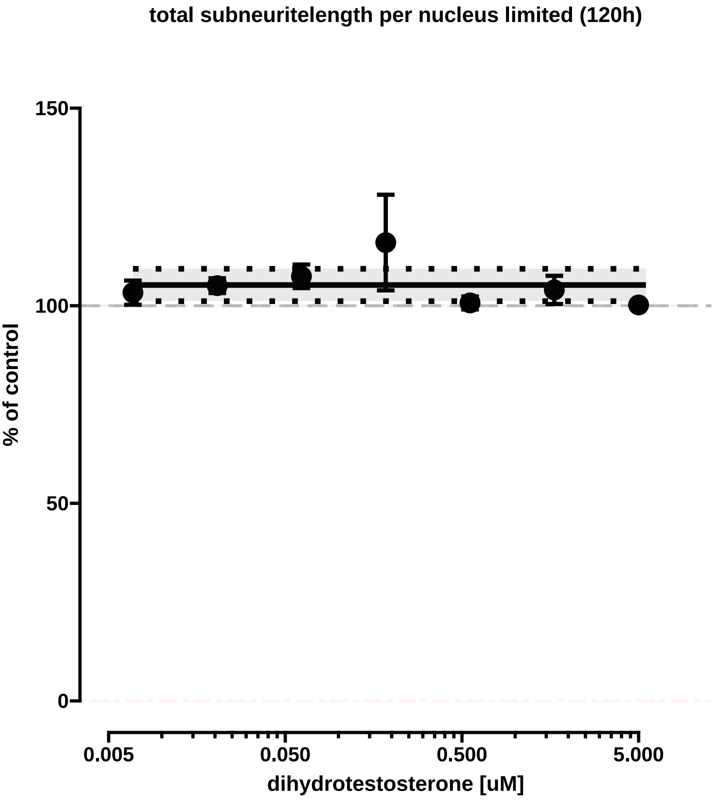

Model: 1-Parameter
Model abbr.: lm.1
Bechmark-Response (BMR): 20

BMCL: NA
BMC: NA
BMCU: NA

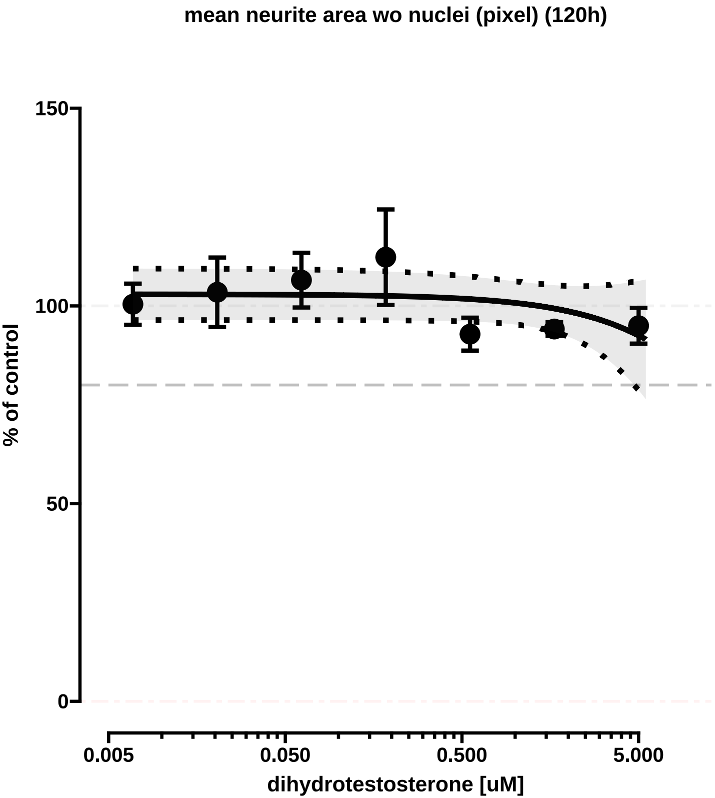

Model: Exponential decay with lower limit at 0
Model abbr.: EXD.2()
Bechmark-Response (BMR): 20

BMCL: NA
BMC: NA
BMCU: NA

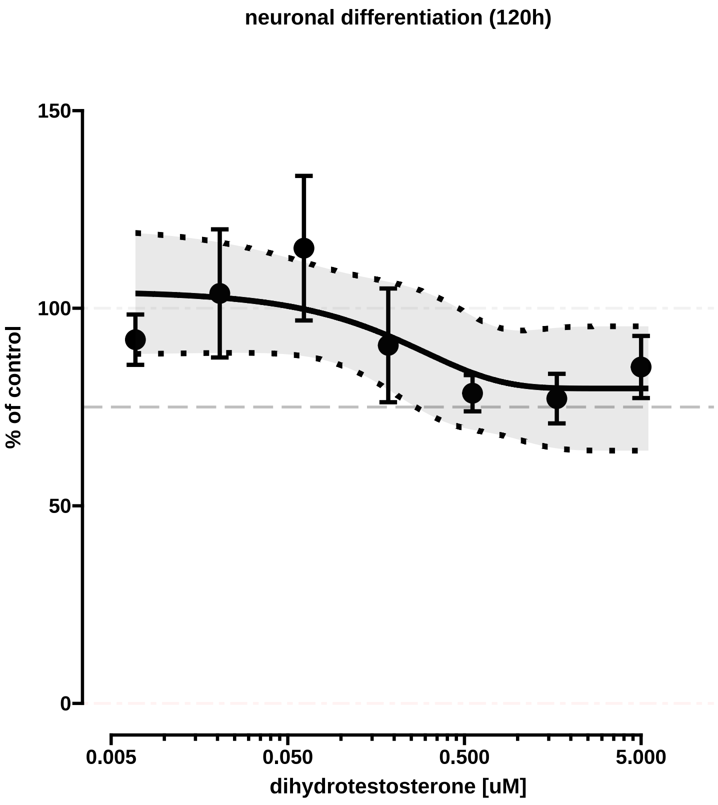

Model: Shifted exponential decay
Model abbr.: EXD.3()
Bechmark-Response (BMR): 25

BMCL: NA
BMC: NA
BMCU: NA

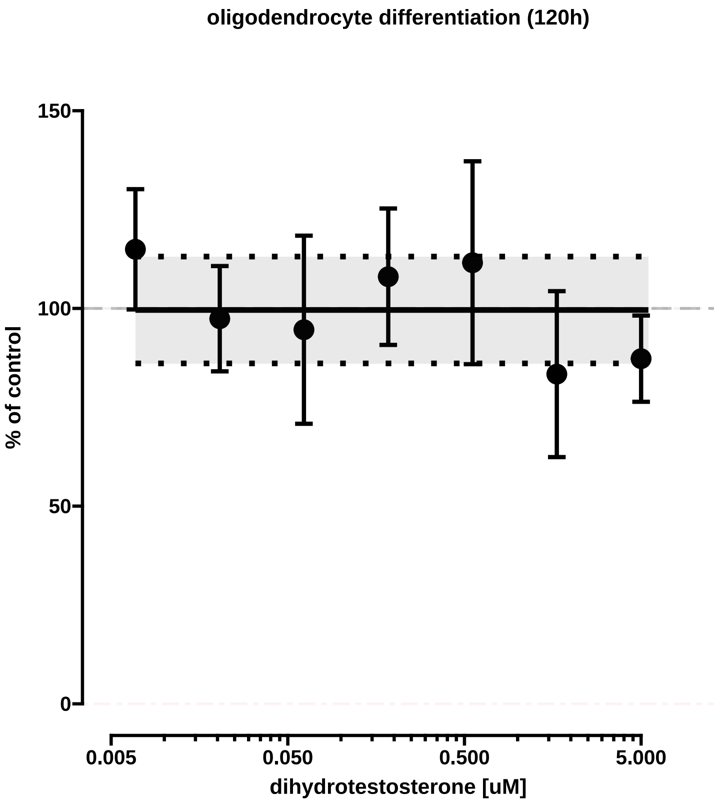

Model: 1-Parameter
Model abbr.: lm.1
Bechmark-Response (BMR): 25

BMCL: NA
BMC: NA
BMCU: NA

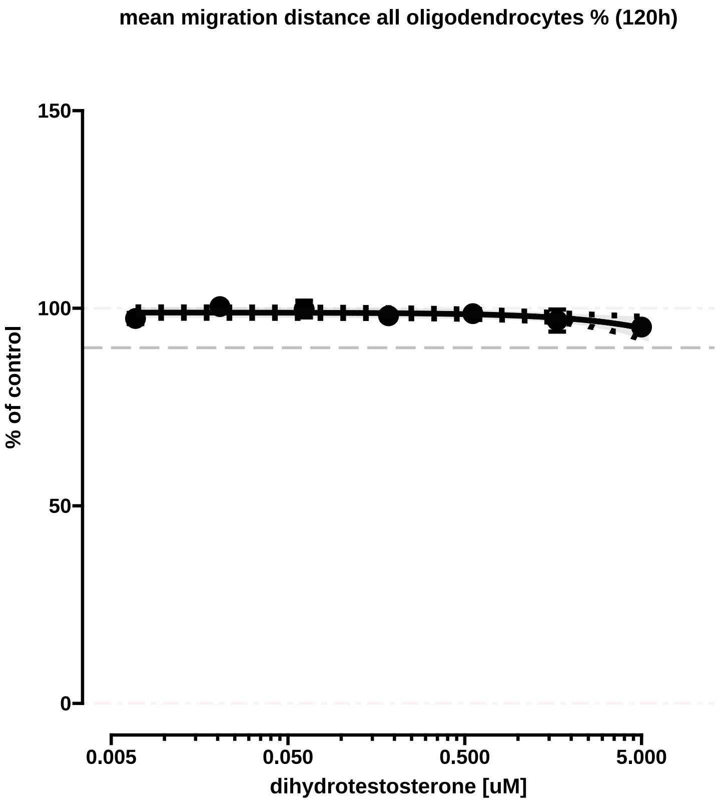

Model: Exponential decay with lower limit at 0
Model abbr.: EXD.2()
Bechmark-Response (BMR): 10

BMCL: NA
BMC: NA
BMCU: NA

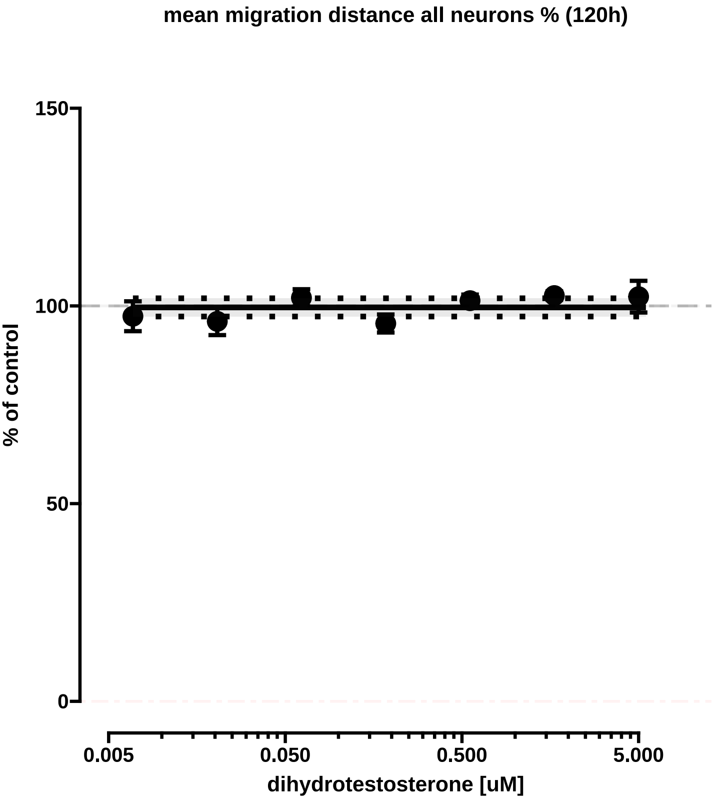

Model: 1-Parameter
Model abbr.: lm.1
Bechmark-Response (BMR): 10

BMCL: NA
BMC: NA
BMCU: NA

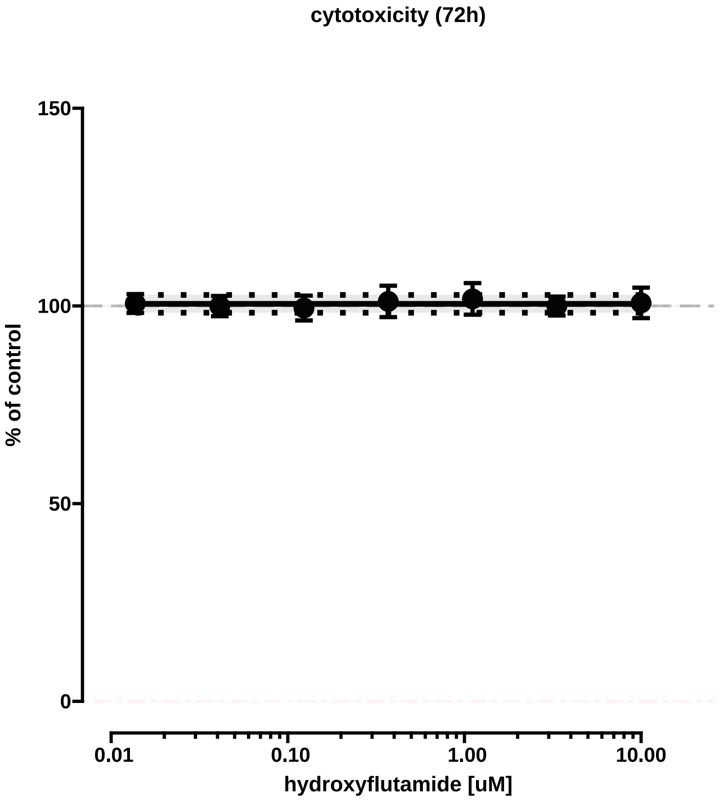

Model: 1-Parameter
Model abbr.: lm.1
Bechmark-Response (BMR): 10

BMCL: NA
BMC: NA
BMCU: NA

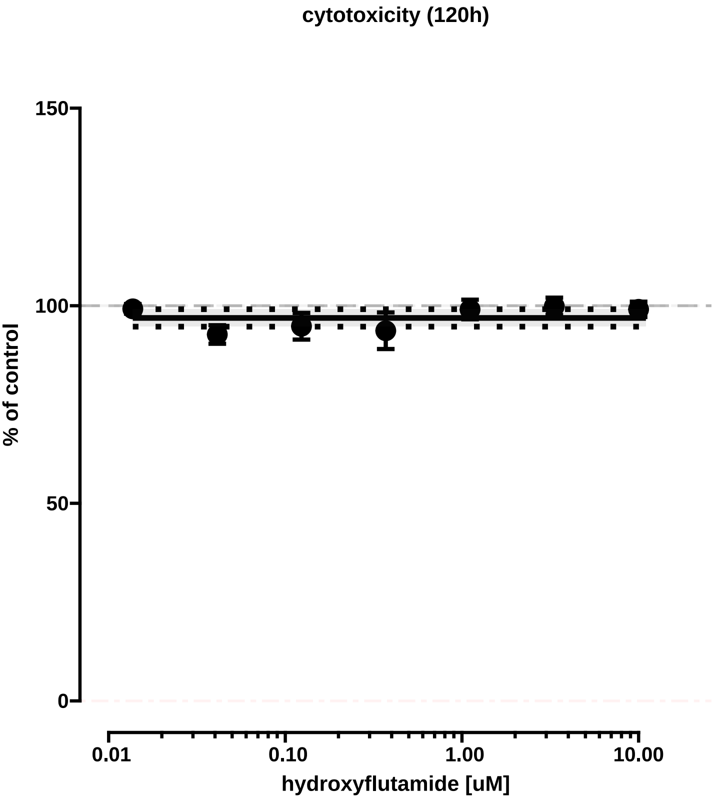

Model: 1-Parameter
Model abbr.: lm.1
Bechmark-Response (BMR): 10

BMCL: NA
BMC: NA
BMCU: NA

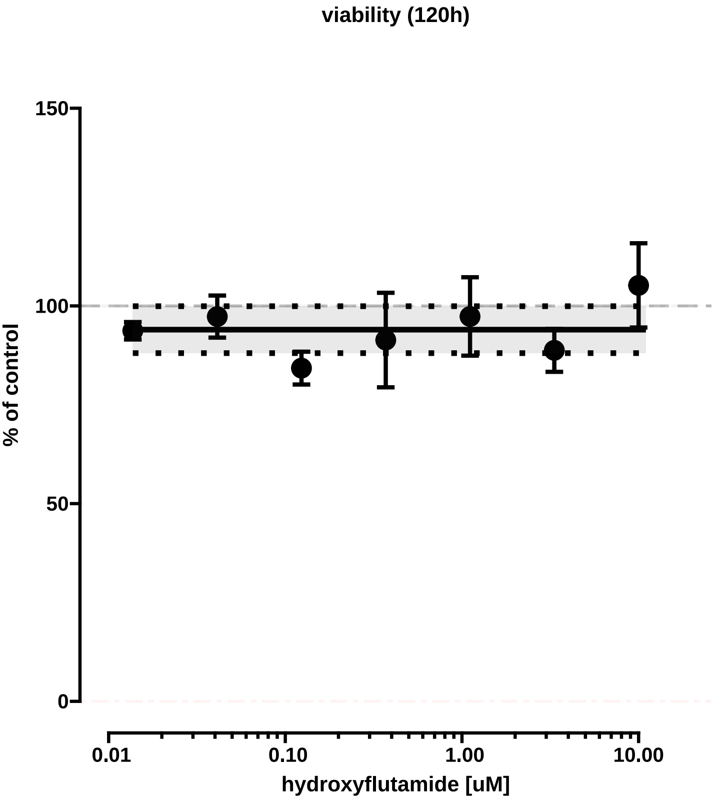

Model: 1-Parameter
Model abbr.: lm.1
Bechmark-Response (BMR): 20

BMCL: NA
BMC: NA
BMCU: NA

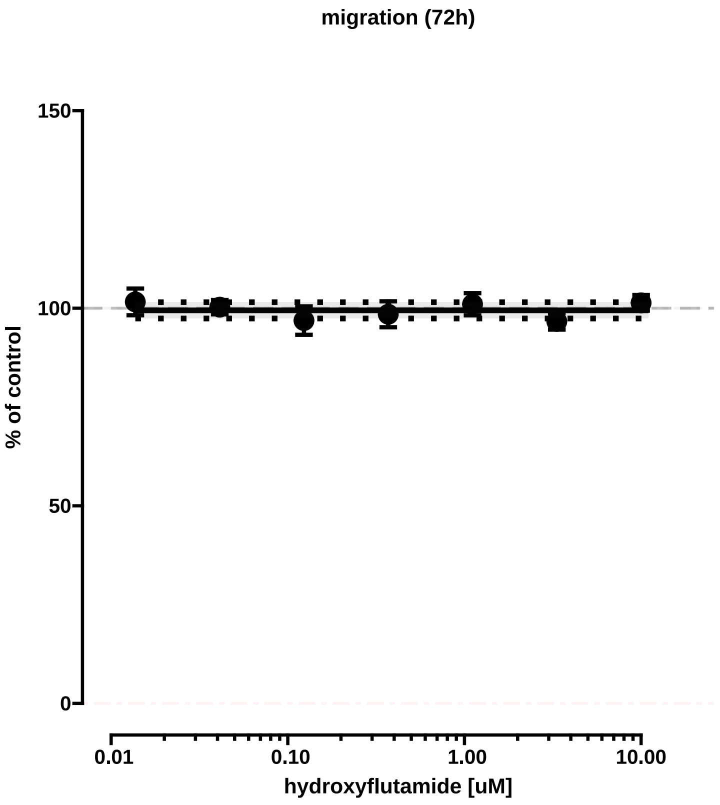

Model: 1-Parameter
Model abbr.: lm.1
Bechmark-Response (BMR): 10

BMCL: NA
BMC: NA
BMCU: NA

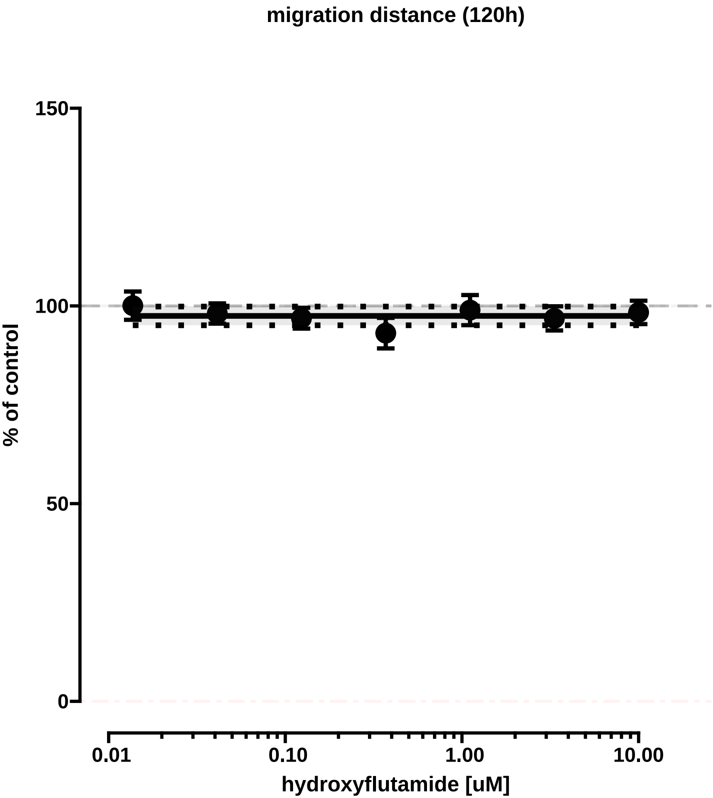

Model: 1-Parameter
Model abbr.: lm.1
Bechmark-Response (BMR): 10

BMCL: NA
BMC: NA
BMCU: NA

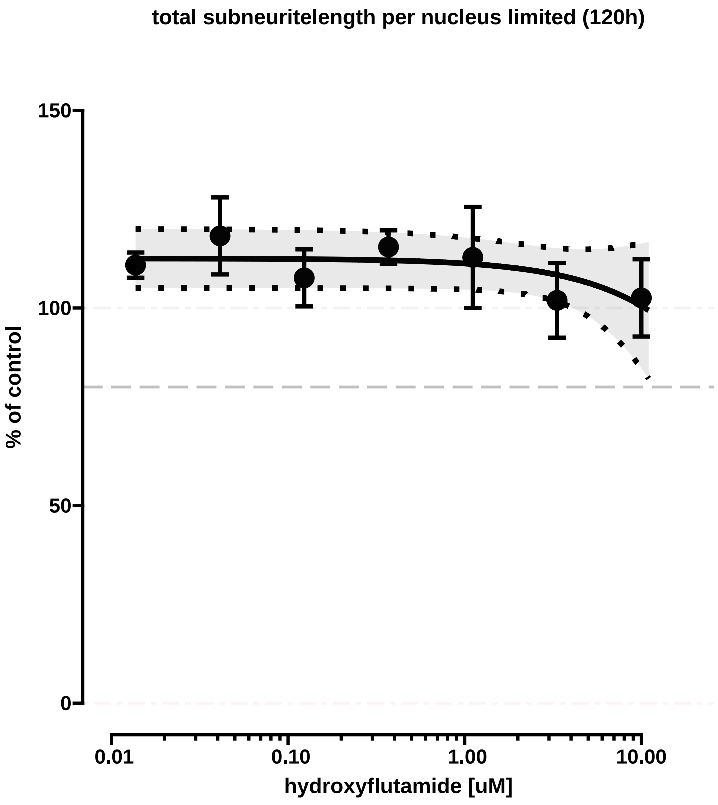

Model: Exponential decay with lower limit at 0
Model abbr.: EXD.2()
Bechmark-Response (BMR): 20

BMCL: NA
BMC: NA
BMCU: NA

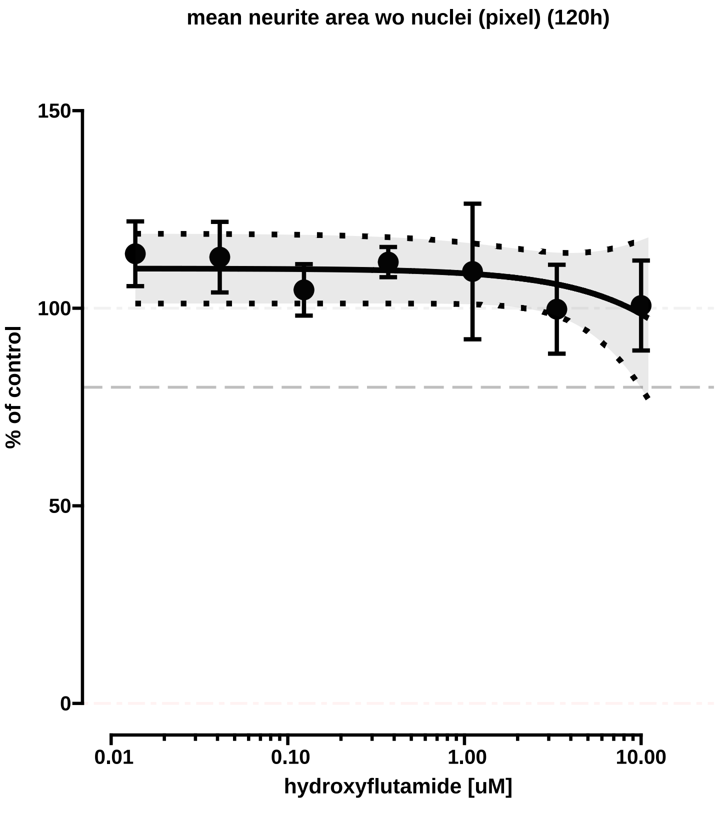

Model: Exponential decay with lower limit at 0
Model abbr.: EXD.2()
Bechmark-Response (BMR): 20

BMCL: NA
BMC: NA
BMCU: NA

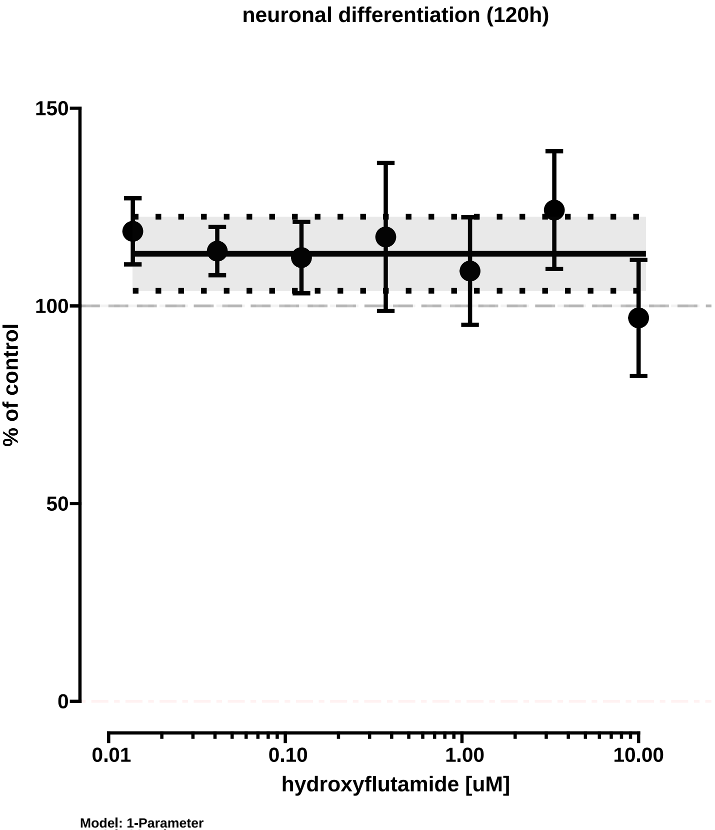

Model: 1-Parameter
Model abbr.: lm.1
Bechmark-Response (BMR): 25

BMCL: NA
BMC: NA
BMCU: NA

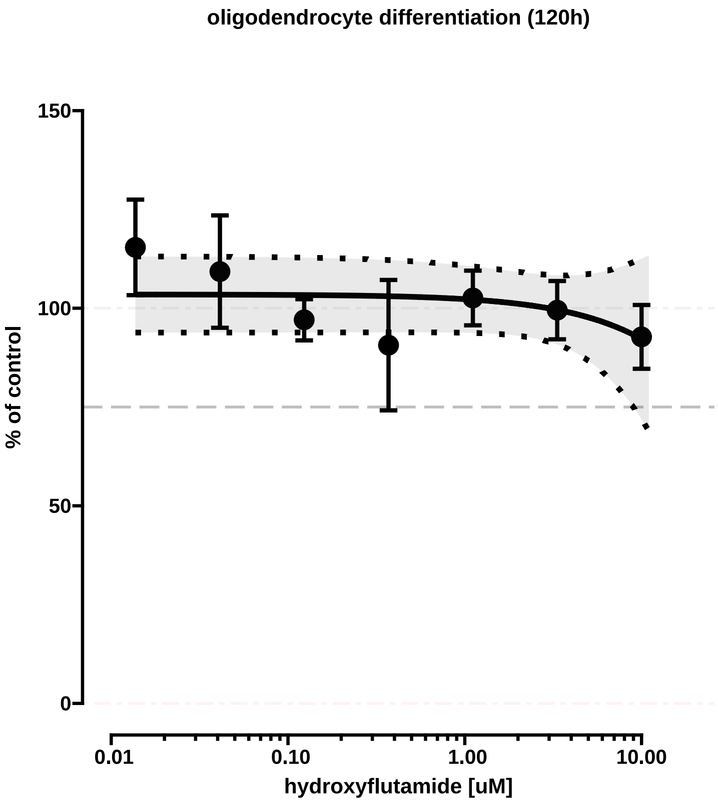

Model: Exponential decay with lower limit at 0
Model abbr.: EXD.2()
Bechmark-Response (BMR): 25

BMCL: NA
BMC: NA
BMCU: NA

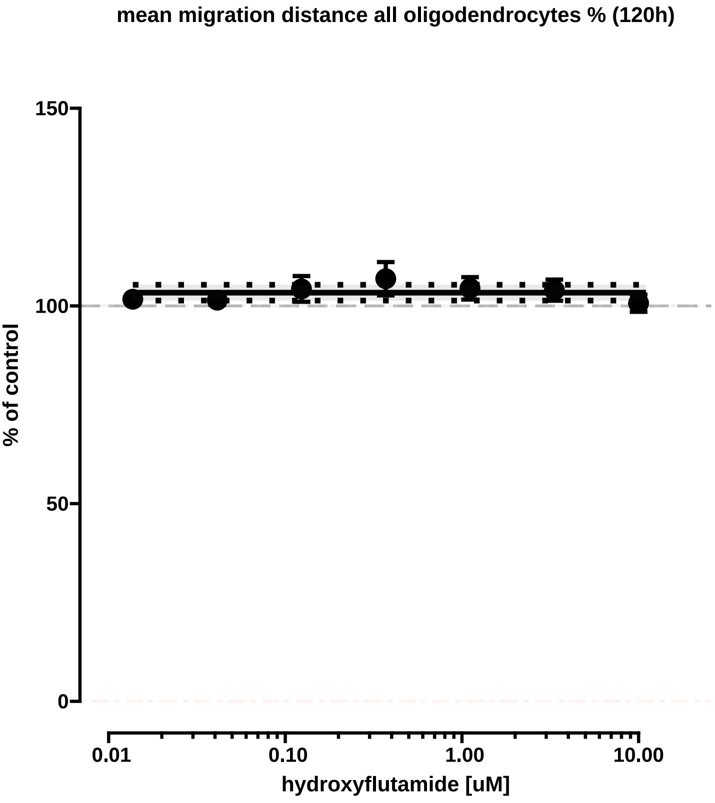

Model: 1-Parameter
Model abbr.: lm.1
Bechmark-Response (BMR): 10

BMCL: NA
BMC: NA
BMCU: NA

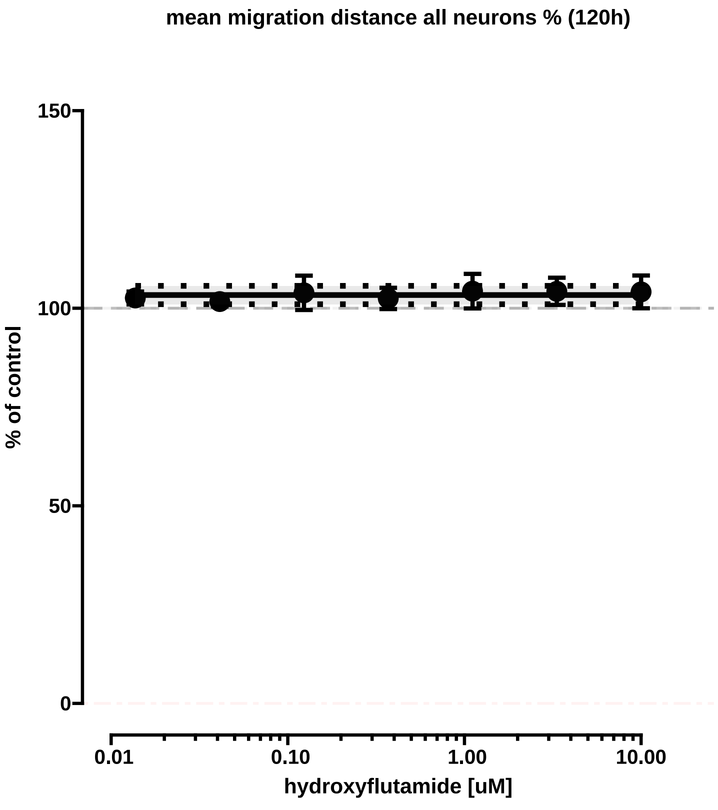

Model: 1-Parameter
Model abbr.: lm.1
Bechmark-Response (BMR): 10

BMCL: NA
BMC: NA
BMCU: NA

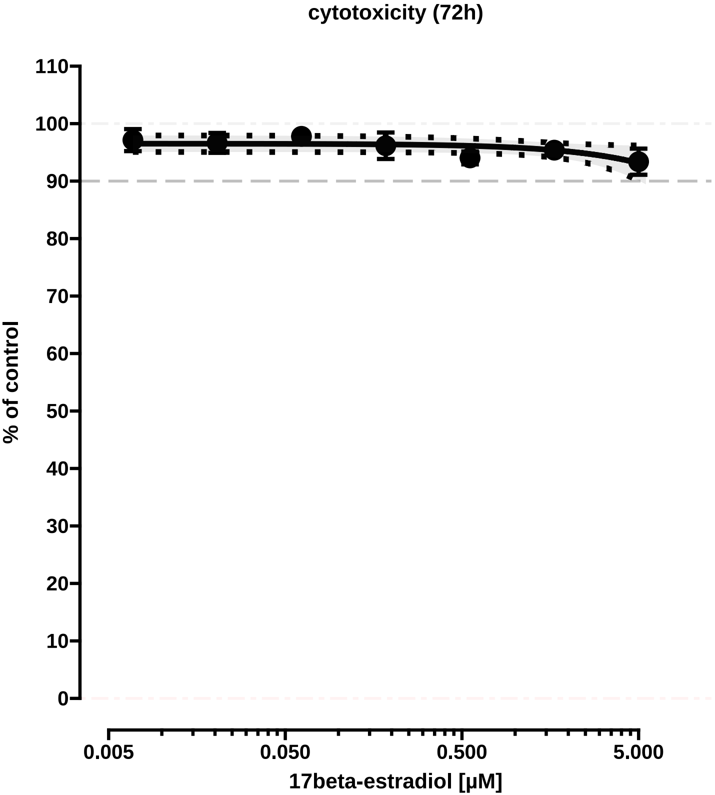

Model: Exponential decay with lower limit at 0
Model abbr.: EXD.2()
Bechmark-Response (BMR): 10

BMCL: NA
BMC: NA
BMCU: NA

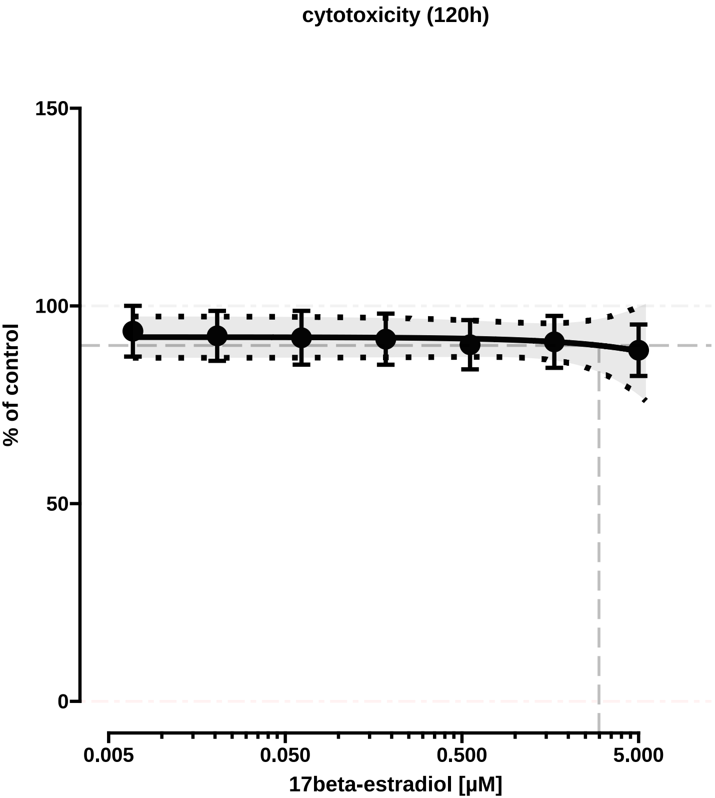

Model: Exponential decay with lower limit at 0
Model abbr.: EXD.2()
Bechmark-Response (BMR): 10

BMCL: NA
BMC: 2.983
BMCU: NA

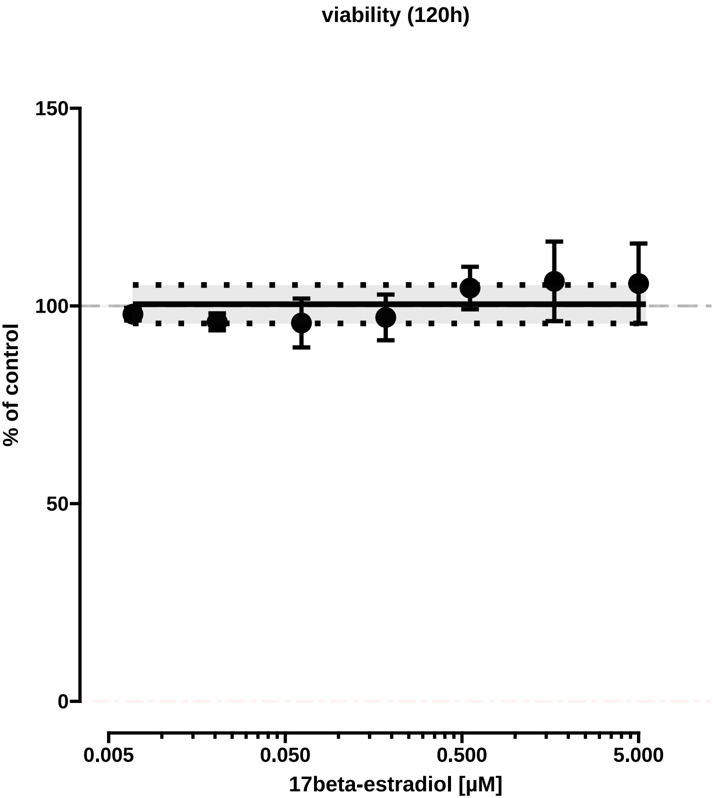

Model: 1-Parameter
Model abbr.: lm.1
Bechmark-Response (BMR): 20

BMCL: NA
BMC: NA
BMCU: NA

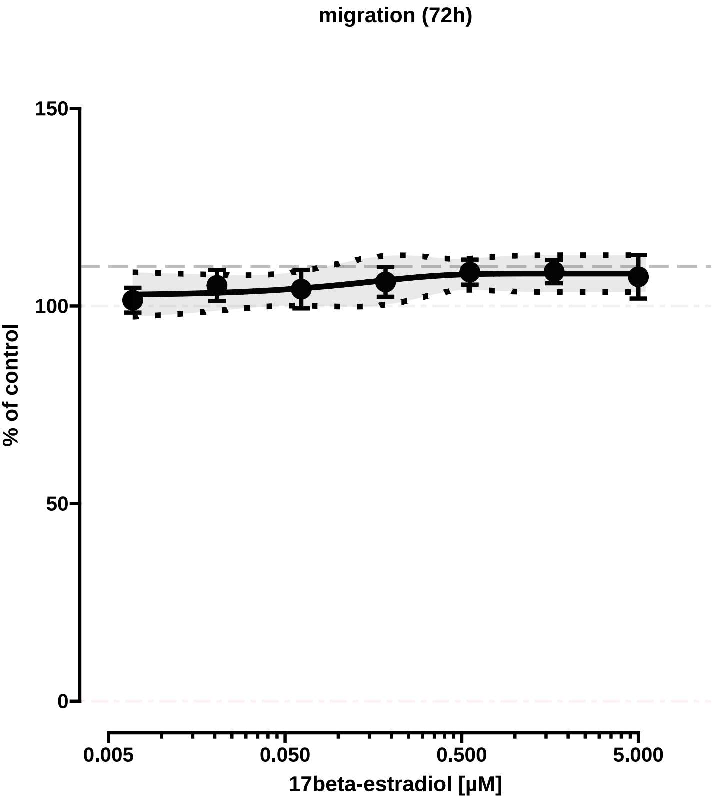

Model: Shifted exponential decay
Model abbr.: EXD.3()
Bechmark-Response (BMR): 10

BMCL: NA
BMC: NA
BMCU: NA

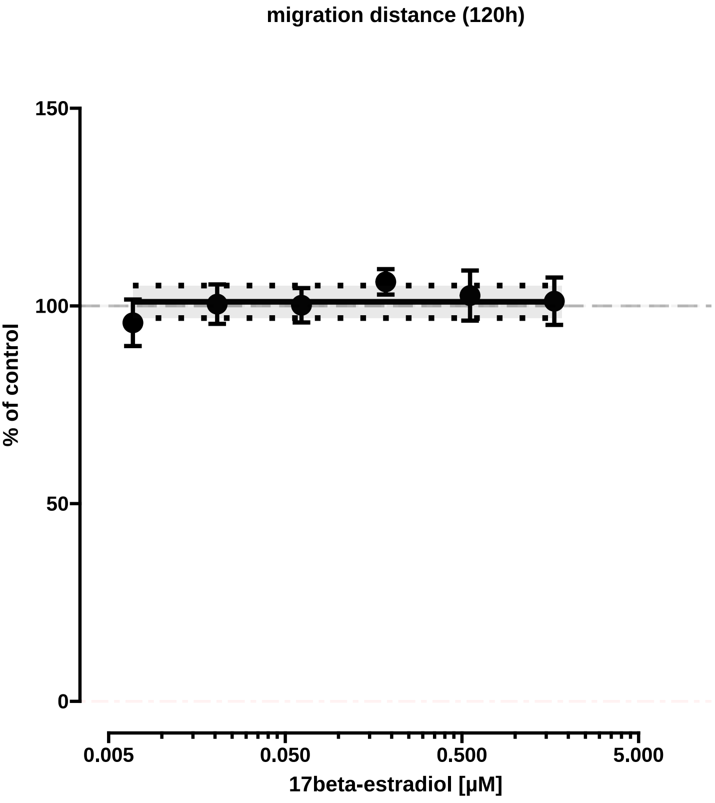

Model: 1-Parameter
Model abbr.: lm.1
Bechmark-Response (BMR): 10

BMCL: NA
BMC: NA
BMCU: NA

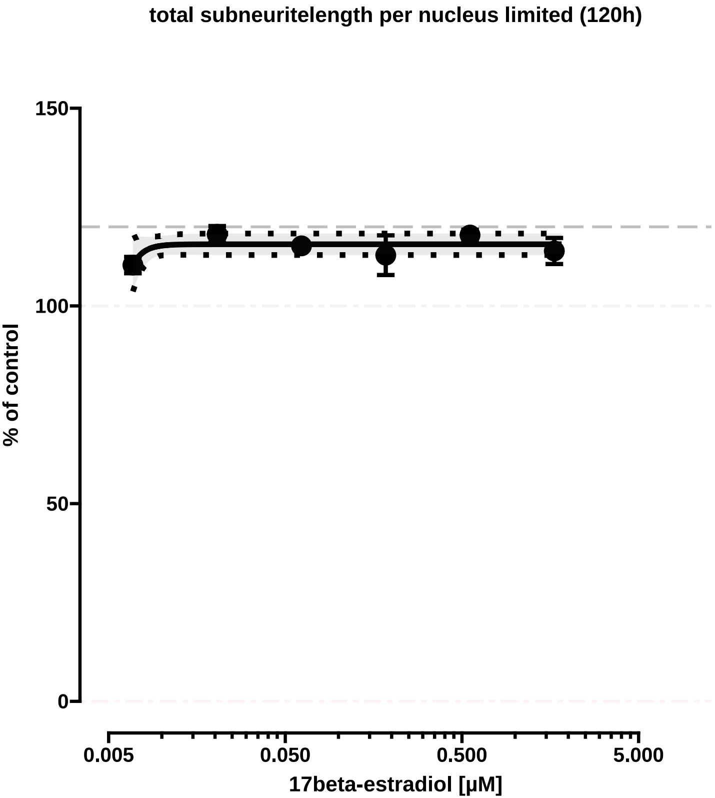

Model: Weibull (type 1) with lower limit at 0
Model abbr.: W1.3()
Bechmark-Response (BMR): 20

BMCL: NA
BMC: NA
BMCU: NA

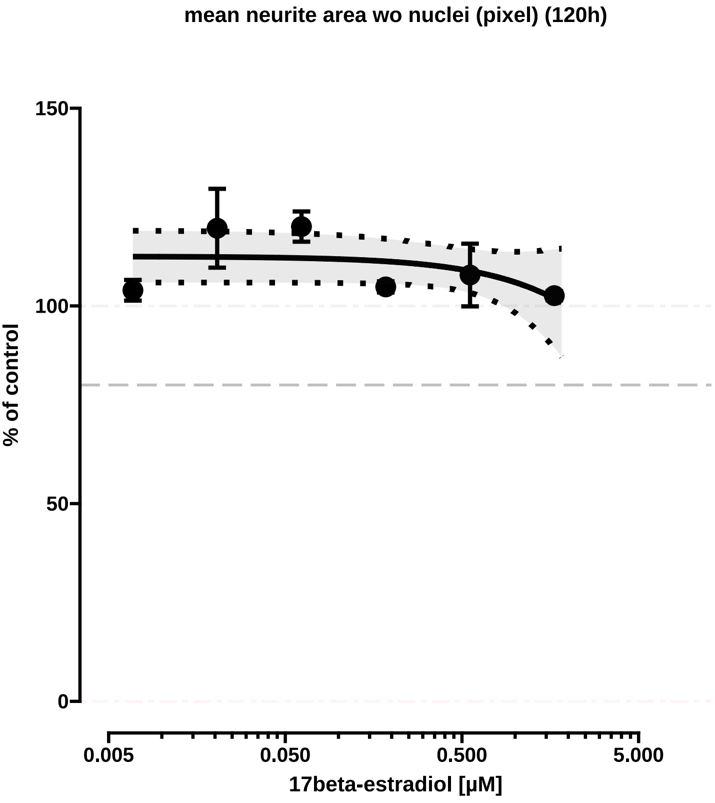

Model: Exponential decay with lower limit at 0
Model abbr.: EXD.2()
Bechmark-Response (BMR): 20

BMCL: NA
BMC: NA
BMCU: NA

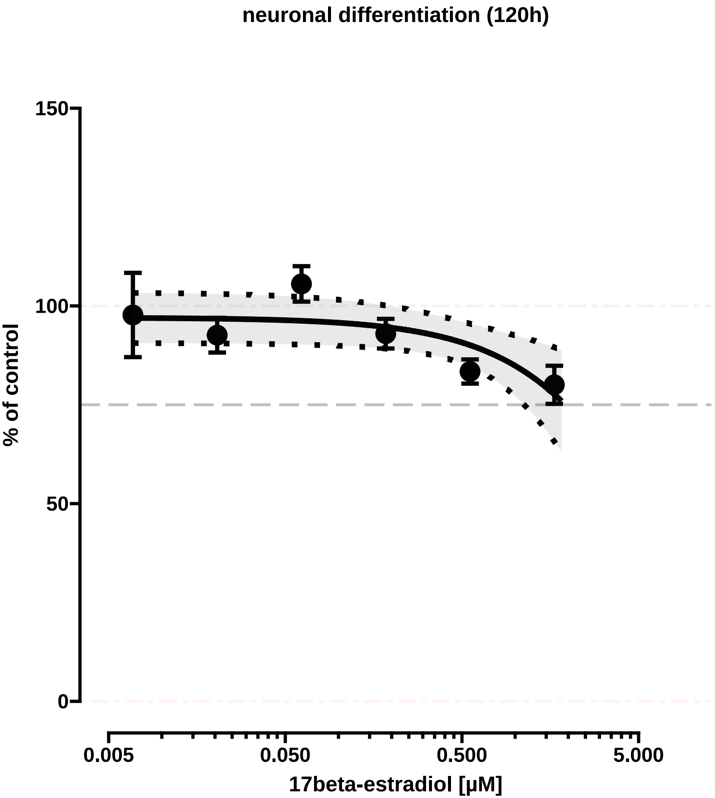

Model: Exponential decay with lower limit at 0
Model abbr.: EXD.2()
Bechmark-Response (BMR): 25

BMCL: NA
BMC: NA
BMCU: NA

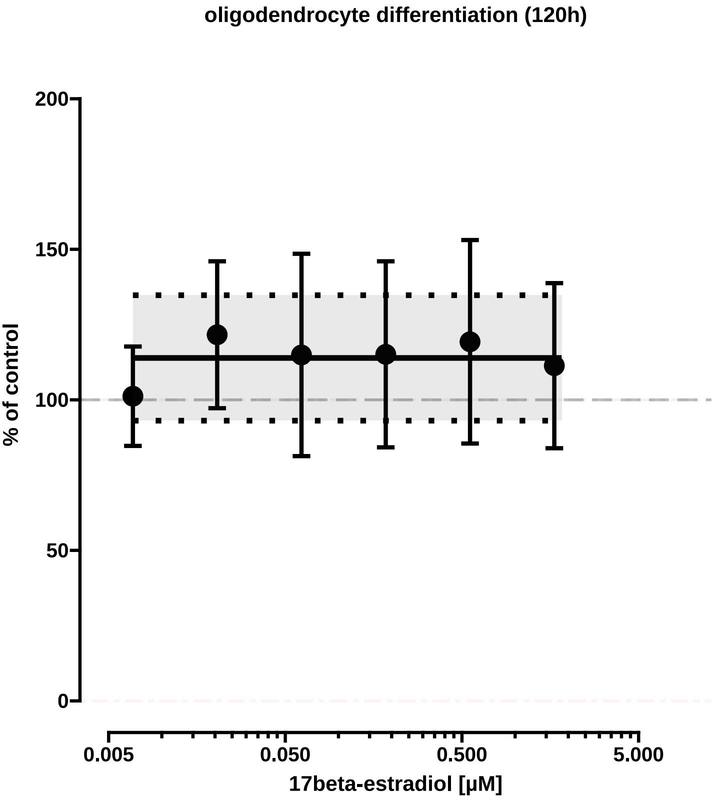

Model: 1-Parameter
Model abbr.: lm.1
Bechmark-Response (BMR): 25

BMCL: NA
BMC: NA
BMCU: NA

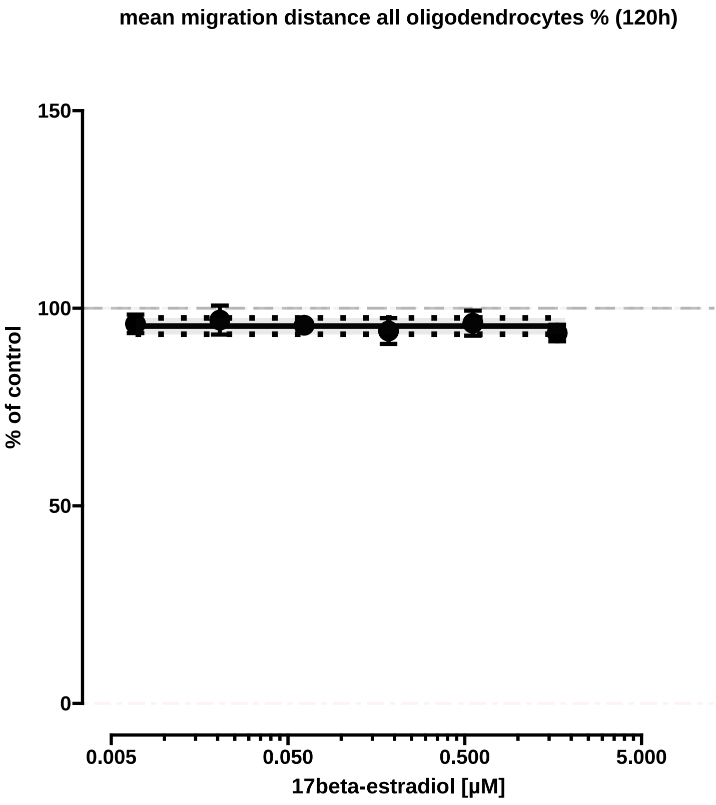

Model: 1-Parameter
Model abbr.: lm.1
Bechmark-Response (BMR): 10

BMCL: NA
BMC: NA
BMCU: NA

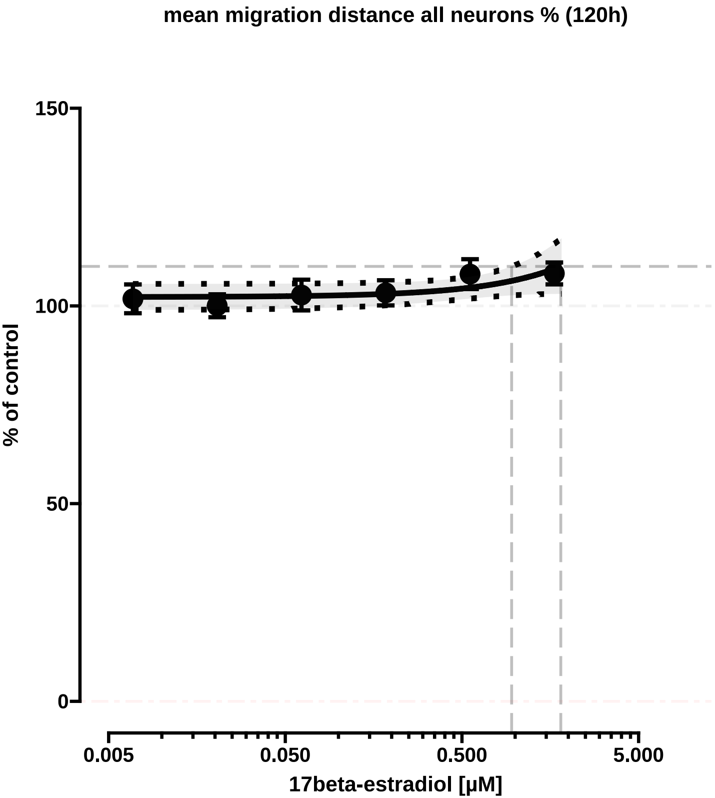

Model: Linear
Model abbr.: lm
Bechmark-Response (BMR): 10

BMCL: 0.956
BMC: 1.814
BMCU: NA

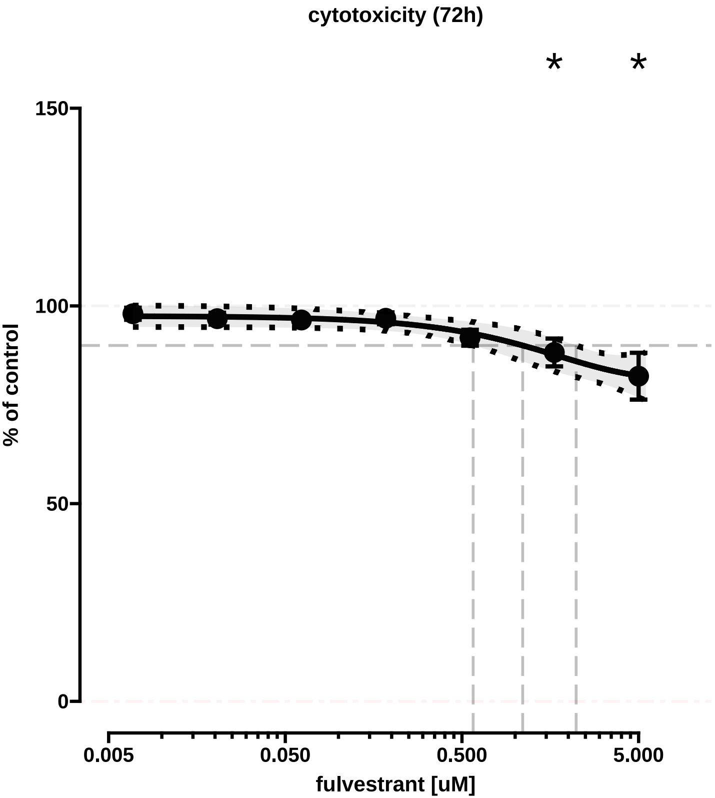

Model: Shifted exponential decay
Model abbr.: EXD.3()
Bechmark-Response (BMR): 10

BMCL: 0.579
BMC: 1.103
BMCU: 2.215

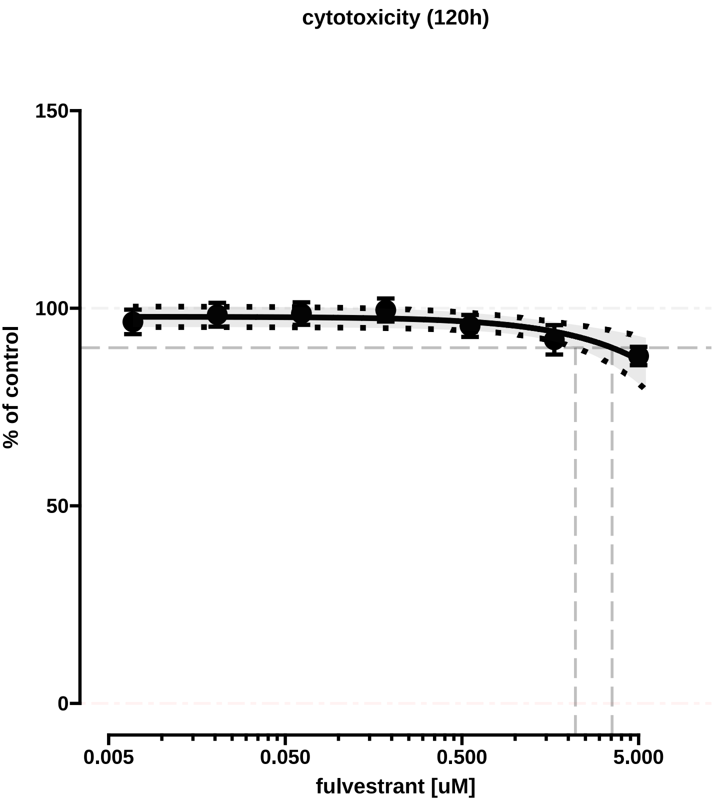

Model: Exponential decay with lower limit at 0
Model abbr.: EXD.2()
Bechmark-Response (BMR): 10

BMCL: 2.194
BMC: 3.54
BMCU: NA

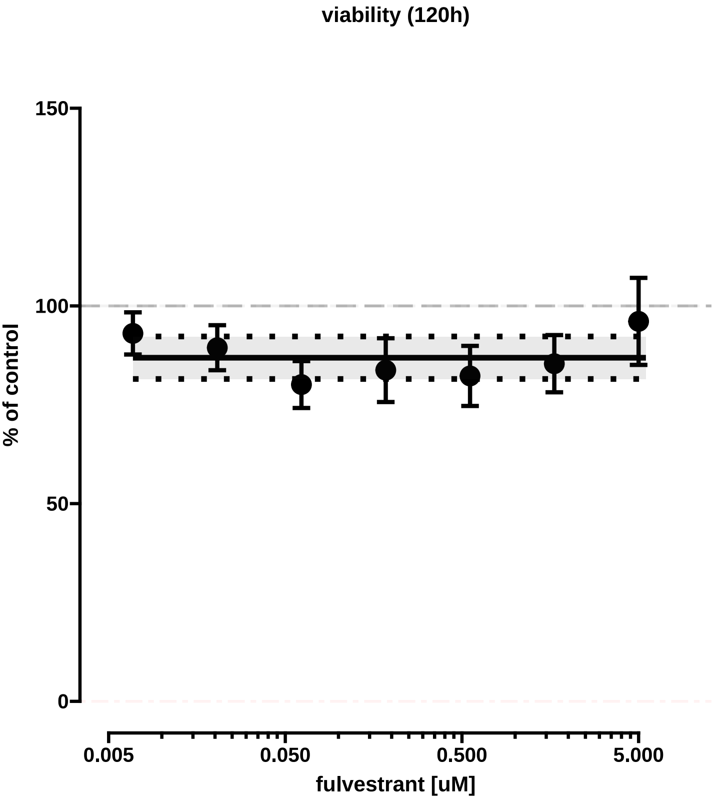

Model: 1-Parameter
Model abbr.: lm.1
Bechmark-Response (BMR): 20

BMCL: NA
BMC: NA
BMCU: NA

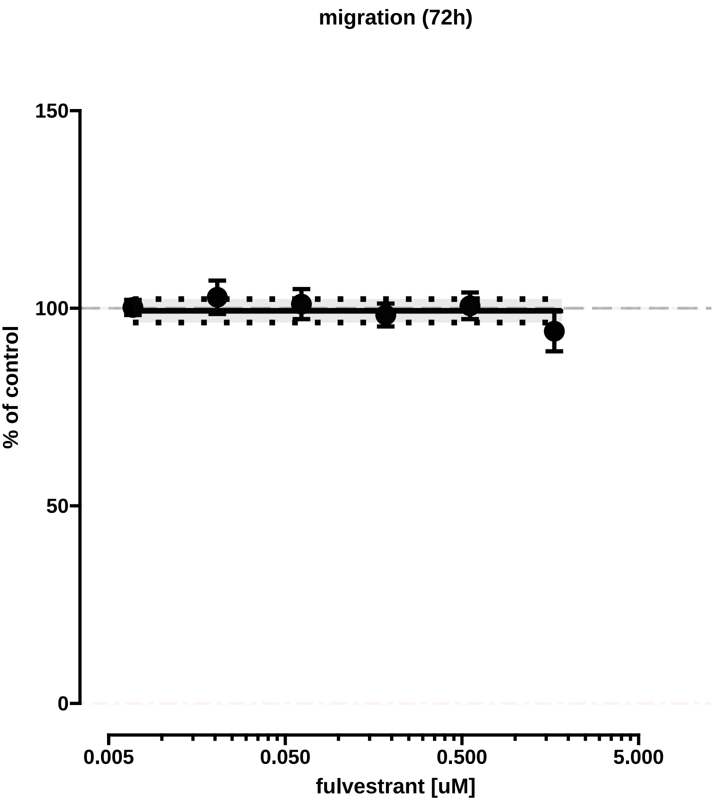

Model: 1-Parameter
Model abbr.: lm.1
Bechmark-Response (BMR): 10

BMCL: NA
BMC: NA
BMCU: NA

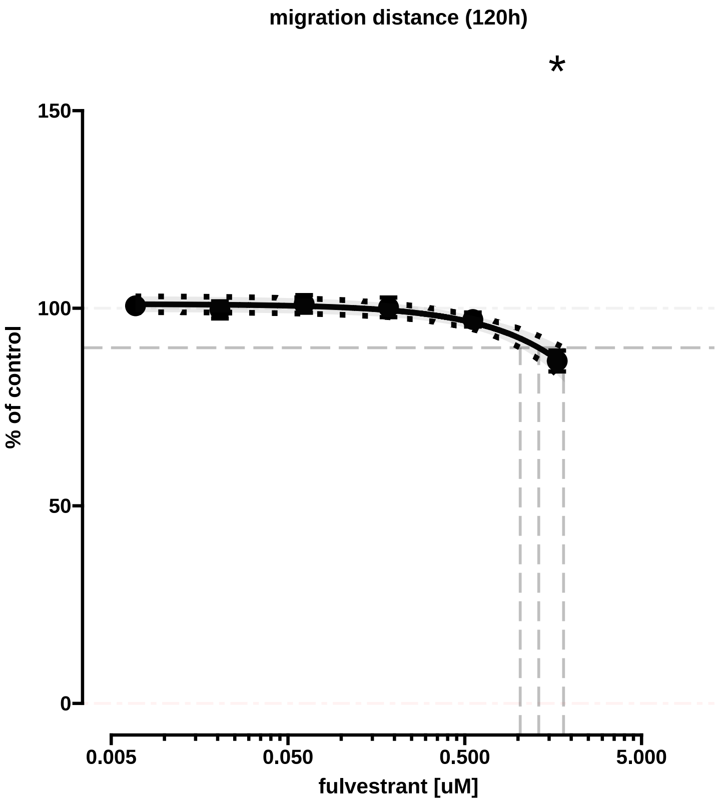

Model: Linear
Model abbr.: lm
Bechmark-Response (BMR): 10

BMCL: 1.029
BMC: 1.31
BMCU: 1.81

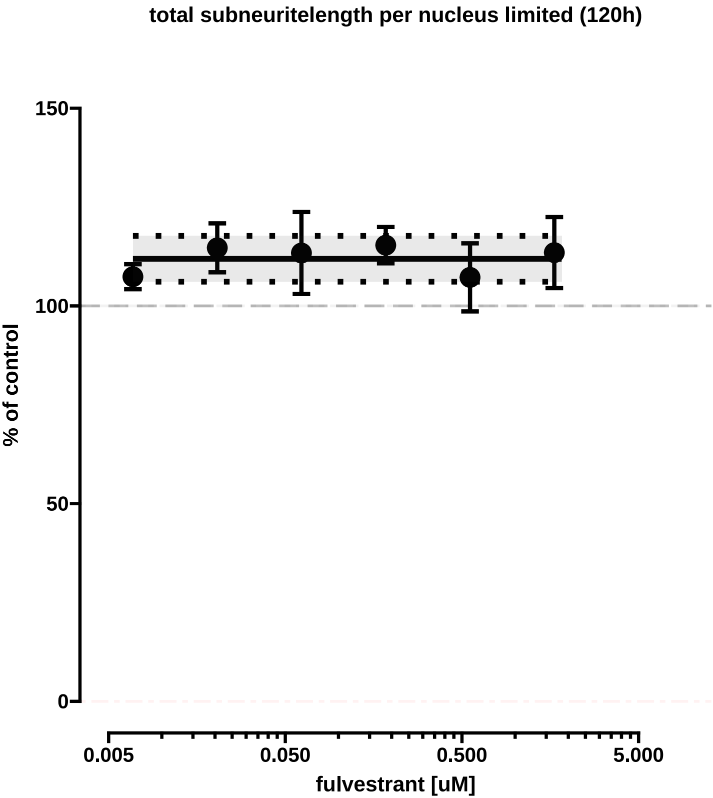

Model: 1-Parameter
Model abbr.: lm.1
Bechmark-Response (BMR): 20

BMCL: NA
BMC: NA
BMCU: NA

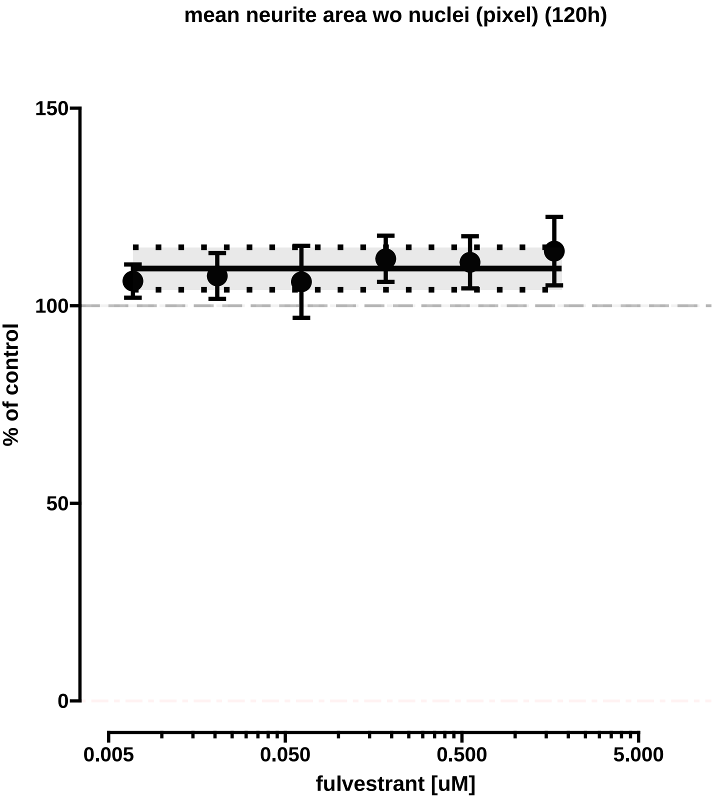

Model: 1-Parameter
Model abbr.: lm.1
Bechmark-Response (BMR): 20

BMCL: NA
BMC: NA
BMCU: NA

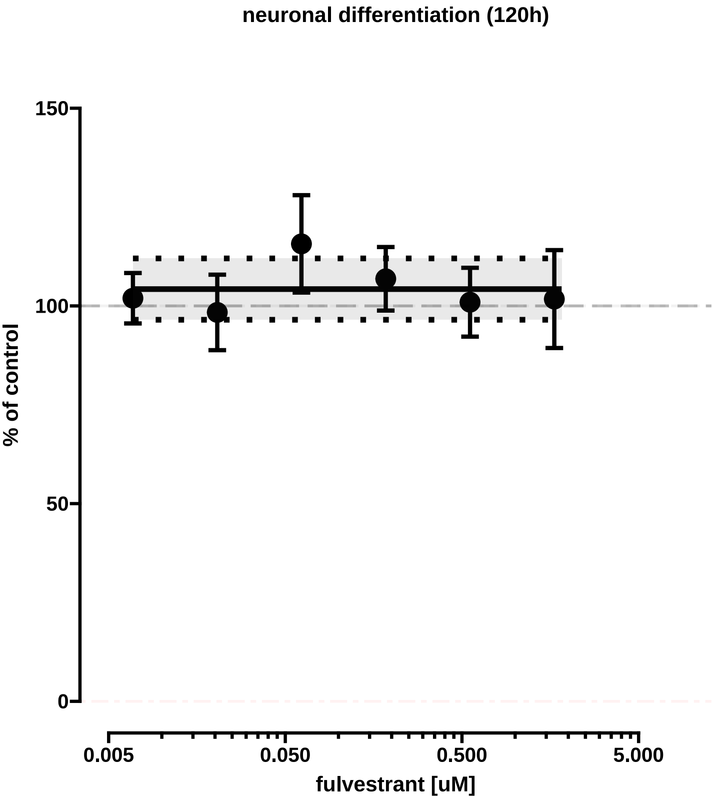

Model: 1-Parameter
Model abbr.: lm.1
Bechmark-Response (BMR): 25

BMCL: NA
BMC: NA
BMCU: NA

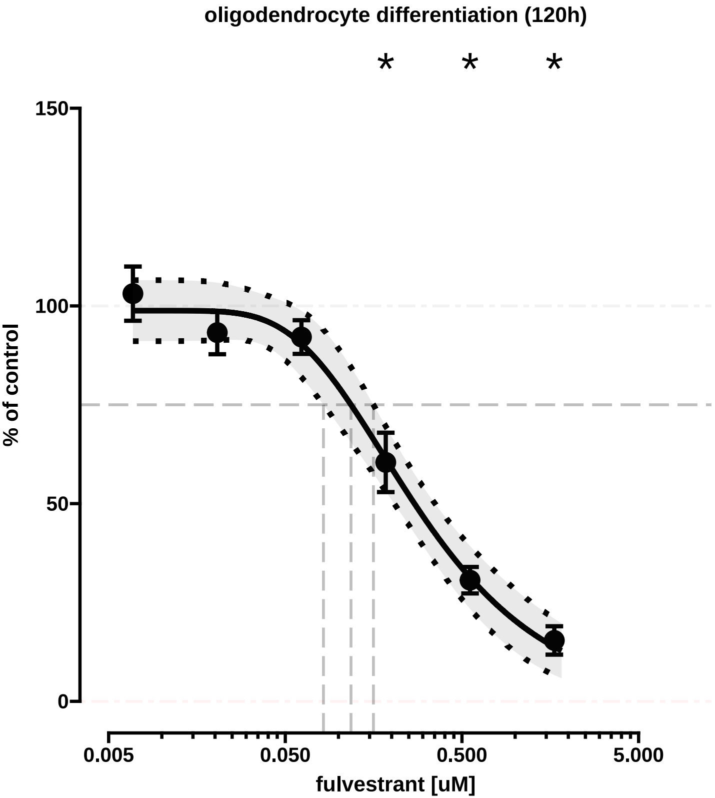

Model: Weibull (type 2) with lower limit at 0
Model abbr.: W2.3()
Bechmark-Response (BMR): 25

BMCL: 0.082
BMC: 0.118
BMCU: 0.158

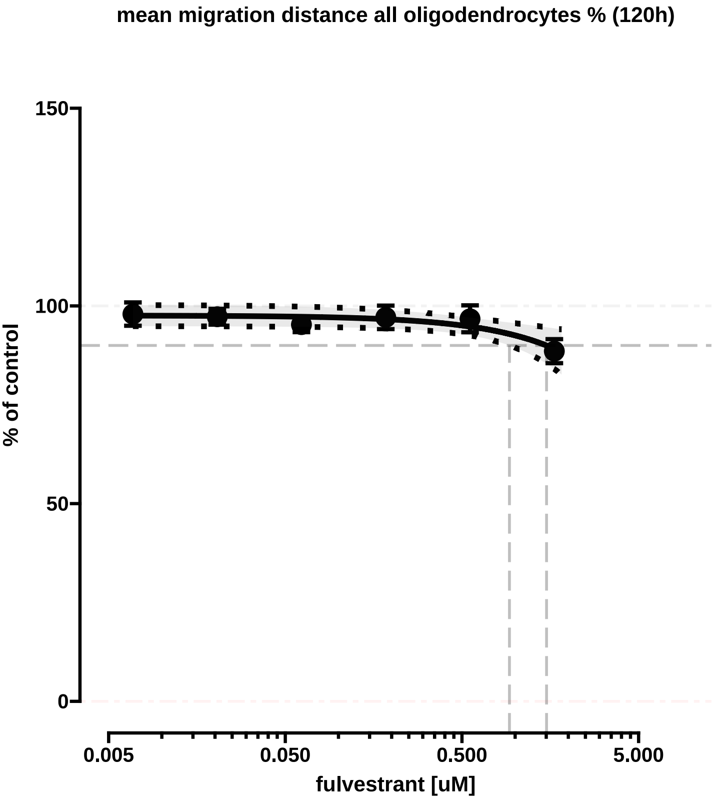

Model: Linear
Model abbr.: lm
Bechmark-Response (BMR): 10

BMCL: 0.929
BMC: 1.506
BMCU: NA

mean migration distance all neurons % (120h)

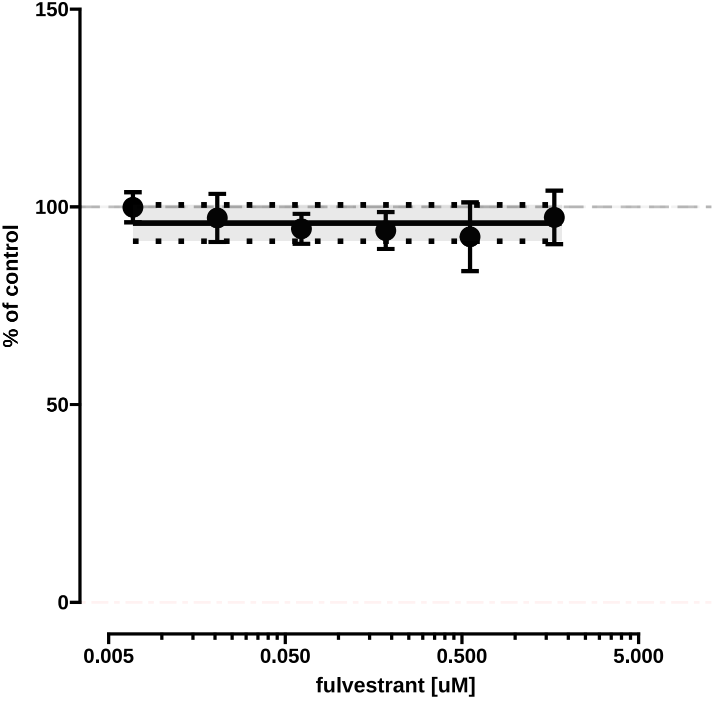

Model: 1-Parameter
Model abbr.: lm.1
Bechmark-Response (BMR): 10

BMCL: NA
BMC: NA
BMCU: NA

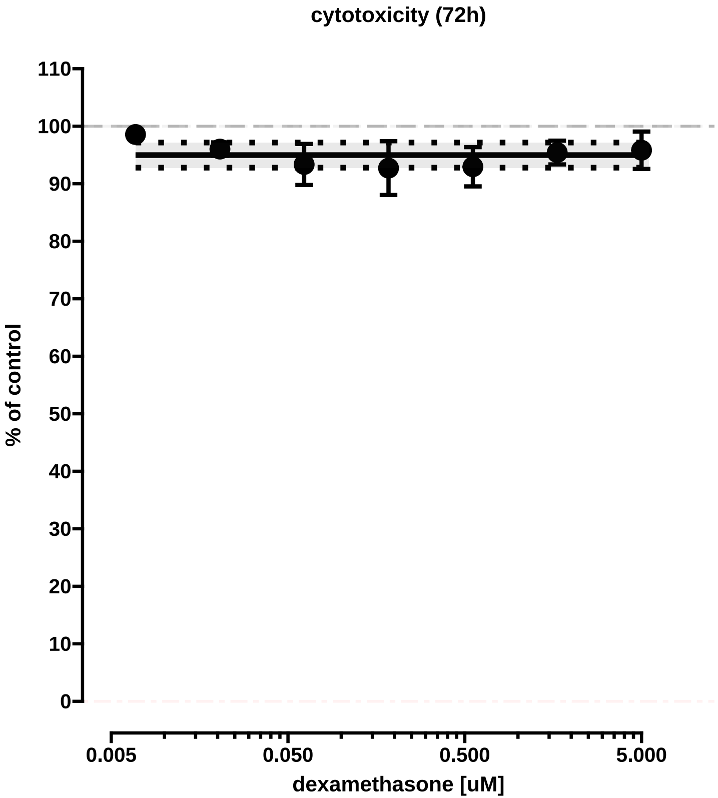

Model: 1-Parameter
Model abbr.: lm.1
Bechmark-Response (BMR): 10

BMCL: NA
BMC: NA
BMCU: NA

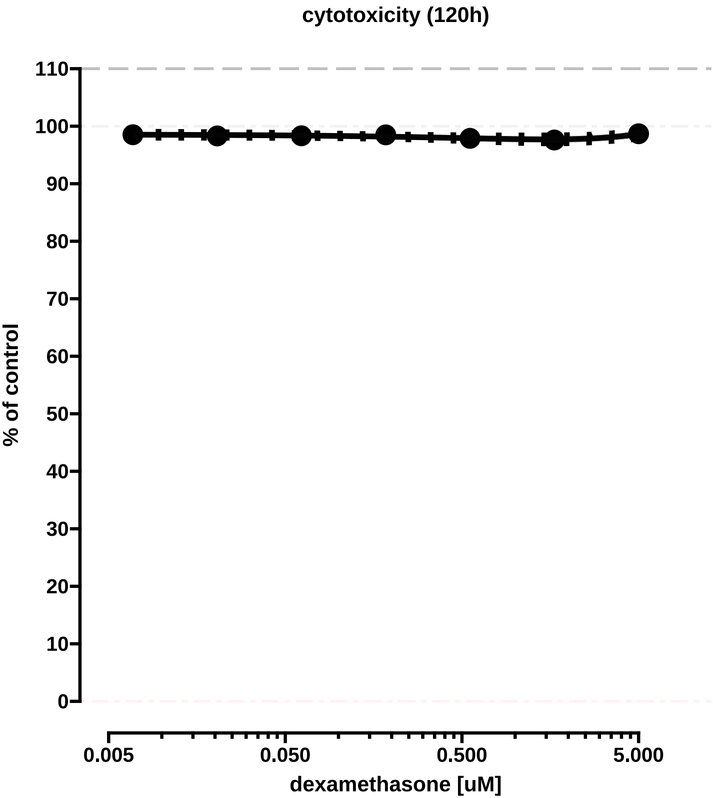

Model: Brain-Cousens (hormesis) with lower limit fixed at 0
Model abbr.: BC.4()
Bechmark-Response (BMR): 10

BMCL: NA
BMC: NA
BMCU: NA

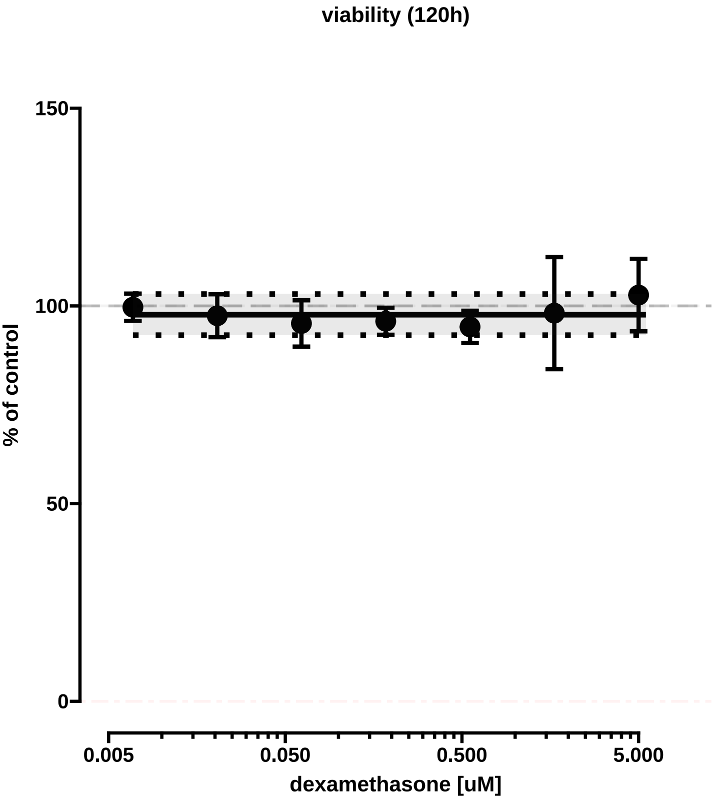

Model: 1-Parameter
Model abbr.: lm.1
Bechmark-Response (BMR): 20

BMCL: NA
BMC: NA
BMCU: NA

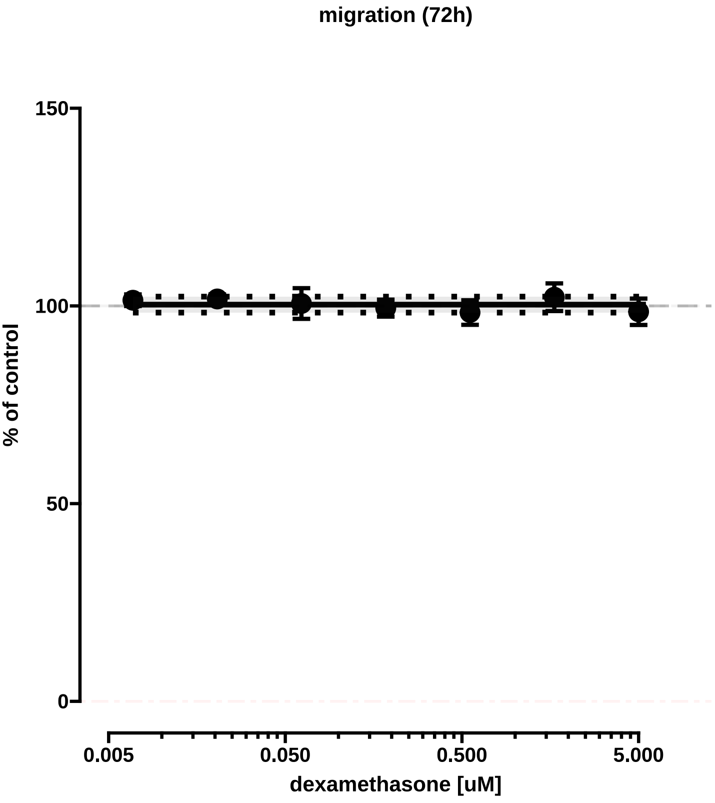

Model: 1-Parameter
Model abbr.: lm.1
Bechmark-Response (BMR): 10

BMCL: NA
BMC: NA
BMCU: NA

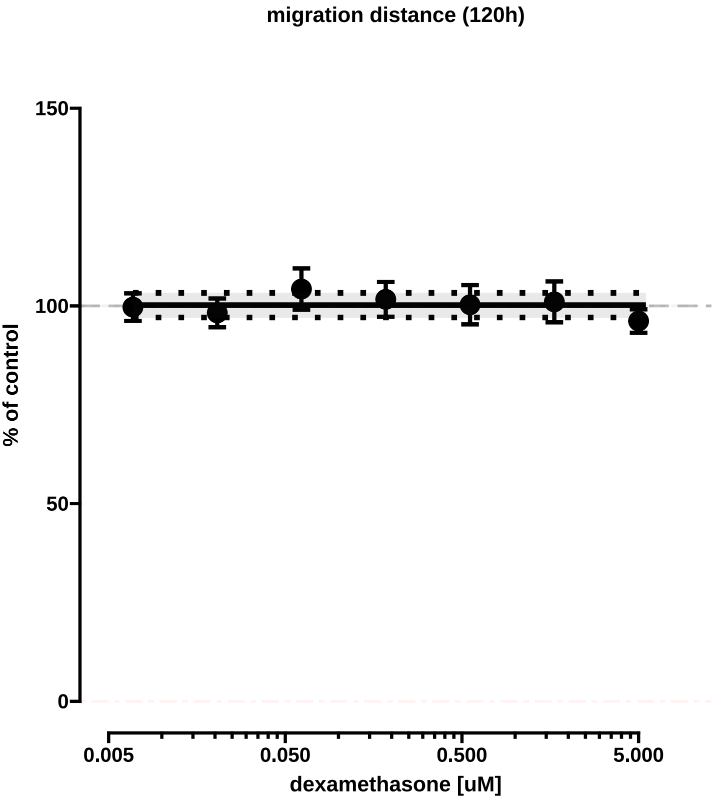

Model: 1-Parameter
Model abbr.: lm.1
Bechmark-Response (BMR): 10

BMCL: NA
BMC: NA
BMCU: NA

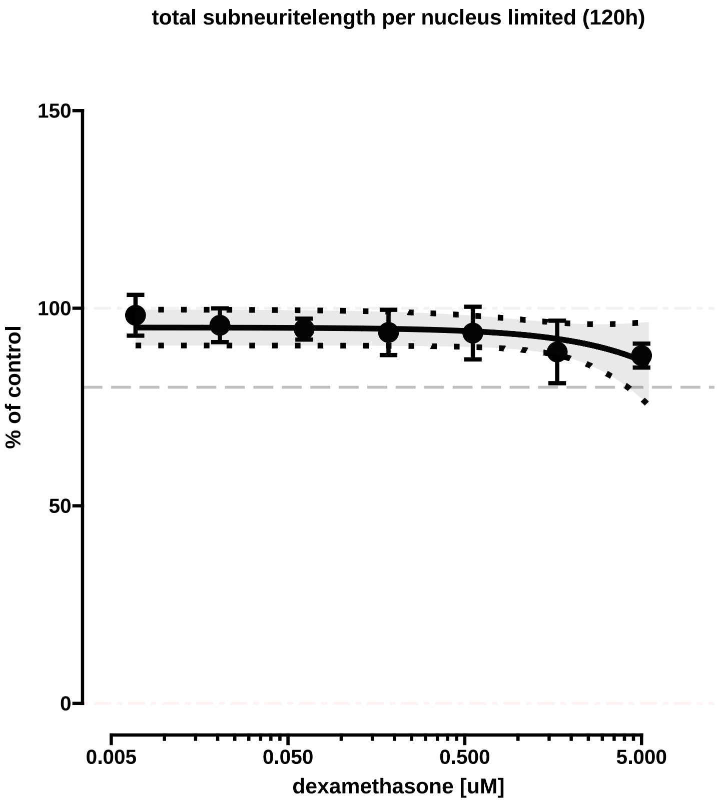

Model: Exponential decay with lower limit at 0
Model abbr.: EXD.2()
Bechmark-Response (BMR): 20

BMCL: NA
BMC: NA
BMCU: NA

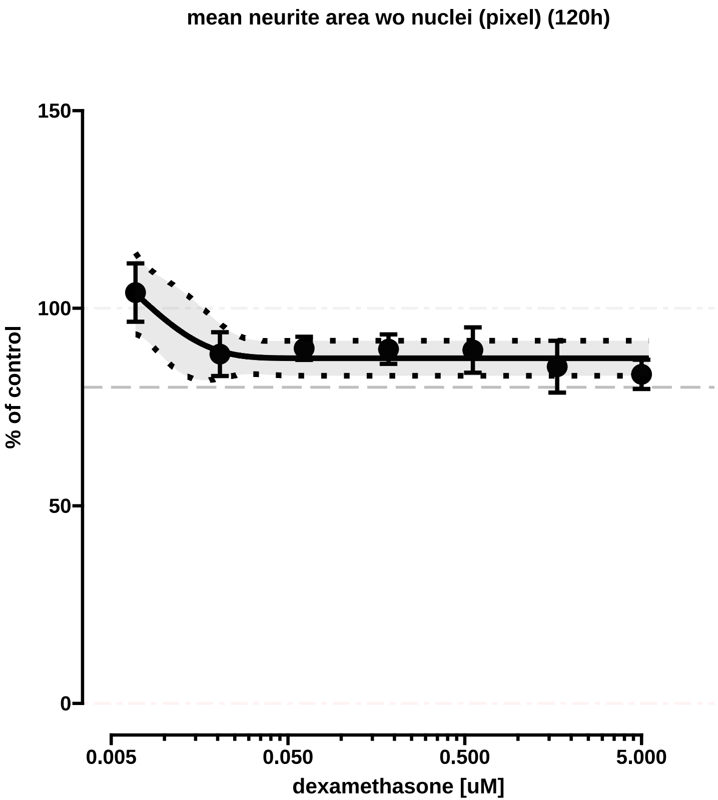

Model: Shifted exponential decay
Model abbr.: EXD.3()
Bechmark-Response (BMR): 20

BMCL: NA
BMC: NA
BMCU: NA

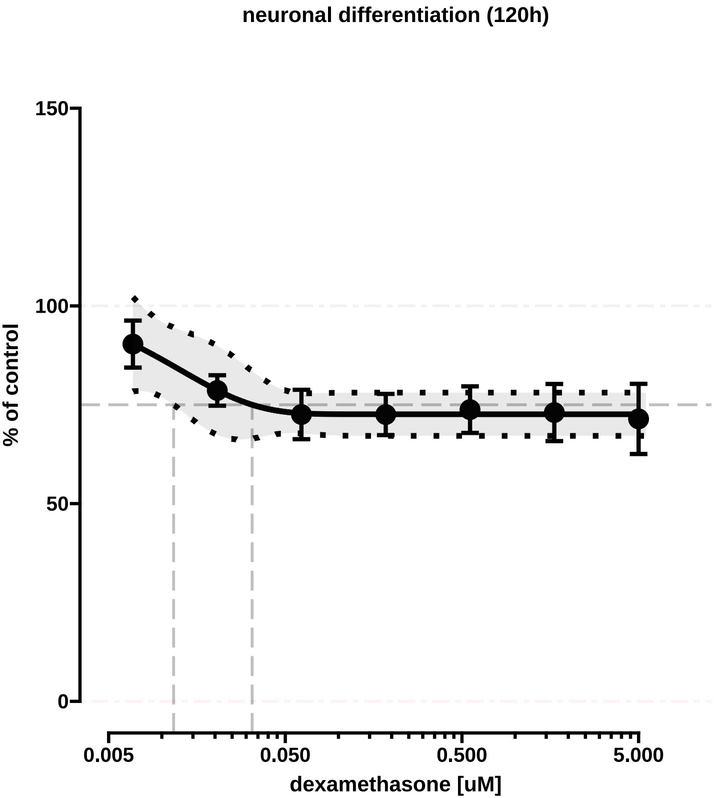

Model: Shifted exponential decay
Model abbr.: EXD.3()
Bechmark-Response (BMR): 25

BMCL: 0.012
BMC: 0.032
BMCU: NA

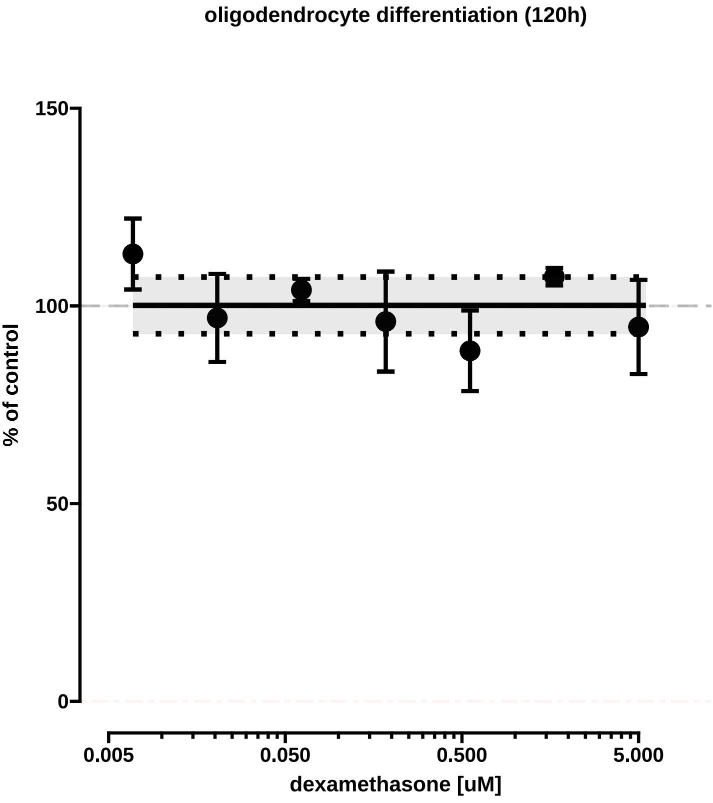

Model: 1-Parameter
Model abbr.: lm.1
Bechmark-Response (BMR): 25

BMCL: NA
BMC: NA
BMCU: NA

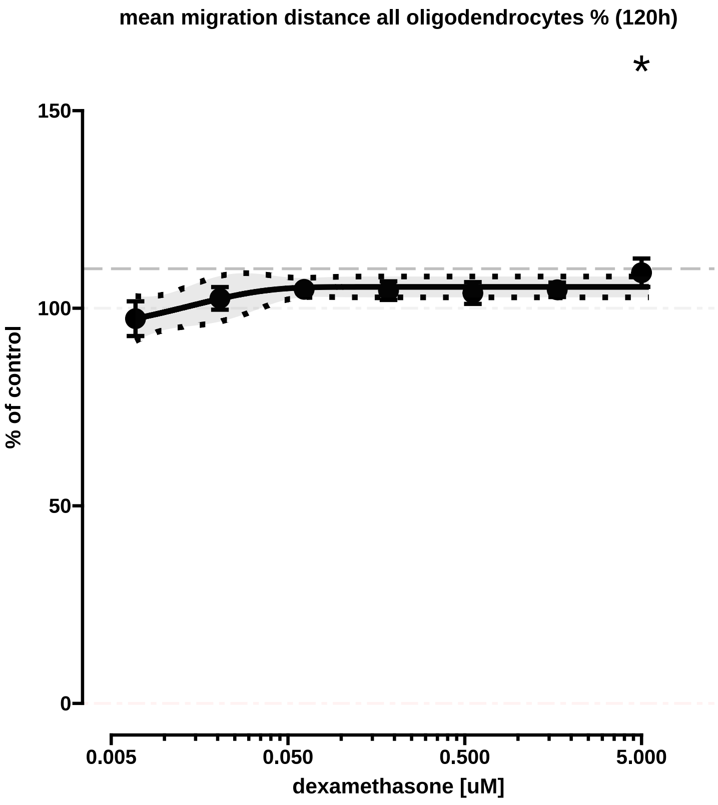

Model: Shifted exponential decay
Model abbr.: EXD.3()
Bechmark-Response (BMR): 10

BMCL: NA
BMC: NA
BMCU: NA

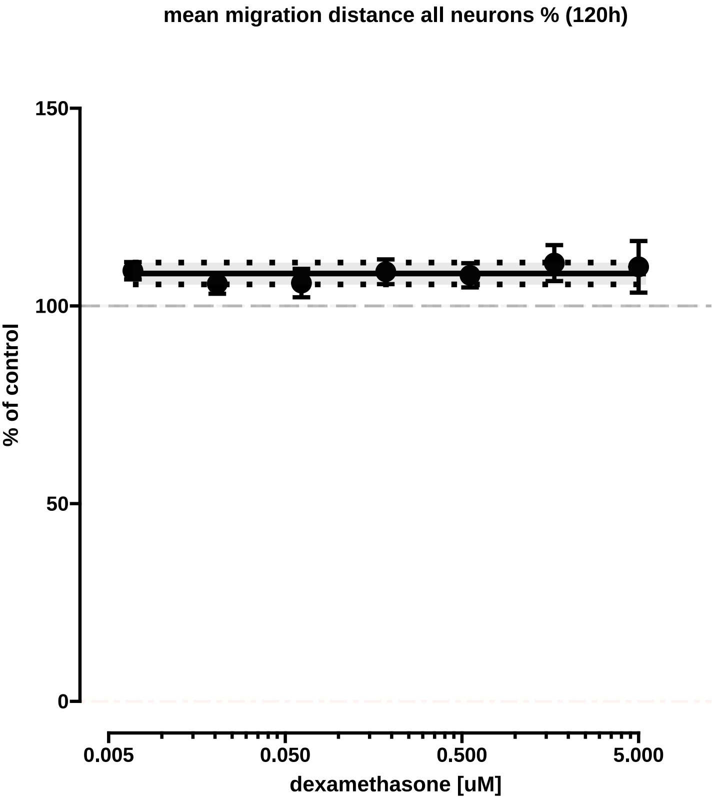

Model: 1-Parameter
Model abbr.: lm.1
Bechmark-Response (BMR): 10

BMCL: NA
BMC: NA
BMCU: NA

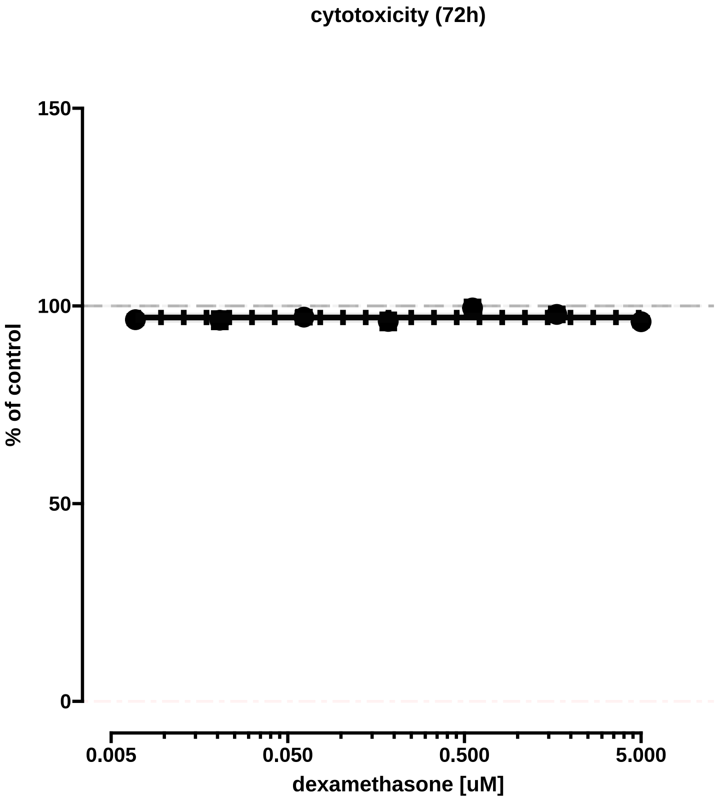

Model: 1-Parameter
Model abbr.: lm.1
Bechmark-Response (BMR): 10

BMCL: NA
BMC: NA
BMCU: NA

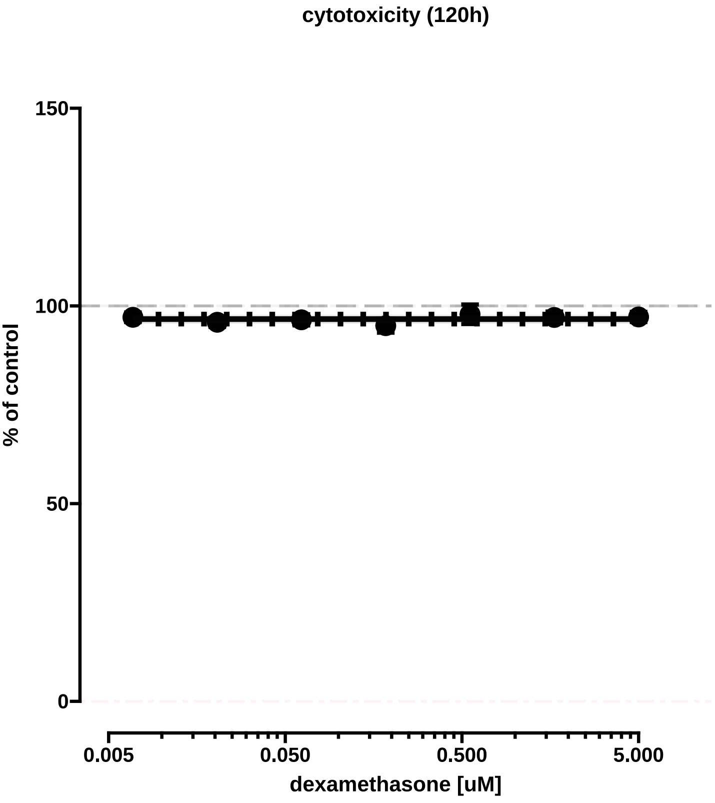

Model: 1-Parameter
Model abbr.: lm.1
Bechmark-Response (BMR): 10

BMCL: NA
BMC: NA
BMCU: NA

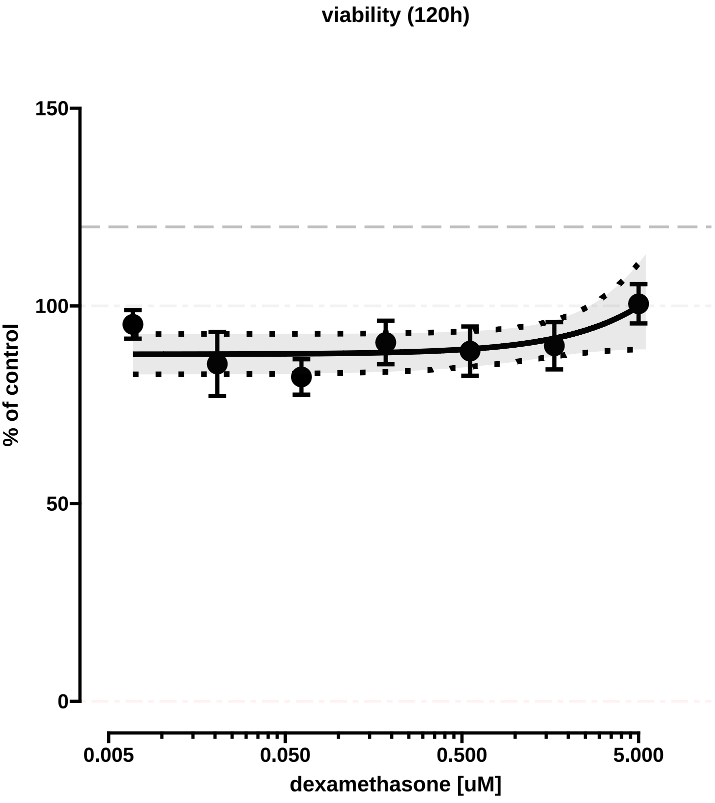

Model: Linear
Model abbr.: lm
Bechmark-Response (BMR): 20

BMCL: NA
BMC: NA
BMCU: NA

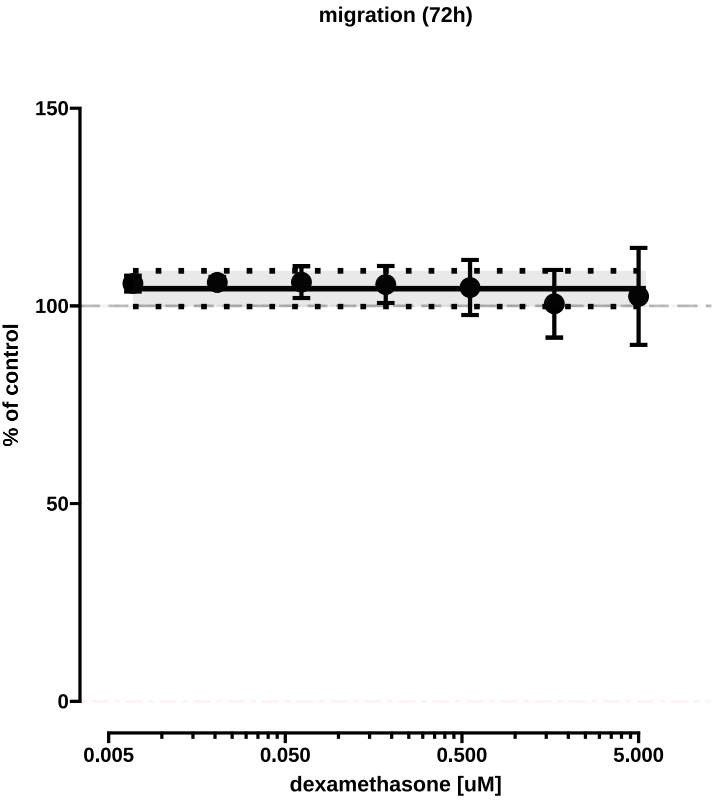

Model: 1-Parameter
Model abbr.: lm.1
Bechmark-Response (BMR): 10

BMCL: NA
BMC: NA
BMCU: NA

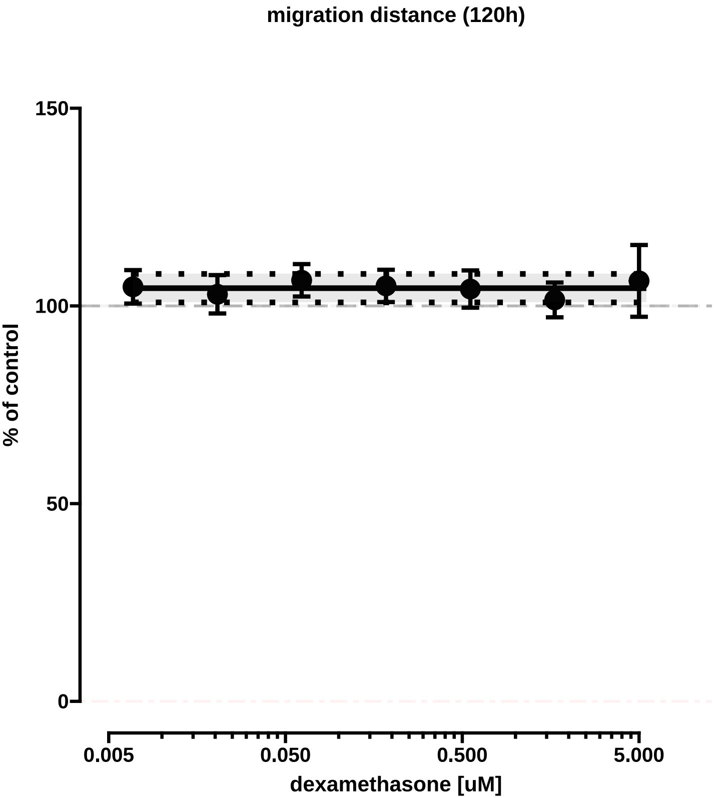

Model: 1-Parameter
Model abbr.: lm.1
Bechmark-Response (BMR): 10

BMCL: NA
BMC: NA
BMCU: NA

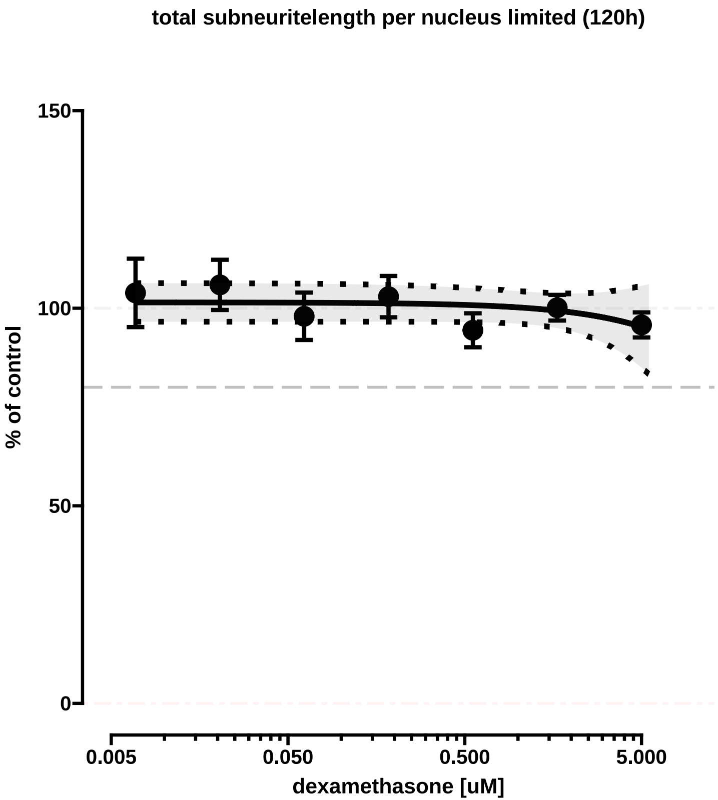

Model: Exponential decay with lower limit at 0
Model abbr.: EXD.2()
Bechmark-Response (BMR): 20

BMCL: NA
BMC: NA
BMCU: NA

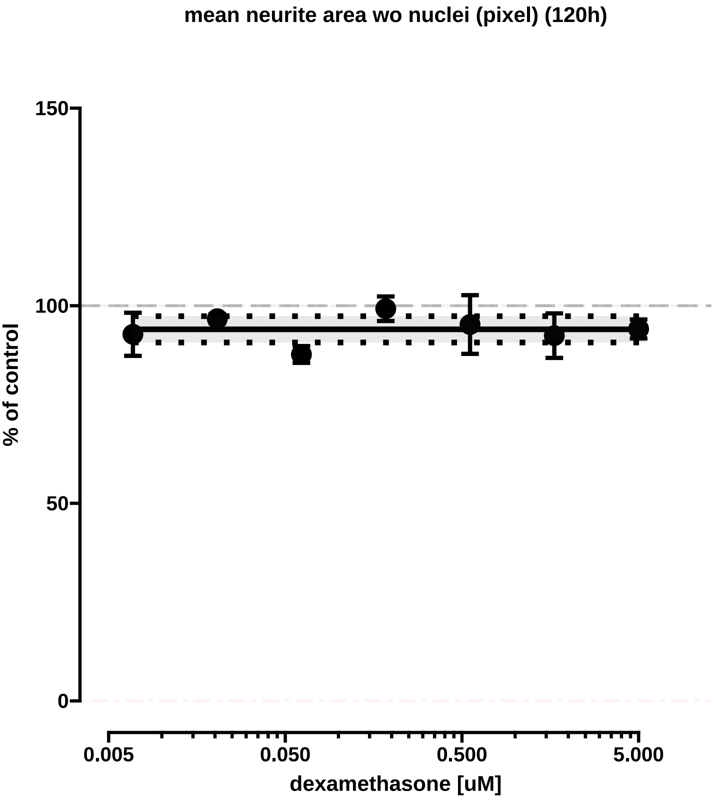

Model: 1-Parameter
Model abbr.: lm.1
Bechmark-Response (BMR): 20

BMCL: NA
BMC: NA
BMCU: NA

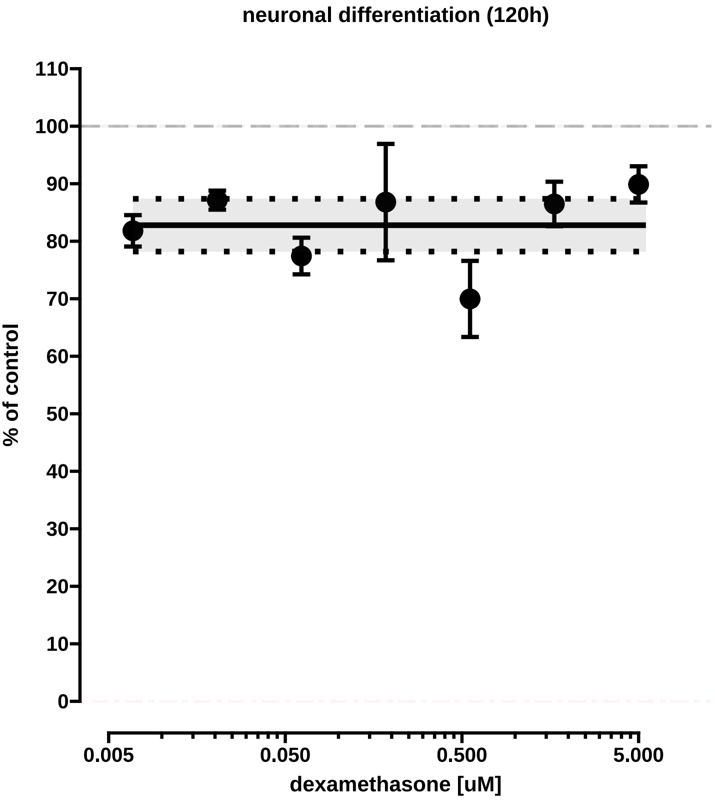

Model: 1-Parameter
Model abbr.: lm.1
Bechmark-Response (BMR): 25

BMCL: NA
BMC: NA
BMCU: NA

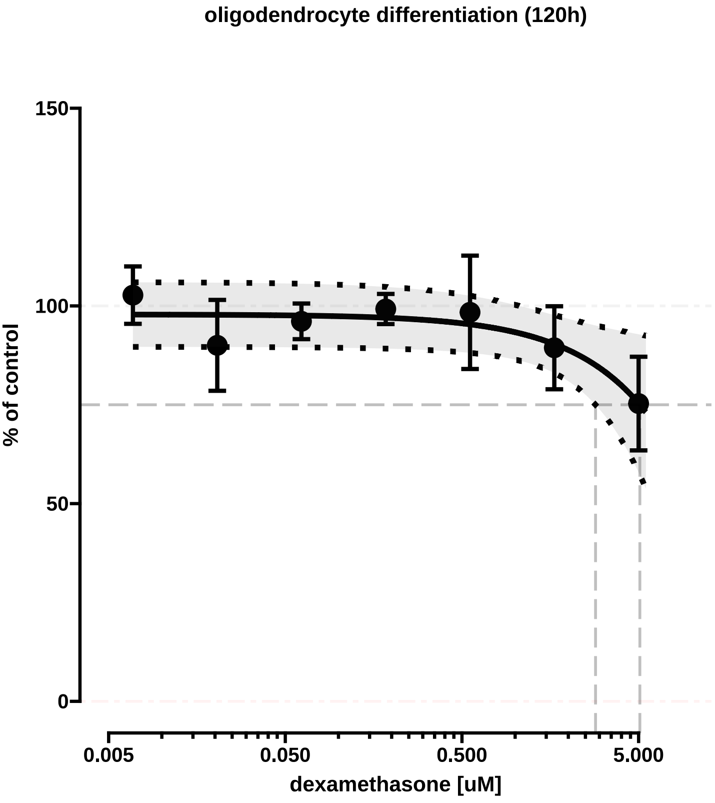

Model: Linear
Model abbr.: lm
Bechmark-Response (BMR): 25

BMCL: 2.852
BMC: 5.083
BMCU: NA

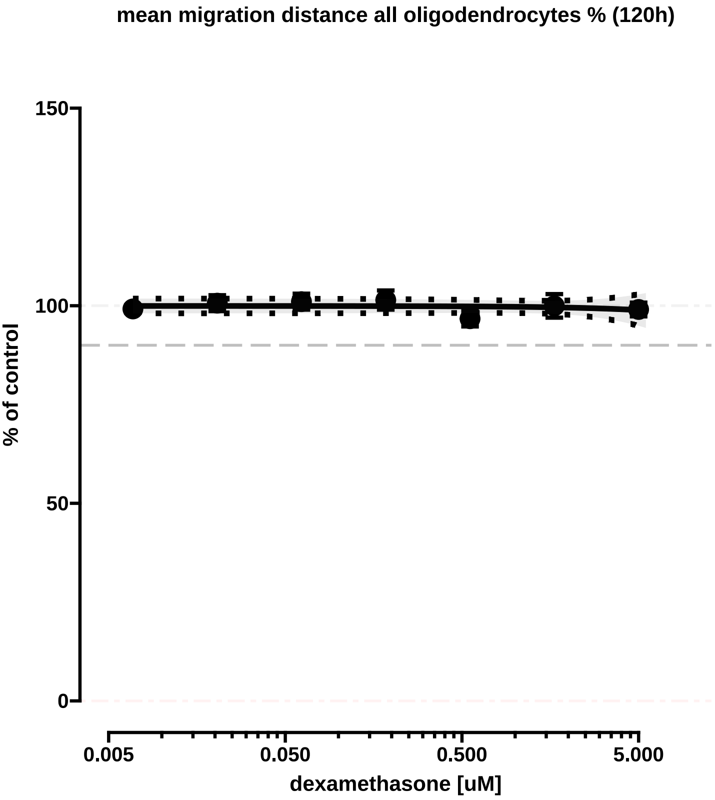

Model: Exponential decay with lower limit at 0
Model abbr.: EXD.2()
Bechmark-Response (BMR): 10

BMCL: NA
BMC: NA
BMCU: NA

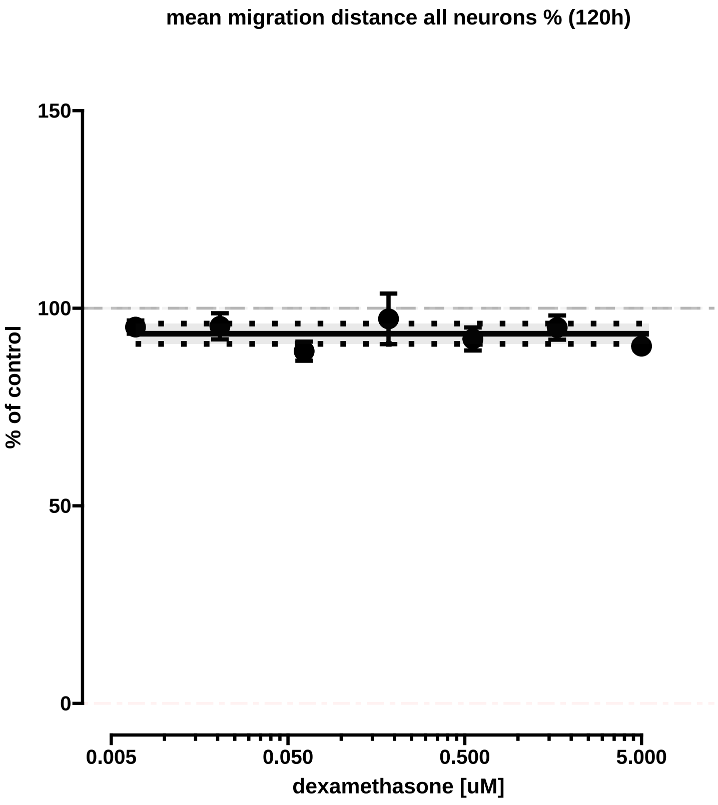

Model: 1-Parameter
Model abbr.: lm.1
Bechmark-Response (BMR): 10

BMCL: NA
BMC: NA
BMCU: NA

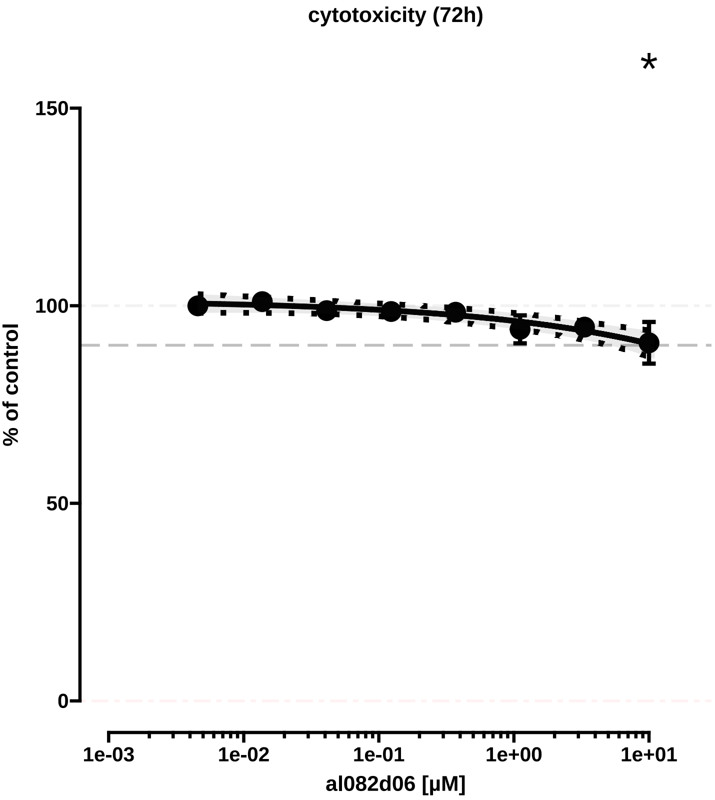

Model: Weibull (type 1) with lower limit at 0
Model abbr.: W1.3()
Bechmark-Response (BMR): 10

BMCL: NA
BMC: NA
BMCU: NA

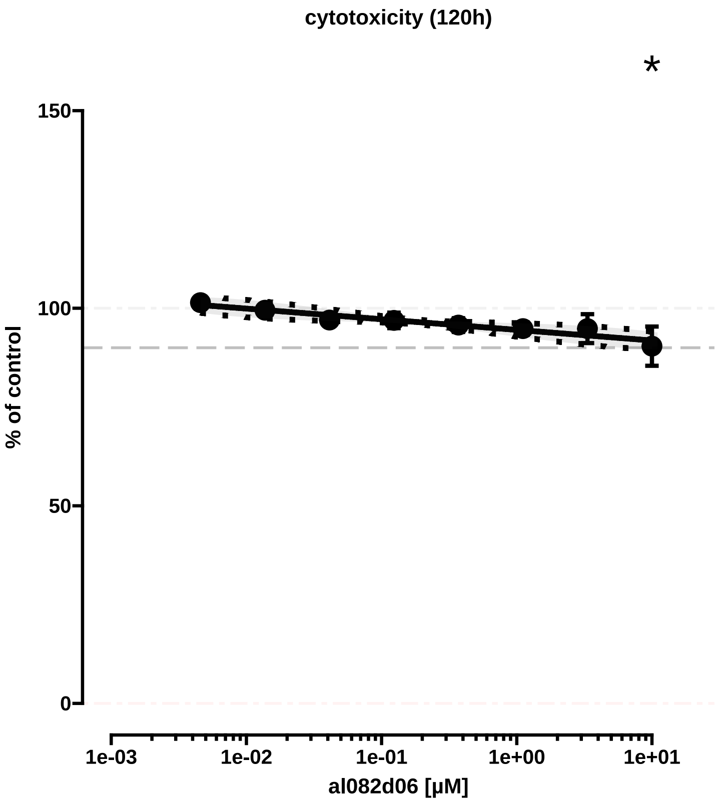

Model: Log-logistic (log(ED50) as parameter) with lower limit at 0
Model abbr.: LL2.3()
Bechmark-Response (BMR): 10

BMCL: NA
BMC: NA
BMCU: NA

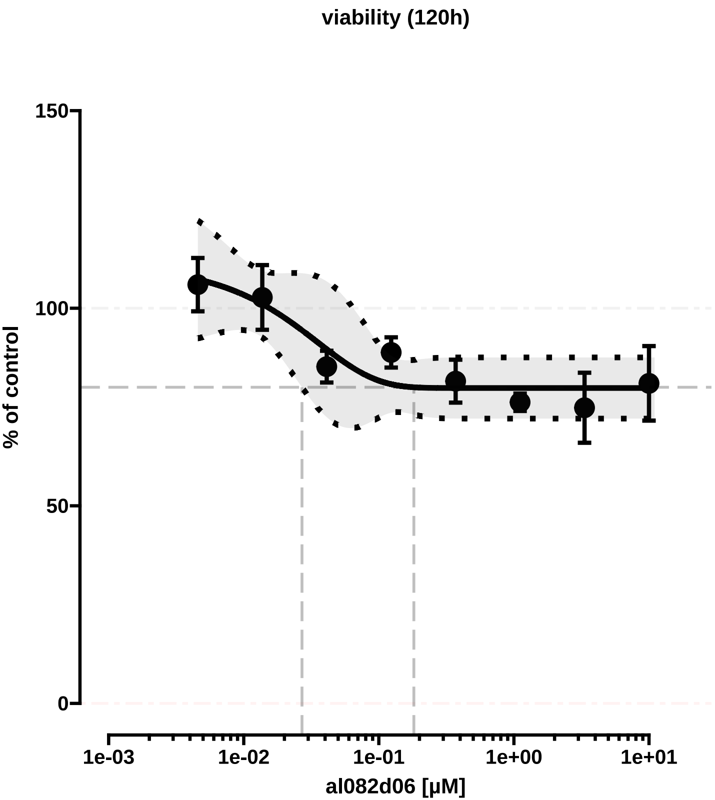

Model: Shifted exponential decay
Model abbr.: EXD.3()
Bechmark-Response (BMR): 20

BMCL: 0.027
BMC: 0.182
BMCU: NA

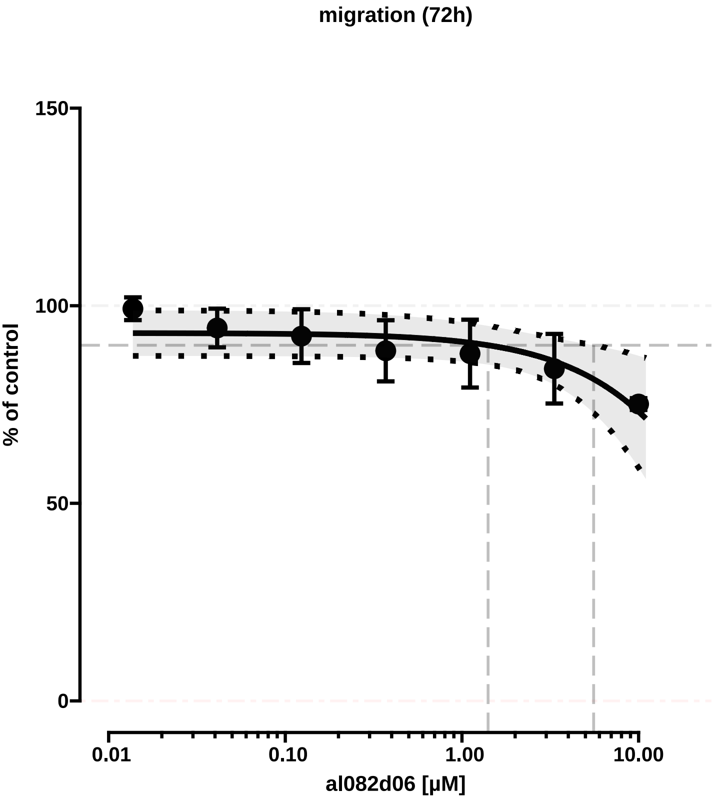

Model: Exponential decay with lower limit at 0
Model abbr.: EXD.2()
Bechmark-Response (BMR): 10

BMCL: NA
BMC: 1.406
BMCU: 5.565

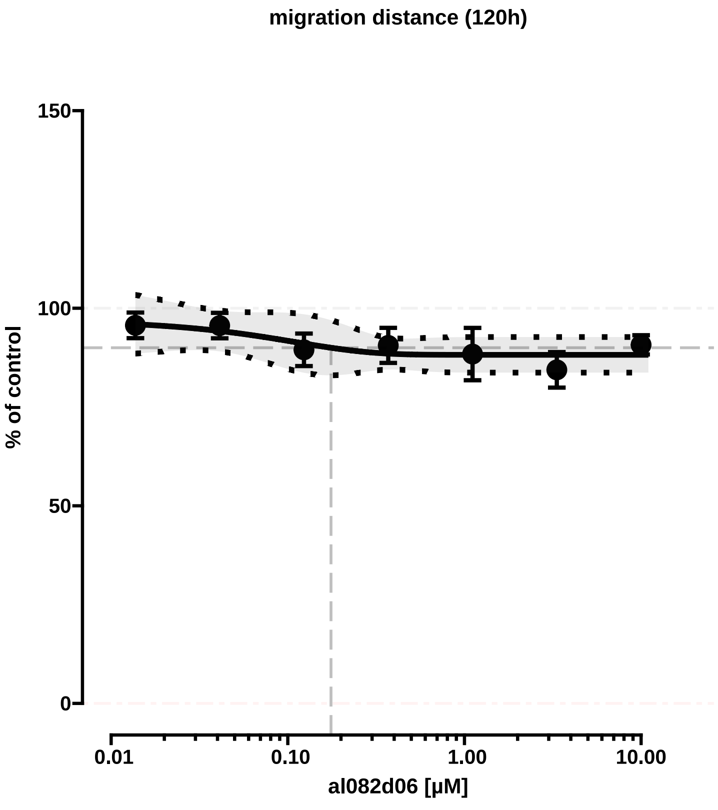

Model: Shifted exponential decay
Model abbr.: EXD.3()
Bechmark-Response (BMR): 10

BMCL: NA
BMC: 0.176
BMCU: NA

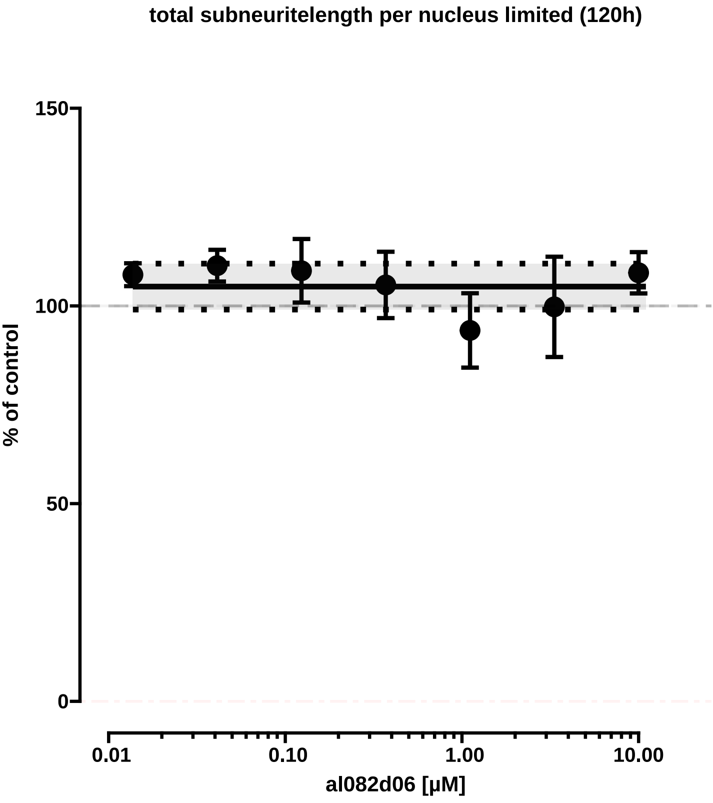

Model: 1-Parameter
Model abbr.: lm.1
Bechmark-Response (BMR): 20

BMCL: NA
BMC: NA
BMCU: NA

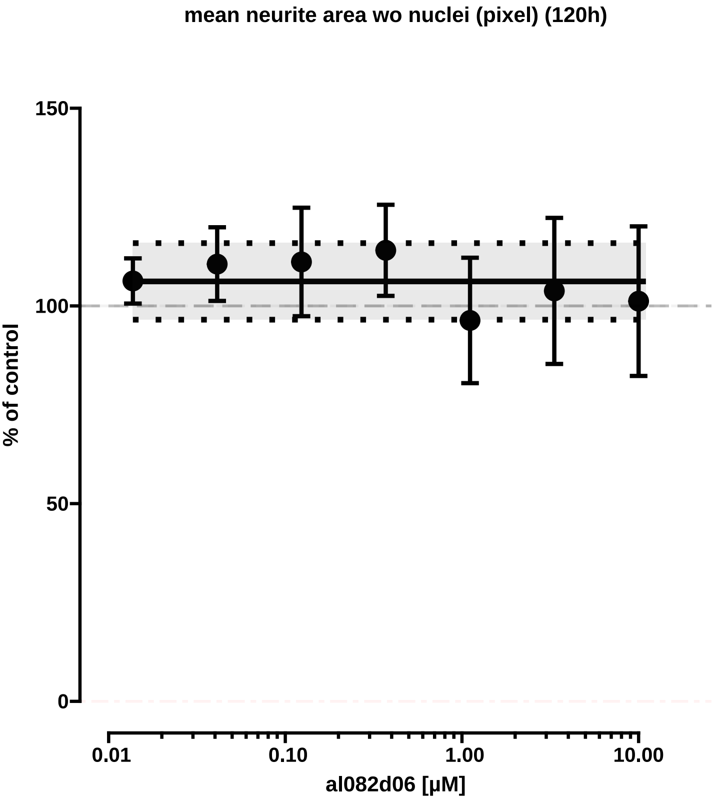

Model: 1-Parameter
Model abbr.: lm.1
Bechmark-Response (BMR): 20

BMCL: NA
BMC: NA
BMCU: NA

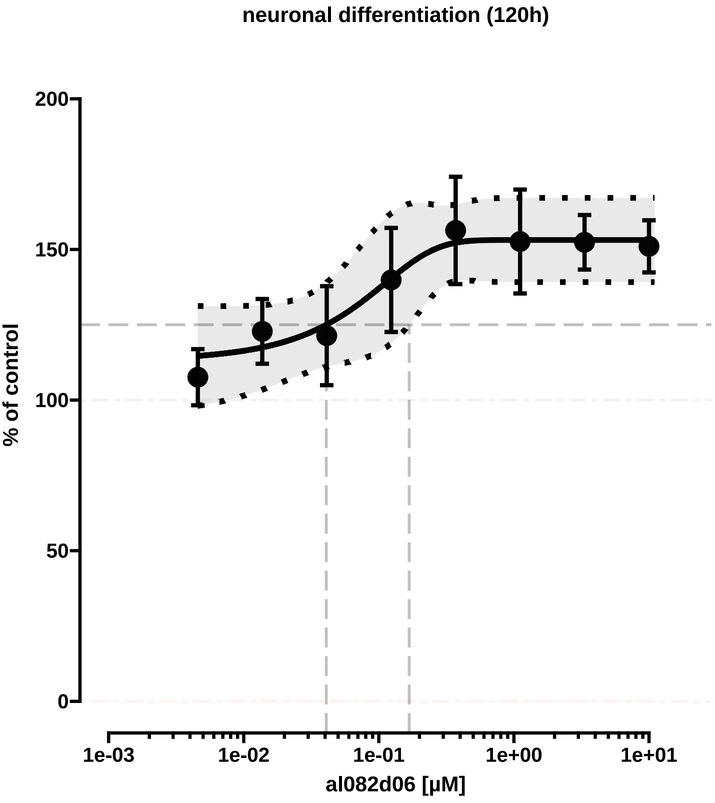

Model: Logistic (ED50 as parameter) with lower limit fixed at 0
Model abbr.: L.3()
Bechmark-Response (BMR): 25

BMCL: NA
BMC: 0.041
BMCU: 0.168

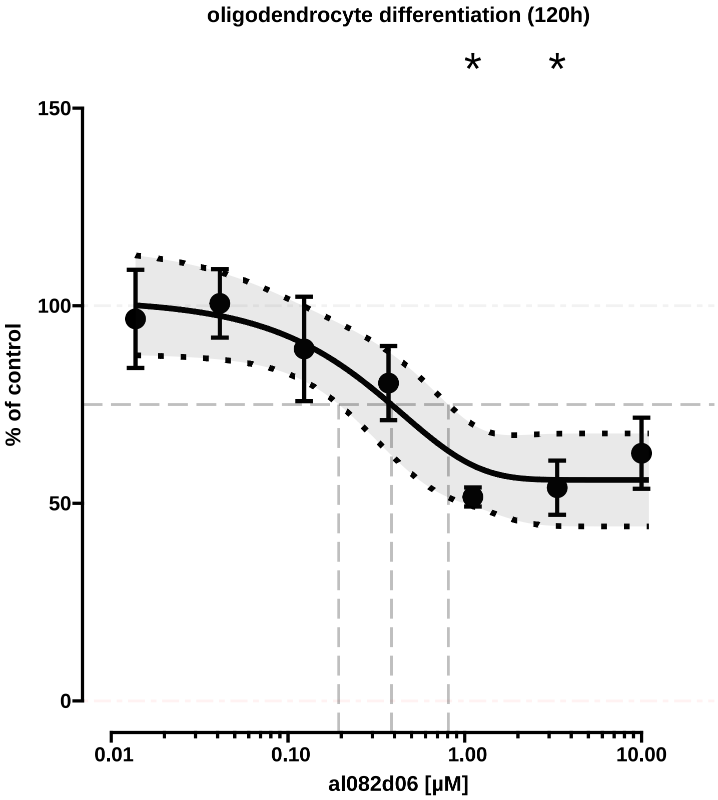

Model: Shifted exponential decay
Model abbr.: EXD.3()
Bechmark-Response (BMR): 25

BMCL: 0.194
BMC: 0.384
BMCU: 0.807

mean migration distance all oligodendrocytes % (120h)

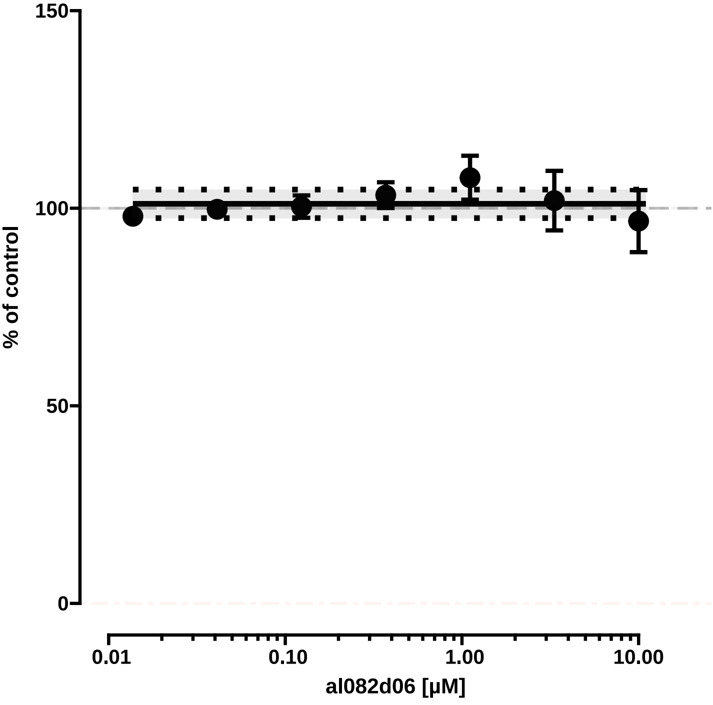

Model: 1-Parameter
Model abbr.: lm.1
Bechmark-Response (BMR): 10

BMCL: NA
BMC: NA
BMCU: NA

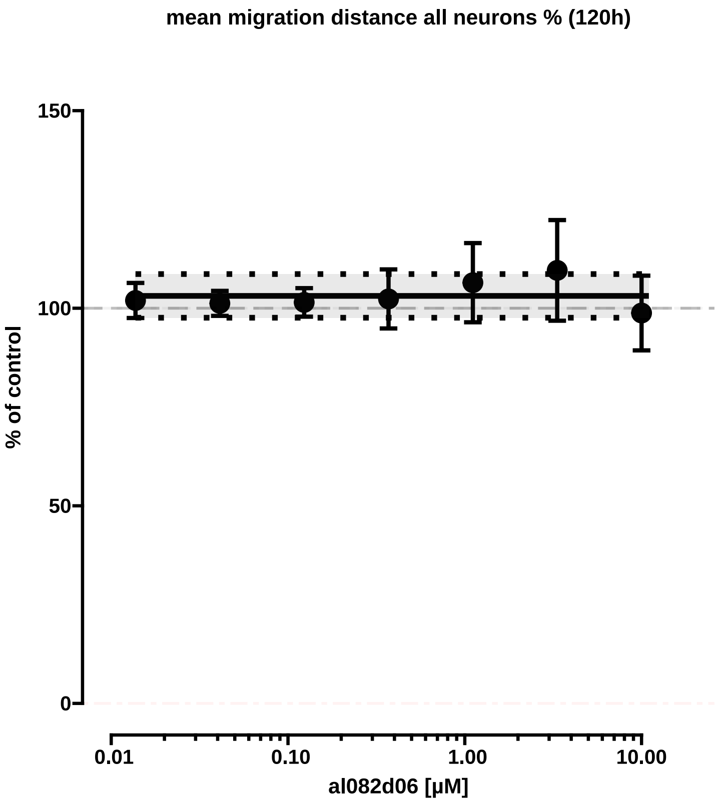

Model: 1-Parameter
Model abbr.: lm.1
Bechmark-Response (BMR): 10

BMCL: NA
BMC: NA
BMCU: NA

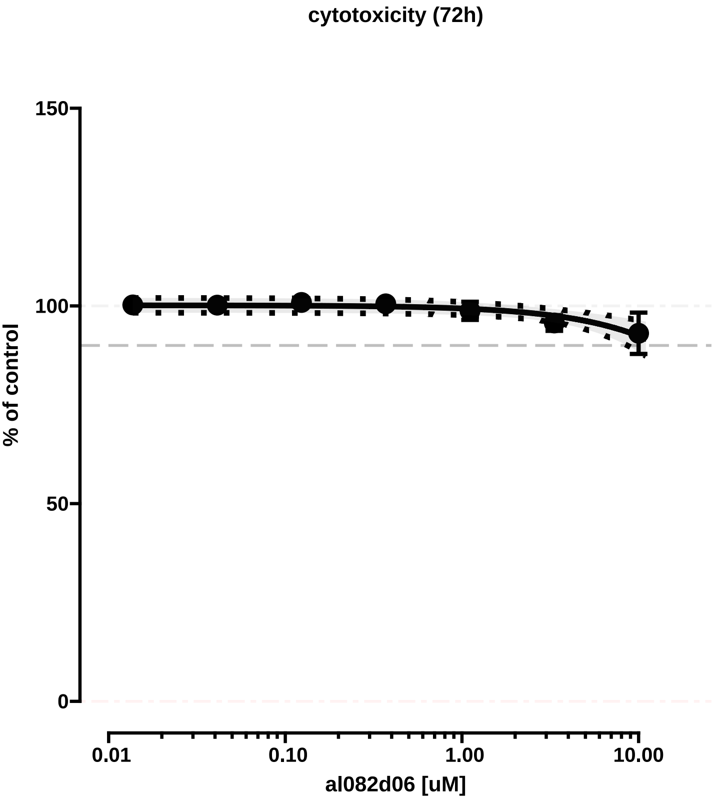

Model: Exponential decay with lower limit at 0
Model abbr.: EXD.2()
Bechmark-Response (BMR): 10

BMCL: NA
BMC: NA
BMCU: NA

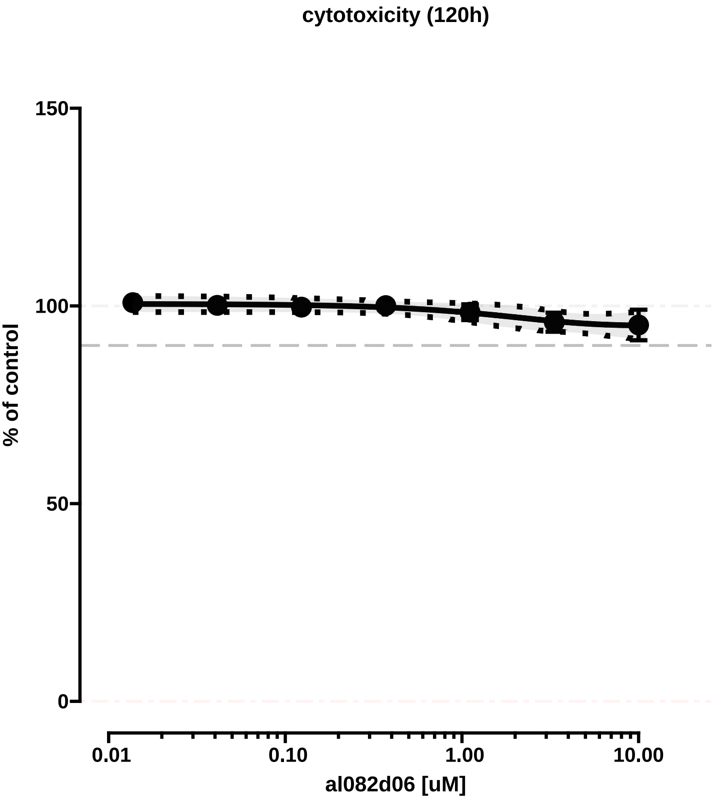

Model: Shifted exponential decay
Model abbr.: EXD.3()
Bechmark-Response (BMR): 10

BMCL: NA
BMC: NA
BMCU: NA

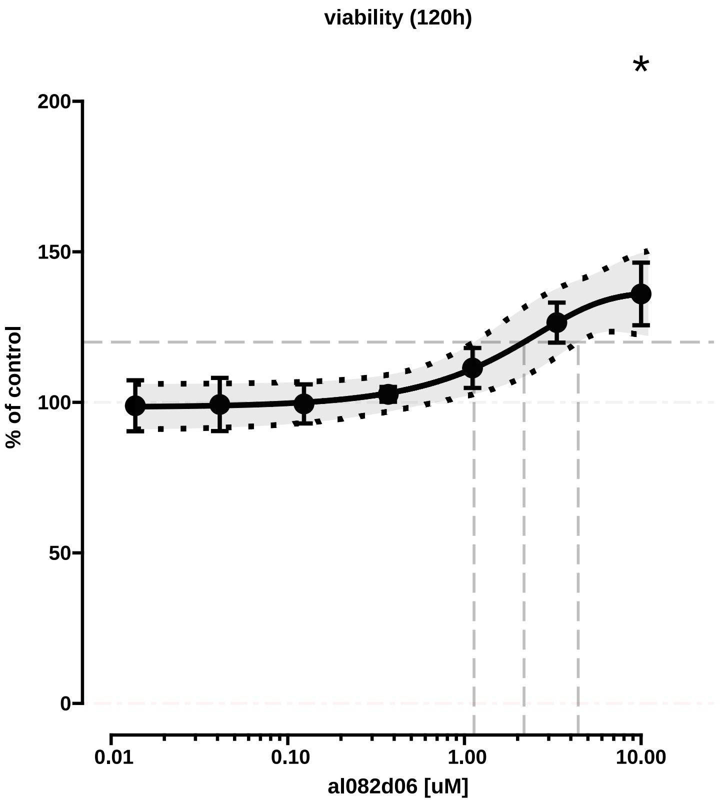

Model: Logistic (ED50 as parameter) with lower limit fixed at 0
Model abbr.: L.3()
Bechmark-Response (BMR): 20

BMCL: 1.133
BMC: 2.174
BMCU: 4.405

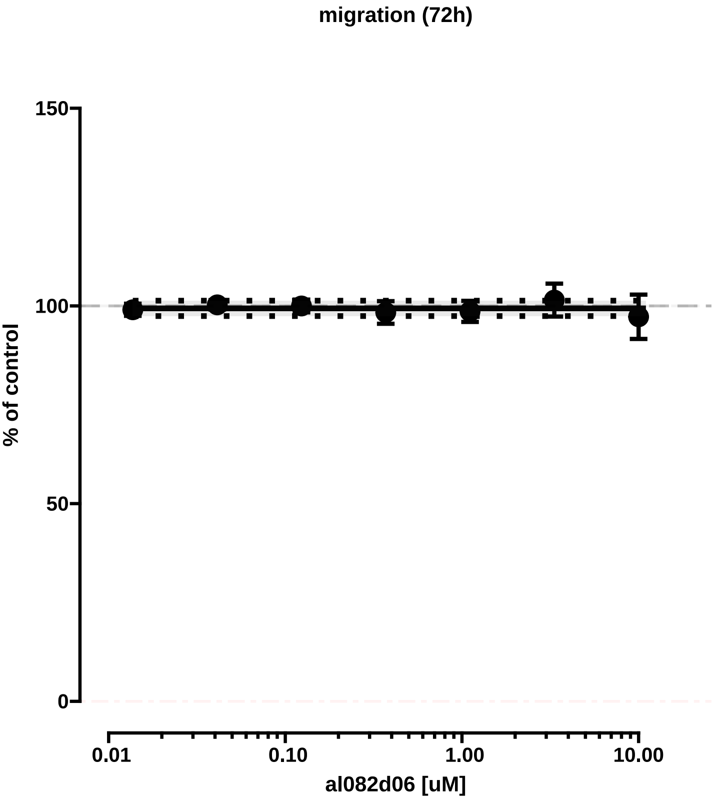

Model: 1-Parameter
Model abbr.: lm.1
Bechmark-Response (BMR): 10

BMCL: NA
BMC: NA
BMCU: NA

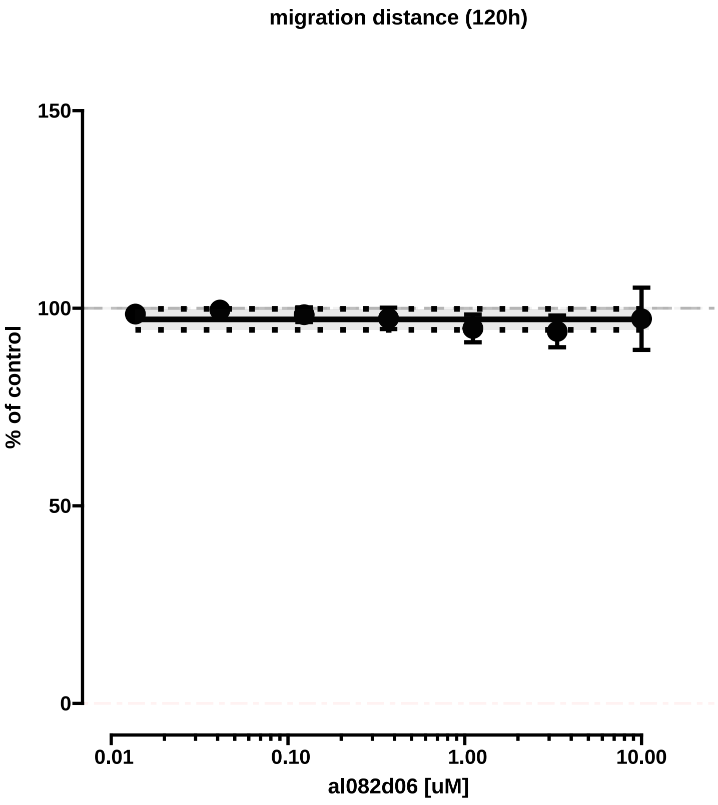

Model: 1-Parameter
Model abbr.: lm.1
Bechmark-Response (BMR): 10

BMCL: NA
BMC: NA
BMCU: NA

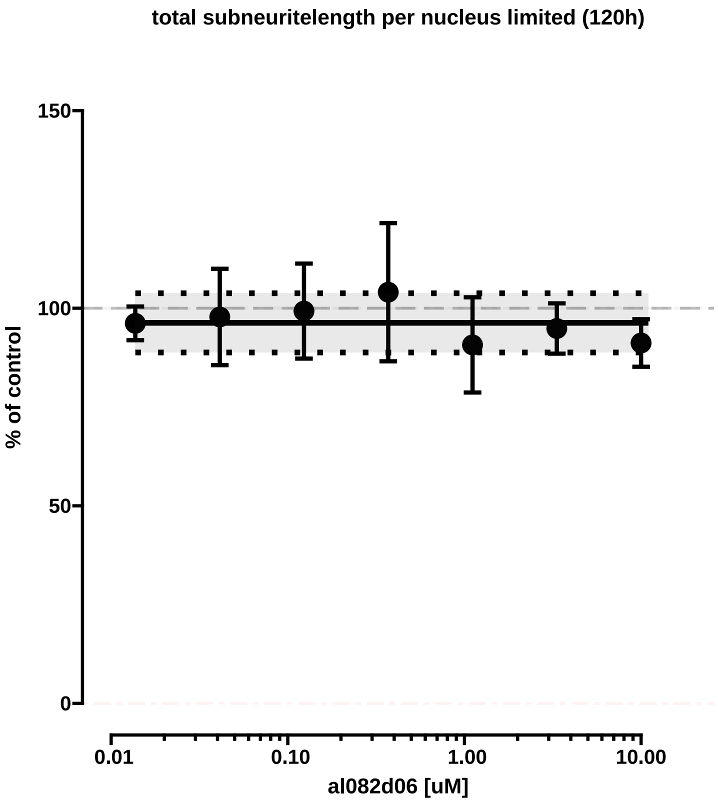

Model: 1-Parameter
Model abbr.: lm.1
Bechmark-Response (BMR): 20

BMCL: NA
BMC: NA
BMCU: NA

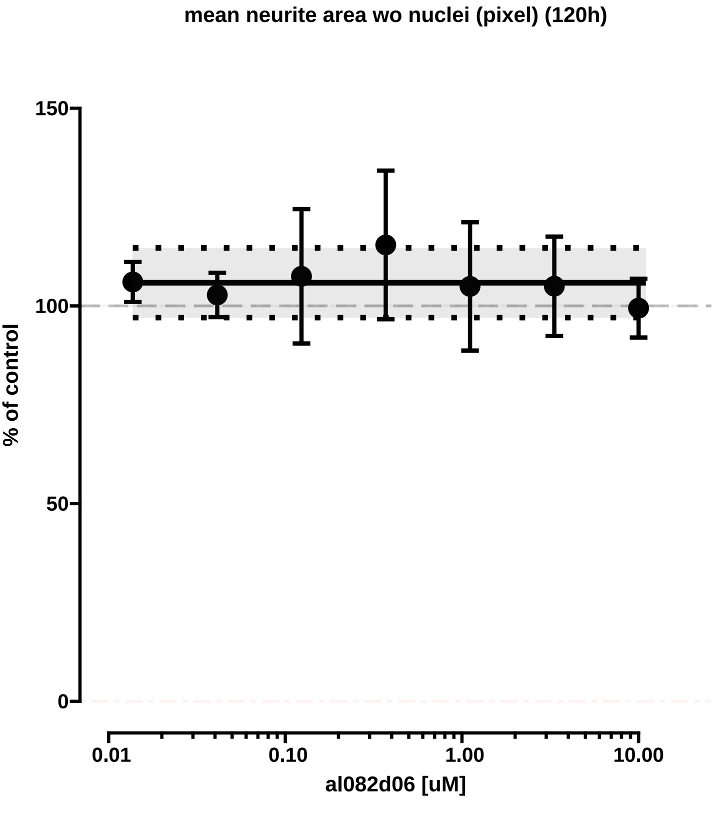

Model: 1-Parameter
Model abbr.: lm.1
Bechmark-Response (BMR): 20

BMCL: NA
BMC: NA
BMCU: NA

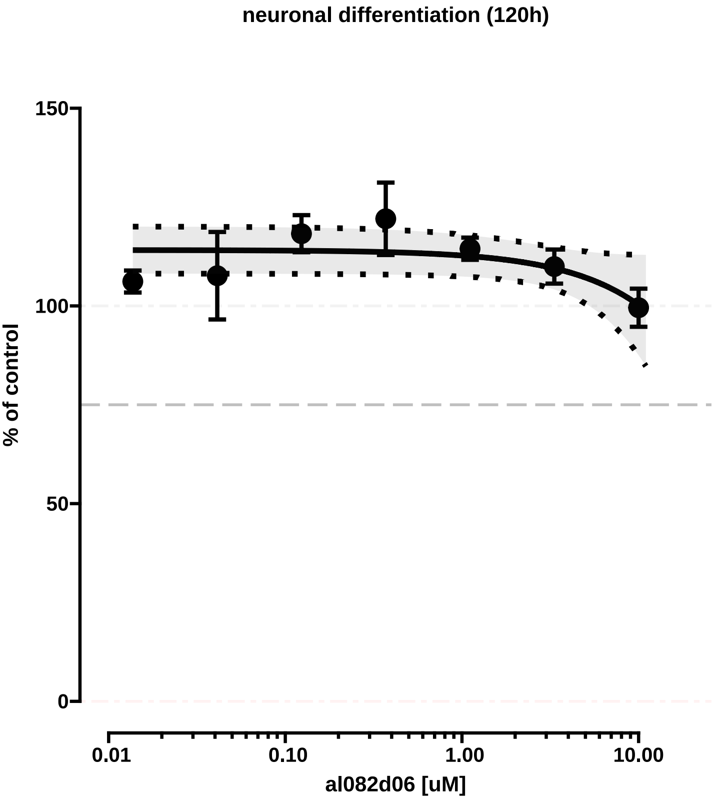

Model: Linear
Model abbr.: lm
Bechmark-Response (BMR): 25

BMCL: NA
BMC: NA
BMCU: NA

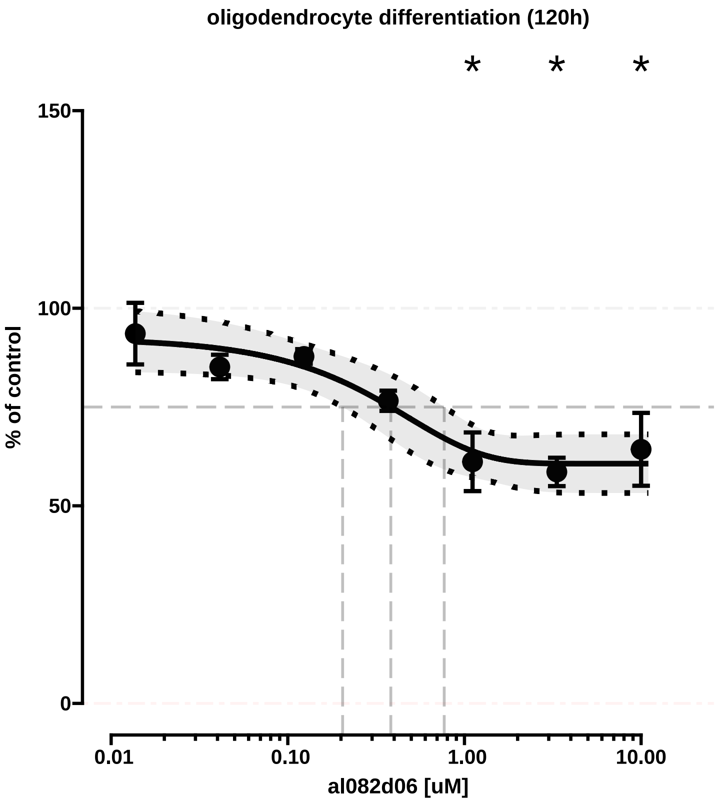

Model: Shifted exponential decay
Model abbr.: EXD.3()
Bechmark-Response (BMR): 25

BMCL: 0.204
BMC: 0.383
BMCU: 0.768

mean migration distance all oligodendrocytes % (120h)

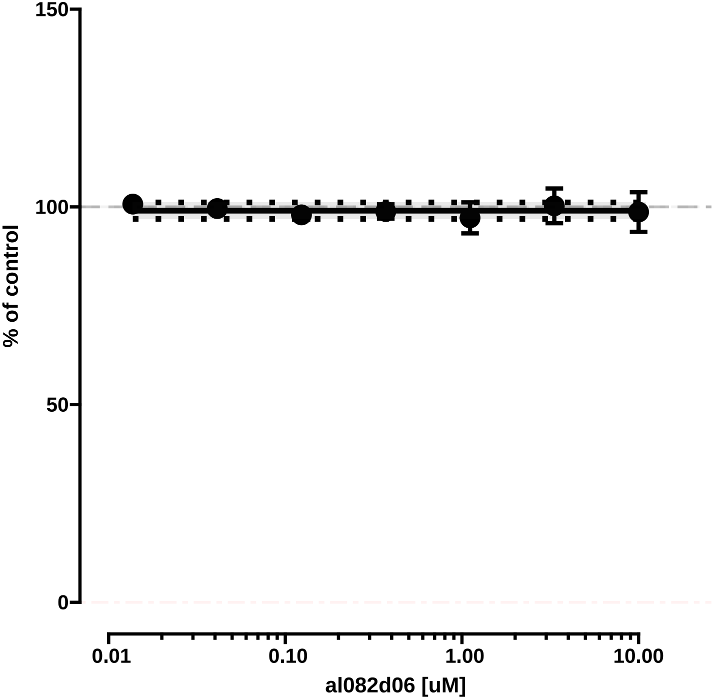

Model: 1-Parameter
Model abbr.: lm.1
Bechmark-Response (BMR): 10

BMCL: NA
BMC: NA
BMCU: NA

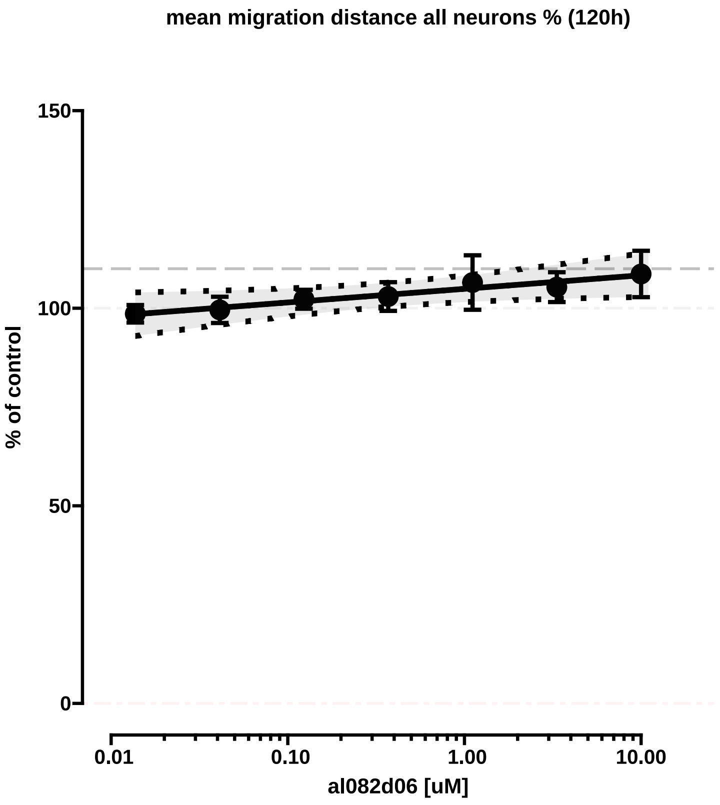

Model: Weibull (type 1) with lower limit at 0
Model abbr.: W1.3()
Bechmark-Response (BMR): 10

BMCL: NA
BMC: NA
BMCU: NA

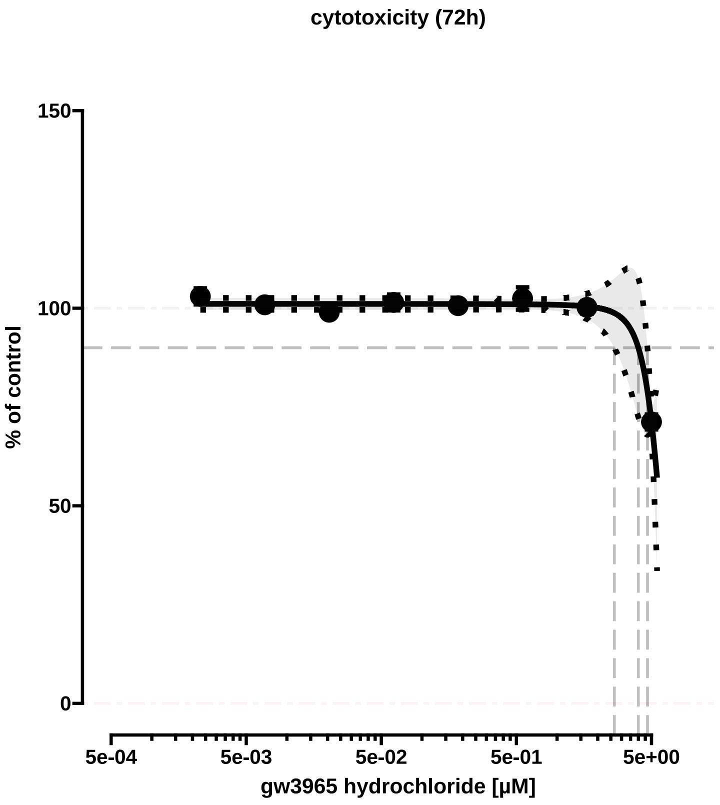

Model: Logistic (ED50 as parameter) with lower limit fixed at 0
Model abbr.: L.3()
Bechmark-Response (BMR): 10

BMCL: 2.656
BMC: 3.997
BMCU: 4.67

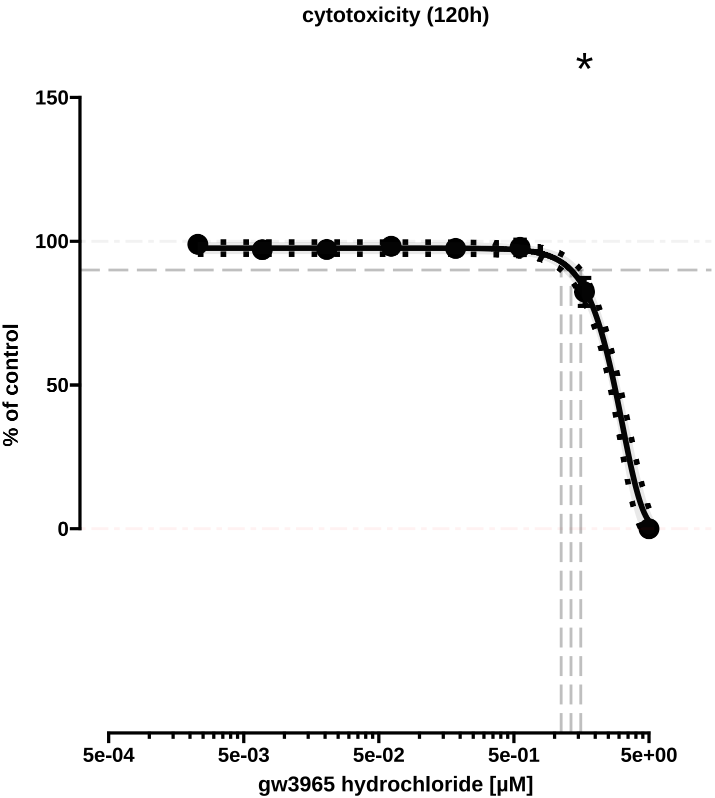

Model: Weibull (type 1) with lower limit at 0
Model abbr.: W1.3()
Bechmark-Response (BMR): 10

BMCL: 1.119
BMC: 1.323
BMCU: 1.563

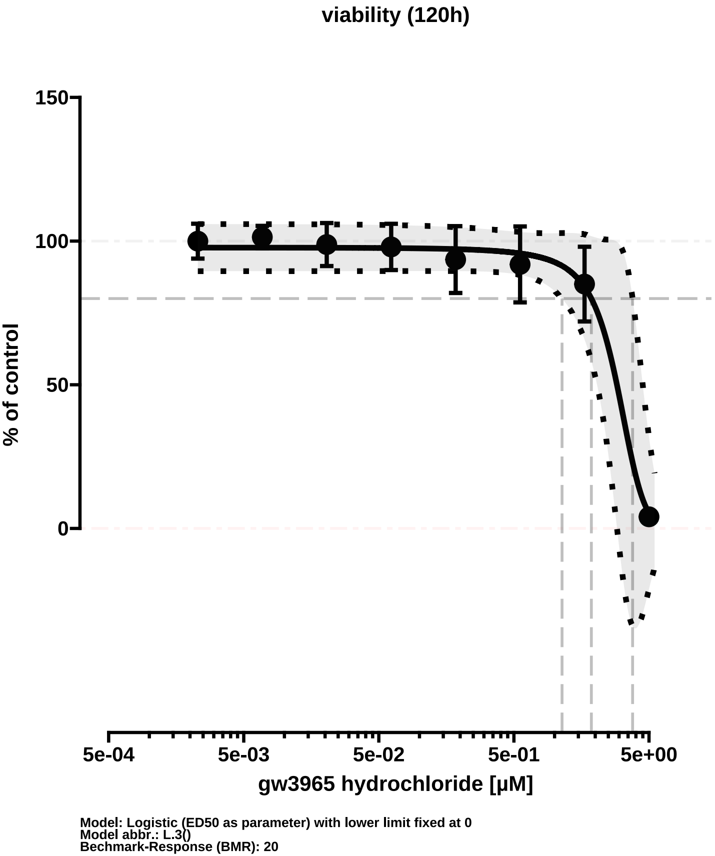

Model: Logistic (ED50 as parameter) with lower limit fixed at 0
Model abbr.: L.3()
Bechmark-Response (BMR): 20

BMCL: 1.136
BMC: 1.872
BMCU: 3.782

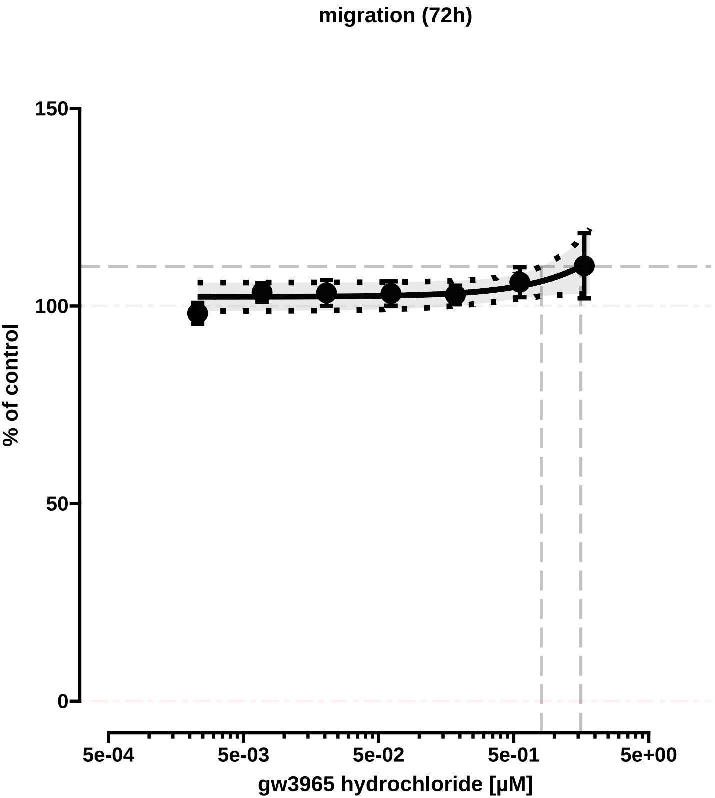

Model: Linear
Model abbr.: lm
Bechmark-Response (BMR): 10

BMCL: 0.801
BMC: 1.568
BMCU: NA

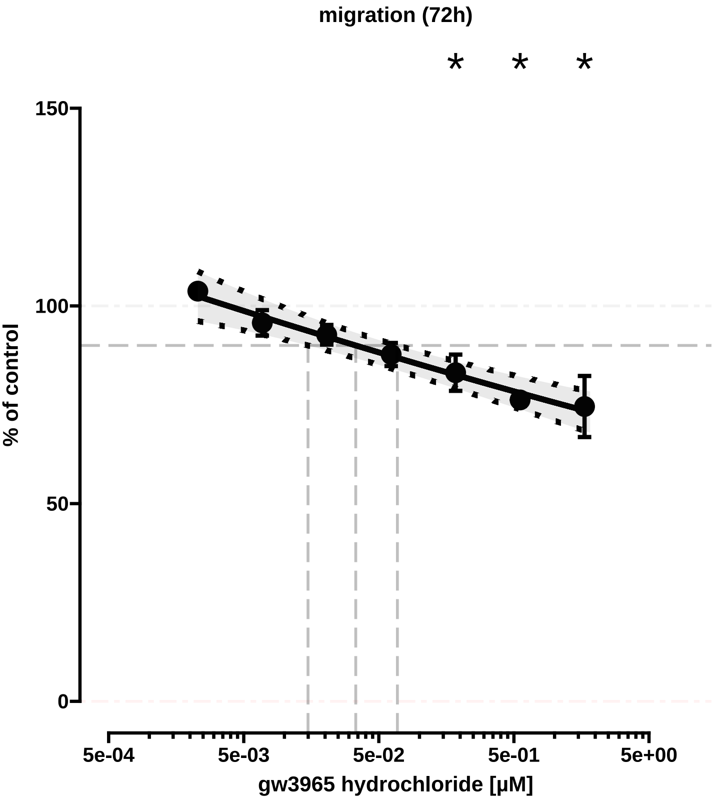

Model: Log-logistic (log(ED50) as parameter) with lower limit at 0
Model abbr.: LL2.3()
Bechmark-Response (BMR): 10

BMCL: 0.015
BMC: 0.034
BMCU: 0.069

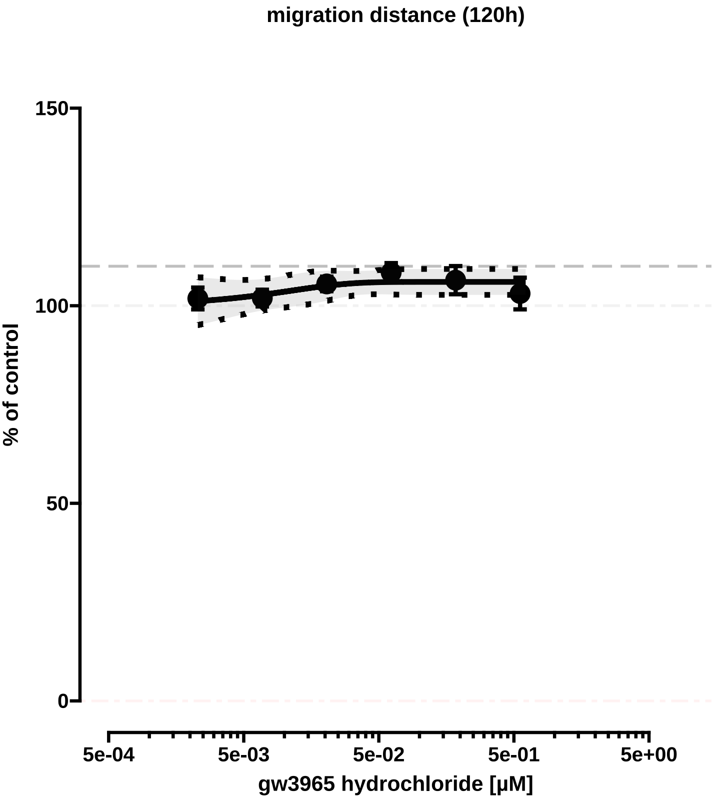

Model: Shifted exponential decay
Model abbr.: EXD.3()
Bechmark-Response (BMR): 10

BMCL: NA
BMC: NA
BMCU: NA

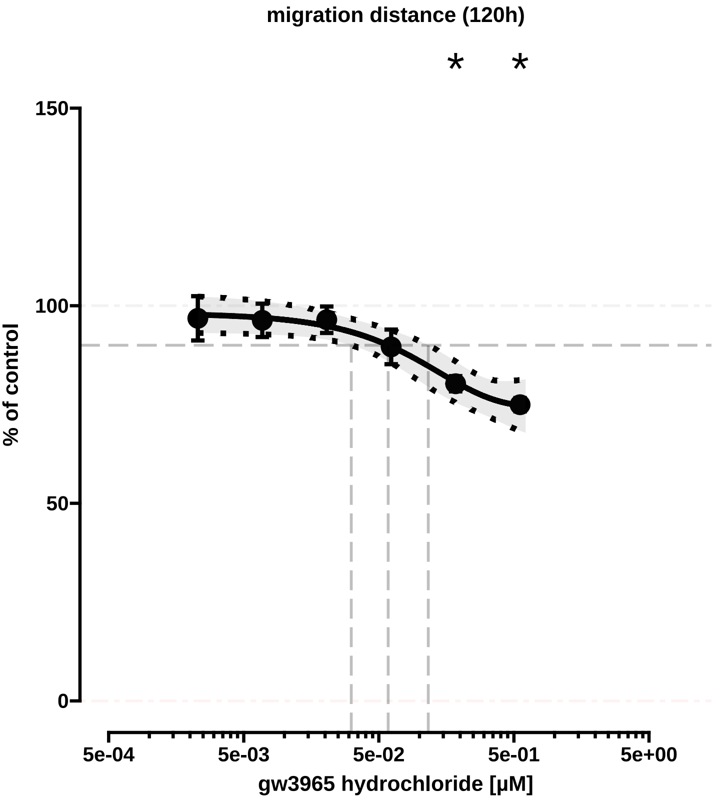

Model: Shifted exponential decay
Model abbr.: EXD.3()
Bechmark-Response (BMR): 10

BMCL: 0.031
BMC: 0.059
BMCU: 0.116

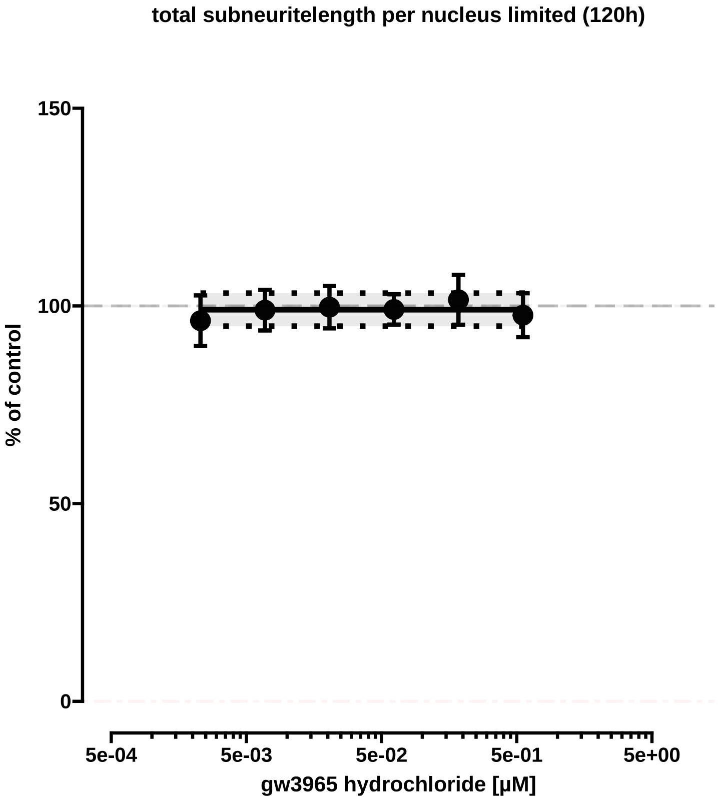

Model: 1-Parameter
Model abbr.: lm.1
Bechmark-Response (BMR): 20

BMCL: NA
BMC: NA
BMCU: NA

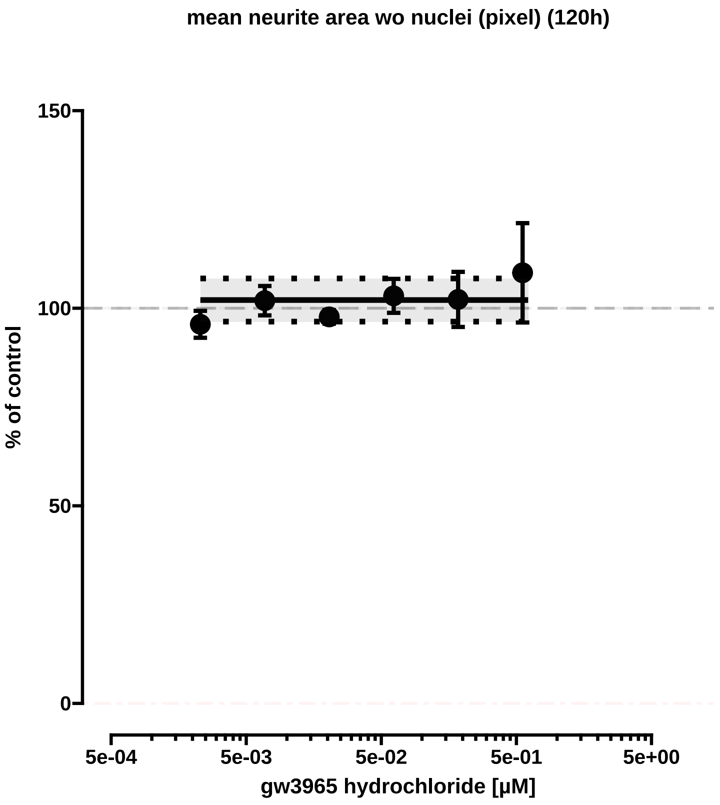

Model: 1-Parameter
Model abbr.: lm.1
Bechmark-Response (BMR): 20

BMCL: NA
BMC: NA
BMCU: NA

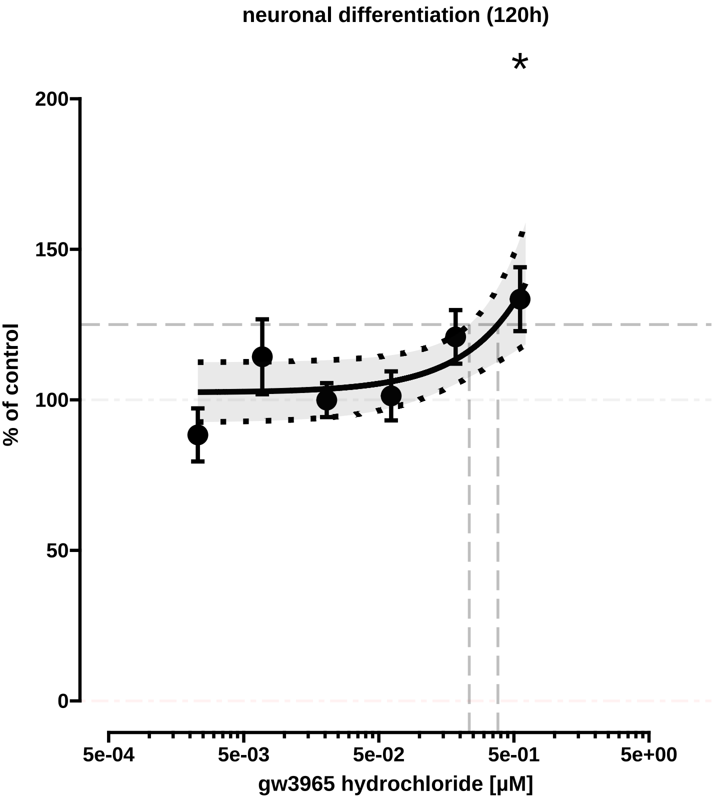

Model: Linear
Model abbr.: lm
Bechmark-Response (BMR): 25

BMCL: 0.234
BMC: 0.381
BMCU: NA

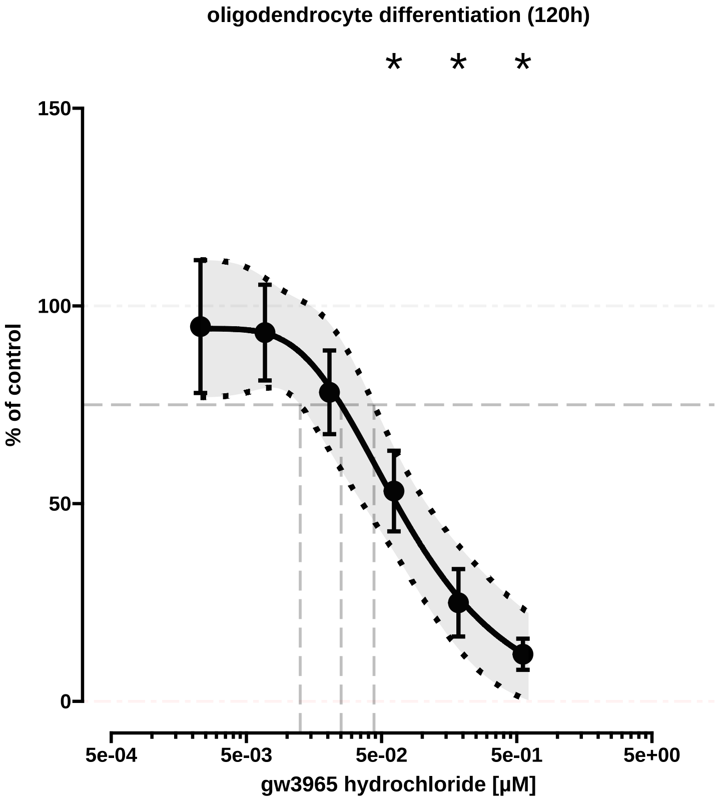

Model: Weibull (type 2) with lower limit at 0
Model abbr.: W2.3()
Bechmark-Response (BMR): 25

BMCL: 0.013
BMC: 0.025
BMCU: 0.044

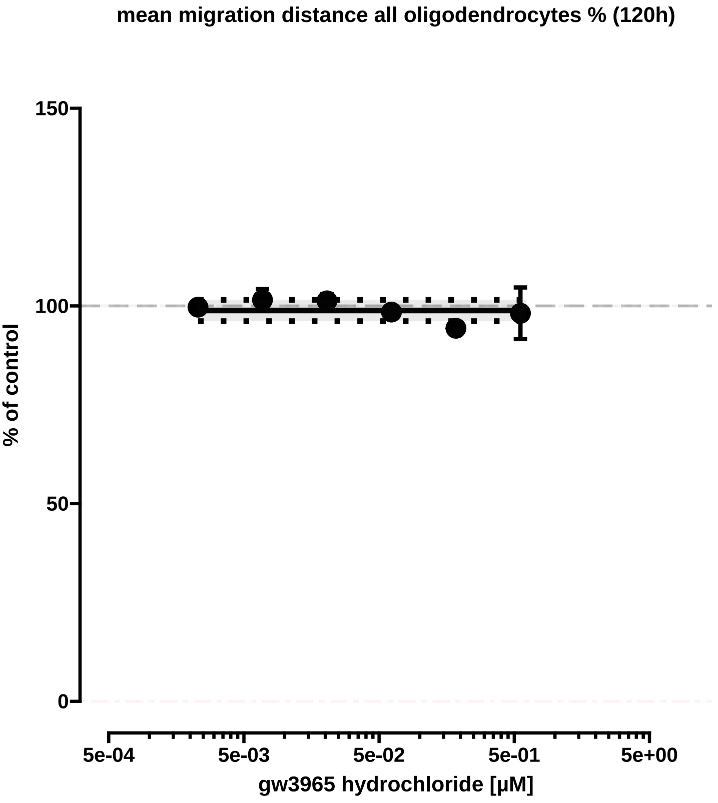

Model: 1-Parameter
Model abbr.: lm.1
Bechmark-Response (BMR): 10

BMCL: NA
BMC: NA
BMCU: NA

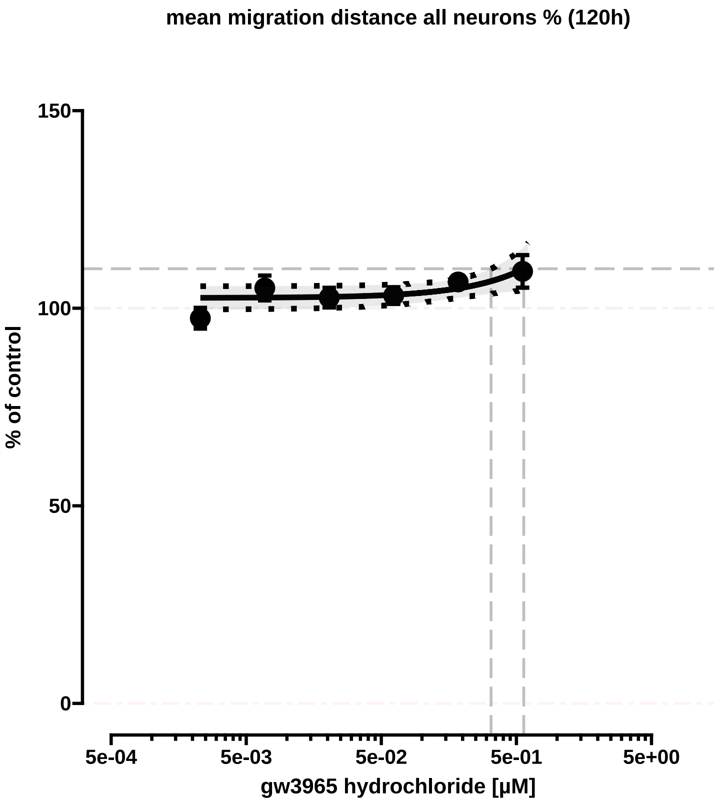

Model: Linear
Model abbr.: lm
Bechmark-Response (BMR): 10

BMCL: 0.324
BMC: 0.567
BMCU: NA

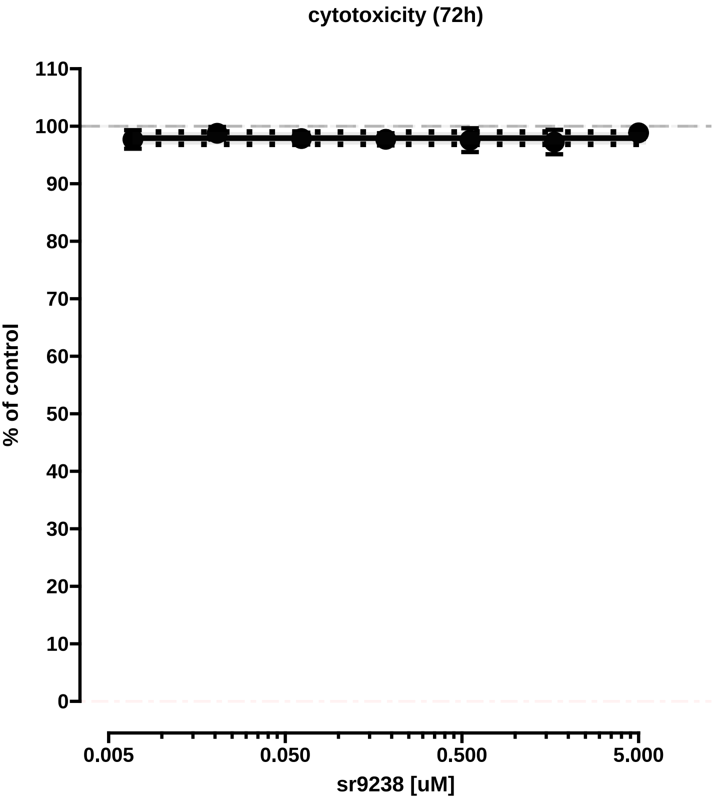

Model: 1-Parameter
Model abbr.: lm.1
Bechmark-Response (BMR): 10

BMCL: NA
BMC: NA
BMCU: NA

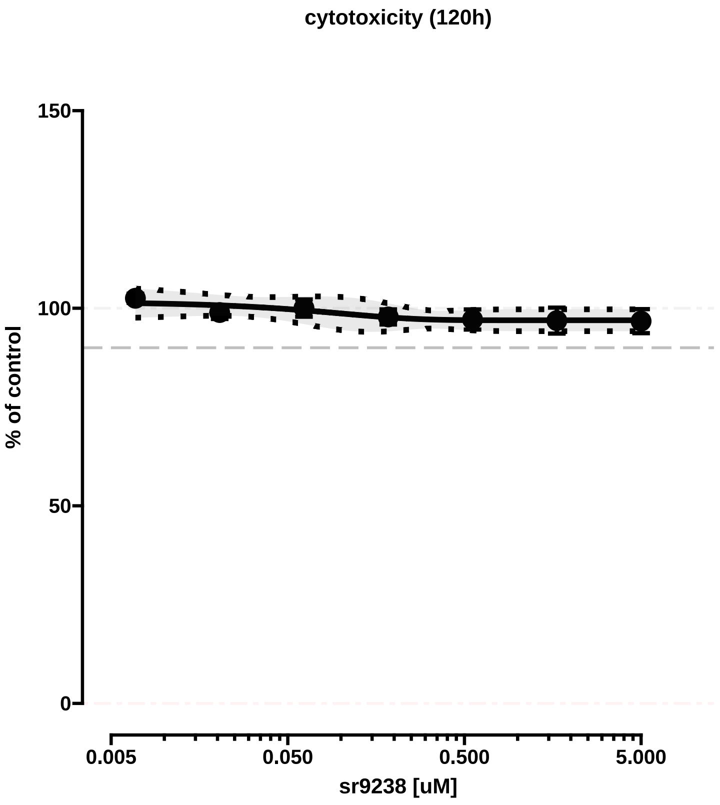

Model: Shifted exponential decay
Model abbr.: EXD.3()
Bechmark-Response (BMR): 10

BMCL: NA
BMC: NA
BMCU: NA

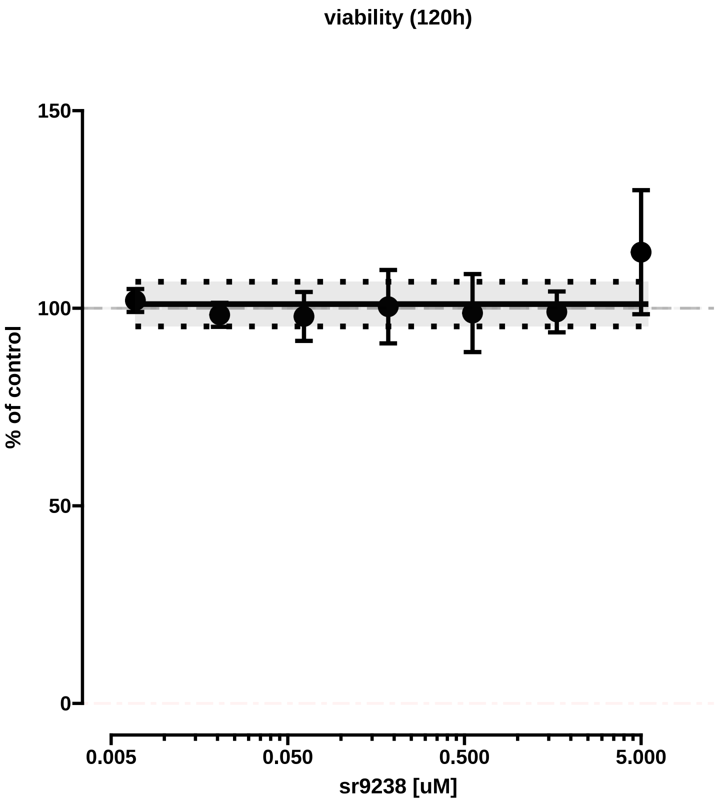

Model: 1-Parameter
Model abbr.: lm.1
Bechmark-Response (BMR): 20

BMCL: NA
BMC: NA
BMCU: NA

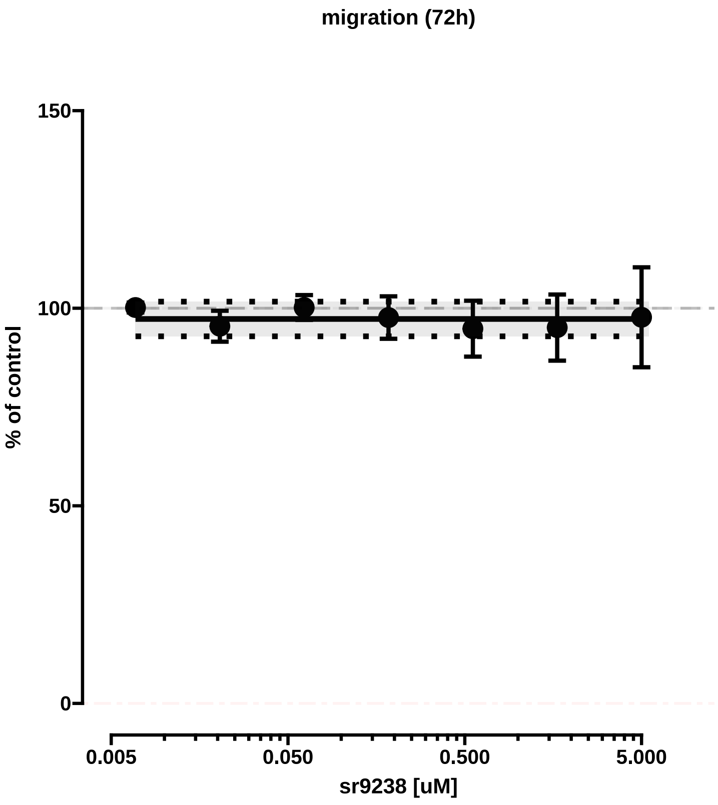

Model: 1-Parameter
Model abbr.: lm.1
Bechmark-Response (BMR): 10

BMCL: NA
BMC: NA
BMCU: NA

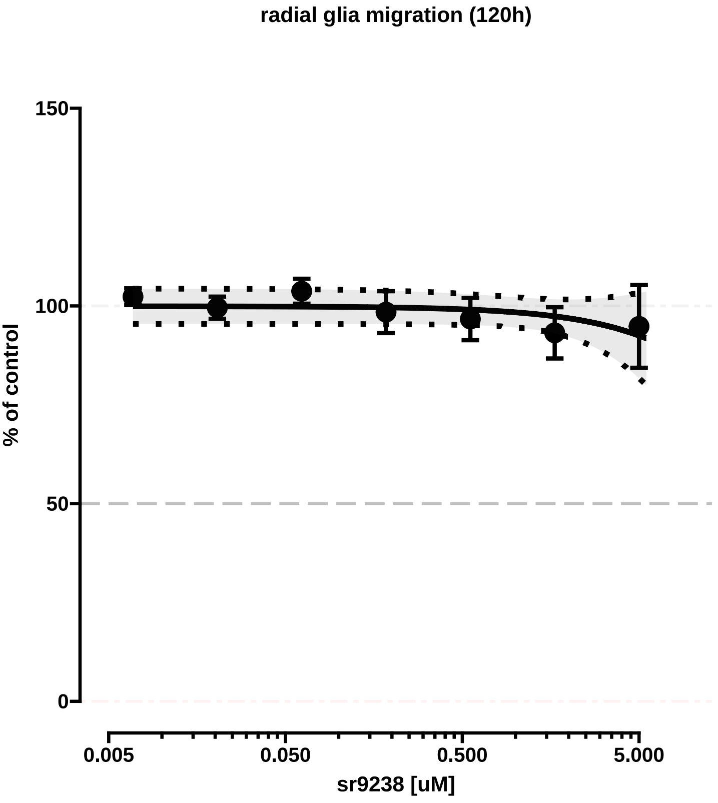

Model: Exponential decay with lower limit at 0
Model abbr.: EXD.2()
Bechmark-Response (BMR): 50

BMCL: NA
BMC: NA
BMCU: NA

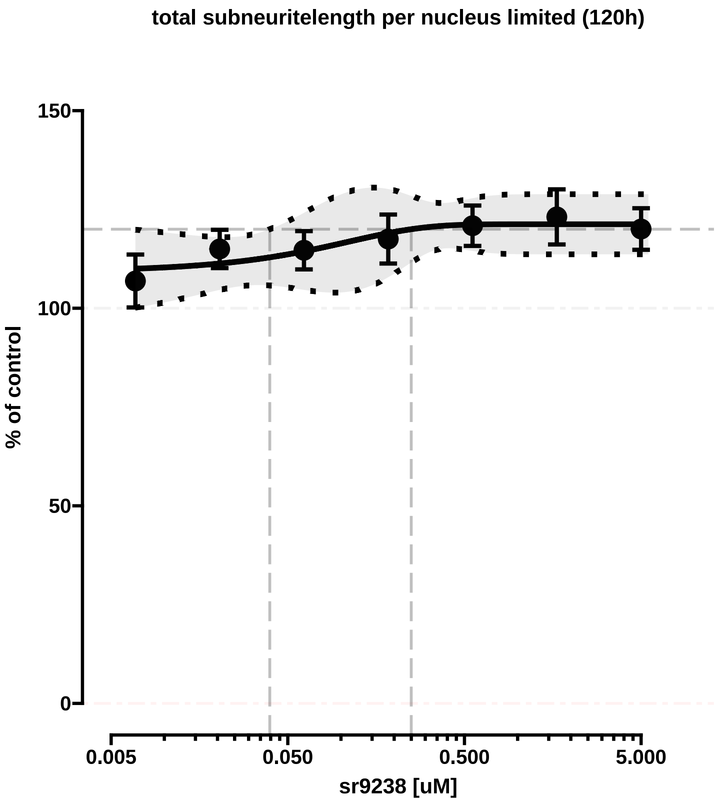

Model: Shifted exponential decay
Model abbr.: EXD.3()
Bechmark-Response (BMR): 20

BMCL: 0.03953
BMC: 0.24984
BMCU: NA

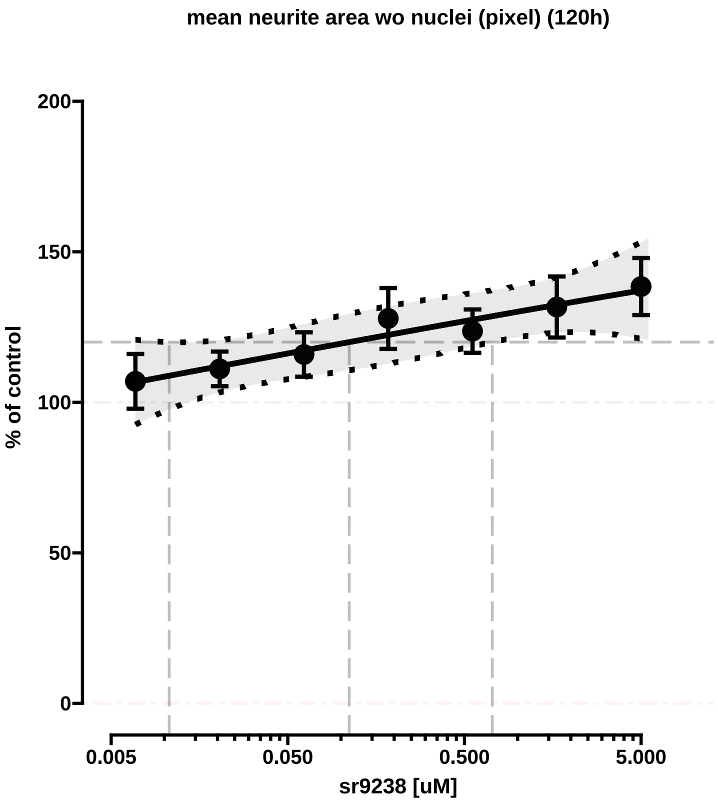

Model: Log-logistic (log(ED50) as parameter) with lower limit at 0
Model abbr.: LL2.3()
Bechmark-Response (BMR): 20

BMCL: 0.01066
BMC: 0.11138
BMCU: 0.719

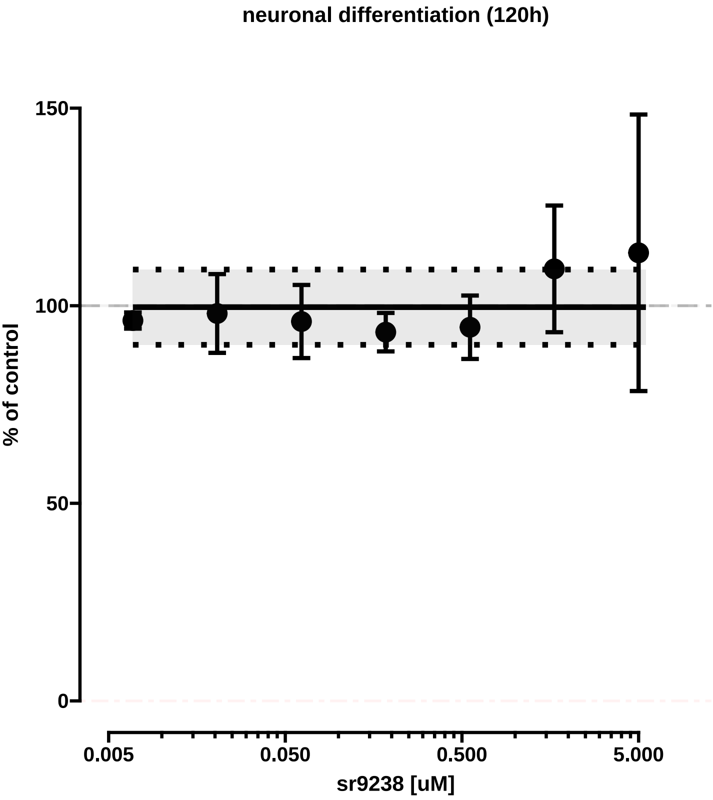

Model: 1-Parameter
Model abbr.: lm.1
Bechmark-Response (BMR): 25

BMCL: NA
BMC: NA
BMCU: NA

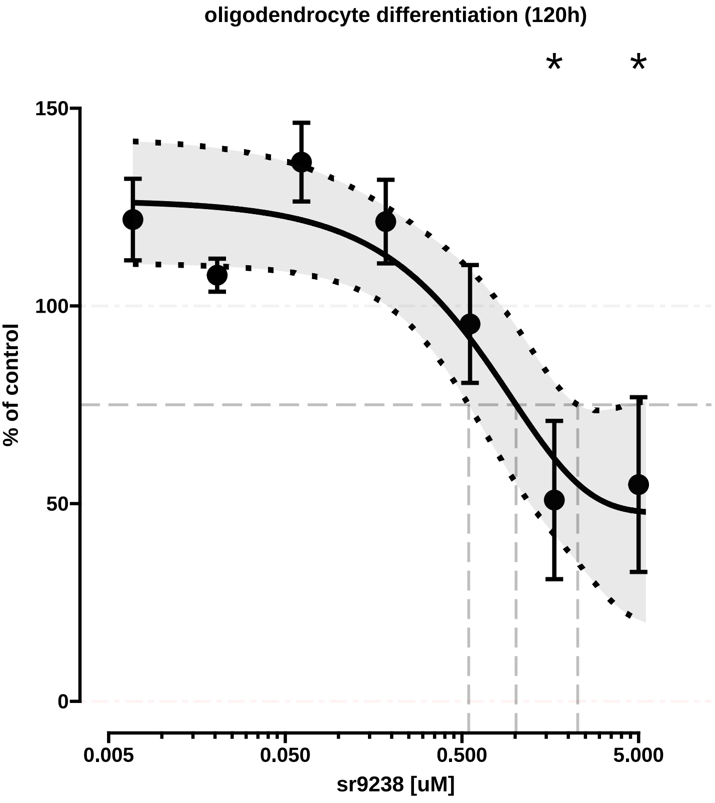

Model: Shifted exponential decay
Model abbr.: EXD.3()
Bechmark-Response (BMR): 25

BMCL: 0.54598
BMC: 1.01218
BMCU: 2.261

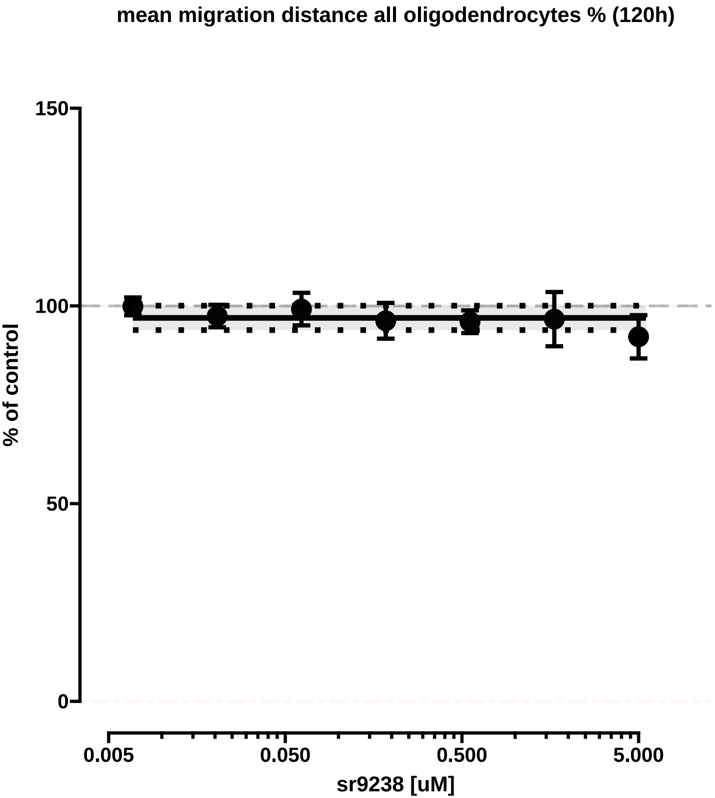

Model: 1-Parameter
Model abbr.: lm.1
Bechmark-Response (BMR): 10

BMCL: NA
BMC: NA
BMCU: NA

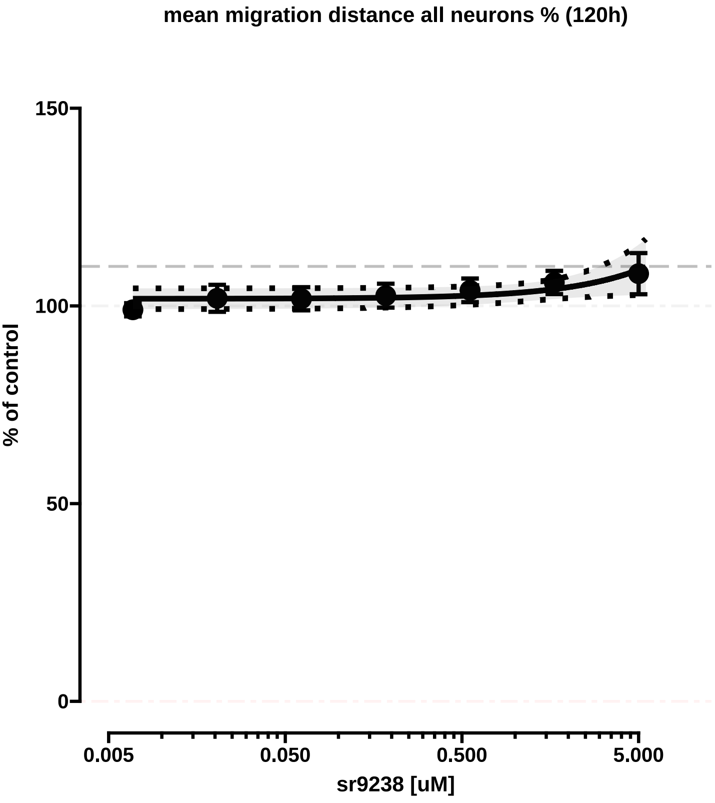

Model: Linear
Model abbr.: lm
Bechmark-Response (BMR): 10

BMCL: NA
BMC: NA
BMCU: NA

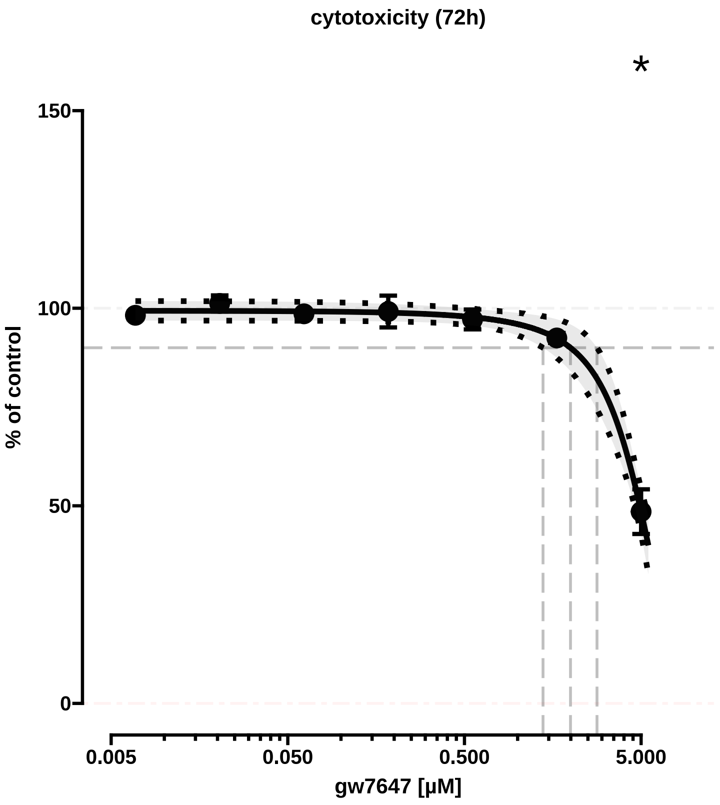

Model: Logistic (ED50 as parameter) with lower limit fixed at 0
Model abbr.: L.3()
Bechmark-Response (BMR): 10

BMCL: 1.393
BMC: 1.992
BMCU: 2.814

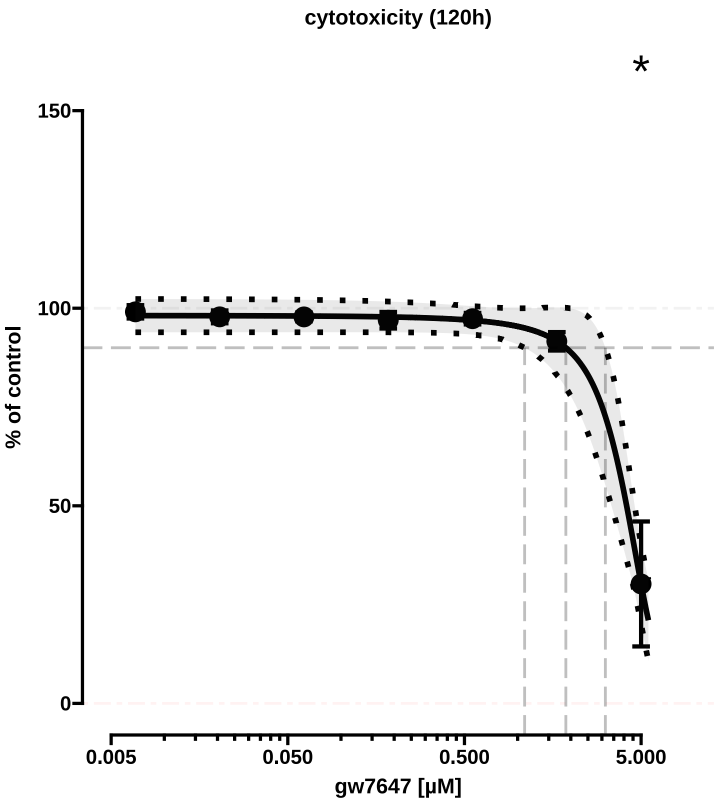

Model: Logistic (ED50 as parameter) with lower limit fixed at 0
Model abbr.: L.3()
Bechmark-Response (BMR): 10

BMCL: 1.095
BMC: 1.875
BMCU: 3.138

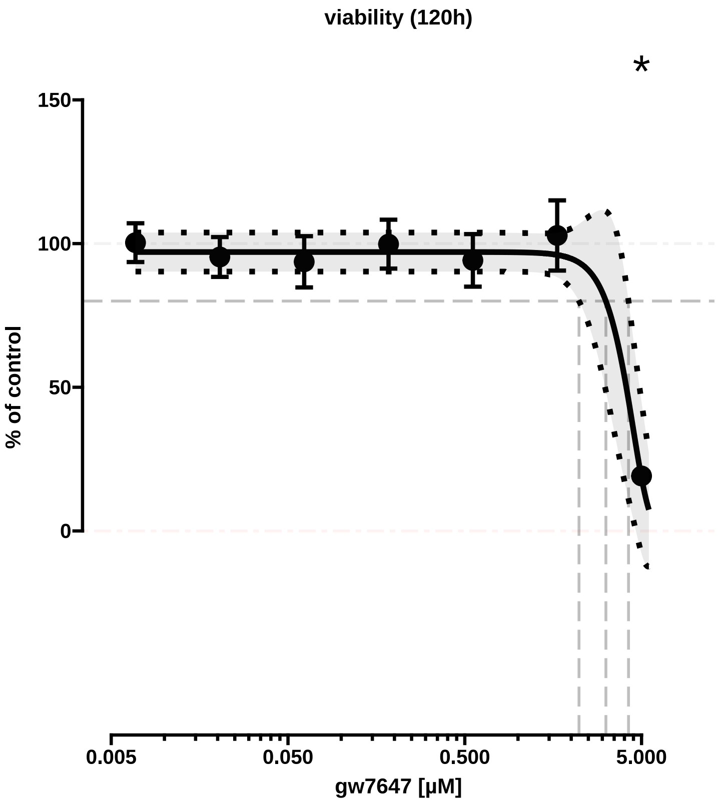

Model: Weibull (type 1) with lower limit at 0
Model abbr.: W1.3()
Bechmark-Response (BMR): 20

BMCL: 2.213
BMC: 3.142
BMCU: 4.22

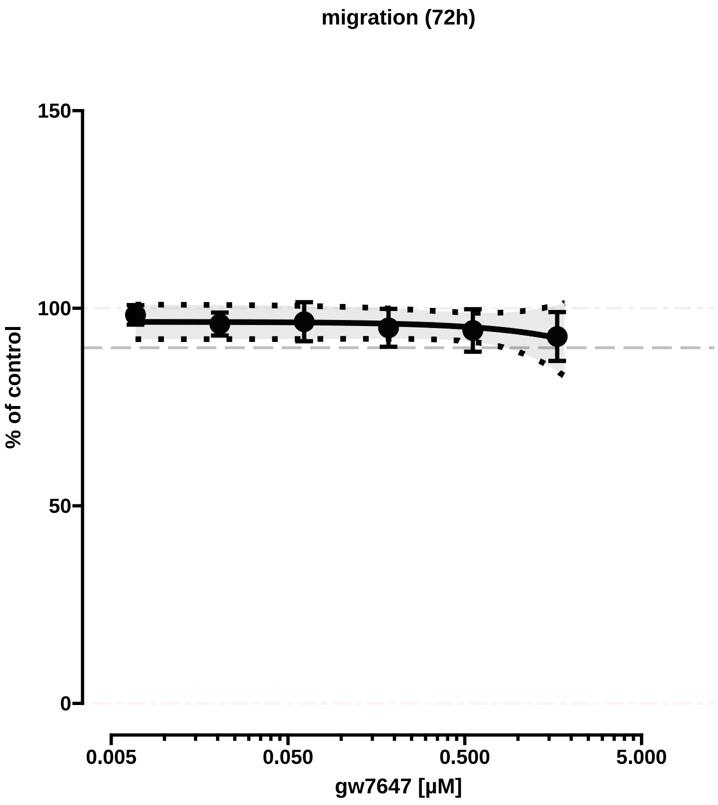

Model: Exponential decay with lower limit at 0
Model abbr.: EXD.2()
Bechmark-Response (BMR): 10

BMCL: NA
BMC: NA
BMCU: NA

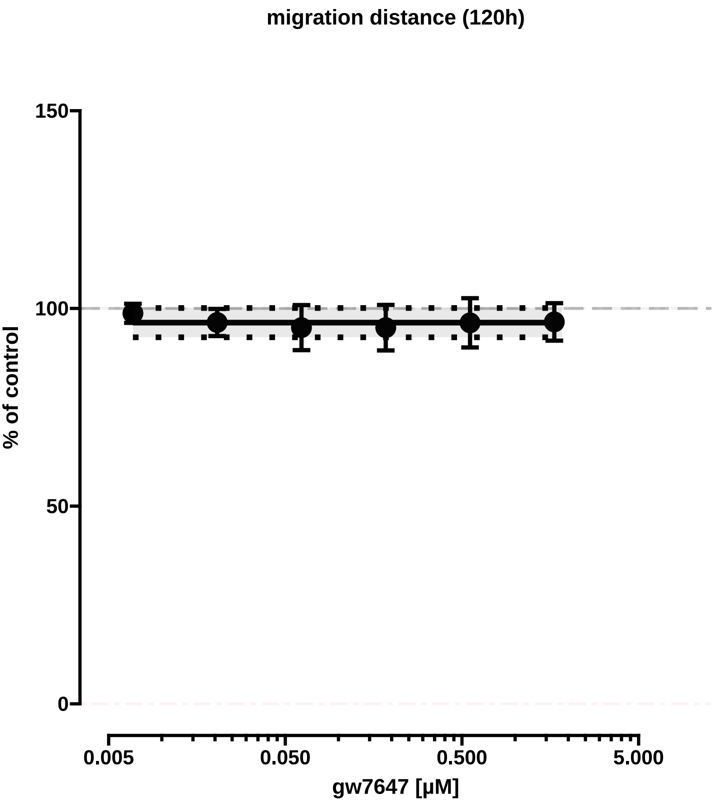

Model: 1-Parameter
Model abbr.: lm.1
Bechmark-Response (BMR): 10

BMCL: NA
BMC: NA
BMCU: NA

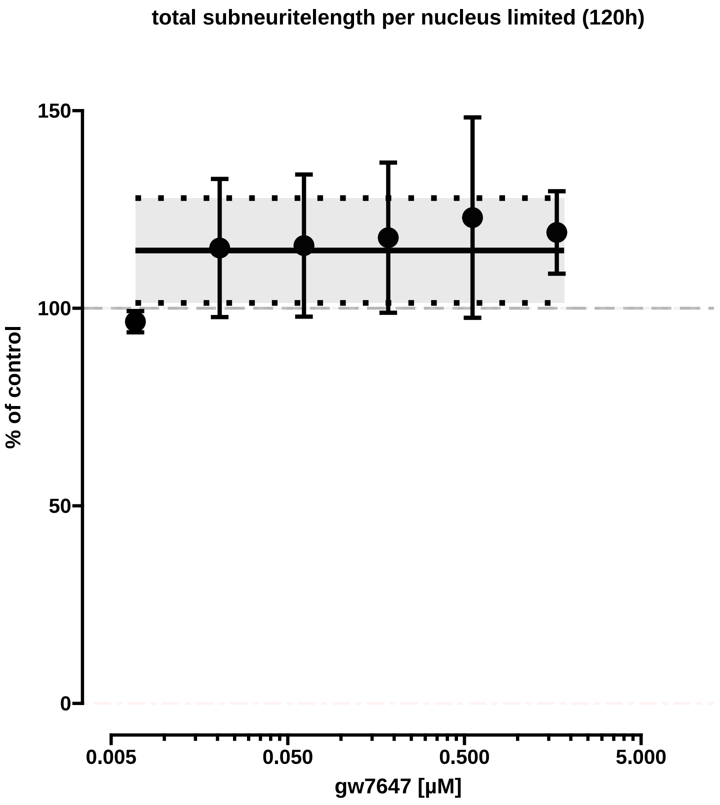

Model: 1-Parameter
Model abbr.: lm.1
Bechmark-Response (BMR): 20

BMCL: NA
BMC: NA
BMCU: NA

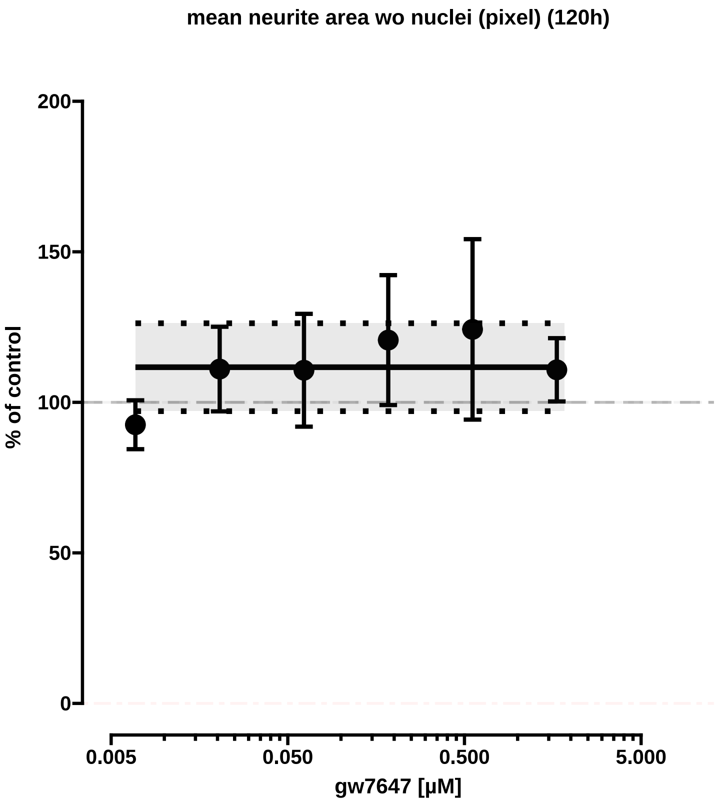

Model: 1-Parameter
Model abbr.: lm.1
Bechmark-Response (BMR): 20

BMCL: NA
BMC: NA
BMCU: NA

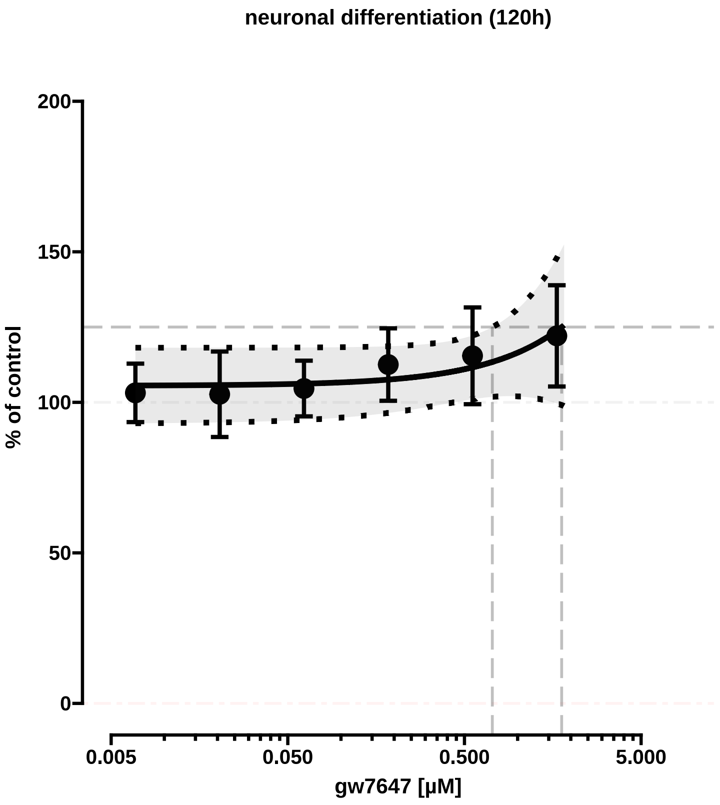

Model: Linear
Model abbr.: lm
Bechmark-Response (BMR): 25

BMCL: 0.719
BMC: 1.775
BMCU: NA

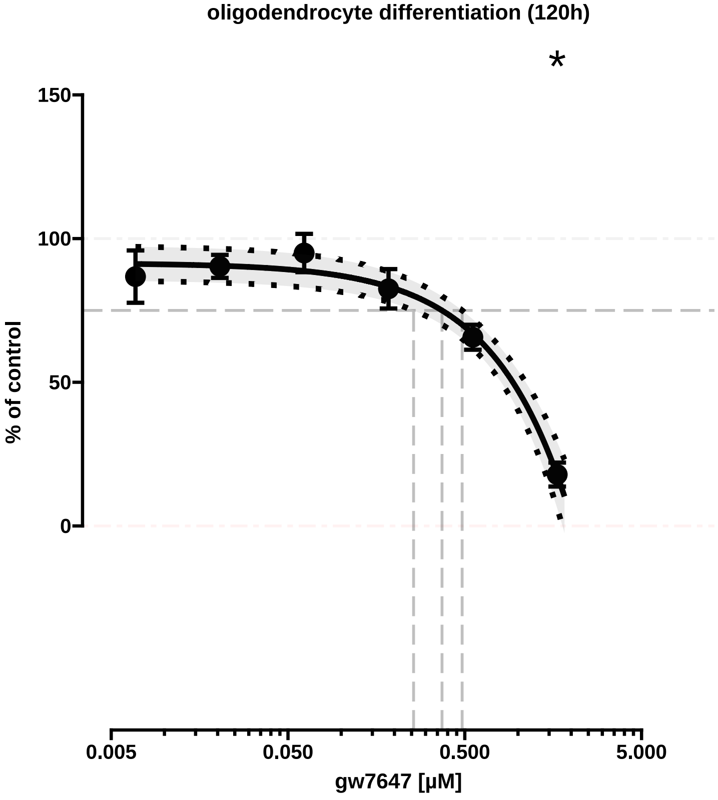

Model: Linear
Model abbr.: lm
Bechmark-Response (BMR): 25

BMCL: 0.257
BMC: 0.372
BMCU: 0.483

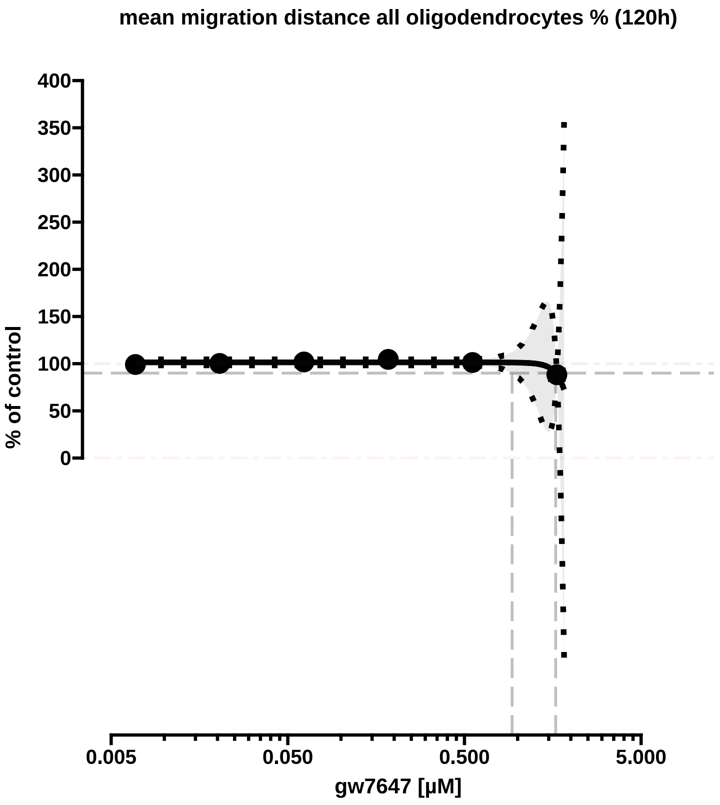

Model: Logistic (ED50 as parameter) with lower limit fixed at 0
Model abbr.: L.3()
Bechmark-Response (BMR): 10

BMCL: 0.93
BMC: 1.642
BMCU: NA

mean migration distance all neurons % (120h)

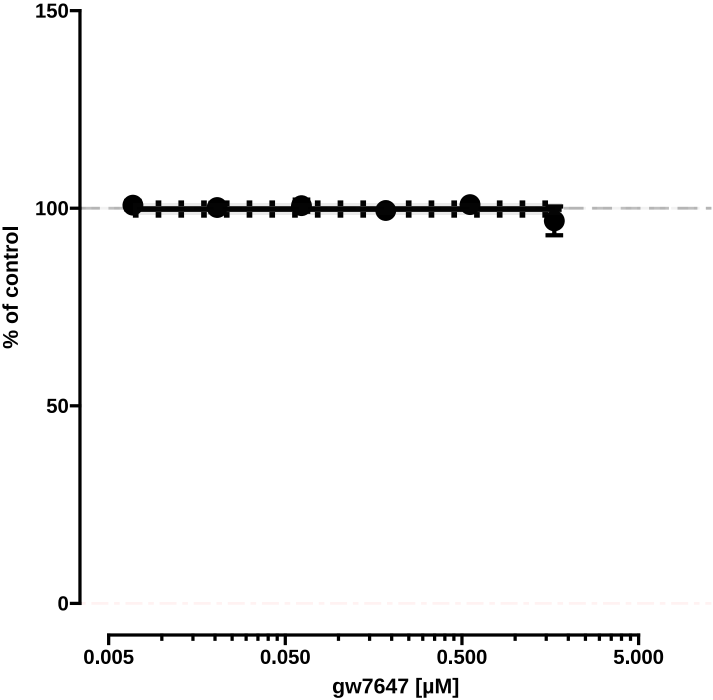

Model: 1-Parameter
Model abbr.: lm.1
Bechmark-Response (BMR): 10

BMCL: NA
BMC: NA
BMCU: NA

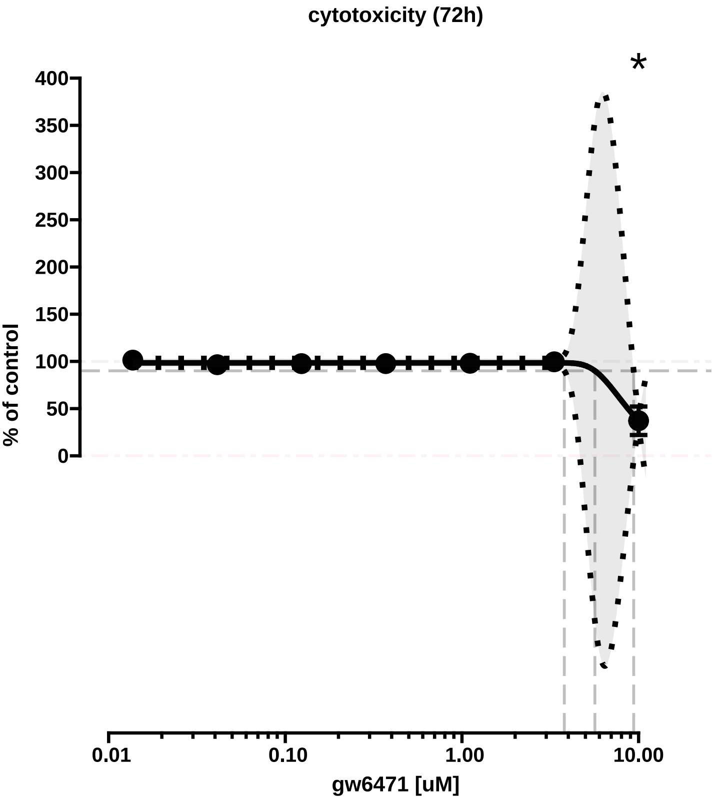

Model: Weibull (type 2) with lower limit at 0
Model abbr.: W2.3()
Bechmark-Response (BMR): 10

BMCL: 3.798
BMC: 5.658
BMCU: 9.381

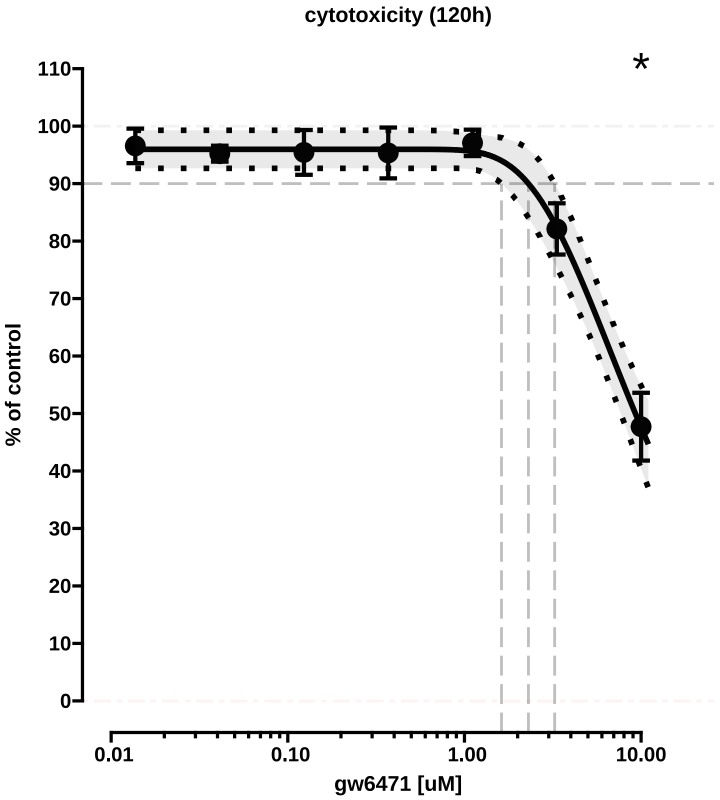

Model: Weibull (type 2) with lower limit at 0
Model abbr.: W2.3()
Bechmark-Response (BMR): 10

BMCL: 1.621
BMC: 2.303
BMCU: 3.241

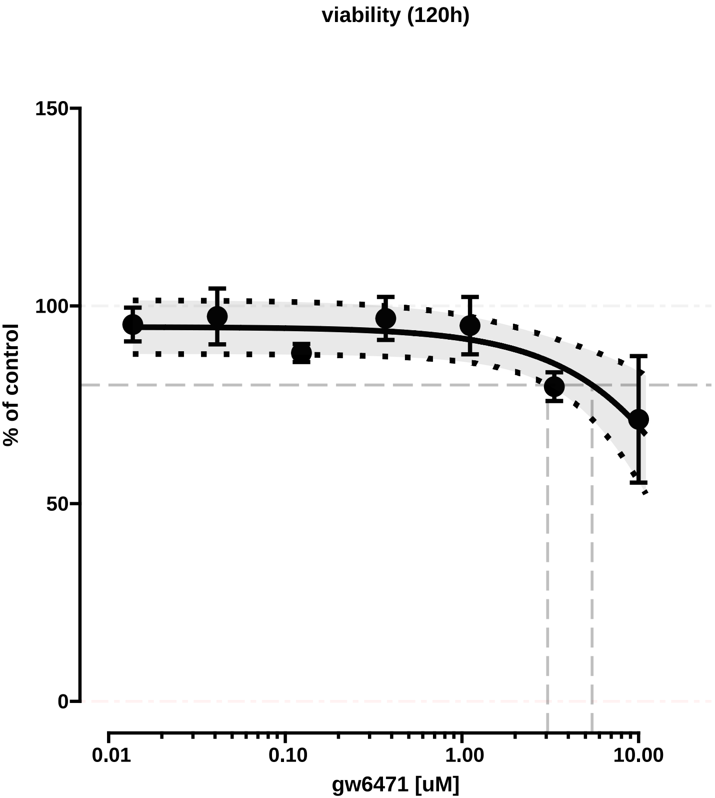

Model: Exponential decay with lower limit at 0
Model abbr.: EXD.2()
Bechmark-Response (BMR): 20

BMCL: 3.056
BMC: 5.453
BMCU: NA

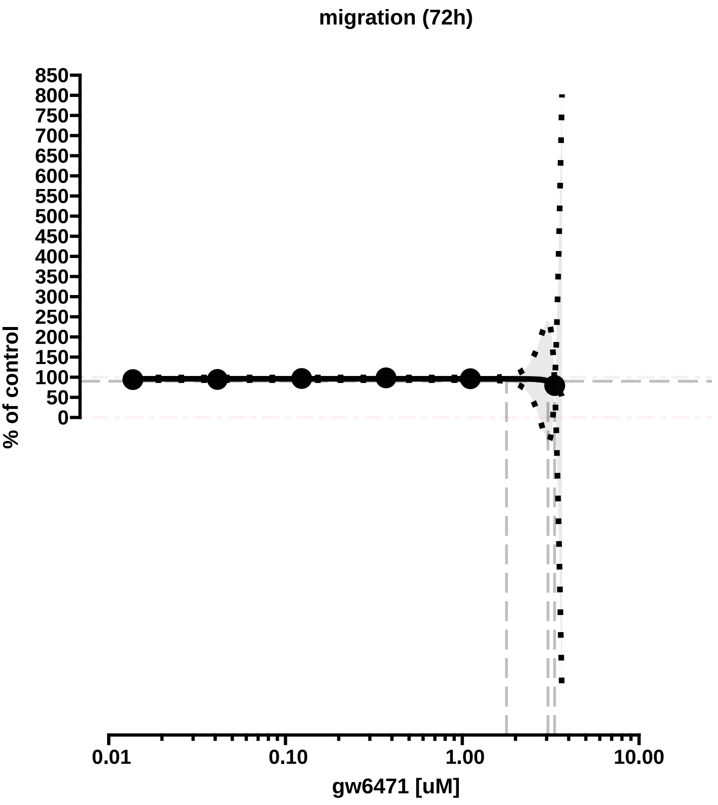

Model: Logistic (ED50 as parameter) with lower limit fixed at 0
Model abbr.: L.3()
Bechmark-Response (BMR): 10

BMCL: 1.78
BMC: 3.054
BMCU: 3.329

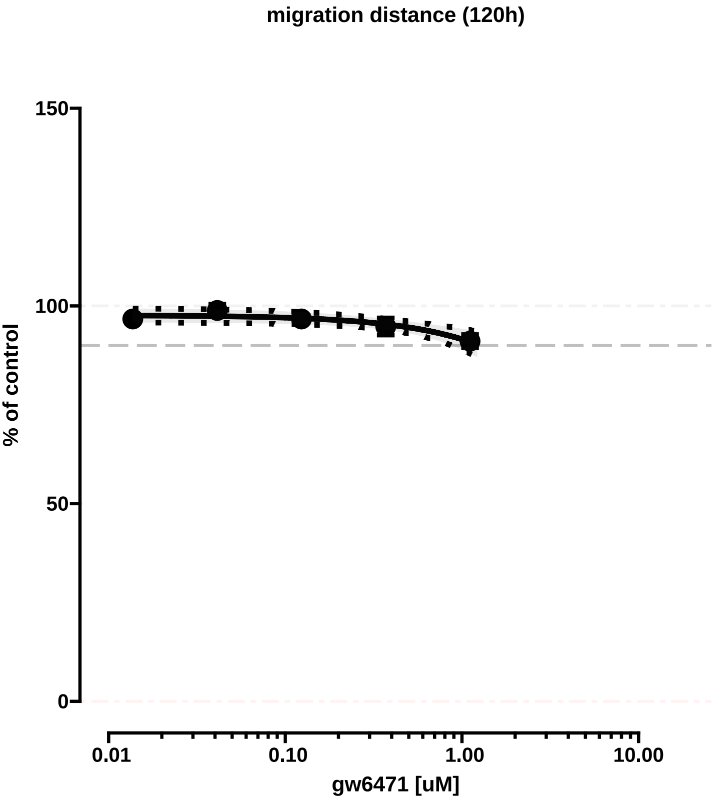

Model: Exponential decay with lower limit at 0
Model abbr.: EXD.2()
Bechmark-Response (BMR): 10

BMCL: NA
BMC: NA
BMCU: NA

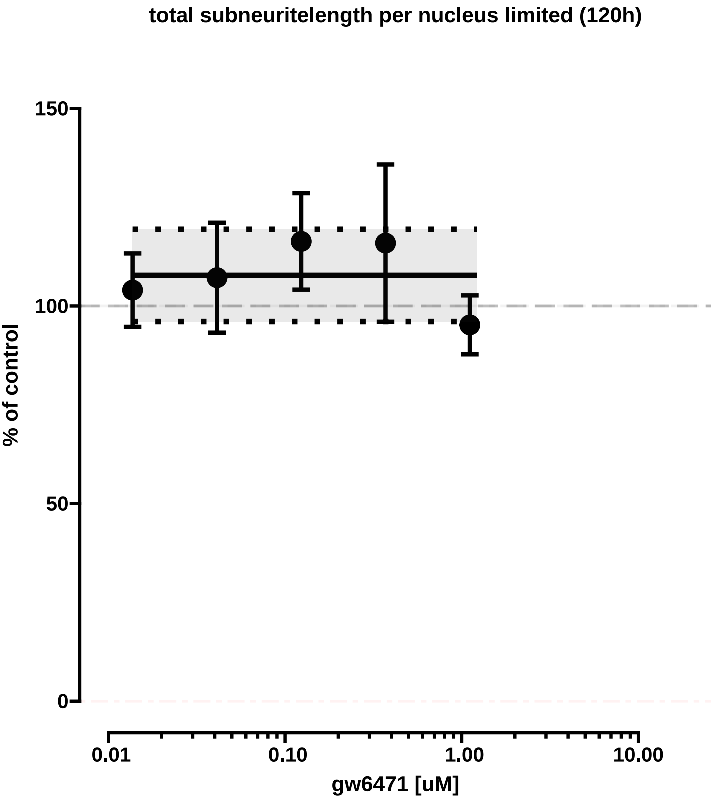

Model: 1-Parameter
Model abbr.: lm.1
Bechmark-Response (BMR): 20

BMCL: NA
BMC: NA
BMCU: NA

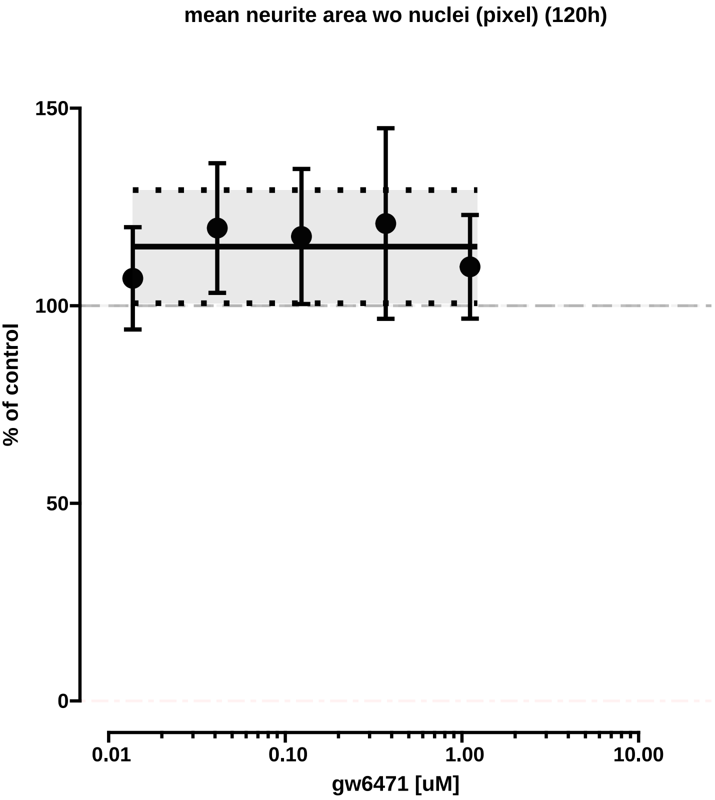

Model: 1-Parameter
Model abbr.: lm.1
Bechmark-Response (BMR): 20

BMCL: NA
BMC: NA
BMCU: NA

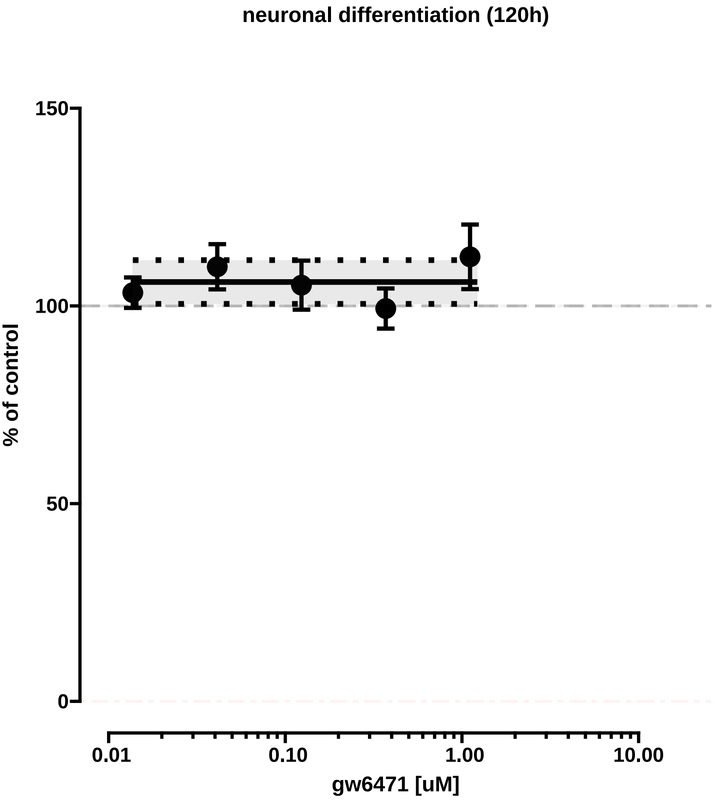

Model: 1-Parameter
Model abbr.: lm.1
Bechmark-Response (BMR): 25

BMCL: NA
BMC: NA
BMCU: NA

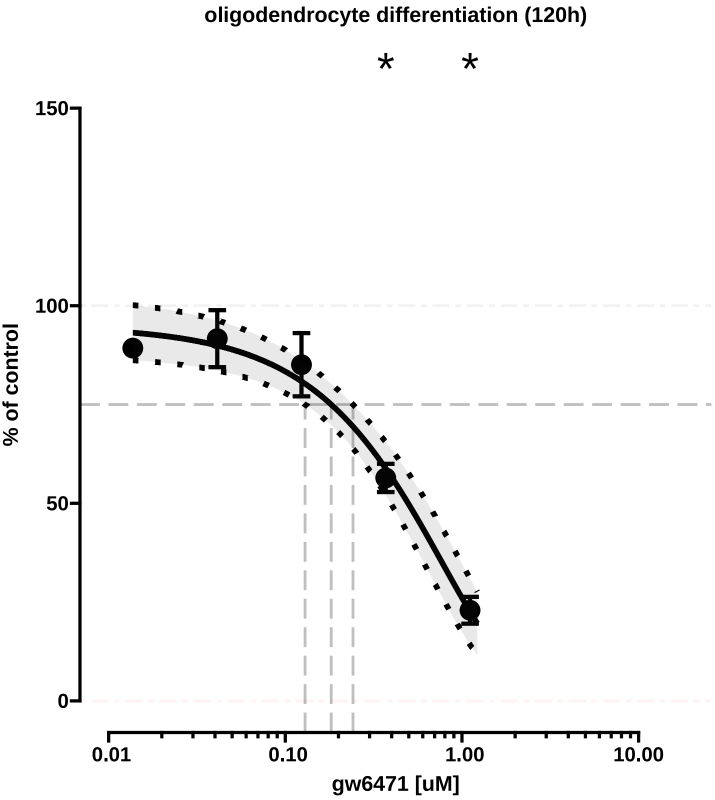

Model: Exponential decay with lower limit at 0
Model abbr.: EXD.2()
Bechmark-Response (BMR): 25

BMCL: 0.129
BMC: 0.182
BMCU: 0.242

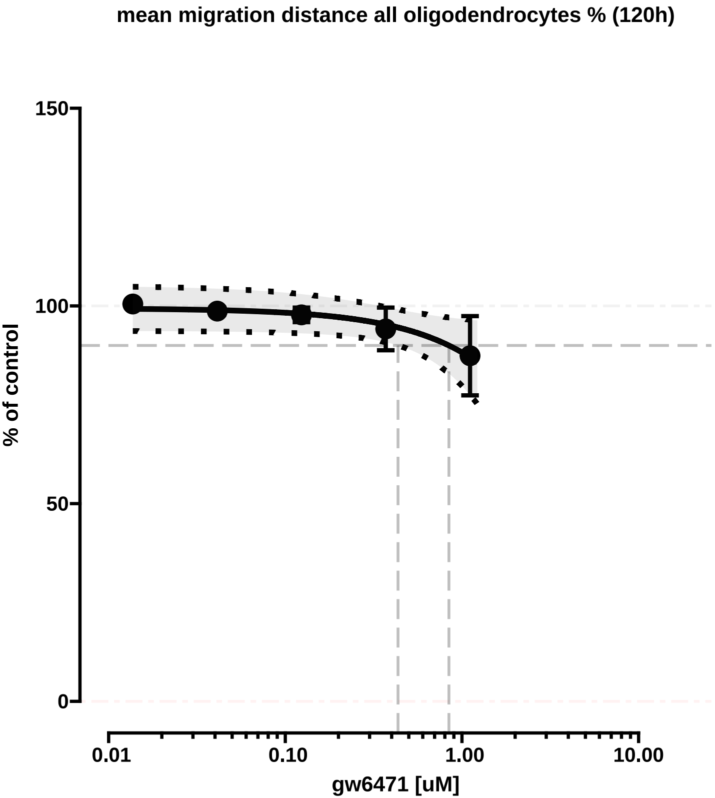

Model: Linear
Model abbr.: lm
Bechmark-Response (BMR): 10

BMCL: 0.435
BMC: 0.844
BMCU: NA

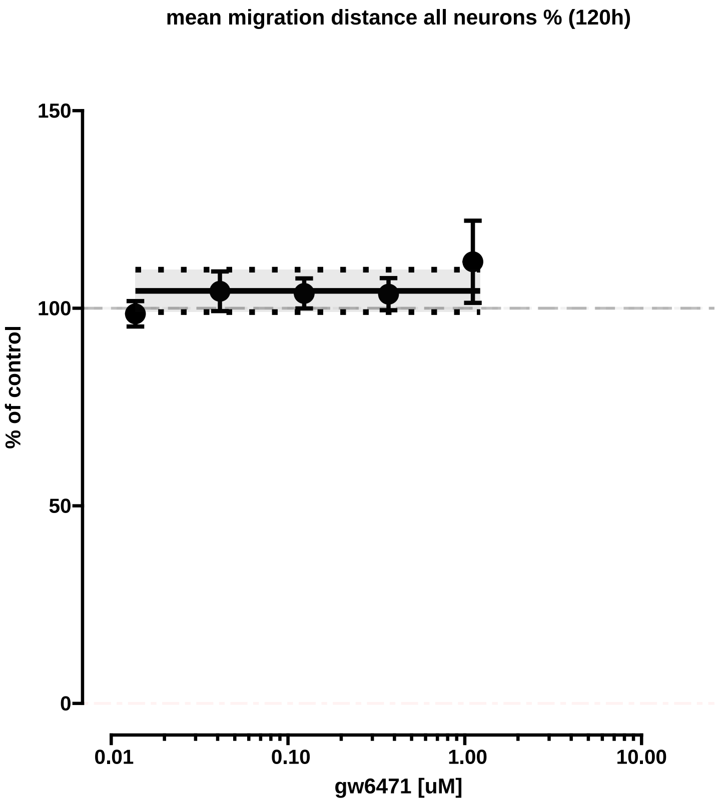

Model: 1-Parameter
Model abbr.: lm.1
Bechmark-Response (BMR): 10

BMCL: NA
BMC: NA
BMCU: NA

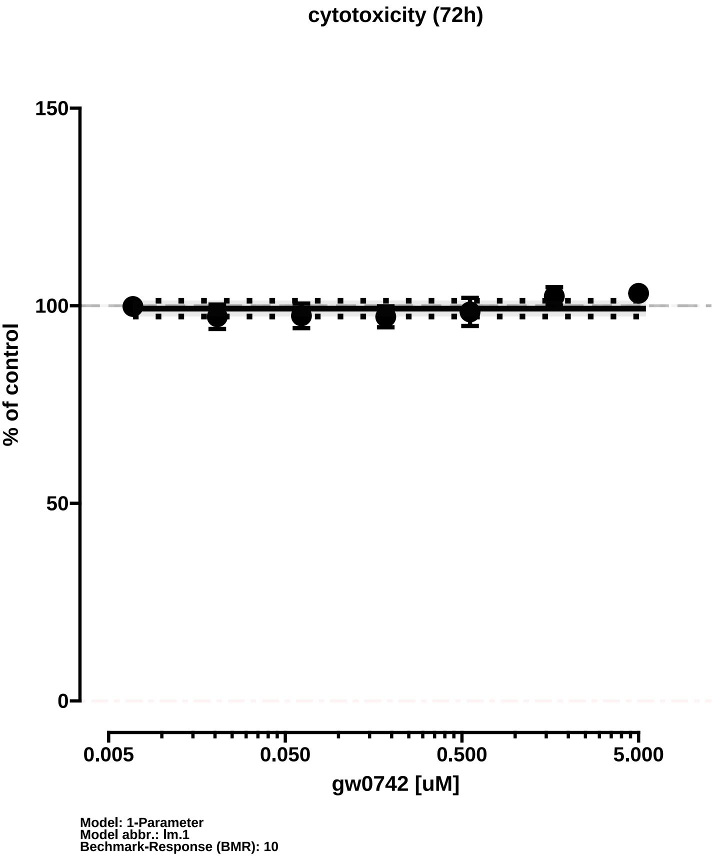

Model: 1-Parameter
Model abbr.: lm.1
Bechmark-Response (BMR): 10

BMCL: NA
BMC: NA
BMCU: NA

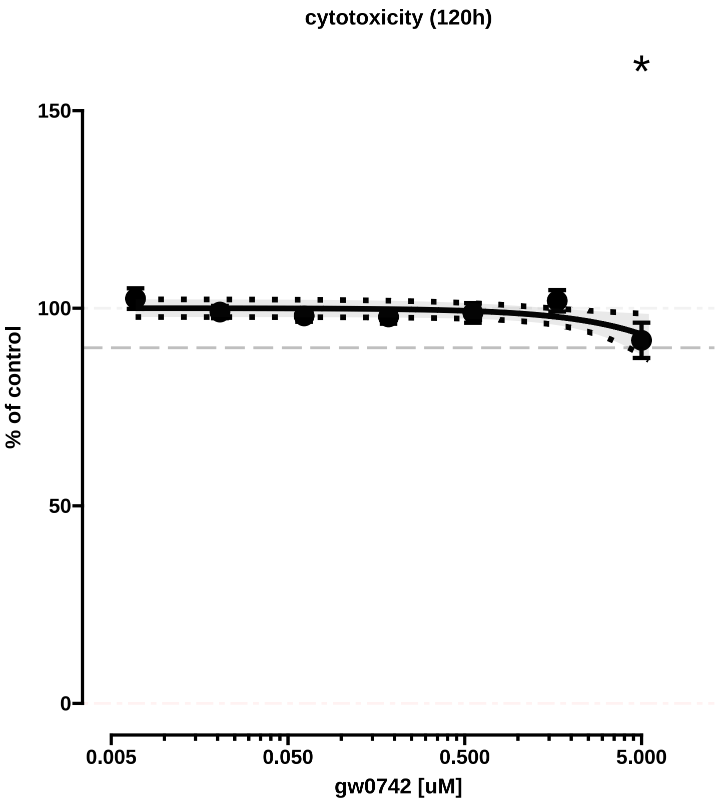

Model: Linear
Model abbr.: lm
Bechmark-Response (BMR): 10

BMCL: NA
BMC: NA
BMCU: NA

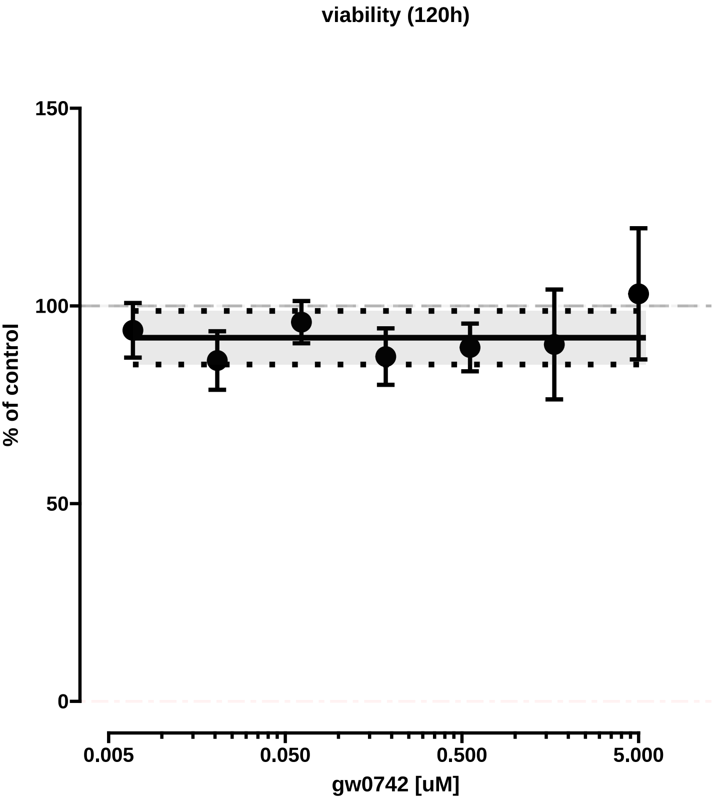

Model: 1-Parameter
Model abbr.: lm.1
Bechmark-Response (BMR): 20

BMCL: NA
BMC: NA
BMCU: NA

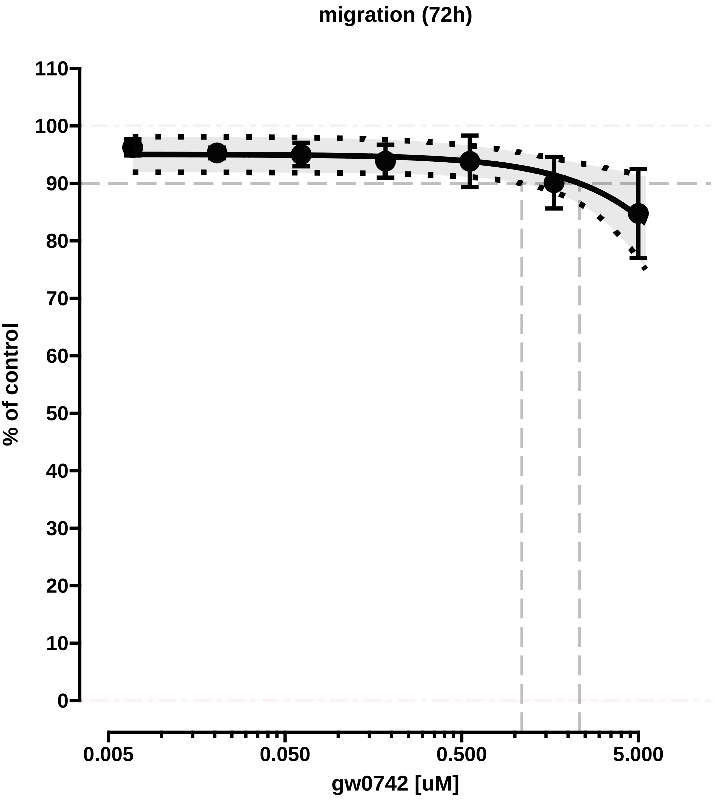

Model: Linear
Model abbr.: lm
Bechmark-Response (BMR): 10

BMCL: 1.095
BMC: 2.323
BMCU: NA

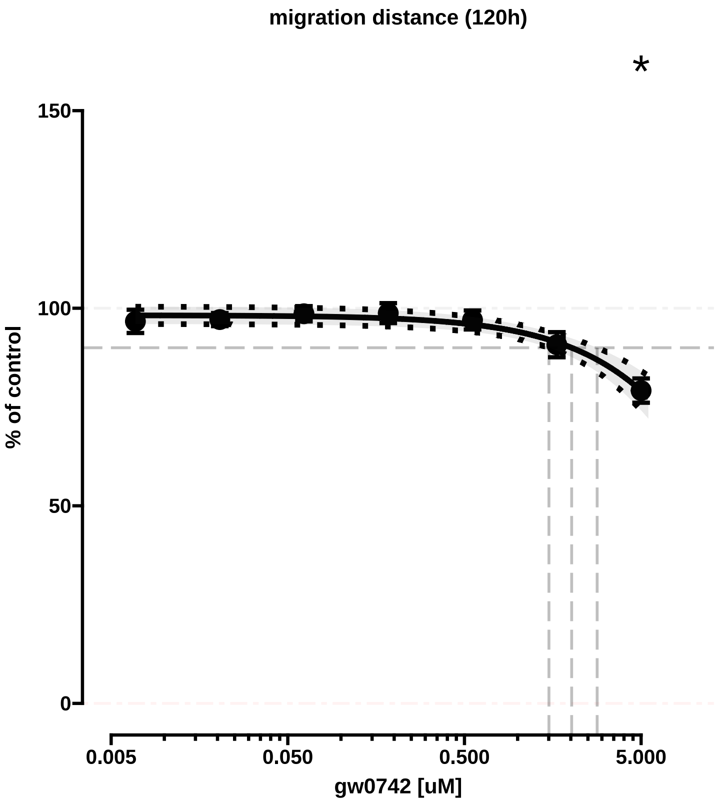

Model: Exponential decay with lower limit at 0
Model abbr.: EXD.2()
Bechmark-Response (BMR): 10

BMCL: 1.504
BMC: 2.022
BMCU: 2.82

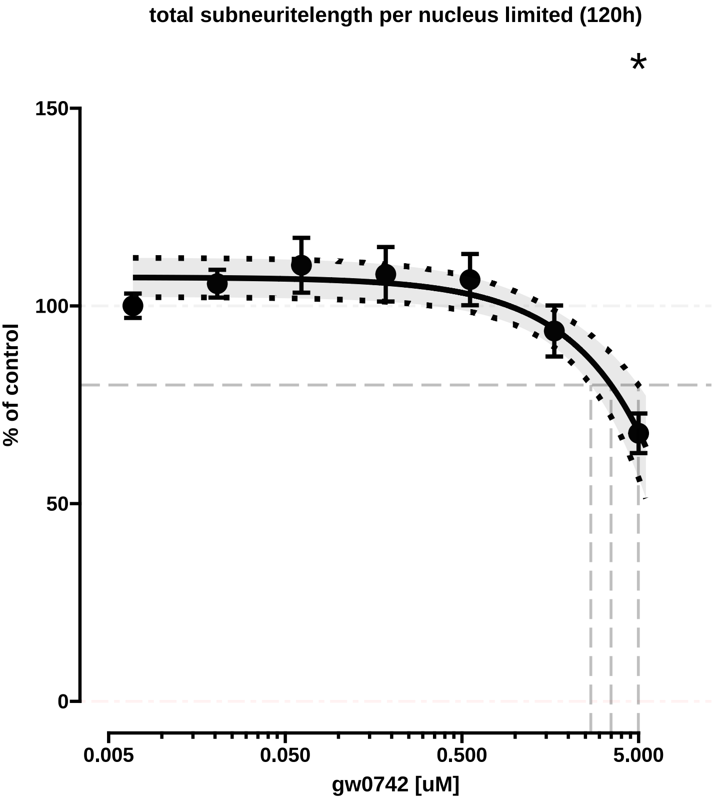

Model: Linear
Model abbr.: lm
Bechmark-Response (BMR): 20

BMCL: 2.682
BMC: 3.49
BMCU: 4.985

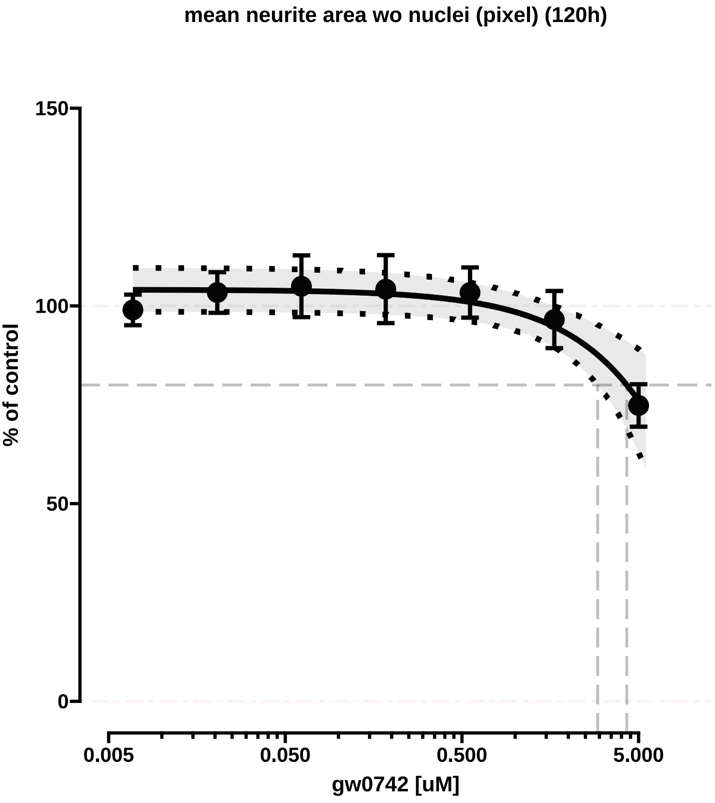

Model: Linear
Model abbr.: lm
Bechmark-Response (BMR): 20

BMCL: 2.933
BMC: 4.286
BMCU: NA

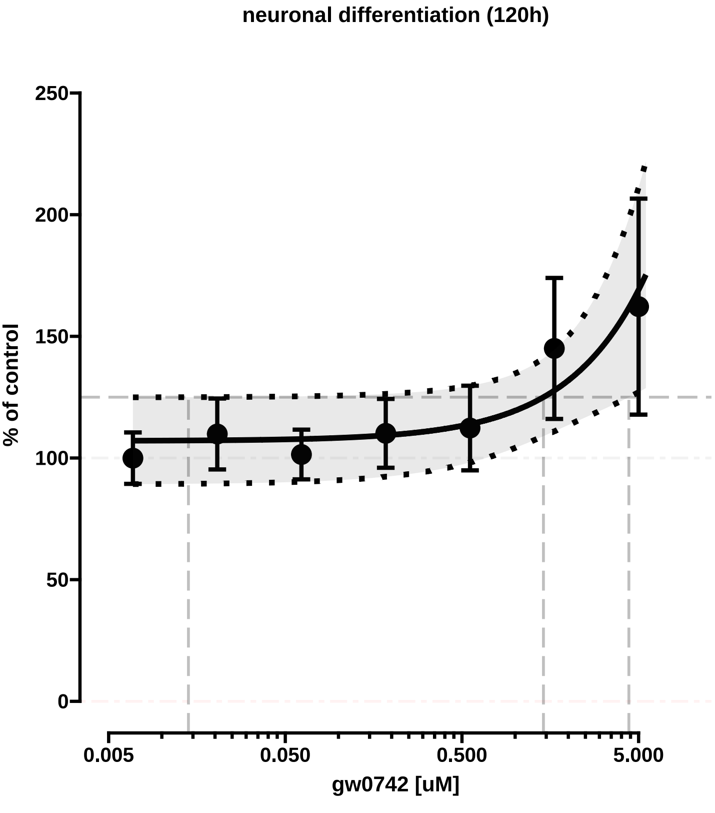

Model: Linear
Model abbr.: lm
Bechmark-Response (BMR): 25

BMCL: 0.014
BMC: 1.447
BMCU: 4.405

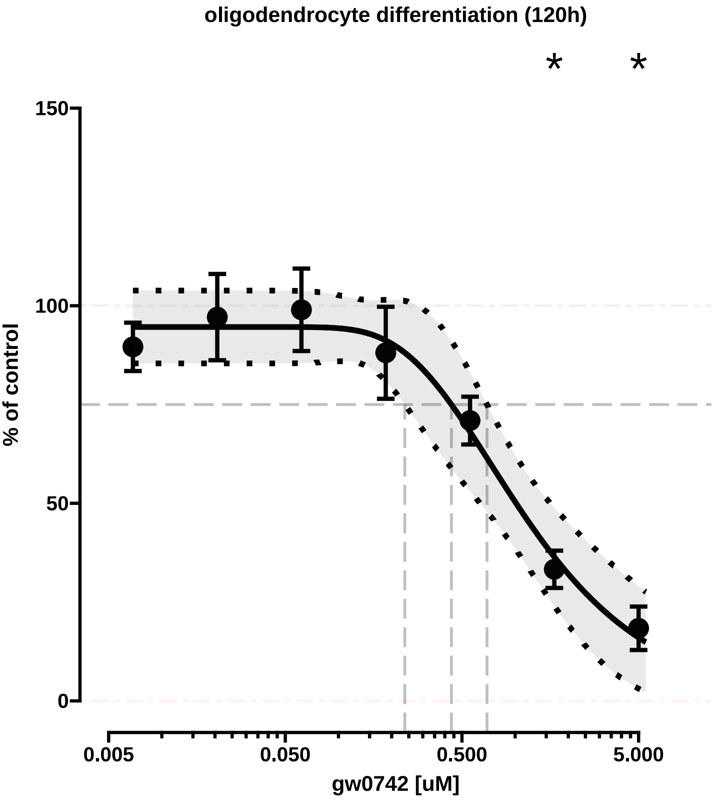

Model: Weibull (type 2) with lower limit at 0
Model abbr.: W2.3()
Bechmark-Response (BMR): 25

BMCL: 0.237
BMC: 0.436
BMCU: 0.693

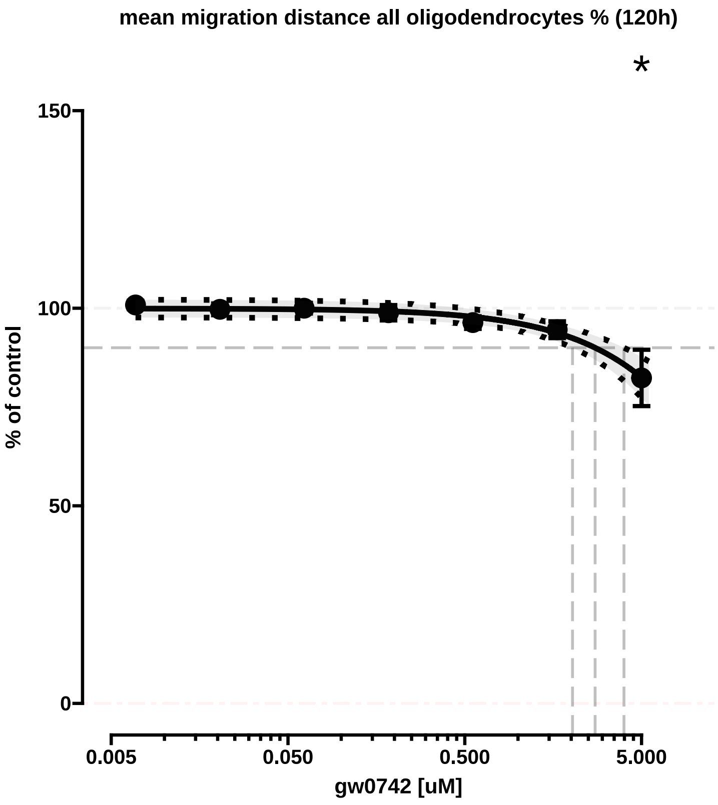

Model: Exponential decay with lower limit at 0
Model abbr.: EXD.2()
Bechmark-Response (BMR): 10

BMCL: 2.035
BMC: 2.731
BMCU: 3.977

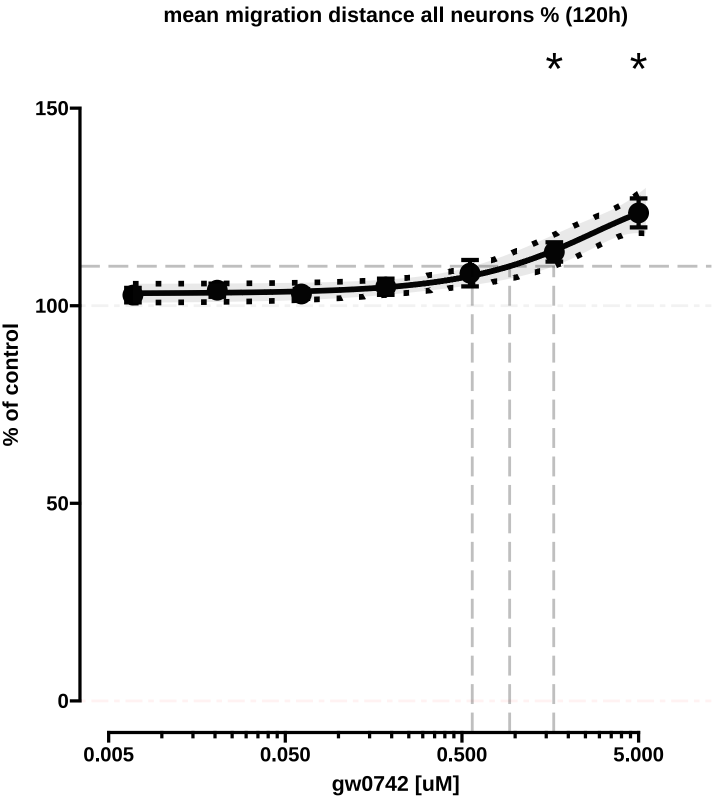

Model: Shifted exponential decay
Model abbr.: EXD.3()
Bechmark-Response (BMR): 10

BMCL: 0.572
BMC: 0.931
BMCU: 1.654

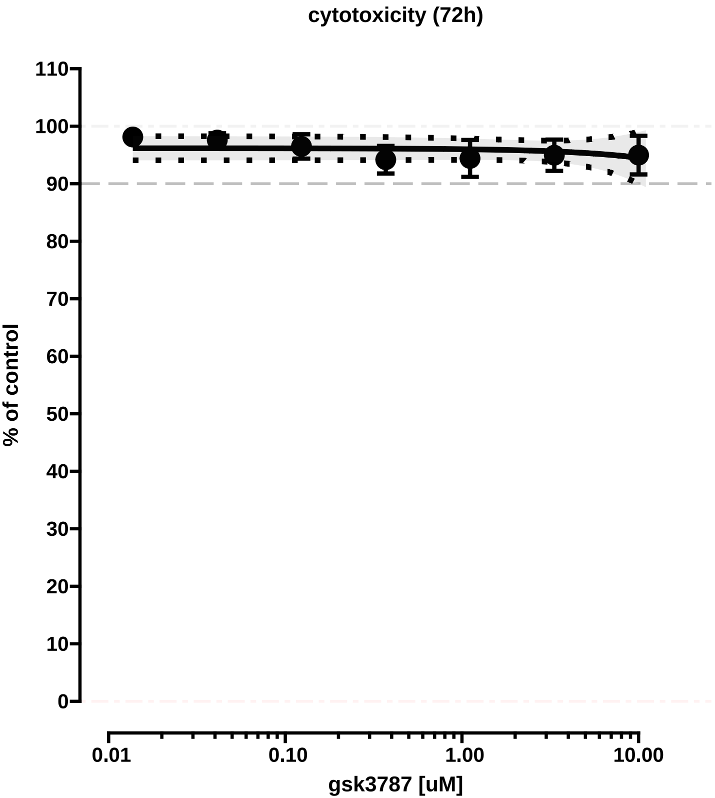

Model: Exponential decay with lower limit at 0
Model abbr.: EXD.2()
Bechmark-Response (BMR): 10

BMCL: NA
BMC: NA
BMCU: NA

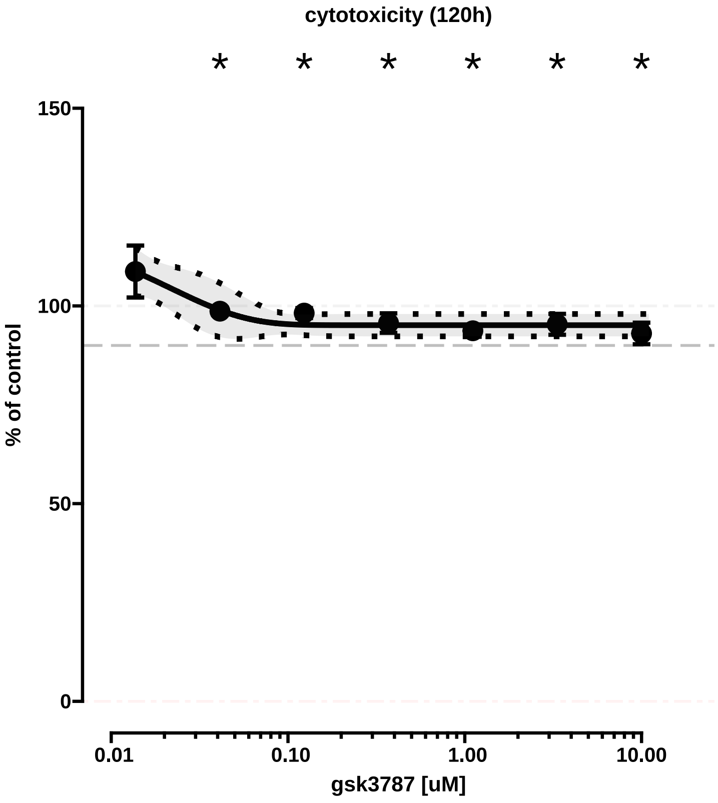

Model: Shifted exponential decay
Model abbr.: EXD.3()
Bechmark-Response (BMR): 10

BMCL: NA
BMC: NA
BMCU: NA

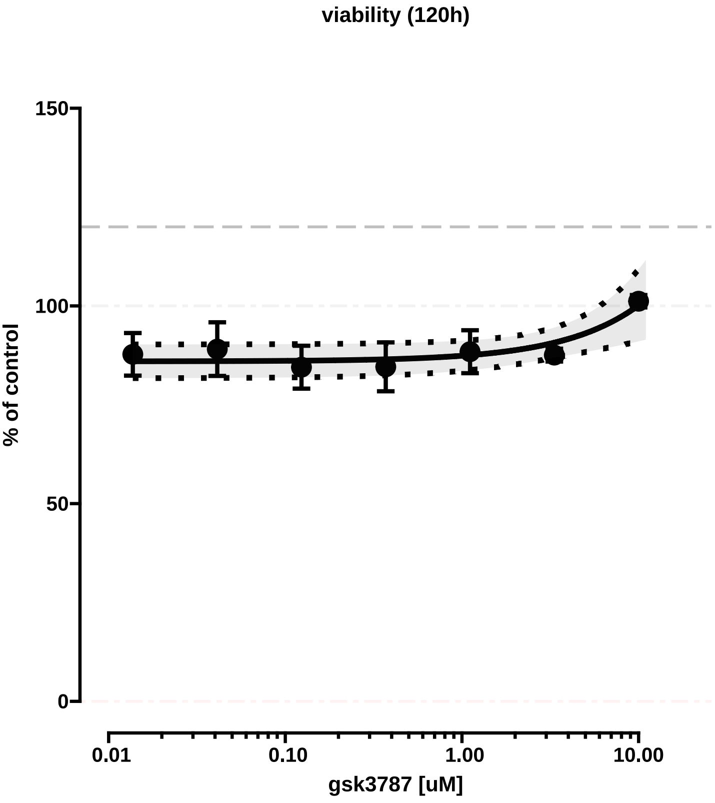

Model: Linear
Model abbr.: lm
Bechmark-Response (BMR): 20

BMCL: NA
BMC: NA
BMCU: NA

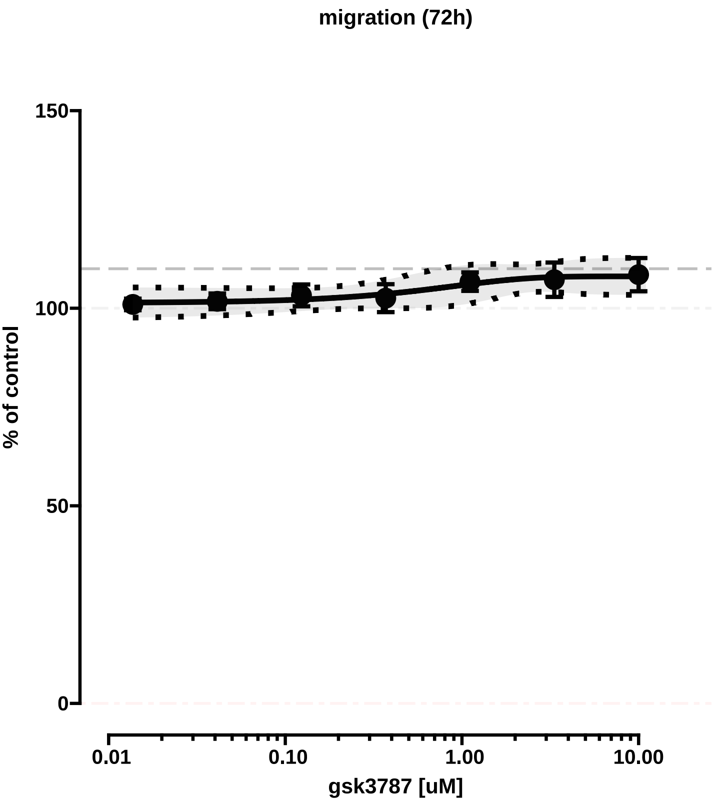

Model: Shifted exponential decay
Model abbr.: EXD.3()
Bechmark-Response (BMR): 10

BMCL: NA
BMC: NA
BMCU: NA

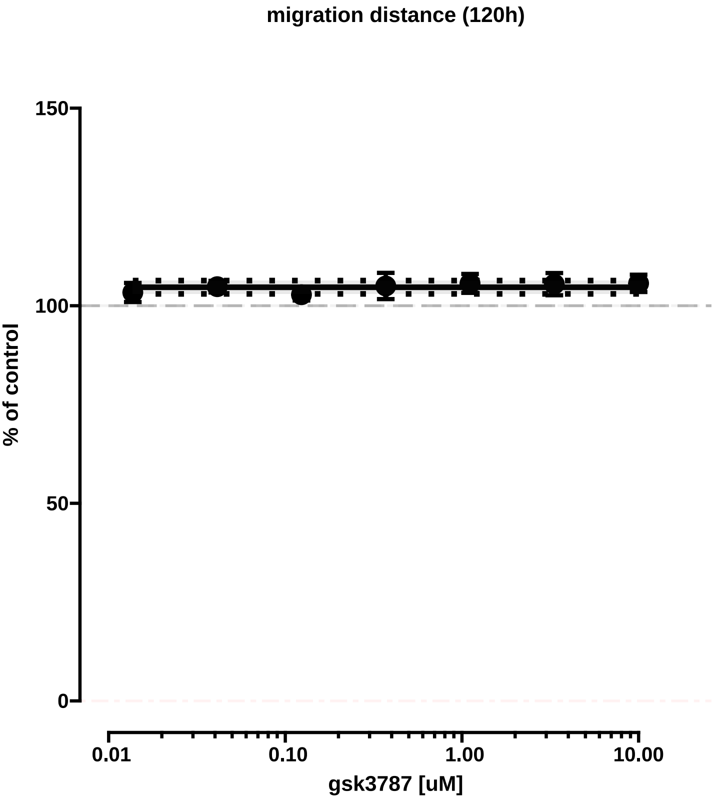

Model: 1-Parameter
Model abbr.: lm.1
Bechmark-Response (BMR): 10

BMCL: NA
BMC: NA
BMCU: NA

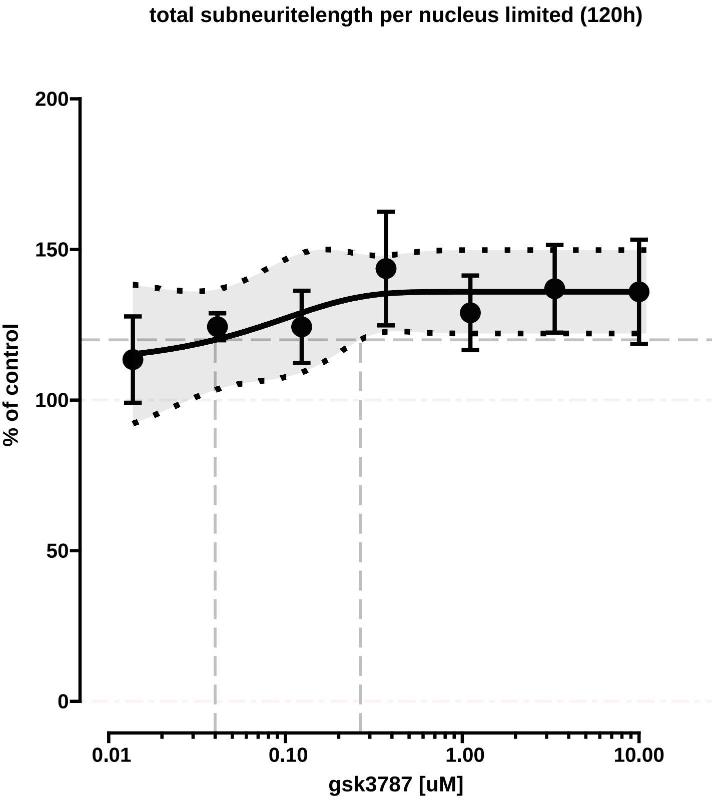

Model: Shifted exponential decay
Model abbr.: EXD.3()
Bechmark-Response (BMR): 20

BMCL: NA
BMC: 0.04
BMCU: 0.265

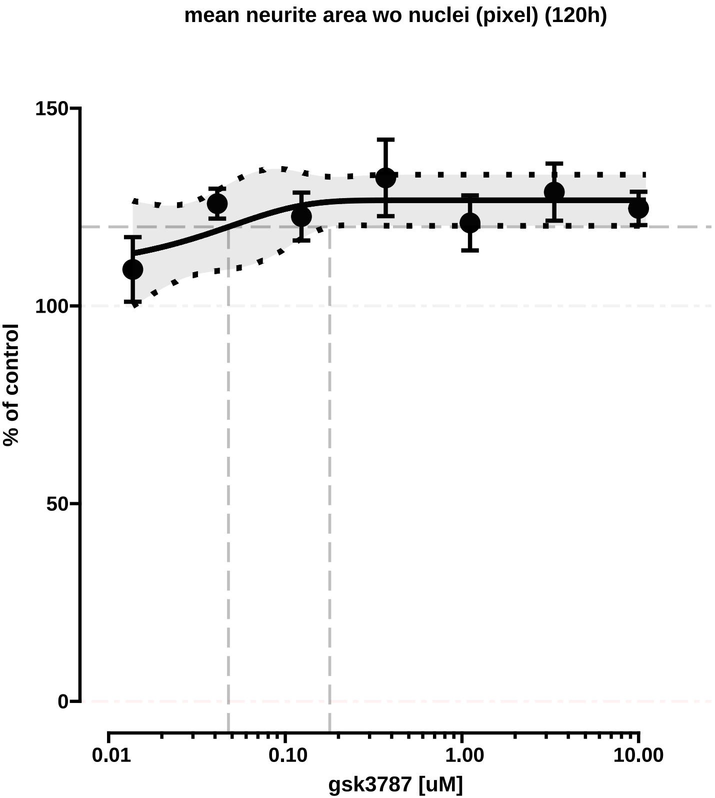

Model: Logistic (ED50 as parameter) with lower limit fixed at 0
Model abbr.: L.3()
Bechmark-Response (BMR): 20

BMCL: NA
BMC: 0.048
BMCU: 0.179

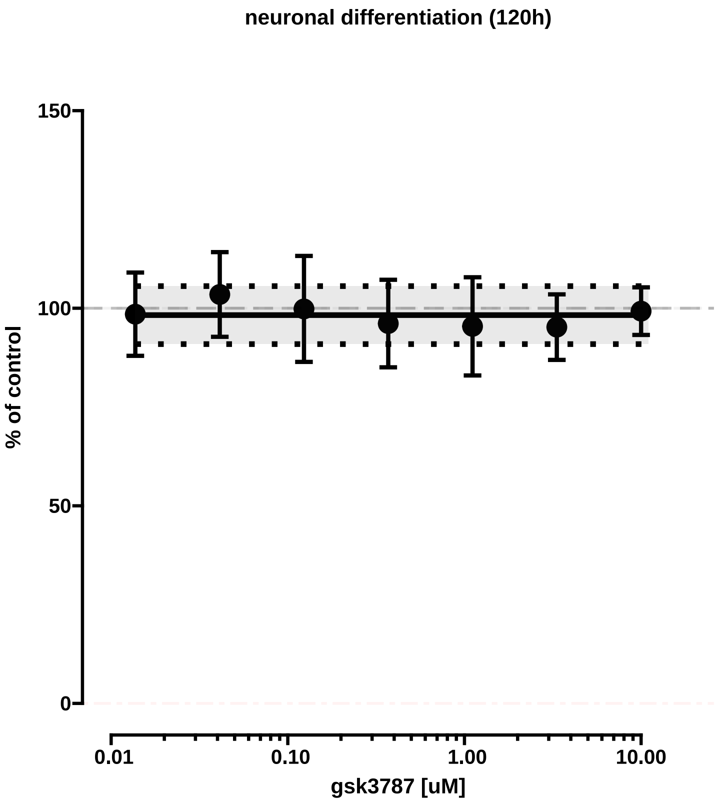

Model: 1-Parameter
Model abbr.: lm.1
Bechmark-Response (BMR): 25

BMCL: NA
BMC: NA
BMCU: NA

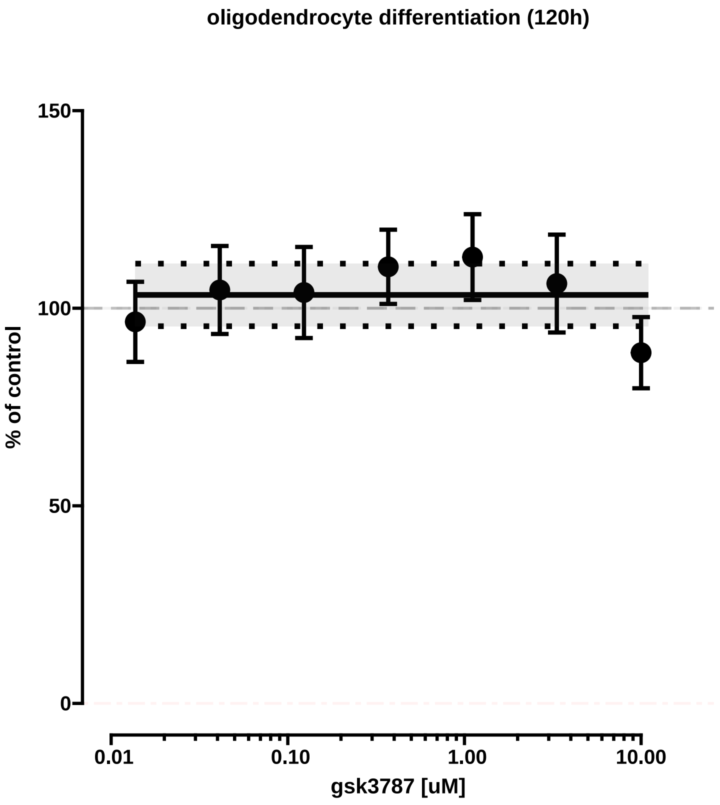

Model: 1-Parameter
Model abbr.: lm.1
Bechmark-Response (BMR): 25

BMCL: NA
BMC: NA
BMCU: NA

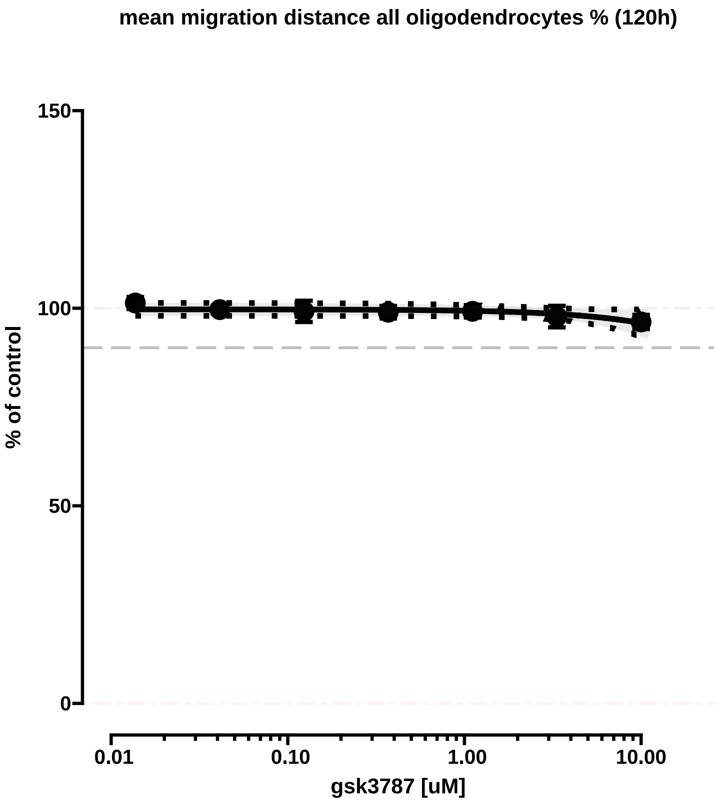

Model: Exponential decay with lower limit at 0
Model abbr.: EXD.2()
Bechmark-Response (BMR): 10

BMCL: NA
BMC: NA
BMCU: NA

mean migration distance all neurons % (120h)

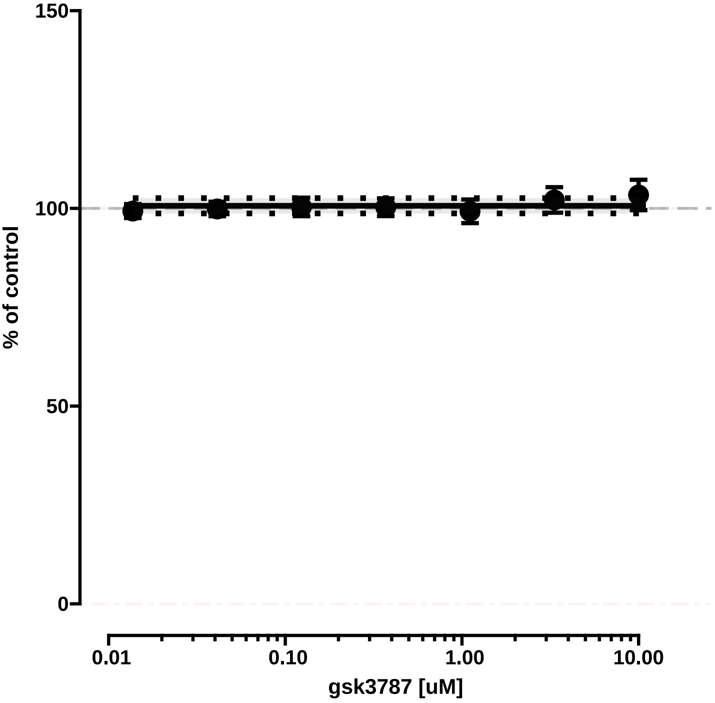

Model: 1-Parameter
Model abbr.: lm.1
Bechmark-Response (BMR): 10

BMCL: NA
BMC: NA
BMCU: NA

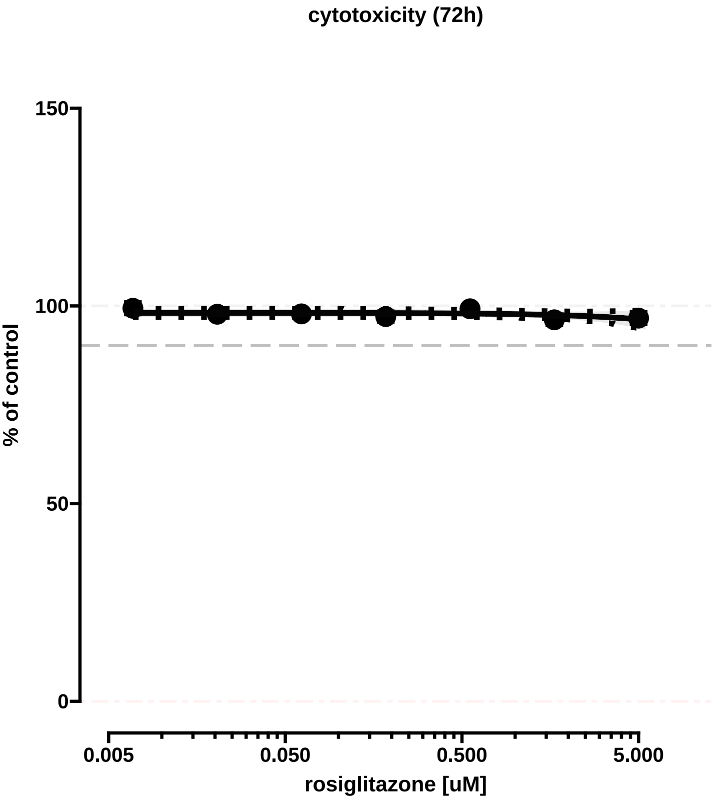

Model: Exponential decay with lower limit at 0
Model abbr.: EXD.2()
Bechmark-Response (BMR): 10

BMCL: NA
BMC: NA
BMCU: NA

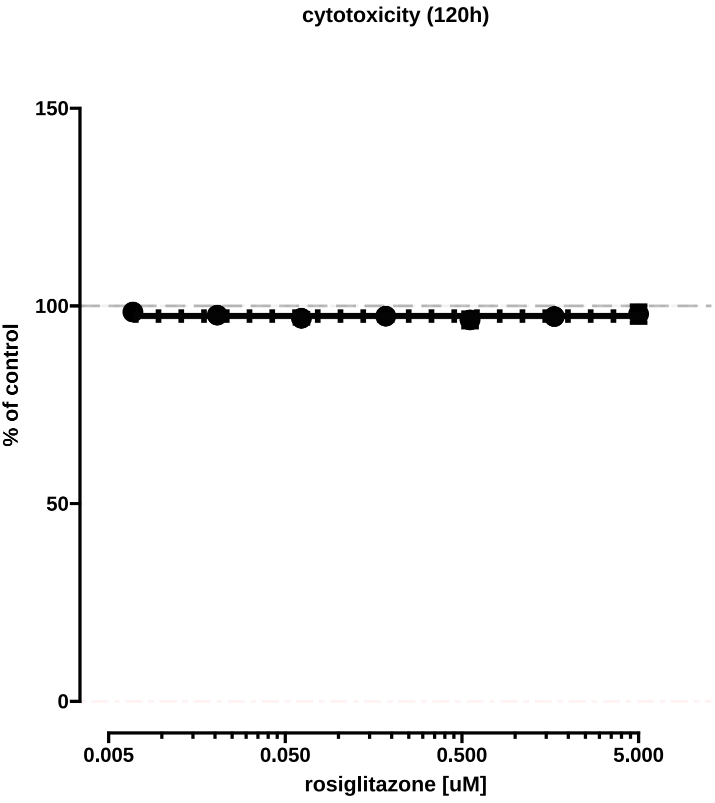

Model: 1-Parameter
Model abbr.: lm.1
Bechmark-Response (BMR): 10

BMCL: NA
BMC: NA
BMCU: NA

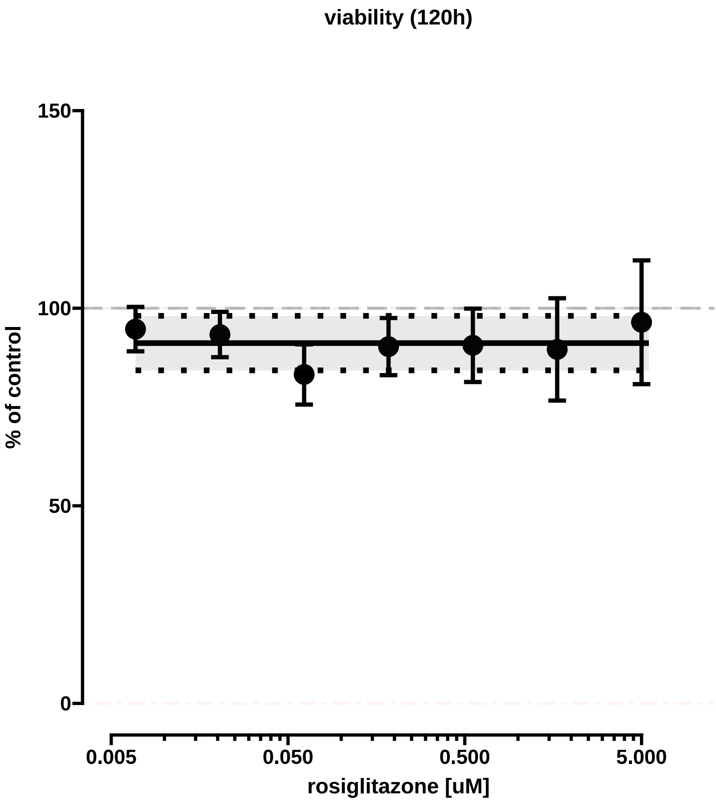

Model: 1-Parameter
Model abbr.: lm.1
Bechmark-Response (BMR): 20

BMCL: NA
BMC: NA
BMCU: NA

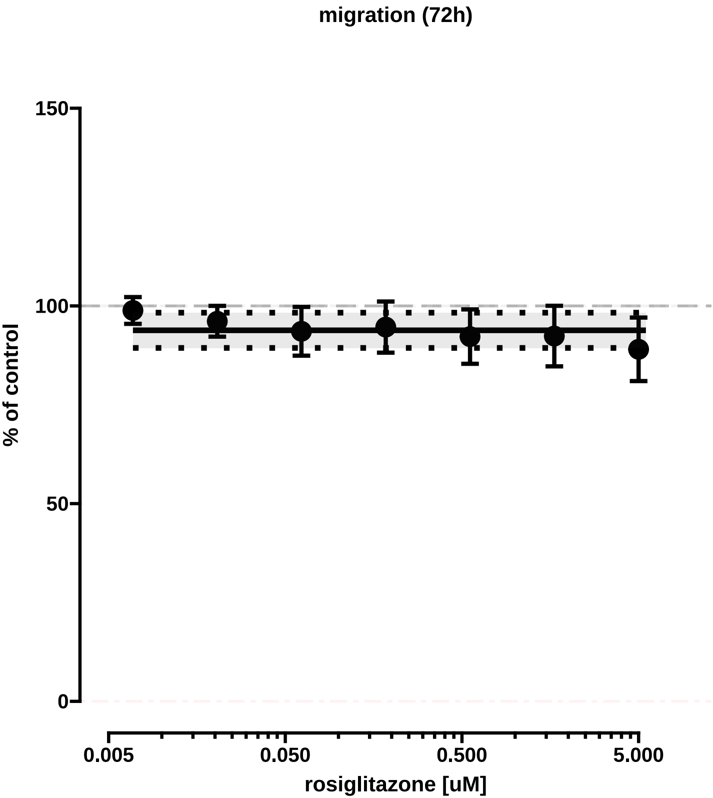

Model: 1-Parameter
Model abbr.: lm.1
Bechmark-Response (BMR): 10

BMCL: NA
BMC: NA
BMCU: NA

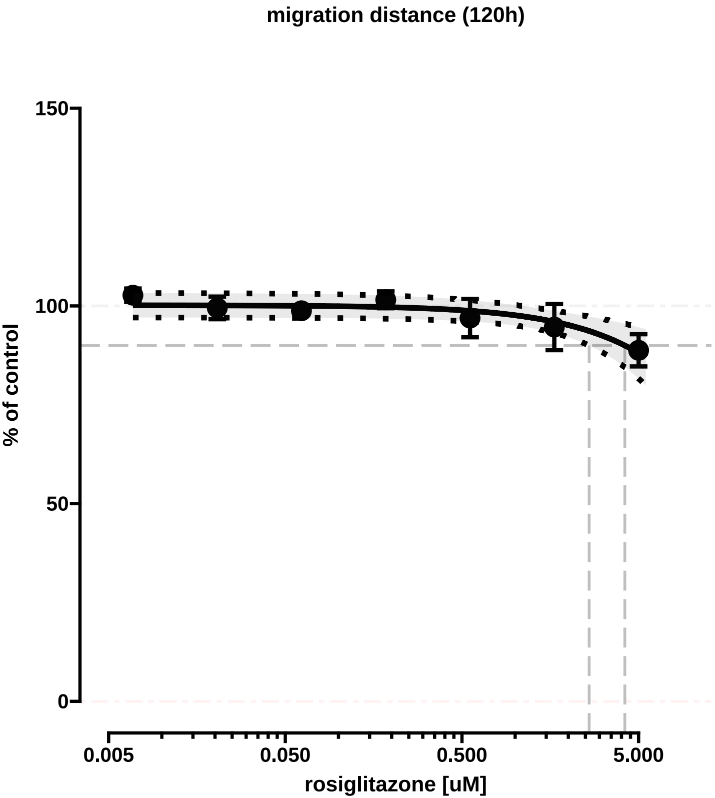

Model: Exponential decay with lower limit at 0
Model abbr.: EXD.2()
Bechmark-Response (BMR): 10

BMCL: 2.625
BMC: 4.178
BMCU: NA

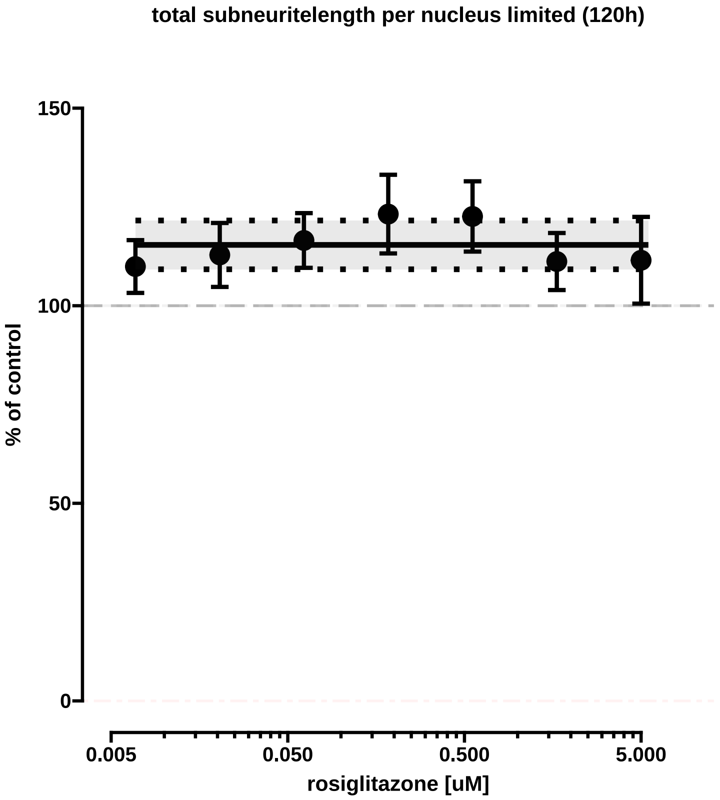

Model: 1-Parameter
Model abbr.: lm.1
Bechmark-Response (BMR): 20

BMCL: NA
BMC: NA
BMCU: NA

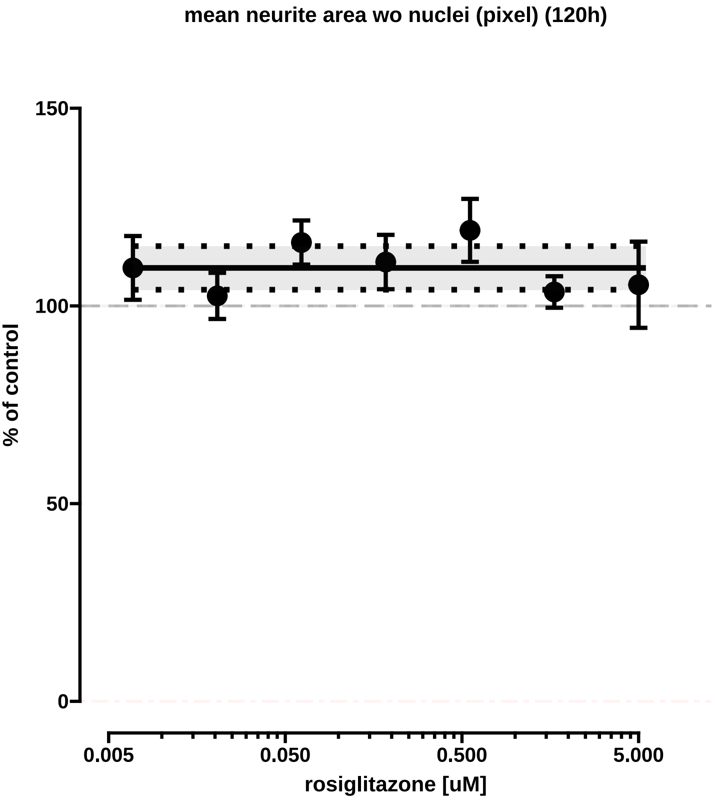

Model: 1-Parameter
Model abbr.: lm.1
Bechmark-Response (BMR): 20

BMCL: NA
BMC: NA
BMCU: NA

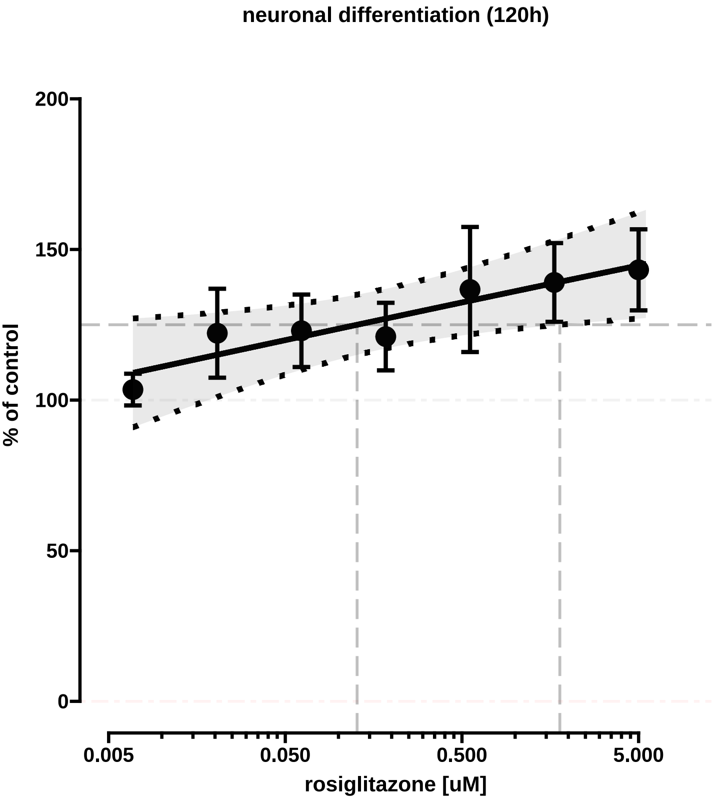

Model: Weibull (type 1) with lower limit at 0
Model abbr.: W1.3()
Bechmark-Response (BMR): 25

BMCL: NA
BMC: 0.128
BMCU: 1.791

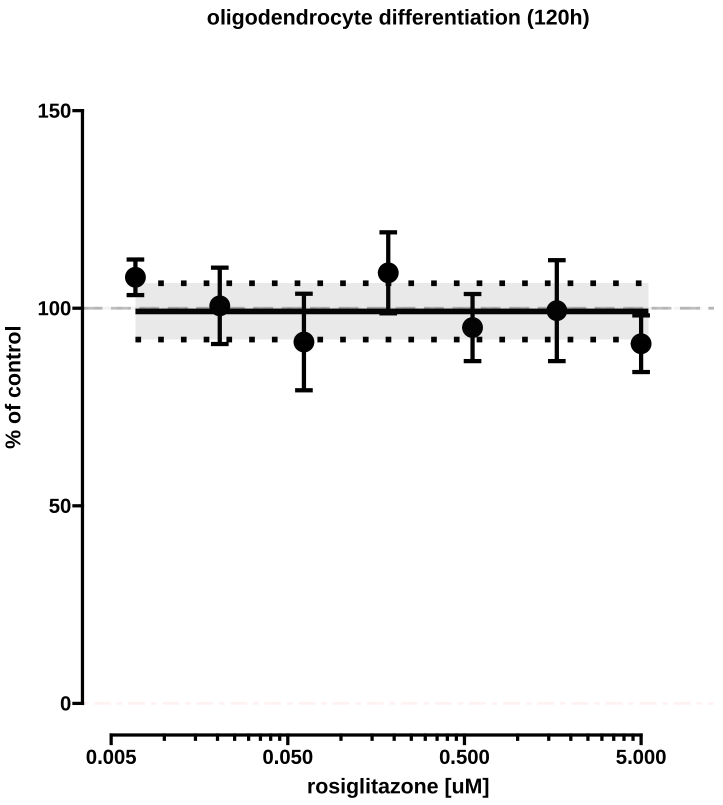

Model: 1-Parameter
Model abbr.: lm.1
Bechmark-Response (BMR): 25

BMCL: NA
BMC: NA
BMCU: NA

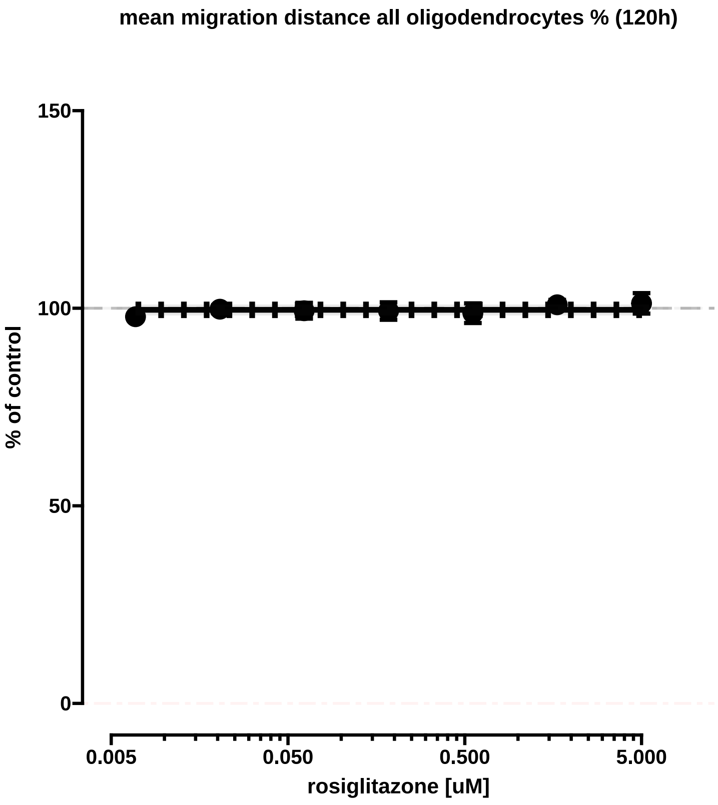

Model: 1-Parameter
Model abbr.: lm.1
Bechmark-Response (BMR): 10

BMCL: NA
BMC: NA
BMCU: NA

mean migration distance all neurons % (120h)

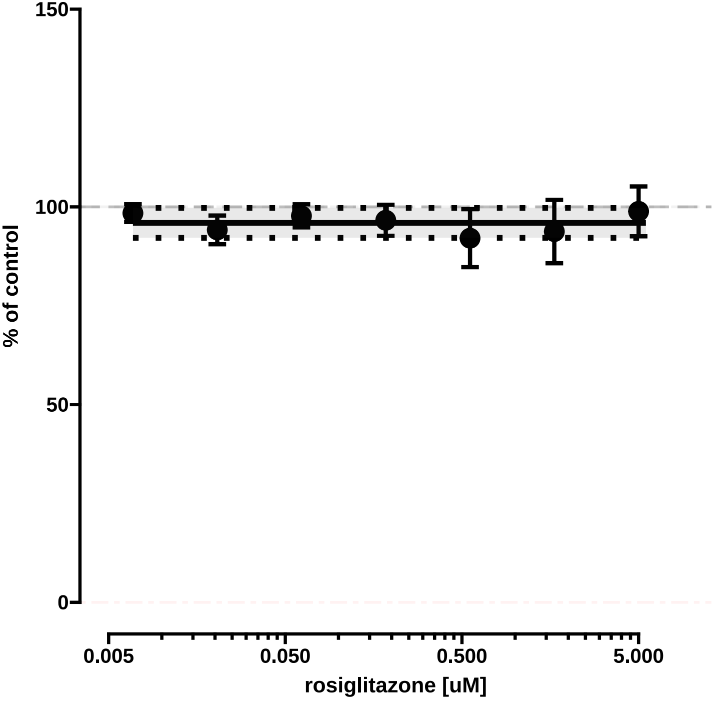

Model: 1-Parameter
Model abbr.: lm.1
Bechmark-Response (BMR): 10

BMCL: NA
BMC: NA
BMCU: NA

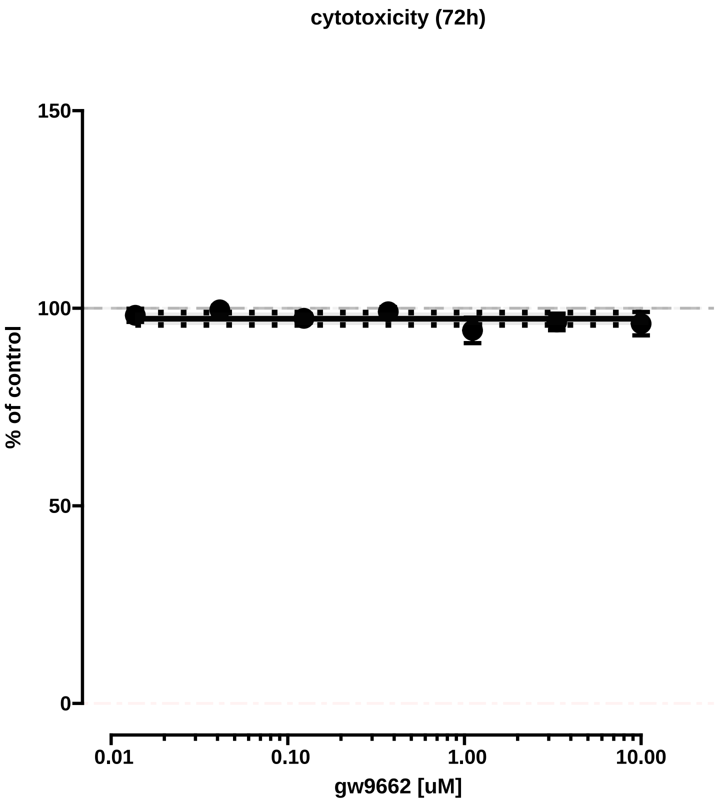

Model: 1-Parameter
Model abbr.: lm.1
Bechmark-Response (BMR): 10

BMCL: NA
BMC: NA
BMCU: NA

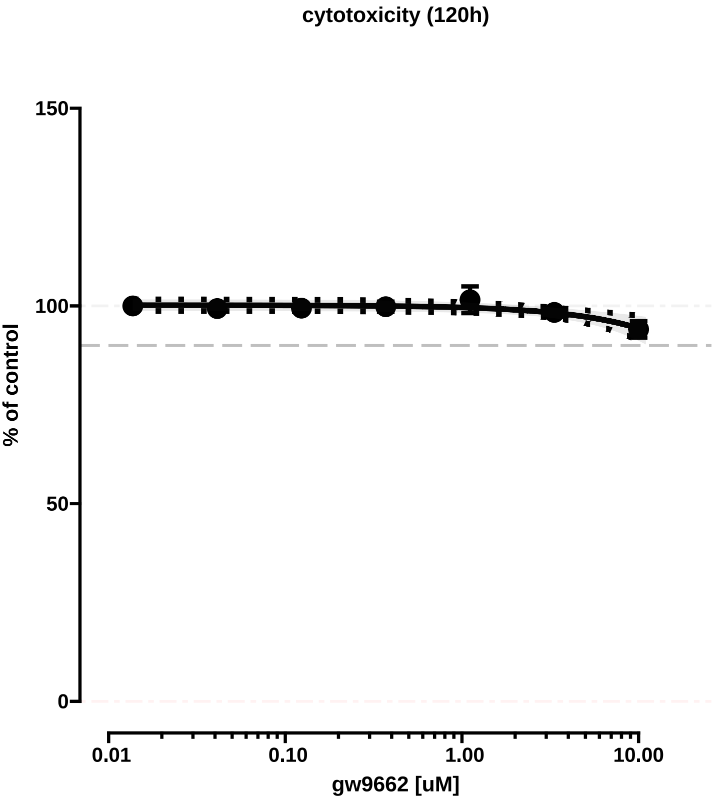

Model: Linear
Model abbr.: lm
Bechmark-Response (BMR): 10

BMCL: NA
BMC: NA
BMCU: NA

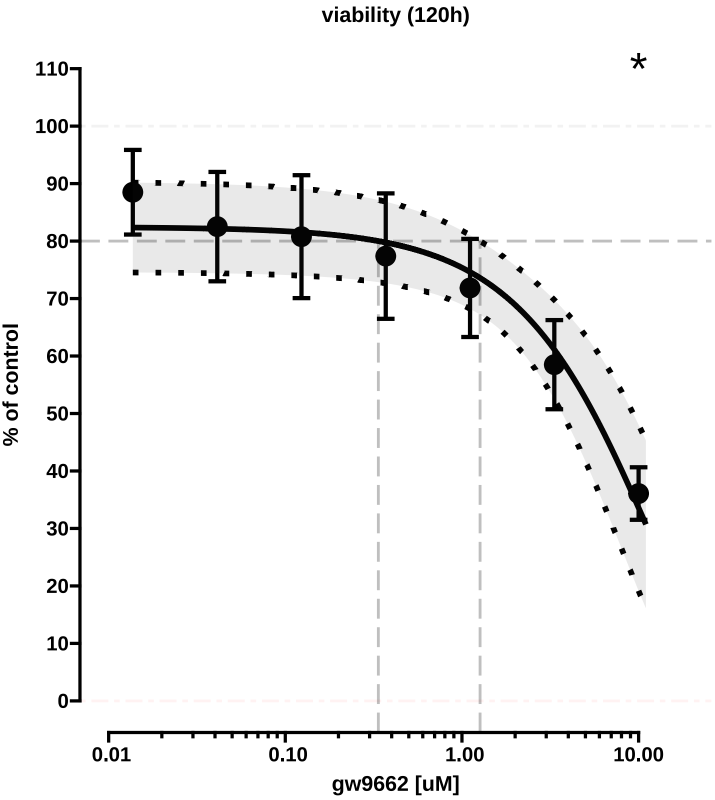

Model: Exponential decay with lower limit at 0
Model abbr.: EXD.2()
Bechmark-Response (BMR): 20

BMCL: NA
BMC: 0.336
BMCU: 1.266

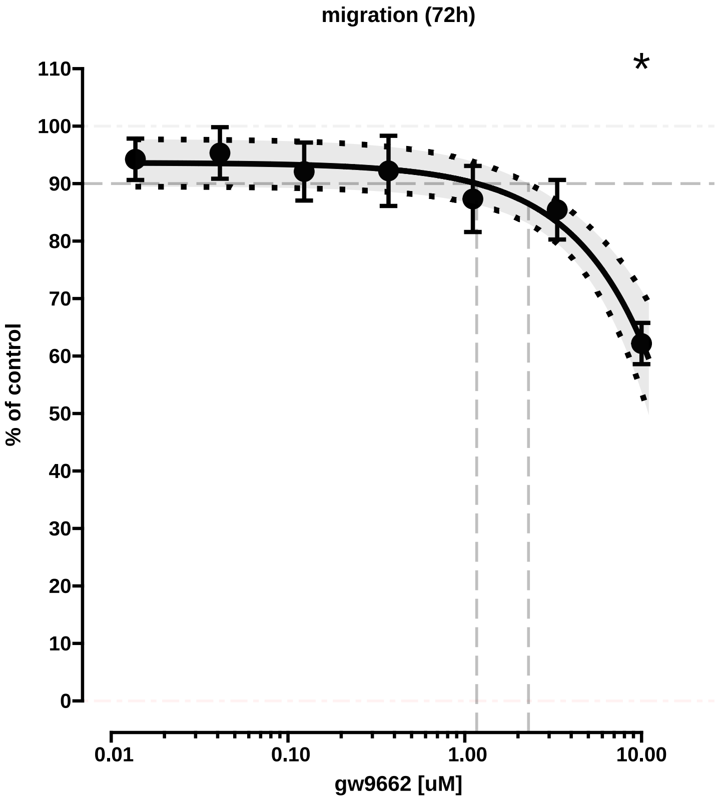

Model: Linear
Model abbr.: lm
Bechmark-Response (BMR): 10

BMCL: NA
BMC: 1.168
BMCU: 2.293

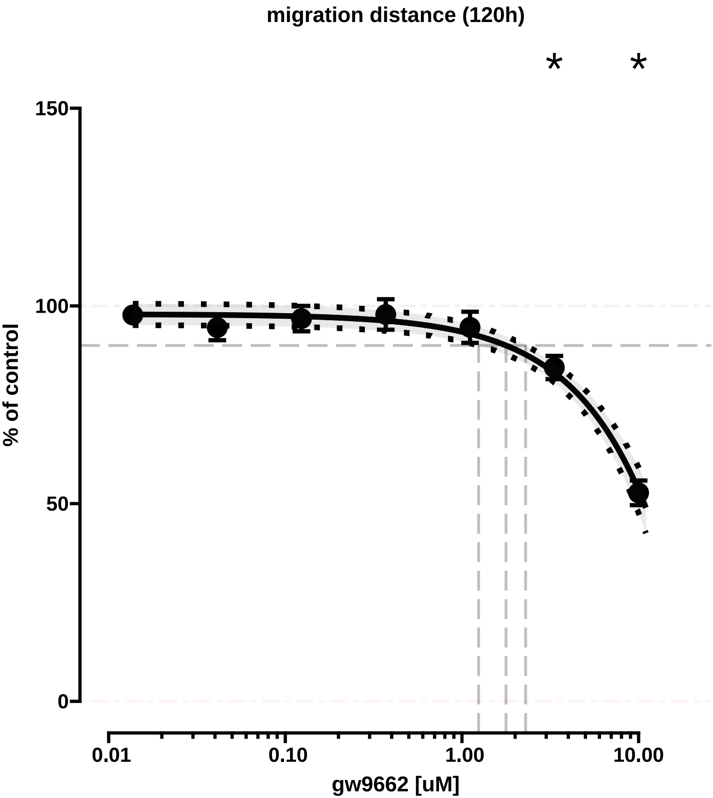

Model: Linear
Model abbr.: lm
Bechmark-Response (BMR): 10

BMCL: 1.243
BMC: 1.776
BMCU: 2.293

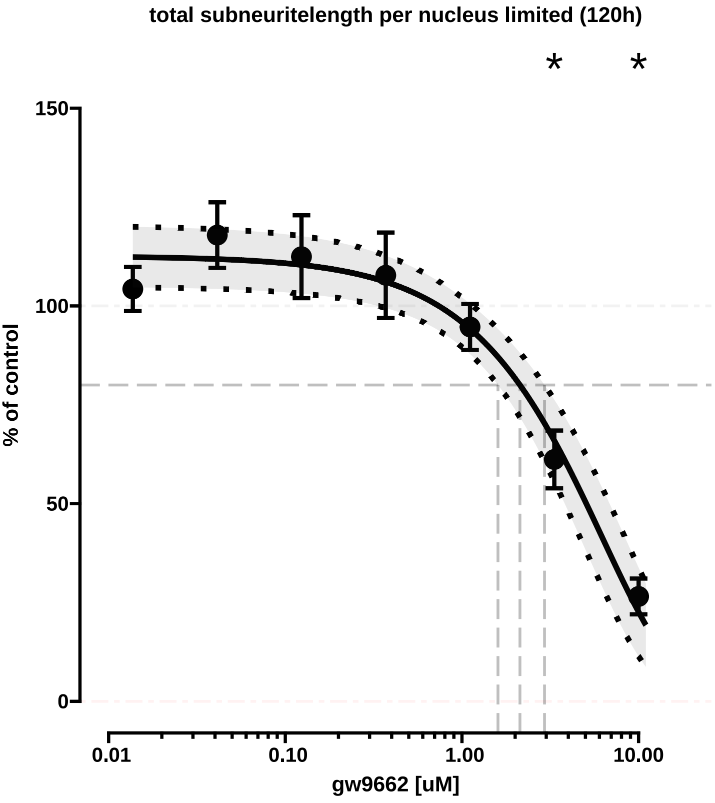

Model: Exponential decay with lower limit at 0
Model abbr.: EXD.2()
Bechmark-Response (BMR): 20

BMCL: 1.599
BMC: 2.129
BMCU: 2.933

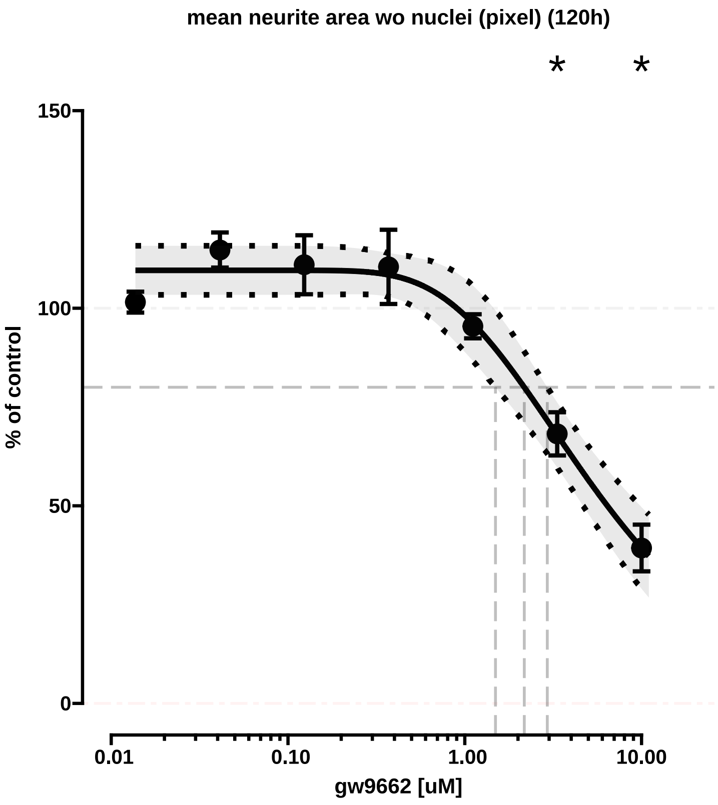

Model: Weibull (type 2) with lower limit at 0
Model abbr.: W2.3()
Bechmark-Response (BMR): 20

BMCL: 1.492
BMC: 2.171
BMCU: 2.929

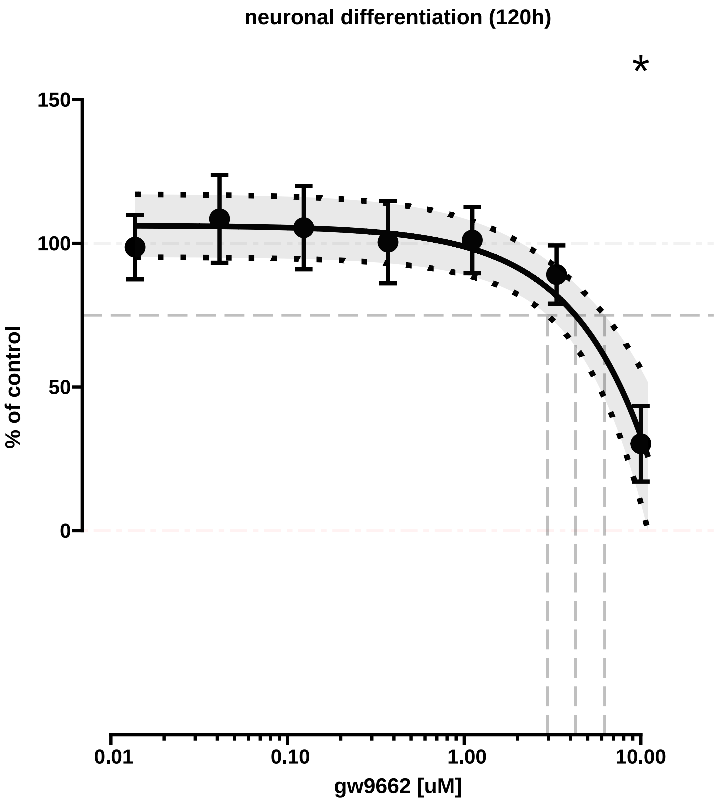

Model: Linear
Model abbr.: lm
Bechmark-Response (BMR): 25

BMCL: 2.961
BMC: 4.261
BMCU: 6.242

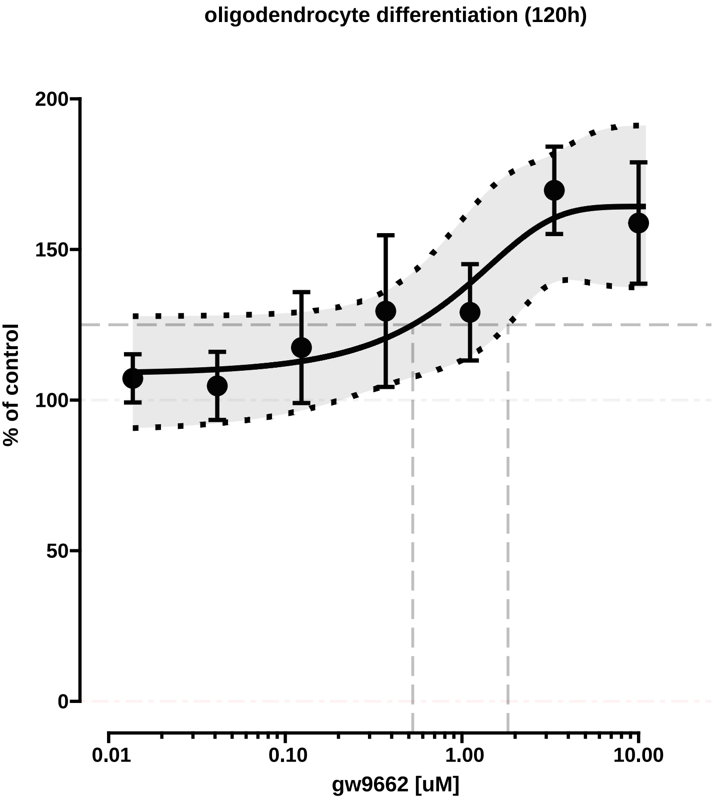

Model: Logistic (ED50 as parameter) with lower limit fixed at 0
Model abbr.: L.3()
Bechmark-Response (BMR): 25

BMCL: NA
BMC: 0.526
BMCU: 1.822

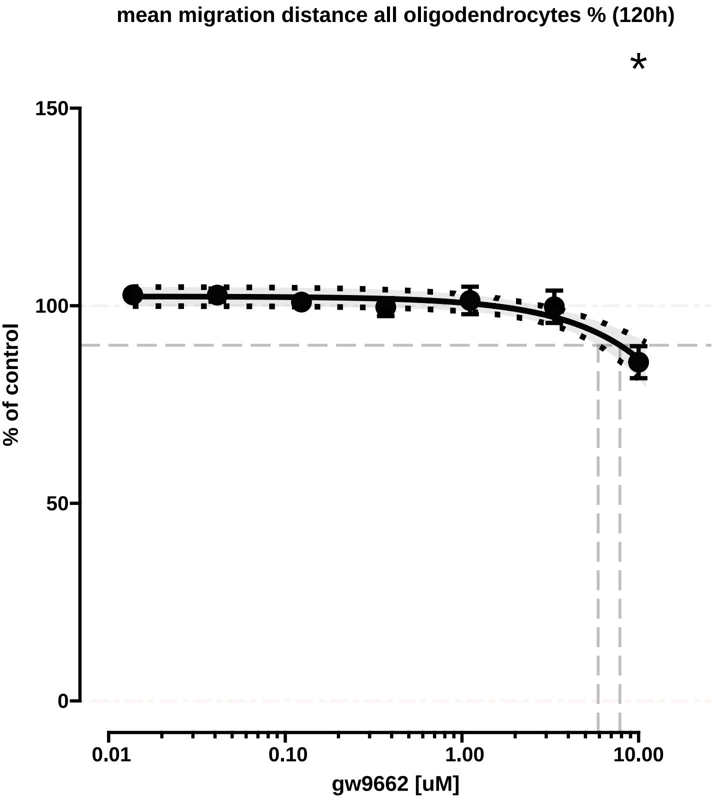

Model: Linear
Model abbr.: lm
Bechmark-Response (BMR): 10

BMCL: 5.901
BMC: 7.836
BMCU: NA

mean migration distance all neurons % (120h)

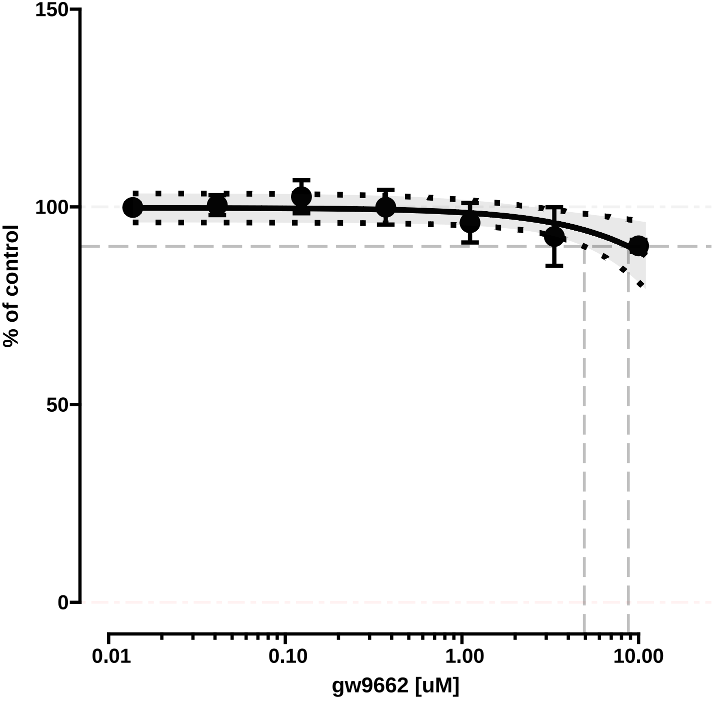

Model: Exponential decay with lower limit at 0
Model abbr.: EXD.2()
Bechmark-Response (BMR): 10

BMCL: 4.929
BMC: 8.751
BMCU: NA

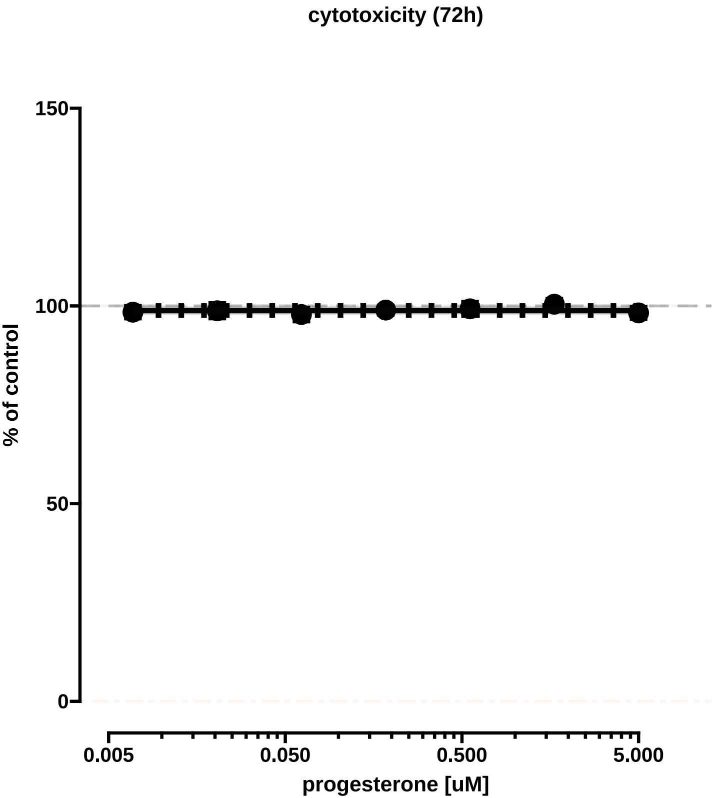

Model: 1-Parameter
Model abbr.: lm.1
Bechmark-Response (BMR): 10

BMCL: NA
BMC: NA
BMCU: NA

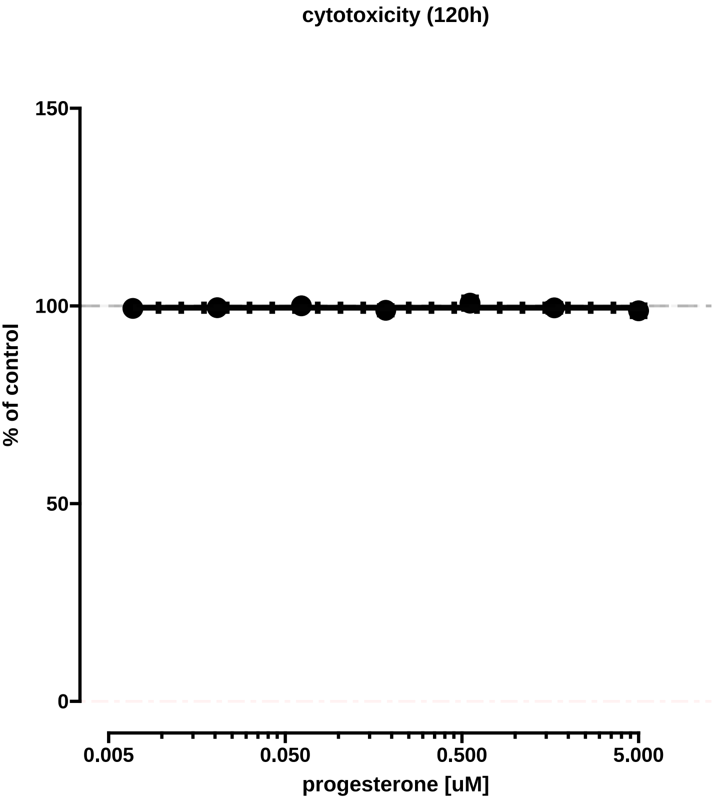

Model: 1-Parameter
Model abbr.: lm.1
Bechmark-Response (BMR): 10

BMCL: NA
BMC: NA
BMCU: NA

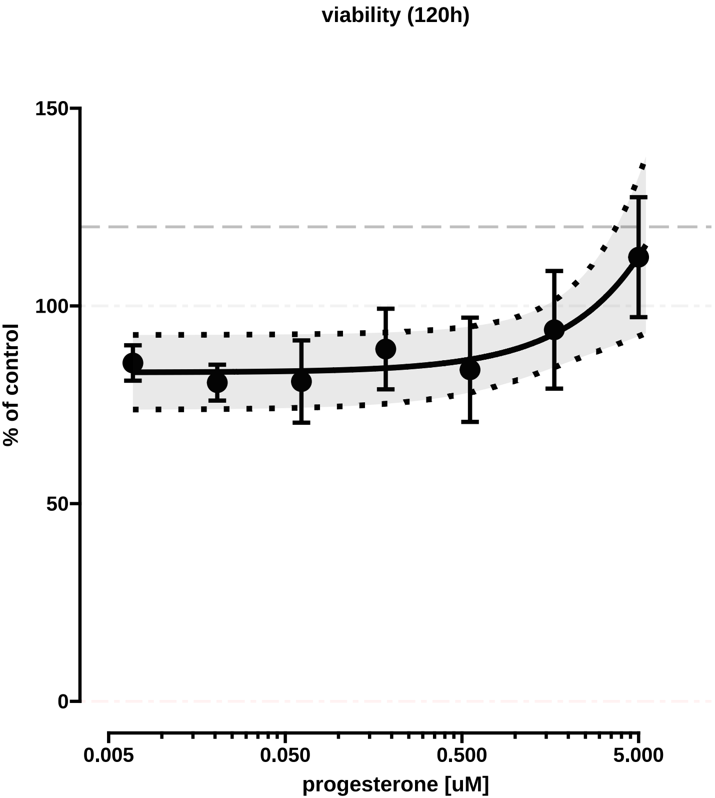

Model: Linear
Model abbr.: lm
Bechmark-Response (BMR): 20

BMCL: NA
BMC: NA
BMCU: NA

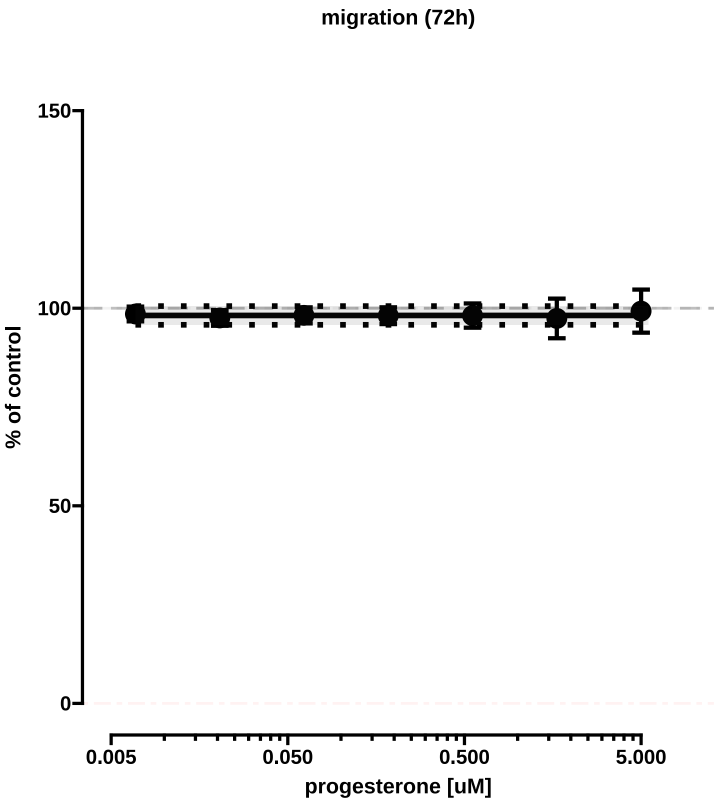

Model: 1-Parameter
Model abbr.: lm.1
Bechmark-Response (BMR): 10

BMCL: NA
BMC: NA
BMCU: NA

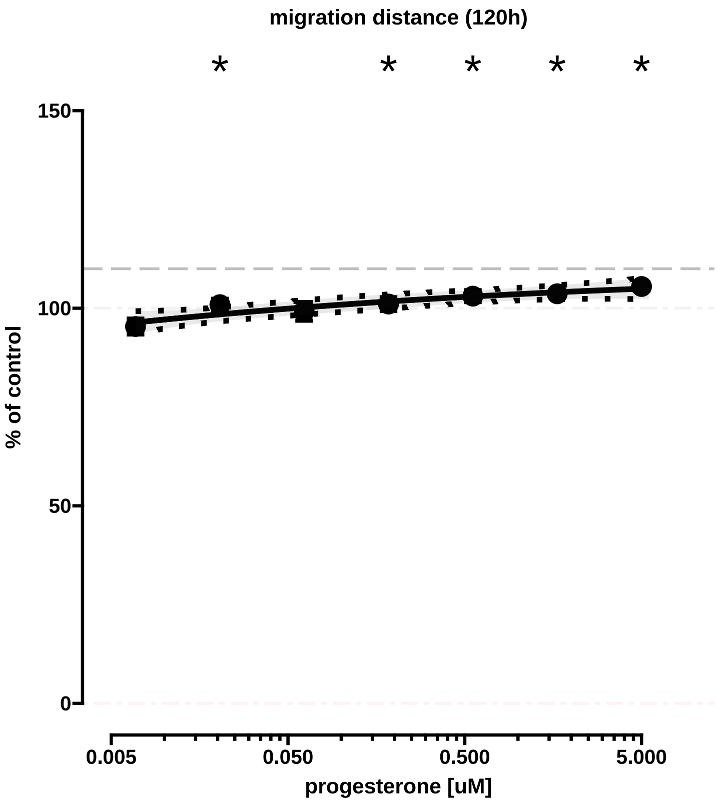

Model: Log-logistic (log(ED50) as parameter) with lower limit at 0
Model abbr.: LL2.3()
Bechmark-Response (BMR): 10

BMCL: NA
BMC: NA
BMCU: NA

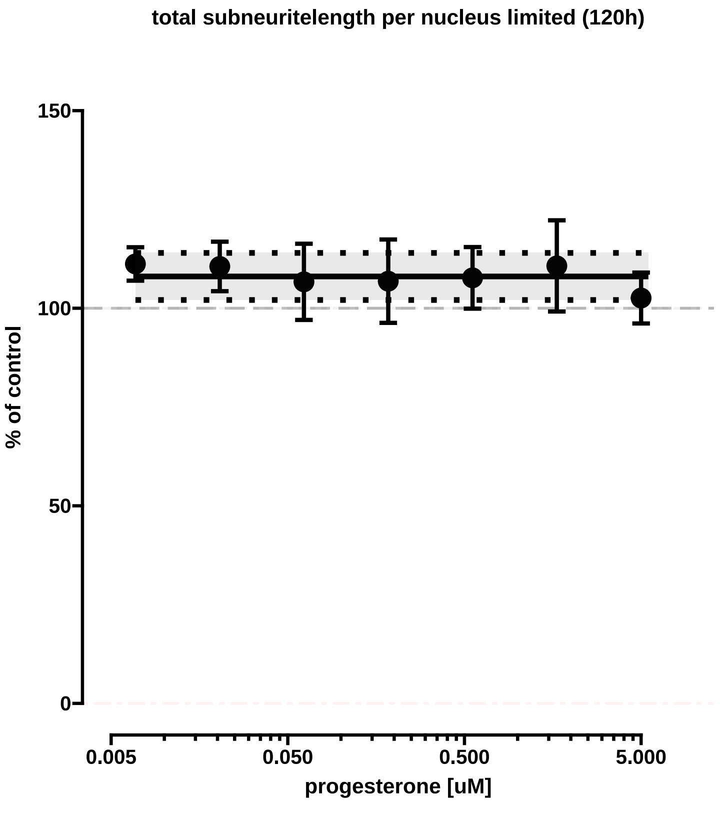

Model: 1-Parameter
Model abbr.: lm.1
Bechmark-Response (BMR): 20

BMCL: NA
BMC: NA
BMCU: NA

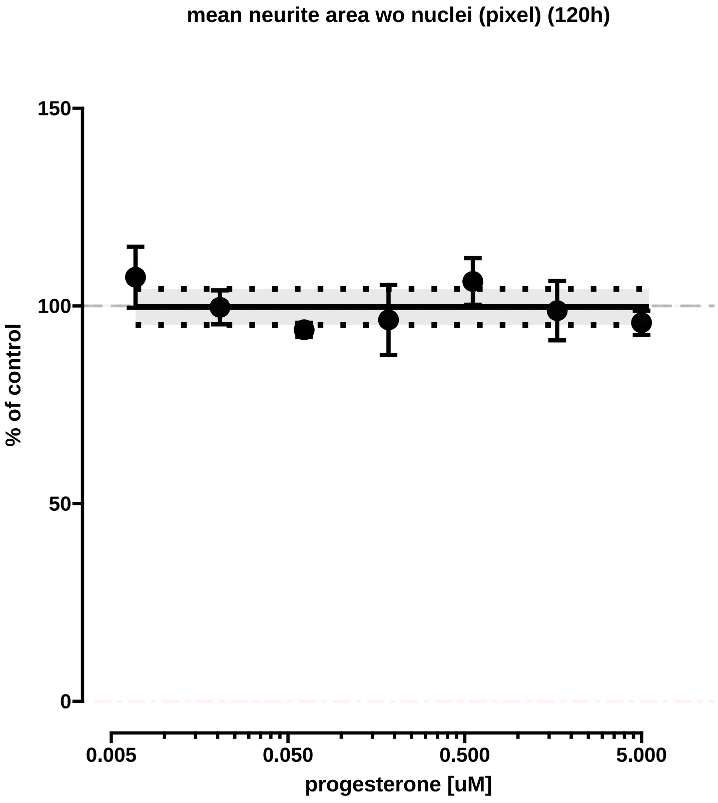

Model: 1-Parameter
Model abbr.: lm.1
Bechmark-Response (BMR): 20

BMCL: NA
BMC: NA
BMCU: NA

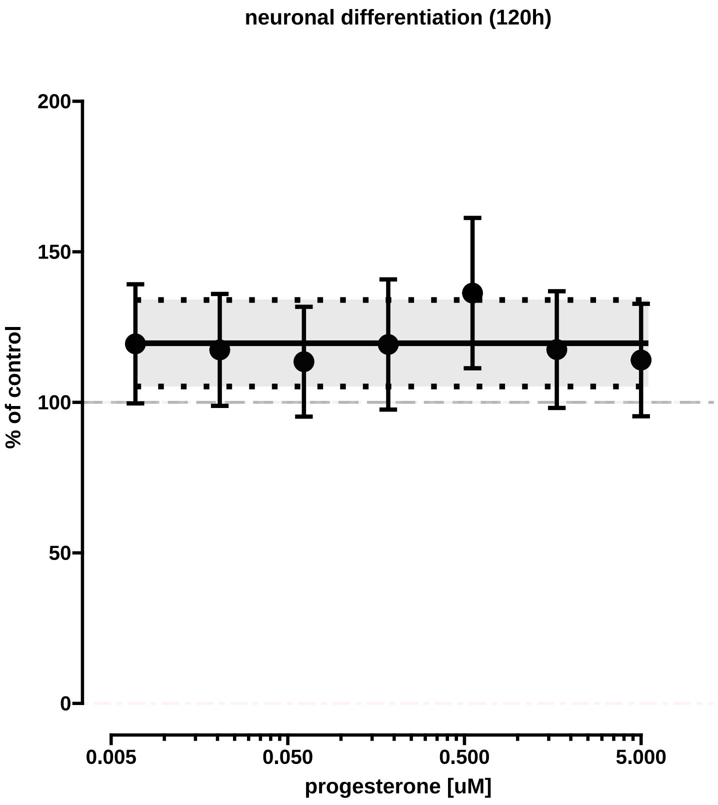

Model: 1-Parameter
Model abbr.: lm.1
Bechmark-Response (BMR): 25

BMCL: NA
BMC: NA
BMCU: NA

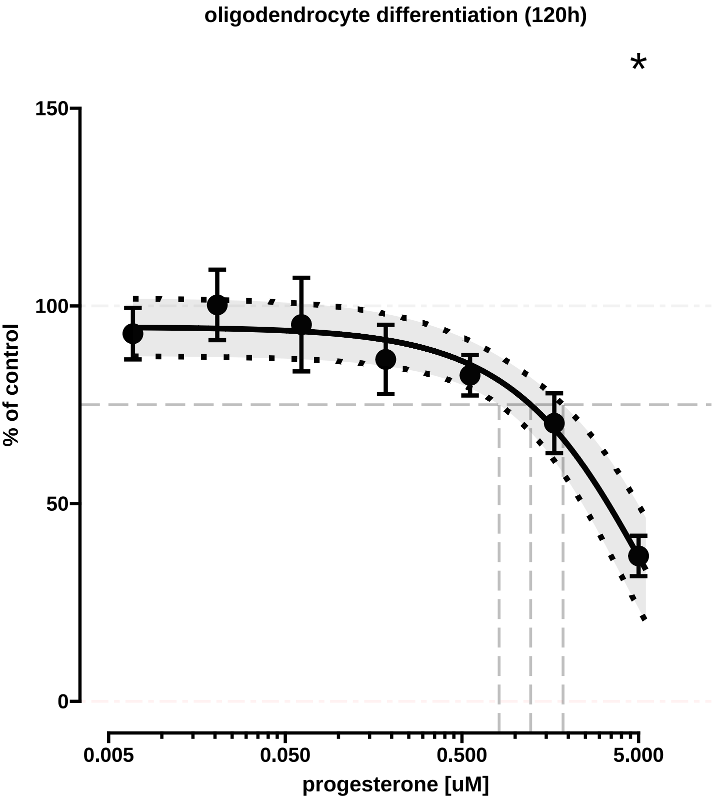

Model: Exponential decay with lower limit at 0
Model abbr.: EXD.2()
Bechmark-Response (BMR): 25

BMCL: 0.812
BMC: 1.225
BMCU: 1.868

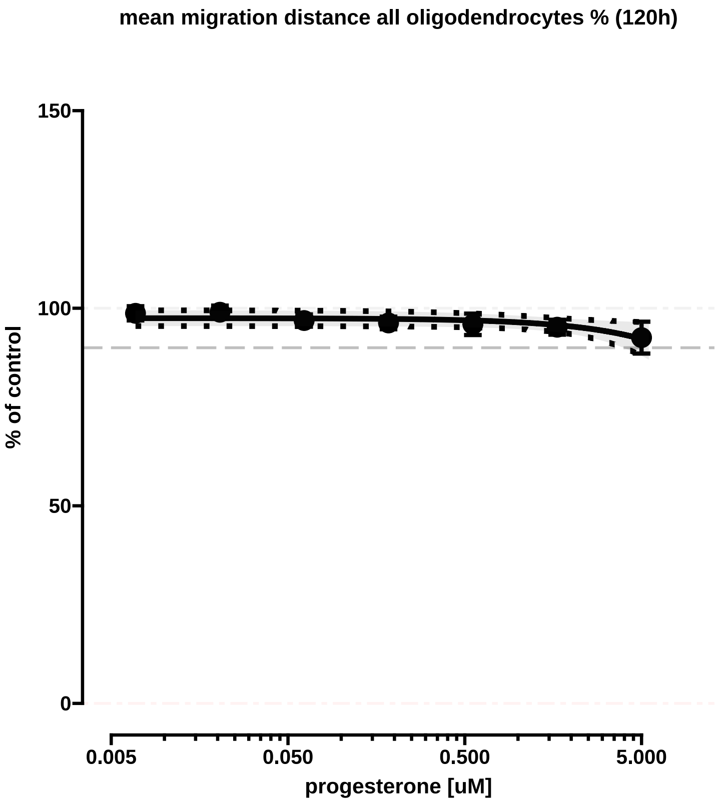

Model: Linear
Model abbr.: lm
Bechmark-Response (BMR): 10

BMCL: NA
BMC: NA
BMCU: NA

mean migration distance all neurons % (120h)

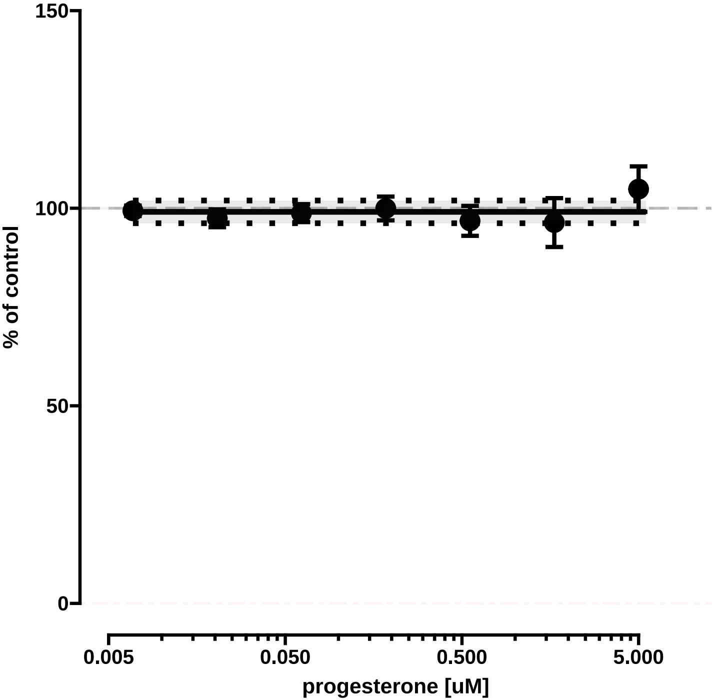

Model: 1-Parameter
Model abbr.: lm.1
Bechmark-Response (BMR): 10

BMCL: NA
BMC: NA
BMCU: NA

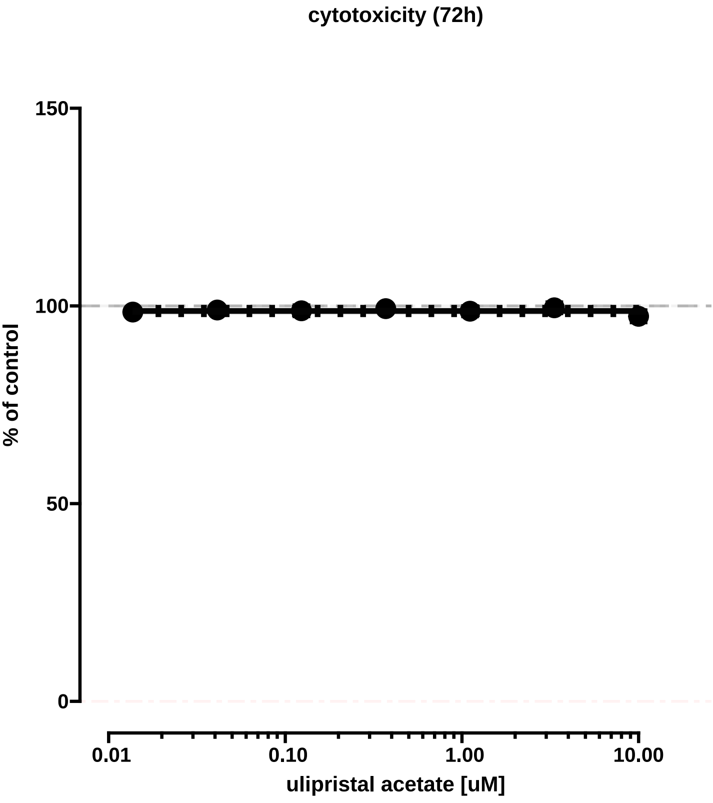

Model: 1-Parameter
Model abbr.: lm.1
Bechmark-Response (BMR): 10

BMCL: NA
BMC: NA
BMCU: NA

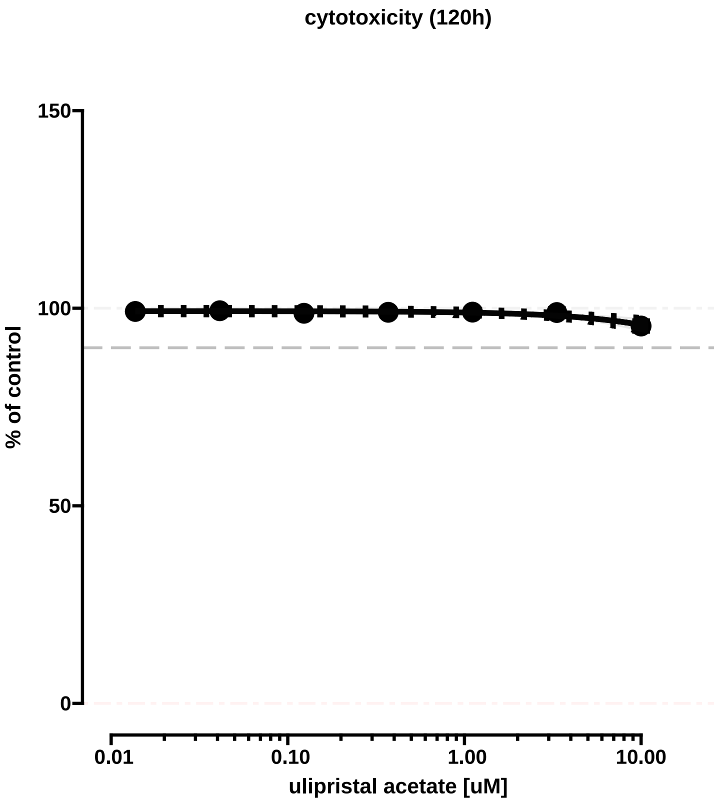

Model: Linear
Model abbr.: lm
Bechmark-Response (BMR): 10

BMCL: NA
BMC: NA
BMCU: NA

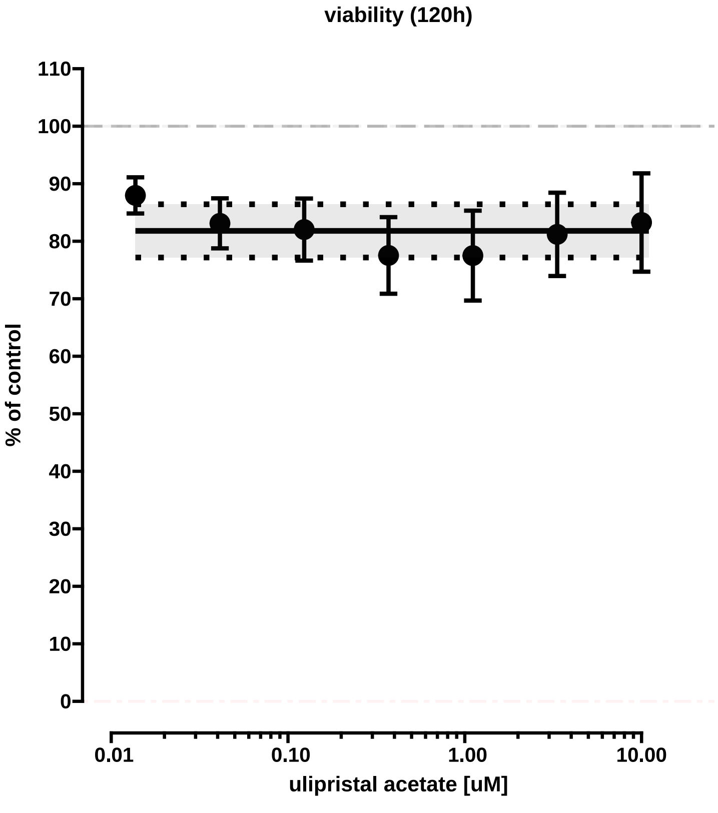

Model: 1-Parameter
Model abbr.: lm.1
Bechmark-Response (BMR): 20

BMCL: NA
BMC: NA
BMCU: NA

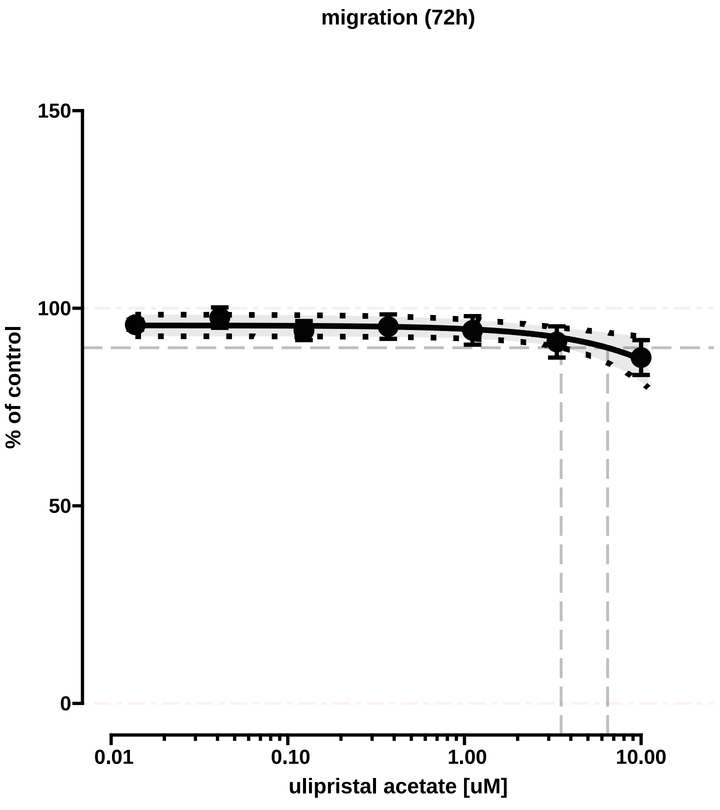

Model: Exponential decay with lower limit at 0
Model abbr.: EXD.2()
Bechmark-Response (BMR): 10

BMCL: 3.526
BMC: 6.463
BMCU: NA

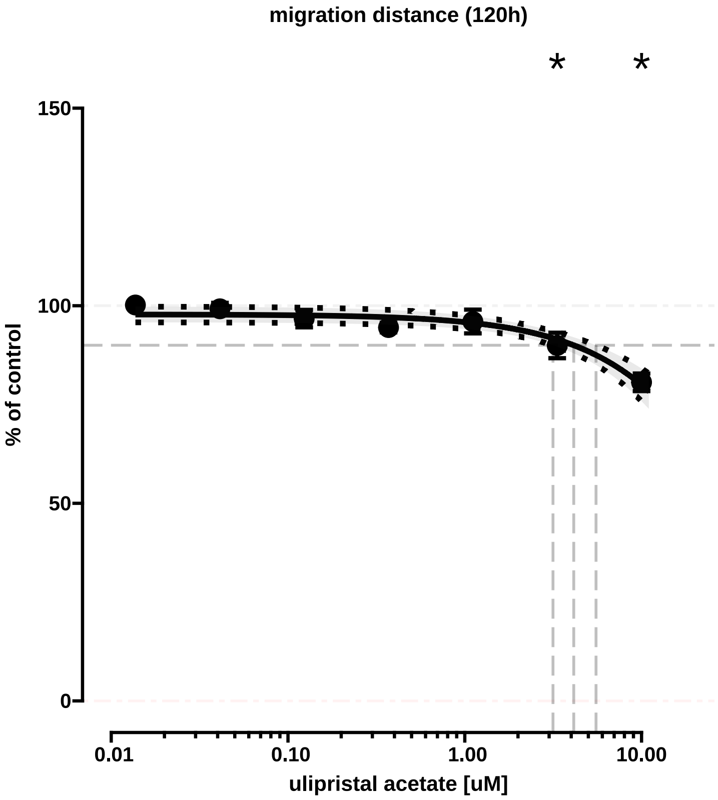

Model: Exponential decay with lower limit at 0
Model abbr.: EXD.2()
Bechmark-Response (BMR): 10

BMCL: 3.155
BMC: 4.134
BMCU: 5.527

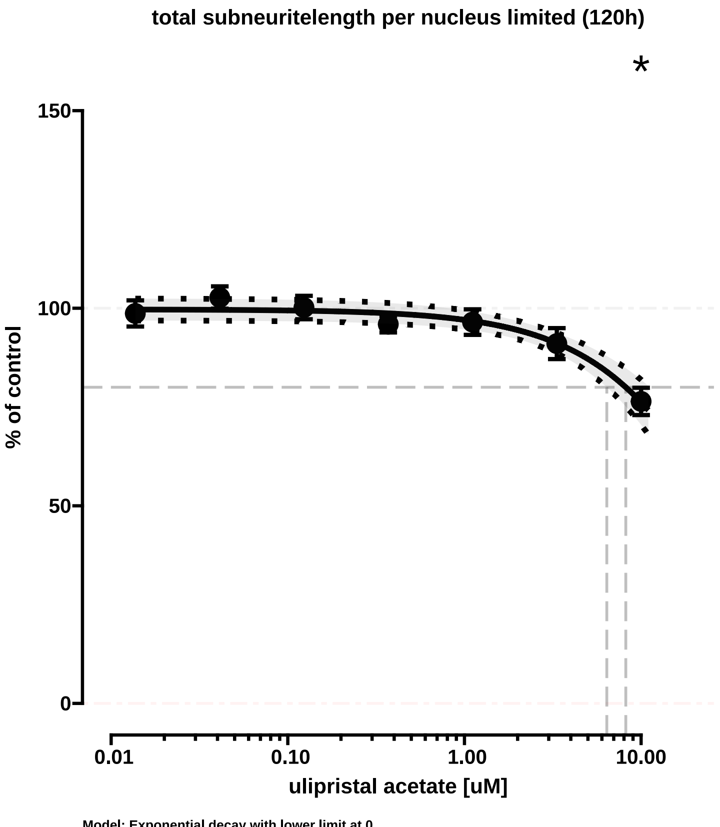

Model: Exponential decay with lower limit at 0
Model abbr.: EXD.2()
Bechmark-Response (BMR): 20

BMCL: 6.394
BMC: 8.19
BMCU: NA

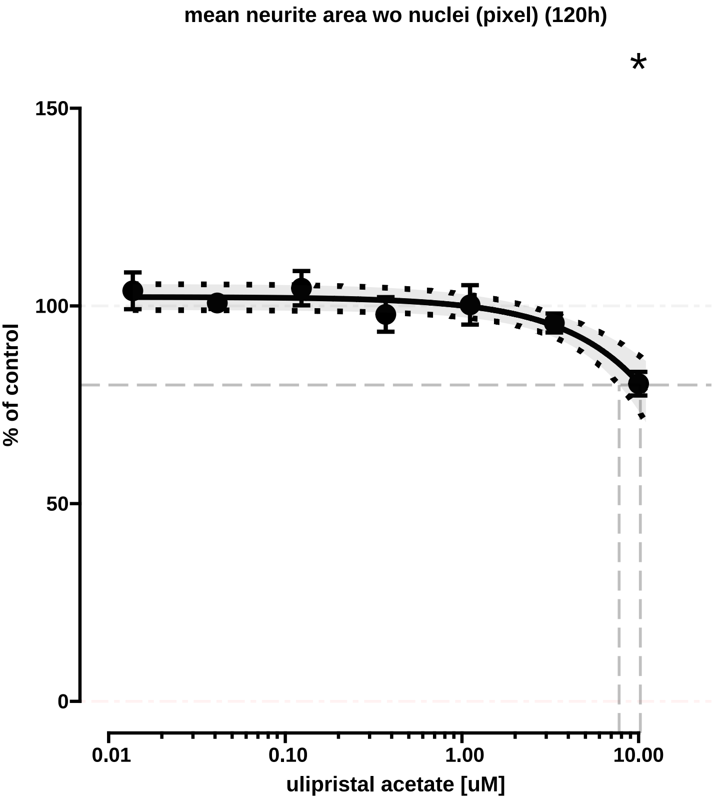

Model: Linear
Model abbr.: lm
Bechmark-Response (BMR): 20

BMCL: 7.758
BMC: 10.233
BMCU: NA

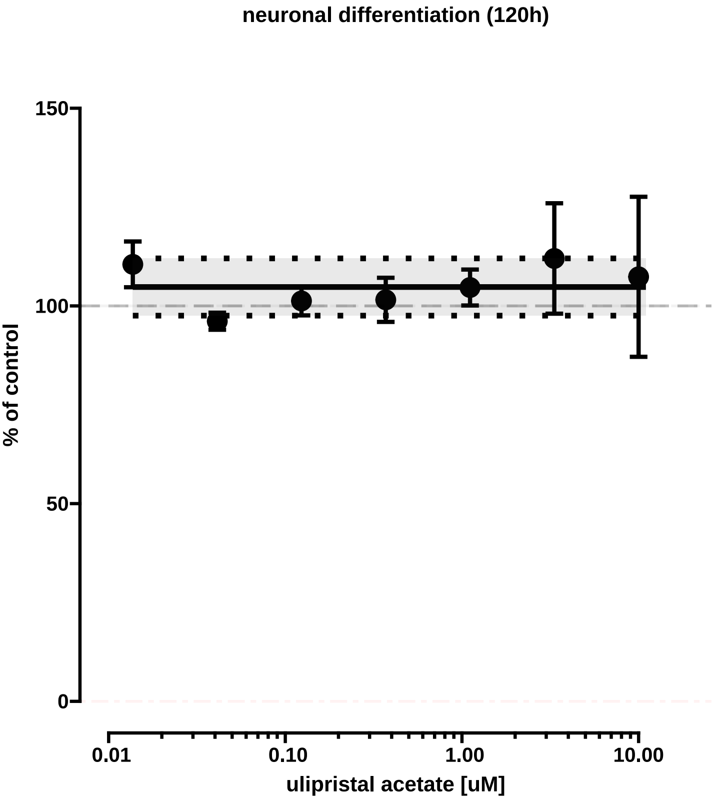

Model: 1-Parameter
Model abbr.: lm.1
Bechmark-Response (BMR): 25

BMCL: NA
BMC: NA
BMCU: NA

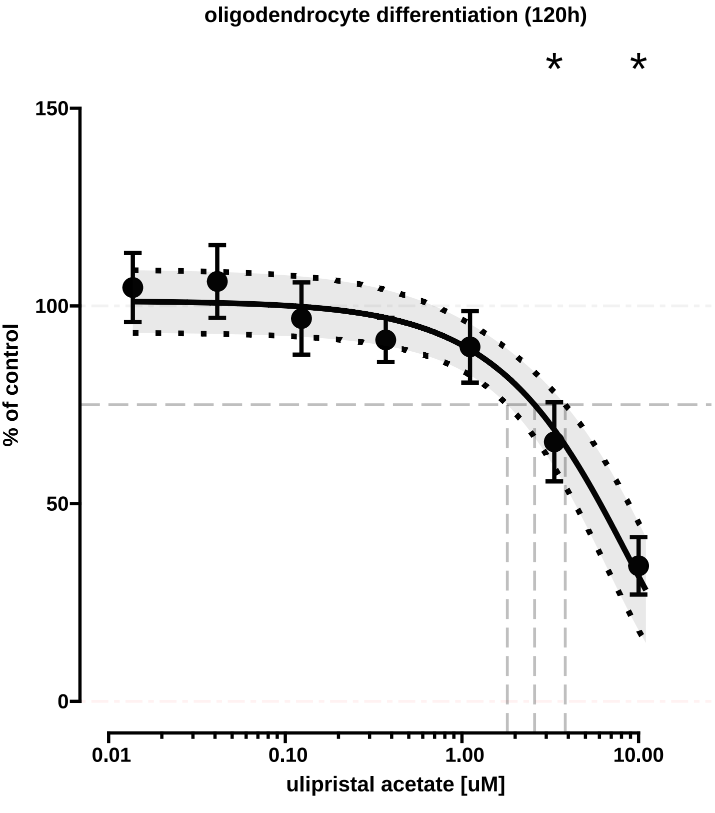

Model: Exponential decay with lower limit at 0
Model abbr.: EXD.2()
Bechmark-Response (BMR): 25

BMCL: 1.806
BMC: 2.578
BMCU: 3.844

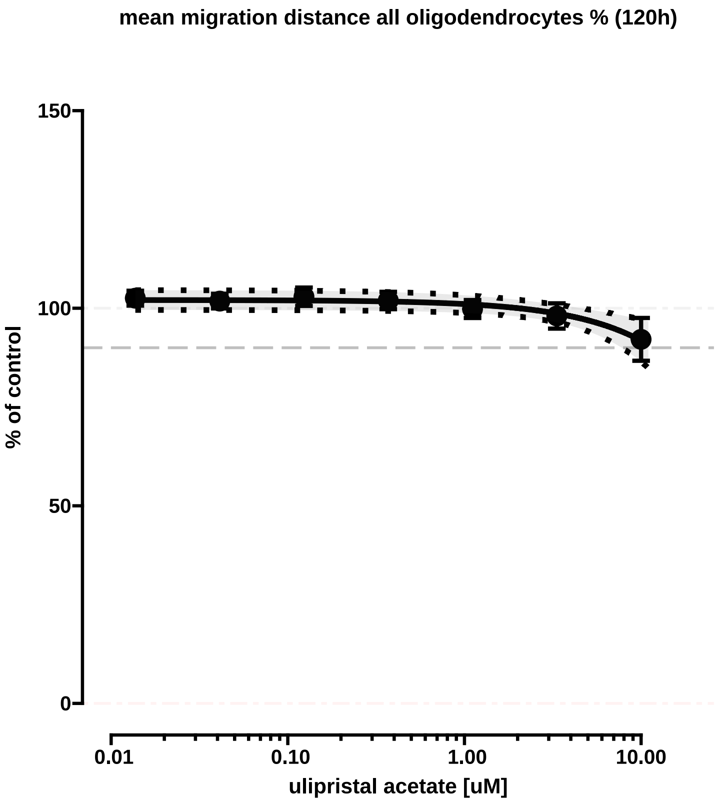

Model: Linear
Model abbr.: lm
Bechmark-Response (BMR): 10

BMCL: NA
BMC: NA
BMCU: NA

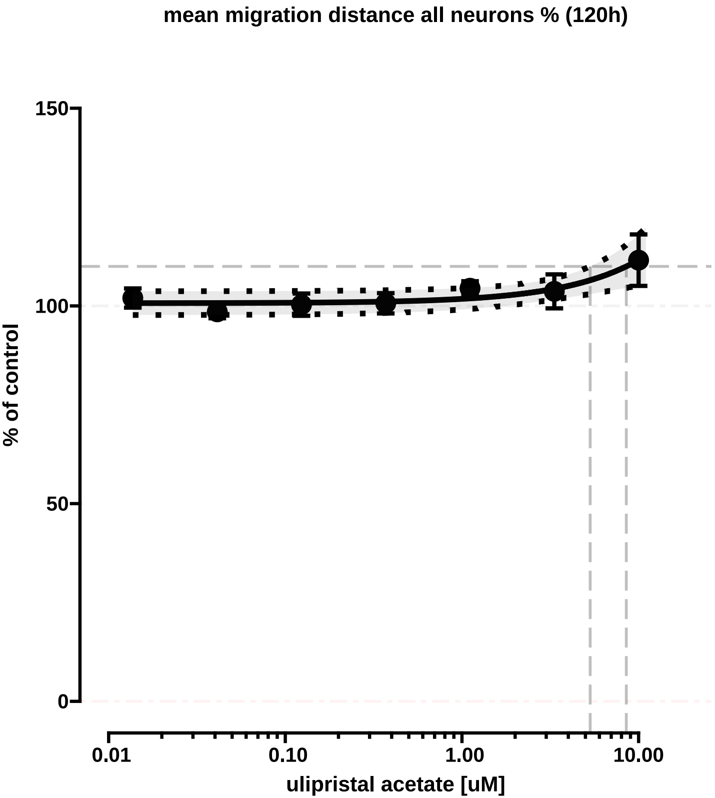

Model: Linear
Model abbr.: lm
Bechmark-Response (BMR): 10

BMCL: 5.32
BMC: 8.52
BMCU: NA

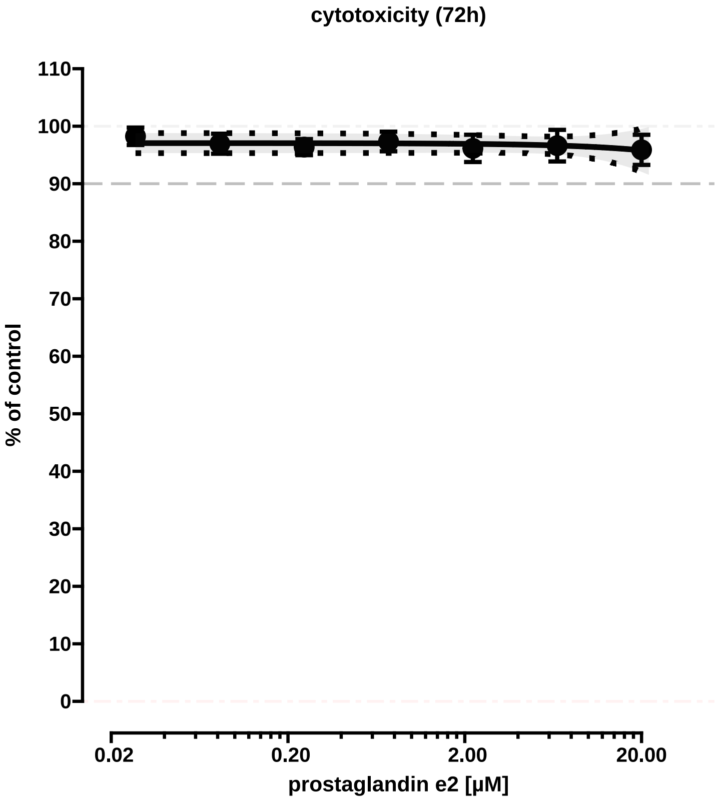

Model: Exponential decay with lower limit at 0
Model abbr.: EXD.2()
Bechmark-Response (BMR): 10

BMCL: NA
BMC: NA
BMCU: NA

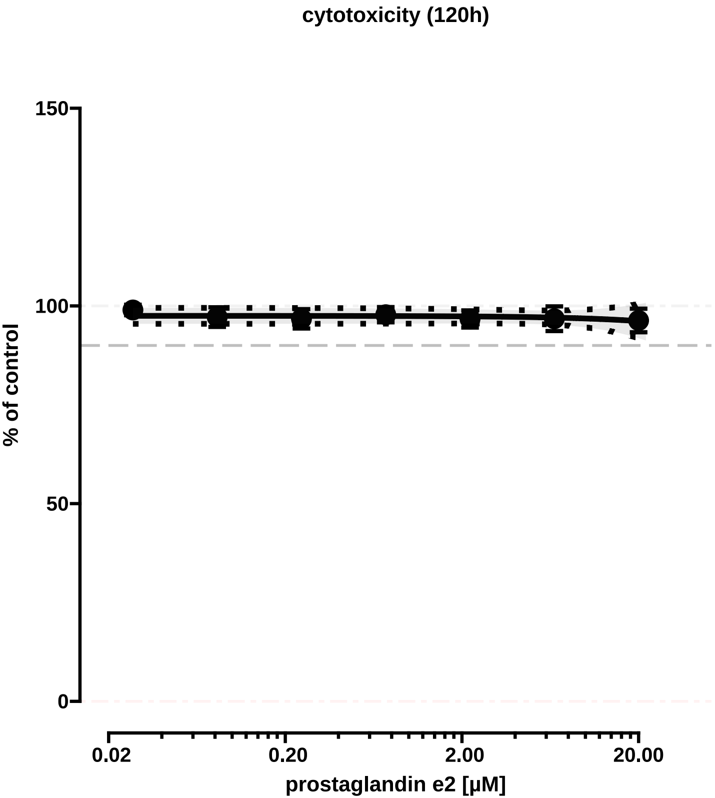

Model: Exponential decay with lower limit at 0
Model abbr.: EXD.2()
Bechmark-Response (BMR): 10

BMCL: NA
BMC: NA
BMCU: NA

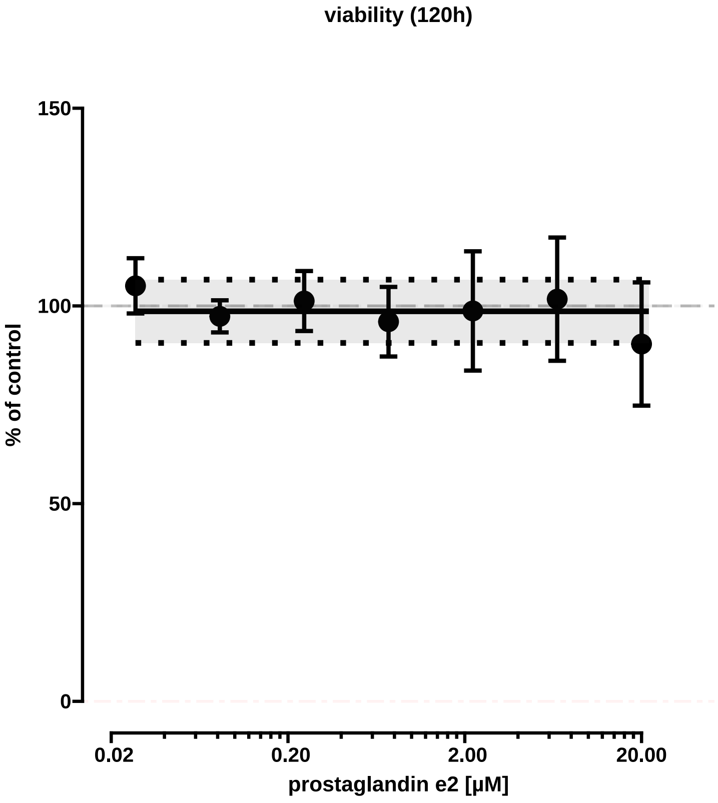

Model: 1-Parameter
Model abbr.: lm.1
Bechmark-Response (BMR): 20

BMCL: NA
BMC: NA
BMCU: NA

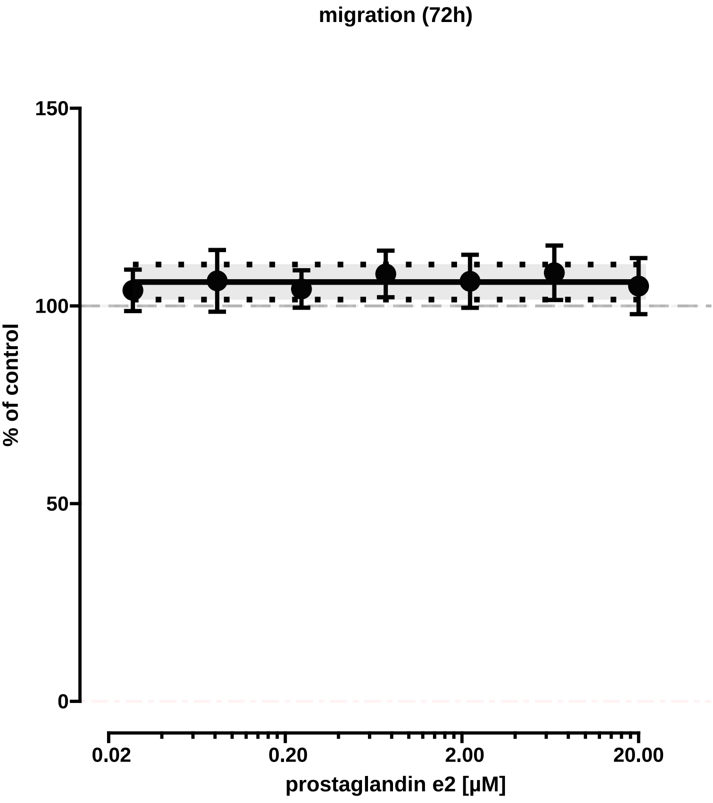

Model: 1-Parameter
Model abbr.: lm.1
Bechmark-Response (BMR): 10

BMCL: NA
BMC: NA
BMCU: NA

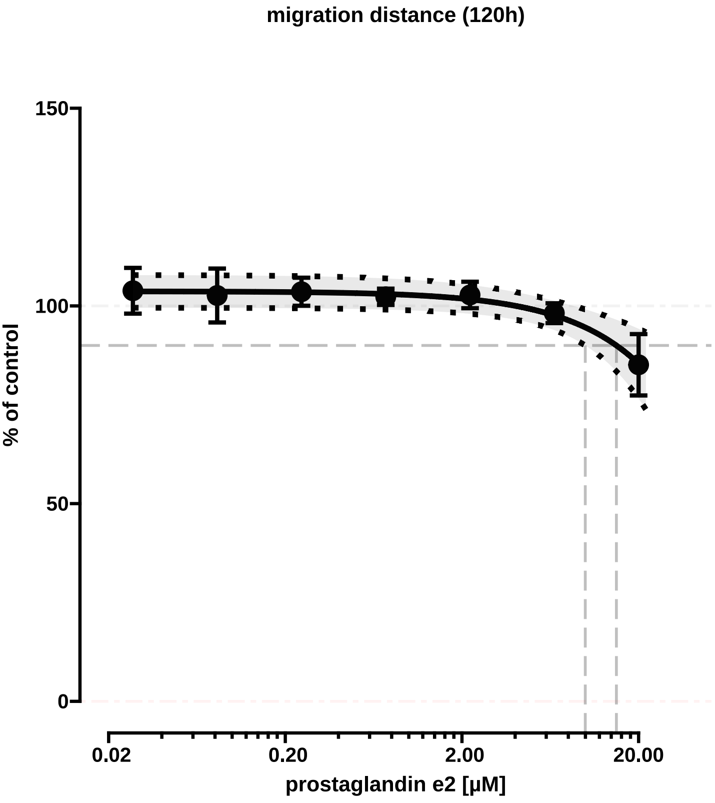

Model: Linear
Model abbr.: lm
Bechmark-Response (BMR): 10

BMCL: 9.98
BMC: 14.977
BMCU: NA

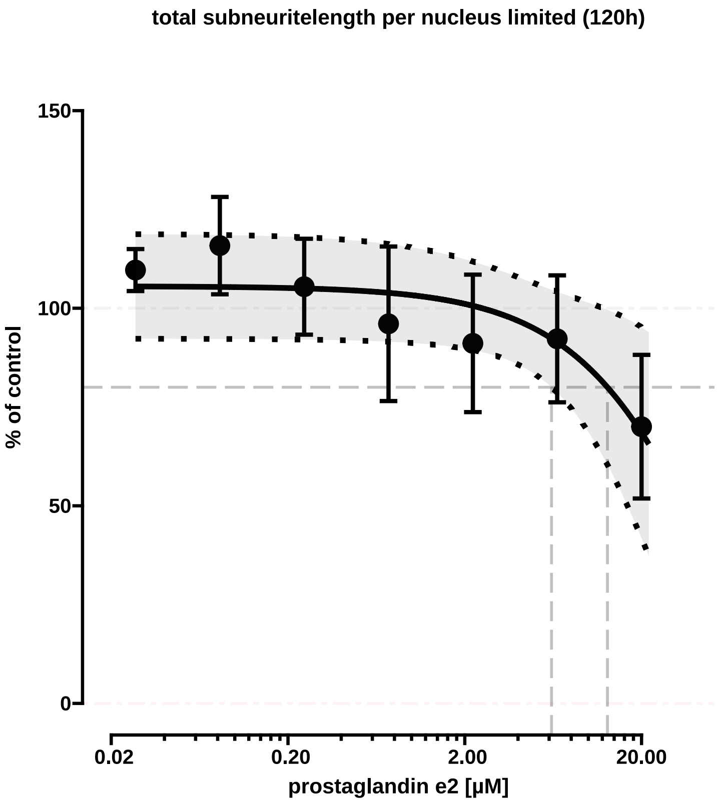

Model: Exponential decay with lower limit at 0
Model abbr.: EXD.2()
Bechmark-Response (BMR): 20

BMCL: 6.191
BMC: 12.824
BMCU: NA

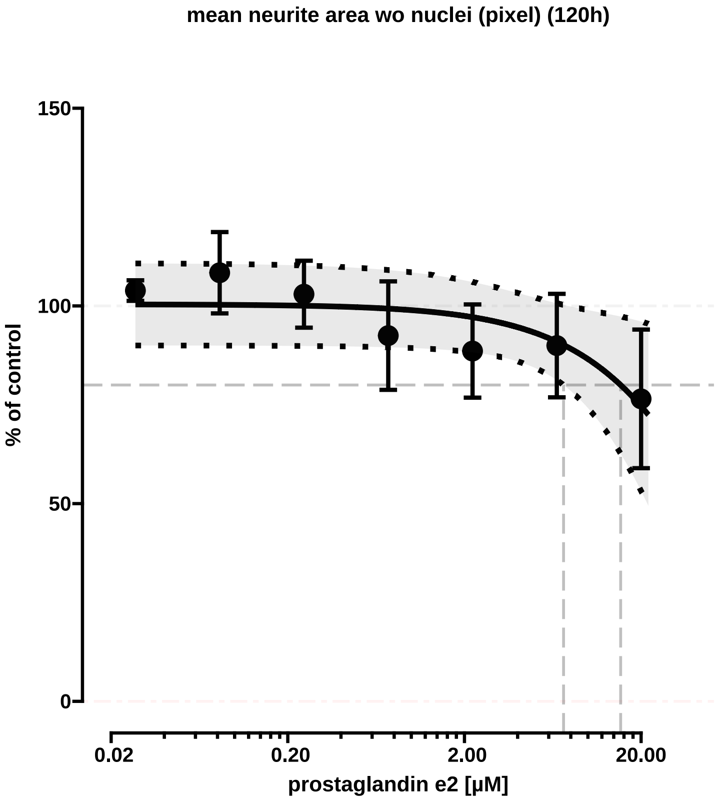

Model: Exponential decay with lower limit at 0
Model abbr.: EXD.2()
Bechmark-Response (BMR): 20

BMCL: 7.278
BMC: 15.321
BMCU: NA

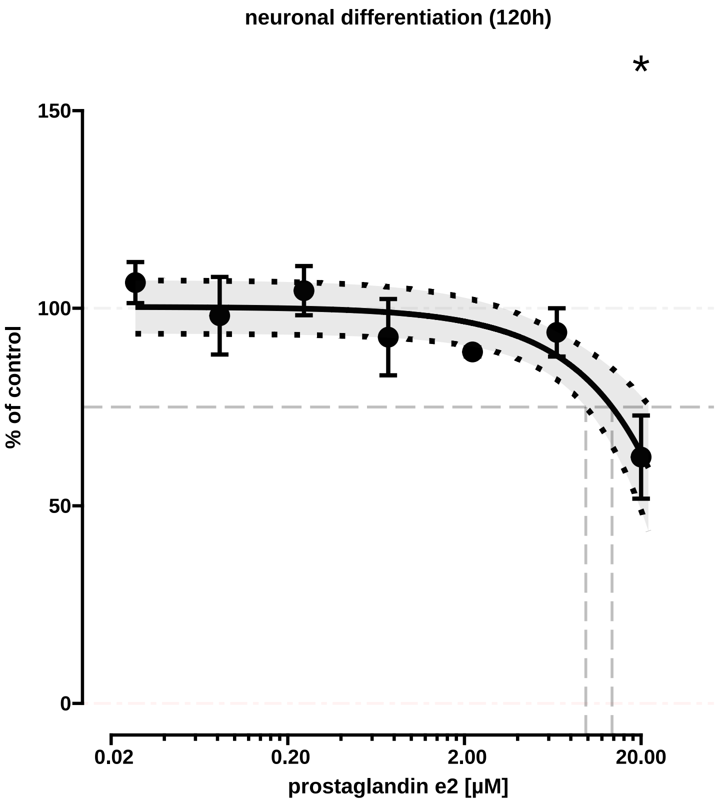

Model: Linear
Model abbr.: lm
Bechmark-Response (BMR): 25

BMCL: 9.729
BMC: 13.693
BMCU: NA

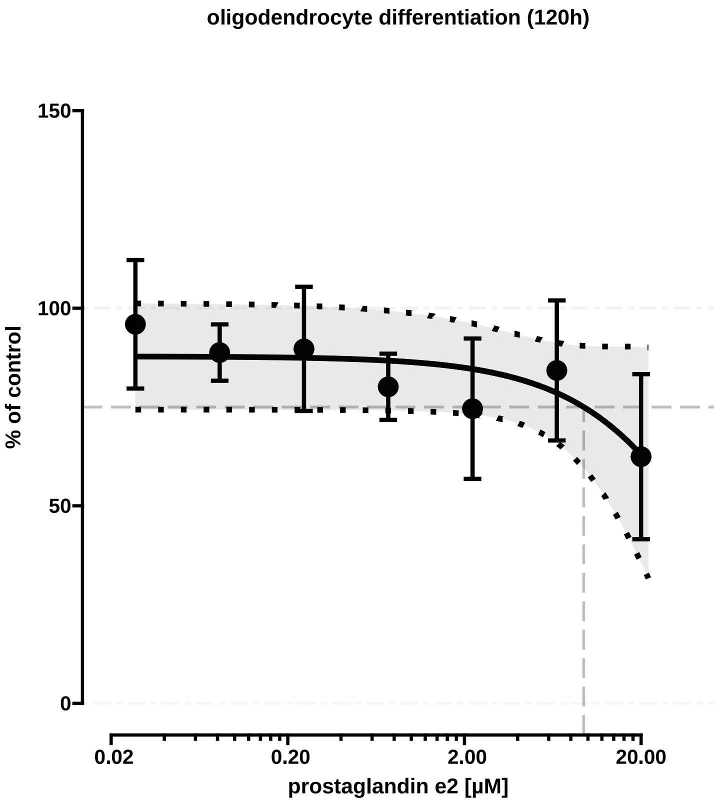

Model: Exponential decay with lower limit at 0
Model abbr.: EXD.2()
Bechmark-Response (BMR): 25

BMCL: NA
BMC: 9.466
BMCU: NA

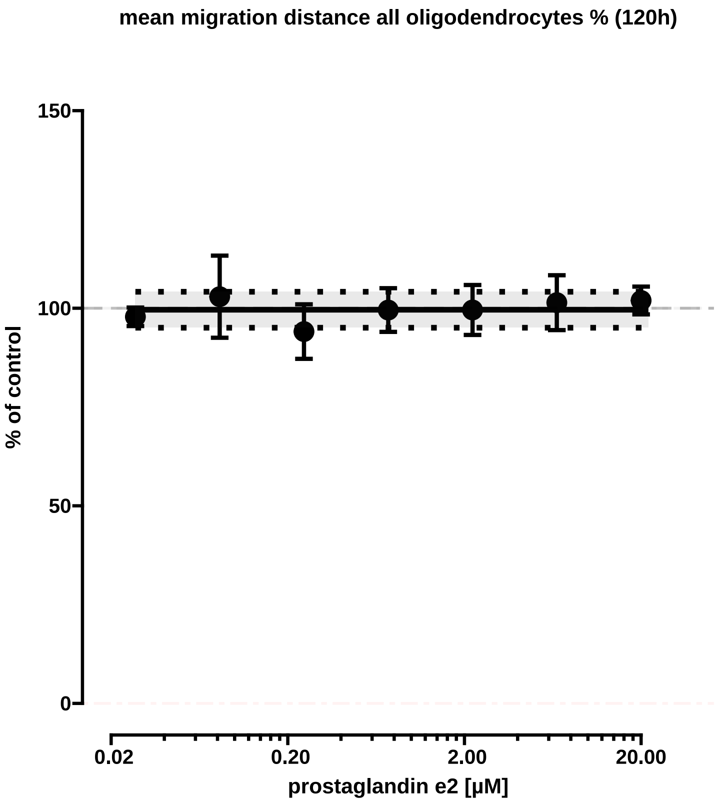

Model: 1-Parameter
Model abbr.: lm.1
Bechmark-Response (BMR): 10

BMCL: NA
BMC: NA
BMCU: NA

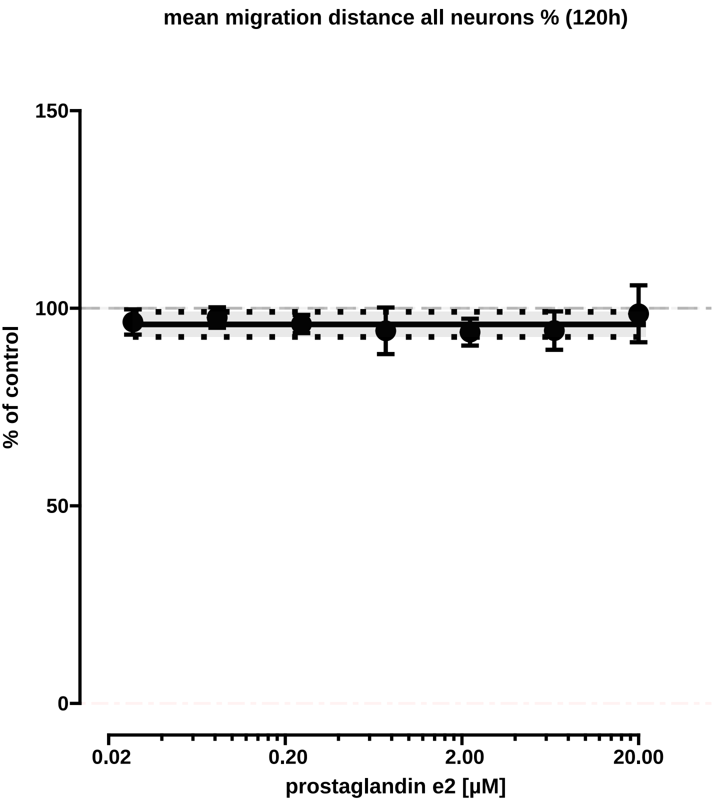

Model: 1-Parameter
Model abbr.: lm.1
Bechmark-Response (BMR): 10

BMCL: NA
BMC: NA
BMCU: NA

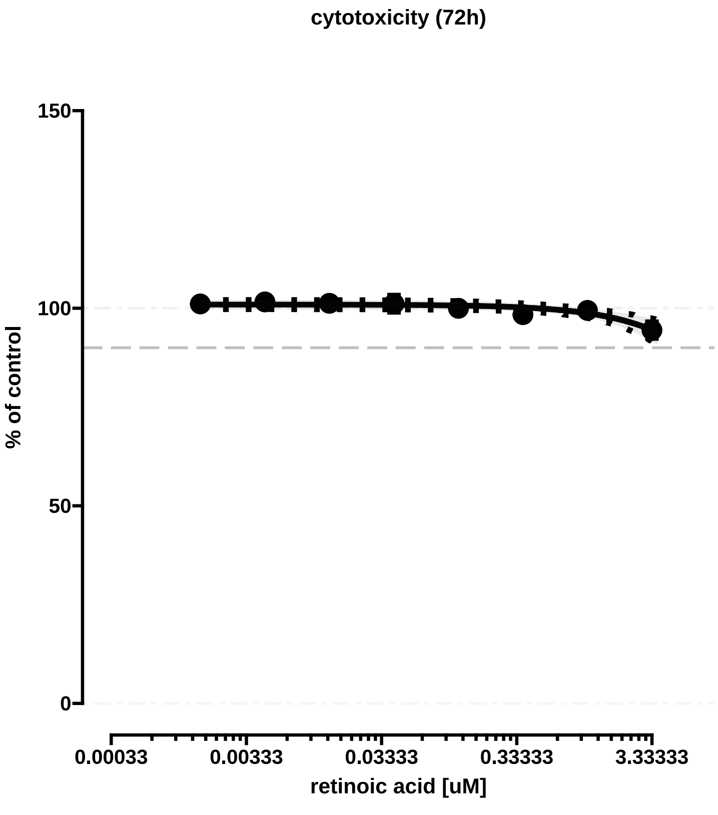

Model: Exponential decay with lower limit at 0
Model abbr.: EXD.2()
Bechmark-Response (BMR): 10

BMCL: NA
BMC: NA
BMCU: NA

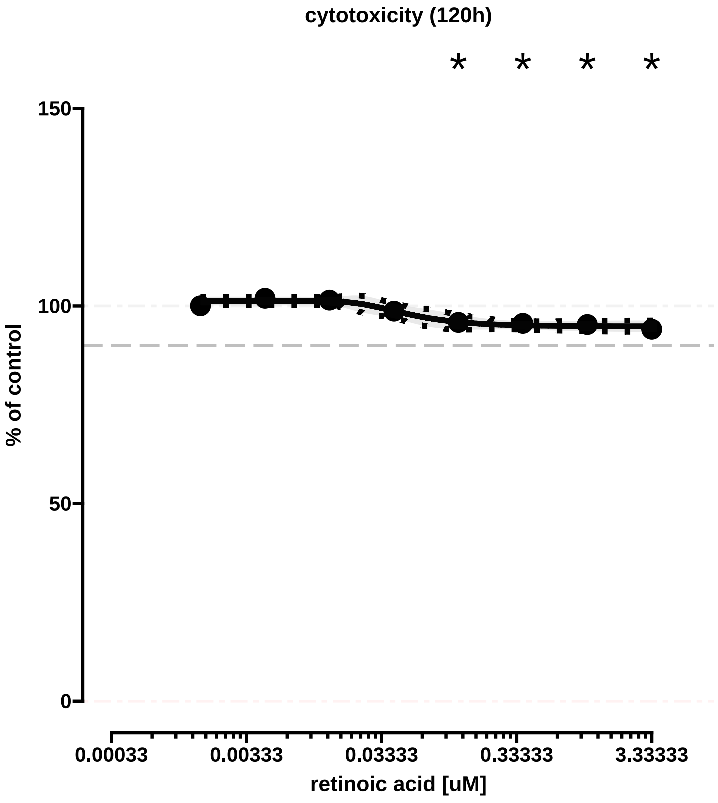

Model: Weibull (type 2)
Model abbr.: W2.4()
Bechmark-Response (BMR): 10

BMCL: NA
BMC: NA
BMCU: NA

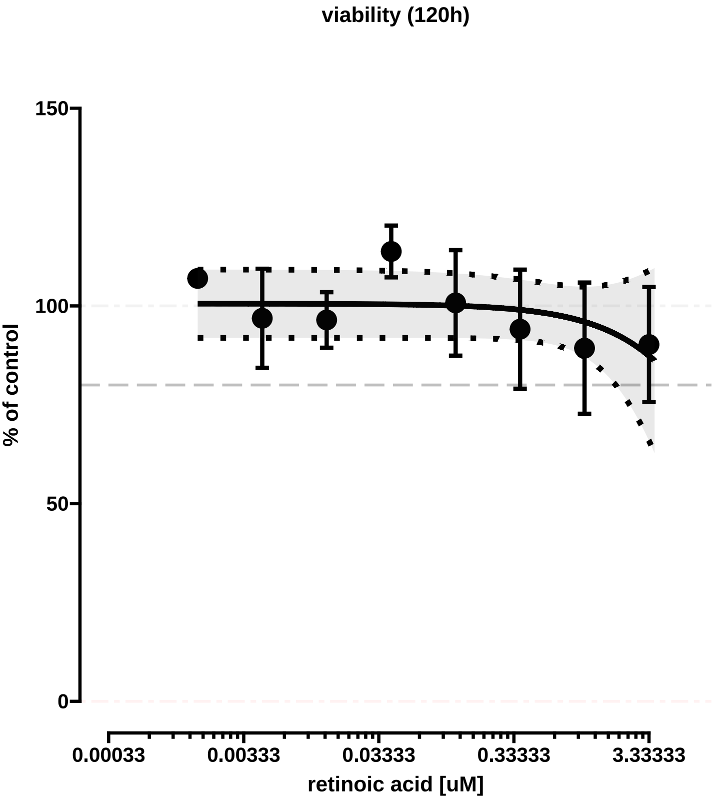

Model: Exponential decay with lower limit at 0
Model abbr.: EXD.2()
Bechmark-Response (BMR): 20

BMCL: NA
BMC: NA
BMCU: NA

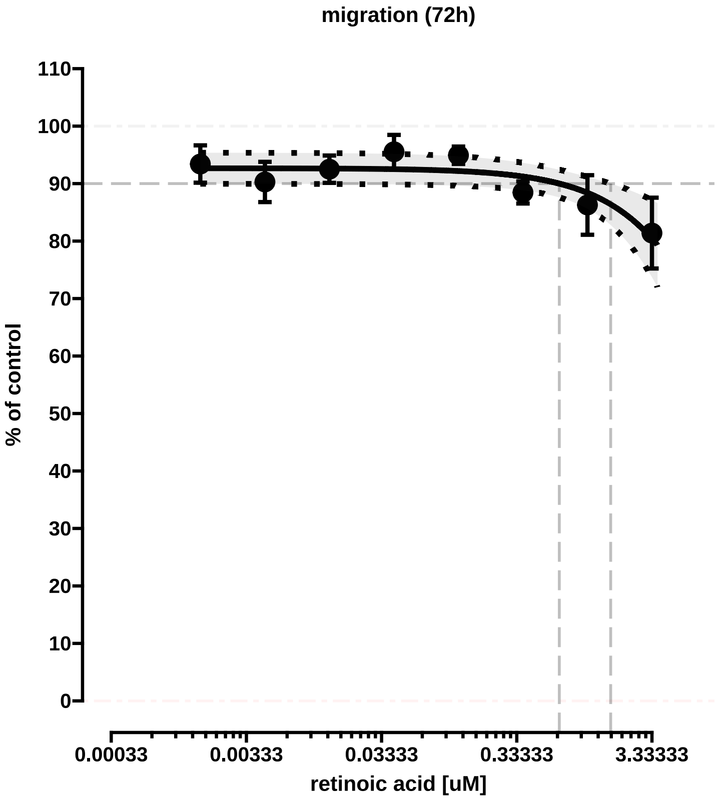

Model: Exponential decay with lower limit at 0
Model abbr.: EXD.2()
Bechmark-Response (BMR): 10

BMCL: NA
BMC: 0.688
BMCU: 1.65

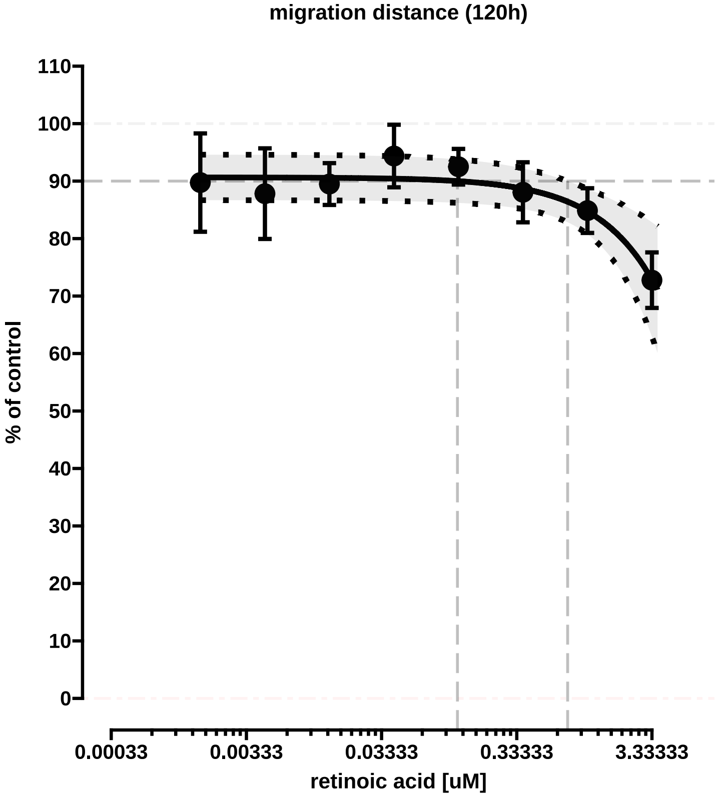

Model: Linear
Model abbr.: lm
Bechmark-Response (BMR): 10

BMCL: NA
BMC: 0.121
BMCU: 0.793

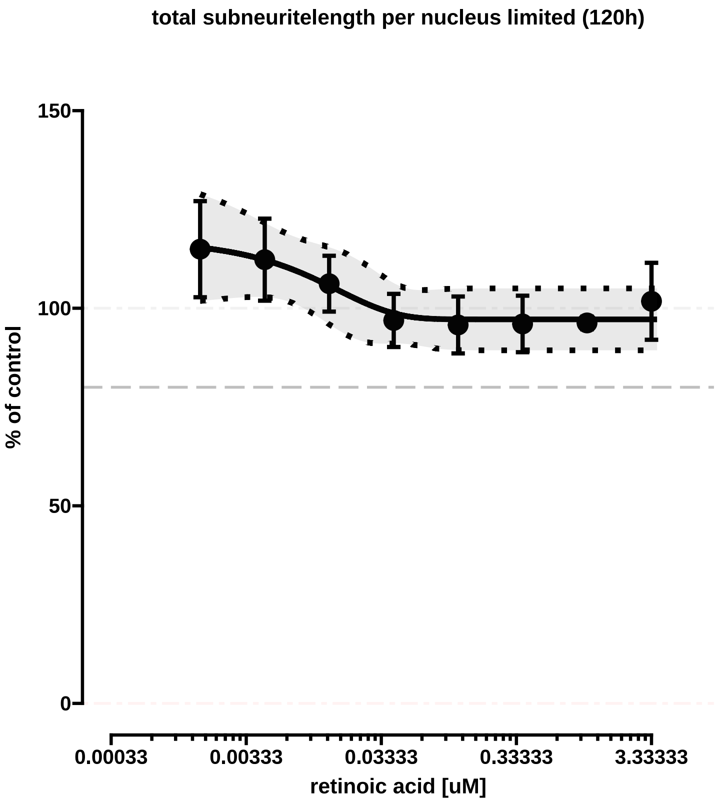

Model: Shifted exponential decay
Model abbr.: EXD.3()
Bechmark-Response (BMR): 20

BMCL: NA
BMC: NA
BMCU: NA

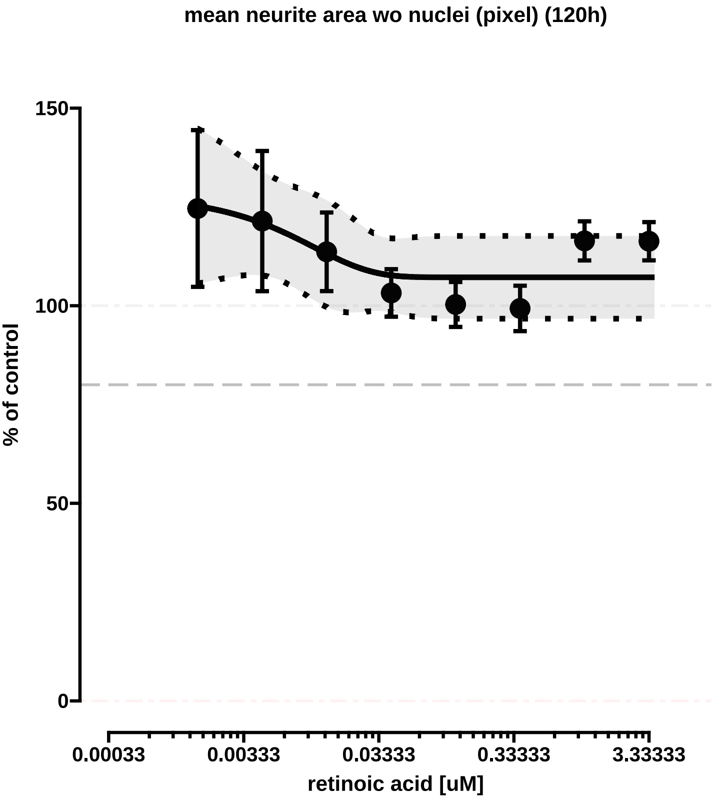

Model: Shifted exponential decay
Model abbr.: EXD.3()
Bechmark-Response (BMR): 20

BMCL: NA
BMC: NA
BMCU: NA

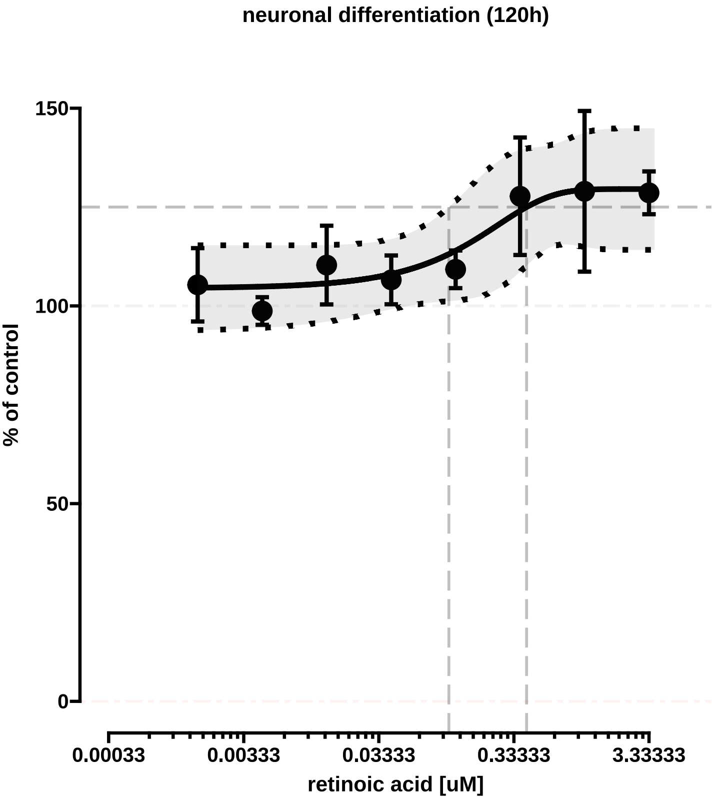

Model: Logistic (ED50 as parameter) with lower limit fixed at 0
Model abbr.: L.3()
Bechmark-Response (BMR): 25

BMCL: 0.11
BMC: 0.414
BMCU: NA

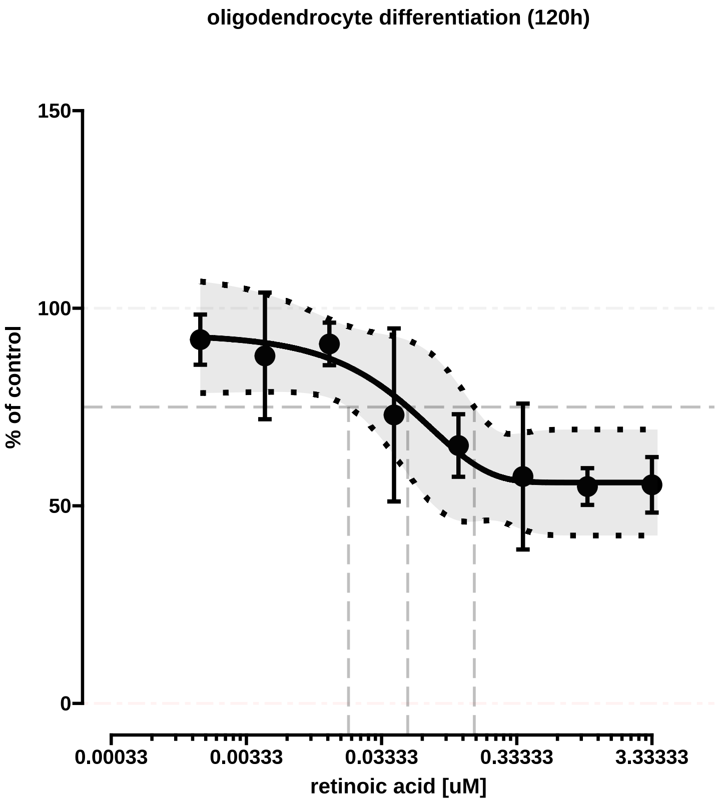

Model: Shifted exponential decay
Model abbr.: EXD.3()
Bechmark-Response (BMR): 25

BMCL: 0.019
BMC: 0.052
BMCU: 0.162

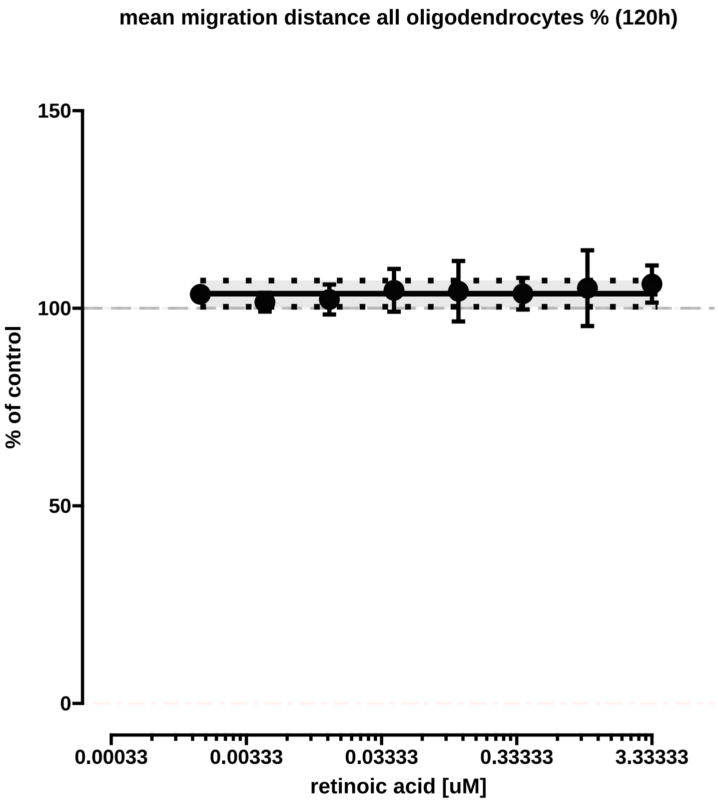

Model: 1-Parameter
Model abbr.: lm.1
Bechmark-Response (BMR): 10

BMCL: NA
BMC: NA
BMCU: NA

mean migration distance all neurons % (120h)

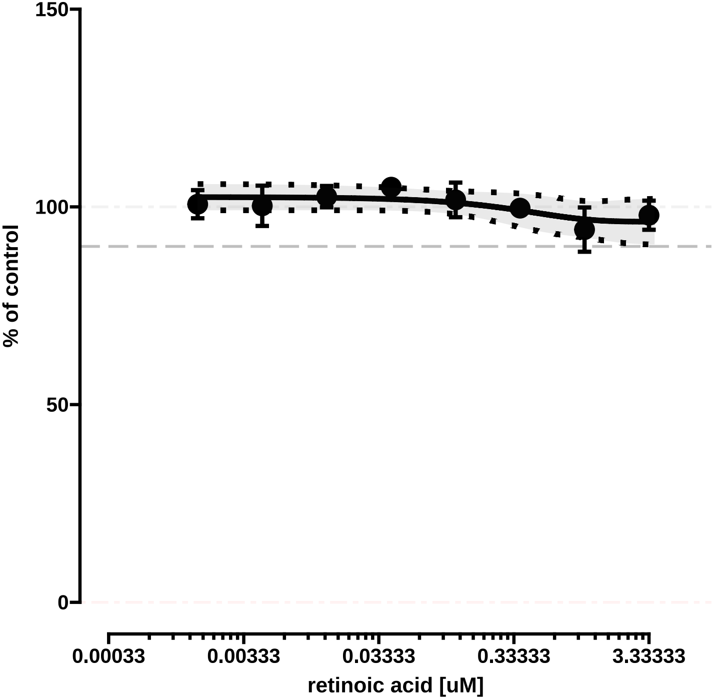

Model: Shifted exponential decay
Model abbr.: EXD.3()
Bechmark-Response (BMR): 10

BMCL: NA
BMC: NA
BMCU: NA

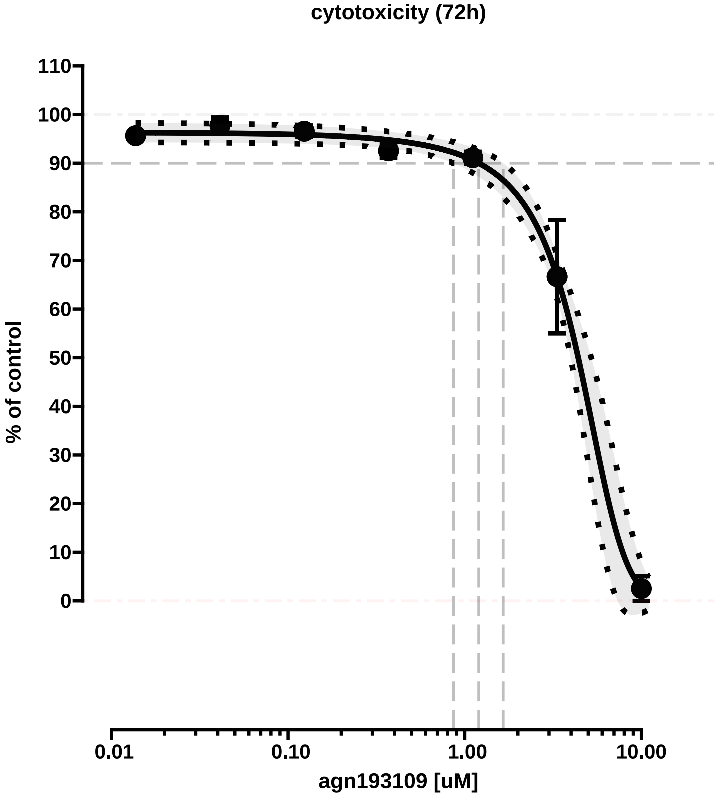

Model: Logistic (ED50 as parameter) with lower limit fixed at 0
Model abbr.: L.3()
Bechmark-Response (BMR): 10

BMCL: 0.863
BMC: 1.201
BMCU: 1.65

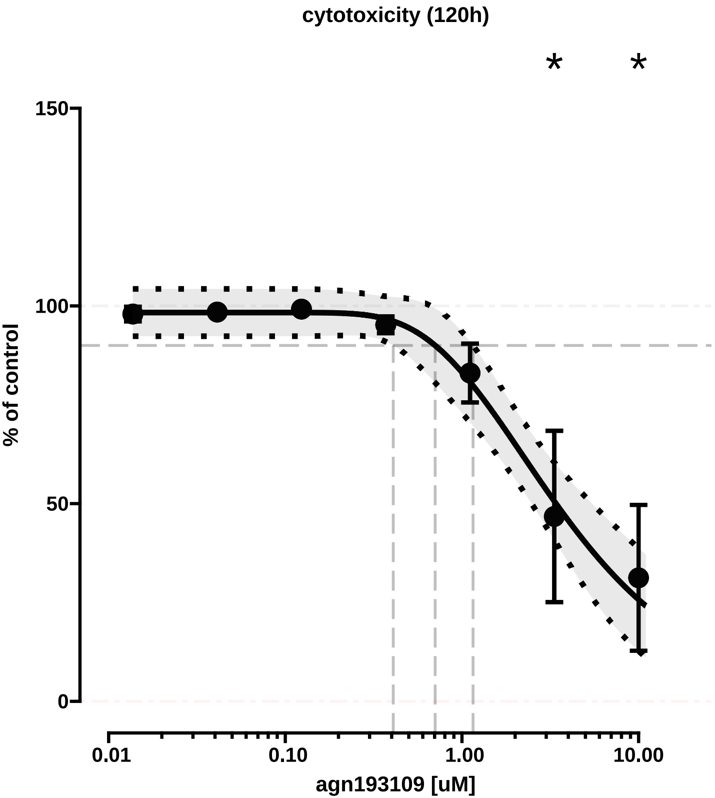

Model: Weibull (type 2) with lower limit at 0
Model abbr.: W2.3()
Bechmark-Response (BMR): 10

BMCL: 0.408
BMC: 0.705
BMCU: 1.155

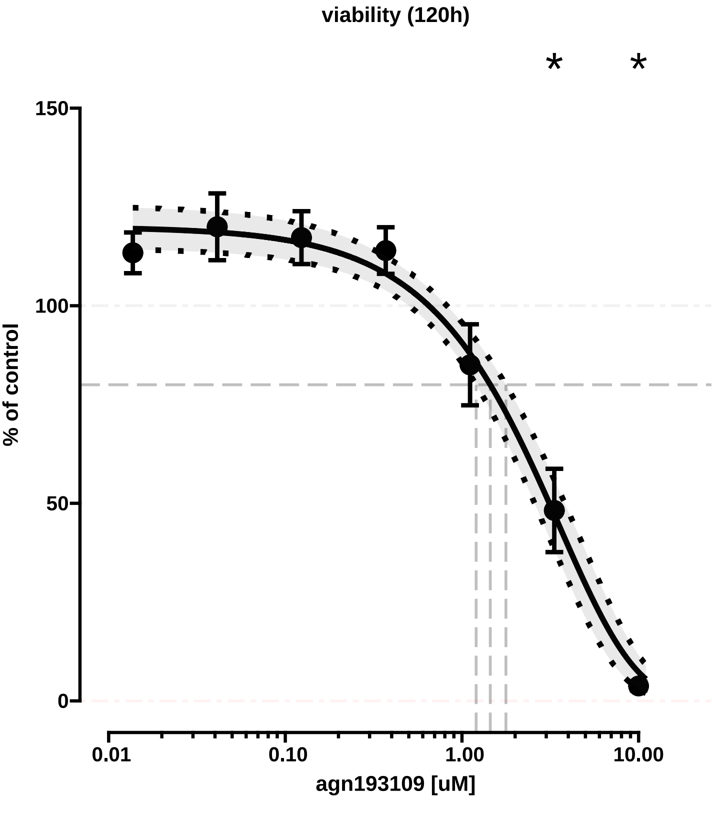

Model: Exponential decay with lower limit at 0
Model abbr.: EXD.2()
Bechmark-Response (BMR): 20

BMCL: 1.203
BMC: 1.446
BMCU: 1.774

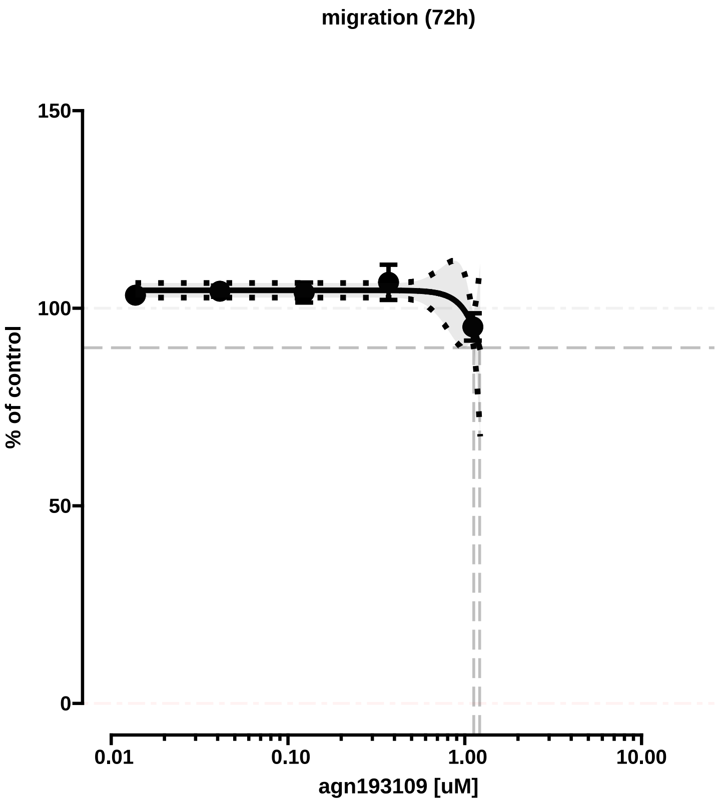

Model: Log-logistic (log(ED50) as parameter) with lower limit at 0
Model abbr.: LL2.3()
Bechmark-Response (BMR): 10

BMCL: 1.124
BMC: 1.214
BMCU: NA

migration distance (120h)

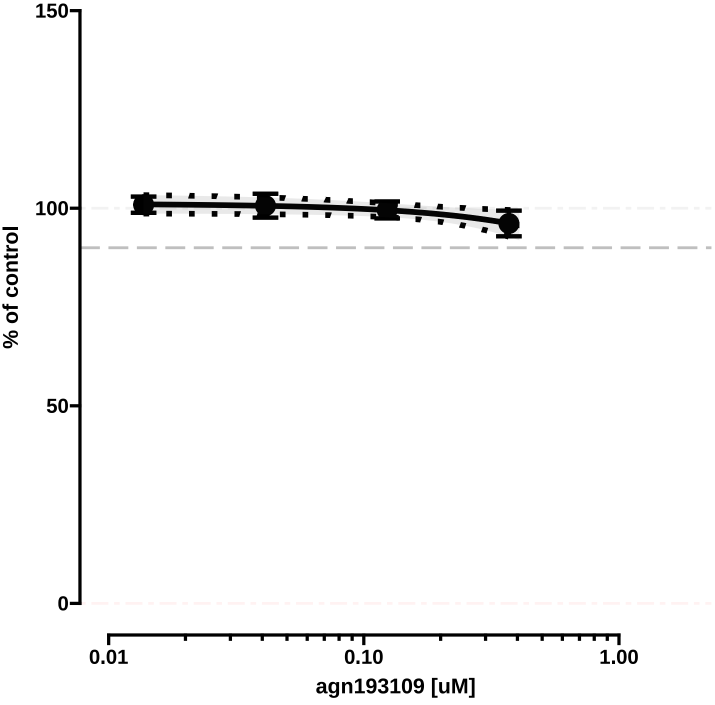

Model: Linear
Model abbr.: lm
Bechmark-Response (BMR): 10

BMCL: NA
BMC: NA
BMCU: NA

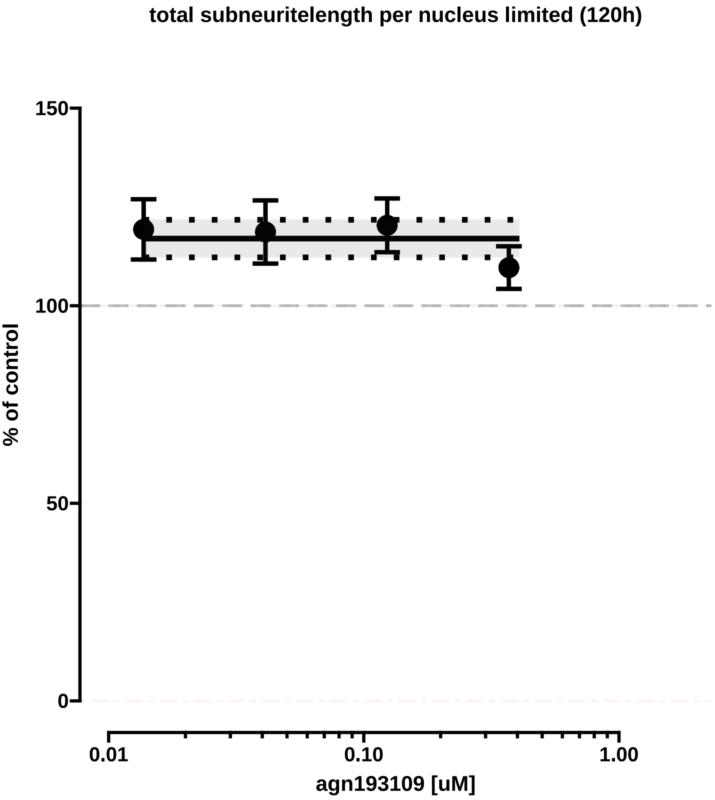

Model: 1-Parameter
Model abbr.: lm.1
Bechmark-Response (BMR): 20

BMCL: NA
BMC: NA
BMCU: NA

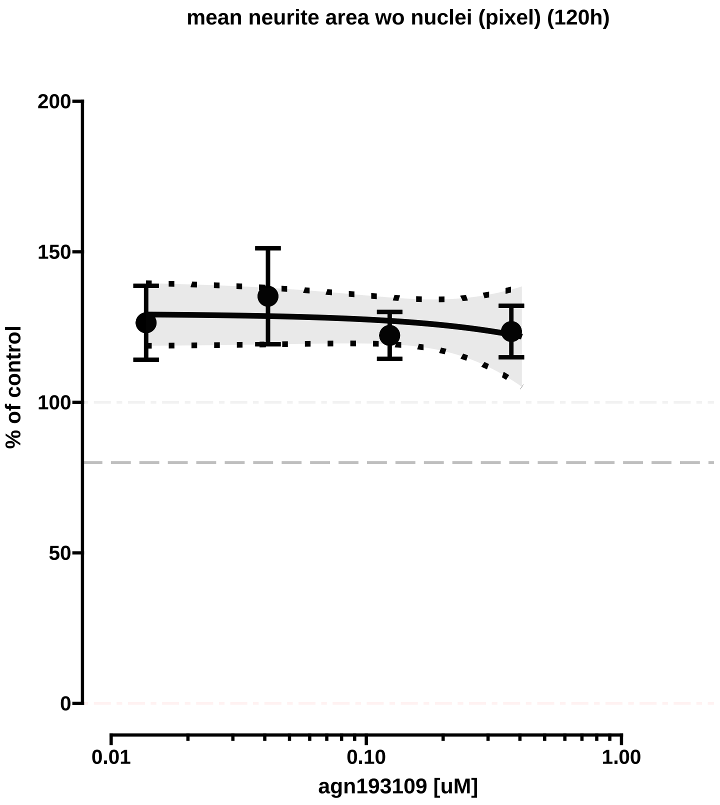

Model: Exponential decay with lower limit at 0
Model abbr.: EXD.2()
Bechmark-Response (BMR): 20

BMCL: NA
BMC: NA
BMCU: NA

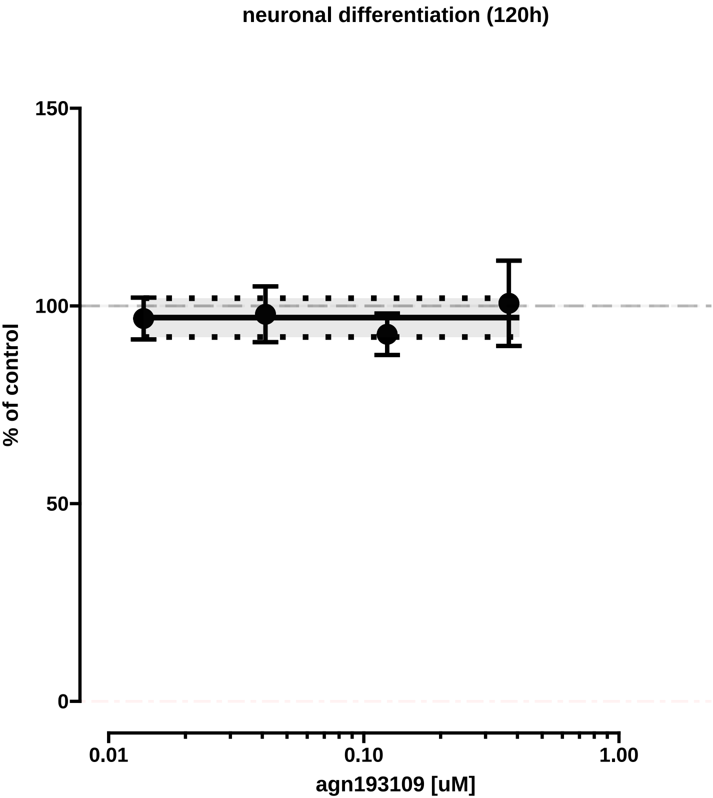

Model: 1-Parameter
Model abbr.: lm.1
Bechmark-Response (BMR): 25

BMCL: NA
BMC: NA
BMCU: NA

oligodendrocyte differentiation (120h)

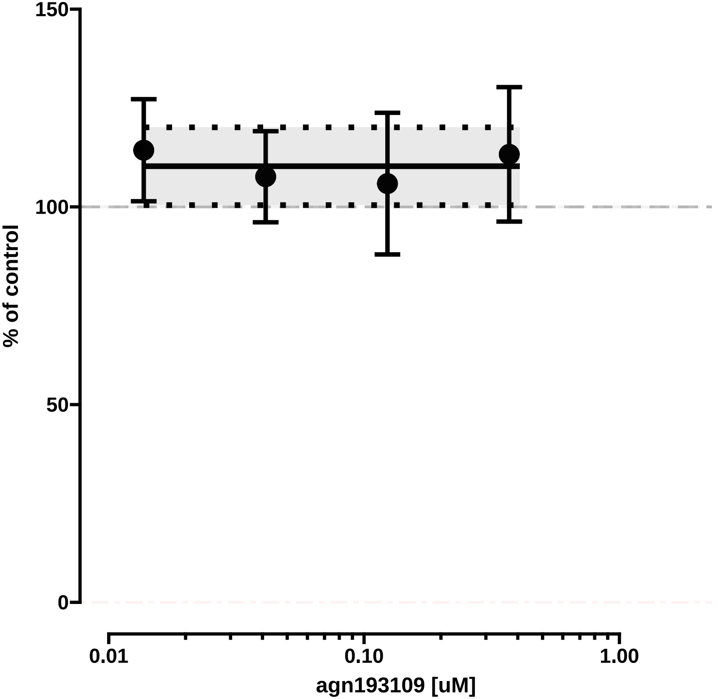

Model: 1-Parameter
Model abbr.: lm.1
Bechmark-Response (BMR): 25

BMCL: NA
BMC: NA
BMCU: NA

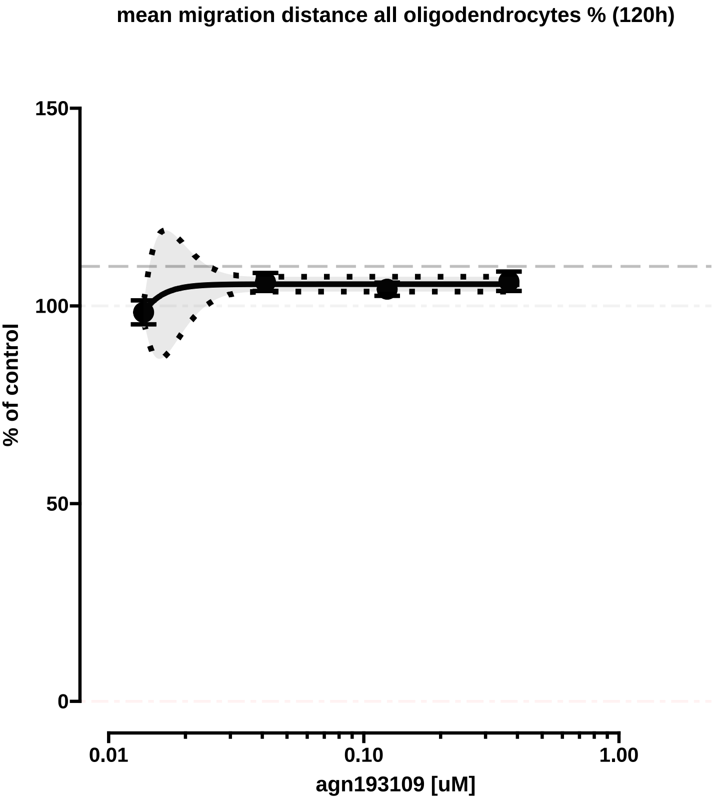

Model: Log-logistic (log(ED50) as parameter) with lower limit at 0
Model abbr.: LL2.3()
Bechmark-Response (BMR): 10

BMCL: NA
BMC: NA
BMCU: NA

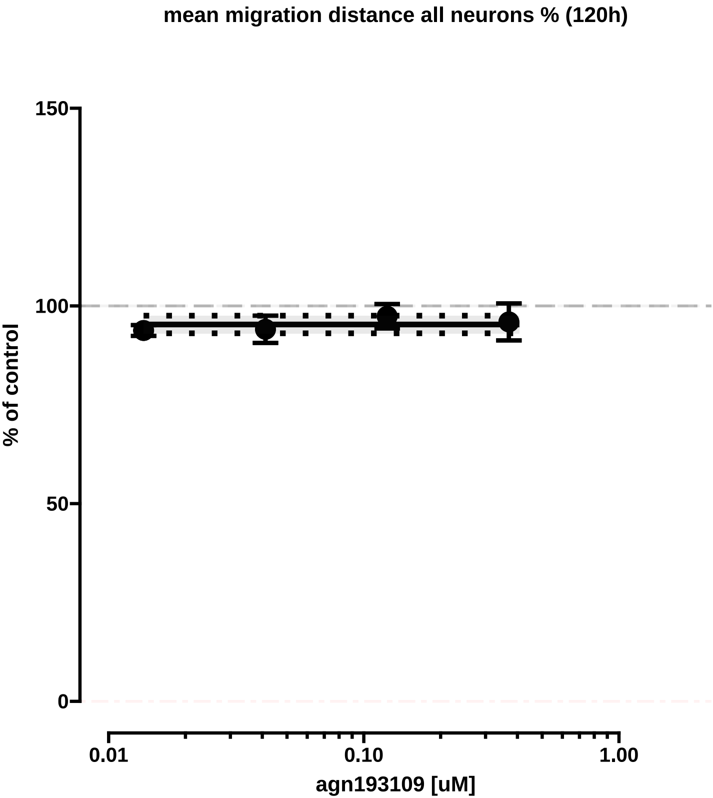

Model: 1-Parameter
Model abbr.: lm.1
Bechmark-Response (BMR): 10

BMCL: NA
BMC: NA
BMCU: NA

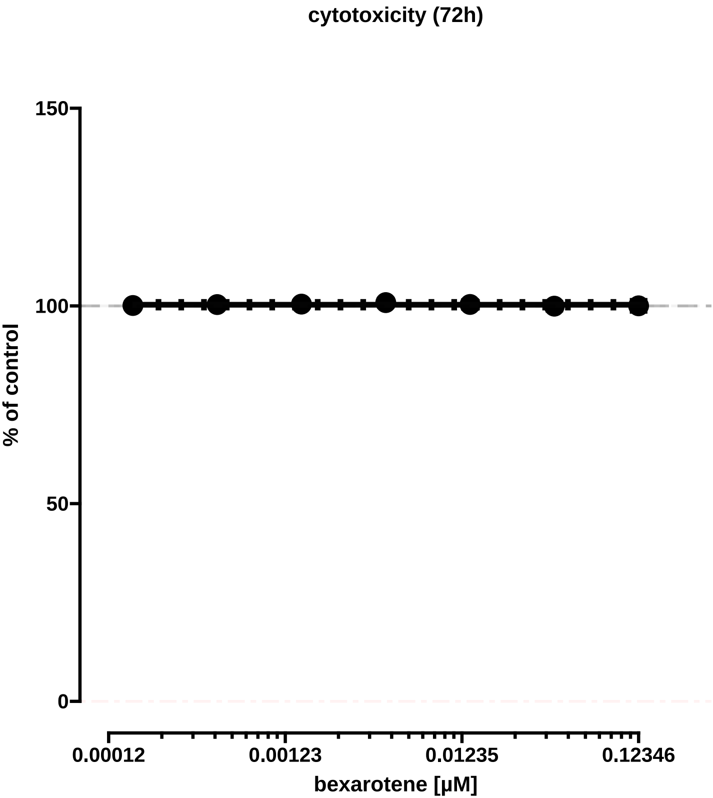

Model: 1-Parameter
Model abbr.: lm.1
Bechmark-Response (BMR): 10

BMCL: NA
BMC: NA
BMCU: NA

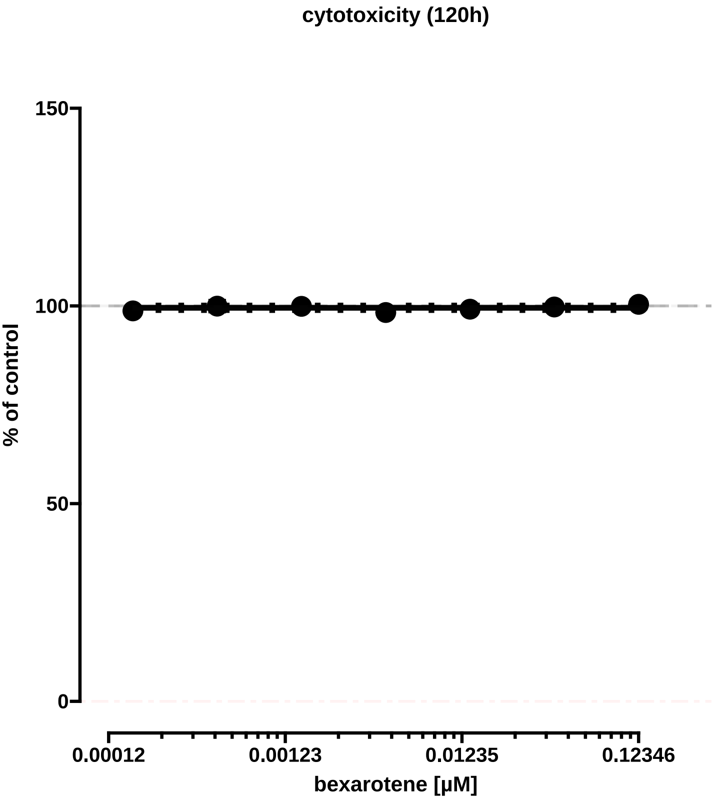

Model: 1-Parameter
Model abbr.: lm.1
Bechmark-Response (BMR): 10

BMCL: NA
BMC: NA
BMCU: NA

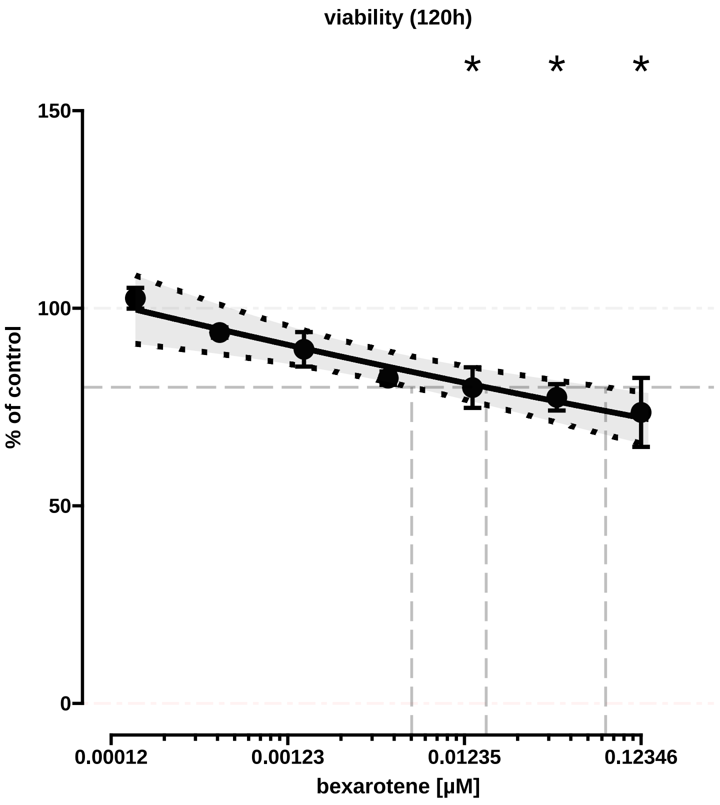

Model: Log-logistic (log(ED50) as parameter) with lower limit at 0
Model abbr.: LL2.3()
Bechmark-Response (BMR): 20

BMCL: 0.006
BMC: 0.016
BMCU: 0.078

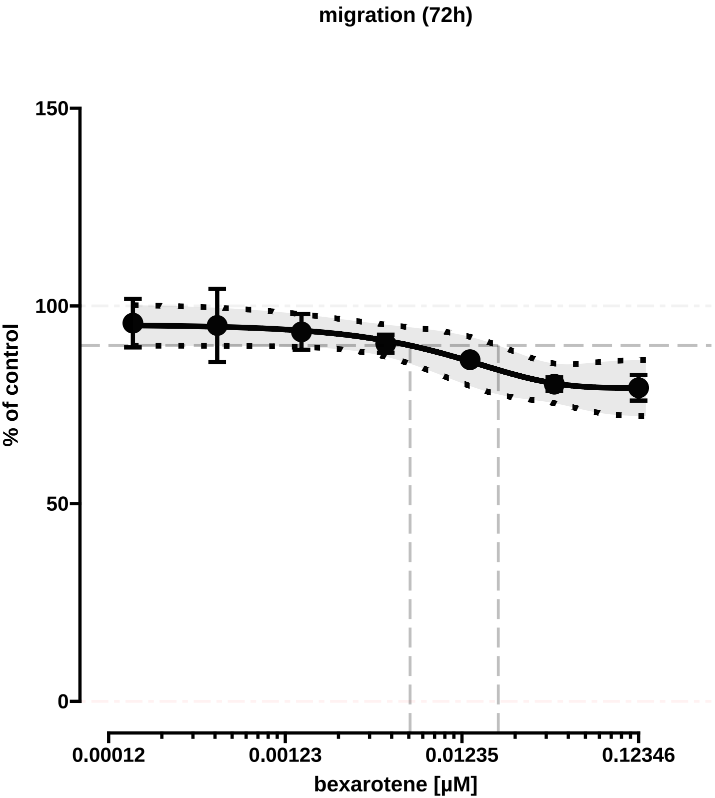

Model: Shifted exponential decay
Model abbr.: EXD.3()
Bechmark-Response (BMR): 10

BMCL: NA
BMC: 0.006
BMCU: 0.02

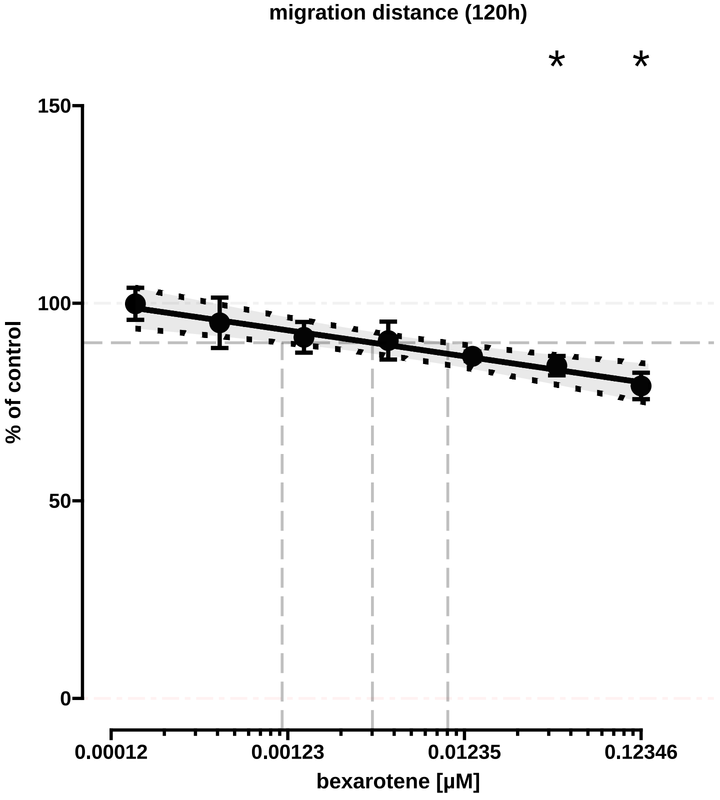

Model: Weibull (type 1) with lower limit at 0
Model abbr.: W1.3()
Bechmark-Response (BMR): 10

BMCL: 0.001
BMC: 0.004
BMCU: 0.01

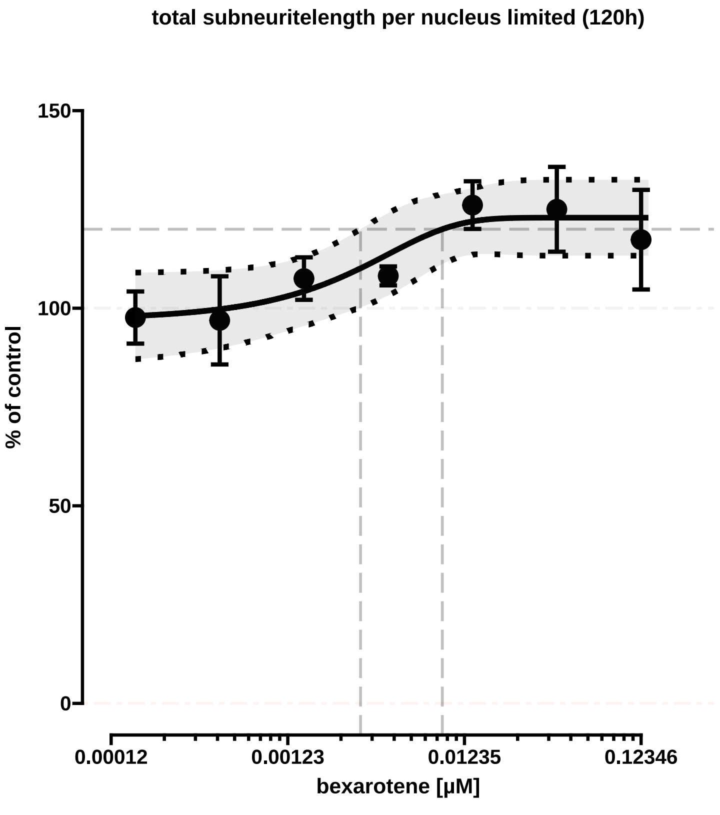

Model: Logistic (ED50 as parameter) with lower limit fixed at 0
Model abbr.: L.3()
Bechmark-Response (BMR): 20

BMCL: 0.003
BMC: 0.009
BMCU: NA

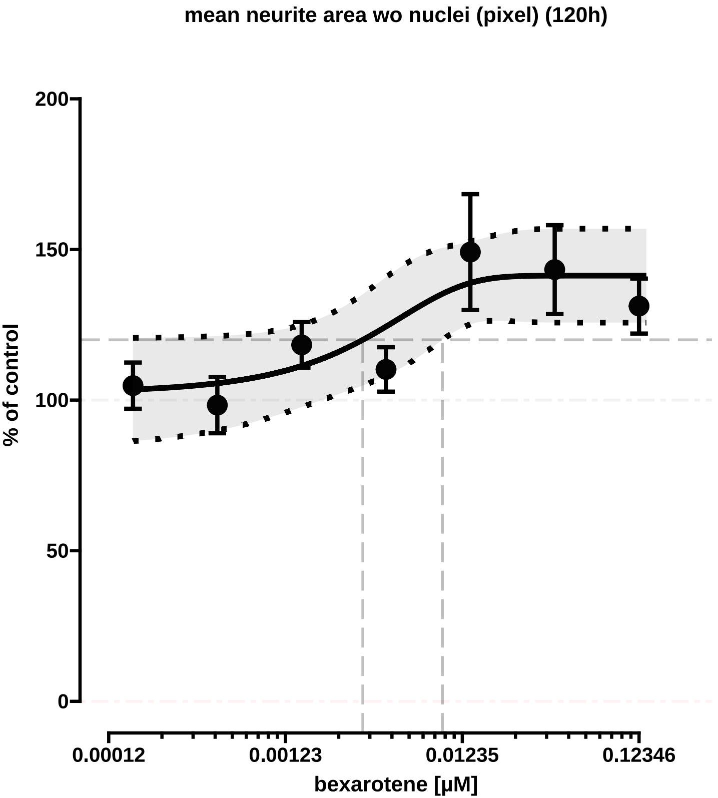

Model: Logistic (ED50 as parameter) with lower limit fixed at 0
Model abbr.: L.3()
Bechmark-Response (BMR): 20

BMCL: NA
BMC: 0.003
BMCU: 0.01

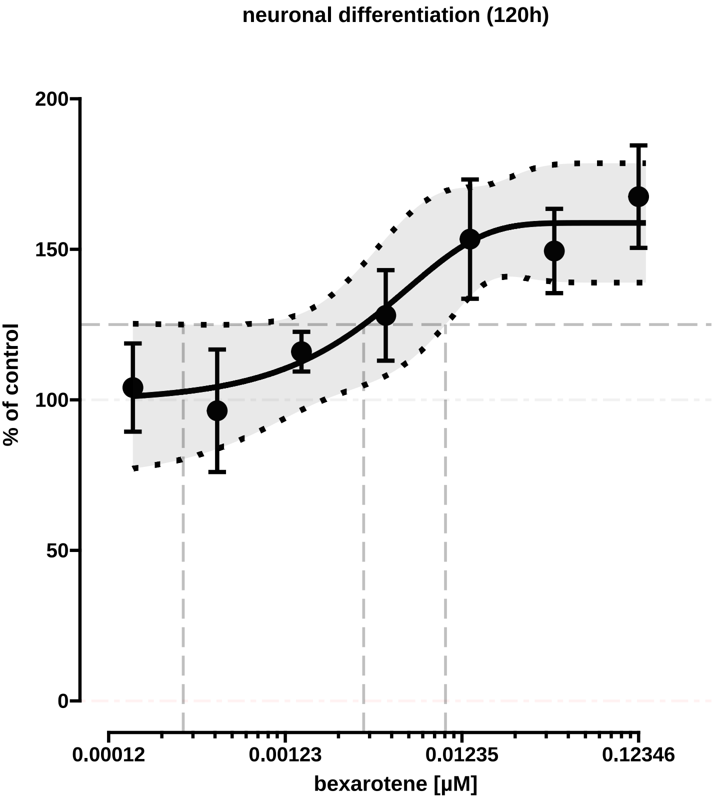

Model: Shifted exponential decay
Model abbr.: EXD.3()
Bechmark-Response (BMR): 25

BMCL: 0
BMC: 0.003
BMCU: 0.01

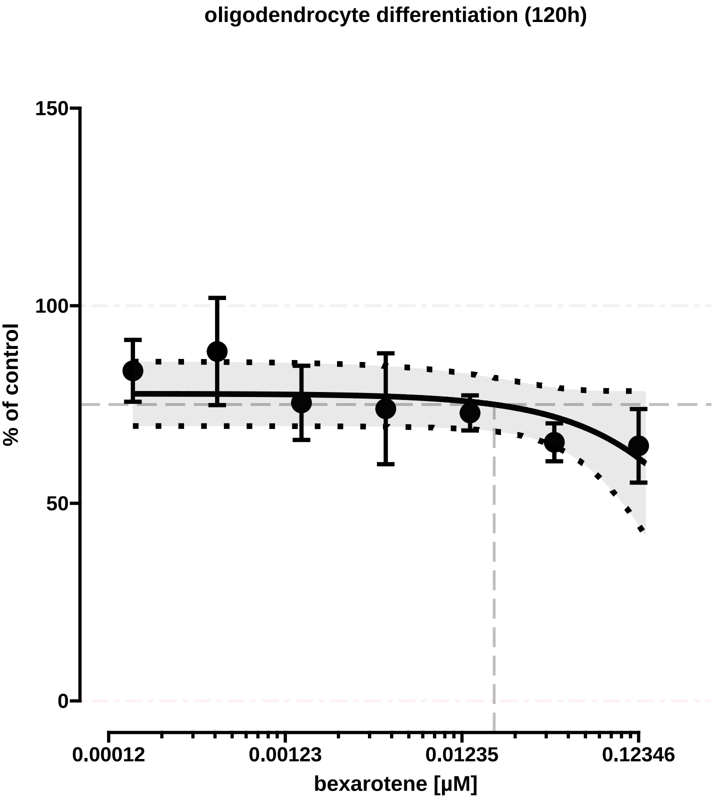

Model: Exponential decay with lower limit at 0
Model abbr.: EXD.2()
Bechmark-Response (BMR): 25

BMCL: NA
BMC: 0.019
BMCU: NA

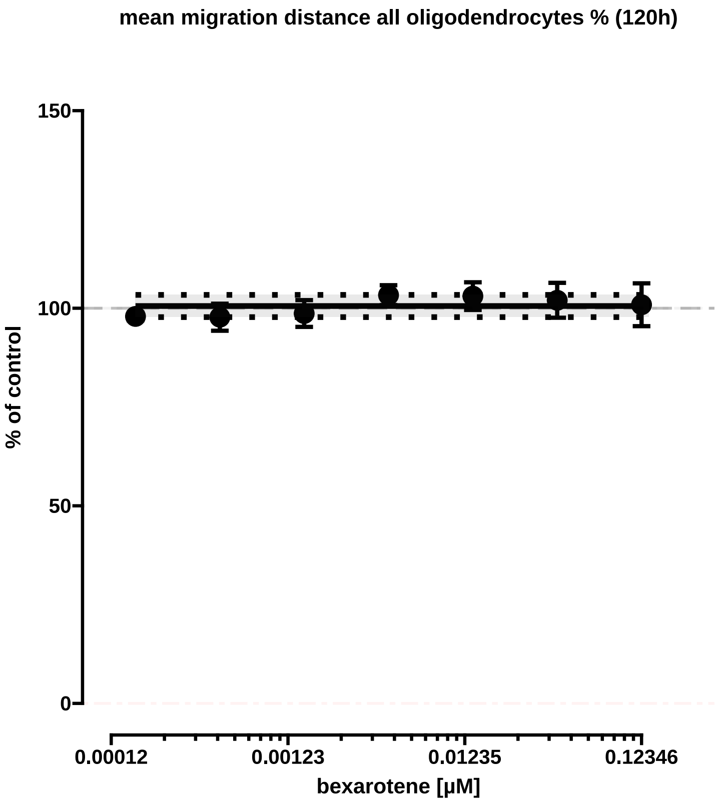

Model: 1-Parameter
Model abbr.: lm.1
Bechmark-Response (BMR): 10

BMCL: NA
BMC: NA
BMCU: NA

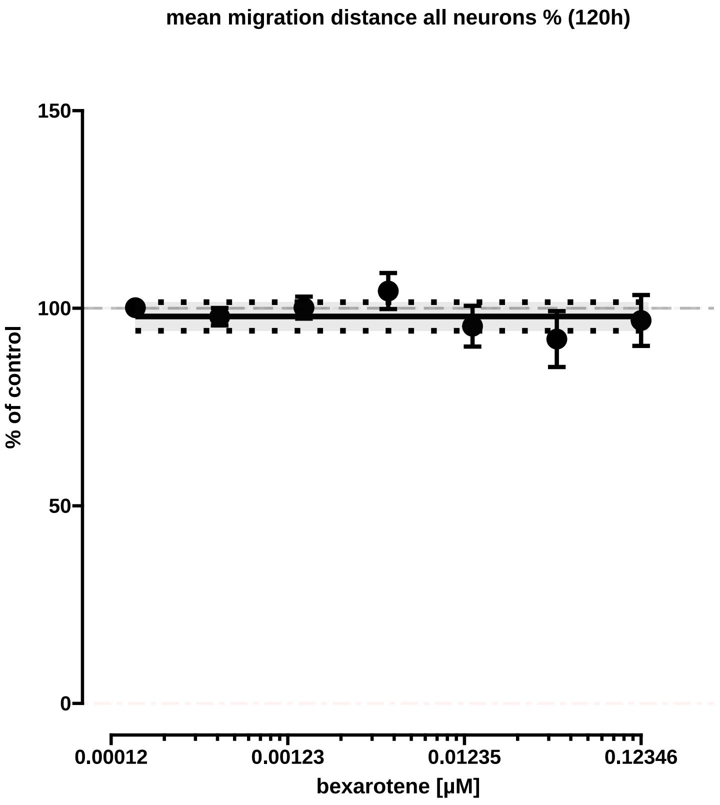

Model: 1-Parameter
Model abbr.: lm.1
Bechmark-Response (BMR): 10

BMCL: NA
BMC: NA
BMCU: NA

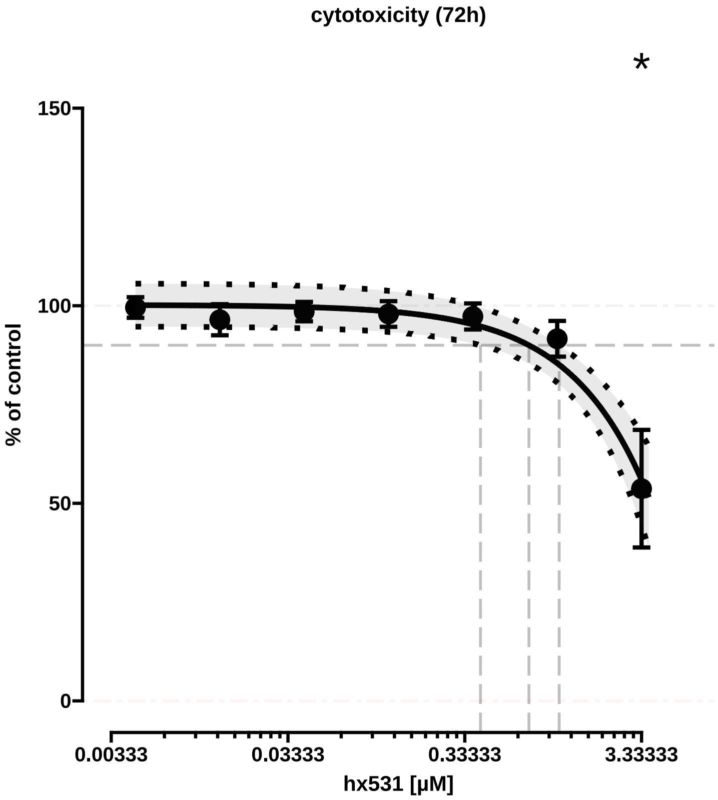

Model: Linear
Model abbr.: lm
Bechmark-Response (BMR): 10

BMCL: 0.409
BMC: 0.769
BMCU: 1.14

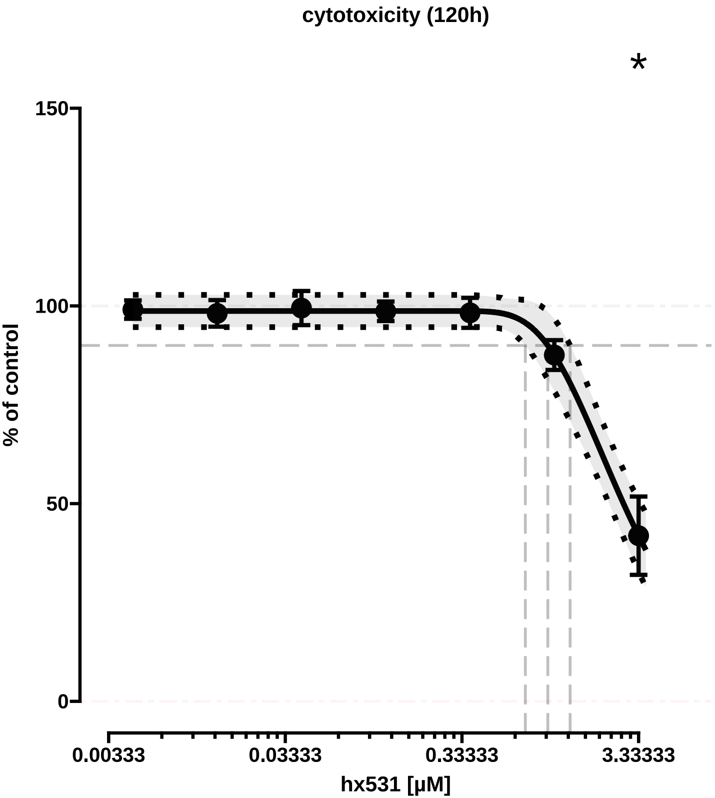

Model: Weibull (type 2) with lower limit at 0
Model abbr.: W2.3()
Bechmark-Response (BMR): 10

BMCL: 0.762
BMC: 1.021
BMCU: 1.365

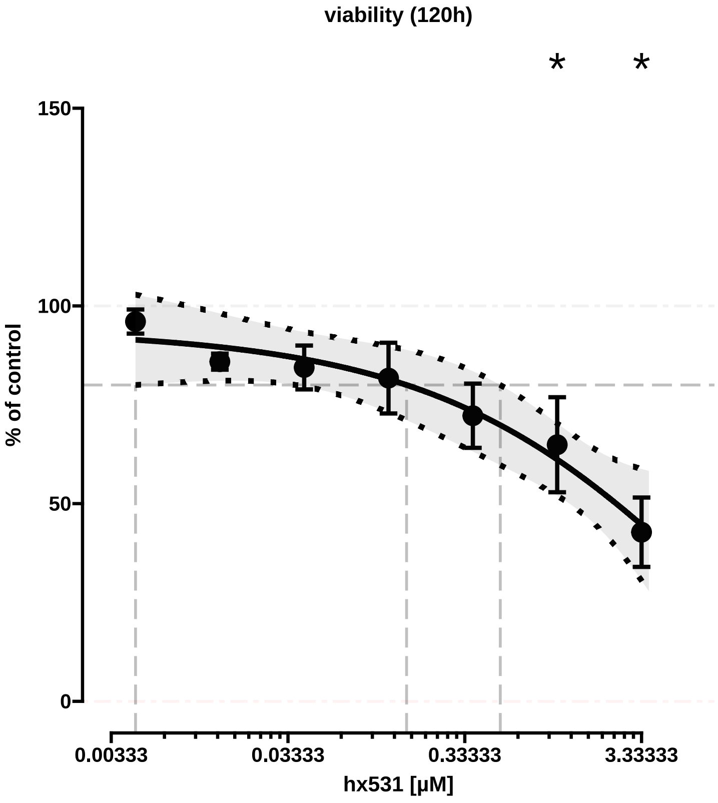

Model: Weibull (type 1) with lower limit at 0
Model abbr.: W1.3()
Bechmark-Response (BMR): 20

BMCL: 0.005
BMC: 0.156
BMCU: 0.529

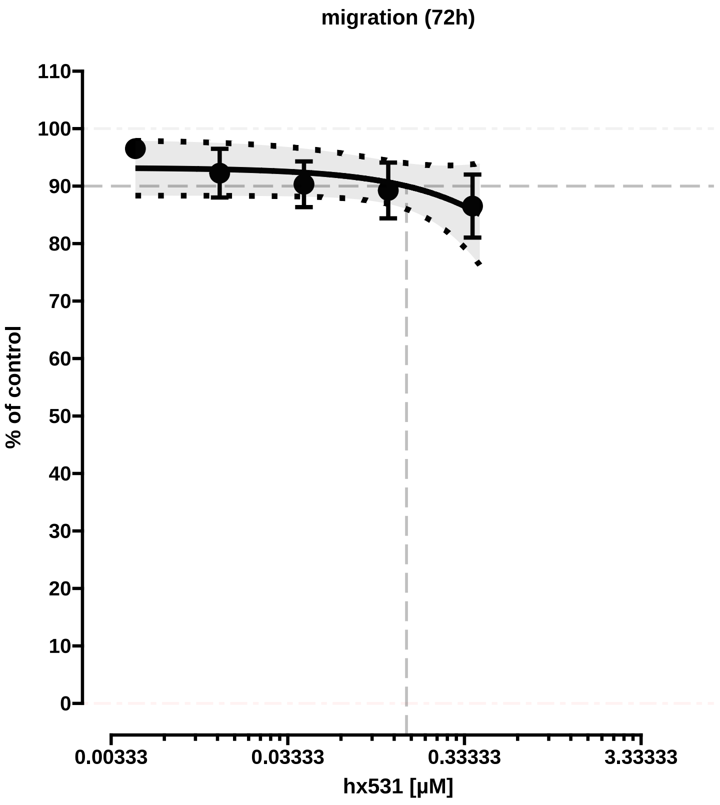

Model: Exponential decay with lower limit at 0
Model abbr.: EXD.2()
Bechmark-Response (BMR): 10

BMCL: NA
BMC: 0.157
BMCU: NA

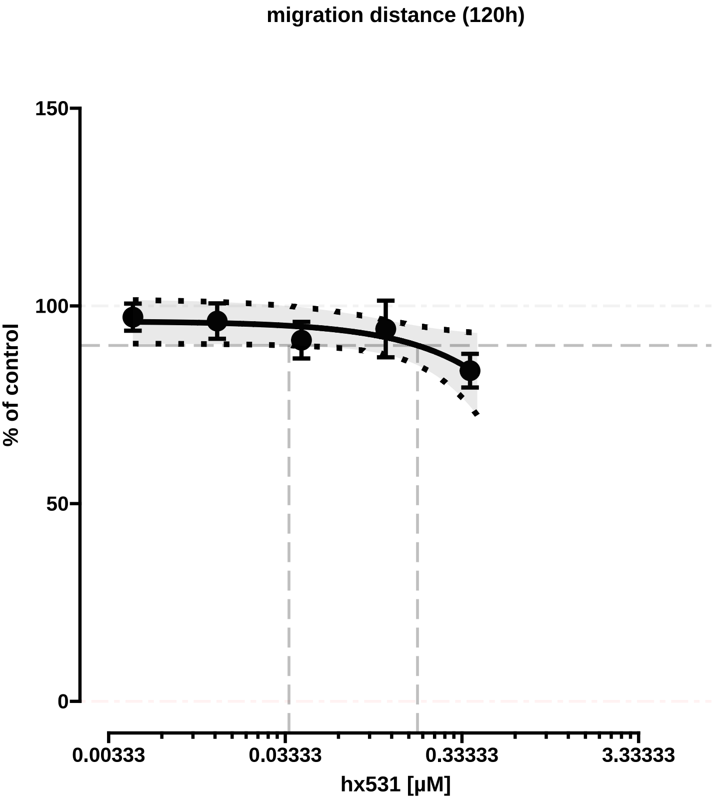

Model: Linear
Model abbr.: lm
Bechmark-Response (BMR): 10

BMCL: 0.035
BMC: 0.187
BMCU: NA

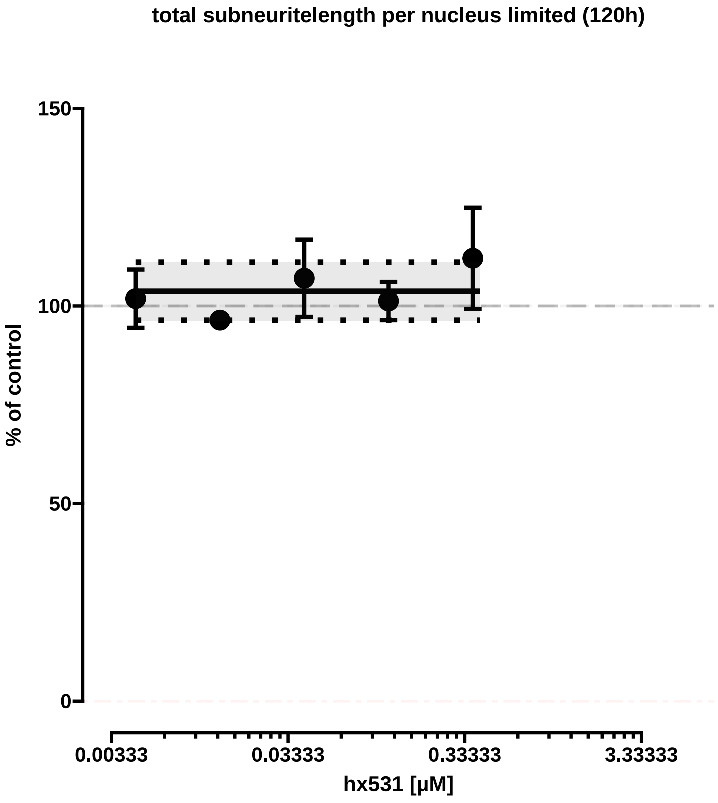

Model: 1-Parameter
Model abbr.: lm.1
Bechmark-Response (BMR): 20

BMCL: NA
BMC: NA
BMCU: NA

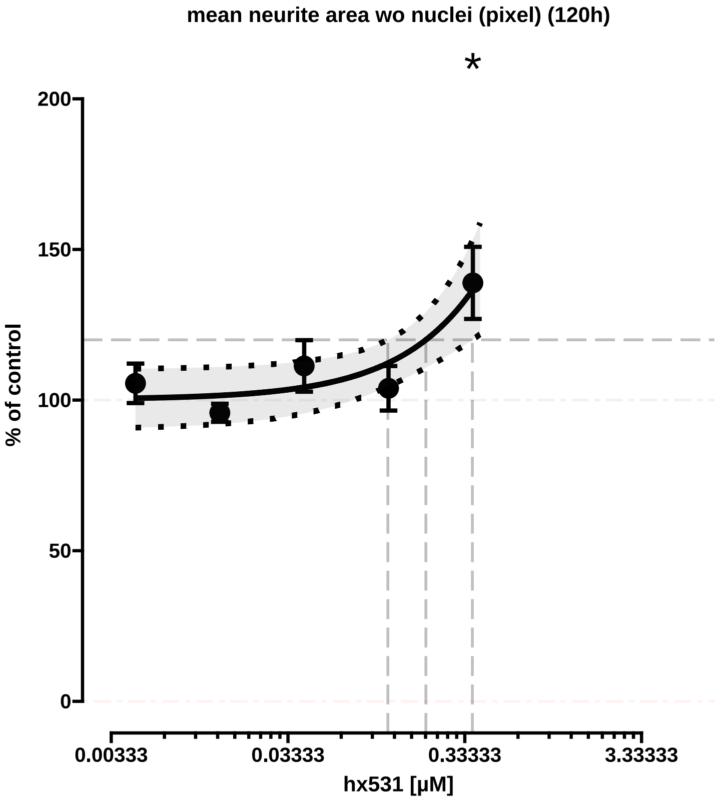

Model: Linear
Model abbr.: lm
Bechmark-Response (BMR): 20

BMCL: 0.122
BMC: 0.201
BMCU: 0.368

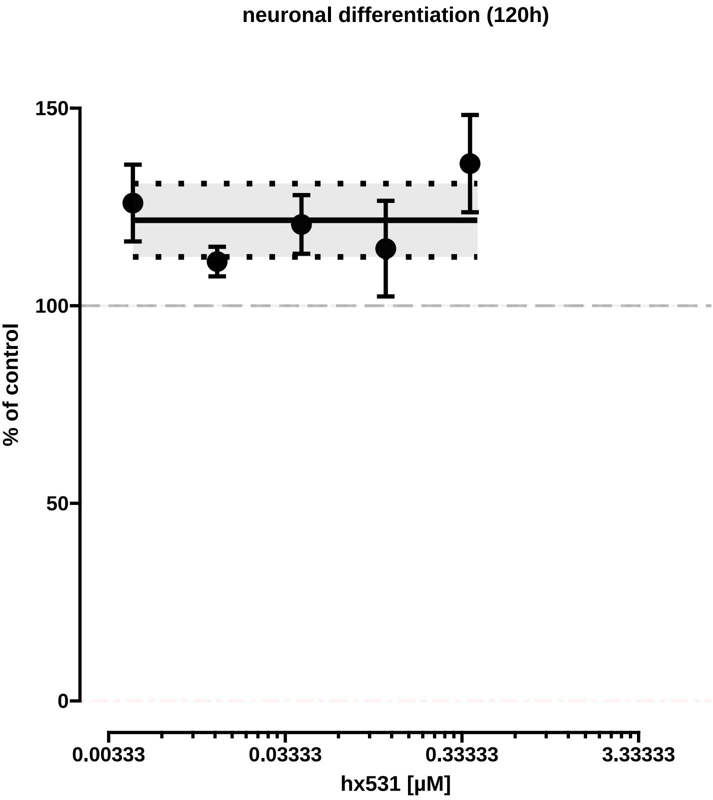

Model: 1-Parameter
Model abbr.: lm.1
Bechmark-Response (BMR): 25

BMCL: NA
BMC: NA
BMCU: NA

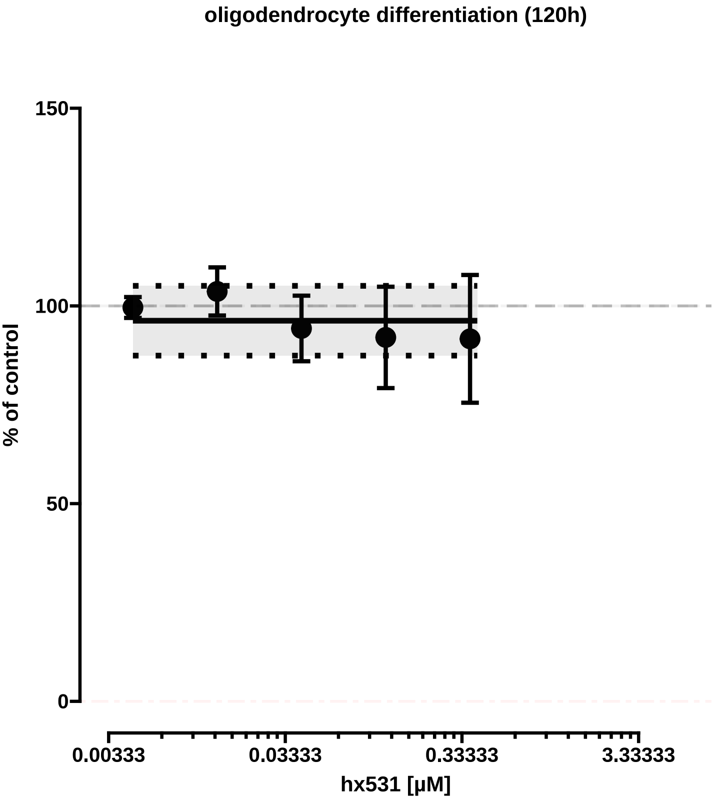

Model: 1-Parameter
Model abbr.: lm.1
Bechmark-Response (BMR): 25

BMCL: NA
BMC: NA
BMCU: NA

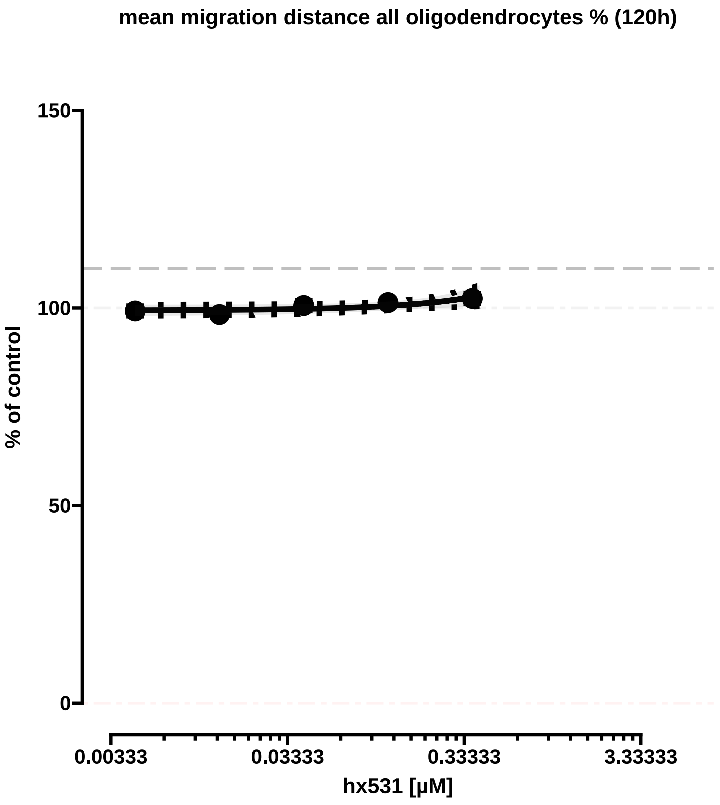

Model: Linear
Model abbr.: lm
Bechmark-Response (BMR): 10

BMCL: NA
BMC: NA
BMCU: NA

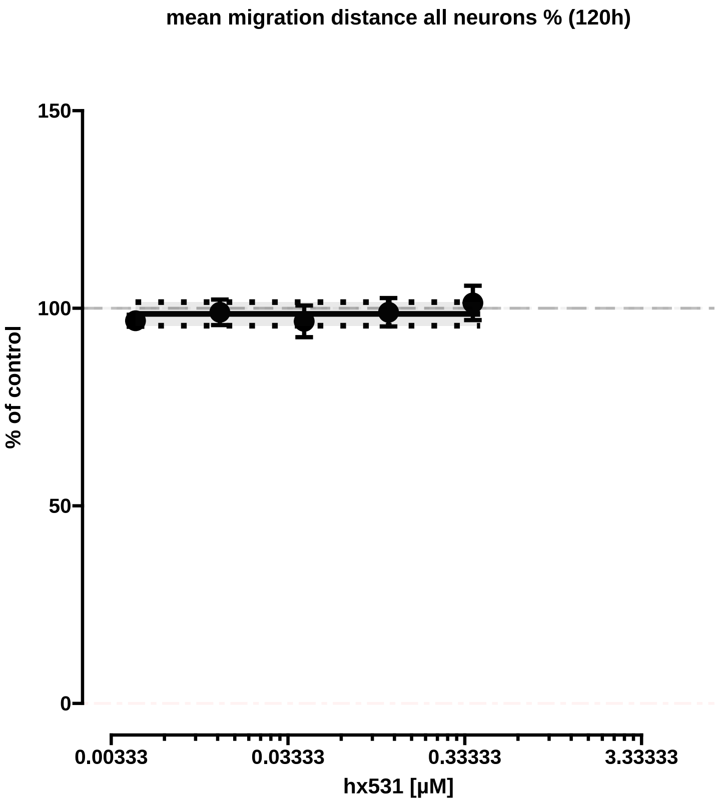

Model: 1-Parameter
Model abbr.: lm.1
Bechmark-Response (BMR): 10

BMCL: NA
BMC: NA
BMCU: NA

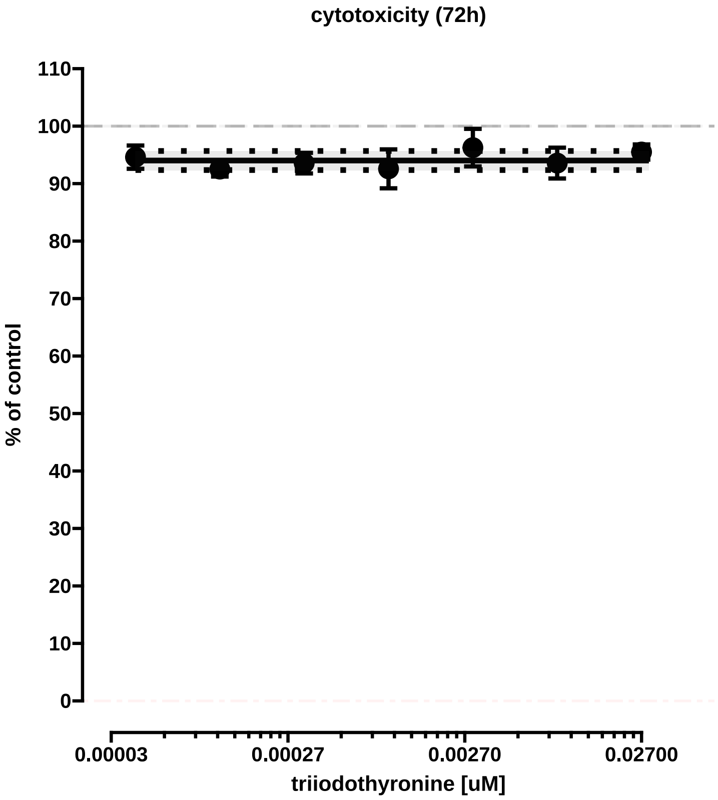

Model: 1-Parameter
Model abbr.: lm.1
Bechmark-Response (BMR): 10

BMCL: NA
BMC: NA
BMCU: NA

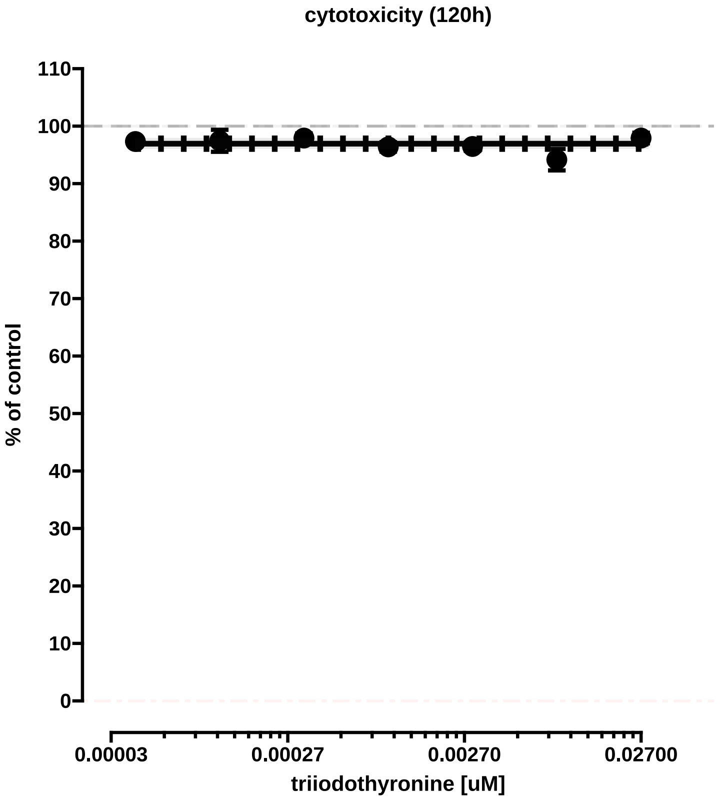

Model: 1-Parameter
Model abbr.: lm.1
Bechmark-Response (BMR): 10

BMCL: NA
BMC: NA
BMCU: NA

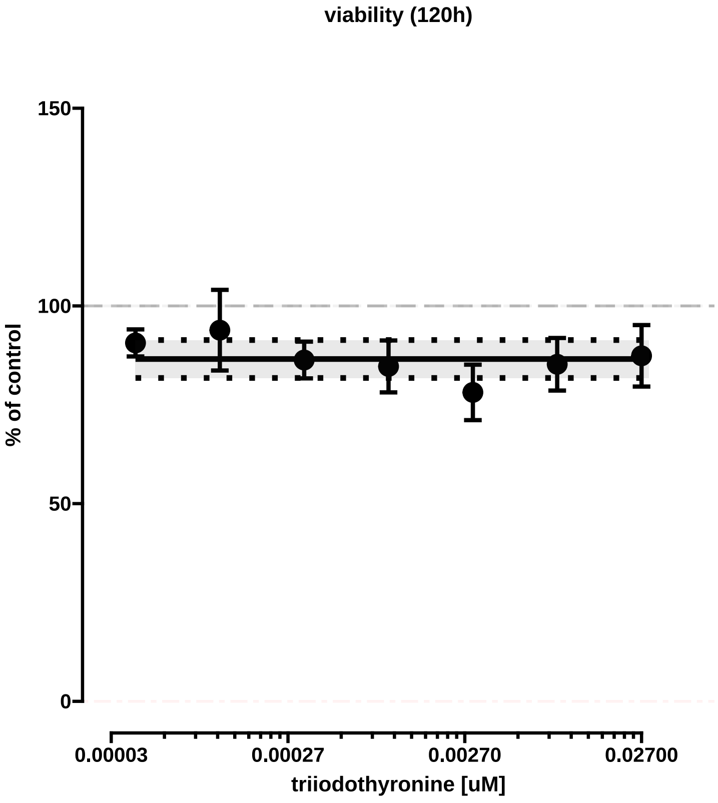

Model: 1-Parameter
Model abbr.: lm.1
Bechmark-Response (BMR): 20

BMCL: NA
BMC: NA
BMCU: NA

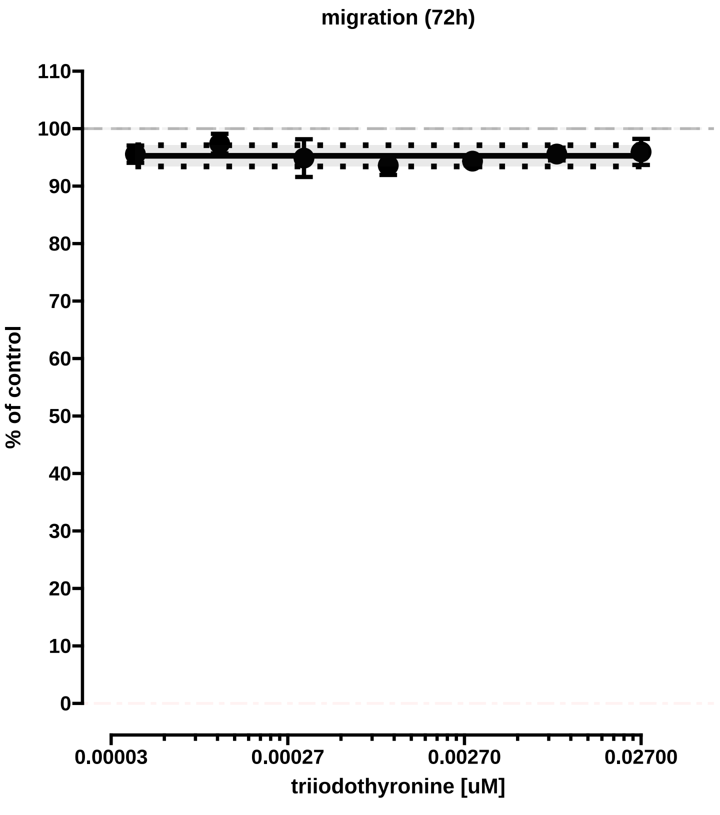

Model: 1-Parameter
Model abbr.: lm.1
Bechmark-Response (BMR): 10

BMCL: NA
BMC: NA
BMCU: NA

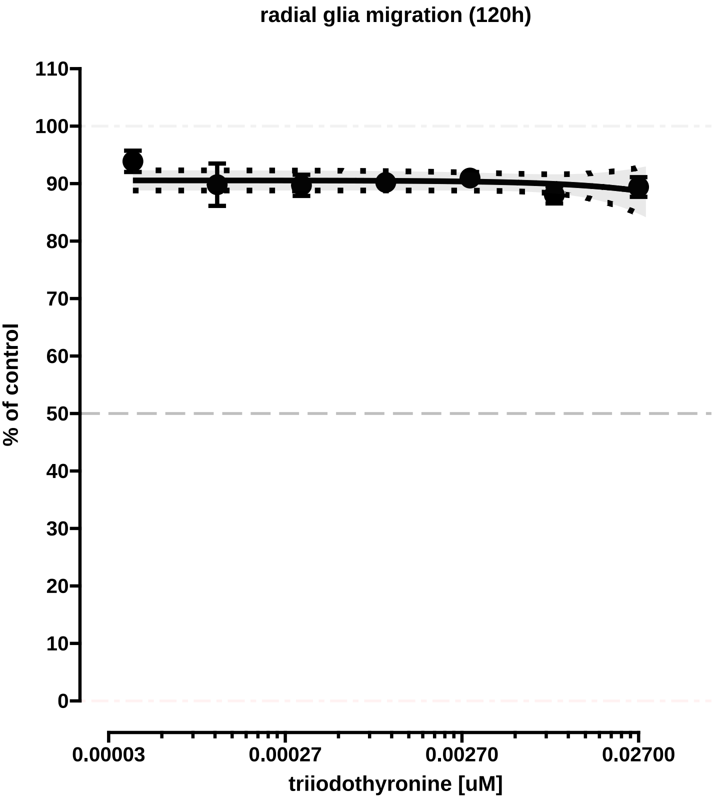

Model: Exponential decay with lower limit at 0
Model abbr.: EXD.2()
Bechmark-Response (BMR): 50

BMCL: NA
BMC: NA
BMCU: NA

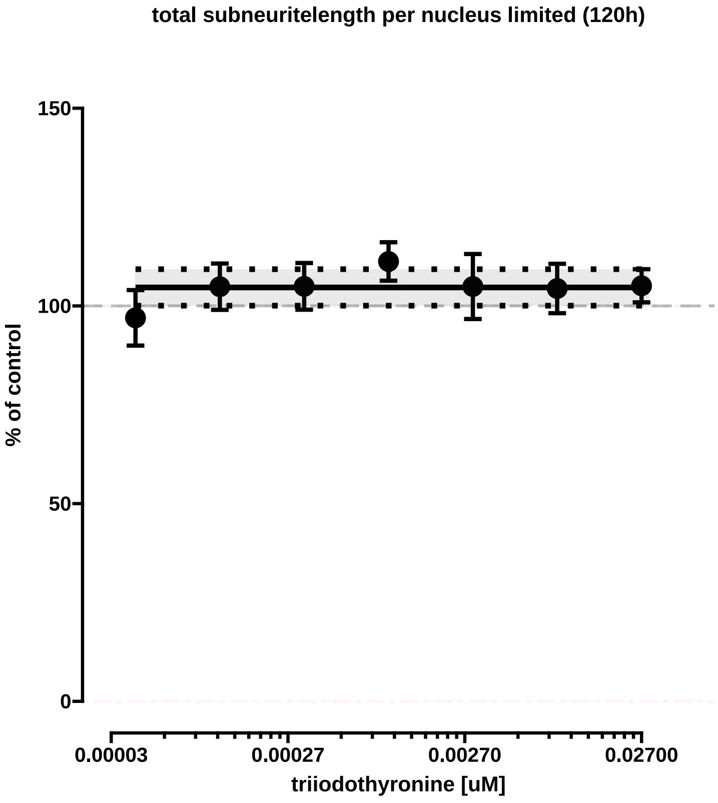

Model: 1-Parameter
Model abbr.: lm.1
Bechmark-Response (BMR): 20

BMCL: NA
BMC: NA
BMCU: NA

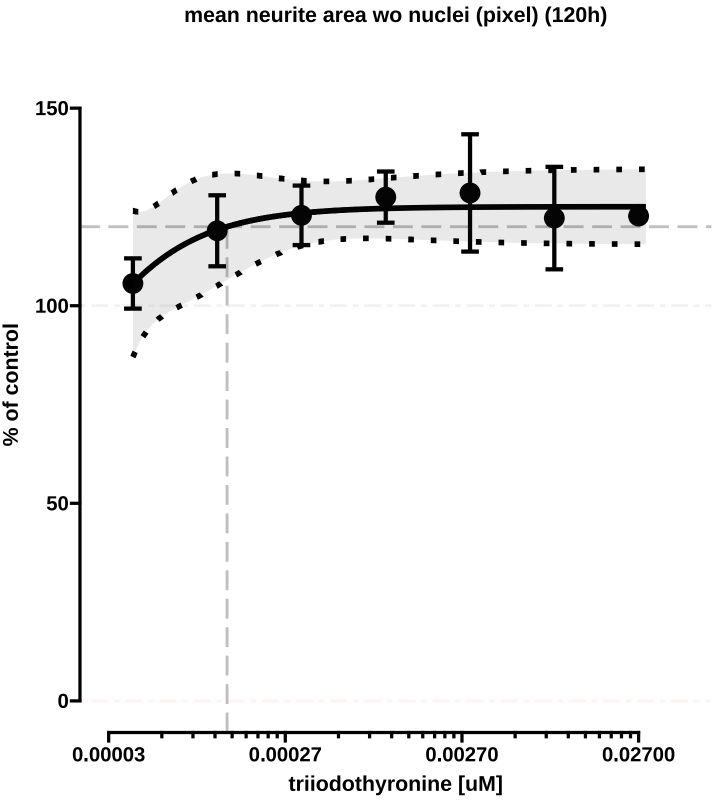

Model: Log-logistic (log(ED50) as parameter) with lower limit at 0
Model abbr.: LL2.3()
Bechmark-Response (BMR): 20

BMCL: NA
BMC: 0.00013
BMCU: NA

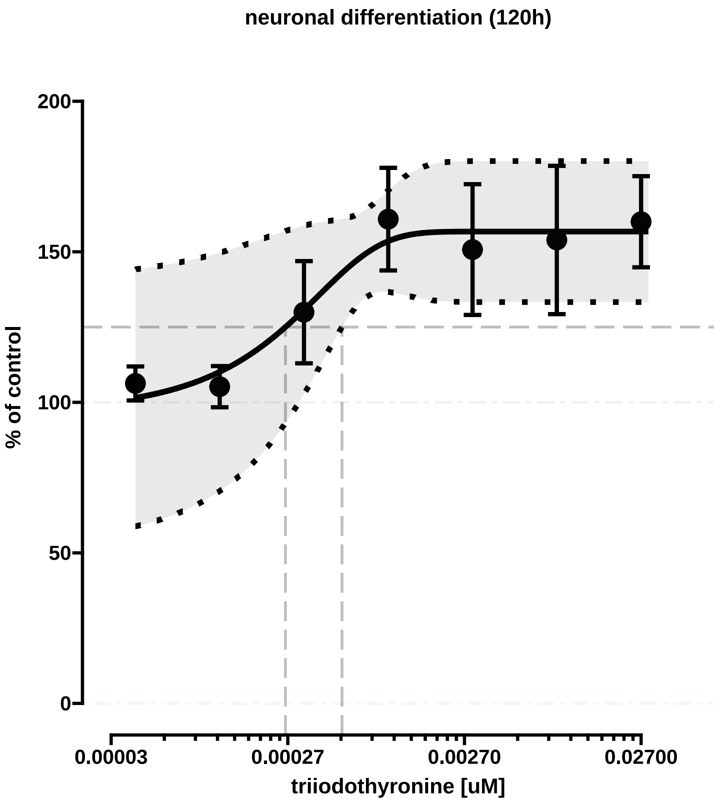

Model: Logistic (ED50 as parameter) with lower limit fixed at 0
Model abbr.: L.3()
Bechmark-Response (BMR): 25

BMCL: NA
BMC: 0.00026
BMCU: 0.001

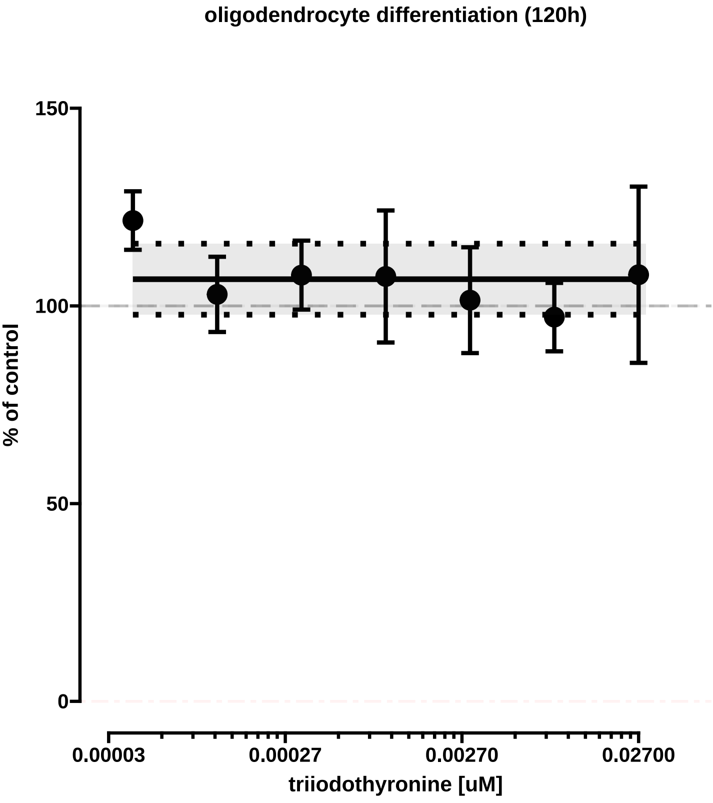

Model: 1-Parameter
Model abbr.: lm.1
Bechmark-Response (BMR): 25

BMCL: NA
BMC: NA
BMCU: NA

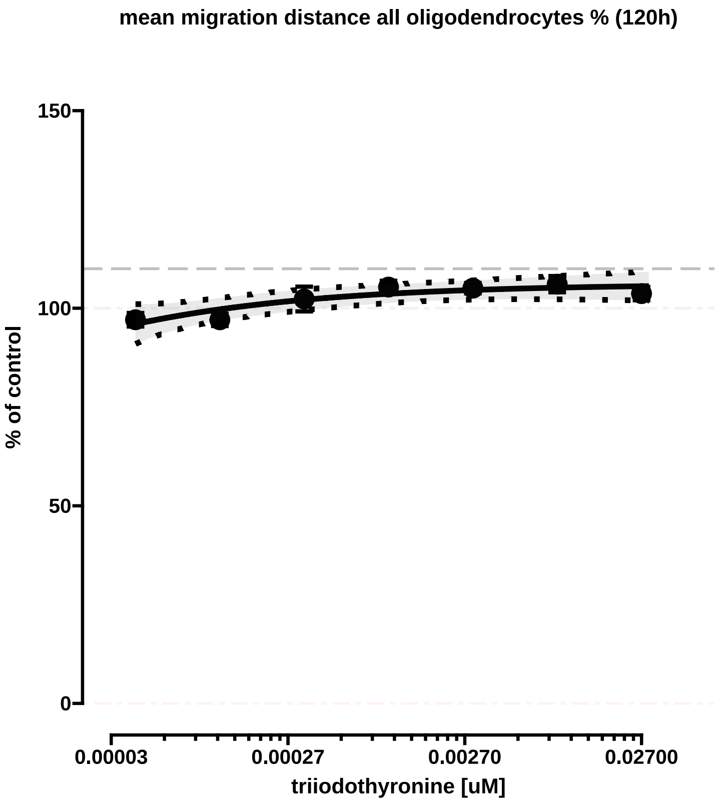

Model: Log-logistic (log(ED50) as parameter) with lower limit at 0
Model abbr.: LL2.3()
Bechmark-Response (BMR): 10

BMCL: NA
BMC: NA
BMCU: NA

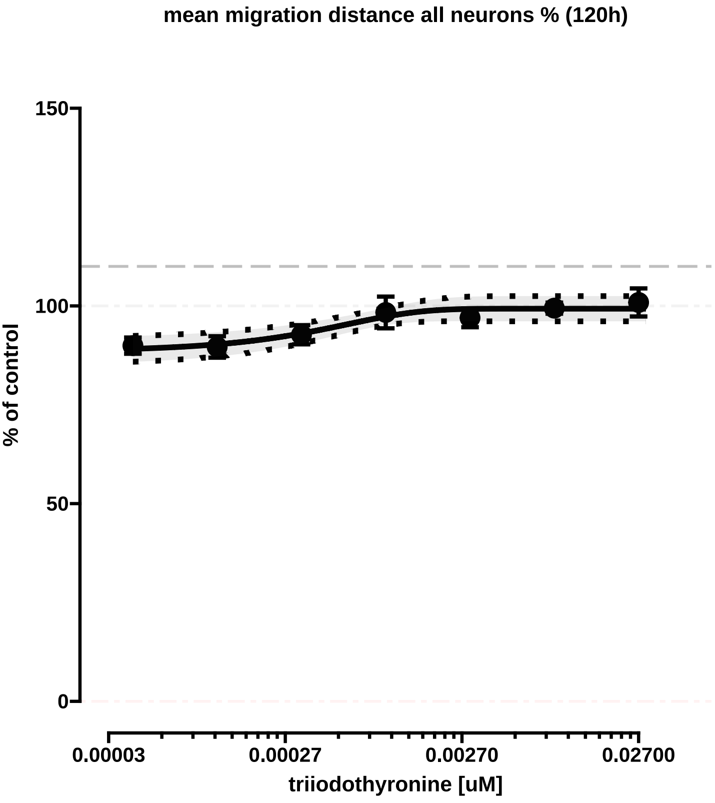

Model: Logistic (ED50 as parameter) with lower limit fixed at 0
Model abbr.: L.3()
Bechmark-Response (BMR): 10

BMCL: NA
BMC: NA
BMCU: NA

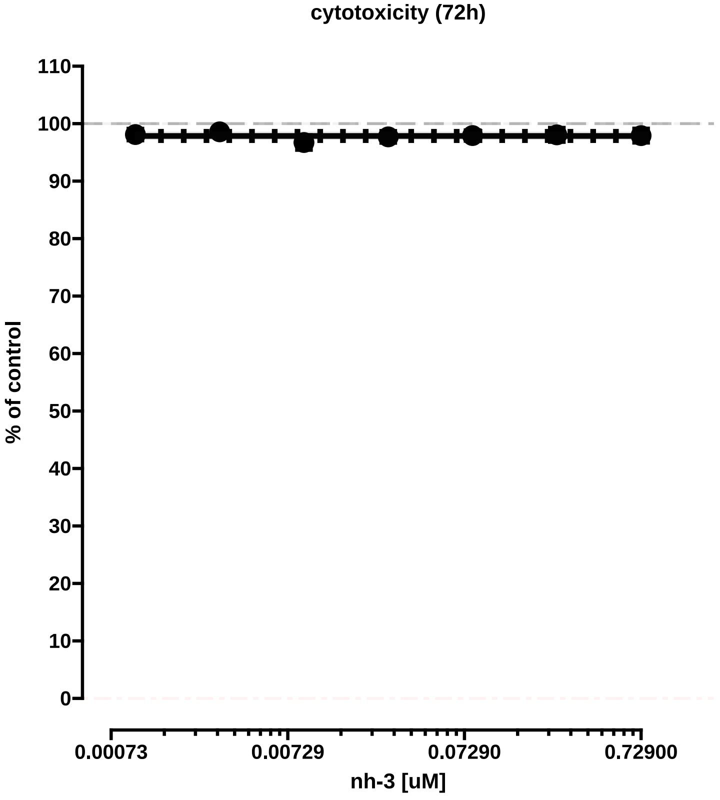

Model: 1-Parameter
Model abbr.: lm.1
Bechmark-Response (BMR): 10

BMCL: NA
BMC: NA
BMCU: NA

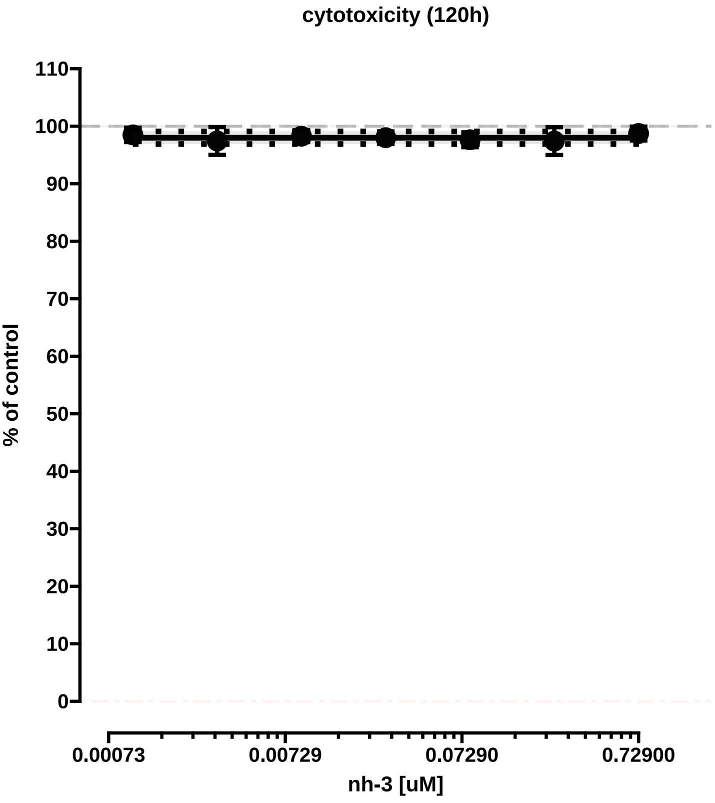

Model: 1-Parameter
Model abbr.: lm.1
Bechmark-Response (BMR): 10

BMCL: NA
BMC: NA
BMCU: NA

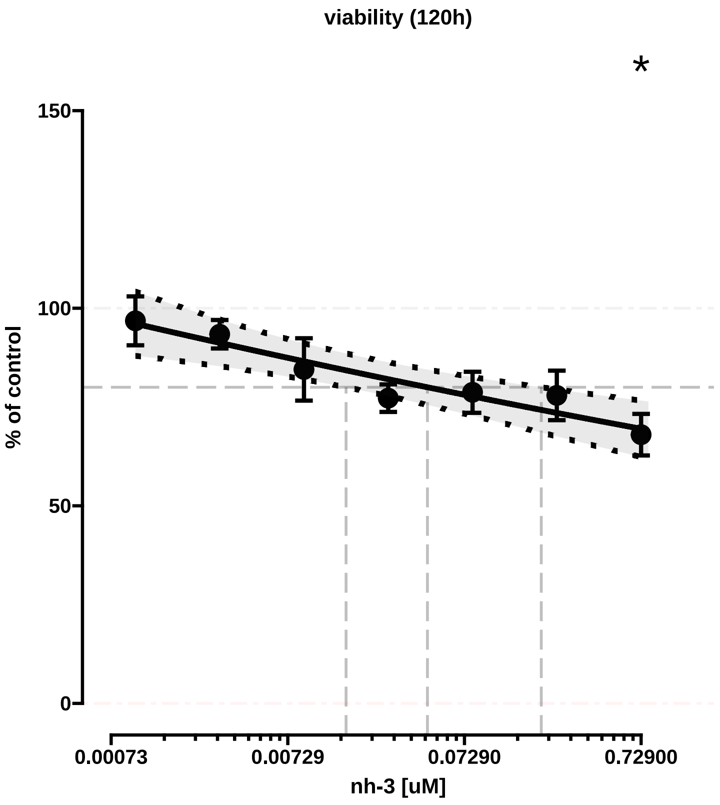

Model: Log-logistic (log(ED50) as parameter) with lower limit at 0
Model abbr.: LL2.3()
Bechmark-Response (BMR): 20

BMCL: 0.016
BMC: 0.045
BMCU: 0.198

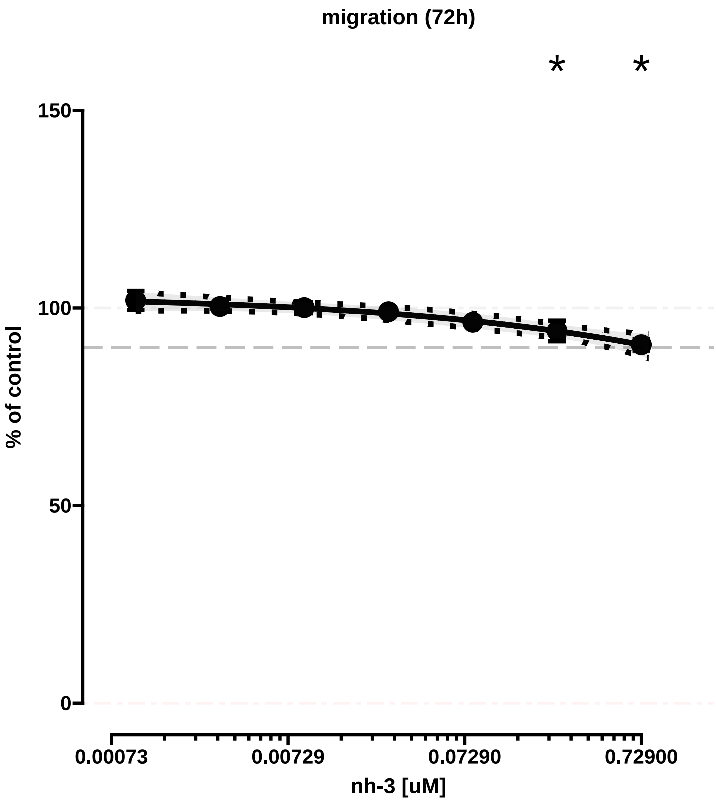

Model: Log-logistic (log(ED50) as parameter) with lower limit at 0
Model abbr.: LL2.3()
Bechmark-Response (BMR): 10

BMCL: NA
BMC: NA
BMCU: NA

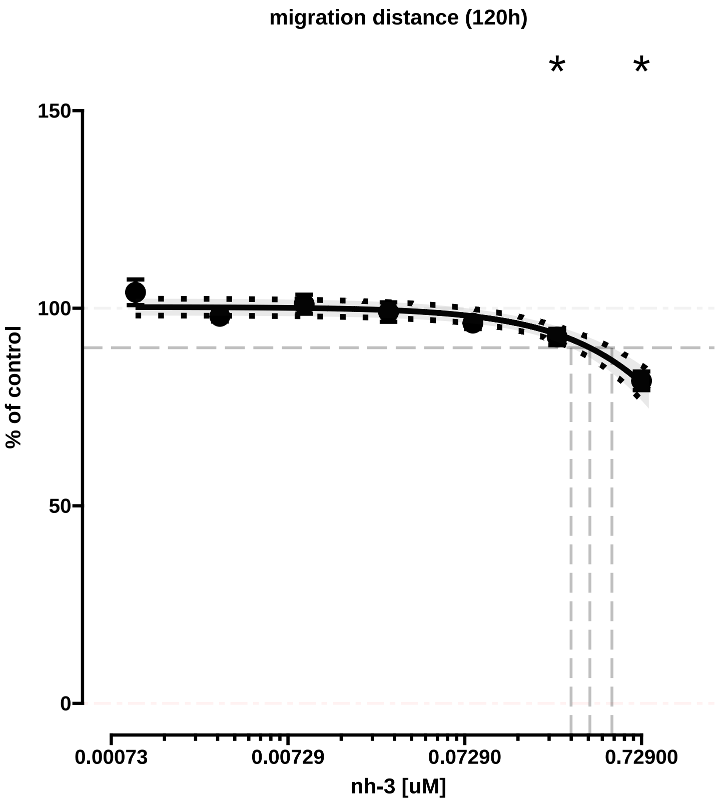

Model: Exponential decay with lower limit at 0
Model abbr.: EXD.2()
Bechmark-Response (BMR): 10

BMCL: 0.291
BMC: 0.372
BMCU: 0.496

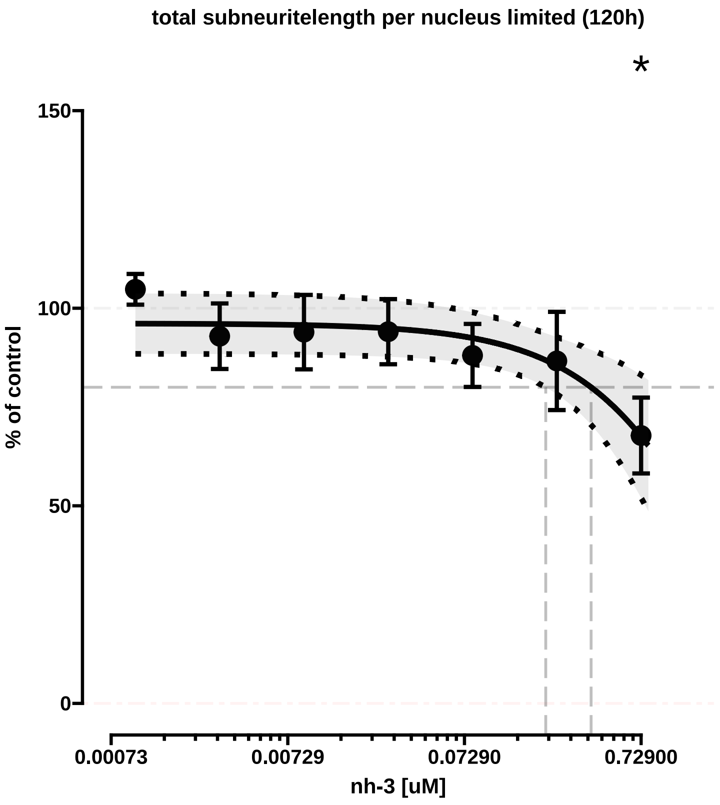

Model: Exponential decay with lower limit at 0
Model abbr.: EXD.2()
Bechmark-Response (BMR): 20

BMCL: 0.21
BMC: 0.38
BMCU: NA

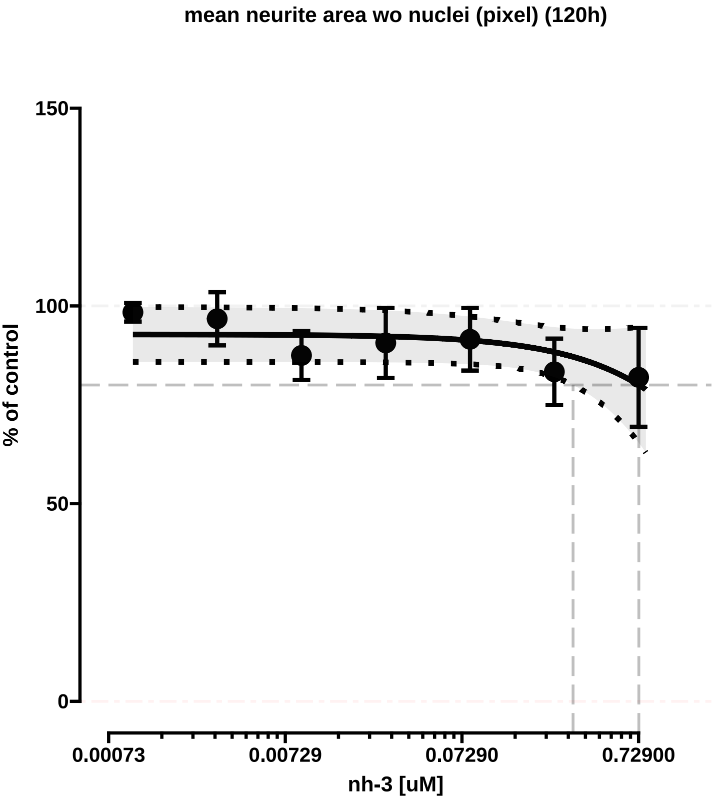

Model: Exponential decay with lower limit at 0
Model abbr.: EXD.2()
Bechmark-Response (BMR): 20

BMCL: 0.31
BMC: 0.731
BMCU: NA

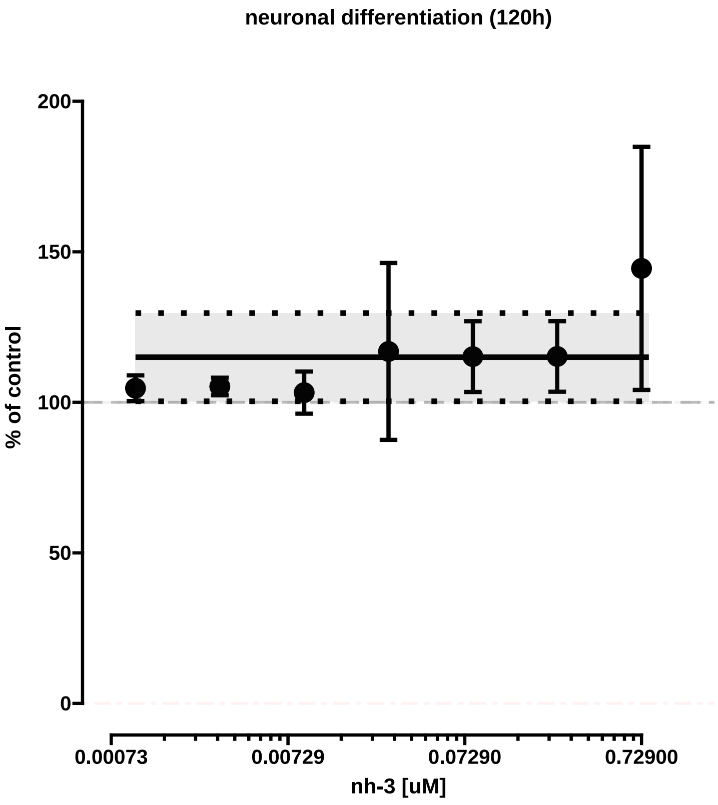

Model: 1-Parameter
Model abbr.: lm.1
Bechmark-Response (BMR): 25

BMCL: NA
BMC: NA
BMCU: NA

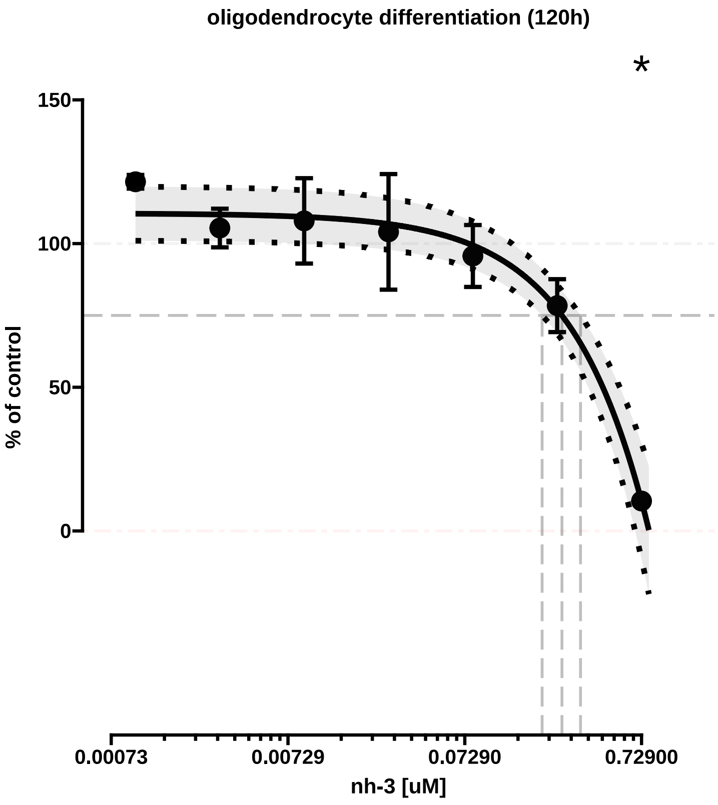

Model: Linear
Model abbr.: lm
Bechmark-Response (BMR): 25

BMCL: 0.2
BMC: 0.258
BMCU: 0.329

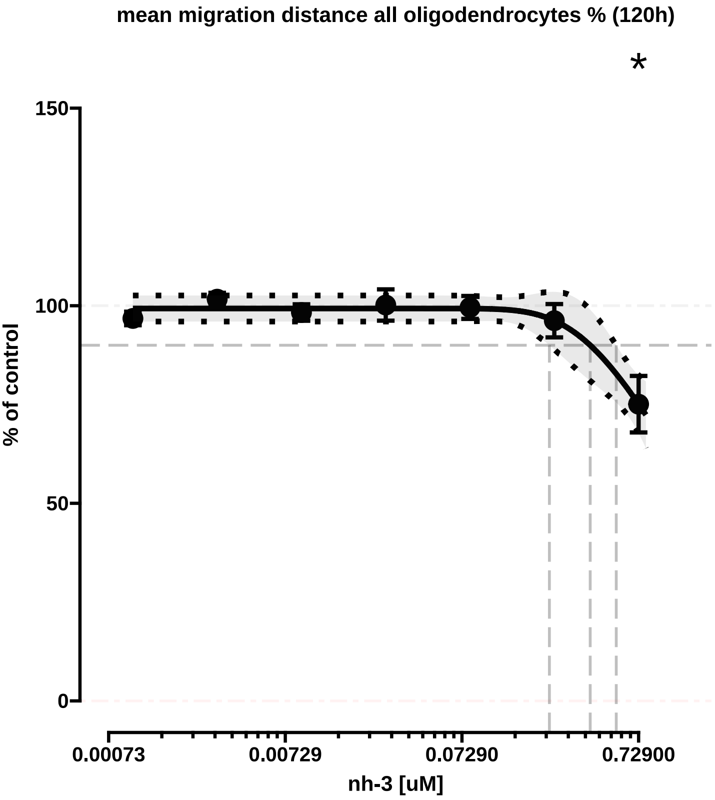

Model: Weibull (type 2) with lower limit at 0
Model abbr.: W2.3()
Bechmark-Response (BMR): 10

BMCL: 0.228
BMC: 0.388
BMCU: 0.545

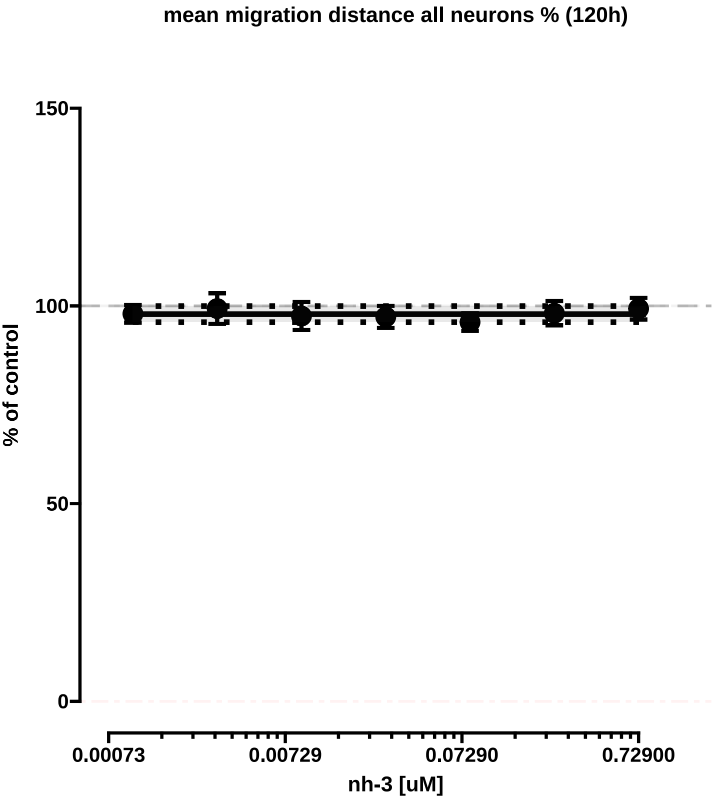

Model: 1-Parameter
Model abbr.: lm.1
Bechmark-Response (BMR): 10

BMCL: NA
BMC: NA
BMCU: NA

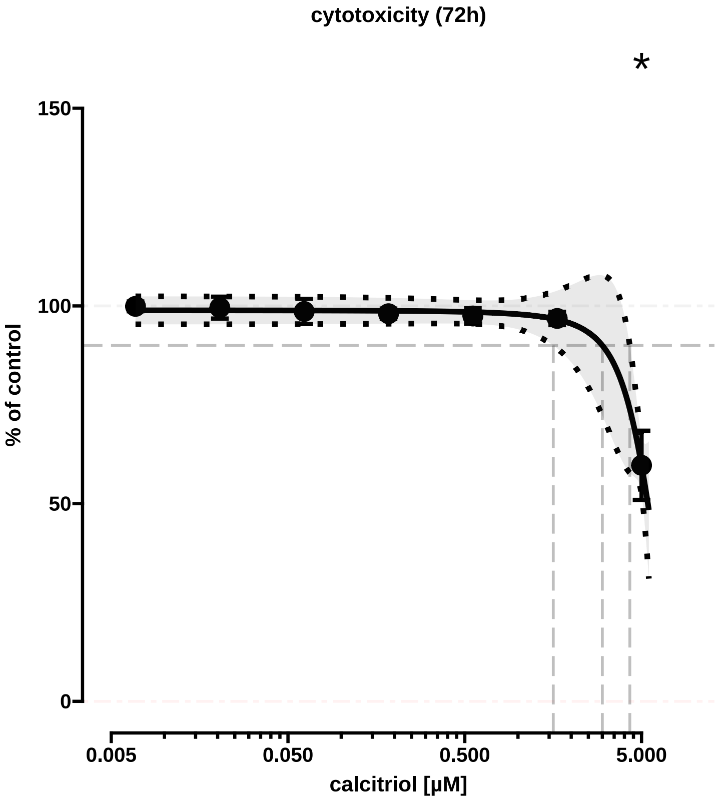

Model: Logistic (ED50 as parameter) with lower limit fixed at 0
Model abbr.: L.3()
Bechmark-Response (BMR): 10

BMCL: 1.583
BMC: 3.001
BMCU: 4.291

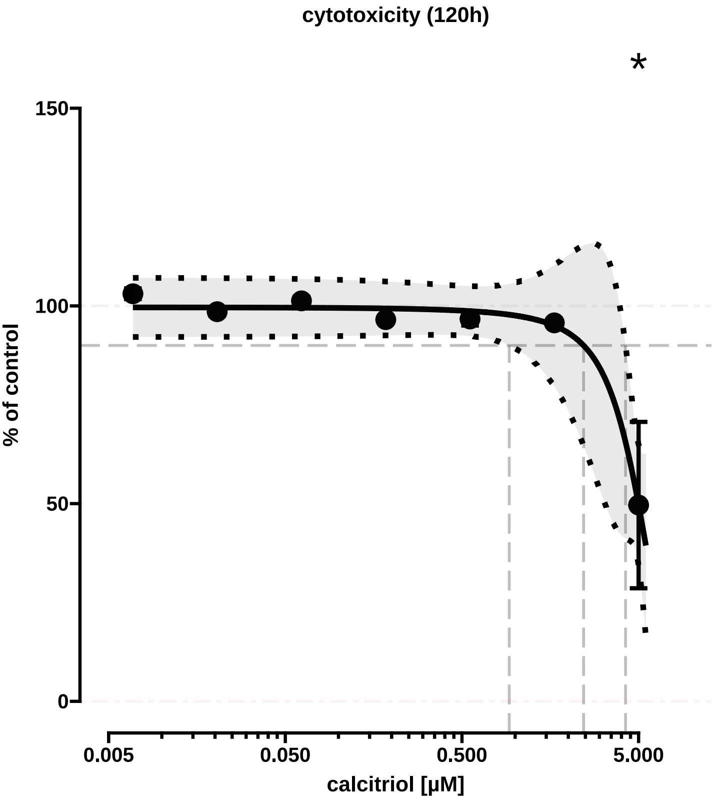

Model: Logistic (ED50 as parameter) with lower limit fixed at 0
Model abbr.: L.3()
Bechmark-Response (BMR): 10

BMCL: 0.926
BMC: 2.44
BMCU: 4.218

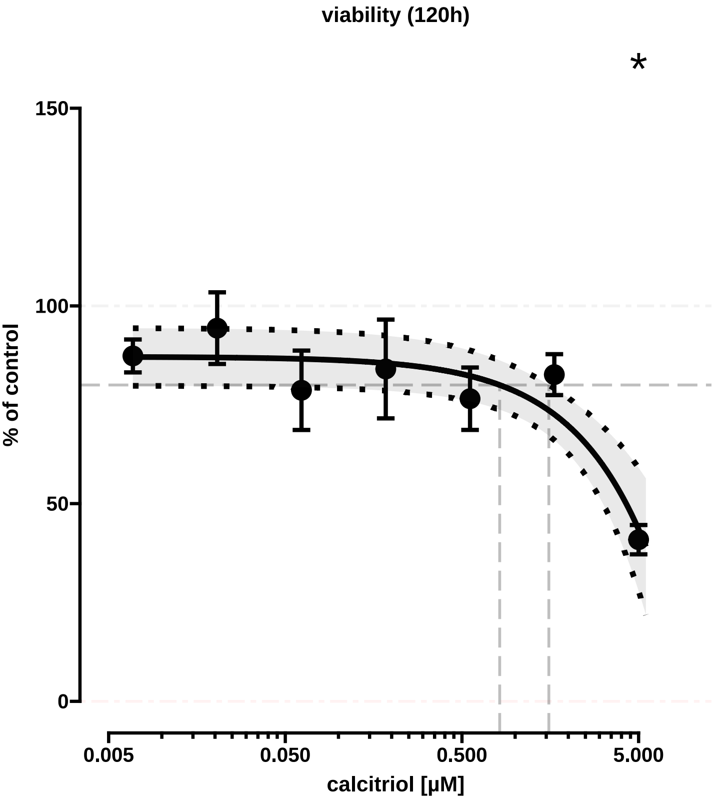

Model: Linear
Model abbr.: lm
Bechmark-Response (BMR): 20

BMCL: NA
BMC: 0.818
BMCU: 1.552

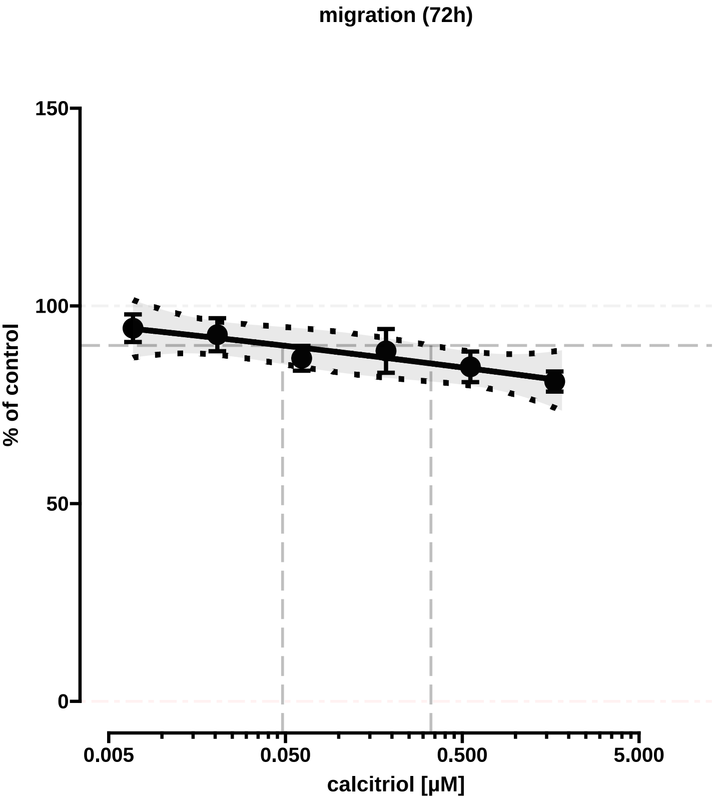

Model: Log-logistic (log(ED50) as parameter) with lower limit at 0
Model abbr.: LL2.3()
Bechmark-Response (BMR): 10

BMCL: NA
BMC: 0.048
BMCU: 0.332

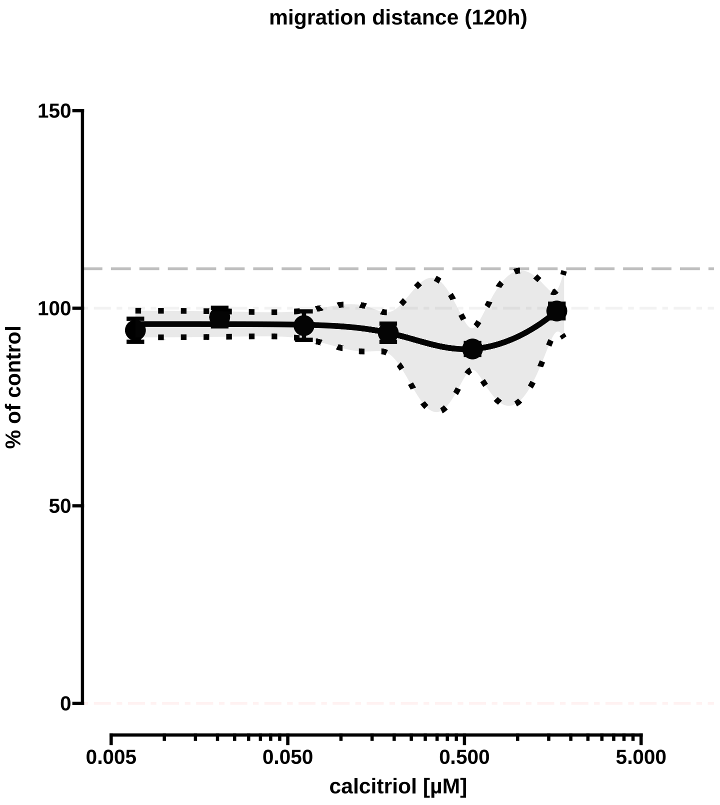

Model: Brain-Cousens (hormesis)
Model abbr.: BC.5()
Bechmark-Response (BMR): 10

BMCL: NA
BMC: NA
BMCU: NA

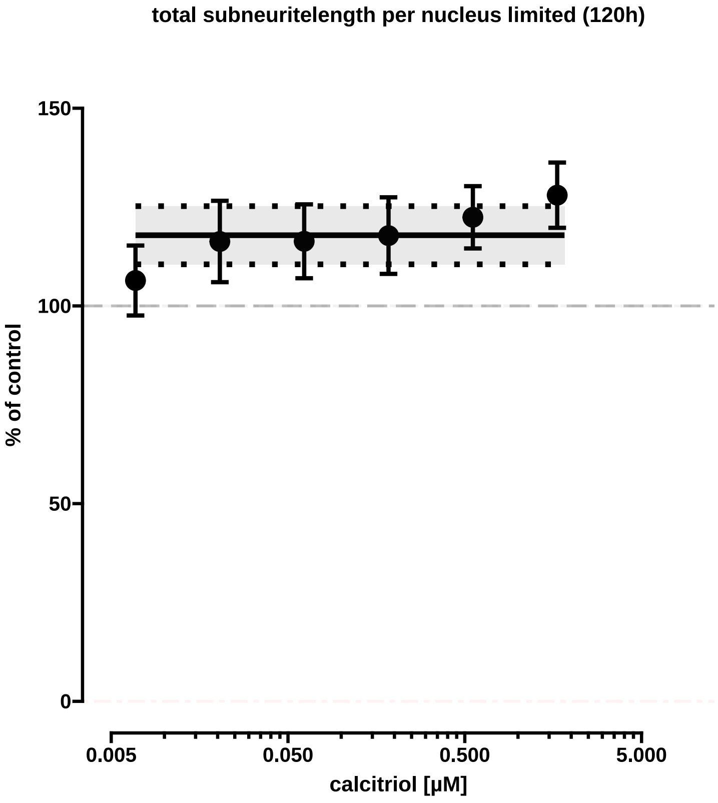

Model: 1-Parameter
Model abbr.: lm.1
Bechmark-Response (BMR): 20

BMCL: NA
BMC: NA
BMCU: NA

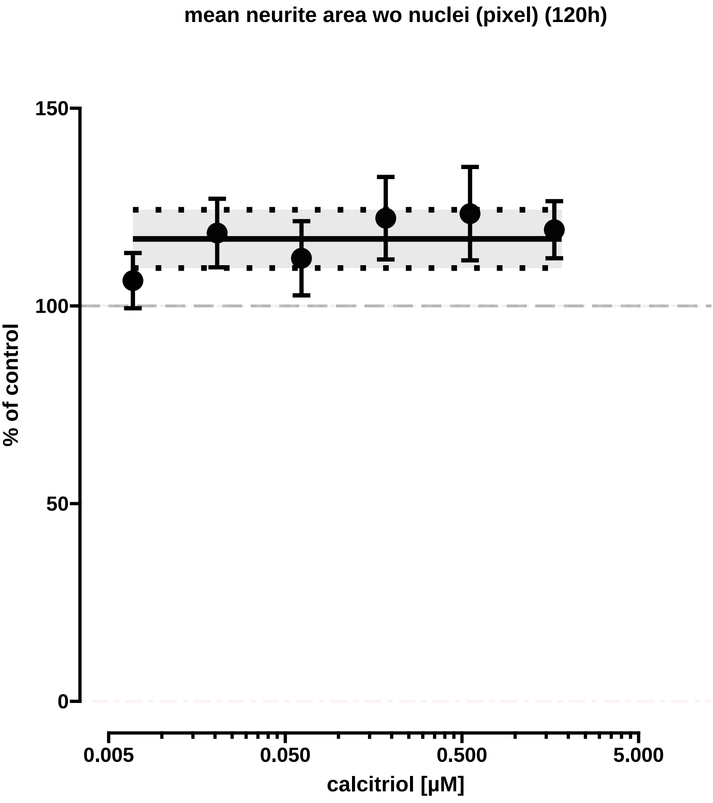

Model: 1-Parameter
Model abbr.: lm.1
Bechmark-Response (BMR): 20

BMCL: NA
BMC: NA
BMCU: NA

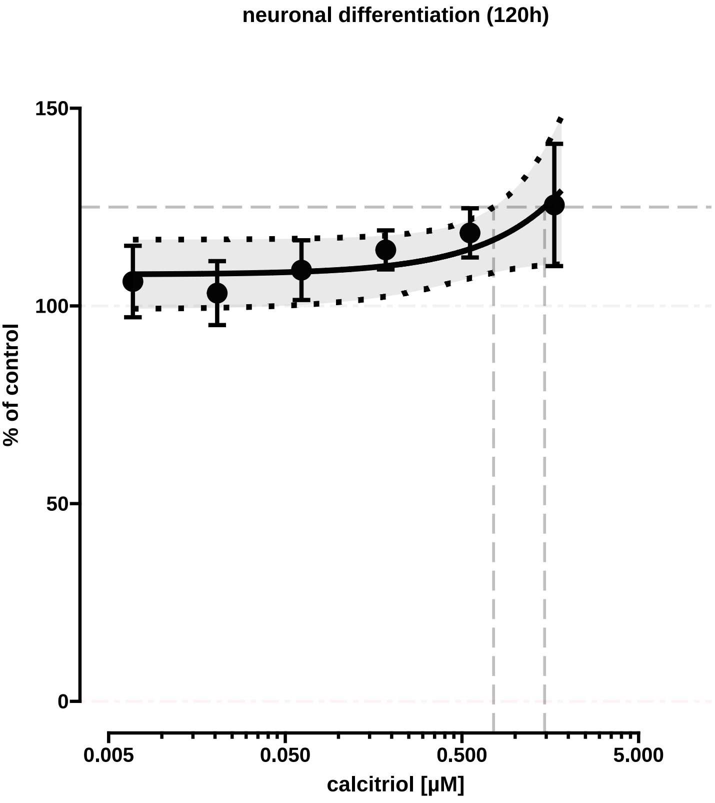

Model: Linear
Model abbr.: lm
Bechmark-Response (BMR): 25

BMCL: 0.755
BMC: 1.469
BMCU: NA

oligodendrocyte differentiation (120h)

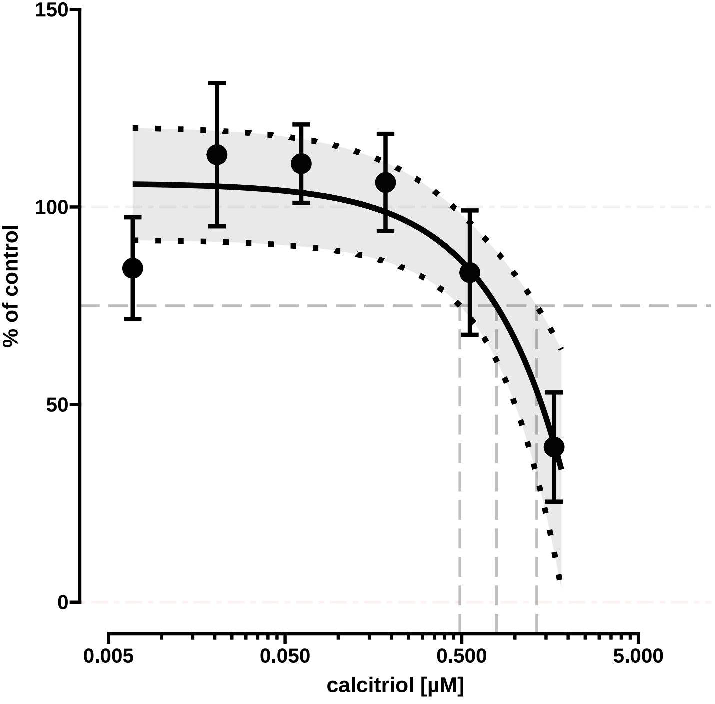

Model: Linear
Model abbr.: lm
Bechmark-Response (BMR): 25

BMCL: 0.488
BMC: 0.786
BMCU: 1.33

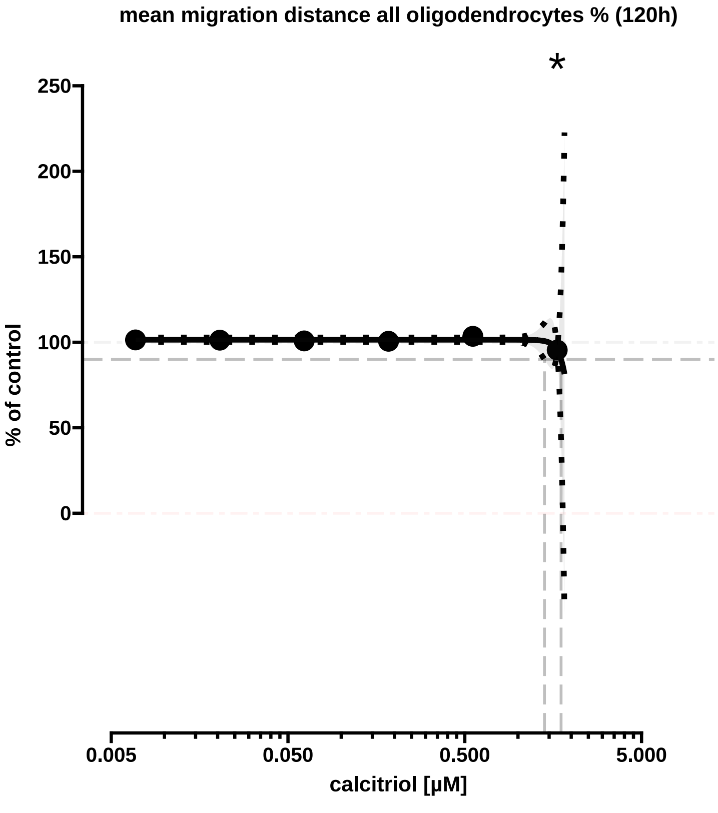

Model: Logistic (ED50 as parameter) with lower limit fixed at 0
Model abbr.: L.3()
Bechmark-Response (BMR): 10

BMCL: 1.412
BMC: 1.753
BMCU: NA

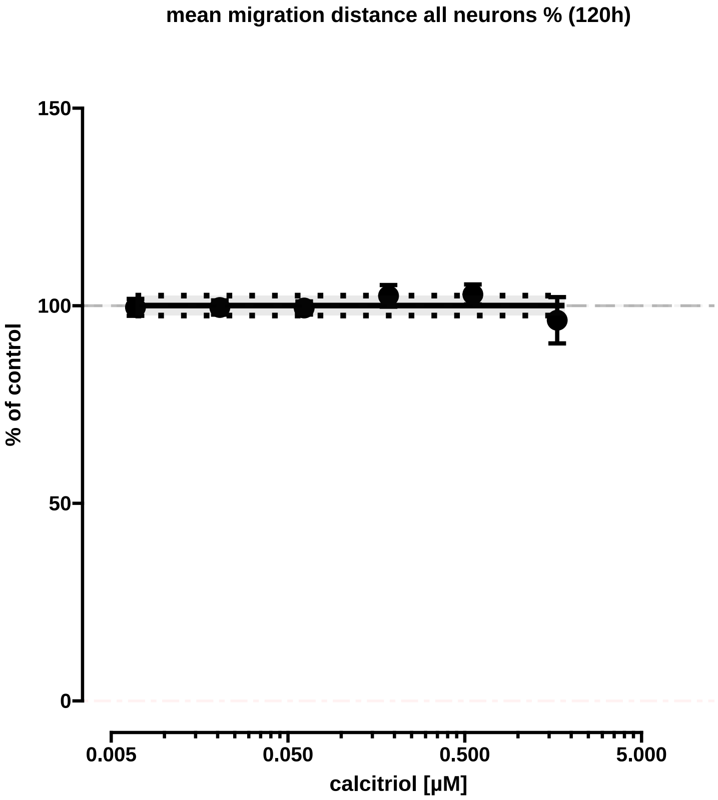

Model: 1-Parameter
Model abbr.: lm.1
Bechmark-Response (BMR): 10

BMCL: NA
BMC: NA
BMCU: NA
