# Supplementary material for: Nuclear hormone receptors control fundamental processes of human fetal neurodevelopment: Basis for endocrine disruption assessment
Source: Environ Int. Author manuscript; Available in PMC 2025 Jun 2. (PMC12127433; doi:10.1016/j.envint.2025.109400)
Supplement: 11 [file NIHMS2077722-supplement-11.pdf]

proliferation (brdu) (72h)

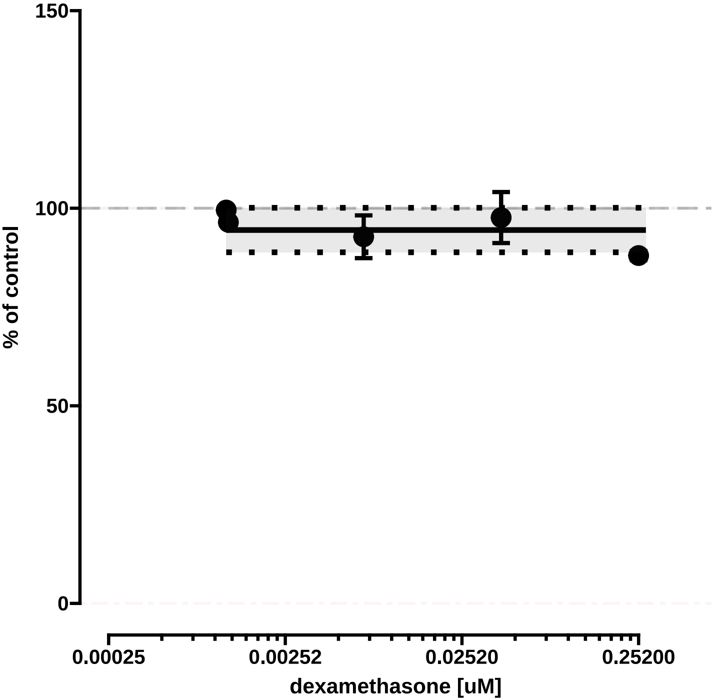

Model: 1-Parameter
Model abbr.: lm.1
Bechmark-Response (BMR): 20

BMCL: NA
BMC: NA
BMCU: NA

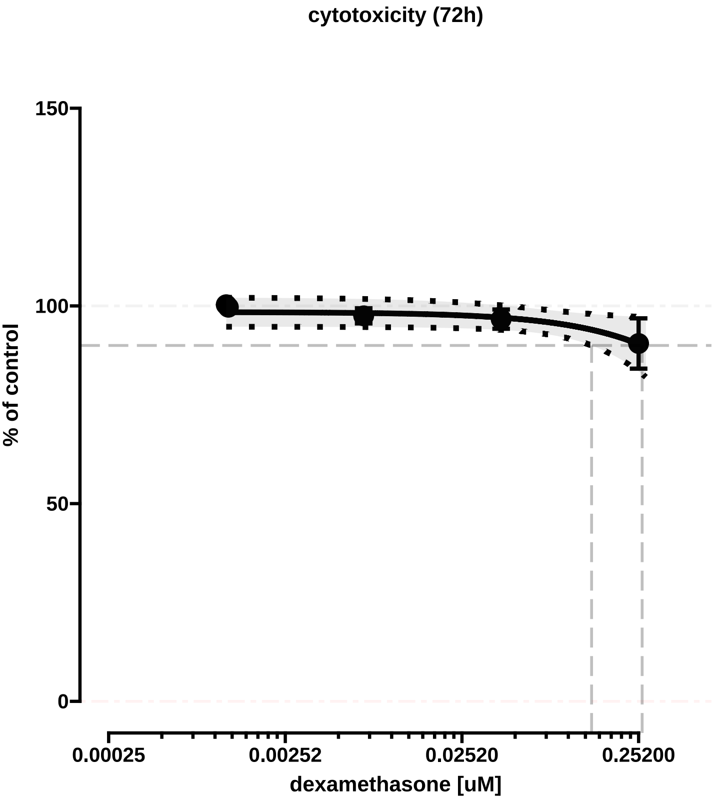

Model: Exponential decay with lower limit at 0
Model abbr.: EXD.2()
Bechmark-Response (BMR): 10

BMCL: 0.137
BMC: 0.264
BMCU: NA

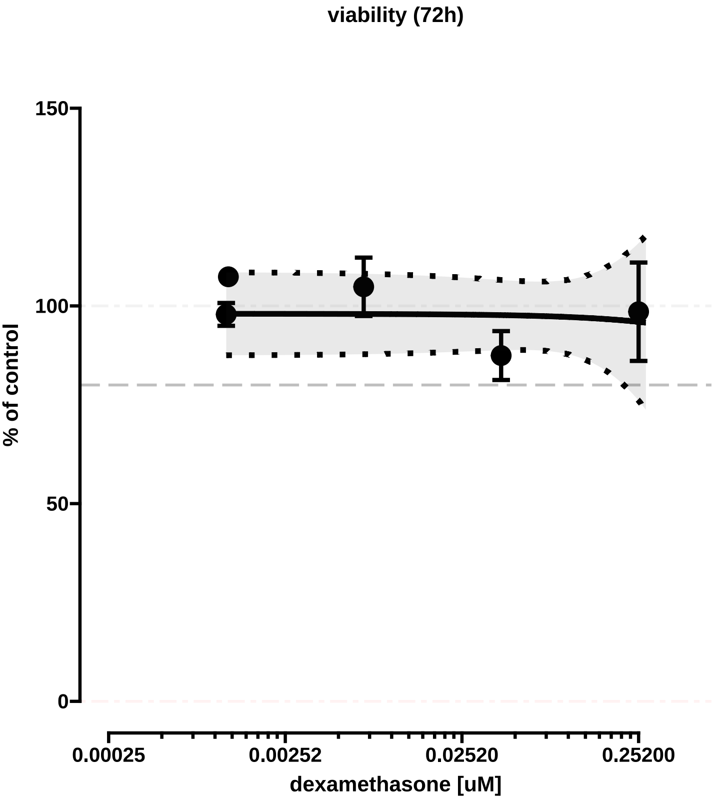

Model: Exponential decay with lower limit at 0
Model abbr.: EXD.2()
Bechmark-Response (BMR): 20

BMCL: NA
BMC: NA
BMCU: NA

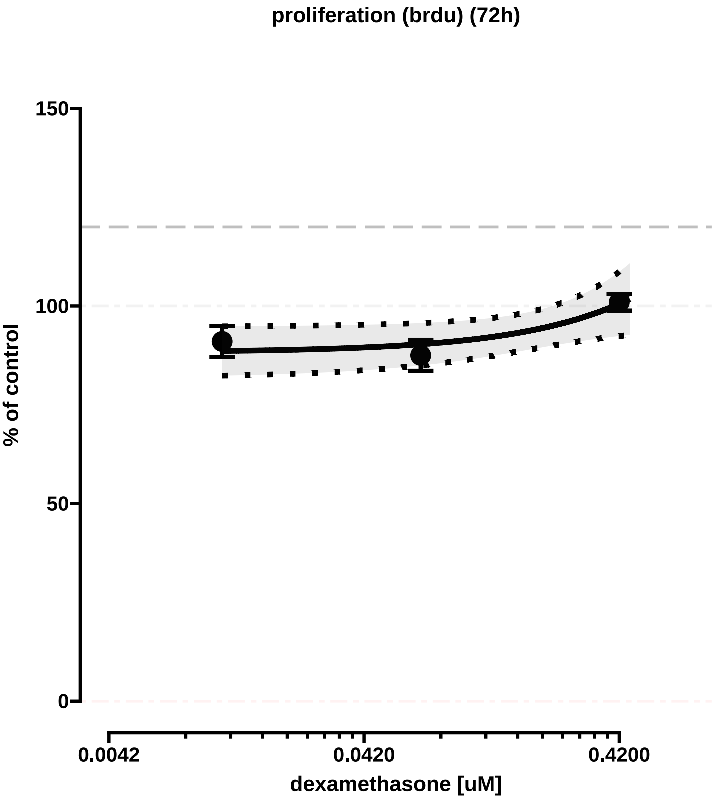

Model: Linear
Model abbr.: lm
Bechmark-Response (BMR): 20

BMCL: NA
BMC: NA
BMCU: NA

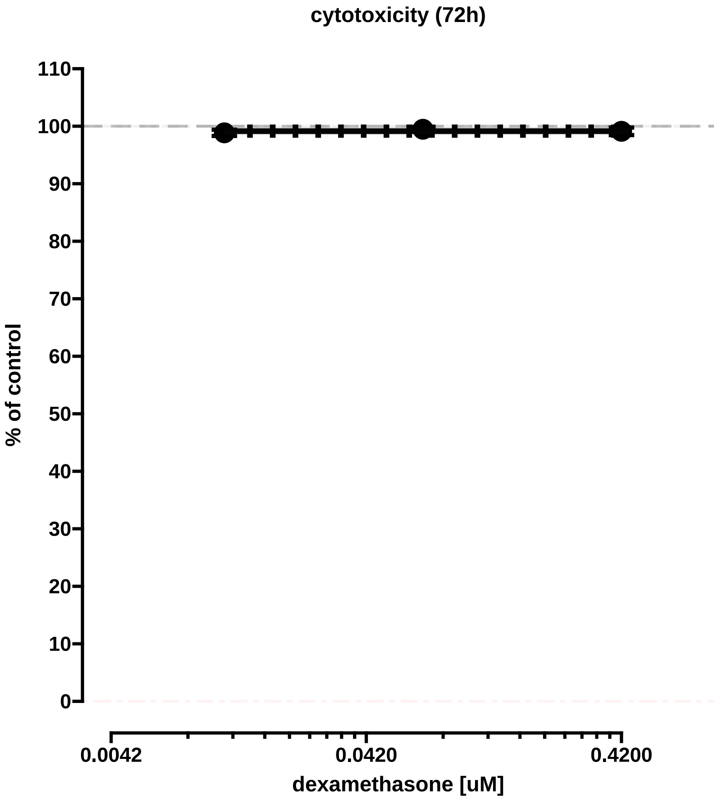

Model: 1-Parameter
Model abbr.: lm.1
Bechmark-Response (BMR): 10

BMCL: NA
BMC: NA
BMCU: NA

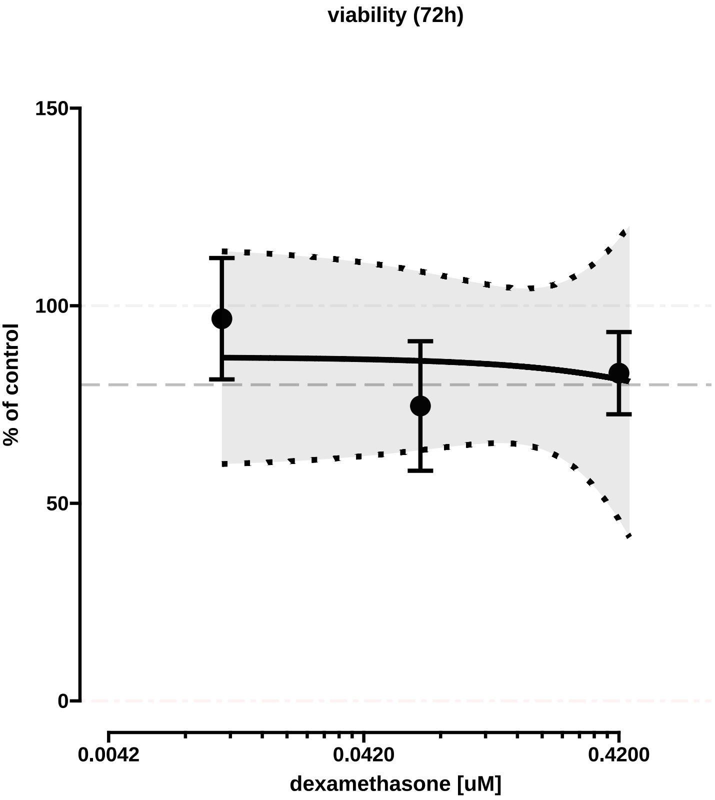

Model: Exponential decay with lower limit at 0
Model abbr.: EXD.2()
Bechmark-Response (BMR): 20

BMCL: NA
BMC: NA
BMCU: NA

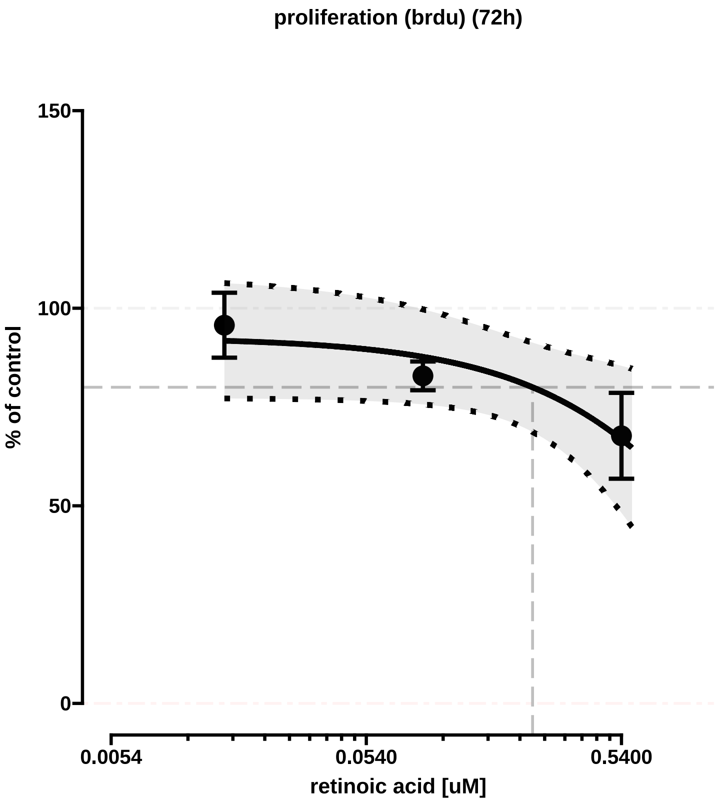

Model: Exponential decay with lower limit at 0
Model abbr.: EXD.2()
Bechmark-Response (BMR): 20

BMCL: NA
BMC: 0.242
BMCU: NA

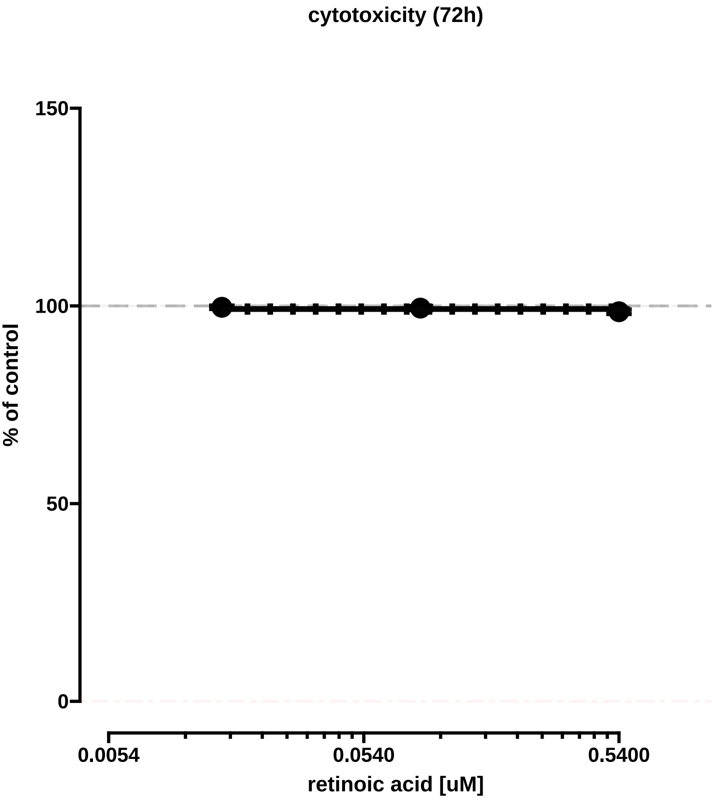

Model: 1-Parameter
Model abbr.: lm.1
Bechmark-Response (BMR): 10

BMCL: NA
BMC: NA
BMCU: NA

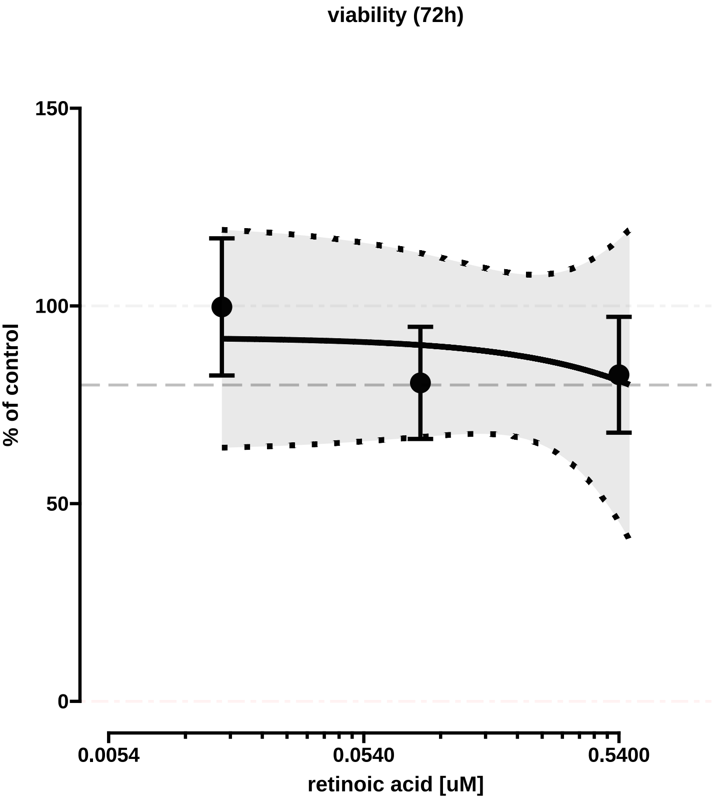

Model: Exponential decay with lower limit at 0
Model abbr.: EXD.2()
Bechmark-Response (BMR): 20

BMCL: NA
BMC: NA
BMCU: NA
